# Multi-Matrix Assessment of Potentially Toxic Elements Across Soil and *Sambucus nigra* Organs: Spatial Patterns, Regulatory Compliance, and Dietary Exposure of Edible Fruits

**DOI:** 10.3390/foods15193573

**Published:** 2026-10-08

**Authors:** Călin Gheorghe Pășcuț, Szilárd Bartha, Voichița Timiş-Gânsac, Adrian Tunduc, Călin Ioan Iovan, Petrică Tudor Moțiu, Ioana Andra Vlad, Praveen Pandey, Anamaria Călugăr, Claudiu Ioan Bunea, Florin Dumitru Bora

**Affiliations:** 1 Department of Forestry and Forest Engineering, University of Oradea, 1 University Street, 410087 Oradea, Romania; cpascut@uoradea.ro (C.G.P.); sbartha@uoradea.ro (S.B.); vtimis@uoradea.ro (V.T.-G.); atunduc@uoradea.ro (A.T.); calin.iovan@uoradea.ro (C.I.I.); 2 Department of Food Engineering, University of Oradea, 1 University Street, 410087 Oradea, Romania; iavlad@uoradea.ro; 3 Division of Plant Breeding and Genetic Resource Conservation, CSIR- Central Institute of Medicinal and Aromatic Plants, Lucknow 226015, India; pandeypraveen1986@yahoo.com; 4 Viticulture and Oenology Department, Advanced Horticultural Research Institute of Transylvania, Faculty of Horticulture and Business in Rural Development, University of Agricultural Science and Veterinary Medicine of Cluj-Napoca, 3–5 Mănăştur Street, 400372 Cluj-Napoca, Romania; anamaria.calugar@usamvcluj.ro (A.C.); claudiu.bunea@usamvcluj.ro (C.I.B.); 5 Laboratory of Chromatography, Advanced Horticultural Research Institute of Transylvania, Faculty of Horticulture and Business in Rural Development, University of Agricultural Science and Veterinary Medicine of Cluj-Napoca, 400372 Cluj-Napoca, Romania

**Keywords:** *Sambucus nigra*, potentially toxic elements, soil-to-organ concentration ratios, edible fruits, ICP-MS, food safety, dietary exposure, urban contamination

## Abstract

Urban environments may influence elemental exposure from wild edible fruits. This study evaluated spatial patterns, soil-to-organ concentration ratios, and food-safety implications of potentially toxic elements in soil and four *Sambucus nigra* organs collected from 94 sites across five ecological sectors in Oradea, Romania (470 samples). Elements were determined by ICP-MS, and the data were evaluated using soil-to-organ concentration ratios, multivariate analyses, regulatory assessment, dietary-exposure assessment, and non-carcinogenic risk analysis. Elemental profiles varied strongly by matrix and sector, with concentrations generally highest in the central urban sector (Z2) and lowest in the woodland-associated sector (Z4). Bark exhibited the highest soil-normalized concentration ratios for potentially toxic elements; however, because bark was analyzed unwashed, these values may include surface deposition and should not be interpreted as evidence of greater internal accumulation relative to the rinsed organs. In fruits, Cr and Ni were detected in 97.9% and 94.7% of samples; Pb, Cd, As, and Sb occurred mainly in Z2, while Hg remained below the detection limit. Z2 fruits contained 5.92 ± 1.10 detected potentially toxic elements per sample, compared with 1.61 ± 0.61 in Z4. No fruit sample exceeded the applicable EU maximum levels for Pb or Cd. For the additional elements evaluated through the screening-level dietary exposure assessment, all calculated THQs and partial HI values were below 1 under the adopted exposure assumptions. The results indicate a pronounced urban multi-element signal but no appreciable non-carcinogenic concern under the adopted assumptions.

## 1. Introduction

Metal and metalloid contamination of foods represents an important intersection between environmental pollution, food-chain integrity, and consumer health [1]. In plant-derived foods, these elements may originate from the natural geochemical background or from anthropogenic sources, including traffic, combustion, industrial activities, construction, and atmospheric deposition [2,3]. Urban and periurban wild edible plants may be contaminated through soil uptake and atmospheric deposition [4,5]. Topolska et al. (2020) [6] identified element-specific relationships among soil characteristics, traffic intensity, and metal concentrations in urban *Sambucus nigra* flowers, while Bărbulescu et al. (2022) [7] demonstrated substantial variability in soil-to-plant elemental relationships across elements, plant species, and sampling environments. Nevertheless, the total concentration of an element in soil does not directly predict its concentration in edible tissues [8,9]. As emphasized by Antoniadis et al. (2017) [10], phytoavailability and transfer are also governed by soil pH, organic matter, sorption capacity, chemical speciation, competitive interactions, and plant-specific uptake, translocation, exclusion, and storage mechanisms [11,12]. Food-safety assessment must therefore trace contaminants from their environmental occurrence to their accumulation in the edible matrix [13,14,15].

*Sambucus nigra* L. is particularly relevant for investigating this environment-to-food pathway because its flowers and ripe fruits are widely used in juices, syrups, jams, fermented beverages, and other food products [16,17]. Its occurrence in both semi-natural and highly anthropogenic environments also makes it a useful indicator of spatial contamination [18,19]. Previous research has demonstrated pronounced organ- and location-dependent variability in its elemental composition [20,21,22]. Qazimi et al. (2024) [23] found elemental differences between wild elder leaves and flowers, while Vlaicu et al. (2025) [24] reported geographical and organ-specific variation in Romanian flowers, berries, and leaves. Crișan et al. (2025) [25] additionally reported ecological-region variability in elderflowers. Using complementary LC-HRMS and ICP-OES analyses, Häßler et al. (2025) [26] further demonstrated distinct chemical and elemental profiles among roots, bark, leaves, flowers, and berries. Figas et al. (2024) [27] also showed that anthropogenic pressure can influence potentially toxic element concentrations in elderflowers. However, previous studies have rarely integrated spatially paired soil and multi-organ sampling across broad environmental gradients [28,29]. Consequently, the relationship between soil elemental signatures and elemental concentrations in ripe fruits remains poorly characterized [30,31].

Previous studies have indicated that the distribution of metals and metalloids among plant organs may be influenced by biological processes such as root exclusion, tissue binding, sequestration, and selective transport [32,33,34]. These mechanisms provide a possible biological context for organ-specific elemental patterns but were not directly evaluated in the present study. Similar partitioning occurs in other perennial food species, with bark and vegetative tissues often retaining more potentially toxic elements than fruits [35,36]. Although Diviš et al. (2015) [37] documented variability in the elemental composition of black elder fruits among cultivars, environmental controls on soil–fruit elemental relationships remain poorly resolved. This limitation is particularly relevant in urban edible landscapes, where fruit contamination reflects land use, plant physiology, and pollution patterns [38,39]. Traffic-related emissions can generate complex mixtures of Fe, Cu, Zn, Cr, Ni, Pb, Sb, and other elements [40,41]. A site-paired multi-matrix design can characterize associations among soil elemental composition, organ-specific elemental profiles, and fruit contamination across environmental gradients [42,43].

The detection of a metal or metalloid in food does not by itself establish regulatory non-compliance or an unacceptable health risk [44,45]. Regulatory interpretation requires comparison with applicable maximum levels expressed on the appropriate mass basis [46,47]. For elderberries, Pb and Cd must be assessed on a wet-weight basis against the limits in Commission Regulation (EU) 2023/915 [48]. Exposure assessment must also account for results below the analytical limit of detection using lower-, middle-, and upper-bound scenarios [49], as recommended by the European Food Safety Authority [50]. Toxicological interpretation requires element-specific caution: dietary Ni exposure has been evaluated in exposure studies [51] and should be interpreted using an appropriate oral health-based guidance value [52], whereas total Cr cannot be linked to the distinct toxicological profiles of Cr(III) and Cr(VI) without chemical speciation [53]. Consistent with the WHO/FAO framework [45], THQ and HI are screening indicators rather than direct probabilities of adverse effects.

Previous studies have reported organ- and location-dependent elemental variability in *Sambucus nigra*, but the extent to which urban environmental contamination is reflected across soil, different plant organs, and edible fruits remains insufficiently resolved. We hypothesized that stronger anthropogenic pressure would be associated with higher multi-element burdens in soil and plant tissues, while organ-specific differences in elemental distribution would be associated with comparatively lower concentrations of potentially toxic elements in ripe fruits. The novelty of this study lies in the site-paired assessment of soil, bark, leaves, flowers, and fruits from 94 georeferenced sites, integrating elemental distribution, soil-to-organ concentration relationships, fruit contamination, regulatory compliance, dietary exposure, and non-carcinogenic risk.

## 2. Materials and Methods

### 2.1. Study Area and Environmental Characterization

The study covered five ecological sectors along an urban-to-semi-natural gradient in metropolitan Oradea, northwestern Romania: eastern periurban, central urban, western transitional, northern/northeastern woodland, and southern/southwestern periurban.

### 2.2. Experimental Design and Sampling Strategy

**Figure 1:**
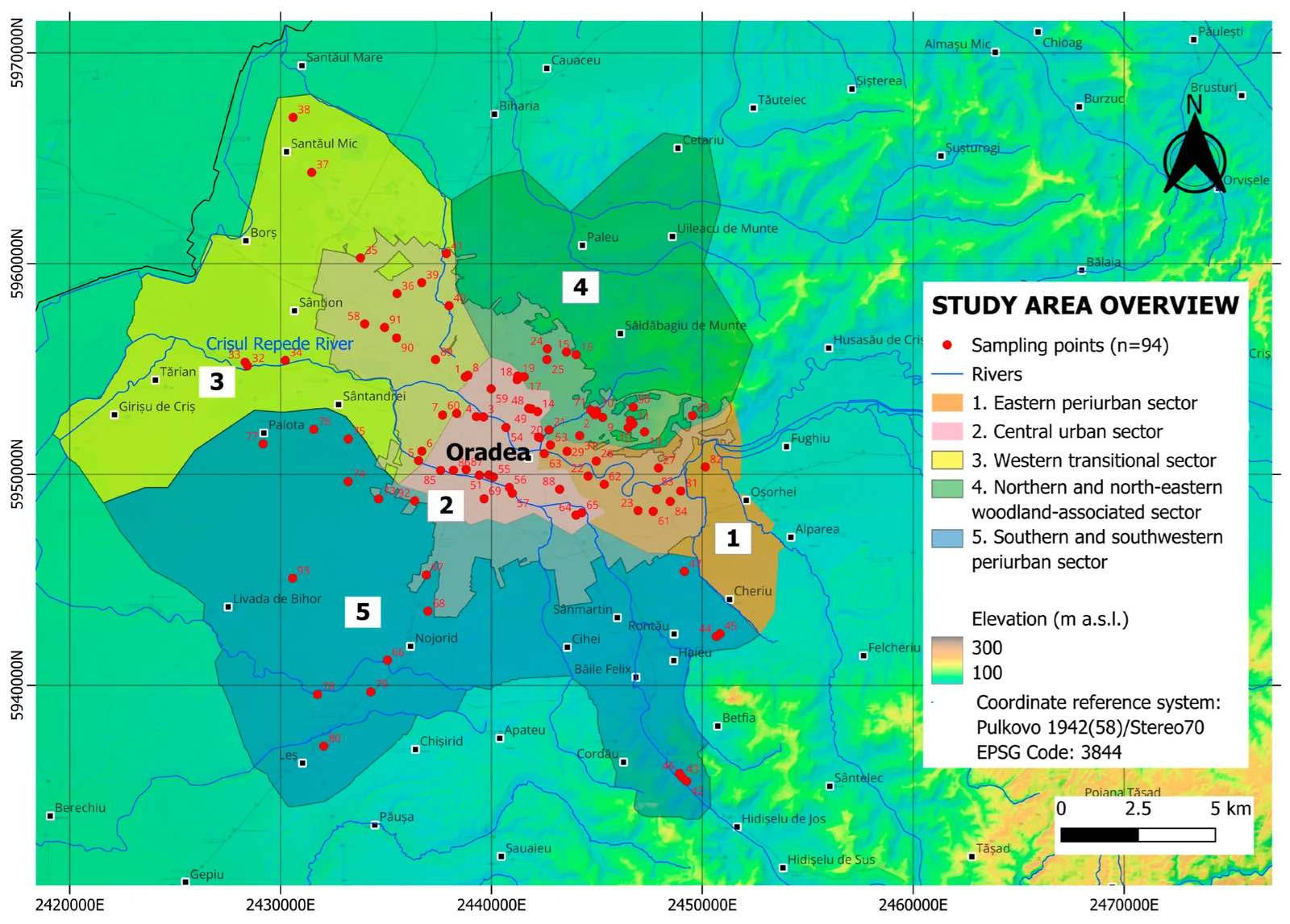
Distribution of the 94 georeferenced sampling sites across the five predefined ecological sectors in the Oradea metropolitan area, northwestern Romania.

A stratified, site-paired, multi-matrix design included 94 georeferenced sites across five sectors: eastern periurban (*n* = 13), central urban (*n* = 24), western transitional (*n* = 20), northern/northeastern woodland (*n* = 18), and southern/southwestern periurban (*n* = 19) (Figure 1).

At each of the 94 georeferenced sites, one mature, visually healthy *Sambucus nigra* L. individual was selected as the fixed plant-associated sampling unit and revisited during the three phenology-specific sampling periods. Sampling was conducted during three phenological stages in 2026: soil, bark, and leaves on 18–31 May; flowers on 1–20 June; and ripe fruits on 10 August–5 September. Sampling dates were adjusted to plant phenology, yielding 470 samples (94 per matrix; Table S1).

### 2.3. Ecological Zoning and Distribution of Sampling Sites

Each sampling location was classified by land use, habitat structure, urbanization, proximity to infrastructure, and ecological continuity into periurban riparian, intensively urbanized, urban–semi-natural transitional, woodland-associated, or periurban–rural sectors. Sector characteristics and sampling-point IDs are provided in Table S2, while sector-specific field conditions and soil-sampling exclusion criteria are summarized in Table S3. These sectors represent composite environmental categories rather than independent measures of specific contamination sources. Site-level distance from major roads and quantitative traffic-intensity data were not available; therefore, sector-related differences were interpreted as spatial associations rather than as causal effects or direct measures of traffic exposure.

### 2.4. Multi-Matrix Sampling of Sambucus nigra and Associated Soil

At each georeferenced sampling site, soil, bark, leaves, flowers, and fruits were collected from the same selected *Sambucus nigra* L. individual and its immediately associated soil environment. This site-paired, multi-matrix approach ensured direct spatial correspondence among the environmental and plant samples collected at each location. Detailed field-sampling specifications for each matrix are provided in Table S4.

#### 2.4.1. Soil Sampling

After removing litter, plant residues, stones, and other visible foreign materials, three to five subsamples were collected at a depth of 0–20 cm within approximately 1–2 m of the plant base using a pre-cleaned stainless-steel soil auger. The subsamples were combined and field-homogenized to obtain one representative composite soil sample of approximately 500–1000 g, which was transferred to a prelabeled polyethylene bag. Samples were handled using powder-free nitrile gloves. The stainless-steel soil auger was decontaminated between sampling sites using ultrapure water produced with a Milli-Q Direct 8 water purification system (Merck KGaA, Darmstadt, Germany), followed by analytical-grade ethanol, to minimize the risk of cross-contamination.

#### 2.4.2. Bark Sampling

External bark fragments were collected approximately 1.0–1.5 m above ground level from several stem orientations, where possible, using a stainless-steel scalpel, and combined into one representative composite sample.

#### 2.4.3. Leaf Sampling

Fully expanded mature leaves were collected from the median canopy layer and several canopy orientations, where possible, using stainless-steel scissors, and combined into one representative composite sample.

#### 2.4.4. Flower Sampling

Inflorescences at full flowering were collected from comparable median-to-upper canopy positions and several canopy orientations, where possible, using stainless-steel scissors, and combined into one representative composite sample.

#### 2.4.5. Fruit Sampling

Fully ripe fruit clusters were collected from comparable median-to-upper canopy positions and several canopy orientations, where possible, using stainless-steel scissors, and combined into one representative composite sample.

### 2.5. Sample Identification, Transport, and Storage

Samples were assigned matrix-specific identification codes: SN-SO-xxx (soil), SN-BA-xxx (bark), SN-LE-xxx (leaves), SN-FL-xxx (flowers), and SN-FR-xxx (fruits). Each code was linked to the sampling site, ecological sector, GPS coordinates, matrix, field visit, and collection date. Immediately after collection, samples were placed in sealed, prelabeled polyethylene bags and protected from direct sunlight and potential cross-contamination during transport. Samples were transported to the laboratory on the same day, generally within approximately 1–3 h of collection. Fruit samples were maintained under refrigerated conditions at approximately 4–8 °C during transport. After laboratory preparation, samples were stored in tightly closed, prelabeled polyethylene containers under dry conditions until analysis.

### 2.6. Sample Preparation

All samples were prepared separately by matrix and sampling site to preserve traceability and minimize cross-contamination. Matrix-specific procedures for plant samples are summarized in Table S5. Unless otherwise specified, bark, leaves, flowers, and fruits were dried to constant mass at 40 ± 2 °C in a UF55 forced-air laboratory oven (Memmert GmbH + Co. KG, Schwabach, Germany), cooled in a silica-gel desiccator, and weighed using an AS 60/220.R2 PLUS analytical balance (RADWAG Balances and Scales, Radom, Poland). The dried material was finely homogenized using an agate mortar and pestle or a Mixer Mill MM 400 with zirconium oxide jars and balls (RETSCH GmbH, Haan, Germany).

#### 2.6.1. Soil Sample Preparation

Soil samples were manually cleaned of roots, plant residues, stones, coarse mineral fragments, and foreign particles using polypropylene (PP) tweezers and ceramic laboratory tools. The unwashed samples were air-dried to constant mass at a controlled laboratory temperature of 22 ± 2 °C and a relative humidity of 50 ± 5%, in a clean and well-ventilated environment, protected from direct sunlight, dust, and potential sources of contamination. After drying, the samples were homogenized, passed through a 2 mm polyethylene sieve, and finely ground using an agate mortar and pestle or a Mixer Mill MM 400 with zirconium oxide jars and balls (RETSCH GmbH, Haan, Germany).

#### 2.6.2. Bark Sample Preparation

Bark samples were manually cleaned of adhering debris, degraded tissues, and foreign particles using plastic tweezers and ceramic laboratory tools, without washing.

#### 2.6.3. Leaf Sample Preparation

Leaf samples were sorted, gently rinsed with Milli-Q water produced using a Milli-Q Direct 8 system (Merck KGaA, Darmstadt, Germany), and blotted dry.

#### 2.6.4. Flower Sample Preparation

Flower samples were sorted, gently rinsed with Milli-Q water, blotted dry, and spread on clean polyethylene trays.

#### 2.6.5. Fruit Sample Preparation

Dry-matter content was determined individually for each of the 94 fruit samples from the recorded fresh and dry masses. Elemental concentrations measured in the dried fruit material were converted from dry weight to wet weight using the sample-specific dry-to-fresh mass ratio, according to:

$$C_{\mathrm{WW}}\;=\;C_{\mathrm{DW}}\;\times \;\frac{m_{\mathrm{dry}}}{m_{\mathrm{fresh}}}$$

where *C*_WW_ is the elemental concentration expressed on a wet-weight basis (mg/kg WW), *C*_DW_ is the corresponding concentration expressed on a dry-weight basis (mg/kg DW), and *m*_dry_ and *m*_fresh_ are the recorded dry and fresh masses of the corresponding fruit sample, respectively. The conversion was performed separately for each fruit sample.

Dry-weight concentrations were used for environmental, spatial, and soil-to-organ concentration-ratio analyses, whereas wet-weight concentrations were used for food-safety, regulatory, and dietary-exposure assessments.

### 2.7. Physicochemical Characterization of Soil Samples

Soil physicochemical characterization included pH, electrical conductivity, salinity, moisture content, organic matter, estimated soil organic carbon, calcium carbonate, total nitrogen, particle-size distribution, potential cation exchange capacity, plant-available phosphorus and potassium, DTPA-extractable Fe, Mn, Zn, and Cu, dry bulk density, and saturated hydraulic conductivity. The pH electrode and conductivity sensor were calibrated before each daily analytical series using certified standards, and conductivity calibration was independently verified using a second-source certified 500 µS cm^−1^ standard. Detailed analytical procedures, instrumentation, calculation equations, calibration-verification procedures, acceptance criteria, replication design, and quality-control measures are provided in the Supplementary Materials.

### 2.8. Microwave-Assisted Acid Digestion of Soil and Plant Samples

Soil and plant samples were subjected to closed-vessel microwave-assisted acid digestion prior to ICP-MS determination. The analytical portions, digestion reagents, microwave operating program, post-digestion treatment, final dilution volumes, acceptance criteria, and digestion-related quality-control measures are summarized in Table S6.

### 2.9. ICP-MS Determination of Macro-, Trace, and Potentially Toxic Elements

Following microwave-assisted digestion, the resulting soil and plant sample solutions were analyzed by quadrupole ICP-MS. Macro-, trace, and potentially toxic elements were quantified from the ion intensities recorded for the selected isotopes using multi-point external calibration with internal-standard correction. Concentrations obtained in the measurement solutions were recalculated to the original samples by accounting for the final digest volume, any additional dilution factor, and the dry mass of the analytical portion. Final results were expressed as mg kg^−1^ dry weight (DW), and concentrations below the method detection limit were reported as <LOD. The complete instrumental configuration, operating conditions, acquisition parameters, interference-control procedure, investigated elements, and monitored isotopes are provided in Table S7.

### 2.10. Method Validation and Quality Assurance/Quality Control

The ICP-MS method was evaluated for calibration performance, sensitivity, precision, trueness, recovery, measurement uncertainty, instrumental drift, and background contribution. Procedural blanks, independently prepared replicates, certified reference materials, and second-source certified standards were included in the analytical workflow. Complete element-specific validation and quality-control results are provided in Tables S8 and S9. For reporting purposes, an element was considered detected when its analytical response corresponded to a concentration ≥LOD, whereas quantitative concentration values were reported and used in concentration-based calculations only when they were ≥LOQ. Values between LOD and LOQ, if present, were regarded as detected but not quantitatively reportable.

### 2.11. Assessment of Soil-to-Organ Concentration Ratios

Soil-to-organ concentration ratios (TFs) were calculated for each element, plant organ, and georeferenced sampling site as:

$${\mathrm{TF}}_{i,j}=\frac{C_{i,j}}{C_{i,\mathrm{soil}}}$$

where *C_i,j_* is the element concentration in organ j (bark, leaves, flowers, or fruits) at site i, and *C_i,soil_* is the corresponding site-paired soil concentration. Concentrations were expressed as mg/kg DW, and TFs were dimensionless. TFs were calculated from site-level concentrations obtained by averaging three independently weighed and digested analytical portions. Only soil-organ pairs in which both concentrations were ≥LOQ were included (maximum *n* = 94); otherwise, TF was not determined (ND). Results were summarized as median [Q1–Q3], and differences among organs were evaluated statistically. Because the soil denominator represents the acid-recoverable rather than the bioavailable elemental concentration, TFs were interpreted as soil-normalized concentration ratios, not as direct measures of uptake efficiency. Bark TFs may also reflect surface deposition because bark samples were analyzed unwashed. Soil-organ associations were assessed using Spearman’s rank correlation coefficient (r*_s_*), with <LOD observations retained as tied censored ranks below the lowest detected concentration.

### 2.12. Dietary Exposure and Human Health Risk Assessment

#### 2.12.1. Regulatory Food-Safety Assessment

Pb and Cd concentrations in individual *Sambucus nigra* L. fruit samples were assessed against the EU maximum levels (MLs) of 0.20 and 0.030 mg/kg wet weight (WW), respectively [48], after conversion from dry weight using the sample-specific dry-matter fraction. Each sample-specific wet-weight concentration was compared with the applicable EU maximum level, and compliance was expressed as the percentage of the corresponding maximum level. Values above 100% were considered exceedances, and their number and proportion were reported. Other PTEs were evaluated through the dietary exposure and non-carcinogenic risk assessment described below.

#### 2.12.2. Estimated Daily Intake

Dietary exposure to Pb, Cd, As, Hg, Cr, Ni, Sb, and Sn from the consumption of edible *Sambucus nigra* L. fruits was estimated on a wet-weight (WW) basis. Concentrations measured on a dry-weight (DW) basis were converted to WW by multiplying each value by the experimentally determined sample-specific dry-matter fraction. Left-censored results (<LOD) were assigned 0, LOD/2, and LOD under the lower-bound (LB), middle-bound (MB), and upper-bound (UB) scenarios, respectively. Results ≥LOD were detected, whereas only those ≥LOQ were quantitatively reportable; no result between LOD and LOQ was used numerically. The MB scenario was used as the primary estimate. Central and upper-end exposure were calculated using the mean and 95th percentile (P95), respectively, of the complete MB concentration distribution across all 94 fruit samples. The P95 scenario reflected higher fruit concentrations, not a higher consumption rate.

Estimated daily intake (EDI) was calculated according to:

$${\mathrm{EDI}}_{i}=\frac{C_{i}\times \mathrm{IR}}{\mathrm{BW}}$$

where *EDI_i_* is the estimated daily intake of element *i* (mg/kg body weight/day), *C_i_* is its concentration in edible fruits (mg/kg WW), *IR* is the assumed daily fruit ingestion rate (0.100 kg/day), and *BW* is the assumed adult body weight (70 kg). An adult body weight of 70 kg was adopted in accordance with previous human-health risk assessments involving Sambucus nigra [23]. In the absence of population-specific data on habitual fresh elderberry consumption, an ingestion rate of 0.100 kg/day was adopted as a conservative hypothetical screening scenario corresponding to daily consumption of a 100 g portion of fresh elderberries. This ingestion rate was not intended to represent measured habitual consumption in the local population. Accordingly, the resulting EDI, THQ, and HI values should be interpreted as scenario-based screening estimates rather than estimates of actual population exposure, consistent with the general risk-assessment framework described by WHO/FAO [45]. Central and P95 EDI values were calculated using the mean and P95 MB concentrations, respectively, with the same ingestion rate and body weight. Mean LB and UB concentrations were additionally used to define the central EDI uncertainty interval associated with left-censored data. Because Hg was <LOD in all fruit samples, its LB, MB, and UB values were entirely censoring-based and were not supported by quantitatively reportable concentrations.

#### 2.12.3. Target Hazard Quotient

Potential non-carcinogenic risk from dietary exposure to Cd, Ni, and Sb was assessed using the target hazard quotient (THQ):

$${\mathrm{THQ}}_{i}=\frac{{\mathrm{EDI}}_{i}}{{\mathrm{RfD}}_{i}}$$

where *EDI*_i_ is the estimated daily intake and *RfD*_i_ is the oral reference dose (mg/kg bw/day). The U.S. EPA IRIS RfDs used were 1.0 × 10^−3^ for Cd in food, 2.0 × 10^−2^ for soluble nickel salts, and 4.0 × 10^−4^ for Sb [54,55,56]. THQs were calculated for the central and P95 exposure scenarios. Values below 1 indicated no appreciable non-carcinogenic concern under the adopted assumptions, whereas values ≥1 indicated potential concern requiring further evaluation.

#### 2.12.4. Hazard Index

Potential cumulative non-carcinogenic risk arising from simultaneous dietary exposure to multiple potentially toxic elements was assessed using the hazard index (HI), calculated as the sum of the element-specific THQs:

$$\mathrm{HI}=\sum_{i=1}^{n}{\mathrm{THQ}}_{i}$$

where *THQ_i_* represents the target hazard quotient of each element included in the assessment.

Separate HI values were calculated for the central and P95 exposure scenarios using the corresponding element-specific THQs. An HI < 1 was interpreted as indicating no appreciable cumulative non-carcinogenic risk under the adopted screening assumptions, whereas an HI ≥ 1 was considered to indicate that combined exposure may warrant further toxicological evaluation. Because the HI approach assumes additivity of effects across the included elements, the resulting values were interpreted as screening indicators rather than quantitative predictions of adverse health outcomes.

To assess spatial variation in dietary exposure and non-carcinogenic risk, the central exposure assessment was also conducted separately for each ecological sector (Z1, *n* = 13; Z2, *n* = 24; Z3, *n* = 20; Z4, *n* = 18; and Z5, *n* = 19). For each element and sector, the arithmetic mean of the complete middle-bound (MB) wet-weight concentration distribution was used to calculate the corresponding EDI using the same ingestion rate (0.100 kg/day) and adult body weight (70 kg) adopted for the overall assessment. Sector-specific THQ and cumulative HI values were subsequently calculated using the same toxicological reference values and element-inclusion criteria applied in the overall non-carcinogenic risk assessment. Sector-specific P95 exposure estimates were not derived because of the relatively small number of samples within individual sectors (*n* = 13–24), which would yield insufficiently stable estimates of the 95th percentile. Accordingly, the spatial assessment was based on central exposure estimates and was used to compare relative dietary exposure and screening-level non-carcinogenic risk among the five ecological sectors.

### 2.13. Statistical Analysis

Three independently weighed and digested analytical portions were averaged to obtain one concentration per matrix and site. These replicates were used only to assess analytical precision and were not treated as independent observations. All inferential analyses used the resulting site-level values, with the 94 georeferenced sites as independent observational units. Sector differences were assessed using ANOVA/Tukey, Welch/Games–Howell, or Kruskal–Wallis and Mann–Whitney U tests, as appropriate. Spearman’s correlation was used for spatial gradients. TFs were compared using Friedman’s test with Kendall’s W and Holm-adjusted post hoc comparisons based on complete cases; Hg was excluded because all fruit concentrations were <LOD. Multiplicity was controlled within related test families using the corresponding post hoc procedures or Benjamini–Hochberg correction. Adjusted *p*-values < 0.05, or *q* < 0.05 for Benjamini–Hochberg correction, were considered significant. For global PCA, the 470 samples were combined, values <LOD were replaced by LOD/2, and concentrations were log_10_-transformed and autoscaled. PCA was interpreted as an exploratory comparison of standardized multielement profiles rather than evidence of direct transfer among matrices. MFA, HCA, PERMANOVA, and PERMDISP provided complementary multivariate analyses.

## 3. Results

### 3.1. Physicochemical Characteristics of Soils Across the Ecological Sectors

The physicochemical characteristics of the investigated soils differed among the five ecological sectors (Table S10). The central urban sector (Z2) generally showed a more mineral-rich and compact soil profile, with higher pH, electrical conductivity, clay-related properties, and extractable nutrient and metal concentrations, whereas the woodland-associated sector (Z4) was characterized by higher organic matter, moisture, and hydraulic conductivity and lower values for several of the physicochemical indicators elevated in Z2. The remaining sectors generally displayed intermediate soil characteristics, indicating a clear physicochemical contrast between the central urban and woodland-associated environments.

### 3.2. Elemental Composition of Soils Along the Urban–Periurban Gradient

Soil elemental composition showed clear spatial differentiation across the five ecological sectors (Table 1), with complete sample-level concentrations provided in Tables S11–S14. Most major elements and potentially toxic elements exhibited positive associations with the predefined spatial gradient (Z4 < Z1 < Z5 < Z3 < Z2), with Z2 generally showing the highest concentrations and Z4 the lowest. In contrast, Al, Li, Rb, and Cs displayed strong negative associations with the gradient. Overall, the elemental dataset showed two contrasting spatial signatures, characterized by higher concentrations of most major elements and PTEs toward the central urban sector and higher concentrations of selected lithogenic elements toward the woodland-associated sector.

To contextualize the absolute soil PTE concentrations, individual site-level results were screened against the normal/reference, alert, and intervention values established by Romanian Order No. 756/1997 (Table S37). The maximum concentrations of Cd, As, Hg, Cr, Ni, Sb, and Sn were 1.17, 9.45, 0.182, 87.7, 66.9, 5.94, and 11.3 mg/kg dry matter, respectively, and remained below the corresponding alert thresholds for both sensitive and less-sensitive land uses. Pb reached a maximum of 62.0 mg/kg dry matter, and seven Z2 sites exceeded the sensitive-use alert threshold of 50 mg/kg; however, all Pb concentrations remained below the sensitive-use intervention threshold of 100 mg/kg and the less-sensitive-use alert threshold of 250 mg/kg. Normal/reference values were exceeded for Pb (57/94 sites), Cd (5/94), As (23/94), Hg (12/94), Cr (85/94), Ni (83/94), and Sb (4/94), but not for Sn. Thus, several elements showed enrichment above normal/reference levels, whereas the absolute contamination severity was generally moderate, with localized alert-level Pb elevations in Z2 and no intervention-threshold exceedances. Because the soil digestion did not include hydrofluoric acid, these comparisons represent contextual screening of acid-recoverable or pseudo-total concentrations rather than a formal regulatory classification.

### 3.3. Organ-Specific Elemental Profiles of Sambucus nigra

Elemental profiles differed among plant organs and across the predefined spatial gradient (Table 2, Table 3, Table 4 and Table 5), revealing organ-specific spatial patterns. Complete georeferenced sample-level concentrations are provided in Tables S15–S30.

#### 3.3.1. Elemental Composition of Bark

Bark exhibited one of the clearest positive spatial patterns among the investigated organs (Table 2), with most major, trace, lithogenic, and potentially toxic elements showing higher concentrations or detection frequencies toward Z2 and generally lower values in Z4. Several lithogenic elements displayed a positive bark gradient despite their contrasting distribution in soil. Overall, bark showed pronounced sector-dependent elemental differentiation, including for Cr, Ni, and the more extensively censored PTEs. Complete individual-sample concentrations are provided in Tables S15–S18.

#### 3.3.2. Elemental Composition of Leaves

Leaves showed a contrasting elemental pattern (Table 3). K, Ca, Mg, and P were inversely associated with the spatial gradient, generally reaching higher concentrations in Z4, whereas most trace, lithogenic, and potentially toxic elements increased toward Z2. Na and S also followed positive spatial patterns, while Mn showed little monotonic variation. Thus, leaves were characterized by a clear divergence between several major nutrients and most trace and potentially toxic elements. Complete individual-sample data are provided in Tables S19–S22.

#### 3.3.3. Elemental Composition of Flowers

Flowers showed clear sector-dependent elemental differentiation, although major-element patterns were less uniform than in bark (Table 4). K varied comparatively little across sectors, whereas several other major elements differed significantly among sectors. Most trace, lithogenic, and potentially toxic elements were generally higher toward Z2 or Z3 and lower in Z4, with particularly clear spatial differentiation for Pb, Cd, As, Cr, Ni, Sb, and Sn. Complete individual-sample data are provided in Tables S23–S26.

#### 3.3.4. Elemental Composition of Fruits

Ripe fruits showed pronounced spatial differentiation, with particular relevance because they represent the edible matrix (Table 5). Most elements showed higher concentrations toward Z2 and lower concentrations toward Z4, but the most important pattern concerned the occurrence of PTEs. Pb, Cd, and As were detected predominantly in Z2, whereas Hg remained below the LOD in all 94 fruit samples; Cr and Ni were detected in most samples and also showed strong positive spatial gradients. Detailed concentration and detection data are provided in Tables S27–S30.

### 3.4. Soil-to-Organ Concentration Ratios and Organ-Specific Element Patterns

As shown in Table 6, macroelements and biologically relevant trace elements exhibited clear organ-specific patterns. Leaves had the highest soil-to-organ concentration ratios for K, Ca, Mg, and S, whereas P was highest in flowers. Among trace elements, Mn, Mo, and Co showed the highest ratios in leaves, while Zn, Cu, B, and Se were highest in flowers. These TF patterns complement the organ-specific concentration profiles presented in Table 2, Table 3, Table 4, Table 5 and Tables S15–S30, but did not always parallel the corresponding soil–organ correlations, indicating that higher soil-normalized ratios did not necessarily imply stronger spatial covariation with soil concentrations.

Lithogenic and potentially toxic elements showed a more consistent organ hierarchy (Table 6), with bark generally exhibiting the highest measured ratios and fruits the lowest. Because bark was analyzed unwashed, its comparatively high ratios may partly reflect surface-deposited particulate material in addition to internal elemental content and should therefore not be interpreted as evidence of greater uptake or translocation. Fruits remained at the lower end of the TF hierarchy, particularly for potentially toxic elements, while no fruit TF could be calculated for Hg because valid quantifiable soil–fruit pairs were unavailable.

### 3.5. Occurrence and Spatial Variation in Potentially Toxic Elements in Edible Fruits

The occurrence of potentially toxic elements in edible *Sambucus nigra* fruits differed markedly among elements and ecological sectors (Table 7, Table 8 and Table 9). Cr and Ni showed the highest detection frequencies, whereas Hg remained below the LOD in all 94 fruit samples. Pb, Cd, As, Sb, and Sn occurred less frequently, with their detections showing pronounced spatial differentiation among sectors (Table 7 and Table S31).

A clear concentration of detections was observed in the central urban sector (Z2), particularly for Pb, Cd, As, Sb, and Sn (Table 8). In contrast, the near-universal detection of Cr and Ni limited the discriminatory value of presence/absence comparisons, although their concentrations still differed markedly among sectors (Table 7, Tables S32 and S33).

The multi-element detection burden also differed strongly among ecological sectors, reaching its highest level in Z2 and its lowest in Z4 (Table 9). Several significant PTE co-detection patterns were observed after FDR adjustment, with the strongest association between Cd and Sb (Table S34). Overall, edible fruits showed a clearly spatially structured PTE occurrence profile, characterized by greater multi-element burden in Z2 and substantially lower burden in Z4.

### 3.6. Dietary Exposure and Human Health Risk Associated with Potentially Toxic Elements in Edible Fruits

#### 3.6.1. Regulatory Compliance of Edible Fruits with Food-Safety Limits

Following conversion from dry weight to wet weight, the maximum Pb concentration was 0.0214 mg/kg WW in SN-FR-065 (Z2), representing 10.70% of the EU maximum level of 0.20 mg/kg WW. For Cd, the maximum was 0.00216 mg/kg WW in SN-FR-087 (Z2), corresponding to 7.20% of the applicable maximum level of 0.030 mg/kg WW. Pb and Cd were detected in 35 and 18 of the 94 fruit samples, respectively. No sample exceeded either maximum level, both of which apply to the washed edible portion (Table 10).

#### 3.6.2. Estimated Dietary Exposure to Potentially Toxic Elements

Ni and Cr showed the highest estimated dietary intakes under both exposure scenarios. P95 exposure estimates were higher than the corresponding central estimates for all investigated elements (Table 11).

#### 3.6.3. Non-Carcinogenic Risk Characterization

All individual THQs and the partial cumulative HI remained below 1 under both the central and P95 exposure scenarios, with Ni showing the highest individual THQ (Table 12).

#### 3.6.4. Spatial Variation in Consumer Exposure and Risk Across Z1–Z5

Sector-specific partial HI was highest in Z2 and lowest in Z4, with Ni representing the dominant contributor in all ecological sectors; all sector-specific HI values remained below 1 (Table 13 and Table S38).

### 3.7. Multivariate Differentiation of Ecological Sectors and Sample Matrices

#### 3.7.1. Global Multivariate Differentiation Among Sample Matrices

The global PCA showed clear differentiation among soil, bark, leaves, flowers, and fruits. PC1 explained 70.96% of the total variance and PC2 accounted for an additional 15.17%, resulting in a cumulative explained variance of 86.13% (Table S39). Soil was clearly separated from the plant matrices, while leaves and flowers showed greater similarity and bark and fruits occupied distinct positions in the multivariate space (Table S40). PC1 was predominantly associated with mineral and trace elements, whereas PC2 mainly reflected variation in major nutrient elements; complete loading coefficients are provided in Table S41.

#### 3.7.2. Integrated Multi-Matrix Differentiation of Ecological Sectors

Multiple factor analysis integrated the site-paired elemental profiles of soil, bark, leaves, flowers, and fruits while balancing the contribution of each matrix block. Dim1 explained 49.20% of the total variance and Dim2 an additional 4.58%, yielding a cumulative explained variance of 53.78% (Table S43). The first dimension represented the dominant integrated multi-matrix gradient, whereas the second captured a weaker complementary pattern. Detailed matrix normalization weights and variable–dimension associations are provided in Tables S42 and S44.

#### 3.7.3. Hierarchical Clustering and Integrated Elemental Signatures

**Figure 2:**
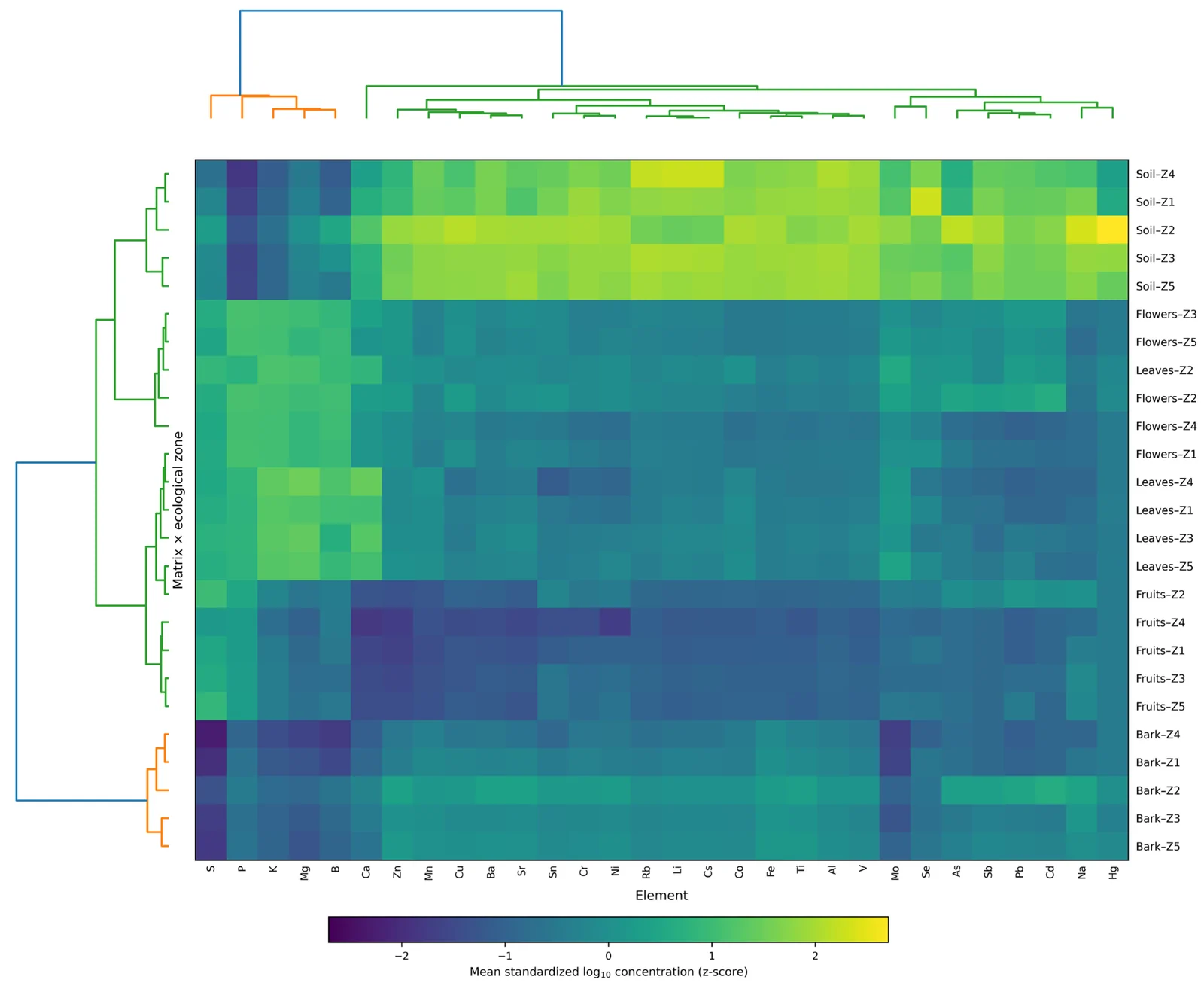
Hierarchical clustering heatmap of standardized elemental profiles across matrix × ecological-sector combinations (Z1–Z5). The 25 profiles represent five matrices (soil, bark, leaves, flowers, and fruits) across sectors Z1–Z5. Observations <LOD were replaced by LOD/2, after which concentrations were log_10_-transformed and autoscaled across the pooled dataset of 470 samples. Cells represent mean standardized values for each matrix × sector combination. Profiles and elements were clustered independently using Euclidean distance and Ward’s linkage.

Hierarchical clustering of the 25 matrix × ecological-sector profiles showed a strong matrix-dependent organization of elemental signatures (Figure 2). Soil formed a distinct cluster, bark and fruits showed matrix-specific clustering, and leaves and flowers were more closely related. Ecological-sector effects were significant within all matrices (PERMANOVA, *p* < 0.001; Table S45), with explained variation ranging from *R*^2^ = 0.486 to 0.607. PERMDISP indicated that sector differentiation in leaves and fruits primarily reflected differences in multivariate centroids, whereas dispersion heterogeneity also contributed to the patterns observed for soil, bark, and flowers. Overall, the multivariate analyses demonstrated that elemental composition was structured by both sample matrix and ecological sector.

## 4. Discussion

### 4.1. Robust and Recurrent Patterns Across the Soil–Plant System

The results revealed a consistent spatial organization of the elemental profile within the soil–Sambucus nigra system, characterized principally by higher elemental burdens in the central urban sector Z2 and lower levels in the woodland-associated sector Z4. Importantly, this sector-related differentiation was detected across all investigated matrices, although its magnitude and direction remained strongly matrix- and organ-dependent.

The lack of uniform correspondence between soil and plant concentrations indicates that plant elemental profiles cannot be interpreted as a direct reflection of total soil concentrations. Such cross-matrix decoupling is consistent with the soil–plant interface framework described by Antoniadis et al. (2017) [10], although uptake, exclusion, translocation, and storage mechanisms were not experimentally evaluated in the present study. Comparable matrix- and organ-dependent elemental variability in *S. nigra* has been reported previously [6,7,23,24], but the present site-paired design demonstrates that spatial differentiation persists across the soil–plant system without uniform propagation of soil concentrations into plant tissues.

Ripe fruits generally occupied the lower end of the soil-normalized concentration distribution for many potentially toxic elements while retaining a detectable sector-related spatial signal. Overall, the convergence of the univariate and multivariate analyses supports a system characterized by recurrent spatial differentiation, pronounced matrix dependence, and organ-specific elemental profiles rather than uniform propagation of soil elemental concentrations into plant tissues. The multivariate analyses further confirmed organization according to both matrix and ecological sector (Tables S46 and S47).

### 4.2. Environmental Signal Versus Biological Mechanisms

Soil-to-organ concentration ratios and soil–organ correlations provide complementary but distinct information on elemental distribution. TF describes the concentration of an element in a plant organ relative to the corresponding soil concentration, whereas Spearman’s coefficient reflects spatial covariation between the two matrices. Consequently, a low TF may coexist with a strong soil–organ correlation, as observed for Sn in fruits, while a comparatively high TF does not necessarily imply strong spatial coupling, as illustrated by Hg in bark (Table 6). These metrics should therefore be interpreted jointly rather than as interchangeable indicators of soil–plant relationships.

Marked organ-specific differences were evident for potentially toxic elements. Bark generally showed the highest soil-normalized concentrations, whereas fruits consistently occupied the lower end of the TF distribution for the evaluable PTEs (Table 6). A similar tendency was observed for several lithogenic/geochemical elements (Table S48). Comparable organ- and environment-dependent elemental distributions in *S. nigra* have been reported previously [26,27], supporting the interpretation that the elemental signature present in soil is not uniformly reproduced across plant organs.

The comparatively low soil-normalized concentrations of many PTEs in fruits could be compatible with several biological processes described in the literature, including root exclusion, tissue retention, cell-wall immobilization, ligand complexation, vacuolar sequestration, and selective transport between plant compartments [33,34]. However, none of these mechanisms was experimentally evaluated in the present study. The observed differences should therefore be interpreted as organ-specific concentration patterns and soil–organ associations rather than as direct evidence of uptake, exclusion, translocation, sequestration, or other specific physiological processes.

Interpretation of the bark results requires additional caution. Because bark samples were analyzed without washing, their comparatively high TFs may reflect both internal elemental content and externally deposited particulate material. Thus, the higher soil-normalized concentrations observed in bark cannot be attributed solely to biological accumulation or transport. Comparable organ-specific trace-element partitioning has also been reported in other woody species [36]. Overall, the present data demonstrate pronounced organ-specific elemental differentiation, while the physiological mechanisms potentially underlying these patterns remain unconfirmed.

### 4.3. Analytical and Methodological Constraints on Interpretation

Several analytical and methodological features define the strength of the conclusions that can be drawn from the observed fruit elemental patterns. For Pb, Cd, As, and Sb, relatively low detection frequencies and substantial left-censoring limit the reliability of fine-scale comparisons based solely on concentration magnitude (Table 7, Table 8 and Tables S31–S33). For these elements, spatial occurrence and detection frequency therefore provide more robust evidence of sector differentiation. However, the observed spatial associations indicate environmental differentiation rather than attribution to a specific contamination source.

Cr and Ni were much more consistently detected and showed clear sector-related concentration patterns, whereas Sn displayed an intermediate occurrence pattern (Table 7 and Tables S31–S33). In contrast, Hg remained below the LOD in all 94 fruit samples. Consequently, the actual Hg concentration in fruits cannot be directly quantified, and its dietary exposure can only be interpreted within the LB, MB, and UB substitution scenarios used in the exposure assessment. Cr requires an additional toxicological qualification: because only total Cr was determined and no chemical speciation was performed, the contributions of Cr(III) and Cr(VI) remain unknown. Accordingly, Cr was not included in the THQ calculation.

The multi-element burden also showed clear sector-related differentiation (Table 9), while recurrent co-detection patterns were observed among several PTEs (Table S34). These associations support the presence of a spatially structured multi-element signal but do not demonstrate a common contamination source. Potential urban inputs may include traffic-related emissions, brake and tire wear, road abrasion, resuspended dust, combustion, industrial or construction activities, and atmospheric deposition [40,41]. However, site-level distance from major roads and quantitative traffic-intensity data were not available; therefore, the present design cannot separate traffic effects from other correlated urban pressures. Traffic-related inputs are consequently discussed only as plausible contributors and not as demonstrated contamination sources. Future studies should combine site-paired multi-matrix sampling with GIS-derived road-distance metrics and direct or official traffic-intensity data. Because the fruits were rinsed before analysis, loose surface particles were reduced, although environmental deposition may still contribute indirectly through the surrounding soil–plant system.

Interpretation of soil-to-organ relationships is additionally affected by sample preparation. Bark was analyzed without washing, whereas leaves, flowers, and fruits were rinsed before analysis. Therefore, bark concentrations and the corresponding TF values may reflect both internally associated elements and externally deposited particulate material. The comparatively high bark TFs should consequently not be interpreted as direct evidence of greater root uptake, translocation, or internal accumulation.

The multivariate results also require qualification. Although PERMANOVA identified significant sector-related differentiation, PERMDISP indicated differences in within-group dispersion for soil, bark, and flowers. For these matrices, the PERMANOVA results may therefore reflect a combination of centroid separation and differences in multivariate dispersion rather than centroid separation alone.

Finally, the study covered a single growing season and therefore characterizes spatial and matrix-dependent variation during 2026 rather than interannual variability. The dietary exposure assessment was also scenario-based: the assumed consumption rate of 0.100 kg/day was predefined for risk characterization and was not derived from measured elderberry consumption in the investigated population. Accordingly, the exposure and partial HI estimates should be interpreted as standardized screening scenarios rather than direct estimates of habitual population exposure. Together, these limitations define the appropriate level of interpretation: the dataset supports robust sector- and organ-specific elemental differentiation, whereas mechanistic attribution, long-term temporal generalization, and precise population-level exposure estimation remain beyond the scope of the present design.

### 4.4. What the Study Adds Beyond an Urban–Rural Concentration Gradient

Comparison with the Romanian soil-quality criteria places the observed spatial gradient within a generally moderate contamination context: none of the investigated PTEs exceeded intervention thresholds, although seven Z2 soils exceeded the Pb alert threshold for sensitive land use (Table S37). Thus, the Z2 pattern represents relative urban enrichment with localized Pb elevations rather than evidence that the study area as a whole was highly contaminated. The principal contribution of the study extends beyond demonstrating higher concentrations in the more urbanized sector. The site-paired multi-matrix design showed that the environmental elemental signal was expressed differently across soil, bark, leaves, flowers, and fruits. Organ-specific profiles and soil-to-organ concentration relationships indicated that higher soil concentrations were not uniformly reflected in plant tissues and that soil concentrations alone did not consistently predict elemental occurrence in edible fruits (Table 2, Table 3, Table 4, Table 5 and Table 6). Bark generally showed the highest soil-to-organ concentration ratios for PTEs, although its unwashed status requires caution because surface deposition may have contributed to the measured concentrations, whereas fruits consistently occupied the lower end of these ratios (Table 6).

This distinction was particularly relevant for the edible-fruit dataset. Although Cr and Ni were frequently detected and Pb, Cd, As, and Sb occurred predominantly in the more urbanized Z2 sector, these occurrence patterns did not translate directly into regulatory non-compliance or elevated screening-level risk (Table 7 and Table 9). Pb and Cd remained below their respective EU maximum levels in every fruit sample (Table 10 and Tables S35–S37), in accordance with Commission Regulation (EU) 2023/915 [48]. Likewise, THQs for Cd, Ni, and Sb remained below 1 under both central and P95 exposure scenarios, and all sector-specific partial HI values were also below 1 (Table 11, Table 12, Table 13 and Table S38). These estimates should nevertheless be interpreted within the adopted exposure assumptions and as a partial assessment, particularly because Cr speciation was not determined and several PTE datasets were affected by left-censoring [50,53].

Taken together, the combination of site-paired soil–organ relationships, organ-specific elemental profiles, fruit occurrence patterns, and food-safety assessment demonstrates a matrix-dependent environment-to-food continuum rather than a uniform transfer of environmental contamination into edible tissues. Importantly, the pronounced urban environmental signal, particularly in Z2, coexisted with full regulatory compliance for Pb and Cd and low screening-level non-carcinogenic risk estimates under the adopted assumptions. The study therefore distinguishes three related but non-equivalent levels of evidence: environmental contamination, occurrence of contaminants in edible tissues, and regulatory or toxicological significance. Integrating these levels within the same spatial and analytical framework provides information that cannot be obtained from soil concentrations or urban–periurban comparisons alone.

### 4.5. Integrated Multi-Matrix Elemental Signatures as Evidence of Environment-to-Food Connectivity

The multivariate analyses supported a matrix-dependent organization of elemental profiles across the soil–*Sambucus nigra* system, distinguishing soil from plant compartments and revealing additional differentiation among plant organs (Tables S39–S45 and Figure 2). Comparable organ- and matrix-dependent elemental relationships have been reported in other soil–plant systems [57,58], while developmental and physiological differences among plant organs may contribute to such patterns [59,60]. However, these mechanisms were not directly evaluated in the present study.

Ecological sector significantly affected the multivariate elemental composition of all investigated matrices, supporting the persistence of spatial environmental information across the soil–plant continuum. Similar preservation of geographical elemental signatures across connected environmental and food matrices has been reported elsewhere [61]. Nevertheless, PERMDISP indicated that, for soil, bark, and flowers, dispersion differences also contributed to the observed PERMANOVA patterns; therefore, these effects should not be interpreted exclusively as centroid separation. Overall, the multivariate results support an association between environmental and edible-fruit elemental profiles within a matrix-dependent system, without demonstrating direct causal transfer pathways for individual elements or identifying a single contamination source.

### 4.6. Implications for Food-Safety Monitoring and Study Limitations

The results support the potential use of *Sambucus nigra* as a complementary biomonitoring species in urban–periurban landscapes, consistent with previous observations [6,27]. However, edible-fruit concentrations should be interpreted together with environmental and vegetative matrices because total soil composition alone does not consistently predict elemental occurrence in fruits. Multi-matrix monitoring may therefore provide a more informative framework for evaluating environmental contamination and its potential relevance to wild edible plants and food-safety assessment [39,58].

Interpretation of these findings should nevertheless consider the single-season design, left-censoring of some PTE datasets, lack of elemental speciation, the unwashed bark matrix, the absence of direct experimental evaluation of physiological mechanisms, and the scenario-based nature of the dietary exposure assessment. An additional limitation is that soils were collected in May, whereas flowers and fruits were sampled later. The May soil data were retained as a common site-specific reference for the same georeferenced plants because total soil elemental concentrations were expected to remain relatively stable within one growing season. However, as seasonal changes in element availability and atmospheric deposition were not assessed, the corresponding TFs and correlations represent spatial associations rather than contemporaneous transfer.

## 5. Conclusions

Across 94 georeferenced sites, the elemental composition of soil and *Sambucus nigra* organs was strongly structured by ecological sector and sample matrix. The central urban sector (Z2) generally showed the highest elemental concentrations and multi-element burden, whereas the woodland-associated sector (Z4) showed the lowest. Soil-to-organ concentration ratios were element- and organ-dependent: unwashed bark showed the highest measured ratios for potentially toxic elements, whereas fruits showed the lowest ratios. In fruits, Cr and Ni were the most frequently detected elements; Pb, Cd, As, and Sb were concentrated mainly in Z2; and Hg remained below the LOD in all samples. Nevertheless, no fruit sample exceeded the applicable EU maximum levels for Pb or Cd. For the other investigated potentially toxic elements, dietary exposure was evaluated at a screening level rather than through regulatory compliance criteria. Under the adopted exposure assumptions, all calculated THQs and partial HI values remained well below 1, indicating no appreciable non-carcinogenic concern within the evaluated screening scenario.

## Figures and Tables

**Table 1:** Spatial variation in soil elemental composition.

Element (mg/kg)	Z1	Z2	Z3	Z4	Z5	Spatial/Statistical Grouping	Spearman’s *r*	*p*-Value
K	1985.2 ± 272.4 ^b^	2651.5 ± 248.2 ^a^	2128.1 ± 389.4 ^b^	1635.1 ± 153.1 ^c^	2091.8 ± 310.8 ^b^	Z2 ^a^; Z1/Z3/Z5 ^b^; Z4 ^c^	0.74	<0.001
Ca	8925.2 ± 1125.9 ^b^	12,532.6 ± 1134.6 ^a^	9562.9 ± 1806.5 ^b^	7233.0 ± 677.5 ^c^	9063.7 ± 921.2 ^b^	Z2 ^a^; Z1/Z3/Z5 ^b^; Z4 ^c^	0.79	<0.001
Mg	1353.5 ± 208.5 ^b^	1838.6 ± 132.4 ^a^	1467.6 ± 292.3 ^b^	1146.6 ± 66.5 ^c^	1365.6 ± 149.4 ^b^	Z2 ^a^; Z1/Z3/Z5 ^b^; Z4 ^c^	0.76	<0.001
Na	343.5 ± 69.5 ^b^	633.1 ± 94.8 ^a^	438.2 ± 178.5 ^b^	229.9 ± 30.0 ^c^	388.8 ± 119.1 ^b^	Z2 ^a^; Z1/Z3/Z5 ^b^; Z4 ^c^	0.80	<0.001
P	44.2 ± 8.5 ^b^	72.7 ± 9.9 ^a^	50.4 ± 17.1 ^b^	30.3 ± 4.0 ^c^	53.4 ± 19.5 ^b^	Z2 ^a^; Z1/Z3/Z5 ^b^; Z4 ^c^	0.71	<0.001
S	981.7 ± 161.1 ^b^	1496.6 ± 162.4 ^a^	1093.5 ± 353.8 ^b^	692.6 ± 74.8 ^c^	1055.4 ± 283.0 ^b^	Z2 ^a^; Z1/Z3/Z5 ^b^; Z4 ^c^	0.75	<0.001
Fe	33,225.5 ± 4418.1 ^b^	42,855.8 ± 5345.4 ^a^	38,775.4 ± 7002.5 ^ab^	25,547.2 ± 6082.3 ^c^	35,208.1 ± 8097.9 ^b^	Z2 ^a^; Z3 ^ab^; Z5/Z1 ^b^; Z4 ^c^	0.66	<0.001
Mn	548.3 ± 124.1 ^c^	1022.5 ± 144.3 ^a^	846.7 ± 241.5 ^ab^	582.0 ± 138.6 ^c^	821.9 ± 193.7 ^b^	Z2 ^a^; Z3 ^ab^; Z5 ^b^; Z4/Z1 ^c^	0.68	<0.001
Zn	71.1 ± 23.1 ^b^	132.2 ± 20.1 ^a^	109.0 ± 34.8 ^a^	65.5 ± 18.3 ^b^	112.9 ± 33.2 ^a^	Z2/Z5/Z3 ^a^; Z1/Z4 ^b^	0.66	<0.001
Cu	31.6 ± 8.9 ^c^	53.1 ± 8.7 ^a^	43.4 ± 10.7 ^b^	27.9 ± 8.5 ^c^	43.9 ± 14.5 ^ab^	Z2 ^a^; Z5 ^ab^; Z3 ^b^; Z1/Z4 ^c^	0.66	<0.001
B	15.7 ± 5.3 ^c^	26.7 ± 4.8 ^a^	22.6 ± 6.2 ^ab^	14.3 ± 3.8 ^c^	18.7 ± 6.5 ^bc^	Z2 ^a^; Z3 ^ab^; Z5 ^bc^; Z1/Z4 ^c^	0.67	<0.001
Mo	1.72 ± 0.74 ^bc^	2.75 ± 0.57 ^a^	2.10 ± 0.90 ^ac^	1.44 ± 0.54 ^c^	2.17 ± 0.77 ^ab^	Z2 ^a^; Z5 ^ab^; Z3 ^ac^; Z1 ^bc^; Z4 ^c^	0.52	<0.001
Co	11.2 ± 4.3 ^bc^	18.6 ± 2.3 ^a^	14.1 ± 3.0 ^b^	9.8 ± 3.2 ^c^	14.8 ± 4.6 ^b^	Z2 ^a^; Z5/Z3 ^b^; Z1 ^bc^; Z4 ^c^	0.65	<0.001
Se	0.56 ± 0.13 ^a^	0.35 ± 0.19 ^b^	0.33 ± 0.20 ^b^	0.38 ± 0.20 ^b^	0.38 ± 0.19 ^b^	Z1 ^a^; Z4/Z5/Z2/Z3 ^b^	−0.19	0.071
Al	26,787.7 ± 2734.9 ^c^	18,167.1 ± 1748.8 ^d^	32,685.2 ± 3152.7 ^b^	37,320.7 ± 3009.1 ^a^	29,568.4 ± 3227.3 ^c^	Z4 ^a^; Z3 ^b^; Z5/Z1 ^c^; Z2 ^d^	−0.68	<0.001
Ti	260.0 ± 25.8 ^b^	182.7 ± 23.1 ^d^	297.6 ± 36.0 ^a^	219.3 ± 34.5 ^c^	293.5 ± 44.9 ^ab^	Z3 ^a^; Z5 ^ab^; Z1 ^b^; Z4 ^c^; Z2 ^d^	−0.24	0.022
V	66.0 ± 5.4 ^c^	84.6 ± 4.4 ^a^	69.8 ± 5.9 ^c^	76.5 ± 6.8 ^b^	68.2 ± 6.5 ^c^	Z2 ^a^; Z4 ^b^; Z3/Z5/Z1 ^c^	0.40	<0.001
Ba	191.8 ± 28.0 ^c^	299.8 ± 28.9 ^a^	243.7 ± 36.8 ^b^	188.5 ± 28.3 ^c^	245.9 ± 39.7 ^b^	Z2 ^a^; Z5/Z3 ^b^; Z1/Z4 ^c^	0.75	<0.001
Sr	55.8 ± 12.5 ^b^	115.6 ± 22.2 ^a^	104.4 ± 26.9 ^a^	64.7 ± 15.4 ^b^	113.0 ± 27.2 ^a^	Z2/Z5/Z3 ^a^; Z4/Z1 ^b^	0.64	<0.001
Li	30.5 ± 6.6 ^c^	14.4 ± 2.8 ^d^	42.4 ± 11.0 ^b^	64.6 ± 13.0 ^a^	32.0 ± 10.0 ^c^	Z4 ^a^; Z3 ^b^; Z5/Z1 ^c^; Z2 ^d^	−0.72	<0.001
Rb	91.1 ± 13.9 ^d^	58.1 ± 5.8 ^e^	133.4 ± 15.0 ^b^	178.0 ± 17.9 ^a^	115.8 ± 13.1 ^c^	Z4 ^a^; Z3 ^b^; Z5 ^c^; Z1 ^d^; Z2 ^e^	−0.70	<0.001
Cs	4.87 ± 0.98 ^c^	2.55 ± 0.57 ^d^	6.97 ± 1.39 ^b^	12.28 ± 1.50 ^a^	6.04 ± 1.63 ^bc^	Z4 ^a^; Z3 ^b^; Z5 ^bc^; Z1 ^c^; Z2 ^d^	−0.74	<0.001
Pb	17.4 ± 4.0 ^c^	41.3 ± 12.5 ^a^	24.3 ± 4.8 ^b^	13.4 ± 3.5 ^c^	25.4 ± 7.7 ^b^	Z2 ^a^; Z5/Z3 ^b^; Z1/Z4 ^c^	0.80	<0.001
Cd	0.27 ± 0.08 ^c^	0.73 ± 0.27 ^a^	0.45 ± 0.17 ^b^	0.14 ± 0.06 ^d^	0.36 ± 0.14 ^bc^	Z2 ^a^; Z3 ^b^; Z5 ^bc^; Z1 ^c^; Z4 ^d^	0.80	<0.001
As	2.09 ± 0.61 (8/13)	6.70 ± 1.44 (24/24)	3.22 ± 1.17 (15/20)	1.62 ± 0.45 (10/18)	3.06 ± 1.02 (15/19)	Z2 > Z3 > Z5 > Z1 > Z4	0.75 †	<0.001 †
Hg	0.025 ± 0.004 (5/13)	0.102 ± 0.049 (24/24)	0.040 ± 0.020 (17/20)	0.014 ± 0.002 (6/18)	0.030 ± 0.013 (14/19)	Z2 > Z3 > Z5 > Z1 > Z4	0.79 †	<0.001†
Cr	52.2 ± 12.3 ^ab^	62.9 ± 15.5 ^a^	47.8 ± 12.1 ^b^	30.9 ± 8.9 ^c^	47.7 ± 14.1 ^b^	Z2 ^a^; Z1 ^ab^; Z3/Z5 ^b^; Z4 ^c^	0.54	<0.001
Ni	30.2 ± 6.7 ^c^	49.5 ± 12.1 ^a^	42.8 ± 10.8 ^ab^	22.0 ± 7.8 ^d^	35.5 ± 9.4 ^bc^	Z2 ^a^; Z3 ^ab^; Z5 ^bc^; Z1 ^c^; Z4 ^d^	0.71	<0.001
Sb	0.78 ± 0.23 ^c^	3.50 ± 1.41 ^a^	1.62 ± 0.72 ^b^	0.45 ± 0.16 ^d^	1.05 ± 0.49 ^bc^	Z2 ^a^; Z3 ^b^; Z5 ^bc^; Z1 ^c^; Z4 ^d^	0.85	<0.001
Sn	2.68 ± 0.87 ^c^	8.43 ± 1.81 ^a^	4.54 ± 1.94 ^b^	1.79 ± 0.74 ^d^	3.41 ± 1.25 ^bc^	Z2 ^a^; Z3 ^b^; Z5 ^bc^; Z1 ^c^; Z4 ^d^	0.82	<0.001

Note: Values are expressed as mean ± SD (mg/kg DW). Different lowercase superscript letters within a row indicate significant sector differences (*p* < 0.05); values sharing a letter do not differ significantly. Spearman’s *r* represents the association with the spatial gradient Z4 < Z1 < Z5 < Z3 < Z2. For As and Hg, values in parentheses show the number of samples ≥LOD relative to the total analyzed (e.g., 5/13). Their means ± SD were calculated using quantitatively reportable concentrations (≥LOQ) only. The symbol “>” indicates descending sector means. † Spearman correlations and associated *p*-values for As and Hg included censored (<LOD) observations.

**Table 2:** Spatial variation in the elemental composition of bark.

Element (mg/kg)	Z1	Z2	Z3	Z4	Z5	Spatial/Statistical Grouping	Spearman’s *r*	*p*-Value
K	1495.3 ± 138.3 ^c^	2324.9 ± 314.2 ^a^	1963.3 ± 306.2 ^b^	1221.4 ± 229.5 ^c^	2021.2 ± 350.1 ^b^	Z2 ^a^; Z5/Z3 ^b^; Z1/Z4 ^c^	0.78	<0.001
Ca	3361.9 ± 413.7 ^cd^	4826.2 ± 640.3 ^a^	4263.8 ± 811.0 ^b^	2926.8 ± 453.9 ^d^	3879.4 ± 602.3 ^bc^	Z2 ^a^; Z3 ^b^; Z5 ^bc^; Z1 ^cd^; Z4 ^d^	0.75	<0.001
Mg	583.4 ± 72.9 ^b^	836.0 ± 94.8 ^a^	657.5 ± 126.0 ^b^	458.9 ± 89.0 ^c^	586.6 ± 108.1 ^b^	Z2 ^a^; Z3/Z5/Z1 ^b^; Z4 ^c^	0.77	<0.001
Na	54.5 ± 6.6 ^c^	125.3 ± 18.9 ^a^	100.7 ± 17.0 ^b^	41.6 ± 6.7 ^c^	87.8 ± 20.0 ^b^	Z2 ^a^; Z3/Z5 ^b^; Z1/Z4 ^c^	0.89	<0.001
P	250.2 ± 27.5 ^b^	338.2 ± 53.8 ^a^	251.1 ± 59.8 ^b^	158.8 ± 32.7 ^c^	267.1 ± 63.3 ^b^	Z2 ^a^; Z5/Z3/Z1 ^b^; Z4 ^c^	0.69	<0.001
S	256.1 ± 71.5 ^bc^	405.7 ± 78.9 ^a^	311.1 ± 98.7 ^b^	195.2 ± 35.0 ^c^	297.1 ± 87.9 ^b^	Z2 ^a^; Z3/Z5 ^b^; Z1 ^bc^; Z4 ^c^	0.68	<0.001
Fe	415.5 ± 90.0 ^c^	749.2 ± 126.4 ^a^	550.0 ± 112.5 ^b^	311.1 ± 89.3 ^c^	612.7 ± 116.1 ^b^	Z2 ^a^; Z5/Z3 ^b^; Z1/Z4 ^c^	0.76	<0.001
Mn	73.7 ± 10.6 ^c^	108.4 ± 17.9 ^a^	89.7 ± 14.8 ^b^	60.3 ± 12.1 ^c^	92.7 ± 15.8 ^b^	Z2 ^a^; Z5/Z3 ^b^; Z1/Z4 ^c^	0.72	<0.001
Zn	27.6 ± 6.5 ^c^	47.0 ± 9.8 ^a^	37.9 ± 9.8 ^b^	24.2 ± 5.5 ^c^	42.5 ± 9.4 ^ab^	Z2 ^a^; Z5 ^ab^; Z3 ^b^; Z1/Z4 ^c^	0.65	<0.001
Cu	9.8 ± 1.3 ^c^	13.6 ± 2.0 ^a^	10.8 ± 1.9 ^bc^	7.6 ± 1.5 ^d^	11.6 ± 1.8 ^b^	Z2 ^a^; Z5 ^b^; Z3 ^bc^; Z1 ^c^; Z4 ^d^	0.70	<0.001
B	12.1 ± 2.7 ^b^	16.9 ± 2.6 ^a^	15.7 ± 3.3 ^a^	11.0 ± 2.6 ^b^	15.4 ± 4.2 ^a^	Z2/Z3/Z5 ^a^; Z1/Z4 ^b^	0.59	<0.001
Mo	0.098 ± 0.031 ^bc^	0.178 ± 0.049 ^a^	0.134 ± 0.046 ^b^	0.090 ± 0.032 ^c^	0.178 ± 0.054 ^a^	Z2/Z5 ^a^; Z3 ^b^; Z1 ^bc^; Z4 ^c^	0.52	<0.001
Co	0.203 ± 0.061 ^cd^	0.433 ± 0.102 ^a^	0.275 ± 0.082 ^bc^	0.167 ± 0.059 ^d^	0.306 ± 0.087 ^b^	Z2 ^a^; Z5 ^b^; Z3 ^bc^; Z1 ^cd^; Z4 ^d^	0.71	<0.001
Se	0.053 ± 0.012 ^ab^	0.052 ± 0.017 ^a^	0.050 ± 0.017 ^ab^	0.038 ± 0.011 ^b^	0.053 ± 0.016 ^a^	Z5/Z2 ^a^; Z1/Z3 ^ab^; Z4 ^b^	0.23	0.026
Al	54.9 ± 15.8 ^d^	137.4 ± 25.3 ^a^	77.9 ± 26.0 ^c^	35.6 ± 8.6 ^d^	99.9 ± 23.3 ^b^	Z2 ^a^; Z5 ^b^; Z3 ^c^; Z1/Z4 ^d^	0.79	<0.001
Ti	3.93 ± 0.97 ^c^	10.84 ± 2.21 ^a^	5.81 ± 1.89 ^b^	2.71 ± 0.77 ^c^	7.32 ± 1.92 ^b^	Z2 ^a^; Z5/Z3 ^b^; Z1/Z4 ^c^	0.81	<0.001
V	0.76 ± 0.15 ^cd^	1.90 ± 0.43 ^a^	0.98 ± 0.26 ^bc^	0.49 ± 0.11 ^d^	1.19 ± 0.39 ^b^	Z2 ^a^; Z5 ^b^; Z3 ^bc^; Z1 ^cd^; Z4 ^d^	0.82	<0.001
Ba	19.9 ± 3.0 ^d^	44.0 ± 4.1 ^a^	24.8 ± 5.0 ^c^	15.0 ± 0.9 ^e^	30.5 ± 3.1 ^b^	Z2 ^a^; Z5 ^b^; Z3 ^c^; Z1 ^d^; Z4 ^e^	0.85	<0.001
Sr	13.0 ± 1.2 ^c^	24.5 ± 2.2 ^a^	15.8 ± 3.3 ^b^	9.1 ± 1.0 ^d^	17.0 ± 2.4 ^b^	Z2 ^a^; Z5/Z3 ^b^; Z1 ^c^; Z4 ^d^	0.86	<0.001
Li	0.57 ± 0.09 ^c^	1.17 ± 0.15 ^a^	0.75 ± 0.13 ^b^	0.33 ± 0.03 ^d^	0.83 ± 0.10 ^b^	Z2 ^a^; Z5/Z3 ^b^; Z1 ^c^; Z4 ^d^	0.87	<0.001
Rb	1.91 ± 0.31 ^c^	3.96 ± 0.45 ^a^	2.71 ± 0.64 ^b^	1.34 ± 0.17 ^d^	2.95 ± 0.39 ^b^	Z2 ^a^; Z5/Z3 ^b^; Z1 ^c^; Z4 ^d^	0.86	<0.001
Cs	0.057 ± 0.010 ^c^	0.135 ± 0.015 ^a^	0.083 ± 0.013 ^b^	0.035 ± 0.005 ^d^	0.090 ± 0.010 ^b^	Z2 ^a^; Z5/Z3 ^b^; Z1 ^c^; Z4 ^d^	0.89	<0.001
Pb	0.103 (1/13)	0.357 ± 0.158 (22/24)	0.144 ± 0.066 (9/20)	<LOD (0/18)	0.199 ± 0.083 (10/19)	Z2 > Z5 > Z3 > Z1; Z4: all <LOD	0.69 †	<0.001 †
Cd	<LOD (0/13)	0.0398 ± 0.0169 (21/24)	0.0198 ± 0.0011 (5/20)	<LOD (0/18)	0.0236 ± 0.0081 (7/19)	Z2 > Z5 > Z3; Z1/Z4: all <LOD	0.66 †	<0.001 †
As	0.019 (1/13)	0.040 ± 0.021 (19/24)	0.025 ± 0.009 (4/20)	<LOD (0/18)	0.032 ± 0.012 (6/19)	Z2 > Z5 > Z3 > Z1; Z4: all <LOD	0.57 †	<0.001 †
Hg	<LOD (0/13)	0.0038 ± 0.0012 (7/24)	0.0024 ± 0.0001 (2/20)	<LOD (0/18)	0.0034 ± 0.0004 (4/19)	Z2 > Z5 > Z3; Z1/Z4: all <LOD	0.29 †	0.005 †
Cr	0.42 ± 0.14 ^cd^	1.40 ± 0.40 ^a^	0.61 ± 0.26 ^bc^	0.27 ± 0.09 ^d^	0.72 ± 0.25 ^b^	Z2 ^a^; Z5 ^b^; Z3 ^bc^; Z1 ^cd^; Z4 ^d^	0.79	<0.001
Ni	0.33 ± 0.10 ^cd^	1.35 ± 0.44 ^a^	0.59 ± 0.21 ^bc^	0.25 ± 0.10 ^d^	0.75 ± 0.25 ^b^	Z2 ^a^; Z5 ^b^; Z3 ^bc^; Z1 ^cd^; Z4 ^d^	0.79	<0.001
Sb	<LOD (0/13)	0.0254 ± 0.0106 (19/24)	0.0091 ± 0.0031 (8/20)	<LOD (0/18)	0.0159 ± 0.0039 (9/19)	Z2 > Z5 > Z3; Z1/Z4: all <LOD	0.61 †	<0.001 †
Sn	0.017 ± 0.004 (9/13)	0.055 ± 0.027 (23/24)	0.029 ± 0.014 (16/20)	0.011 ± 0.003 (8/18)	0.042 ± 0.020 (15/19)	Z2 > Z5 > Z3 > Z1 > Z4	0.62 †	<0.001 †

Note: Values are expressed as mean ± SD (mg/kg DW). Different lowercase superscript letters within a row indicate significant sector differences (*p* < 0.05); values sharing a letter do not differ significantly. Spearman’s *r* describes the association with the spatial gradient Z4 < Z1 < Z5 < Z3 < Z2. For Pb, Cd, As, Hg, Sb, and Sn, parentheses indicate samples ≥LOD/total samples analyzed (e.g., 5/13). Means ± SD were calculated using concentrations ≥LOQ only. “>” indicates descending sector means. † Correlations and *p*-values for these elements included censored (<LOD) observations.

**Table 3:** Spatial variation in the elemental composition of leaves.

Element (mg/kg)	Z1	Z2	Z3	Z4	Z5	Spatial/Statistical Grouping	Spearman’s *r*	*p*-Value
K	22,537 ± 860 ^c^	20,497 ± 774 ^d^	23,909 ± 668 ^b^	25,688 ± 930 ^a^	22,505 ± 1068 ^c^	Z4 ^a^; Z3 ^b^; Z1/Z5 ^c^; Z2 ^d^	−0.68	<0.001
Ca	11,210 ± 992 ^c^	9926 ± 1092 ^d^	12,805 ± 903 ^b^	14,283 ± 1112 ^a^	11,520 ± 1579 ^c^	Z4 ^a^; Z3 ^b^; Z5/Z1 ^c^; Z2 ^d^	−0.57	<0.001
Mg	5354 ± 502 ^cd^	5021 ± 614 ^d^	6349 ± 465 ^ab^	6930 ± 693 ^a^	5906 ± 934 ^bc^	Z4 ^a^; Z3 ^ab^; Z5 ^bc^; Z1 ^cd^; Z2 ^d^	−0.49	<0.001
Na	48.2 ± 1.0 ^c^	56.8 ± 1.2 ^a^	51.4 ± 1.0 ^b^	41.8 ± 1.5 ^e^	46.8 ± 1.3 ^d^	Z2 ^a^; Z3 ^b^; Z1 ^c^; Z5 ^d^; Z4 ^e^	0.93	<0.001
P	3408 ± 150 ^c^	3155 ± 158 ^d^	3607 ± 167 ^ab^	3761 ± 190 ^a^	3462 ± 262 ^bc^	Z4 ^a^; Z3 ^ab^; Z5 ^bc^; Z1 ^c^; Z2 ^d^	−0.57	<0.001
S	2007 ± 113 ^c^	2398 ± 72 ^a^	2198 ± 85 ^b^	1829 ± 126 ^d^	2043 ± 108 ^c^	Z2 ^a^; Z3 ^b^; Z5/Z1 ^c^; Z4 ^d^	0.89	<0.001
Fe	150.6 ± 14.9 ^cd^	173.3 ± 13.6 ^a^	161.4 ± 13.7 ^bc^	138.7 ± 10.6 ^d^	166.3 ± 13.2 ^ab^	Z2 ^a^; Z5 ^ab^; Z3 ^bc^; Z1 ^cd^; Z4 ^d^	0.62	<0.001
Mn	86.6 ± 15.1 ^a^	96.6 ± 16.1 ^a^	86.7 ± 13.4 ^a^	99.2 ± 17.7 ^a^	89.5 ± 17.1 ^a^	Z1/Z2/Z3/Z4/Z5 ^a^	0.01	0.932
Zn	32.5 ± 5.9 ^b^	37.7 ± 5.7 ^a^	34.4 ± 4.8 ^ab^	32.1 ± 3.8 ^b^	36.7 ± 5.4 ^ab^	Z2 ^a^; Z5/Z3 ^ab^; Z1/Z4 ^b^	0.36	<0.001
Cu	8.88 ± 1.04 ^b^	10.51 ± 1.28 ^a^	8.26 ± 1.09 ^b^	7.22 ± 1.07 ^c^	9.16 ± 0.91 ^b^	Z2 ^a^; Z5/Z1/Z3 ^b^; Z4 ^c^	0.59	<0.001
B	33.3 ± 2.4 ^ab^	31.4 ± 3.0 ^b^	28.7 ± 3.8 ^c^	34.9 ± 2.4 ^a^	31.4 ± 3.3 ^bc^	Z4 ^a^; Z1 ^ab^; Z2 ^b^; Z5 ^bc^; Z3 ^c^	−0.41	<0.001
Mo	0.595 ± 0.225 ^bc^	0.806 ± 0.208 ^a^	0.518 ± 0.157 ^c^	0.538 ± 0.134 ^bc^	0.689 ± 0.159 ^ab^	Z2 ^a^; Z5 ^ab^; Z1/Z4 ^bc^; Z3 ^c^	0.34	<0.001
Co	0.352 ± 0.093 ^ab^	0.445 ± 0.117 ^a^	0.355 ± 0.144 ^ab^	0.285 ± 0.116 ^b^	0.386 ± 0.115 ^ab^	Z2 ^a^; Z5/Z3/Z1 ^ab^; Z4 ^b^	0.37	<0.001
Se	0.076 ± 0.029 ^ab^	0.095 ± 0.026 ^a^	0.059 ± 0.024 ^b^	0.055 ± 0.018 ^b^	0.076 ± 0.021 ^ab^	Z2 ^a^; Z5/Z1 ^ab^; Z3/Z4 ^b^	0.37	<0.001
Al	34.7 ± 10.9 ^b^	42.5 ± 4.3 ^a^	38.5 ± 6.2 ^ab^	24.2 ± 5.3 ^c^	33.9 ± 4.9 ^b^	Z2 ^a^; Z3 ^ab^; Z1/Z5 ^b^; Z4 ^c^	0.67	<0.001
Ti	2.25 ± 0.53 ^b^	2.95 ± 0.43 ^a^	2.63 ± 0.40 ^ab^	1.65 ± 0.23 ^c^	2.31 ± 0.31 ^b^	Z2 ^a^; Z3 ^ab^; Z5/Z1 ^b^; Z4 ^c^	0.74	<0.001
V	0.75 ± 0.17 ^bc^	1.02 ± 0.14 ^a^	0.98 ± 0.13 ^a^	0.62 ± 0.12 ^c^	0.86 ± 0.12 ^b^	Z2/Z3 ^a^; Z5 ^b^; Z1 ^bc^; Z4 ^c^	0.72	<0.001
Ba	19.6 ± 2.4 ^c^	25.2 ± 2.7 ^a^	23.1 ± 2.8 ^ab^	16.5 ± 2.3 ^d^	21.0 ± 2.0 ^bc^	Z2 ^a^; Z3 ^ab^; Z5 ^bc^; Z1 ^c^; Z4 ^d^	0.78	<0.001
Sr	12.9 ± 1.1 ^b^	15.7 ± 1.4 ^a^	15.6 ± 1.3 ^a^	11.2 ± 1.6 ^c^	13.6 ± 1.3 ^b^	Z2/Z3 ^a^; Z5/Z1 ^b^; Z4 ^c^	0.76	<0.001
Li	0.59 ± 0.08 ^cd^	0.81 ± 0.08 ^a^	0.70 ± 0.08 ^b^	0.52 ± 0.07 ^d^	0.64 ± 0.06 ^bc^	Z2 ^a^; Z3 ^b^; Z5 ^bc^; Z1 ^cd^; Z4 ^d^	0.80	<0.001
Rb	1.98 ± 0.34 ^cd^	2.80 ± 0.28 ^a^	2.46 ± 0.39 ^b^	1.84 ± 0.23 ^d^	2.15 ± 0.25 ^c^	Z2 ^a^; Z3 ^b^; Z5 ^c^; Z1 ^cd^; Z4 ^d^	0.76	<0.001
Cs	0.059 ± 0.011 ^bc^	0.084 ± 0.011 ^a^	0.077 ± 0.011 ^a^	0.050 ± 0.010 ^c^	0.064 ± 0.009 ^b^	Z2/Z3 ^a^; Z5 ^b^; Z1 ^bc^; Z4 ^c^	0.78	<0.001
Pb	0.041 ± 0.003 (2/13)	0.191 ± 0.044 (21/24)	0.086 ± 0.023 (9/20)	0.042 (1/18)	0.122 ± 0.039 (10/19)	Z2 > Z5 > Z3 > Z4 > Z1	0.65 †	<0.001 †
Cd	<LOD (0/13)	0.0196 ± 0.0050 (16/24)	0.0093 ± 0.0026 (5/20)	<LOD (0/18)	0.0084 ± 0.0012 (3/19)	Z2 > Z3 > Z5; Z1/Z4: all <LOD	0.58 †	<0.001 †
As	0.0095 ± 0.0007 (2/13)	0.0245 ± 0.0078 (17/24)	0.0111 ± 0.0019 (7/20)	0.0070 (1/18)	0.0140 ± 0.0035 (6/19)	Z2 > Z5 > Z3 > Z1 > Z4	0.54 †	<0.001 †
Hg	0.0012 (1/13)	0.0016 ± 0.0004 (6/24)	<LOD (0/20)	<LOD (0/18)	0.0013 (1/19)	Z2 > Z5 > Z1; Z3/Z4: all <LOD	0.27 †	0.010 †
Cr	0.21 ± 0.06 ^c^	0.67 ± 0.23 ^a^	0.38 ± 0.14 ^b^	0.13 ± 0.03 ^c^	0.38 ± 0.10 ^b^	Z2 ^a^; Z5/Z3 ^b^; Z1/Z4 ^c^	0.85	<0.001
Ni	0.17 ± 0.05 (13/13) ^d^	0.70 ± 0.20 (24/24) ^a^	0.35 ± 0.12 (20/20) ^c^	0.14 ± 0.04 (17/18) ^d^	0.47 ± 0.12 (19/19) ^b^	Z2 ^a^; Z5 ^b^; Z3 ^c^; Z1/Z4 ^d^	0.80 †	<0.001 †
Sb	0.0043 ± 0.0002 (2/13)	0.0092 ± 0.0025 (14/24)	0.0055 (1/20)	<LOD (0/18)	0.0063 ± 0.0015 (5/19)	Z2 > Z5 > Z3 > Z1; Z4: all <LOD	0.44 †	<0.001 †
Sn	0.0060 ± 0.0015 (11/13)	0.0216 ± 0.0065 (20/24)	0.0125 ± 0.0038 (15/20)	0.0048 ± 0.0008 (5/18)	0.0164 ± 0.0042 (14/19)	Z2 > Z5 > Z3 > Z1 > Z4	0.59 †	<0.001 †

Note: Values are expressed as mean ± SD (mg/kg DW), with Z1 (*n* = 13), Z2 (*n* = 24), Z3 (*n* = 20), Z4 (*n* = 18), and Z5 (*n* = 19). Sector means, SDs, ANOVA, and Tukey HSD comparisons were based exclusively on concentrations ≥LOQ (*p* < 0.05); values sharing a lowercase letter are not significantly different. For Ni, one <LOD result in Z4 was excluded, reducing the quantitative analysis to *n* = 17 for that sector. Spearman’s *ρ* describes the association with the spatial gradient Z4 < Z1 < Z5 < Z3 < Z2. For Pb, Cd, As, Hg, Sb, and Sn, parentheses indicate samples ≥LOD/total samples analyzed, whereas means ± SD include concentrations ≥LOQ only. “>” indicates descending sector means and is descriptive rather than a post hoc grouping. † In Spearman analyses, <LOD results were treated as tied censored observations ranked below quantitatively reportable concentrations.

**Table 4:** Spatial variation in the elemental composition of flowers.

Element (mg/kg)	Z1	Z2	Z3	Z4	Z5	Spatial/Statistical Grouping	Spearman’s *r*	*p*-Value
K	17,945.4 ± 489.6 ^a^	18,410.0 ± 632.3 ^a^	18,414.1 ± 906.9 ^a^	18,163.6 ± 604.3 ^a^	18,003.8 ± 550.6 ^a^	Z1/Z2/Z3/Z4/Z5 ^a^	0.21	0.044
Ca	6245.5 ± 511.1 ^b^	6942.6 ± 415.8 ^a^	7229.1 ± 429.4 ^a^	6186.3 ± 365.6 ^b^	6134.2 ± 556.4 ^b^	Z3/Z2 ^a^; Z1/Z4/Z5 ^b^	0.60	<0.001
Mg	3983.1 ± 292.1 ^b^	4473.3 ± 347.8 ^a^	4481.3 ± 402.1 ^a^	3983.1 ± 349.5 ^b^	3915.2 ± 386.9 ^b^	Z3/Z2 ^a^; Z1/Z4/Z5 ^b^	0.52	<0.001
Na	47.0 ± 3.0 ^b^	52.4 ± 3.2 ^a^	53.7 ± 3.6 ^a^	46.1 ± 4.1 ^b^	46.4 ± 3.2 ^b^	Z3/Z2 ^a^; Z1/Z5/Z4 ^b^	0.61	<0.001
P ‡	6144.6 ± 271.4 ^bc^	6452.0 ± 257.6 ^a^	6388.6 ± 466.9 ^ab^	5825.6 ± 397.2 ^c^	6066.7 ± 341.7 ^bc^	Z2 ^a^; Z3 ^ab^; Z1/Z5 ^bc^; Z4 ^c^	0.52	<0.001
S	1909.1 ± 198.0 ^ab^	1983.2 ± 124.2 ^a^	1996.7 ± 165.5 ^a^	1823.1 ± 111.9 ^b^	1759.8 ± 162.4 ^b^	Z3/Z2 ^a^; Z1 ^ab^; Z4/Z5 ^b^	0.38	<0.001
Fe	114.2 ± 9.0 ^c^	135.2 ± 10.3 ^a^	124.0 ± 7.9 ^b^	100.1 ± 8.4 ^d^	123.2 ± 8.7 ^b^	Z2 ^a^; Z3/Z5 ^b^; Z1 ^c^; Z4 ^d^	0.77	<0.001
Mn	56.5 ± 6.6 ^b^	68.3 ± 7.5 ^a^	60.6 ± 8.0 ^b^	60.7 ± 7.7 ^b^	60.2 ± 8.7 ^b^	Z2 ^a^; Z4/Z3/Z5/Z1 ^b^	0.38	<0.001
Zn	35.2 ± 5.0 ^b^	42.4 ± 4.1 ^a^	39.4 ± 4.1 ^a^	33.9 ± 2.5 ^b^	39.5 ± 4.4 ^a^	Z2/Z5/Z3 ^a^; Z1/Z4 ^b^	0.60	<0.001
Cu	11.41 ± 0.85 ^b^	13.85 ± 1.29 ^a^	11.52 ± 0.96 ^b^	9.64 ± 0.95 ^c^	11.85 ± 0.90 ^b^	Z2 ^a^; Z5/Z3/Z1 ^b^; Z4 ^c^	0.74	<0.001
B	31.9 ± 2.1 ^ab^	32.9 ± 2.7 ^a^	30.6 ± 2.7 ^b^	32.6 ± 1.6 ^ab^	31.8 ± 2.4 ^ab^	Z2 ^a^; Z4/Z1/Z5 ^ab^; Z3 ^b^	−0.01	0.919
Mo	0.471 ± 0.118 ^bc^	0.663 ± 0.144 ^a^	0.471 ± 0.128 ^bc^	0.433 ± 0.081 ^c^	0.567 ± 0.088 ^ab^	Z2 ^a^; Z5 ^ab^; Z3/Z1 ^bc^; Z4 ^c^	0.48	<0.001
Co	0.133 ± 0.030 ^bc^	0.199 ± 0.037 ^a^	0.154 ± 0.029 ^b^	0.111 ± 0.028 ^c^	0.154 ± 0.030 ^b^	Z2 ^a^; Z3/Z5 ^b^; Z1 ^bc^; Z4 ^c^	0.70	<0.001
Se	0.089 ± 0.021 ^ab^	0.095 ± 0.020 ^a^	0.071 ± 0.017 ^bc^	0.066 ± 0.014 ^c^	0.084 ± 0.018 ^ab^	Z2 ^a^; Z1/Z5 ^ab^; Z3 ^bc^; Z4 ^c^	0.33	0.001
Al ‡	27.9 ± 6.2 ^bcd^	34.6 ± 2.7 ^a^	32.8 ± 3.5 ^ab^	22.8 ± 2.7 ^d^	29.5 ± 2.6 ^c^	Z2 ^a^; Z3 ^ab^; Z5 ^c^; Z1 ^bcd^; Z4 ^d^	0.75	<0.001
Ti ‡	1.72 ± 0.36 ^b^	2.31 ± 0.30 ^a^	2.05 ± 0.27 ^b^	1.32 ± 0.14 ^c^	1.86 ± 0.21 ^b^	Z2 ^a^; Z3/Z5/Z1 ^b^; Z4 ^c^	0.78	<0.001
V	0.57 ± 0.11 ^c^	0.85 ± 0.09 ^a^	0.74 ± 0.08 ^b^	0.48 ± 0.07 ^d^	0.67 ± 0.08 ^b^	Z2 ^a^; Z3/Z5 ^b^; Z1 ^c^; Z4 ^d^	0.85	<0.001
Ba	18.0 ± 2.4 ^d^	24.8 ± 2.1 ^a^	21.8 ± 1.9 ^b^	15.2 ± 1.9 ^e^	19.9 ± 1.6 ^c^	Z2 ^a^; Z3 ^b^; Z5 ^c^; Z1 ^d^; Z4 ^e^	0.87	<0.001
Sr	13.0 ± 1.1 ^b^	16.9 ± 1.4 ^a^	15.9 ± 1.3 ^a^	11.1 ± 1.3 ^c^	13.9 ± 1.1 ^b^	Z2/Z3 ^a^; Z5/Z1 ^b^; Z4 ^c^	0.86	<0.001
Li	0.49 ± 0.06 ^cd^	0.69 ± 0.07 ^a^	0.60 ± 0.07 ^b^	0.45 ± 0.05 ^d^	0.55 ± 0.05 ^bc^	Z2 ^a^; Z3 ^b^; Z5 ^bc^; Z1 ^cd^; Z4 ^d^	0.82	<0.001
Rb	2.24 ± 0.30 ^cd^	3.11 ± 0.29 ^a^	2.82 ± 0.29 ^b^	2.18 ± 0.23 ^d^	2.43 ± 0.22 ^c^	Z2 ^a^; Z3 ^b^; Z5 ^c^; Z1 ^cd^; Z4 ^d^	0.81	<0.001
Cs	0.051 ± 0.009 ^bc^	0.073 ± 0.008 ^a^	0.069 ± 0.009 ^a^	0.045 ± 0.007 ^c^	0.057 ± 0.006 ^b^	Z2/Z3 ^a^; Z5 ^b^; Z1 ^bc^; Z4 ^c^	0.82	<0.001
Pb	0.042 ± 0.007 (4/13)	0.181 ± 0.059 (24/24)	0.065 ± 0.024 (19/20)	0.038 (1/18)	0.091 ± 0.040 (16/19)	Z2 > Z5 > Z3 > Z1 > Z4	0.83 †	<0.001 †
Cd	0.0054 (1/13)	0.0179 ± 0.0061 (24/24)	0.0075 ± 0.0025 (17/20)	<LOD (0/18)	0.0069 ± 0.0014 (13/19)	Z2 > Z3 > Z5 > Z1; Z4: all <LOD	0.86 †	<0.001 †
As	0.008 ± 0.002 (4/13)	0.021 ± 0.007 (24/24)	0.009 ± 0.002 (15/20)	0.007 (1/18)	0.009 ± 0.003 (16/19)	Z2 > Z5 > Z3 > Z1 > Z4	0.80 †	<0.001 †
Hg	<LOD (0/13)	0.0012 ± 0.0003 (9/24)	<LOD (0/20)	<LOD (0/18)	0.0010 (1/19)	Z2 > Z5; Z1/Z3/Z4: all <LOD	0.40 †	<0.001 †
Cr ‡	0.29 ± 0.06 ^c^	0.66 ± 0.14 ^a^	0.39 ± 0.09 ^b^	0.18 ± 0.02 ^d^	0.40 ± 0.08 ^b^	Z2 ^a^; Z5/Z3 ^b^; Z1 ^c^; Z4 ^d^	0.87	<0.001
Ni ‡	0.20 ± 0.02 ^c^	0.67 ± 0.16 ^a^	0.35 ± 0.08 ^b^	0.15 ± 0.03 ^d^	0.42 ± 0.08 ^b^	Z2 ^a^; Z5/Z3 ^b^; Z1 ^c^; Z4 ^d^	0.87	<0.001
Sb	0.0037 ± 0.0005 (2/13)	0.0096 ± 0.0033 (24/24)	0.0041 ± 0.0007 (17/20)	<LOD (0/18)	0.0046 ± 0.0012 (13/19)	Z2 > Z5 > Z3 > Z1; Z4: all <LOD	0.83 †	<0.001 †
Sn	0.008 ± 0.001 (13/13)	0.026 ± 0.007 (24/24)	0.014 ± 0.004 (20/20)	0.005 ± 0.001 (17/18)	0.015 ± 0.005 (19/19)	Z2 > Z5 > Z3 > Z1 > Z4	0.84 †	<0.001 †

Note: Values are expressed as mean ± SD (mg/kg DW), with Z1 (*n* = 13), Z2 (*n* = 24), Z3 (*n* = 20), Z4 (*n* = 18), and Z5 (*n* = 19). Sector differences were assessed using one-way ANOVA followed by Tukey’s HSD; ‡ Welch’s ANOVA and Games–Howell tests were used for P, Al, Ti, Cr, and Ni because of variance heterogeneity. Different lowercase letters indicate significant differences (*p* < 0.05); values sharing a letter do not differ significantly. Spearman’s *r* describes the association with the spatial gradient Z4 < Z1 < Z5 < Z3 < Z2 using individual samples. For Pb, Cd, As, Hg, Sb, and Sn, parentheses indicate detected samples (≥LOD)/total samples analyzed, whereas means ± SD were calculated exclusively from quantitatively reportable concentrations (≥LOQ). A single ≥LOQ result is reported without SD. “>” indicates descending sector means and is descriptive only. † In Spearman analyses, <LOD results were treated as tied censored observations ranked below quantitatively reportable concentrations.

**Table 5:** Spatial variation in the elemental composition of fruits.

Element (mg/kg)	Z1	Z2	Z3	Z4	Z5	Spatial/Statistical Grouping	Spearman’s *r*	*p*-Value
K	3401.5 ± 260.1 ^b^	4184.8 ± 239.7 ^a^	3680.2 ± 491.8 ^b^	2521.6 ± 295.4 ^c^	3637.8 ± 497.5 ^b^	Z2 ^a^; Z3/Z5/Z1 ^b^; Z4 ^c^	0.79	<0.001
Ca	2170.8 ± 176.2 ^b^	2572.3 ± 198.8 ^a^	2319.2 ± 173.8 ^b^	1875.9 ± 276.0 ^c^	2354.1 ± 251.8 ^b^	Z2 ^a^; Z5/Z3/Z1 ^b^; Z4 ^c^	0.69	<0.001
Mg	874.6 ± 82.7 ^c^	1075.6 ± 77.3 ^a^	934.0 ± 141.8 ^bc^	743.1 ± 88.1 ^d^	983.3 ± 114.4 ^b^	Z2 ^a^; Z5 ^b^; Z3 ^bc^; Z1 ^c^; Z4 ^d^	0.69	<0.001
Na	65.4 ± 14.0 ^b^	91.8 ± 13.4 ^a^	79.2 ± 20.1 ^ab^	47.3 ± 11.7 ^c^	76.7 ± 17.7 ^b^	Z2 ^a^; Z3 ^ab^; Z5/Z1 ^b^; Z4 ^c^	0.67	<0.001
P	1300.7 ± 125.3 ^b^	2053.1 ± 174.2 ^a^	1406.3 ± 184.8 ^b^	1316.7 ± 194.2 ^b^	1462.7 ± 228.7 ^b^	Z2 ^a^; Z5/Z3/Z4/Z1 ^b^	0.68	<0.001
S	1795.8 ± 245.8 ^c^	2682.9 ± 184.8 ^a^	1946.0 ± 311.8 ^c^	1441.9 ± 226.1 ^d^	2435.4 ± 344.4 ^b^	Z2 ^a^; Z5 ^b^; Z3/Z1 ^c^; Z4 ^d^	0.71	<0.001
Fe	36.1 ± 2.8 ^c^	44.9 ± 3.0 ^a^	39.1 ± 3.0 ^b^	31.8 ± 2.5 ^d^	40.5 ± 2.4 ^b^	Z2 ^a^; Z5/Z3 ^b^; Z1 ^c^; Z4 ^d^	0.81	<0.001
Mn	15.5 ± 1.7 ^b^	19.5 ± 2.2 ^a^	16.9 ± 2.2 ^b^	17.1 ± 1.9 ^b^	17.6 ± 2.7 ^b^	Z2 ^a^; Z5/Z4/Z3/Z1 ^b^	0.39	<0.001
Zn	11.1 ± 1.6 ^cd^	13.9 ± 1.5 ^a^	12.3 ± 1.4 ^bc^	10.9 ± 1.2 ^d^	13.1 ± 1.5 ^ab^	Z2 ^a^; Z5 ^ab^; Z3 ^bc^; Z1 ^cd^; Z4 ^d^	0.57	<0.001
Cu	4.78 ± 0.27 ^c^	5.89 ± 0.52 ^a^	4.84 ± 0.44 ^c^	4.15 ± 0.43 ^d^	5.24 ± 0.36 ^b^	Z2 ^a^; Z5 ^b^; Z3/Z1 ^c^; Z4 ^d^	0.72	<0.001
B	17.4 ± 1.0 ^ab^	18.2 ± 1.4 ^a^	16.2 ± 1.6 ^b^	18.0 ± 1.2 ^a^	17.2 ± 1.4 ^ab^	Z2/Z4 ^a^; Z1/Z5 ^ab^; Z3 ^b^	0.004	0.971
Mo	0.221 ± 0.060 ^bc^	0.301 ± 0.053 ^a^	0.211 ± 0.048 ^c^	0.202 ± 0.038 ^c^	0.262 ± 0.044 ^ab^	Z2 ^a^; Z5 ^ab^; Z1 ^bc^; Z3/Z4 ^c^	0.48	<0.001
Co	0.052 ± 0.010 ^bc^	0.078 ± 0.014 ^a^	0.059 ± 0.010 ^b^	0.044 ± 0.010 ^c^	0.063 ± 0.013 ^b^	Z2 ^a^; Z5/Z3 ^b^; Z1 ^bc^; Z4 ^c^	0.66	<0.001
Se	0.052 ± 0.013 ^ab^	0.059 ± 0.012 ^a^	0.044 ± 0.011 ^bc^	0.040 ± 0.008 ^c^	0.051 ± 0.010 ^ab^	Z2 ^a^; Z1/Z5 ^ab^; Z3 ^bc^; Z4 ^c^	0.41	<0.001
Al	8.4 ± 2.1 ^b^	10.6 ± 0.8 ^a^	9.5 ± 1.1 ^b^	6.6 ± 0.8 ^c^	8.8 ± 0.8 ^b^	Z2 ^a^; Z3/Z5/Z1 ^b^; Z4 ^c^	0.76	<0.001
Ti	0.52 ± 0.10 ^c^	0.73 ± 0.10 ^a^	0.61 ± 0.07 ^b^	0.39 ± 0.05 ^d^	0.57 ± 0.06 ^bc^	Z2 ^a^; Z3 ^b^; Z5 ^bc^; Z1 ^c^; Z4 ^d^	0.82	<0.001
V	0.18 ± 0.04 ^c^	0.26 ± 0.03 ^a^	0.23 ± 0.03 ^b^	0.15 ± 0.02 ^d^	0.21 ± 0.03 ^b^	Z2 ^a^; Z3/Z5 ^b^; Z1 ^c^; Z4 ^d^	0.81	<0.001
Ba	6.2 ± 0.8 ^c^	8.5 ± 0.7 ^a^	7.4 ± 0.7 ^b^	5.2 ± 0.7 ^d^	7.0 ± 0.6 ^b^	Z2 ^a^; Z3/Z5 ^b^; Z1 ^c^; Z4 ^d^	0.86	<0.001
Sr	4.5 ± 0.4 ^b^	5.7 ± 0.5 ^a^	5.4 ± 0.4 ^a^	3.8 ± 0.5 ^c^	4.7 ± 0.4 ^b^	Z2/Z3 ^a^; Z5/Z1 ^b^; Z4 ^c^	0.83	<0.001
Li	0.14 ± 0.02 ^c^	0.20 ± 0.02 ^a^	0.17 ± 0.02 ^b^	0.12 ± 0.01 ^c^	0.16 ± 0.02 ^b^	Z2 ^a^; Z3/Z5 ^b^; Z1/Z4 ^c^	0.83	<0.001
Rb	0.75 ± 0.12 ^cd^	1.02 ± 0.10 ^a^	0.92 ± 0.12 ^b^	0.71 ± 0.09 ^d^	0.83 ± 0.08 ^c^	Z2 ^a^; Z3 ^b^; Z5 ^c^; Z1 ^cd^; Z4 ^d^	0.76	<0.001
Cs	0.013 ± 0.002 ^d^	0.020 ± 0.002 ^a^	0.018 ± 0.002 ^b^	0.012 ± 0.002 ^d^	0.016 ± 0.002 ^c^	Z2 ^a^; Z3 ^b^; Z5 ^c^; Z1/Z4 ^d^	0.81	<0.001
Pb	<LOD (0/13)	0.078 ± 0.016 (21/24)	0.039 ± 0.004 (5/20)	<LOD (0/18)	0.050 ± 0.008 (9/19)	Z2 > Z5 > Z3; Z1/Z4: all <LOD	0.66 †	<0.001 †
Cd	<LOD (0/13)	0.0078 ± 0.0012 (16/24)	0.0052 ± 0.0001 (2/20)	<LOD (0/18)	<LOD (0/19)	Z2 > Z3; Z1/Z4/Z5: all <LOD	0.60 †	<0.001 †
As	<LOD (0/13)	0.0099 ± 0.0019 (17/24)	<LOD (0/20)	<LOD (0/18)	0.0070 ± 0.0000 (2/19)	Z2 > Z5; Z1/Z3/Z4: all <LOD	0.59 †	<0.001 †
Hg	<LOD (0/13)	<LOD (0/24)	<LOD (0/20)	<LOD (0/18)	<LOD (0/19)	All sectors: <LOD	**—**	**—**
Cr	0.097 ± 0.019 ^c^ (13/13)	0.262 ± 0.065 ^a^ (24/24)	0.145 ± 0.036 ^b^ (20/20)	0.066 ± 0.006 ^c^ (16/18)	0.150 ± 0.032 ^b^ (19/19)	Z2 ^a^; Z5/Z3 ^b^; Z1/Z4 ^c^	0.89 †	<0.001 †
Ni	0.082 ± 0.011 ^c^ (13/13)	0.275 ± 0.066 ^a^ (24/24)	0.148 ± 0.039 ^b^ (20/20)	0.072 ± 0.008 ^c^ (13/18)	0.181 ± 0.031 ^b^ (19/19)	Z2 ^a^; Z5/Z3 ^b^; Z1/Z4 ^c^	0.85 †	<0.001 †
Sb	<LOD (0/13)	0.0041 ± 0.0008 (16/24)	<LOD (0/20)	<LOD (0/18)	<LOD (0/19)	Z2; Z1/Z3/Z4/Z5: all <LOD	0.59 †	<0.001 †
Sn	0.0040 ± 0.0000 (4/13)	0.0098 ± 0.0031 (24/24)	0.0060 ± 0.0013 (16/20)	<LOD (0/18)	0.0069 ± 0.0014 (15/19)	Z2 > Z5 > Z3 > Z1; Z4: all <LOD	0.78 †	<0.001 †

Note: Values are expressed as mean ± SD (mg/kg DW) for the five ecological sectors (Z1, *n* = 13; Z2, *n* = 24; Z3, *n* = 20; Z4, *n* = 18; Z5, *n* = 19). Different lowercase superscript letters indicate significant differences among sectors according to one-way ANOVA followed by Tukey’s HSD test (*p* < 0.05). Spearman’s *r* describes the association with the ordinal spatial gradient Z4 < Z1 < Z5 < Z3 < Z2. For censored elements, values in parentheses indicate the number of samples ≥LOD. Detection was defined as ≥LOD, whereas sector means, SDs, and ANOVA/Tukey analyses were based exclusively on quantitatively reportable concentrations ≥LOQ; observations between LOD and LOQ were not used numerically. For Pb, Cd, As, Sb, and Sn, “>” indicates only the descriptive descending order of sector means and does not imply significant pairwise differences. “All <LOD” indicates that no sample in the respective sector had a concentration ≥LOD. † For Spearman analysis, observations <LOD were assigned tied ranks below the lowest detected concentration. No correlation or inferential comparison was performed for Hg because all fruit samples were <LOD.

**Table 6:** Site-paired soil-to-organ concentration ratios (TFs) and Spearman correlations (r_s_) between soil and organ concentrations for the investigated elements in *Sambucus nigra*.

Element	Bark	Leaves	Flowers	Fruits	Organ-Specific TF Hierarchy	Preferential Organ
Macroelements						
K	TF: 0.864 [0.736–0.996] r_s_: 0.60 ***	TF: 10.85 [8.68–13.70] r_s_: −0.66 ***	TF: 8.69 [7.31–10.24] r_s_: 0.18 ns	TF: 1.61 [1.48–1.86] r_s_: 0.69 ***	Leaves > Flowers > Fruits > Bark	Leaves
Ca	TF: 0.403 [0.352–0.478] r_s_: 0.62 ***	TF: 1.23 [0.959–1.67] r_s_: −0.59 ***	TF: 0.679 [0.606–0.804] r_s_: 0.43 ***	TF: 0.239 [0.215–0.268] r_s_: 0.58 ***	Leaves > Flowers > Bark > Fruits	Leaves
Mg	TF: 0.441 [0.378–0.500] r_s_: 0.64 ***	TF: 3.94 [3.23–5.29] r_s_: −0.50 ***	TF: 2.93 [2.48–3.41] r_s_: 0.43 ***	TF: 0.640 [0.559–0.729] r_s_: 0.53 ***	Leaves > Flowers > Fruits > Bark	Leaves
Na	TF: 0.203 [0.160–0.256] r_s_: 0.71 ***	TF: 0.129 [0.096–0.171] r_s_: 0.76 ***	TF: 0.124 [0.093–0.169] r_s_: 0.50 ***	TF: 0.182 [0.142–0.234] r_s_: 0.57 ***	Bark > Fruits > Leaves > Flowers	Bark
P	TF: 5.18 [4.24–6.48] r_s_: 0.52 ***	TF: 74.93 [47.69–102.81] r_s_: −0.57 ***	TF: 128.91 [91.40–173.76] r_s_: 0.53 ***	TF: 32.38 [24.65–38.47] r_s_: 0.42 ***	Flowers > Leaves > Fruits > Bark	Flowers
S	TF: 0.265 [0.227–0.343] r_s_: 0.52 ***	TF: 2.10 [1.63–2.48] r_s_: 0.67 ***	TF: 1.84 [1.41–2.19] r_s_: 0.36 ***	TF: 1.94 [1.65–2.43] r_s_: 0.56 ***	Leaves > Fruits > Flowers > Bark	Leaves
Microelements and biologically relevant trace elements						
Fe	TF: 0.0152 [0.0128–0.0170] r_s_: 0.85 ***	TF: 0.00443 [0.00380–0.00529] r_s_: 0.45 ***	TF: 0.00333 [0.00306–0.00380] r_s_: 0.76 ***	TF: 0.00107 [0.00098–0.00123] r_s_: 0.76 ***	Bark > Leaves > Flowers > Fruits	Bark
Mn	TF: 0.108 [0.099–0.126] r_s_: 0.89 ***	TF: 0.114 [0.090–0.152] r_s_: 0.10 ns	TF: 0.0786 [0.064–0.097] r_s_: 0.49 ***	TF: 0.0224 [0.0185–0.0265] r_s_: 0.61 ***	Leaves > Bark > Flowers > Fruits	Leaves
Zn	TF: 0.363 [0.335–0.401] r_s_: 0.94 ***	TF: 0.353 [0.259–0.478] r_s_: 0.21 *	TF: 0.376 [0.308–0.509] r_s_: 0.54 ***	TF: 0.123 [0.097–0.161] r_s_: 0.50 ***	Flowers > Bark > Leaves > Fruits	Flowers
Cu	TF: 0.264 [0.238–0.291] r_s_: 0.91 ***	TF: 0.214 [0.174–0.275] r_s_: 0.40 ***	TF: 0.282 [0.242–0.346] r_s_: 0.66 ***	TF: 0.119 [0.103–0.146] r_s_: 0.69 ***	Flowers > Bark > Leaves > Fruits	Flowers
B	TF: 0.725 [0.626–0.826] r_s_: 0.85 ***	TF: 1.54 [1.21–2.19] r_s_: −0.30 **	TF: 1.62 [1.20–2.09] r_s_: 0.12 ns	TF: 0.851 [0.676–1.14] r_s_: 0.18 ns	Flowers > Leaves > Fruits > Bark	Flowers
Mo	TF: 0.0655 [0.054–0.084] r_s_: 0.73 ***	TF: 0.328 [0.230–0.437] r_s_: 0.26 *	TF: 0.266 [0.202–0.346] r_s_: 0.49 ***	TF: 0.121 [0.092–0.155] r_s_: 0.50 ***	Leaves > Flowers > Fruits > Bark	Leaves
Co	TF: 0.0205 [0.0172–0.0226] r_s_: 0.93 ***	TF: 0.0258 [0.0193–0.0374] r_s_: 0.27 **	TF: 0.0107 [0.0092–0.0136] r_s_: 0.68 ***	TF: 0.00427 [0.00370–0.00540] r_s_: 0.70 ***	Leaves > Bark > Flowers > Fruits	Leaves
Se	TF: 0.130 [0.096–0.203] r_s_: 0.51 ***	TF: 0.180 [0.121–0.396] r_s_: 0.12 ns	TF: 0.204 [0.159–0.385] r_s_: 0.44 ***	TF: 0.127 [0.095–0.251] r_s_: 0.33 **	Flowers > Leaves > Bark > Fruits	Flowers
Lithogenic and geochemically associated elements						
Al	TF: 0.00270 [0.00163–0.00513] r_s_: −0.66 ***	TF: 0.00124 [0.00091–0.00181] r_s_: −0.57 ***	TF: 0.00106 [0.00081–0.00157] r_s_: −0.52 ***	TF: 0.00031 [0.00024–0.00047] r_s_: −0.60 ***	Bark > Leaves > Flowers > Fruits	Bark
Ti	TF: 0.0205 [0.0149–0.0388] r_s_: −0.21 *	TF: 0.00942 [0.00749–0.0119] r_s_: −0.15 ns	TF: 0.00723 [0.00605–0.00921] r_s_: −0.14 ns	TF: 0.00214 [0.00181–0.00285] r_s_: −0.20 ns	Bark > Leaves > Flowers > Fruits	Bark
V	TF: 0.0143 [0.00943–0.0203] r_s_: 0.32 **	TF: 0.0119 [0.00998–0.0136] r_s_: 0.18 ns	TF: 0.00957 [0.00798–0.0107] r_s_: 0.34 ***	TF: 0.00295 [0.00251–0.00327] r_s_: 0.30 **	Bark > Leaves > Flowers > Fruits	Bark
Ba	TF: 0.109 [0.0918–0.143] r_s_: 0.74 ***	TF: 0.0901 [0.0775–0.0999] r_s_: 0.56 ***	TF: 0.0853 [0.0757–0.0948] r_s_: 0.68 ***	TF: 0.0294 [0.0258–0.0324] r_s_: 0.66 ***	Bark > Leaves > Flowers > Fruits	Bark
Sr	TF: 0.176 [0.140–0.222] r_s_: 0.63 ***	TF: 0.159 [0.125–0.195] r_s_: 0.51 ***	TF: 0.162 [0.128–0.197] r_s_: 0.57 ***	TF: 0.0554 [0.0437–0.0663] r_s_: 0.54 ***	Bark > Flowers > Leaves > Fruits	Bark
Li	TF: 0.0208 [0.0147–0.0505] r_s_: −0.74 ***	TF: 0.0197 [0.0134–0.0407] r_s_: −0.55 ***	TF: 0.0168 [0.0116–0.0351] r_s_: −0.54 ***	TF: 0.00481 [0.00330–0.00997] r_s_: −0.55 ***	Bark > Leaves > Flowers > Fruits	Bark
Rb	TF: 0.0228 [0.0160–0.0458] r_s_: −0.69 ***	TF: 0.0197 [0.0144–0.0339] r_s_: −0.55 ***	TF: 0.0222 [0.0179–0.0375] r_s_: −0.51 ***	TF: 0.00749 [0.00557–0.0129] r_s_: −0.52 ***	Bark > Flowers > Leaves > Fruits	Bark
Cs	TF: 0.0131 [0.00945–0.0352] r_s_: −0.75 ***	TF: 0.0114 [0.00792–0.0221] r_s_: −0.54 ***	TF: 0.00996 [0.00726–0.0201] r_s_: −0.53 ***	TF: 0.00273 [0.00190–0.00524] r_s_: −0.55 ***	Bark > Leaves > Flowers > Fruits	Bark
Potentially toxic and environmentally relevant trace elements						
Pb	TF: 0.00834 [0.00501–0.0109] r_s_: 0.54 ***	TF: 0.00439 [0.00305–0.00544] r_s_: 0.58 ***	TF: 0.00340 [0.00222–0.00471] r_s_: 0.76 ***	TF: 0.00183 [0.00143–0.00222] r_s_: 0.61 ***	Bark > Leaves > Flowers > Fruits	Bark
Cd	TF: 0.0543 [0.0388–0.0918] r_s_: 0.48 ***	TF: 0.0247 [0.0172–0.0408] r_s_: 0.47 ***	TF: 0.0179 [0.0127–0.0262] r_s_: 0.82 ***	TF: 0.0110 [0.00773–0.0150] r_s_: 0.52 ***	Bark > Leaves > Flowers > Fruits	Bark
As	TF: 0.00547 [0.00444–0.0111] r_s_: 0.54 ***	TF: 0.00441 [0.00288–0.00520] r_s_: 0.39 ***	TF: 0.00278 [0.00204–0.00383] r_s_: 0.74 ***	TF: 0.00158 [0.00112–0.00177] r_s_: 0.63 ***	Bark > Leaves > Flowers > Fruits	Bark
Hg	TF: 0.0435 [0.0246–0.0857] r_s_: 0.29 **	TF: 0.0126 [0.00968–0.0322] r_s_: 0.19 ns	TF: 0.00941 [0.00683–0.0139] r_s_: 0.35 ***	ND	Bark > Leaves > Flowers; Fruit ND	Bark
Cr	TF: 0.0131 [0.00837–0.0190] r_s_: 0.52 ***	TF: 0.00707 [0.00496–0.00969] r_s_: 0.49 ***	TF: 0.00798 [0.00622–0.00936] r_s_: 0.64 ***	TF: 0.00293 [0.00220–0.00381] r_s_: 0.58 ***	Bark > Flowers > Leaves > Fruits	Bark
Ni	TF: 0.0169 [0.0112–0.0247] r_s_: 0.53 ***	TF: 0.00924 [0.00612–0.0138] r_s_: 0.54 ***	TF: 0.00928 [0.00697–0.0128] r_s_: 0.64 ***	TF: 0.00400 [0.00310–0.00536] r_s_: 0.63 ***	Bark > Flowers > Leaves > Fruits	Bark
Sb	TF: 0.00813 [0.00435–0.0143] r_s_: 0.56 ***	TF: 0.00457 [0.00239–0.00670] r_s_: 0.33**	TF: 0.00267 [0.00211–0.00371] r_s_: 0.81 ***	TF: 0.00121 [0.00086–0.00138] r_s_: 0.57 ***	Bark > Leaves > Flowers > Fruits	Bark
Sn	TF: 0.00708 [0.00443–0.0118] r_s_: 0.37 ***	TF: 0.00283 [0.00208–0.00412] r_s_: 0.54 ***	TF: 0.00333 [0.00252–0.00470] r_s_: 0.79 ***	TF: 0.00134 [0.00106–0.00185] r_s_: 0.74 ***	Bark > Flowers > Leaves > Fruits	Bark

Note: Elemental concentrations are expressed on a dry-weight basis (mg/kg DW). The soil-to-organ concentration ratio (TF) was calculated for each georeferenced soil–plant pair as TF = *C*organ/*C*soil and is reported as median [Q1–Q3]. All 94 paired sites were included when no results were <LOQ; for Pb, Cd, As, Hg, Cr, Ni, Sb, and Sn, TFs were calculated only when both soil and organ concentrations were ≥LOQ, resulting in variable effective sample sizes (*n*TF). Cases without valid quantifiable pairs are reported as ND. Spearman’s r_s_ was calculated across the 94 sites, with <LOD observations retained as tied censored values ranked below the lowest detected concentration. Statistical significance was defined as * *p* < 0.05, ** *p* < 0.01, and *** *p* < 0.001; ns, not significant. Organ-specific TF rankings were based descriptively on median values and do not represent statistical pairwise comparisons. Q1, first quartile; Q3, third quartile; TF, soil-to-organ concentration ratio; r_s_, Spearman’s rank correlation coefficient; LOD, limit of detection; LOQ, limit of quantification; ND, not determined.

**Table 7:** Occurrence, distributional characteristics, and spatial trends of potentially toxic and environmentally relevant trace elements in edible *Sambucus nigra* L. fruits.

Element	Overall Detection, n/94 (%) [95% CI]	Mean ± SD * (mg/kg DW)	*CV* (%) *	Median [Q1–Q3] *	*P95* (mg/kg *DW*) *	Observed Range * (mg/kg DW)	Maximum Concentration; Sampling Point	Detection Heterogeneity Among Sectors †, *p*	Cramér’s V †	Spearman *r*_s_ ‡	*p* ‡
Pb	35/94 (37.2%) [28.1–47.3]	0.065 ± 0.021	31.9	0.064 [0.044–0.082]	0.095	0.035–0.105	0.105; SN-FR-065 (Z2)	<0.001	0.703	0.66	<0.001
Cd	18/94 (19.1%) [12.5–28.3]	0.0075 ± 0.0014	18.5	0.0077 [0.0071–0.0083]	0.0092	0.0051–0.0104	0.0104; SN-FR-087 (Z2)	<0.001	0.714	0.60	<0.001
As	19/94 (20.2%) [13.3–29.4]	0.0096 ± 0.0020	21.1	0.0090 [0.0085–0.0100]	0.0140	0.0070–0.0140	0.014; SN-FR-014/SN-FR-094 (Z2)	<0.001	0.745	0.59	<0.001
Hg	0/94 (0.0%) [0.0–3.9]	—	—	—	—	—	All samples <LOD	—	—	—	—
Cr	92/94 (97.9%) [92.6–99.4]	0.156 ± 0.081	51.8	0.140 [0.090–0.200]	0.315	0.060–0.460	0.460; SN-FR-064 (Z2)	0.053	0.303	0.89	<0.001
Ni	89/94 (94.7%) [88.1–97.7]	0.168 ± 0.086	51.2	0.160 [0.090–0.220]	0.320	0.060–0.410	0.410; SN-FR-094 (Z2)	<0.001	0.487	0.85	<0.001
Sb	16/94 (17.0%) [10.8–25.9]	0.0041 ± 0.0008	20.5	0.0039 [0.0035–0.0045]	0.0058	0.0031–0.0059	0.0059; SN-FR-050 (Z2)	<0.001	0.773	0.59	<0.001
Sn	59/94 (62.8%) [52.7–71.9]	0.0076 ± 0.0029	38.3	0.0070 [0.0060–0.0095]	0.0131	0.0040–0.0160	0.016; SN-FR-065 (Z2)	<0.001	0.765	0.78	<0.001

Note: Elemental concentrations are expressed on a dry-weight basis (mg/kg DW) for 94 fruit samples collected across Z1–Z5. Overall detection was defined as the number and percentage of samples with concentrations ≥LOD, with 95% Wilson confidence intervals. The investigated potentially toxic elements were Pb, Cd, As, Hg, Cr, Ni, Sb, and Sn. * Mean ± SD, CV, median [Q1–Q3], P95, range, and maximum were calculated using observations ≥LOQ. No PTE result included in these quantitative descriptors fell between LOD and LOQ. CV was calculated as (SD/mean) × 100. † Differences in detection frequency among sectors were evaluated using the Fisher–Freeman–Halton exact test, with Cramér’s V as effect size. ‡ Spearman’s rank correlation coefficient (r_s_) assessed associations with the predefined spatial gradient Z4 < Z1 < Z5 < Z3 < Z2. For datasets containing <LOD observations, these were retained as tied censored values below the lowest detected concentration. For Hg, all 94 samples were <LOD; therefore, concentration descriptors and detection-related statistical analyses were not calculated. LOD, limit of detection; LOQ, limit of quantification; SD, standard deviation; CV, coefficient of variation; Q1–Q3, first and third quartiles; P95, 95th percentile; CI, confidence interval.

**Table 8:** Detection prevalence, sector heterogeneity, and effect-size statistics for potentially toxic elements in edible *Sambucus nigra* fruits.

Element	Overall Detection n/94 (%)	95% CI	Five-Sector Heterogeneity, *p* †	Cramér’s V	Z2 Detection	Non-Z2 Detection	Prevalence Ratio Z2/Non-Z2 [95% CI]	Fisher *p* ‡
Pb	35/94 (37.2%)	28.1–47.3	<0.001	0.703	21/24 (87.5%)	14/70 (20.0%)	4.38 [2.67–7.16]	<0.001
Cd	18/94 (19.1%)	12.5–28.3	<0.001	0.714	16/24 (66.7%)	2/70 (2.9%)	23.33 [5.78–94.15]	<0.001
As	19/94 (20.2%)	13.3–29.4	<0.001	0.745	17/24 (70.8%)	2/70 (2.9%)	24.79 [6.18–99.53]	<0.001
Hg	0/94 (0.0%)	0.0–3.9	—	—	0/24	0/70	NE §	—
Cr	92/94 (97.9%)	92.6–99.4	0.053	0.303	24/24 (100%)	68/70 (97.1%)	1.03 [0.95–1.09]	1.000
Ni	89/94 (94.7%)	88.1–97.7	<0.001	0.487	24/24 (100%)	65/70 (92.9%)	1.08 [0.98–1.17]	0.324
Sb	16/94 (17.0%)	10.8–25.9	<0.001	0.773	16/24 (66.7%)	0/70 (0.0%)	NE §	<0.001
Sn	59/94 (62.8%)	52.7–71.9	<0.001	0.765	24/24 (100%)	35/70 (50.0%)	2.00 [1.58–2.53]	<0.001

Note: Overall detection prevalence is expressed as the number and percentage of samples with concentrations ≥LOD; 95% confidence intervals (CIs) for detection prevalence were calculated using the Wilson score method. † Differences in detection status among Z1–Z5 were evaluated using the Fisher–Freeman–Halton exact test for the 5 × 2 contingency table; Cramér’s V quantifies the strength of the association between ecological sector and detection status. ‡ The Z2 versus non-Z2 comparison was evaluated using Fisher’s exact test. Prevalence ratios (PRs) compare the detection prevalence in Z2 with that in all remaining sectors combined (non-Z2); 95% CIs for PRs were estimated on the logarithmic scale using the Katz method. § The prevalence ratio could not be conventionally estimated for Sb because no Sb detections occurred in the non-Z2 group. NE = not estimable; LOD = limit of detection.

**Table 9:** Multi-element detection burden of potentially toxic elements in individual edible fruit samples.

Sector	*n*	Detected PTEs/Sample, Mean ± SD	Median [Q1–Q3]	Range	≥3 PTEs, *n* (%)	≥4 PTEs, *n* (%)	≥5 PTEs, *n* (%)	Statistical Grouping †
Z1	13	2.31 ± 0.48	2 [2–3]	2–3	4 (30.8%)	0 (0%)	0 (0%)	c
Z2	24	5.92 ± 1.10	6 [5.75–7]	3–7	24 (100%)	23 (95.8%)	21 (87.5%)	a
Z3	20	3.15 ± 0.49	3 [3–3]	2–4	19 (95.0%)	4 (20.0%)	0 (0%)	b
Z4	18	1.61 ± 0.61	2 [1–2]	0–2	0 (0%)	0 (0%)	0 (0%)	d
Z5	19	3.37 ± 0.68	3 [3–4]	2–4	17 (89.5%)	9 (47.4%)	0 (0%)	b

Note: PTEs = potentially toxic elements. The multi-element detection burden was defined for each individual fruit sample as the number of potentially toxic elements detected at or above the analytical limit of detection (≥LOD) among the eight investigated elements (Pb, Cd, As, Hg, Cr, Ni, Sb, and Sn); therefore, the theoretical score ranged from 0 to 8 detected PTEs per sample. Because Hg was <LOD in all samples, the maximum observed score was 7. Values are summarized by ecological sector as mean ± SD, median [Q1–Q3], and observed range. The columns ≥3 PTEs, ≥4 PTEs, and ≥5 PTEs indicate the number and percentage of fruit samples in which at least 3, 4, or 5 PTEs, respectively, were simultaneously detected. Samples with concentrations <LOD were classified as non-detections, and no numerical substitution was applied. † Differences among sectors were evaluated using the Kruskal–Wallis test (*H*(4) = 75.76, *p* < 0.001; ε^2^ = 0.806), followed by Mann–Whitney U tests with Holm adjustment. Different lowercase letters indicate significant differences at adjusted *p* < 0.05. Q1 = first quartile; Q3 = third quartile; SD = standard deviation; LOD = limit of detection; ε^2^ = epsilon-squared effect size.

**Table 10:** Regulatory assessment of Pb and Cd concentrations in edible *Sambucus nigra* L. fruits relative to the maximum levels established by Commission Regulation (EU) 2023/915.

Element	Detection, n/94 (%)	EU ML (mg/kg WW)	Maximum Concentration (mg/kg WW)	Sample (Sector)	Maximum %ML
Pb	35/94 (37.2%)	0.200	0.0214	SN-FR-065 (Z2)	10.70%
Cd	18/94 (19.1%)	0.030	0.00216	SN-FR-087 (Z2)	7.20%

Note: ML = maximum level; WW = wet weight; LOD = limit of detection. Detection represents the number and percentage of samples with concentrations ≥LOD. The percentage of the applicable maximum level was calculated for each sample as %ML = (*C*WW/ML) × 100, where *C*WW is the concentration in mg/kg WW and ML is the applicable maximum level. According to Commission Regulation (EU) 2023/915, the MLs are 0.20 mg/kg WW for Pb in elderberries and 0.030 mg/kg WW for Cd in berries and small fruits and apply to the washed edible portion. No sample exceeded either ML.

**Table 11:** Estimated dietary exposure to potentially toxic elements associated with the consumption of edible *Sambucus nigra* L. fruits under central and upper-percentile exposure scenarios.

Element	Detection n/94 (%)	Mean MB Concentration (mg/kg WW)	P95 MB Concentration (mg/kg WW)	Central EDI (mg/kg bw/day)	P95 EDI (mg/kg bw/day)	LB–UB EDI (mg/kg bw/day)
Pb	35/94 (37.2)	0.005111	0.018403	7.301 × 10^−6^	2.629 × 10^−5^	7.277 × 10^−6^–7.324 × 10^−6^
Cd	18/94 (19.1)	0.0003179	0.001694	4.542 × 10^−7^	2.420 × 10^−6^	4.340 × 10^−7^–4.744 × 10^−7^
As	19/94 (20.2)	0.0004547	0.002091	6.495 × 10^−7^	2.988 × 10^−6^	5.796 × 10^−7^–7.195 × 10^−7^
Hg	0/94 (0.0)	0.00003498	0.00003708	4.998 × 10^−8^	5.296 × 10^−8^	0–9.996 × 10^−8^
Cr	92/94 (97.9)	0.032087	0.065443	4.584 × 10^−5^	9.349 × 10^−5^	4.583 × 10^−5^–4.584 × 10^−5^
Ni	89/94 (94.7)	0.033517	0.069493	4.788 × 10^−5^	9.928 × 10^−5^	4.787 × 10^−5^–4.789 × 10^−5^
Sb	16/94 (17.0)	0.0001706	0.0009387	2.438 × 10^−7^	1.341 × 10^−6^	2.128 × 10^−7^–2.748 × 10^−7^
Sn	59/94 (62.8)	0.001026	0.002596	1.466 × 10^−6^	3.709 × 10^−6^	1.434 × 10^−6^–1.499 × 10^−6^

Note: Detection is reported as the number and percentage of fruit samples with concentrations ≥LOD (*n* = 94). Quantitative concentrations used in the dietary-exposure assessment were ≥LOQ; no fruit PTE result between LOD and LOQ was used numerically. Left-censored results were treated using the LB, MB, and UB approach described in Section 2.12.2. Concentrations used for dietary-exposure assessment were converted from dry weight (DW) to wet weight (WW) using the sample-specific dry-matter fraction. The MB scenario was used as the primary exposure estimate. Mean and P95 MB concentrations represent the arithmetic mean and 95th percentile, respectively, of the complete MB concentration distribution. Estimated daily intake was calculated as EDI = (C × IR)/BW, assuming an ingestion rate (IR) of 0.100 kg/day and an adult body weight (BW) of 70 kg. Central and P95 EDI values were based on mean and P95 MB concentrations, respectively, whereas the LB–UB interval represents central EDI estimates obtained under the two censoring bounds. EDI is expressed as mg/kg bw/day. P95 EDI reflects an upper-percentile concentration scenario, not increased consumption, because IR was held constant. For Hg, which was <LOD in all samples, exposure estimates were derived entirely from the LB–MB–UB substitution procedure. Abbreviations: PTEs, potentially toxic elements; EDI, estimated daily intake; LB, lower bound; MB, middle bound; UB, upper bound; P95, 95th percentile; LOD, limit of detection; LOQ, limit of quantification; WW, wet weight; DW, dry weight; DM, dry matter; IR, ingestion rate; BW, body weight.

**Table 12:** Non-carcinogenic risk characterization associated with the consumption of edible *Sambucus nigra* L. fruits under central and upper-percentile exposure scenarios.

Element	RfD (mg/kg bw/day)	Central THQ	P95 THQ	Risk Interpretation
Cd	1.0 × 10^−3^	4.542 × 10^−4^	2.420 × 10^−3^	THQ < 1
Ni	2.0 × 10^−2^	2.394 × 10^−3^	4.964 × 10^−3^	THQ < 1
Sb	4.0 × 10^−4^	6.095 × 10^−4^	3.353 × 10^−3^	THQ < 1
Pb	—	NE	NE	No oral RfD adopted
As	—	NE	NE	Total As; no speciation
Hg	—	NE	NE	All observations <LOD; species not determined
Cr	—	NE	NE	Total Cr; no Cr(III)/Cr(VI) speciation
Sn	—	NE	NE	No directly applicable benchmark adopted
Cumulative HI *	—	0.00346	0.01074	HI < 1

Note: RfD = oral reference dose; THQ = target hazard quotient; HI = hazard index; NE = not evaluated quantitatively; LOD = limit of detection; P95 = 95th percentile; PTEs = potentially toxic elements. THQ was calculated as EDI/RfD using the central and P95 EDI values reported in Table 11. The adopted U.S. EPA IRIS chronic oral RfDs were 1.0 × 10^−3^ for Cd in food, 2.0 × 10^−2^ for soluble nickel salts, and 4.0 × 10^−4^ for Sb (all in mg/kg bw/day) [54,55,56]. THQ < 1 and HI < 1 were interpreted as indicating no appreciable non-carcinogenic concern under the adopted screening assumptions. * The cumulative HI was calculated only from the THQs for Cd, Ni, and Sb. Pb was not assigned an oral RfD within the adopted quantitative framework; As and Cr were measured as total elemental concentrations and were not interpreted using species-specific toxicity values; Hg was below the analytical LOD in all fruit samples and mercury speciation was not determined; and no directly applicable oral benchmark was adopted for Sn. Consequently, the reported HI represents a partial cumulative screening index for Cd, Ni, and Sb and should not be interpreted as a quantitative cumulative risk estimate for all eight investigated PTEs.

**Table 13:** Spatial variation in non-carcinogenic dietary risk associated with the consumption of edible *Sambucus nigra* L. fruits across the five ecological sectors.

Sector	*n*	Cd THQ	Ni THQ	Sb THQ	Cumulative HI	Dominant Contributor (% HI)	Risk Interpretation
Z1	13	2.478 × 10^−5^	1.220 × 10^−3^	9.293 × 10^−5^	1.337 × 10^−3^	Ni (91.2%)	HI < 1
Z2	24	1.574 × 10^−3^	4.131 × 10^−3^	2.099 × 10^−3^	7.803 × 10^−3^	Ni (52.9%)	HI < 1
Z3	20	1.724 × 10^−4^	2.207 × 10^−3^	9.336 × 10^−5^	2.473 × 10^−3^	Ni (89.3%)	HI < 1
Z4	18	2.497 × 10^−5^	7.875 × 10^−4^	9.365 × 10^−5^	9.062 × 10^−4^	Ni (86.9%)	HI < 1
Z5	19	2.520 × 10^−5^	2.733 × 10^−3^	9.451 × 10^−5^	2.853 × 10^−3^	Ni (95.8%)	HI < 1

Note: THQ = target hazard quotient; HI = hazard index; MB = middle bound. Sector-specific THQs were calculated from the mean MB concentration within each sector using EDI = (C × IR)/BW and THQ = EDI/RfD, with IR = 0.100 kg/day and BW = 70 kg. U.S. EPA IRIS chronic oral RfDs of 1.0 × 10^−3^ mg/kg bw/day for Cd, 2.0 × 10^−2^ mg/kg bw/day for Ni, and 4.0 × 10^−4^ mg/kg bw/day for Sb were applied [54,55,56]. The cumulative HI was calculated from the THQs for Cd, Ni, and Sb only. Z1 = eastern periurban sector; Z2 = central urban sector; Z3 = western transitional sector; Z4 = woodland-associated sector; Z5 = southern/southwestern periurban sector. Sector-specific P95 estimates were not calculated because the subgroup sizes (*n* = 13–24) were considered insufficient for stable upper-percentile estimation.

## Data Availability

The original contributions presented in this study are included in the article/Supplementary Material. Further inquiries can be directed to the corresponding authors.

## Supplementary Materials

The following supporting information can be downloaded at https://www.mdpi.com/article/10.3390/foods15193573/s1: Table S1: Distribution of sampling locations and collected samples by biological and environmental matrix within the *Sambucus nigra* monitoring network; Table S2: Ecological and anthropogenic characterization of the *Sambucus nigra* sampling sectors in the Oradea metropolitan area, Romania; Table S3: Zone-Specific Field-Sampling Considerations for Soil Samples Collected within the *Sambucus nigra* Monitoring Network; Table S4: Technical field-sampling metadata for soil and biological matrices included in the *Sambucus nigra* monitoring network; Table S5: Matrix-specific laboratory preparation workflow for *Sambucus nigra* plant samples prior to analysis; Table S6: Matrix-specific closed-vessel microwave-assisted acid digestion conditions applied to soil and *Sambucus nigra* plant samples prior to ICP-MS analysis; Table S7: ICP-MS instrumental configuration, operating conditions, acquisition parameters, and monitored isotopes used for the multi-element analysis of soil and *Sambucus nigra* plant matrices; Table S8: Comprehensive ICP-MS analytical validation, calibration performance, sensitivity parameters, precision, uncertainty estimation, and quality-assurance criteria applied to the multi-element determination of soil and *Sambucus nigra* plant matrices; Table S9: Assessment of the trueness and precision of the ICP-MS method using certified reference materials and second-source certified standard solutions for soil and *Sambucus nigra* plant-tissue matrices; Table S10: Physicochemical characteristics of soil samples collected across the five ecological sectors of the Oradea metropolitan area; Table S11: Individual concentrations of soil macroelements measured in georeferenced soil samples collected across the investigated ecological sectors of the Oradea metropolitan area, Romania; Table S12: Individual concentrations of environmentally associated microelements and biologically relevant trace elements measured in georeferenced soil samples collected across the investigated ecological sectors of the Oradea metropolitan area, Romania; Table S13: Individual concentrations of lithogenic and geochemically associated elements in georeferenced soil samples collected across the ecological sectors of the Oradea metropolitan area, Romania; Table S14: Individual concentrations of potentially toxic and environmentally relevant trace elements measured in georeferenced soil samples collected across the investigated ecological sectors of the Oradea metropolitan area, Romania; Table S15: Individual concentrations of macroelements in *Sambucus nigra* L. bark samples collected from georeferenced sampling points across the investigated ecological sectors of the Oradea metropolitan area, Romania; Table S16: Individual concentrations of environmentally associated microelements and biologically relevant trace elements measured in georeferenced bark samples of *Sambucus nigra* L. collected across the investigated ecological sectors of the Oradea metropolitan area, Romania; Table S17: Individual concentrations of lithogenic and geochemically associated elements in *Sambucus nigra* L. bark samples collected at georeferenced sampling points across the ecological sectors of the Oradea metropolitan area, Romania; Table S18: Individual concentrations of potentially toxic and environmentally relevant trace elements in *Sambucus nigra* L. bark samples collected at georeferenced sampling points across the ecological sectors of the Oradea metropolitan area, Romania; Table S19: Individual concentrations of macroelements in *Sambucus nigra* L. leaf samples collected from georeferenced sampling points across the investigated ecological sectors of the Oradea metropolitan area, Romania; Table S20: Individual concentrations of environmentally associated microelements and biologically relevant trace elements measured in georeferenced leaf samples of *Sambucus nigra* L. collected across the investigated ecological sectors of the Oradea metropolitan area, Romania; Table S21: Individual concentrations of lithogenic and geochemically associated elements in *Sambucus nigra* L. leaf samples collected at georeferenced sampling points across the ecological sectors of the Oradea metropolitan area, Romania; Table S22: Individual concentrations of potentially toxic and environmentally relevant trace elements measured in georeferenced *Sambucus nigra* leaf samples collected across the investigated ecological sectors of the Oradea metropolitan area, Romania; Table S23: Individual concentrations of macroelements in *Sambucus nigra* L. flower samples collected from georeferenced sampling points across the investigated ecological sectors of the Oradea metropolitan area, Romania; Table S24: Individual concentrations of environmentally associated microelements and biologically relevant trace elements measured in georeferenced flower samples of *Sambucus nigra* L. collected across the investigated ecological sectors of the Oradea metropolitan area, Romania; Table S25: Individual concentrations of lithogenic and geochemically associated elements in *Sambucus nigra* L. flower samples collected at georeferenced sampling points across the ecological sectors of the Oradea metropolitan area, Romania; Table S26: Individual concentrations of potentially toxic and environmentally relevant trace elements measured in georeferenced *Sambucus nigra* L. flower samples collected across the investigated ecological sectors of the Oradea metropolitan area, Romania; Table S27: Individual concentrations of macroelements in *Sambucus nigra* L. fruit samples collected from georeferenced sampling points across the investigated ecological sectors of the Oradea metropolitan area, Romania; Table S28: Individual concentrations of environmentally associated microelements and biologically relevant trace elements measured in georeferenced *Sambucus nigra* L. fruit samples collected across the investigated ecological sectors of the Oradea metropolitan area, Romania; Table S29: Individual concentrations of lithogenic and geochemically associated elements in *Sambucus nigra* L. fruit samples collected at georeferenced sampling points across the ecological sectors of the Oradea metropolitan area, Romania; Table S30: Individual concentrations of potentially toxic and environmentally relevant trace elements measured in georeferenced *Sambucus nigra* L. fruit samples collected across the investigated ecological sectors of the Oradea metropolitan area, Romania; Table S31: Detection frequency and descriptive distribution of potentially toxic element concentrations in edible fruits; Table S32: Rank-based spatial differentiation of potentially toxic elements in edible fruits with <LOD observations retained as tied lowest ranks; Table S33: Robustness assessment of sector effects for Cr and Ni concentrations in edible fruits; Table S34: Pairwise co-detection associations among potentially toxic elements in edible fruits; Table S35: Sample-specific fresh- and dry-mass characteristics, dry-matter content, moisture content, and wet-weight concentrations of macroelements and biologically relevant micro/trace elements in edible *Sambucus nigra* L. fruits; Table S36: Sample-specific fresh- and dry-mass characteristics, dry-matter content, moisture content, and wet-weight concentrations of lithogenic/geochemically associated and potentially toxic elements in edible *Sambucus nigra* L. fruits; Table S37: Regulatory reference values, soil quality thresholds, and legally established maximum levels relevant to the investigated elements in soil and *Sambucus nigra* L. matrices; Table S38: Sector-specific middle-bound concentrations and estimated daily intakes of potentially toxic elements in edible *Sambucus nigra* L. fruits across the five ecological sectors; Table S39: Variance structure of the global principal component analysis of elemental profiles across soil and *Sambucus nigra* matrices; Table S40: Centroid scores of the five sample matrices on the first two principal components of the global PCA; Table S41: Complete element loading matrix and relative contributions to PC1 and PC2 obtained from the global PCA of the 470 soil and plant samples; Table S42: Block normalization parameters of the multiple factor analysis of site-paired multi-matrix elemental profiles; Table S43: Global variance structure and explained variance of the multiple factor analysis of site-paired multi-matrix elemental profiles; Table S44: Leading matrix-specific elemental variables contributing to the first two MFA dimensions; Table S45: Integrated hierarchical-clustering structure and inferential validation of matrix- and ecological-zone-specific elemental signatures; Table S46: Cross-matrix comparison of sector-level elemental patterns and Spearman rank associations with the predefined spatial gradient in soil and *Sambucus nigra* organs; Table S47: Visual summary of the dominant cross-matrix spatial elemental signatures observed across the soil–*Sambucus nigra* continuum; Table S48: Complete-case within-site consistency of organ-specific partitioning of investigated elements across *Sambucus nigra* organs.

## Abbreviations

The following abbreviations are used in this manuscript:

ANOVA	Analysis of variance
BW	Body weight
CI	Confidence interval
CV	Coefficient of variation
DM	Dry matter
DTPA	Diethylenetriaminepentaacetic acid
DW	Dry weight
EDI	Estimated daily intake
EFSA	European Food Safety Authority
EU	European Union
FDR	False discovery rate
HCA	Hierarchical cluster analysis
HI	Hazard index
HSD	Honestly significant difference
ICP-MS	Inductively coupled plasma mass spectrometry
IR	Ingestion rate
LB	Lower bound
LOD	Limit of detection
LOQ	Limit of quantification
MB	Middle bound
MFA	Multiple factor analysis
ML	Maximum level
ND	Not determined
P90	90th percentile
P95	95th percentile
PCA	Principal component analysis
PERMANOVA	Permutational multivariate analysis of variance
PERMDISP	Permutational analysis of multivariate dispersions
PR	Prevalence ratio
PTE	Potentially toxic element
Q1	First quartile
Q3	Third quartile
RfD	Reference dose
SD	Standard deviation
TF	Soil-to-organ concentration ratio
THQ	Target hazard quotient
UB	Upper bound
WW	Wet weight
