# Supplementary material for: Multi-Matrix Assessment of Potentially Toxic Elements Across Soil and Sambucus nigra Organs: Spatial Patterns, Regulatory Compliance, and Dietary Exposure of Edible Fruits
Source: Foods. 2026 Oct 8;15(19):3573. doi: 10.3390/foods15193573 (PMC13664746; doi:10.3390/foods15193573)
Supplement: Supplementary file 1 [file foods-15-03573-s001.zip › foods-4596996-supplementary.pdf]

foods

MDPI

Table S1. Distribution of sampling locations and collected samples by biological and environmental matrix within the *Sambucus nigra* monitoring network.

Zone	Soil samples (n)	Bark samples (n)	Leaf samples (n)	Flower samples (n)	Fruit samples (n)	Total samples (n)
Z1 – Eastern periurban sector	13	13	13	13	13	65
Z2 – Central urban sector	24	24	24	24	24	120
Z3 – Western transitional sector	20	20	20	20	20	100
Z4 – Northern and north-eastern woodland-associated sector	18	18	18	18	18	90
Z5 – Southern and south-western periurban sector	19	19	19	19	19	95
TOTAL	94	94	94	94	94	470

Table S2. Ecological and anthropogenic characterization of the *Sambucus nigra* sampling sectors in the Oradea metropolitan area, Romania.

Zone	Habitat Classification	Main environmental and anthropogenic characteristics	Sampling point IDs
Z1	Periurban ecological corridor	The eastern sector of the metropolitan area is characterized by heterogeneous periurban landscapes positioned along the Crișul Repede River corridor. The area includes fragmented riparian vegetation, transitional agricultural interfaces, dispersed residential infrastructure, and semi-natural ecological patches. The environmental mosaic reflects dynamic interactions between anthropogenic activities and natural fluvial ecosystems.	22, 23, 26, 27, 29, 53, 61, 62, 63, 81, 82, 83, 84
Z2	Urban ecosystem	This sector represents the most intensively urbanized component of the study area. Sampling sites are distributed along highly anthropogenized environments characterized by dense infrastructure, major transportation corridors, extensive artificial surfaces, fragmented urban vegetation, and continuous human activity adjacent to the Crișul Repede River.	3, 4, 14, 18, 19, 20, 48, 49, 50, 51, 52, 54, 55, 56, 57, 59, 64, 65, 69, 85, 86, 87, 88, 94
Z3	Transitional ecological sector	The western sector includes landscapes situated at the interface between urbanized environments and semi-natural ecological systems. The area comprises transportation infrastructure, partially industrialized sectors, fragmented riparian habitats, open vegetation patches, and transitional ecological mosaics influenced by both urban and peripheral environmental processes.	1, 5, 6, 7, 8, 32, 33, 34, 35, 36, 37, 38, 39, 40, 41, 58, 60, 89, 90, 91

Z4	Transitional woodland ecosystem	This ecological sector is situated within the hilly northern and northeastern surroundings of Oradea and includes semi-natural woodland environments, forest-edge vegetation, lower infrastructural density, and increased ecological continuity relative to the urban sectors. The area is characterized by heterogeneous topography and reduced direct urban disturbance.	2, 9, 10, 11, 12, 13, 15, 16, 17, 21, 24, 25, 28, 30, 31, 70, 71, 72
Z5	Periurban–rural transitional ecosystem	This sector encompasses several periurban and rural-transition localities located within the southern and southwestern margins of the Oradea metropolitan area, including Sântandrei, Leș, Nojorid, Livada de Bihor, Palota, Cheriu, and Hidișelul de Jos. The landscape consists of mixed agricultural areas, periurban settlements, open herbaceous vegetation, and fragmented semi-natural habitats.	42, 43, 44, 45, 46, 47, 66, 67, 68, 73, 74, 75, 76, 77, 78, 79, 80, 92, 93

Table S3. Zone-Specific Field-Sampling Considerations for Soil Samples Collected within the *Sambucus nigra* Monitoring Network.

Zone	Zone-specific sampling context	Exclusion/removal criteria
Z1 – Eastern periurban sector	Periurban and riparian microsites located around the sampled *S. nigra* individuals	Litter, plant residues, stones, coarse debris, visible foreign materials, and locally disturbed microsites
Z2 – Central urban sector	Urban plant-associated microsites located around the sampled shrubs	Surface litter, plant fragments, stones, construction debris, visible deposits, and highly disturbed or compacted microsites
Z3 – Western transitional sector	Transitional microsites located at the interface between urbanized and semi-natural environments	Litter, superficial roots, stones, coarse fragments, visible foreign materials, and recently disturbed microsites
Z4 – Northern and northeastern woodland-associated sector	Semi-natural woodland and forest-edge microsites corresponding to the sampled shrubs	Leaf litter, woody debris, stones, coarse roots, visible organic residues, and non-soil foreign materials
Z5 – Southern and southwestern periurban sector	Periurban–rural microsites located around the sampled shrubs	Plant and agricultural residues, stones, coarse debris, visible foreign materials, and disturbed microsites

Note: At each location, one composite soil sample was obtained from 3–5 subsamples collected at a depth of 0–20 cm within approximately 1–2 m of the corresponding *Sambucus nigra* individual. Approximately 500–1000 g of field-moist soil was collected using a pre-cleaned stainless-steel soil auger and transferred to a pre-labeled polyethylene bag. Sampling tools were decontaminated between locations using deionized water and analytical-grade ethanol.

Table S4. Technical field-sampling metadata for soil and biological matrices included in the *Sambucus nigra* monitoring network.

Matrix	Sampling period / phenological stage	Sampling position	Sampling strategy	Target fresh mass per site	Matrix-specific inclusion criteria	Matrix-specific exclusion criteria	Field tools
Soil	Visit 1: 18–31 May 2026; collected	Topsoil (0–20 cm) within approximately	Composite sample obtained from	500–1000 g	Representative plant-	Recently disturbed soil; waste	Pre-cleaned stainless-

	once per site during active vegetation, concurrently with bark and leaf sampling	1–2 m of the plant base	3–5 subsamples collected around the selected plant		associated root-zone topsoil	deposits; visible chemical spills; excessive plant debris	steel soil auger
Bark	Visit 1: 18–31 May 2026; active vegetation period	External bark layer at 1.0–1.5 m above ground level	Bark fragments collected from several stem orientations, where possible	20–50 g	Mature individuals with intact, visibly healthy bark	Necrosis; fungal infection; severe mechanical injury; abnormal bark degradation; mosses; lichens; bird droppings; visible surface deposits	Stainless-steel scalpel
Leaf	Visit 1: 18–31 May 2026; active vegetation period; fully expanded mature leaves	Median canopy layer	Leaves collected from several canopy orientations, where possible	100–200 g	Fully expanded, mature, visibly healthy leaves	Young; senescent; chlorotic; mechanically damaged; pathogen-affected; visibly contaminated leaves	Stainless-steel scissors
Flower	Visit 2: 1–20 June 2026; full flowering stage	Median or upper canopy positions	Inflorescences collected from several canopy orientations, where possible	100–200 g	Fully developed, visibly healthy inflorescences	Senescent; dehydrated; mechanically damaged; pathogen-affected; visibly contaminated inflorescences	Stainless-steel scissors
Fruit	Visit 3: 10 August–5 September 2026; full ripening stage	Median or upper canopy positions	Fruit clusters collected from several canopy orientations, where possible	100–200 g	Fully developed, physiologically mature, visibly healthy berries	Immature; overripe; dehydrated; mechanically damaged; bird-damaged; pathogen-affected; visibly contaminated berries	Stainless-steel scissors

Note: Field sampling was conducted during the 2026 growing season through three phenology-specific visits to the same network of 94 georeferenced sampling sites, each represented by the same selected *Sambucus nigra* L. individual. Soil, bark, and leaves were collected during Visit 1 (18–31 May 2026), flowers during Visit 2 at full flowering (1–20 June 2026), and ripe fruits during Visit 3 at full ripening (10 August–5 September 2026). Each matrix was collected once per site, and the exact sampling date within each interval

was adjusted according to the phenological development of the selected individual. Consequently, all environmental and biological matrices were site-paired through the same georeferenced plant-associated sampling unit but were not temporally paired across all matrices. Immediately after collection, samples were placed in sealed, prelabeled polyethylene bags, protected from direct sunlight and potential cross-contamination, and handled using powder-free nitrile gloves. Reusable sampling tools were cleaned between sampling sites using ultrapure water produced with a Milli-Q Direct 8 water purification system (Merck KGaA, Darmstadt, Germany), followed by analytical-grade ethanol. Samples were transported to the laboratory on the same day, generally within approximately 1–3 h of collection. Fruit samples were maintained under refrigerated conditions at approximately 4–8 °C during transport. Each sample received a unique identification code linked to the ecological sector, matrix type, sampling-point ID, GPS coordinates, and collection date.

Table S5. Matrix-specific laboratory preparation workflow for *Sambucus nigra* plant samples prior to analysis

Biological matrix	Initial laboratory sorting/cleaning	Rinsing step	Pre-drying treatment	Drying and weighing	Homogenization/grinding	Final storage
Bark	Removal of adhering dust, plant debris, degraded tissue fragments, and foreign particles	Not washed, to avoid removal or redistribution of surface-associated elements	No cutting required unless large fragments were present	Dried to constant weight at 40 ± 2 °C, cooled in a silica-gel desiccator, and weighed	Ground to a fine homogeneous powder using non-contaminating grinding equipment	Stored in pre-labeled polyethylene containers under dry conditions
Leaves	Removal of damaged, chlorotic, senescent, or visibly contaminated tissues	Gently rinsed with Milli-Q water and blotted dry	Whole or fragmented leaves processed after blotting	Dried to constant weight at 40 ± 2 °C, cooled in a silica-gel desiccator, and weighed	Homogenized and ground to a fine powder	Stored in pre-labeled polyethylene containers under dry conditions
Flowers	Removal of senescent, discolored, damaged, or visibly contaminated floral parts	Gently rinsed with Milli-Q water and blotted dry	Inflorescences spread in a thin layer on polyethylene trays	Dried to constant weight at 40 ± 2 °C, cooled in a silica-gel desiccator, and weighed	Carefully homogenized due to fragile floral structure until a uniform powder was obtained	Stored in pre-labeled polyethylene containers under dry conditions
Fruits	Removal of immature, dehydrated, mechanically damaged, bird-damaged, or deteriorated berries; stalks removed	Rinsed with Milli-Q water and blotted dry	Fruits gently cut or crushed to facilitate water removal	Dried to constant weight at 40 ± 2 °C, cooled in a silica-gel desiccator, and weighed	Ground to a fine homogeneous powder using non-contaminating grinding equipment	Stored in pre-labeled polyethylene containers under dry conditions

2.7. Physicochemical Characterization of Soil Samples

Physicochemical analyses were performed using air-dried soil samples homogenized and passed through a 2 mm polyethylene sieve, except for moisture content, dry bulk density, and saturated hydraulic conductivity, which were determined using field-moist or undisturbed soil samples. Sample masses were recorded using an AS 60/220.R2 PLUS analytical balance (RADWAG Balances and Scales, Radom, Poland), and ultrapure water was produced using a Milli-Q Direct 8 water purification system (Merck KGaA, Darmstadt, Germany).

Soil pH and electrical conductivity of the 1:5 soil-to-water extract ($EC_{1:5}$) were determined in separate 1:5 (w/v) soil-to-water suspensions. Air-dried soil (10.00 g) was mixed with 50 mL of ultrapure water, agitated for 60 min at 180 rpm using a KS 260 control orbital shaker (IKA-Werke GmbH & Co. KG, Staufen, Germany), and allowed to equilibrate for 30 min. Measurements were performed using an Orion Versa Star Pro VSTAR52 multiparameter meter (Thermo Fisher Scientific, Waltham, MA, USA), equipped with an 8157BNUMD ROSS Ultra Triode pH electrode and a 013005MD DuraProbe conductivity sensor.

Before each daily analytical series, the pH electrode was calibrated using certified buffer solutions at pH 4.01, 7.00, and 10.01. Calibration was accepted when the electrode slope was within 95–105% of the theoretical response and the zero-point offset was within ±30 mV. If either acceptance criterion was not met, the electrode was conditioned and recalibrated before sample measurement.

Before each daily analytical series, the conductivity sensor was calibrated using a certified 1413 μS cm^{-1} standard. Calibration was independently verified using a second-source certified standard of 500 μS cm^{-1}, which was not used for calibration. Verification was accepted when the measured value was within ±5% of the certified value. If this criterion was not met, the sensor was recalibrated and the verification was repeated before sample analysis. The same calibration and verification procedure was applied to both $EC_{1:5}$ and saturated-paste extract conductivity (EC_e) measurements. EC values were temperature-corrected to 25 °C. $EC_{1:5}$ values were expressed as μS cm^{-1}, whereas EC_e values were expressed as dS m^{-1}. The procedures followed the analytical principles of ISO 10390:2021 and ISO 11265:2025.

Soil salinity was characterized using the electrical conductivity of the saturated-paste extract (EC_e). Ultrapure water was gradually added to approximately 100 g of air-dried soil until a homogeneous saturated paste was obtained. After overnight equilibration, the saturation extract was recovered by vacuum filtration and analyzed using the conductivity module of the Orion Versa Star Pro VSTAR52 meter.

Moisture content was determined gravimetrically according to ISO 11465:2025. Approximately 10.00 g of field-moist soil was dried at 105 ± 2 °C to constant mass in a UF55 forced-air laboratory oven (Memmert GmbH + Co. KG, Schwabach, Germany), cooled in a silica-gel desiccator, and reweighed. Moisture content was expressed as the percentage of mass lost relative to the initial field-moist soil mass.

Soil organic matter (OM) was determined by loss on ignition. Approximately 5.00 g of soil previously dried at 105 °C was transferred into pre-ignited and preweighed porcelain crucibles and heated at 550 ± 25 °C for 4 h in an L 9/11/SKM programmable muffle furnace (Nabertherm GmbH, Lilienthal, Germany). After cooling in a silica-gel desiccator, the crucibles were reweighed, and OM was expressed as the percentage of mass lost relative to the oven-dry soil mass.

Estimated soil organic carbon (SOC_{est}) was calculated from the loss-on-ignition-derived OM content as OM/1.724 using the conventional Van Bemmelen conversion factor.

Calcium carbonate content was determined volumetrically according to the Scheibler principle using a Calcimeter 08.53 (Royal Eijkelkamp, Giesbeek, The Netherlands). Approximately 1.00 g of air-dried soil was reacted with 10 mL of 4 mol L^{-1} hydrochloric acid in a sealed calcimeter vessel. The volume of released CO_2 was corrected for ambient temperature and atmospheric pressure, and the result was expressed as $CaCO_3$ equivalent on a dry-weight basis. The procedure was based on the analytical principle of ISO 10693:1995.

Total nitrogen (TN) was determined by dry combustion using a vario MAX cube elemental analyzer operated in CN mode (Elementar Analysensysteme GmbH, Langenselbold, Germany). Approximately 300–500 mg of finely ground soil was weighed into combustion crucibles and analyzed in an oxygen-rich atmosphere. Nitrogen-containing combustion products were reduced to molecular nitrogen and quantified using a thermal conductivity detector. Calibration was performed using certified acetanilide standards, and TN was expressed as a percentage of dry soil mass. The procedure was based on the analytical principle of ISO 13878:1998.

Particle-size distribution was determined by laser diffraction using a Mastersizer 3000 particle-size analyzer coupled to a Hydro EV wet-dispersion unit (Malvern Panalytical Ltd., Malvern, United Kingdom). Approximately 2.00 g of soil was treated stepwise with 30% hydrogen peroxide to remove organic matter and subsequently dispersed in a 5% sodium hexametaphosphate solution. After overnight agitation, the suspension was introduced into the wet-dispersion unit and analyzed after stable dispersion and obscuration had been achieved. Particle-size fractions were classified as clay (<2 μm), silt (2–50 μm), and sand (50–2000 μm) and reported as volume-based percentages. Three consecutive instrumental readings were recorded for each independently prepared dispersion.

Potential cation exchange capacity (CEC_{pot}) was determined using 1 mol L^{-1} ammonium acetate buffered at pH 7.00. Air-dried soil (5.00 g) was treated with 50 mL of ammonium acetate for 60 min and filtered. The ammonium-saturated soil residue retained on the filter was washed with ethanol to remove excess ammonium acetate. The adsorbed ammonium ions were subsequently displaced using 1 mol L^{-1} potassium chloride and quantified spectrophotometrically at 660 nm using a UV-1900i Plus UV–Vis spectrophotometer (Shimadzu Corporation, Kyoto, Japan). CEC_{pot} was expressed as cmol(+) kg^{-1} dry soil. The procedure was based on the analytical principle of ISO/TS 22171:2023.

Ammonium acetate-extractable potassium was determined in the initial ammonium acetate extract and interpreted as exchangeable potassium. Potassium concentrations were quantified using an Agilent 5800 Vertical Dual View ICP-OES instrument (Agilent Technologies, Santa Clara, CA, USA) after external calibration with matrix-matched standards. Results were expressed as mg kg^{-1} dry soil.

Olsen-extractable phosphorus was determined by extracting 2.50 g of air-dried soil with 50 mL of 0.5 mol L^{-1} sodium hydrogen carbonate adjusted to pH 8.5. Samples were agitated for 30 min at 180 rpm and filtered. Phosphorus was determined using the molybdenum-blue spectrophotometric method at 880 nm with the UV-1900i Plus spectrophotometer and expressed as mg kg^{-1} dry soil. The procedure was based on the analytical principle of ISO 11263:1994.

DTPA-extractable Fe, Mn, Zn, and Cu were determined using an extractant containing 0.005 mol L^{-1} diethylenetriaminepentaacetic acid, 0.1 mol L^{-1} triethanolamine, and 0.01 mol L^{-1} $CaCl_2$, adjusted to pH 7.30. Air-dried soil (10.00 g) was mixed with 20 mL of extractant and agitated for 2 h at room temperature. Extracts were filtered through 0.45 μm membrane filters and analyzed using the Agilent 5800 ICP-OES instrument. ΣDTPA was defined as the sum of the DTPA-extractable Fe, Mn, Zn, and Cu concentrations and expressed as mg kg^{-1} dry soil. The extraction conditions were based on the Lindsay–Norvell DTPA soil-test procedure.

Dry bulk density and saturated hydraulic conductivity were determined using three independent undisturbed soil cores collected from each sampling site in stainless-steel rings of known internal volume. Saturated hydraulic conductivity was determined first on each intact core using a Soil Water Permeameter (Royal Eijkelkamp, Giesbeek, The Netherlands). Soil cores were slowly saturated from the base with deaerated water for 24 h and analyzed under a constant hydraulic head. Saturated hydraulic conductivity was calculated according to Darcy's law and expressed as cm h^{-1}.

Following the hydraulic-conductivity measurements, the same soil cores were dried at 105 ± 2 °C to constant mass. Dry bulk density was determined from the oven-dry soil mass and the known internal volume of the sampling ring using the core method described in ISO 11272:2017 and expressed as g cm^{-3}.

Chemical determinations were performed in triplicate using independently prepared analytical portions for each sampling site. Particle-size distribution was determined using independently prepared dispersions, with three consecutive instrumental readings recorded for each dispersion. Dry bulk density and saturated hydraulic conductivity were determined using three independent undisturbed soil cores per sampling site. Method-specific reagent blanks, calibration standards, calibration-verification solutions, duplicate quality-control samples, and laboratory-control soil samples were included where applicable.

Replicate measurements were averaged to obtain a single site-level value for each parameter before sector-level statistical analysis. Sector-level results were expressed as mean ± standard deviation, with n representing the number of sampling sites within each ecological sector. Unless otherwise specified, concentration-based results were reported on a dry-weight basis.

Table S6. Matrix-specific closed-vessel microwave-assisted acid digestion conditions applied to soil and *Sambucus nigra* plant samples prior to ICP-MS analysis

Digestion parameter	Soil samples	Plant samples: bark, leaves, flowers, and fruits
Analytical portion	0.5000 g	0.3000 g
Nitric acid addition	6.0 mL of 65% HNO_3, trace-metal grade	6.0 mL of 65% HNO_3, trace-metal grade
Hydrogen peroxide addition	2.0 mL of 30% H_2O_2, trace-analysis grade	2.0 mL of 30% H_2O_2, trace-analysis grade
Microwave program	25–120 °C over 10 min (max. 500 W); 120–180 °C over 15 min (max. 1000 W); hold at 180 °C for 20 min (max. 1000 W)	Same program
Final digest volume	50.0 mL	25.0 mL
Procedural digestion blanks	Complete reagent mixture without soil; one blank included in each microwave digestion batch	Complete reagent mixture without plant material; one blank included in each microwave digestion batch
Independent digestion replicates	Three independently weighed and digested analytical portions per sampling site	Three independently weighed and digested analytical portions per biological matrix and sampling site
Re-digestion criterion	Samples containing visible residues or producing unacceptable recoveries were subjected to a new digestion	Samples containing visible residues or producing unacceptable recoveries were subjected to a new digestion
Digest storage before analysis	Stored in acid-cleaned, tightly closed polypropylene tubes at 4 °C until ICP-MS determination	Stored in acid-cleaned, tightly closed polypropylene tubes at 4 °C until ICP-MS determination

Elemental fraction represented	Operationally defined acid-recoverable or pseudo-total elemental fraction	Acid-digestible elemental fraction

Note: HNO_3 = nitric acid; H_2O_2 = hydrogen peroxide; ICP-MS = inductively coupled plasma mass spectrometry. Bark, leaf, flower, and fruit samples were grouped because the same microwave-assisted digestion procedure was applied to all plant matrices. Procedural blanks underwent the complete digestion procedure. The three independently prepared analytical portions were used to assess analytical precision and were averaged to obtain one concentration per matrix and sampling site for subsequent site-level analyses. Because hydrofluoric acid was not included in the soil digestion mixture, soil concentrations represent an operationally defined acid-recoverable or pseudo-total fraction rather than complete dissolution of silicate-bound elements.

Table S7. ICP-MS instrumental configuration, operating conditions, acquisition parameters, and monitored isotopes used for the multi-element analysis of soil and *Sambucus nigra* plant matrices.

Instrumental parameter	Operating condition
ICP-MS instrument	iCAP Q quadrupole inductively coupled plasma mass spectrometer (Thermo Fisher Scientific, Bremen, Germany)
Autosampler	ASX-520 autosampler (Teledyne CETAC Technologies, Omaha, NE, USA)
Sample delivery system	Peristaltic pump connected to a micro-concentric nebulizer
Nebulizer	Micro-concentric MicroMist nebulizer, nominal uptake rate 0.4 mL min^{-1}
Spray chamber	Temperature-controlled cyclonic spray chamber
Spray-chamber temperature	2.1 °C
Torch	Standard quartz ICP torch
Injector	Quartz injector tube, 1.5 mm internal diameter
Sampler cone	Nickel sampler cone
Skimmer cone	Nickel skimmer cone with a 0.5 mm insert
Radiofrequency power	1550 W
Plasma gas	Argon 5.0, purity ≥99.999%
Plasma gas flow rate	14.0 L min^{-1}
Auxiliary gas	Argon 5.0
Auxiliary gas flow rate	Automatically optimized during daily instrument tuning
Nebulizer gas	Argon 5.0
Nebulizer gas flow rate	Automatically optimized during daily instrument tuning to obtain maximum ion sensitivity and stable signal intensity
Collision gas	Helium 6.0, purity ≥99.9999%
Helium flow rate	4.5 mL min^{-1}
Acquisition mode	Quantitative multi-element acquisition
Collision-cell mode	Helium kinetic energy discrimination mode (He-KED)
Interference-control procedure	Polyatomic and matrix-related spectral interferences were reduced using helium collision gas and kinetic energy discrimination
Instrument equilibration	At least 45 min before tuning and sample analysis
Tuning solution	TUNE B iCAP Q multielement tuning solution containing Ba, Bi, Ce, Co, In, Li, and U at 1.0 μg L^{-1} in 2% HNO_3 and 0.5% HCl
Instrument tuning	Performed before each analytical sequence to maximize singly charged ion sensitivity and ensure stable instrumental response
Oxide-formation criterion	CeO^+/Ce^+ ratio maintained below 2%
Doubly charged-ion criterion	Ba^{2+}/Ba^+ ratio maintained below 2%
Points per peak	3
Scans per acquisition replicate	7
Dwell time	3 ms per monitored mass

Integration time	1–5 ms, depending on the monitored isotope and expected signal intensity
Background correction	Two background-correction points per monitored mass
Sample uptake time	90 s
Rinsing procedure	The sample-introduction system was rinsed between measurements with dilute nitric acid until the signal returned to the instrumental background level
Data-acquisition software	Qtegra™ Intelligent Scientific Data Solution software (Thermo Fisher Scientific)
Macroelements determined	K, Ca, Mg, Na, P, and S
Environmentally associated microelements and biologically relevant trace elements determined	Fe, Mn, Zn, Cu, B, Mo, Co, and Se
Lithogenic and geochemically associated elements determined	Al, Ti, V, Ba, Sr, Li, Rb, and Cs
Potentially toxic and environmentally relevant trace elements determined	Pb, Cd, As, Hg, Cr, Ni, Sb, and Sn
Potassium isotope	^{39}K
Calcium isotope	^{44}Ca
Magnesium isotope	^{24}Mg
Sodium isotope	^{23}Na
Phosphorus isotope	^{31}P
Sulfur isotope	^{34}S
Iron isotope	^{56}Fe
Manganese isotope	^{55}Mn
Zinc isotope	^{64}Zn
Copper isotope	^{63}Cu
Boron isotope	^{11}B
Molybdenum isotope	^{98}Mo
Cobalt isotope	^{59}Co
Selenium isotope	^{80}Se
Aluminium isotope	^{27}Al
Titanium isotope	^{48}Ti
Vanadium isotope	^{51}V
Barium isotope	^{137}Ba
Strontium isotope	^{88}Sr
Lithium isotope	^{7}Li
Rubidium isotope	^{85}Rb
Caesium isotope	^{133}Cs
Lead isotope	^{208}Pb
Cadmium isotope	^{111}Cd
Arsenic isotope	^{75}As
Mercury isotope	^{202}Hg
Chromium isotope	^{52}Cr
Nickel isotope	^{60}Ni
Antimony isotope	^{121}Sb
Tin isotope	^{118}Sn
Sample solution introduced into the instrument	Digested soil and plant samples appropriately diluted with ultrapure water in a nitric-acid matrix

Concentration calculation	Elemental concentrations were calculated by accounting for the initial dry sample mass, final digestion volume, and all subsequent dilution factors
Reporting basis	Dry-weight basis for soil, bark, leaf, flower, and fruit matrices
Reporting units	mg kg^{-1} dry weight; concentrations may be converted to μg kg^{-1} for elements present at very low levels

Table S8. Comprehensive ICP-MS analytical validation, calibration performance, sensitivity parameters, precision, uncertainty estimation, and quality-assurance criteria applied to the multi-element determination of soil and *Sambucus nigra* plant matrices.

Element	Isotope monitored	Calibration range	Calibration points	R^2	Slope	LOD	LOQ	Recovery (%)	Intra-day RSD (%)	Inter-day RSD (%)	Expanded uncertainty (%)	Instrument drift (%)	BEC	CRM agreement (%)	Internal standard	Collision mode
K	^{39}K	0.01–50 mg/L	7	0.9988	0.991	0.018 mg/L	0.060 mg/L	93.6	5.2	6.4	7.7	3.2	0.009 mg/L	94.3	Sc	He-KED
Ca	^{44}Ca	0.01–50 mg/L	7	0.9991	0.993	0.021 mg/L	0.070 mg/L	95.1	4.9	6.0	7.1	2.8	0.011 mg/L	95.8	Sc	He-KED
Mg	^{24}Mg	0.005–20 mg/L	6	0.9995	0.997	0.004 mg/L	0.014 mg/L	97.2	3.8	4.9	5.9	2.1	0.003 mg/L	98.1	Sc	He-KED
Na	^{23}Na	0.005–20 mg/L	6	0.9992	0.994	0.008 mg/L	0.027 mg/L	94.8	4.6	5.7	6.8	2.5	0.005 mg/L	95.4	Sc	He-KED
P	^{31}P	0.01–20 mg/L	6	0.9992	0.994	0.009 mg/L	0.030 mg/L	94.1	5.1	6.3	7.6	3.0	0.004 mg/L	94.9	Sc	He-KED
S	^{34}S	0.02–50 mg/L	7	0.9989	0.991	0.021 mg/L	0.070 mg/L	92.7	5.8	7.1	8.5	3.4	0.010 mg/L	93.6	Sc	He-KED
Fe	^{56}Fe	0.1–1000 µg/L	7	0.9990	0.992	0.074 µg/L	0.247 µg/L	92.8	6.9	8.3	9.5	3.9	0.025 µg/L	93.5	Ge	He-KED
Mn	^{55}Mn	0.05–250 µg/L	6	0.9998	0.999	0.006 µg/L	0.020 µg/L	98.3	2.7	3.5	4.4	1.5	0.003 µg/L	99.0	Ge	He-KED
Zn	^{64}Zn	0.1–1000 µg/L	7	0.9993	0.995	0.052 µg/L	0.173 µg/L	94.2	7.3	8.7	10.2	4.1	0.018 µg/L	95.0	Rh	He-KED
Cu	^{63}Cu	0.05–500 µg/L	6	0.9997	0.998	0.009 µg/L	0.030 µg/L	97.6	3.5	4.6	5.5	2.0	0.003 µg/L	98.2	Rh	He-KED
B	^{11}B	0.05–500 µg/L	6	0.9994	0.996	0.015 µg/L	0.050 µg/L	95.3	4.9	6.1	7.2	2.7	0.006 µg/L	96.0	Sc	He-KED
Mo	^{98}Mo	0.01–100 µg/L	6	0.9998	0.999	0.003 µg/L	0.010 µg/L	98.2	2.9	3.8	4.7	1.6	0.001 µg/L	98.8	Rh	He-KED
Co	^{59}Co	0.005–50 µg/L	6	0.9997	0.998	0.003 µg/L	0.010 µg/L	97.3	3.7	4.6	5.4	2.0	0.001 µg/L	98.1	Ge	He-KED
Se	^{80}Se	0.01–100 µg/L	6	0.9993	0.995	0.012 µg/L	0.040 µg/L	94.6	5.9	7.1	8.3	3.1	0.005 µg/L	95.2	Ge	He-KED
Al	^{27}Al	0.1–1000 µg/L	7	0.9991	0.993	0.061 µg/L	0.203 µg/L	93.4	6.8	8.1	9.6	3.8	0.021 µg/L	94.2	Ge	He-KED
Ti	^{48}Ti	0.05–500 µg/L	6	0.9992	0.994	0.022 µg/L	0.073 µg/L	93.1	6.5	7.9	9.2	3.6	0.009 µg/L	94.0	Ge	He-KED
V	^{51}V	0.01–100 µg/L	6	0.9996	0.998	0.006 µg/L	0.020 µg/L	96.7	4.0	5.1	6.0	2.2	0.002 µg/L	97.3	Ge	He-KED
Ba	^{137}Ba	0.01–250 µg/L	6	0.9996	0.998	0.010 µg/L	0.033 µg/L	96.1	4.5	5.4	6.6	2.4	0.004 µg/L	97.0	In	He-KED
Sr	^{88}Sr	0.01–250 µg/L	6	0.9999	1.000	0.002 µg/L	0.007 µg/L	98.7	2.1	2.9	3.6	1.1	0.001 µg/L	99.1	Rh	He-KED
Li	^{7}Li	0.05–100 µg/L	6	0.9997	0.998	0.004 µg/L	0.013 µg/L	96.4	3.1	4.2	5.3	1.8	0.002 µg/L	97.2	Sc	He-KED
Rb	^{85}Rb	0.01–100 µg/L	6	0.9998	0.999	0.003 µg/L	0.010 µg/L	99.1	2.4	3.1	4.0	1.2	0.001 µg/L	99.4	Rh	He-KED
Cs	^{133}Cs	0.005–50 µg/L	6	0.9999	1.000	0.001 µg/L	0.004 µg/L	99.3	2.0	2.8	3.5	1.0	0.0004 µg/L	99.6	In	He-KED
Pb	^{208}Pb	0.005–50 µg/L	6	0.9997	0.999	0.003 µg/L	0.010 µg/L	97.1	3.9	4.8	5.7	2.2	0.001 µg/L	98.0	Bi/In	He-KED
Cd	^{111}Cd	0.005–25 µg/L	6	0.9998	0.999	0.002 µg/L	0.006 µg/L	97.9	3.6	4.4	5.1	1.9	0.001 µg/L	98.5	In	He-KED

As	^{75}As	0.01–50 μg/L	6	0.9995	0.997	0.007 μg/L	0.023 μg/L	95.8	4.8	5.9	7.0	2.7	0.003 μg/L	96.3	Ge	He-KED
Hg	^{202}Hg	0.005–20 μg/L	6	0.9991	0.992	0.004 μg/L	0.013 μg/L	89.4	8.6	9.8	11.5	4.8	0.002 μg/L	90.7	In	He-KED
Cr	^{52}Cr	0.05–100 μg/L	6	0.9994	0.996	0.018 μg/L	0.060 μg/L	91.7	6.4	7.5	8.9	3.4	0.007 μg/L	92.6	Ge	He-KED
Ni	^{60}Ni	0.02–100 μg/L	6	0.9996	0.998	0.011 μg/L	0.037 μg/L	96.9	4.1	5.2	6.1	2.3	0.004 μg/L	97.4	Ge	He-KED
Sb	^{121}Sb	0.005–50 μg/L	6	0.9997	0.999	0.003 μg/L	0.010 μg/L	97.0	3.8	4.9	5.8	2.1	0.001 μg/L	97.8	In	He-KED
Sn	^{118}Sn	0.01–100 μg/L	6	0.9995	0.997	0.007 μg/L	0.023 μg/L	95.9	4.7	5.8	6.9	2.6	0.003 μg/L	96.6	In	He-KED

Note: R^2 = coefficient of determination; LOD = limit of detection; LOQ = limit of quantification; RSD = relative standard deviation; BEC = background equivalent concentration; CRM = certified reference material; He-KED = helium kinetic energy discrimination mode. Ten independently prepared procedural blanks (n = 10), containing all digestion reagents but no sample material, were processed through the complete digestion and ICP-MS workflow. LOD and LOQ were estimated from the procedural-blank results using the 3σ and 10σ criteria, respectively. Intra-day precision was evaluated separately for each matrix using six independently prepared portions of the same homogenized material analyzed within one day (n = 6). Inter-day precision was assessed using three independently prepared portions per day over three analytical days (n = 9 per matrix). Each digest was measured in three instrumental replicates, and their mean was treated as one analytical result. Precision was expressed as %RSD. For each element, the recovery and CRM agreement values reported in the table correspond to the evaluated matrix showing the greatest absolute deviation from 100%, whereas the intra-day RSD, inter-day RSD, and expanded uncertainty values represent the highest values obtained among the evaluated soil and plant matrices. Thus, the reported validation parameters reflect the most conservative analytical performance observed across the investigated matrices.

Table S9. Assessment of the trueness and precision of the ICP-MS method using certified reference materials and second-source certified standard solutions for soil and *Sambucus nigra* plant-tissue matrices.

Element	Isotope	Matrix / validation role	Reference material or verification solution	Certified or target value	Measured value (Mean ± SD, n = 3)	Recovery (%)	Relative error (%)	Interpretation
K	^{39}K	Fruit CRM	NIST SRM 3287 Blueberry (Fruit)	4490 ± 220 mg kg^{-1}	4382 ± 53 mg kg^{-1}	97.6	2.4	Satisfactory agreement
Ca	^{44}Ca	Fruit CRM	NIST SRM 3287 Blueberry (Fruit)	323 ± 16 mg kg^{-1}	313.0 ± 4.7 mg kg^{-1}	96.9	3.1	Satisfactory agreement
Mg	^{24}Mg	Fruit CRM	NIST SRM 3287 Blueberry (Fruit)	313.7 ± 7.2 mg kg^{-1}	307.7 ± 3.4 mg kg^{-1}	98.1	1.9	Excellent agreement
Na	^{23}Na	Food-matrix CRM used as a surrogate for fruit	NIST SRM 1568c Rice Flour	15.9 ± 1.6 mg kg^{-1}	15.22 ± 0.32 mg kg^{-1}	95.7	4.3	Satisfactory agreement
P	^{31}P	Fruit CRM	NIST SRM 3287 Blueberry (Fruit)	671 ± 21 mg kg^{-1}	665.6 ± 8.7 mg kg^{-1}	99.2	0.8	Excellent agreement

S	^{34}S	Food-matrix CRM used as a surrogate for fruit	NIST SRM 1568c Rice Flour	1155.5 ± 8.4 mg kg^{-1}	1125 ± 11 mg kg^{-1}	97.4	2.6	Satisfactory agreement
Fe	^{56}Fe	Fruit CRM	NIST SRM 3287 Blueberry (Fruit)	12.20 ± 0.74 mg kg^{-1}	11.79 ± 0.21 mg kg^{-1}	96.6	3.4	Satisfactory agreement
Mn	^{55}Mn	Fruit CRM	NIST SRM 3287 Blueberry (Fruit)	8.47 ± 0.59 mg kg^{-1}	8.33 ± 0.12 mg kg^{-1}	98.3	1.7	Excellent agreement
Zn	^{64}Zn	Fruit CRM	NIST SRM 3287 Blueberry (Fruit)	6.49 ± 0.61 mg kg^{-1}	6.15 ± 0.15 mg kg^{-1}	94.8	5.2	Acceptable agreement
Cu	^{63}Cu	Fruit CRM	NIST SRM 3287 Blueberry (Fruit)	2.22 ± 0.16 mg kg^{-1}	2.16 ± 0.04 mg kg^{-1}	97.3	2.7	Satisfactory agreement
B	^{11}B	Botanical CRM used as a surrogate for bark, leaves, and flowers	NIST SRM 1573a Tomato Leaves	33.13 ± 0.42 mg kg^{-1}	32.70 ± 0.52 mg kg^{-1}	98.7	1.3	Excellent agreement
Mo	^{98}Mo	Food-matrix CRM used as a surrogate for fruit	NIST SRM 1568c Rice Flour	0.638 ± 0.021 mg kg^{-1}	0.615 ± 0.014 mg kg^{-1}	96.4	3.6	Satisfactory agreement
Co	^{59}Co	Soil CRM comparator (aqua-regia-extractable value)*	ERM-CC141 Loam Soil	7.9 ± 0.9 mg kg^{-1}	7.58 ± 0.19 mg kg^{-1}	95.9	4.1	Indicative only; method equivalence required*
Se	^{80}Se	Food-matrix CRM used as a surrogate for fruit	NIST SRM 1568c Rice Flour	0.0647 ± 0.0023 mg kg^{-1}	0.0605 ± 0.0018 mg kg^{-1}	93.5	6.5	Acceptable agreement
Al	^{27}Al	Botanical CRM used as a surrogate for bark, leaves, and flowers	NIST SRM 1573a Tomato Leaves	598.4 ± 7.1 mg kg^{-1}	563.7 ± 11.8 mg kg^{-1}	94.2	5.8	Acceptable agreement
Ti	^{48}Ti	Second-source calibration verification	Second-source certified standard solution†	10.00 µg L^{-1}	9.78 ± 0.18 µg L^{-1}	97.8	2.2	Satisfactory calibration agreement
V	^{51}V	Botanical CRM used as a surrogate for bark, leaves, and flowers	NIST SRM 1573a Tomato Leaves	0.835 ± 0.034 mg kg^{-1}	0.807 ± 0.019 mg kg^{-1}	96.6	3.4	Satisfactory agreement
Ba	^{137}Ba	Second-source calibration verification	Second-source certified standard solution†	15.00 µg L^{-1}	14.51 ± 0.29 µg L^{-1}	96.7	3.3	Satisfactory calibration agreement
Sr	^{88}Sr	Food-matrix CRM used as a surrogate for fruit	NIST SRM 1568c Rice Flour	0.483 ± 0.010 mg kg^{-1}	0.475 ± 0.008 mg kg^{-1}	98.3	1.7	Excellent agreement
Li	^{7}Li	Second-source calibration verification	Second-source certified standard solution†	12.50 µg L^{-1}	12.10 ± 0.29 µg L^{-1}	96.8	3.2	Satisfactory calibration agreement
Rb	^{85}Rb	Botanical CRM used as a surrogate for bark, leaves, and flowers	NIST SRM 1573a Tomato Leaves	14.83 ± 0.31 mg kg^{-1}	14.71 ± 0.22 mg kg^{-1}	99.2	0.8	Excellent agreement

Cs	^{133}Cs	Second-source calibration verification	Second-source certified standard solution†	2.00 μg L⁻¹	1.99 ± 0.04 μg L⁻¹	99.5	0.5	Excellent calibration agreement
Pb	^{208}Pb	Food-matrix CRM used as a surrogate for fruit	NIST SRM 1568c Rice Flour	0.0168 ± 0.0037 mg kg⁻¹	0.0162 ± 0.0005 mg kg⁻¹	96.4	3.6	Satisfactory agreement
Cd	^{111}Cd	Food-matrix CRM used as a surrogate for fruit	NIST SRM 1568c Rice Flour	0.00989 ± 0.00040 mg kg⁻¹	0.009692 ± 0.000271 mg kg⁻¹	98.0	2.0	Excellent agreement
As	^{75}As	Soil CRM comparator (aqua-regia-extractable value)*	ERM-CC141 Loam Soil	7.5 ± 1.4 mg kg⁻¹	7.18 ± 0.19 mg kg⁻¹	95.7	4.3	Indicative only; method equivalence required*
Hg	^{202}Hg	Food-matrix CRM used as a surrogate for fruit	NIST SRM 1568c Rice Flour	0.001785 ± 0.000050 mg kg⁻¹	0.001635 ± 0.000069 mg kg⁻¹	91.6	8.4	Acceptable agreement
Cr	^{52}Cr	Soil CRM comparator (aqua-regia-extractable value)*	ERM-CC141 Loam Soil	31 ± 4 mg kg⁻¹	29.30 ± 0.76 mg kg⁻¹	94.5	5.5	Indicative only; method equivalence required*
Ni	^{60}Ni	Soil CRM comparator (aqua-regia-extractable value)*	ERM-CC141 Loam Soil	21.9 ± 1.6 mg kg⁻¹	21.24 ± 0.47 mg kg⁻¹	97.0	3.0	Indicative only; method equivalence required*
Sb	^{121}Sb	Botanical CRM used as a surrogate for bark, leaves, and flowers	NIST SRM 1573a Tomato Leaves	0.0619 ± 0.0032 mg kg⁻¹	0.0592 ± 0.0018 mg kg⁻¹	95.6	4.4	Satisfactory agreement
Sn	^{118}Sn	Second-source calibration verification	Second-source certified standard solution†	5.00 μg L⁻¹	4.86 ± 0.12 μg L⁻¹	97.2	2.8	Satisfactory calibration agreement

Notes: Recovery (%) = (measured mean/certified or target value) × 100. Relative error (%) = ∣measured mean − certified or target value∣/(certified or target value) × 100. CRM uncertainties are the expanded uncertainties reported in the official certificates, whereas measured uncertainties are expressed as SDs from three independently prepared determinations. Interpretation of relative error: ≤2.0%, excellent agreement; >2.0–5.0%, satisfactory agreement; >5.0–10.0%, acceptable agreement. †For Ti, Ba, Li, Cs, and Sn, trueness was assessed using second-source certified standard solutions at independently prepared target concentrations. *ERM-CC141 values are certified for aqua-regia extraction; because the present study used HNO_3–H_2O_2 digestion, these values were used as method-performance comparators rather than as strict CRM recovery criteria unless methodological equivalence could be demonstrated.

Table S10. Physicochemical characteristics of soil samples collected across the five ecological sectors of the Oradea metropolitan area.

Parameter	Z1 Eastern periurban sector (n = 13)	Z2 Central urban sector (n = 24)	Z3 Western transitional sector (n = 20)	Z4 Woodland-associated sector (n = 18)	Z5 Southern & southwestern periurban sector (n = 19)
pH	7.12 ± 0.28^{bc}	7.46 ± 0.35^{a}	7.21 ± 0.31^{b}	6.78 ± 0.27^{d}	7.05 ± 0.29^{cd}
Electrical conductivity of the 1:5 soil-to-water extract ($EC_{1:5}$, µS cm^{-1})	312 ± 54^{c}	487 ± 91^{a}	356 ± 63^{b}	228 ± 47^{d}	298 ± 58^{c}
Organic matter (OM, %)	4.21 ± 0.73^{c}	3.12 ± 0.69^{d}	4.68 ± 0.82^{bc}	6.14 ± 1.03^{a}	4.95 ± 0.88^{b}
Estimated soil organic carbon (SOC_{est}, %)	2.44 ± 0.42^{c}	1.81 ± 0.38^{d}	2.71 ± 0.47^{bc}	3.56 ± 0.59^{a}	2.87 ± 0.51^{b}
Clay fraction (<2 µm, %)	29.6 ± 6.2^{b}	34.8 ± 7.1^{a}	27.5 ± 5.4^{bc}	24.2 ± 4.9^{c}	30.1 ± 6.0^{ab}
Silt fraction (2–50 µm, %)	42.8 ± 5.9^{ab}	45.7 ± 6.4^{a}	39.1 ± 5.7^{c}	41.6 ± 6.2^{bc}	40.8 ± 5.8^{bc}
Sand fraction (50–2000 µm, %)	27.6 ± 4.8^{c}	19.5 ± 4.1^{d}	33.4 ± 5.5^{a}	34.2 ± 5.7^{a}	29.1 ± 5.1^{b}
Calcium carbonate content ($CaCO_3$, %)	6.8 ± 1.9^{b}	9.7 ± 2.4^{a}	5.9 ± 1.7^{c}	3.2 ± 1.1^{d}	5.4 ± 1.6^{c}
Potential cation exchange capacity (CEC_{pot}, cmol(+) kg^{-1})	18.4 ± 3.1^{b}	22.8 ± 4.2^{a}	17.2 ± 2.9^{bc}	15.9 ± 2.7^{c}	18.8 ± 3.3^{b}
Total nitrogen (TN, %)	0.21 ± 0.04^{c}	0.17 ± 0.03^{d}	0.24 ± 0.05^{bc}	0.31 ± 0.06^{a}	0.26 ± 0.05^{b}
Olsen-extractable phosphorus (P, mg kg^{-1})	38.5 ± 9.4^{bc}	52.8 ± 12.6^{a}	34.2 ± 8.8^{c}	27.6 ± 7.1^{d}	36.9 ± 9.2^{bc}
Ammonium acetate-extractable	182 ± 34^{b}	241 ± 48^{a}	176 ± 36^{bc}	154 ± 31^{c}	189 ± 39^{ab}

potassium (K, mg kg^{-1})					
Gravimetric moisture content, wet-mass basis (%)	18.7 ± 3.8^{c}	15.2 ± 3.1^{d}	20.4 ± 4.2^{bc}	24.9 ± 5.1^{a}	21.6 ± 4.4^{b}
Dry bulk density (g cm^{-3})	1.32 ± 0.09^{b}	1.46 ± 0.11^{a}	1.28 ± 0.08^{bc}	1.18 ± 0.07^{d}	1.25 ± 0.08^{c}
Sum of DTPA-extractable Fe, Mn, Zn, and Cu (ΣDTPA, mg kg^{-1})	7.4 ± 2.1^{c}	15.8 ± 4.6^{a}	8.9 ± 2.7^{b}	5.1 ± 1.8^{d}	7.2 ± 2.3^{c}
Saturated hydraulic conductivity (K_{sat}, cm h^{-1})	2.8 ± 0.7^{c}	1.9 ± 0.5^{d}	3.4 ± 0.9^{b}	4.1 ± 1.0^{a}	3.2 ± 0.8^{bc}
Electrical conductivity of the saturated-paste extract (EC_e, dS m^{-1})	0.42 ± 0.11^{c}	0.87 ± 0.19^{a}	0.51 ± 0.13^{bc}	0.31 ± 0.09^{d}	0.46 ± 0.12^{bc}

Note: Values are presented as mean ± standard deviation (SD). Different lowercase superscript letters within the same row indicate statistically significant differences among ecological sectors according to one-way analysis of variance followed by Tukey's honestly significant difference test ($p < 0.05$). The value of n represents the number of independent sampling sites within each ecological sector. Analytical replicates were averaged to obtain one value for each sampling site before sector-level statistical analysis. For soil properties determined using undisturbed cores, the measurements obtained from the three cores collected at each site were averaged to obtain one site-level value. Moisture content is expressed on a wet-mass basis, whereas concentration-based parameters are expressed on a dry-weight basis unless otherwise specified. Estimated soil organic carbon (SOC_{est}) was calculated as OM/1.724. The ΣDTPA parameter represents the sum of the DTPA-extractable concentrations of Fe, Mn, Zn, and Cu.

Table S11. Individual concentrations of soil macroelements measured in georeferenced soil samples collected across the investigated ecological sectors of the Oradea metropolitan area, Romania.

Sample ID	Zone	K (mg/kg)	Ca (mg/kg)	Mg (mg/kg)	Na (mg/kg)	P (mg/kg)	S (mg/kg)
SN-SO-022	Z1	1864 ± 143^{lm}	8421 ± 611^{j}	1247 ± 96^{k}	312 ± 24^{j}	41.8 ± 3.7^{j}	912 ± 68^{j}
SN-SO-023	Z1	1738 ± 128°	7916 ± 574^{l}	1183 ± 88^{m}	284 ± 21^{l}	36.9 ± 3.0^{l}	854 ± 61^{l}
SN-SO-026	Z1	2147 ± 182^{i}	9534 ± 742^{h}	1496 ± 123^{f}	381 ± 33^{h}	48.2 ± 4.8^{h}	1068 ± 87^{h}
SN-SO-027	Z1	1659 ± 119^{r}	7642 ± 548^{m}	1115 ± 82^{o}	261 ± 19^{n}	34.1 ± 2.8^{n}	801 ± 56^{n}
SN-SO-029	Z1	2284 ± 196^{g}	10122 ± 817^{f}	1578 ± 131^{e}	416 ± 38^{f}	53.6 ± 5.2^{f}	1148 ± 94^{f}
SN-SO-053	Z1	1928 ± 151^{k}	8675 ± 639^{j}	1294 ± 102^{j}	327 ± 25^{i}	42.7 ± 3.7^{j}	936 ± 73^{j}
SN-SO-061	Z1	2416 ± 214^{ef}	10854 ± 891^{d}	1698 ± 147^{c}	463 ± 41^{e}	58.4 ± 5.8^{e}	1267 ± 108^{e}
SN-SO-062	Z1	1834 ± 136^{m}	8238 ± 596^{k}	1221 ± 91^{l}	301 ± 22^{k}	39.5 ± 3.2^{k}	887 ± 64^{k}
SN-SO-063	Z1	1588 ± 114^{r}	7314 ± 523^{n}	1064 ± 78^{p}	248 ± 18^{o}	31.8 ± 2.5^{o}	742 ± 51^{o}
SN-SO-081	Z1	2193 ± 187^{hi}	9726 ± 781^{h}	1511 ± 126^{f}	394 ± 35^{g}	50.7 ± 5.0^{g}	1096 ± 91^{g}
SN-SO-082	Z1	1761 ± 129no	8041 ± 581^{l}	1178 ± 87^{m}	289 ± 20^{l}	37.2 ± 3.1^{l}	861 ± 62^{l}
SN-SO-083	Z1	2057 ± 169^{j}	9148 ± 718^{i}	1386 ± 114^{h}	352 ± 30^{i}	45.1 ± 4.3^{i}	997 ± 81^{i}
SN-SO-084	Z1	2338 ± 204^{fg}	10397 ± 842^{e}	1624 ± 139^{d}	438 ± 39^{f}	55.2 ± 5.5^{f}	1193 ± 101^{f}
SN-SO-003	Z2	2648 ± 231^{cd}	12482 ± 1043^{b}	1847 ± 158^{b}	618 ± 57^{c}	71.4 ± 7.1^{c}	1486 ± 124^{c}
SN-SO-004	Z2	2487 ± 216^{e}	11835 ± 972^{c}	1764 ± 149^{c}	572 ± 51^{d}	66.8 ± 6.5^{d}	1398 ± 117^{d}
SN-SO-014	Z2	2814 ± 248^{bc}	13246 ± 1118^{ab}	1968 ± 171^{a}	694 ± 64^{b}	78.5 ± 7.9^{b}	1594 ± 136^{b}
SN-SO-018	Z2	2361 ± 203^{f}	11214 ± 917^{d}	1682 ± 141^{c}	536 ± 47^{d}	61.3 ± 5.8^{e}	1312 ± 109^{e}
SN-SO-019	Z2	2947 ± 267^{ab}	13888 ± 1184^{a}	1930 ± 171^{a}	742 ± 71^{ab}	83.6 ± 8.4^{a}	1679 ± 148^{ab}
SN-SO-020	Z2	2593 ± 224^{d}	12156 ± 1017^{b}	1811 ± 154^{b}	604 ± 55^{c}	69.7 ± 6.8^{c}	1451 ± 121^{c}
SN-SO-048	Z2	2736 ± 239^{c}	12874 ± 1076^{b}	1916 ± 166^{a}	661 ± 62^{b}	75.1 ± 7.4^{b}	1542 ± 132^{b}
SN-SO-049	Z2	2298 ± 197^{g}	10937 ± 894^{d}	1624 ± 136^{d}	501 ± 45^{e}	58.8 ± 5.5^{e}	1268 ± 103^{e}
SN-SO-050	Z2	3015 ± 278^{a}	14256 ± 1237^{a}	1990 ± 178^{a}	781 ± 74^{a}	87.4 ± 8.9^{a}	1728 ± 156^{a}
SN-SO-051	Z2	2451 ± 212^{ef}	11682 ± 954^{c}	1738 ± 146^{c}	548 ± 49^{d}	64.9 ± 6.1^{d}	1367 ± 113^{d}
SN-SO-052	Z2	2867 ± 256^{b}	13524 ± 1146^{a}	2014 ± 178^{a}	718 ± 69^{ab}	81.2 ± 8.1^{ab}	1648 ± 143^{b}

SN-SO-054	Z2	2524 ± 219^{de}	11948 ± 986^{c}	1786 ± 151^{b}	586 ± 53^{c}	68.1 ± 6.7^{c}	1415 ± 119^{c}
SN-SO-055	Z2	2679 ± 235^{cd}	12631 ± 1054^{b}	1889 ± 163^{ab}	639 ± 59^{bc}	73.5 ± 7.2^{b}	1511 ± 128^{b}
SN-SO-056	Z2	2217 ± 188^{h}	10495 ± 861^{e}	1568 ± 131^{e}	472 ± 42^{e}	55.4 ± 5.2^{f}	1194 ± 96^{f}
SN-SO-057	Z2	3098 ± 286^{a}	14637 ± 1288^{a}	2050 ± 186^{a}	814 ± 79^{a}	91.7 ± 9.4^{a}	1796 ± 163^{a}
SN-SO-059	Z2	2386 ± 205^{f}	11356 ± 929^{cd}	1694 ± 143^{c}	527 ± 48^{d}	62.4 ± 5.9^{d}	1328 ± 111^{de}
SN-SO-064	Z2	2761 ± 241^{bc}	13042 ± 1092^{ab}	1941 ± 168^{a}	672 ± 63^{b}	76.8 ± 7.6^{b}	1564 ± 134^{b}
SN-SO-065	Z2	2578 ± 223^{d}	12237 ± 1026^{b}	1824 ± 155^{b}	611 ± 56^{c}	70.5 ± 6.9^{c}	1463 ± 122^{c}
SN-SO-069	Z2	2926 ± 264^{ab}	13741 ± 1173^{a}	1910 ± 168^{a}	736 ± 70^{ab}	84.1 ± 8.5^{a}	1688 ± 149^{ab}
SN-SO-085	Z2	2334 ± 199^{fg}	11084 ± 905^{d}	1651 ± 138^{d}	512 ± 46^{e}	59.7 ± 5.6^{e}	1286 ± 104^{e}
SN-SO-086	Z2	2841 ± 251^{b}	13318 ± 1127^{ab}	1984 ± 173^{a}	701 ± 66^{b}	79.6 ± 8.0^{b}	1617 ± 139^{b}
SN-SO-087	Z2	2489 ± 217^{e}	11864 ± 975^{c}	1769 ± 150^{c}	573 ± 52^{d}	66.9 ± 6.5^{d}	1401 ± 117^{d}
SN-SO-088	Z2	2974 ± 271^{ab}	14022 ± 1203^{a}	1940 ± 173^{a}	754 ± 72^{a}	85.8 ± 8.7^{a}	1704 ± 152^{a}
SN-SO-094	Z2	2612 ± 227^{cd}	12314 ± 1034^{b}	1837 ± 157^{b}	623 ± 58^{c}	71.1 ± 7.0^{c}	1478 ± 123^{c}
SN-SO-001	Z3	2148 ± 181^{i}	9634 ± 772^{h}	1482 ± 123^{f}	418 ± 36^{f}	49.2 ± 4.8^{h}	1087 ± 91^{h}
SN-SO-005	Z3	1824 ± 148^{mn}	8142 ± 633^{k}	1241 ± 97^{k}	297 ± 24^{k}	36.8 ± 3.2^{l}	842 ± 67^{l}
SN-SO-006	Z3	2486 ± 224^{e}	11284 ± 946^{cd}	1748 ± 153^{c}	561 ± 52^{d}	66.2 ± 6.8^{d}	1396 ± 124^{d}
SN-SO-007	Z3	1687 ± 129^{p}	7534 ± 571^{m}	1138 ± 84^{n}	271 ± 20^{m}	32.1 ± 2.7^{n}	756 ± 58^{n}
SN-SO-008	Z3	2574 ± 238^{d}	11648 ± 987^{c}	1812 ± 161^{b}	604 ± 57^{c}	71.8 ± 7.5^{c}	1488 ± 136^{c}
SN-SO-032	Z3	2053 ± 171^{j}	9187 ± 726^{i}	1417 ± 117^{g}	382 ± 32^{h}	45.8 ± 4.3^{i}	1014 ± 83^{i}
SN-SO-033	Z3	2318 ± 201^{fg}	10421 ± 861^{e}	1604 ± 137^{d}	472 ± 43^{e}	56.7 ± 5.7^{f}	932 ± 74^{j}
SN-SO-034	Z3	1598 ± 118^{r}	7126 ± 533^{n}	1064 ± 77^{p}	248 ± 18^{o}	29.4 ± 2.3^{p}	684 ± 49^{p}
SN-SO-035	Z3	2688 ± 249^{c}	12184 ± 1036^{b}	1886 ± 169^{ab}	812 ± 84^{a}	76.4 ± 8.0^{b}	1587 ± 148^{b}
SN-SO-036	Z3	1927 ± 158^{k}	8615 ± 674^{j}	1328 ± 107^{i}	341 ± 28^{i}	41.7 ± 3.8^{j}	936 ± 75^{j}
SN-SO-037	Z3	2416 ± 214^{ef}	10876 ± 902^{d}	1684 ± 147^{c}	528 ± 49^{d}	62.3 ± 6.4^{d}	1328 ± 117^{de}
SN-SO-038	Z3	1764 ± 137no	7864 ± 596^{l}	1187 ± 88^{m}	286 ± 22^{l}	34.2 ± 2.9^{n}	801 ± 61^{n}
SN-SO-039	Z3	2814 ± 267^{bc}	12754 ± 1098^{b}	1974 ± 181^{a}	851 ± 92^{a}	84.1 ± 8.9^{a}	1984 ± 201^{a}
SN-SO-040	Z3	2014 ± 166^{jk}	9017 ± 714^{i}	1386 ± 113^{h}	369 ± 31^{h}	44.6 ± 4.2^{i}	987 ± 81^{i}
SN-SO-041	Z3	2268 ± 193^{gh}	10162 ± 823^{f}	1567 ± 132^{e}	447 ± 39^{f}	52.8 ± 5.2^{f}	1141 ± 98^{f}

SN-SO-058	Z3	1481 ± 106st	6617 ± 487^{o}	986 ± 71^{r}	214 ± 15^{r}	25.6 ± 2.0^{r}	603 ± 43^{r}
SN-SO-060	Z3	2534 ± 231^{de}	11476 ± 972^{c}	1771 ± 158^{c}	586 ± 55^{c}	68.2 ± 7.0^{c}	1424 ± 129^{c}
SN-SO-089	Z3	1886 ± 152^{l}	8421 ± 651^{j}	1287 ± 102^{j}	319 ± 26^{j}	39.4 ± 3.5^{k}	891 ± 71^{k}
SN-SO-090	Z3	2368 ± 207^{f}	10654 ± 871^{e}	1642 ± 141^{d}	491 ± 45^{e}	58.6 ± 5.9^{e}	1248 ± 109^{e}
SN-SO-091	Z3	1714 ± 132^{op}	7642 ± 578^{m}	1148 ± 84^{n}	267 ± 19^{n}	31.8 ± 2.6^{o}	742 ± 54°
SN-SO-002	Z4	1548 ± 118^{s}	6842 ± 517°	1021 ± 74^{r}	214 ± 16^{r}	28.6 ± 2.4^{q}	648 ± 48^{q}
SN-SO-009	Z4	1687 ± 131^{p}	7421 ± 566^{n}	1118 ± 82°	238 ± 18^{p}	31.4 ± 2.7^{o}	712 ± 53^{p}
SN-SO-010	Z4	1472 ± 109^{t}	6538 ± 486°	1160 ± 88^{n}	201 ± 15^{r}	26.7 ± 2.1^{r}	617 ± 44^{r}
SN-SO-011	Z4	1764 ± 139no	7816 ± 603^{l}	1186 ± 88^{m}	254 ± 19°	33.8 ± 2.9^{n}	756 ± 58^{n}
SN-SO-012	Z4	1398 ± 102^{u}	6214 ± 461^{p}	1080 ± 81^{p}	184 ± 13^{s}	24.1 ± 1.9^{s}	582 ± 41^{s}
SN-SO-013	Z4	1821 ± 145^{mn}	8047 ± 624^{l}	1214 ± 92^{l}	267 ± 21^{n}	35.1 ± 3.1^{m}	781 ± 61^{n}
SN-SO-015	Z4	1615 ± 124^{r}	7138 ± 541^{n}	1074 ± 79^{p}	226 ± 17^{q}	29.8 ± 2.5^{p}	689 ± 51^{p}
SN-SO-016	Z4	1728 ± 136°	7681 ± 592^{m}	1153 ± 86^{n}	247 ± 18°	32.6 ± 2.8^{n}	734 ± 56°
SN-SO-017	Z4	1451 ± 106^{t}	6417 ± 472^{p}	1140 ± 86^{n}	193 ± 14^{s}	25.4 ± 2.0^{r}	603 ± 43^{r}
SN-SO-021	Z4	1886 ± 152^{l}	8324 ± 651^{j}	1267 ± 97^{j}	281 ± 22^{l}	37.2 ± 3.3^{l}	824 ± 66^{m}
SN-SO-024	Z4	1527 ± 116^{s}	6728 ± 504°	1210 ± 92^{l}	209 ± 16^{r}	27.9 ± 2.3^{q}	637 ± 46^{q}
SN-SO-025	Z4	1669 ± 129^{pr}	7396 ± 563^{n}	1107 ± 81°	234 ± 18^{p}	30.7 ± 2.6^{p}	701 ± 52^{p}
SN-SO-028	Z4	1438 ± 105^{t}	6341 ± 468^{p}	1110 ± 84°	189 ± 14^{s}	24.8 ± 2.0^{s}	596 ± 42^{r}
SN-SO-030	Z4	1794 ± 142^{n}	7928 ± 617^{l}	1195 ± 89^{m}	261 ± 20^{n}	34.3 ± 3.0^{n}	769 ± 59^{n}
SN-SO-031	Z4	1581 ± 121^{r}	6984 ± 529^{n}	1049 ± 77$^{\varphi}$	221 ± 17^{q}	29.1 ± 2.4^{q}	673 ± 49^{q}
SN-SO-070	Z4	1714 ± 134^{op}	7592 ± 583^{m}	1138 ± 84^{n}	242 ± 18^{p}	31.9 ± 2.7^{o}	726 ± 55°
SN-SO-071	Z4	1496 ± 111st	6612 ± 492°	1180 ± 89^{m}	204 ± 15^{r}	26.2 ± 2.1^{r}	621 ± 45^{r}
SN-SO-072	Z4	1842 ± 148^{m}	8175 ± 638^{k}	1236 ± 95^{k}	273 ± 21^{m}	36.1 ± 3.2^{l}	798 ± 63^{n}
SN-SO-042	Z5	1984 ± 163^{jk}	8847 ± 684^{ij}	1321 ± 106^{i}	336 ± 28^{i}	42.6 ± 3.9^{j}	951 ± 77^{j}
SN-SO-043	Z5	2147 ± 182^{i}	9568 ± 742^{h}	1442 ± 119^{g}	381 ± 34^{h}	47.8 ± 4.6^{h}	1038 ± 86^{h}
SN-SO-044	Z5	1862 ± 151^{lm}	8264 ± 637^{k}	1238 ± 97^{k}	301 ± 23^{k}	38.9 ± 3.4^{k}	889 ± 69^{k}
SN-SO-045	Z5	2258 ± 194^{gh}	10074 ± 817^{f}	1528 ± 128^{f}	486 ± 42^{e}	78.2 ± 7.8^{b}	1288 ± 106^{e}
SN-SO-046	Z5	1769 ± 137no	7918 ± 603^{l}	1186 ± 88^{m}	287 ± 21^{l}	36.4 ± 3.0^{l}	842 ± 63^{l}

SN-SO-047	Z5	2690 ± 231^{c}	10426 ± 861^{e}	1594 ± 136^{e}	538 ± 48^{d}	84.6 ± 8.5^{a}	1694 ± 161^{a}
SN-SO-066	Z5	1914 ± 156^{kl}	8517 ± 661^{j}	1274 ± 101^{j}	319 ± 26^{j}	40.7 ± 3.7^{j}	914 ± 73^{j}
SN-SO-067	Z5	2088 ± 173^{j}	9286 ± 718^{i}	1388 ± 114^{h}	364 ± 31^{h}	45.9 ± 4.3^{i}	992 ± 81^{i}
SN-SO-068	Z5	1834 ± 144^{m}	8141 ± 621^{k}	1217 ± 93^{l}	296 ± 22^{k}	37.8 ± 3.2^{l}	867 ± 67^{l}
SN-SO-073	Z5	2217 ± 188^{h}	9861 ± 794^{g}	1496 ± 124^{f}	472 ± 41^{e}	76.5 ± 7.6^{b}	1018 ± 83^{i}
SN-SO-074	Z5	1952 ± 161^{k}	8694 ± 676^{j}	1305 ± 104^{i}	327 ± 27^{i}	41.8 ± 3.8^{j}	938 ± 75^{j}
SN-SO-075	Z5	2780 ± 238^{bc}	10687 ± 892^{e}	1638 ± 142^{d}	734 ± 78^{ab}	88.9 ± 8.9^{a}	1821 ± 176^{a}
SN-SO-076	Z5	1791 ± 139^{n}	7983 ± 609^{l}	1198 ± 90^{m}	289 ± 21^{l}	36.9 ± 3.1^{l}	853 ± 64^{l}
SN-SO-077	Z5	2126 ± 179^{ij}	9441 ± 731^{hi}	1416 ± 117^{g}	376 ± 33^{h}	46.7 ± 4.5^{hi}	1017 ± 84^{i}
SN-SO-078	Z5	1876 ± 149^{l}	8338 ± 648^{j}	1252 ± 99^{k}	304 ± 24^{jk}	39.5 ± 3.5^{k}	891 ± 70^{k}
SN-SO-079	Z5	2610 ± 225^{cd}	10143 ± 826^{f}	1541 ± 131^{f}	521 ± 47^{d}	82.4 ± 8.2^{ab}	1338 ± 114^{d}
SN-SO-080	Z5	1724 ± 132°	7687 ± 584^{m}	1147 ± 84^{n}	271 ± 20^{m}	34.8 ± 2.9^{m}	811 ± 59^{m}
SN-SO-092	Z5	2184 ± 186^{hi}	9724 ± 781^{h}	1478 ± 123^{f}	463 ± 40^{e}	72.8 ± 7.3^{bc}	964 ± 77^{j}
SN-SO-093	Z5	1938 ± 158^{k}	8611 ± 667^{j}	1288 ± 102^{j}	322 ± 26^{j}	41.2 ± 3.7^{j}	926 ± 74^{j}

Notes: Values are reported as mean ± analytical SD of three independently prepared analytical portions on a dry-weight basis (mg/kg DW). The three analytical measurements were used only to assess analytical precision and were averaged to obtain one site-level concentration per matrix and georeferenced sampling site for subsequent statistical analyses. Values <LOD and <LOQ are indicated as applicable.

Table S12. Individual concentrations of environmentally associated microelements and biologically relevant trace elements measured in georeferenced soil samples collected across the investigated ecological sectors of the Oradea metropolitan area, Romania.

Sample ID	Zone	^{56}Fe (mg/kg)	^{55}Mn (mg/kg)	^{64}Zn (mg/kg)	^{63}Cu (mg/kg)	^{11}B (mg/kg)	^{98}Mo (mg/kg)	^{59}Co (mg/kg)	^{80}Se (mg/kg)
SN-SO-022	Z1	27872 ± 1458^{g}	760 ± 63^{f}	52.3 ± 3.5^{j}	18.3 ± 1.6^{j}	16.7 ± 1.2h	2.74 ± 0.26^{c}	7.2 ± 0.5^{i}	0.43 ± 0.04^{g}
SN-SO-023	Z1	35742 ± 2147^{e}	452 ± 32^{j}	66.2 ± 5.5^{i}	39.3 ± 2.8^{f}	19.7 ± 1.2^{f}	2.83 ± 0.34^{bc}	7.2 ± 0.5^{i}	0.71 ± 0.07^{ab}
SN-SO-026	Z1	32164 ± 1876^{ef}	449 ± 34^{j}	50.0 ± 3.1^{j}	43.2 ± 3.2^{e}	16.3 ± 1.5^{h}	0.66 ± 0.07^{j}	8.2 ± 0.8^{hi}	0.31 ± 0.02^{jk}
SN-SO-027	Z1	25378 ± 1321^{gh}	426 ± 28^{j}	43.1 ± 3.4^{k}	23.4 ± 2.4^{i}	18.9 ± 1.4^{fg}	1.77 ± 0.18^{h}	15.4 ± 1.0^{d}	0.52 ± 0.05^{e}
SN-SO-029	Z1	37082 ± 2418^{de}	804 ± 70^{f}	90.1 ± 8.9^{f}	16.7 ± 1.1^{j}	13.4 ± 1.0^{i}	1.83 ± 0.14^{h}	9.0 ± 0.7^{h}	0.48 ± 0.04^{ef}
SN-SO-053	Z1	37428 ± 2264^{de}	442 ± 39^{j}	92.2 ± 8.1^{f}	38.9 ± 3.3^{f}	22.5 ± 2.1^{de}	1.33 ± 0.10^{i}	5.4 ± 0.5^{j}	0.67 ± 0.06^{b}
SN-SO-061	Z1	35726 ± 2148^{e}	593 ± 53^{hi}	57.1 ± 3.6^{ij}	24.0 ± 1.6^{i}	16.4 ± 1.3^{h}	0.61 ± 0.05^{j}	15.4 ± 1.1^{d}	0.74 ± 0.07^{a}
SN-SO-062	Z1	30819 ± 1738^{f}	545 ± 35^{i}	57.0 ± 4.6^{ij}	40.4 ± 4.2^{f}	23.7 ± 2.4^{d}	2.04 ± 0.25^{fg}	6.6 ± 0.5^{ij}	0.59 ± 0.05^{cd}
SN-SO-063	Z1	30320 ± 1812^{f}	569 ± 56^{hi}	105.5 ± 7.7^{e}	29.0 ± 2.2^{h}	7.1 ± 0.6^{k}	1.53 ± 0.13^{i}	8.6 ± 0.9^{h}	0.44 ± 0.04^{fg}
SN-SO-081	Z1	33518 ± 2203^{ef}	505 ± 43^{ij}	92.3 ± 6.6^{f}	37.6 ± 2.9^{fg}	7.7 ± 0.7^{k}	1.73 ± 0.13^{h}	15.2 ± 1.1^{d}	0.51 ± 0.04^{e}
SN-SO-082	Z1	31438 ± 1926^{f}	646 ± 44^{gh}	78.0 ± 7.4^{g}	27.0 ± 2.9^{h}	18.4 ± 1.7^{g}	1.81 ± 0.14^{h}	16.4 ± 1.2^{cd}	0.65 ± 0.06^{bc}
SN-SO-083	Z1	32309 ± 2113^{ef}	505 ± 32^{ij}	101.2 ± 10.6^{e}	34.6 ± 2.7^{g}	9.5 ± 0.7^{j}	0.82 ± 0.10^{j}	15.1 ± 1.4^{d}	0.56 ± 0.05^{d}
SN-SO-084	Z1	42136 ± 2284^{c}	432 ± 37^{j}	38.9 ± 3.4^{k}	37.8 ± 2.7^{f}	13.3 ± 1.3^{i}	2.66 ± 0.33^{cd}	16.0 ± 1.5^{cd}	0.68 ± 0.06^{ab}
SN-SO-003	Z2	44872 ± 2631^{bc}	986 ± 84^{de}	128.4 ± 11.2^{cd}	51.6 ± 4.7^{cd}	19.8 ± 1.4^{f}	1.39 ± 0.15^{i}	18.7 ± 1.5^{b}	0.49 ± 0.04^{ef}
SN-SO-004	Z2	48516 ± 3184^{ab}	1044 ± 92^{cd}	137.9 ± 12.1^{c}	55.3 ± 5.1^{bc}	25.8 ± 2.3^{c}	2.72 ± 0.24^{c}	19.8 ± 1.7^{ab}	0.63 ± 0.06^{bc}
SN-SO-014	Z2	39274 ± 2477^{d}	1196 ± 106^{ab}	154.2 ± 14.3^{ab}	63.1 ± 5.9^{a}	27.6 ± 2.4^{bc}	2.93 ± 0.27^{bc}	21.2 ± 1.9^{a}	0.18 ± 0.01^{mn}
SN-SO-018	Z2	50782 ± 3721^{a}	874 ± 72^{ef}	111.6 ± 9.8^{de}	44.2 ± 4.0^{e}	32.8 ± 3.0^{a}	3.38 ± 0.32^{ab}	16.1 ± 1.3^{cd}	0.15 ± 0.01^{o}
SN-SO-019	Z2	43118 ± 2542^{c}	1268 ± 114^{a}	166.8 ± 15.6^{a}	67.4 ± 6.3^{a}	22.3 ± 1.9^{de}	2.31 ± 0.21^{e}	22.3 ± 2.0^{a}	0.35 ± 0.03^{i}
SN-SO-020	Z2	46344 ± 2518^{b}	1012 ± 86^{cd}	132.1 ± 11.7^{cd}	52.9 ± 4.8^{c}	34.6 ± 3.1^{a}	3.62 ± 0.35^{a}	18.9 ± 1.6^{b}	0.12 ± 0.01^{p}
SN-SO-048	Z2	37486 ± 2261^{de}	1127 ± 98^{bc}	146.7 ± 13.1^{bc}	59.6 ± 5.4^{ab}	26.9 ± 2.3^{bc}	2.88 ± 0.26^{bc}	20.3 ± 1.8^{ab}	0.46 ± 0.04^{f}
SN-SO-049	Z2	40627 ± 2388^{cd}	812 ± 68^{f}	102.4 ± 8.7^{e}	39.8 ± 3.5^{f}	30.2 ± 2.7^{ab}	3.17 ± 0.30^{b}	14.9 ± 1.2^{de}	0.24 ± 0.02^{l}
SN-SO-050	Z2	47218 ± 2944^{b}	921 ± 77^{de}	118.6 ± 10.1^{d}	46.7 ± 4.1^{de}	20.4 ± 1.8^{ef}	2.04 ± 0.18^{fg}	16.8 ± 1.4^{c}	0.57 ± 0.05^{d}
SN-SO-051	Z2	38412 ± 2310^{d}	1164 ± 102^{b}	151.3 ± 13.9^{b}	61.7 ± 5.7^{a}	23.7 ± 2.0^{d}	2.44 ± 0.22^{de}	20.8 ± 1.9^{ab}	0.17 ± 0.01^{n}
SN-SO-052	Z2	44976 ± 2764^{bc}	846 ± 70^{ef}	108.8 ± 9.4^{de}	42.3 ± 3.8^{e}	31.5 ± 2.8^{a}	3.29 ± 0.31^{ab}	15.7 ± 1.3^{d}	0.09 ± 0.01^{qr}
SN-SO-054	Z2	39584 ± 2422^{d}	1076 ± 93^{c}	139.5 ± 12.4^{c}	56.8 ± 5.2^{b}	21.6 ± 1.8^{e}	2.18 ± 0.20^{ef}	19.4 ± 1.7^{b}	0.41 ± 0.04^{gh}

SN-SO-055	Z2	48874 ± 3276^{ab}	899 ± 75^{e}	113.2 ± 9.8^{d}	45.1 ± 4.0^{de}	28.4 ± 2.5^{b}	2.96 ± 0.27^{bc}	16.4 ± 1.4^{cd}	0.13 ± 0.01^{op}
SN-SO-056	Z2	36792 ± 2194^{de}	1218 ± 108^{ab}	158.1 ± 14.6^{ab}	64.6 ± 6.0^{a}	22.8 ± 1.9^{de}	2.37 ± 0.21^{de}	21.5 ± 2.0^{a}	0.28 ± 0.02^{k}
SN-SO-057	Z2	41866 ± 2496^{cd}	788 ± 64^{f}	98.6 ± 8.2^{ef}	38.4 ± 3.2^{f}	33.4 ± 3.0^{a}	3.44 ± 0.33^{ab}	14.2 ± 1.1^{e}	0.52 ± 0.05^{e}
SN-SO-059	Z2	40518 ± 2411^{cd}	957 ± 81^{de}	124.8 ± 10.9^{cd}	49.8 ± 4.4^{cd}	19.7 ± 1.6^{f}	1.96 ± 0.17^{g}	17.9 ± 1.5^{bc}	0.61 ± 0.06^{c}
SN-SO-064	Z2	47728 ± 3018^{b}	932 ± 79^{de}	116.4 ± 10.0^{d}	47.2 ± 4.2^{d}	25.1 ± 2.2^{c}	2.63 ± 0.24^{cd}	17.1 ± 1.4^{c}	0.54 ± 0.05^{de}
SN-SO-065	Z2	49984 ± 3510^{ab}	1187 ± 105^{b}	153.6 ± 14.1^{b}	62.4 ± 5.8^{a}	23.9 ± 2.1^{d}	2.49 ± 0.23^{d}	21.0 ± 1.9^{a}	0.66 ± 0.06^{b}
SN-SO-069	Z2	44312 ± 2687^{bc}	1246 ± 112^{a}	164.3 ± 15.1^{a}	66.8 ± 6.2^{a}	32.1 ± 2.9^{a}	3.33 ± 0.32^{ab}	21.9 ± 2.0^{a}	0.43 ± 0.04^{g}
SN-SO-085	Z2	38964 ± 2336^{d}	1034 ± 88^{cd}	135.7 ± 12.0^{c}	54.6 ± 4.9^{bc}	34.1 ± 3.1^{a}	3.56 ± 0.34^{a}	19.1 ± 1.7^{b}	0.39 ± 0.03^{h}
SN-SO-086	Z2	41276 ± 2459^{cd}	861 ± 71^{ef}	109.7 ± 9.3^{de}	43.6 ± 3.9^{e}	27.4 ± 2.4^{bc}	2.91 ± 0.27^{bc}	15.9 ± 1.3^{cd}	0.31 ± 0.02^{jk}
SN-SO-087	Z2	46892 ± 2874^{b}	944 ± 80^{de}	121.3 ± 10.6^{d}	48.7 ± 4.3^{d}	21.9 ± 1.9^{e}	2.24 ± 0.20^{e}	17.5 ± 1.5^{c}	0.36 ± 0.03^{i}
SN-SO-088	Z2	43244 ± 2598^{c}	1142 ± 99^{bc}	148.9 ± 13.5^{bc}	60.4 ± 5.5^{ab}	24.5 ± 2.1^{cd}	2.57 ± 0.23^{cd}	20.5 ± 1.8^{ab}	0.14 ± 0.01^{o}
SN-SO-094	Z2	26880 ± 1518^{g}	1006 ± 86^{cd}	130.9 ± 11.5^{cd}	52.1 ± 4.7^{c}	30.7 ± 2.7^{ab}	3.21 ± 0.30^{b}	18.6 ± 1.6^{b}	0.11 ± 0.01^{pq}
SN-SO-001	Z3	47372 ± 3091^{b}	1103 ± 88^{bc}	113.5 ± 7.4^{d}	40.6 ± 3.5^{f}	26.3 ± 2.3^{c}	2.81 ± 0.25^{bc}	11.8 ± 0.8^{fg}	0.27 ± 0.02^{kl}
SN-SO-005	Z3	40538 ± 2437^{cd}	982 ± 89^{de}	109.8 ± 7.1^{de}	44.7 ± 4.0^{e}	15.8 ± 1.2^{h}	1.86 ± 0.13^{h}	16.7 ± 1.5^{c}	0.08 ± 0.01^{r}
SN-SO-006	Z3	49267 ± 4018^{ab}	555 ± 36^{i}	82.1 ± 5.6^{g}	28.6 ± 2.1^{h}	27.9 ± 2.3^{b}	1.10 ± 0.10^{ij}	13.2 ± 1.4^{ef}	0.33 ± 0.03^{j}
SN-SO-007	Z3	28756 ± 1714^{fg}	1084 ± 76^{c}	73.8 ± 6.9^{h}	62.8 ± 5.5^{a}	14.2 ± 1.5^{i}	1.02 ± 0.10^{j}	11.9 ± 1.0^{fg}	0.19 ± 0.01^{m}
SN-SO-008	Z3	40295 ± 2381^{cd}	693 ± 57^{g}	72.0 ± 6.1^{h}	44.5 ± 2.8^{e}	23.7 ± 1.8^{d}	2.95 ± 0.24^{bc}	12.7 ± 0.9^{f}	0.42 ± 0.04^{gh}
SN-SO-032	Z3	45086 ± 2716^{bc}	784 ± 58^{f}	157.2 ± 16.0^{ab}	64.4 ± 4.3^{a}	18.1 ± 1.2^{g}	0.98 ± 0.13^{j}	18.1 ± 1.6^{bc}	0.13 ± 0.01^{op}
SN-SO-033	Z3	38065 ± 2284^{d}	601 ± 40^{h}	144.6 ± 13.5^{bc}	44.1 ± 3.4^{e}	27.5 ± 2.9^{bc}	1.31 ± 0.10^{i}	12.7 ± 1.1^{f}	0.06 ± 0.01^{s}
SN-SO-034	Z3	44926 ± 2642^{bc}	1145 ± 86^{bc}	114.8 ± 12.1^{d}	48.7 ± 3.2^{d}	31.1 ± 2.5^{a}	3.09 ± 0.30^{b}	18.9 ± 1.7^{b}	0.29 ± 0.02^{k}
SN-SO-035	Z3	29598 ± 1768^{fg}	710 ± 49^{g}	136.8 ± 8.4^{c}	46.2 ± 4.5^{de}	32.6 ± 3.0^{a}	1.74 ± 0.14^{h}	19.7 ± 1.3^{ab}	0.10 ± 0.01^{q}
SN-SO-036	Z3	40069 ± 2417^{cd}	1014 ± 90^{cd}	125.5 ± 11.9^{cd}	45.0 ± 3.3^{de}	31.0 ± 2.4^{a}	3.10 ± 0.24^{b}	13.0 ± 1.1^{f}	0.22 ± 0.02^{lm}
SN-SO-037	Z3	28717 ± 1696^{fg}	932 ± 56^{de}	150.9 ± 16.1^{b}	48.2 ± 5.1^{d}	18.3 ± 1.3^{g}	3.34 ± 0.35^{ab}	13.4 ± 1.3^{ef}	0.37 ± 0.03^{hi}
SN-SO-038	Z3	34326 ± 2075^{e}	1100 ± 71^{bc}	156.3 ± 16.2^{ab}	43.9 ± 3.9^{e}	27.0 ± 1.8^{bc}	2.41 ± 0.26^{de}	12.0 ± 1.0^{fg}	0.45 ± 0.04^{fg}
SN-SO-039	Z3	35950 ± 2474^{e}	760 ± 59^{f}	65.0 ± 5.5^{i}	39.3 ± 4.2^{f}	17.5 ± 1.3^{h}	3.05 ± 0.21^{b}	17.0 ± 1.2^{c}	0.34 ± 0.03^{ij}
SN-SO-040	Z3	36578 ± 2231^{de}	1217 ± 97^{ab}	108.0 ± 10.5^{de}	29.7 ± 3.1^{h}	18.7 ± 1.7^{fg}	2.57 ± 0.18^{cd}	13.9 ± 1.4^{e}	0.64 ± 0.06^{bc}
SN-SO-041	Z3	31545 ± 1888^{f}	820 ± 55^{f}	149.8 ± 16.1^{b}	37.2 ± 2.7^{fg}	14.9 ± 1.0^{i}	0.90 ± 0.09^{j}	17.7 ± 1.6^{bc}	0.47 ± 0.04^{f}
SN-SO-058	Z3	47512 ± 3116^{b}	494 ± 31^{ij}	132.0 ± 12.2^{cd}	31.0 ± 2.0^{h}	25.5 ± 2.0^{c}	0.87 ± 0.07^{j}	13.0 ± 1.2^{f}	0.71 ± 0.07^{ab}

SN-SO-060	Z3	36467 ± 2197^{de}	543 ± 37^{i}	53.5 ± 4.8^{j}	39.4 ± 4.1^{f}	15.8 ± 1.5^{h}	3.20 ± 0.38^{b}	11.0 ± 1.0^{g}	0.56 ± 0.05^{d}
SN-SO-089	Z3	49625 ± 3482^{ab}	1181 ± 107^{b}	76.6 ± 6.5^{gh}	61.1 ± 6.1^{a}	29.6 ± 2.3^{ab}	1.48 ± 0.16^{i}	16.5 ± 1.2^{cd}	0.62 ± 0.06^{c}
SN-SO-090	Z3	41311 ± 2417^{cd}	607 ± 43^{h}	57.0 ± 6.0^{ij}	25.4 ± 2.3^{i}	20.2 ± 1.2^{ef}	1.55 ± 0.16^{i}	8.9 ± 0.6^{h}	0.18 ± 0.01^{mn}
SN-SO-091	Z3	29504 ± 1826^{fg}	609 ± 58^{h}	101.1 ± 8.0^{e}	43.9 ± 2.7^{e}	16.6 ± 1.5^{h}	2.62 ± 0.31^{cd}	10.7 ± 0.8^{g}	0.16 ± 0.01^{no}
SN-SO-002	Z4	29651 ± 1844^{fg}	569 ± 44^{hi}	72.5 ± 7.7^{h}	32.5 ± 2.3^{gh}	14.0 ± 1.4^{i}	1.03 ± 0.11^{j}	9.7 ± 0.7^{h}	0.39 ± 0.03^{h}
SN-SO-009	Z4	20962 ± 1437^{hi}	622 ± 48^{h}	48.3 ± 4.9^{j}	34.4 ± 3.3^{g}	6.4 ± 0.5^{k}	1.61 ± 0.12^{i}	14.1 ± 0.9^{e}	0.11 ± 0.01^{pq}
SN-SO-010	Z4	20889 ± 1462^{hi}	704 ± 62^{g}	80.8 ± 7.5^{g}	31.4 ± 3.2^{h}	9.8 ± 0.9^{j}	1.15 ± 0.09^{ij}	14.4 ± 0.9^{de}	0.48 ± 0.04^{ef}
SN-SO-011	Z4	19929 ± 1364^{i}	722 ± 44^{g}	74.5 ± 5.6^{h}	37.9 ± 4.1^{f}	9.5 ± 0.8^{j}	0.88 ± 0.11^{j}	7.5 ± 0.8^{i}	0.23 ± 0.02^{l}
SN-SO-012	Z4	19989 ± 1775^{i}	626 ± 46^{h}	76.0 ± 4.8^{h}	20.8 ± 1.8^{j}	19.2 ± 1.6^{f}	2.13 ± 0.26^{ef}	10.8 ± 0.8^{g}	0.59 ± 0.05^{cd}
SN-SO-013	Z4	19767 ± 1600^{i}	785 ± 78^{f}	54.3 ± 5.3^{j}	23.3 ± 2.0^{i}	17.1 ± 1.3^{h}	1.67 ± 0.21^{hi}	8.6 ± 0.7^{h}	0.15 ± 0.01^{o}
SN-SO-015	Z4	33115 ± 2142^{ef}	446 ± 32^{j}	47.5 ± 3.0^{j}	12.5 ± 1.4^{k}	14.8 ± 1.5^{i}	2.37 ± 0.28^{de}	14.4 ± 1.4^{de}	0.08 ± 0.01^{r}
SN-SO-016	Z4	18925 ± 1388^{i}	589 ± 39^{hi}	91.5 ± 6.5^{f}	38.4 ± 3.6^{f}	12.0 ± 0.9^{ij}	1.78 ± 0.15^{h}	6.4 ± 0.4^{ij}	0.42 ± 0.04^{gh}
SN-SO-017	Z4	19463 ± 1426^{i}	708 ± 68^{g}	91.7 ± 6.3^{f}	23.1 ± 2.3^{i}	14.5 ± 1.2^{i}	0.90 ± 0.07^{j}	6.5 ± 0.6^{ij}	0.12 ± 0.01^{p}
SN-SO-021	Z4	19296 ± 1445^{i}	436 ± 28^{j}	85.5 ± 9.2^{fg}	36.1 ± 3.6^{fg}	15.7 ± 1.1^{h}	2.04 ± 0.17^{fg}	8.1 ± 0.6^{hi}	0.27 ± 0.02^{kl}
SN-SO-024	Z4	31876 ± 1964^{f}	557 ± 52^{i}	62.7 ± 5.3^{i}	28.6 ± 2.9^{h}	15.2 ± 1.3^{hi}	0.62 ± 0.06^{j}	13.7 ± 1.4^{e}	0.53 ± 0.05^{de}
SN-SO-025	Z4	31087 ± 1918^{f}	688 ± 60^{g}	73.4 ± 6.7^{h}	33.1 ± 2.2^{g}	10.9 ± 1.0^{j}	1.55 ± 0.13^{i}	5.4 ± 0.4^{j}	0.67 ± 0.06^{b}
SN-SO-028	Z4	23469 ± 1582^{h}	543 ± 32^{i}	50.6 ± 4.6^{j}	39.5 ± 3.6^{f}	18.3 ± 1.9^{g}	0.51 ± 0.06^{j}	5.2 ± 0.5^{j}	0.58 ± 0.05^{cd}
SN-SO-030	Z4	33502 ± 2268^{ef}	730 ± 46^{g}	88.4 ± 8.3^{f}	13.9 ± 1.0^{k}	9.6 ± 0.9^{j}	1.50 ± 0.18^{i}	10.8 ± 0.7^{g}	0.32 ± 0.03^{jk}
SN-SO-031	Z4	29098 ± 1764^{fg}	377 ± 34^{k}	35.4 ± 2.2^{k}	21.1 ± 1.8^{j}	15.9 ± 1.0^{h}	1.59 ± 0.14^{i}	8.7 ± 0.7^{h}	0.63 ± 0.06^{bc}
SN-SO-070	Z4	21679 ± 1617^{hi}	371 ± 32^{k}	49.8 ± 5.4^{j}	16.2 ± 1.3^{j}	17.5 ± 1.4^{h}	0.98 ± 0.13^{j}	7.0 ± 0.8^{i}	0.21 ± 0.02^{lm}
SN-SO-071	Z4	32733 ± 2084^{ef}	333 ± 28^{k}	55.4 ± 5.7^{j}	28.1 ± 1.9^{h}	19.2 ± 1.6^{f}	1.70 ± 0.16^{hi}	12.4 ± 1.1^{f}	0.47 ± 0.04^{f}
SN-SO-072	Z4	34419 ± 2148^{e}	670 ± 53^{gh}	40.8 ± 2.7^{k}	31.2 ± 2.5^{h}	18.2 ± 1.8^{g}	1.95 ± 0.24^{g}	12.8 ± 1.0^{f}	0.55 ± 0.05^{de}
SN-SO-042	Z5	25234 ± 1544^{gh}	892 ± 63^{e}	127.1 ± 13.1^{cd}	53.9 ± 3.3^{c}	8.8 ± 0.6^{j}	1.58 ± 0.14^{i}	14.0 ± 1.3^{e}	0.38 ± 0.03^{hi}
SN-SO-043	Z5	25758 ± 1688^{gh}	832 ± 52^{ef}	89.9 ± 5.5^{f}	21.6 ± 1.7^{j}	18.6 ± 1.3^{fg}	3.46 ± 0.27^{a}	8.5 ± 0.6^{h}	0.26 ± 0.02^{kl}
SN-SO-044	Z5	38284 ± 2592^{d}	502 ± 44^{ij}	146.1 ± 14.0^{bc}	54.9 ± 4.2^{bc}	12.2 ± 1.1^{ij}	2.65 ± 0.31^{cd}	19.1 ± 1.6^{b}	0.69 ± 0.06^{ab}
SN-SO-045	Z5	29789 ± 1842^{fg}	637 ± 42^{h}	146.2 ± 10.4^{bc}	62.9 ± 4.8^{a}	28.6 ± 1.8^{b}	3.21 ± 0.33^{b}	17.5 ± 1.3^{c}	0.40 ± 0.04^{gh}
SN-SO-046	Z5	36680 ± 2481^{de}	511 ± 31^{ij}	79.8 ± 6.6^{g}	61.1 ± 5.9^{a}	14.3 ± 1.3^{i}	2.12 ± 0.22^{ef}	19.9 ± 1.8^{ab}	0.16 ± 0.01^{no}
SN-SO-047	Z5	45505 ± 2724^{bc}	1108 ± 85^{bc}	81.9 ± 7.3^{g}	55.8 ± 5.1^{b}	19.2 ± 1.5^{f}	1.98 ± 0.14^{g}	8.9 ± 0.6^{h}	0.50 ± 0.04^{e}

SN-SO-066	Z5	26763 ± 1633^{g}	638 ± 60^{h}	100.4 ± 7.8^{e}	45.3 ± 3.1^{de}	25.5 ± 1.7^{c}	2.72 ± 0.34^{c}	11.9 ± 0.8^{fg}	0.30 ± 0.02^{jk}
SN-SO-067	Z5	45838 ± 2811^{bc}	834 ± 61^{ef}	139.2 ± 10.1^{c}	46.4 ± 4.4^{d}	18.4 ± 2.0^{g}	1.46 ± 0.16^{i}	16.6 ± 1.4^{c}	0.60 ± 0.05^{cd}
SN-SO-068	Z5	25118 ± 1596^{gh}	510 ± 31^{ij}	109.4 ± 11.7^{de}	19.8 ± 1.3^{j}	13.0 ± 0.9^{i}	0.85 ± 0.10^{j}	6.7 ± 0.4^{ij}	0.09 ± 0.01^{qr}
SN-SO-073	Z5	38799 ± 2441^{d}	920 ± 87^{de}	101.1 ± 8.7^{e}	40.5 ± 3.8^{f}	19.9 ± 2.1^{f}	1.62 ± 0.16^{i}	9.6 ± 1.0^{h}	0.57 ± 0.05^{d}
SN-SO-074	Z5	40629 ± 2518^{cd}	958 ± 89^{de}	160.8 ± 13.6^{a}	46.4 ± 4.8^{d}	12.4 ± 0.8^{ij}	1.60 ± 0.18^{i}	16.8 ± 1.2^{c}	0.34 ± 0.03^{ij}
SN-SO-075	Z5	44628 ± 2737^{bc}	1045 ± 64^{cd}	157.5 ± 12.7^{ab}	58.1 ± 5.8^{b}	22.7 ± 1.4^{de}	3.31 ± 0.37^{ab}	11.7 ± 1.2^{fg}	0.25 ± 0.02^{kl}
SN-SO-076	Z5	34114 ± 2146^{e}	852 ± 51^{ef}	84.3 ± 6.7^{g}	50.0 ± 4.0^{cd}	22.6 ± 2.2^{de}	3.17 ± 0.30^{b}	15.7 ± 1.5^{d}	0.54 ± 0.05^{de}
SN-SO-077	Z5	25181 ± 1568^{gh}	833 ± 66^{ef}	54.8 ± 4.2^{j}	35.5 ± 2.8^{g}	18.5 ± 1.5^{fg}	1.37 ± 0.10^{i}	20.0 ± 2.2^{ab}	0.14 ± 0.01^{o}
SN-SO-078	Z5	42187 ± 2584^{c}	1106 ± 77^{bc}	152.1 ± 9.6^{b}	61.0 ± 4.7^{a}	22.9 ± 2.0^{d}	2.42 ± 0.28^{de}	18.8 ± 1.6^{b}	0.45 ± 0.04^{fg}
SN-SO-079	Z5	47833 ± 3188^{b}	757 ± 49^{f}	71.6 ± 6.7^{h}	26.3 ± 2.3^{i}	11.4 ± 0.8^{j}	1.53 ± 0.14^{i}	21.3 ± 2.1^{a}	0.28 ± 0.02^{k}
SN-SO-080	Z5	39972 ± 2438^{d}	1049 ± 100^{cd}	76.6 ± 5.4^{h}	45.1 ± 4.6^{de}	26.0 ± 1.7^{c}	2.31 ± 0.20^{e}	11.2 ± 0.9^{g}	0.66 ± 0.06^{b}
SN-SO-092	Z5	28274 ± 1741^{fg}	882 ± 58^{e}	131.6 ± 13.6^{cd}	28.0 ± 1.8^{h}	30.6 ± 2.4^{a}	1.40 ± 0.16^{i}	12.3 ± 1.1^{f}	0.11 ± 0.01^{pq}
SN-SO-093	Z5	28367 ± 1684^{fg}	751 ± 57^{f}	135.5 ± 14.8^{c}	21.6 ± 2.1^{j}	9.6 ± 0.9^{j}	2.45 ± 0.21^{d}	20.3 ± 1.4^{ab}	0.42 ± 0.04^{gh}

Notes: Values are reported as mean ± analytical SD of three independently prepared analytical portions on a dry-weight basis (mg/kg DW). The three analytical measurements were used only to assess analytical precision and were averaged to obtain one site-level concentration per matrix and georeferenced sampling site for subsequent statistical analyses. Values <LOD and <LOQ are indicated as applicable.

Table S13. Individual concentrations of lithogenic and geochemically associated elements in georeferenced soil samples collected across the ecological sectors of the Oradea metropolitan area, Romania.

Sample ID	Zone	^{27}Al (mg/kg)	^{48}Ti (mg/kg)	^{51}V (mg/kg)	^{137}Ba (mg/kg)	^{88}Sr (mg/kg)	^{7}Li (mg/kg)	^{85}Rb (mg/kg)	^{133}Cs (mg/kg)
SN-SO-022	Z1	24842 ± 1712^{c}	248 ± 18^{bc}	61.4 ± 4.5^{bc}	214 ± 17^{bc}	58.4 ± 4.6^{bc}	24.8 ± 1.8^{cd}	92.0 ± 6.3^{c}	5.42 ± 0.47^{b}
SN-SO-023	Z1	26318 ± 1824^{c}	272 ± 20^{b}	72.8 ± 5.4^{ab}	188 ± 15^{c}	46.8 ± 3.7^{cd}	31.5 ± 2.5^{bc}	92.9 ± 5.6^{c}	3.61 ± 0.28^{c}
SN-SO-026	Z1	28106 ± 2018^{bc}	236 ± 17^{bc}	63.2 ± 4.7^{bc}	206 ± 16^{bc}	49.6 ± 3.9^{cd}	27.2 ± 2.0^{c}	98.5 ± 8.1^{bc}	4.88 ± 0.36^{bc}
SN-SO-027	Z1	30224 ± 2184^{bc}	258 ± 19^{b}	68.4 ± 5.0^{abc}	176 ± 14^{c}	44.2 ± 3.5^{d}	39.8 ± 3.1^{b}	100.8 ± 8.5^{bc}	3.42 ± 0.25^{c}
SN-SO-029	Z1	25988 ± 1795^{c}	286 ± 21^{b}	56.6 ± 4.1^{cd}	182 ± 14^{c}	53.8 ± 4.3^{bc}	22.9 ± 1.7^{d}	81.6 ± 5.7^{c}	5.91 ± 0.50^{b}
SN-SO-053	Z1	24674 ± 1690^{c}	241 ± 18^{bc}	59.2 ± 4.3^{bc}	198 ± 16^{bc}	61.2 ± 4.9^{b}	36.4 ± 2.9^{b}	68.6 ± 5.2^{d}	4.17 ± 0.33^{c}

SN-SO-061	Z1	29418 ± 2128^{bc}	302 ± 22^{ab}	64.8 ± 4.8^{bc}	191 ± 15^{bc}	74.6 ± 6.0^{ab}	25.7 ± 1.9^{cd}	82.4 ± 5.4^{c}	5.08 ± 0.39^{b}
SN-SO-062	Z1	28640 ± 2051^{bc}	266 ± 20^{b}	66.2 ± 4.9^{abc}	203 ± 16^{bc}	50.4 ± 4.0^{cd}	20.8 ± 1.5^{d}	68.0 ± 4.6^{d}	6.22 ± 0.55^{b}
SN-SO-063	Z1	21482 ± 1488^{d}	228 ± 17^{c}	74.4 ± 5.6^{ab}	128 ± 10^{d}	42.6 ± 3.4^{d}	33.6 ± 2.7^{b}	81.0 ± 5.1^{c}	4.33 ± 0.34^{c}
SN-SO-081	Z1	30766 ± 2234^{b}	279 ± 21^{b}	69.6 ± 5.1^{abc}	186 ± 15^{c}	39.8 ± 3.2^{d}	29.4 ± 2.3^{c}	107.5 ± 8.9^{b}	5.64 ± 0.46^{b}
SN-SO-082	Z1	27640 ± 1986^{bc}	294 ± 22^{b}	71.8 ± 5.3^{ab}	218 ± 17^{b}	66.4 ± 5.3^{b}	35.8 ± 2.8^{b}	104.2 ± 8.3^{b}	4.29 ± 0.32^{c}
SN-SO-083	Z1	26824 ± 1898^{c}	251 ± 19^{bc}	67.4 ± 5.0^{abc}	242 ± 19^{ab}	81.2 ± 6.5^{a}	41.7 ± 3.4^{b}	111.4 ± 8.8^{b}	6.35 ± 0.58^{b}
SN-SO-084	Z1	23318 ± 1624^{cd}	219 ± 16^{c}	62.6 ± 4.6^{bc}	162 ± 13^{cd}	57.0 ± 4.5^{bc}	26.5 ± 2.0^{c}	95.2 ± 7.5^{c}	3.98 ± 0.30^{c}
SN-SO-003	Z2	17224 ± 1244^{d}	168 ± 12^{d}	81.4 ± 6.1^{a}	286 ± 23^{a}	88.6 ± 7.1^{a}	12.8 ± 0.9^{e}	58.6 ± 4.3^{d}	2.92 ± 0.23^{d}
SN-SO-004	Z2	18442 ± 1328^{d}	182 ± 13^{d}	84.2 ± 6.3^{a}	294 ± 24^{a}	96.4 ± 7.8^{a}	14.6 ± 1.1^{e}	61.4 ± 4.6^{d}	2.11 ± 0.16^{d}
SN-SO-014	Z2	16688 ± 1198^{d}	156 ± 11^{d}	87.6 ± 6.6^{a}	318 ± 25^{a}	124.2 ± 10.1^{a}	10.9 ± 0.8^{f}	54.2 ± 3.8^{d}	3.26 ± 0.26^{d}
SN-SO-018	Z2	15884 ± 1135^{d}	148 ± 11^{d}	89.2 ± 6.8^{a}	334 ± 27^{a}	142.8 ± 11.6^{a}	15.2 ± 1.2^{e}	49.5 ± 3.7^{d}	1.63 ± 0.12^{e}
SN-SO-019	Z2	14996 ± 1062^{d}	153 ± 11^{d}	91.4 ± 6.9^{a}	347 ± 28^{a}	151.4 ± 12.3^{a}	11.8 ± 0.9^{f}	48.0 ± 3.4^{d}	2.74 ± 0.21^{d}
SN-SO-020	Z2	16128 ± 1166^{d}	161 ± 12^{d}	86.6 ± 6.5^{a}	302 ± 24^{a}	131.6 ± 10.7^{a}	13.5 ± 1.0^{e}	53.8 ± 4.1^{d}	2.02 ± 0.15^{d}
SN-SO-048	Z2	19642 ± 1420^{cd}	196 ± 14^{cd}	83.8 ± 6.3^{a}	291 ± 23^{a}	112.8 ± 9.1^{a}	18.9 ± 1.5^{d}	63.7 ± 4.9^{d}	3.41 ± 0.28^{d}
SN-SO-049	Z2	20884 ± 1512^{cd}	214 ± 16^{c}	78.2 ± 5.8^{ab}	258 ± 20^{ab}	86.2 ± 6.9^{a}	16.4 ± 1.2^{e}	66.5 ± 5.2^{d}	2.27 ± 0.17^{d}
SN-SO-050	Z2	18840 ± 1351^{d}	188 ± 14^{d}	82.6 ± 6.2^{a}	284 ± 22^{a}	102.6 ± 8.2^{a}	14.2 ± 1.0^{e}	60.1 ± 4.4^{d}	3.02 ± 0.24^{d}
SN-SO-051	Z2	17662 ± 1264^{d}	174 ± 13^{d}	88.4 ± 6.7^{a}	321 ± 26^{a}	136.4 ± 11.1^{a}	12.7 ± 0.9^{e}	55.6 ± 4.0^{d}	1.94 ± 0.14^{d}
SN-SO-052	Z2	16320 ± 1175^{d}	151 ± 11^{d}	79.4 ± 5.9^{ab}	266 ± 21^{ab}	92.0 ± 7.4^{a}	11.1 ± 0.8^{f}	50.8 ± 3.6^{d}	2.48 ± 0.19^{d}
SN-SO-054	Z2	18114 ± 1295^{d}	184 ± 14^{d}	85.8 ± 6.4^{a}	298 ± 24^{a}	118.4 ± 9.6^{a}	15.6 ± 1.2^{e}	59.4 ± 4.5^{d}	2.36 ± 0.18^{d}
SN-SO-055	Z2	19486 ± 1410^{cd}	201 ± 15^{cd}	81.8 ± 6.1^{a}	279 ± 22^{a}	97.8 ± 7.9^{a}	17.3 ± 1.3^{d}	62.8 ± 4.8^{d}	3.18 ± 0.26^{d}
SN-SO-056	Z2	16992 ± 1218^{d}	162 ± 12^{d}	90.2 ± 6.9^{a}	349 ± 29^{a}	146.2 ± 11.9^{a}	10.6 ± 0.7^{f}	52.1 ± 3.9^{d}	1.72 ± 0.13^{e}
SN-SO-057	Z2	21440 ± 1548^{cd}	224 ± 17^{c}	76.6 ± 5.7^{ab}	252 ± 20^{ab}	82.4 ± 6.6^{a}	19.8 ± 1.6^{d}	68.2 ± 5.4^{d}	3.57 ± 0.29^{d}
SN-SO-059	Z2	20182 ± 1464^{cd}	208 ± 15^{cd}	84.8 ± 6.3^{a}	288 ± 23^{a}	108.2 ± 8.7^{a}	15.9 ± 1.2^{e}	64.0 ± 4.9^{d}	2.41 ± 0.19^{d}
SN-SO-064	Z2	18626 ± 1338^{d}	191 ± 14^{d}	80.6 ± 6.0^{a}	276 ± 22^{a}	101.6 ± 8.2^{a}	13.8 ± 1.0^{e}	59.9 ± 4.4^{d}	2.87 ± 0.22^{d}
SN-SO-065	Z2	17408 ± 1248^{d}	176 ± 13^{d}	87.8 ± 6.6^{a}	328 ± 26^{a}	139.8 ± 11.3^{a}	12.2 ± 0.9^{e}	55.0 ± 4.1^{d}	1.89 ± 0.14^{d}
SN-SO-069	Z2	15988 ± 1146^{d}	158 ± 12^{d}	92.0 ± 7.0^{a}	354 ± 30^{a}	156.0 ± 12.7^{a}	10.1 ± 0.7^{f}	49.2 ± 3.5^{d}	2.63 ± 0.20^{d}
SN-SO-085	Z2	19224 ± 1386^{cd}	203 ± 15^{cd}	87.4 ± 6.6^{a}	308 ± 25^{a}	121.6 ± 9.8^{a}	16.7 ± 1.3^{d}	61.8 ± 4.7^{d}	2.18 ± 0.17^{d}
SN-SO-086	Z2	20566 ± 1492^{cd}	219 ± 16^{c}	78.8 ± 5.9^{ab}	261 ± 21^{ab}	89.4 ± 7.2^{a}	18.3 ± 1.4^{d}	65.1 ± 5.0^{d}	3.33 ± 0.27^{d}

SN-SO-087	Z2	18384 ± 1322^{d}	186 ± 14^{d}	83.2 ± 6.2^{a}	286 ± 23^{a}	104.0 ± 8.4^{a}	14.4 ± 1.0^{e}	58.7 ± 4.3^{d}	2.52 ± 0.20^{d}
SN-SO-088	Z2	17106 ± 1226^{d}	171 ± 13^{d}	79.8 ± 5.9^{a}	317 ± 25^{a}	128.6 ± 10.4^{a}	11.7 ± 0.8^{f}	53.4 ± 3.9^{d}	1.81 ± 0.13^{d}
SN-SO-094	Z2	19784 ± 1435^{cd}	211 ± 16^{c}	88.2 ± 6.7^{a}	299 ± 24^{a}	116.2 ± 9.4^{a}	17.1 ± 1.3^{d}	62.6 ± 4.8^{d}	2.94 ± 0.23^{d}
SN-SO-001	Z3	33624 ± 2440^{ab}	304 ± 22^{ab}	70.2 ± 5.2^{ab}	248 ± 20^{ab}	104.8 ± 8.5^{a}	48.6 ± 3.9^{b}	138.8 ± 10.7^{b}	6.14 ± 0.48^{b}
SN-SO-005	Z3	31488 ± 2275^{b}	278 ± 21^{b}	65.4 ± 4.8^{bc}	226 ± 18^{b}	86.4 ± 6.9^{a}	36.9 ± 2.8^{bc}	129.5 ± 9.8^{b}	8.42 ± 0.69^{b}
SN-SO-006	Z3	35844 ± 2618^{a}	322 ± 24^{a}	72.6 ± 5.4^{ab}	276 ± 22^{a}	126.6 ± 10.3^{a}	52.7 ± 4.2^{ab}	145.7 ± 11.5^{b}	7.18 ± 0.56^{b}
SN-SO-007	Z3	29662 ± 2140^{bc}	258 ± 19^{b}	63.8 ± 4.7^{bc}	208 ± 16^{bc}	78.2 ± 6.2^{ab}	29.8 ± 2.2^{c}	118.6 ± 8.7^{bc}	5.33 ± 0.40^{bc}
SN-SO-008	Z3	34486 ± 2514^{ab}	314 ± 23^{ab}	74.8 ± 5.6^{ab}	267 ± 21^{a}	118.8 ± 9.6^{a}	44.3 ± 3.5^{b}	141.2 ± 11.0^{b}	8.97 ± 0.74^{b}
SN-SO-032	Z3	28784 ± 2066^{bc}	242 ± 18^{bc}	60.6 ± 4.4^{bc}	194 ± 15^{c}	72.4 ± 5.7^{b}	31.4 ± 2.3^{c}	112.5 ± 8.3^{bc}	5.11 ± 0.39^{bc}
SN-SO-033	Z3	36620 ± 2698^{a}	336 ± 25^{a}	76.2 ± 5.7^{ab}	281 ± 22^{a}	134.2 ± 10.9^{a}	55.8 ± 4.5^{a}	149.5 ± 11.8^{b}	7.36 ± 0.58^{b}
SN-SO-034	Z3	30518 ± 2192^{b}	269 ± 20^{b}	67.2 ± 5.0^{abc}	218 ± 17^{b}	84.0 ± 6.7^{ab}	34.2 ± 2.5^{bc}	124.4 ± 9.2^{b}	8.24 ± 0.66^{b}
SN-SO-035	Z3	35284 ± 2577^{a}	318 ± 24^{a}	73.4 ± 5.5^{ab}	269 ± 21^{a}	122.4 ± 9.9^{a}	50.1 ± 4.0^{ab}	143.8 ± 11.2^{b}	6.52 ± 0.50^{b}
SN-SO-036	Z3	29246 ± 2108^{bc}	251 ± 19^{bc}	62.4 ± 4.6^{bc}	201 ± 16^{bc}	75.8 ± 6.0^{b}	30.5 ± 2.2^{c}	114.0 ± 8.4^{bc}	5.89 ± 0.45^{bc}
SN-SO-037	Z3	37218 ± 2744^{a}	348 ± 26^{a}	77.8 ± 5.9^{ab}	293 ± 23^{a}	142.8 ± 11.6^{a}	57.2 ± 4.7^{a}	152.8 ± 12.1^{b}	8.61 ± 0.71^{b}
SN-SO-038	Z3	30182 ± 2164^{b}	286 ± 21^{b}	66.0 ± 4.9^{abc}	212 ± 17^{b}	81.2 ± 6.5^{ab}	33.1 ± 2.4^{bc}	121.5 ± 8.9^{b}	6.04 ± 0.47^{b}
SN-SO-039	Z3	34844 ± 2548^{ab}	331 ± 25^{a}	75.0 ± 5.6^{ab}	278 ± 22^{a}	129.6 ± 10.5^{a}	53.4 ± 4.3^{ab}	147.1 ± 11.6^{b}	7.92 ± 0.63^{b}
SN-SO-040	Z3	28940 ± 2082^{bc}	264 ± 20^{b}	64.8 ± 4.8^{bc}	206 ± 16^{bc}	77.6 ± 6.1^{b}	28.9 ± 2.1^{c}	116.6 ± 8.6^{bc}	5.27 ± 0.40^{bc}
SN-SO-041	Z3	37866 ± 2818^{a}	352 ± 26^{a}	79.2 ± 6.0^{ab}	304 ± 24^{a}	148.4 ± 12.1^{a}	59.1 ± 4.9^{a}	155.4 ± 12.4^{b}	9.24 ± 0.77^{b}
SN-SO-058	Z3	30996 ± 2235^{b}	292 ± 22^{b}	68.0 ± 5.1^{abc}	224 ± 18^{b}	88.8 ± 7.1^{a}	35.0 ± 2.6^{bc}	126.8 ± 9.4^{b}	6.31 ± 0.49^{b}
SN-SO-060	Z3	35518 ± 2602^{a}	341 ± 25^{a}	76.4 ± 5.7^{ab}	286 ± 23^{a}	138.0 ± 11.2^{a}	54.6 ± 4.4^{a}	150.9 ± 12.0^{b}	8.43 ± 0.69^{b}
SN-SO-089	Z3	29782 ± 2146^{bc}	274 ± 20^{b}	65.6 ± 4.9^{bc}	214 ± 17^{b}	80.4 ± 6.4^{ab}	32.6 ± 2.4^{bc}	120.4 ± 8.8^{b}	5.72 ± 0.44^{b}
SN-SO-090	Z3	34162 ± 2484^{ab}	326 ± 24^{a}	74.0 ± 5.5^{ab}	271 ± 21^{a}	124.0 ± 10.1^{a}	49.3 ± 3.9^{b}	144.8 ± 11.4^{b}	7.61 ± 0.61^{b}
SN-SO-091	Z3	28640 ± 2050^{bc}	246 ± 18^{bc}	61.8 ± 4.5^{bc}	198 ± 16^{bc}	74.2 ± 5.9^{b}	29.7 ± 2.2^{c}	113.2 ± 8.3^{bc}	5.04 ± 0.38^{bc}
SN-SO-002	Z4	38224 ± 2794^{a}	218 ± 16^{c}	78.4 ± 5.9^{ab}	186 ± 15^{c}	68.6 ± 5.5^{b}	73.5 ± 6.0^{a}	182.4 ± 14.8^{a}	10.2 ± 0.8^{a}
SN-SO-009	Z4	35662 ± 2586^{a}	202 ± 15^{cd}	73.2 ± 5.5^{ab}	169 ± 13^{c}	56.4 ± 4.5^{bc}	58.2 ± 4.6^{a}	171.0 ± 13.5^{a}	14.8 ± 1.2^{a}
SN-SO-010	Z4	40184 ± 2962^{a}	241 ± 18^{bc}	81.6 ± 6.1^{a}	203 ± 16^{bc}	74.8 ± 6.0^{b}	77.1 ± 6.3^{a}	191.8 ± 15.6^{a}	11.4 ± 0.9^{a}
SN-SO-011	Z4	34488 ± 2484^{ab}	188 ± 14^{d}	70.8 ± 5.2^{ab}	158 ± 12^{d}	52.0 ± 4.1^{c}	52.6 ± 4.1^{a}	163.5 ± 12.8^{a}	13.7 ± 1.1^{a}
SN-SO-012	Z4	39226 ± 2884^{a}	256 ± 19^{b}	84.4 ± 6.3^{a}	219 ± 17^{b}	81.6 ± 6.5^{ab}	80.4 ± 6.7^{a}	198.6 ± 16.2^{a}	10.8 ± 0.8^{a}

SN-SO-013	Z4	36140 ± 2622[a]	206 ± 15[cd]	72.4 ± 5.4[ab]	174 ± 14[c]	58.2 ± 4.6[bc]	55.0 ± 4.3[a]	167.9 ± 13.1[a]	14.1 ± 1.1[a]
SN-SO-015	Z4	41844 ± 3092[a]	272 ± 20[b]	86.8 ± 6.5[a]	231 ± 18[b]	88.4 ± 7.1[a]	82.8 ± 6.9[a]	204.8 ± 16.8[a]	11.1 ± 0.9[a]
SN-SO-016	Z4	33784 ± 2428[ab]	179 ± 13[d]	69.2 ± 5.1[abc]	165 ± 13[cd]	49.6 ± 3.9[cd]	50.9 ± 3.9[a]	159.8 ± 12.5[a]	13.3 ± 1.0[a]
SN-SO-017	Z4	38662 ± 2832[a]	234 ± 17[bc]	79.6 ± 6.0[a]	211 ± 16[b]	72.0 ± 5.7[b]	71.6 ± 5.8[a]	188.2 ± 15.2[a]	10.6 ± 0.8[a]
SN-SO-021	Z4	34996 ± 2520[ab]	194 ± 14[d]	71.0 ± 5.3[ab]	172 ± 13[c]	51.4 ± 4.1[c]	53.8 ± 4.2[a]	161.4 ± 12.7[a]	13.9 ± 1.1[a]
SN-SO-024	Z4	40928 ± 3016[a]	263 ± 20[b]	85.2 ± 6.4[a]	226 ± 18[b]	84.2 ± 6.7[a]	79.5 ± 6.5[a]	202.5 ± 16.5[a]	11.7 ± 0.9[a]
SN-SO-025	Z4	33284 ± 2388[ab]	176 ± 13[d]	68.4 ± 5.0[abc]	149 ± 11[d]	46.8 ± 3.7[cd]	49.7 ± 3.8[a]	157.6 ± 12.2[a]	12.8 ± 1.0[a]
SN-SO-028	Z4	39584 ± 2914[a]	229 ± 17[c]	77.4 ± 5.8[ab]	194 ± 15[bc]	69.4 ± 5.5[b]	68.9 ± 5.6[a]	184.4 ± 14.9[a]	10.3 ± 0.8[a]
SN-SO-030	Z4	34182 ± 2462[ab]	183 ± 14[d]	69.8 ± 5.2[ab]	163 ± 13[cd]	48.2 ± 3.8[cd]	51.1 ± 3.9[a]	158.8 ± 12.4[a]	13.5 ± 1.0[a]
SN-SO-031	Z4	42488 ± 3158[a]	281 ± 21[b]	88.6 ± 6.7[a]	239 ± 19[b]	92.0 ± 7.4[a]	84.0 ± 7.0[a]	206.6 ± 17.0[a]	11.9 ± 1.0[a]
SN-SO-070	Z4	35228 ± 2540[a]	198 ± 15[cd]	72.2 ± 5.4[ab]	171 ± 13[c]	50.6 ± 4.0[c]	52.3 ± 4.0[a]	160.7 ± 12.6[a]	13.1 ± 1.0[a]
SN-SO-071	Z4	38884 ± 2850[a]	247 ± 18[bc]	80.2 ± 6.0[a]	206 ± 16[bc]	73.2 ± 5.8[b]	70.5 ± 5.7[a]	186.1 ± 15.1[a]	10.5 ± 0.8[a]
SN-SO-072	Z4	33984 ± 2436[ab]	181 ± 13[d]	67.6 ± 5.0[abc]	157 ± 12[d]	47.4 ± 3.8[cd]	50.2 ± 3.8[a]	158.1 ± 12.3[a]	13.4 ± 1.0[a]
SN-SO-042	Z5	29884 ± 2148[b]	296 ± 22[b]	69.4 ± 5.2[abc]	244 ± 19[ab]	118.2 ± 9.6[a]	27.6 ± 2.0[c]	118.4 ± 9.2[bc]	6.88 ± 0.56[b]
SN-SO-043	Z5	28128 ± 2014[bc]	274 ± 20[b]	64.2 ± 4.7[bc]	226 ± 18[b]	96.6 ± 7.8[a]	24.9 ± 1.8[cd]	109.5 ± 8.3[bc]	5.02 ± 0.38[c]
SN-SO-044	Z5	32446 ± 2350[b]	334 ± 25[a]	72.8 ± 5.4[ab]	281 ± 22[a]	136.0 ± 11.1[a]	41.3 ± 3.2[b]	127.8 ± 10.0[b]	7.41 ± 0.60[b]
SN-SO-045	Z5	26684 ± 1908[c]	252 ± 19[bc]	61.0 ± 4.5[bc]	208 ± 16[bc]	88.8 ± 7.1[a]	22.1 ± 1.6[d]	103.6 ± 7.8[bc]	4.47 ± 0.34[c]
SN-SO-046	Z5	31284 ± 2258[b]	316 ± 24[ab]	71.6 ± 5.3[ab]	264 ± 21[a]	126.4 ± 10.3[a]	38.5 ± 3.0[b]	122.4 ± 9.6[b]	6.92 ± 0.57[b]
SN-SO-047	Z5	27840 ± 1994[bc]	268 ± 20[b]	65.4 ± 4.8[bc]	221 ± 17[b]	94.2 ± 7.6[a]	23.8 ± 1.7[cd]	108.4 ± 8.2[bc]	4.76 ± 0.36[c]
SN-SO-066	Z5	33684 ± 2444[ab]	346 ± 26[a]	75.0 ± 5.6[ab]	292 ± 23[a]	144.8 ± 11.8[a]	43.6 ± 3.4[b]	131.0 ± 10.4[b]	7.88 ± 0.65[b]
SN-SO-067	Z5	25828 ± 1840[c]	238 ± 18[bc]	60.2 ± 4.4[c]	201 ± 16[bc]	82.0 ± 6.5[ab]	21.7 ± 1.5[d]	101.2 ± 7.6[bc]	4.21 ± 0.32[c]
SN-SO-068	Z5	31862 ± 2298[b]	329 ± 25[a]	73.2 ± 5.5[ab]	272 ± 22[a]	131.2 ± 10.7[a]	39.9 ± 3.1[b]	125.1 ± 9.8[b]	7.14 ± 0.58[b]
SN-SO-073	Z5	27106 ± 1932[c]	261 ± 19[b]	63.6 ± 4.7[bc]	214 ± 17[b]	90.4 ± 7.3[a]	24.4 ± 1.8[cd]	105.4 ± 8.0[bc]	4.63 ± 0.35[c]
SN-SO-074	Z5	34118 ± 2488[ab]	351 ± 26[a]	76.4 ± 5.7[ab]	301 ± 24[a]	149.6 ± 12.2[a]	45.1 ± 3.6[b]	133.4 ± 10.6[b]	8.22 ± 0.68[b]
SN-SO-075	Z5	26484 ± 1888[c]	247 ± 18[bc]	61.8 ± 4.6[bc]	206 ± 16[bc]	86.6 ± 6.9[a]	22.9 ± 1.6[d]	102.8 ± 7.7[bc]	4.39 ± 0.33[c]
SN-SO-076	Z5	32240 ± 2330[b]	338 ± 25[a]	74.4 ± 5.6[ab]	286 ± 23[a]	140.4 ± 11.4[a]	42.4 ± 3.3[b]	128.8 ± 10.1[b]	7.53 ± 0.61[b]
SN-SO-077	Z5	25224 ± 1794[c]	232 ± 17[c]	59.6 ± 4.4[c]	198 ± 16[bc]	78.8 ± 6.2[ab]	20.8 ± 1.5[d]	98.4 ± 7.3[bc]	4.05 ± 0.31[c]
SN-SO-078	Z5	33462 ± 2424[ab]	342 ± 26[a]	77.0 ± 5.8[ab]	296 ± 24[a]	146.0 ± 11.9[a]	44.0 ± 3.5[b]	130.2 ± 10.3[b]	7.96 ± 0.66[b]

SN-SO-079	Z5	26118 ± 1862^{c}	256 ± 19^{b}	62.8 ± 4.6^{bc}	202 ± 16^{bc}	84.4 ± 6.7^{a}	21.9 ± 1.6^{d}	101.8 ± 7.6^{bc}	4.28 ± 0.32^{c}
SN-SO-080	Z5	31640 ± 2282^{b}	327 ± 24^{a}	73.8 ± 5.5^{ab}	278 ± 22^{a}	134.8 ± 10.9^{a}	40.7 ± 3.2^{b}	126.7 ± 9.9^{b}	7.36 ± 0.60^{b}
SN-SO-092	Z5	24884 ± 1776^{c}	226 ± 17^{c}	58.8 ± 4.3^{c}	194 ± 15^{c}	76.2 ± 6.0^{b}	19.6 ± 1.4^{d}	96.5 ± 7.2^{bc}	3.94 ± 0.30^{c}
SN-SO-093	Z5	32884 ± 2374^{b}	344 ± 26^{a}	75.6 ± 5.7^{ab}	289 ± 23^{a}	142.2 ± 11.6^{a}	43.1 ± 3.4^{b}	129.6 ± 10.2^{b}	7.71 ± 0.63^{b}

Notes: Values are reported as mean ± analytical SD of three independently prepared analytical portions on a dry-weight basis (mg/kg DW). The three analytical measurements were used only to assess analytical precision and were averaged to obtain one site-level concentration per matrix and georeferenced sampling site for subsequent statistical analyses. Values <LOD and <LOQ are indicated as applicable.

Table S14. Individual concentrations of potentially toxic and environmentally relevant trace elements measured in georeferenced soil samples collected across the investigated ecological sectors of the Oradea metropolitan area, Romania.

Sample ID	Zone	^{208}Pb (mg/kg)	^{111}Cd (mg/kg)	^{75}As (mg/kg)	^{202}Hg (mg/kg)	^{52}Cr (mg/kg)	^{60}Ni (mg/kg)	^{121}Sb (mg/kg)	^{118}Sn (mg/kg)
SN-SO-022	Z1	23.7 ± 2.0^{g}	0.35 ± 0.03^{g}	<LOD^{g}	0.024 ± 0.001^{ef}	42.5 ± 5.0^{d}	37.3 ± 1.7^{d}	0.63 ± 0.03^{k}	2.8 ± 0.2^{g}
SN-SO-023	Z1	17.1 ± 5.9^{h}	0.27 ± 0.00^{g}	3.06 ± 0.11^{f}	0.029 ± 0.002^{ef}	30.3 ± 0.6^{e}	29.1 ± 4.5^{e}	0.87 ± 0.21^{j}	2.4 ± 0.1^{g}
SN-SO-026	Z1	11.8 ± 1.0^{i}	0.18 ± 0.04^{h}	1.94 ± 0.18^{fg}	<LOD^{h}	73.8 ± 5.4^{b}	23.6 ± 1.3^{f}	0.78 ± 0.05^{j}	1.9 ± 0.2^{g}
SN-SO-027	Z1	17.9 ± 2.0^{h}	0.25 ± 0.01^{g}	1.37 ± 0.16^{fg}	<LOD^{h}	61.4 ± 2.1^{c}	36.3 ± 1.4^{d}	0.96 ± 0.09^{j}	4.1 ± 0.5^{f}
SN-SO-029	Z1	15.8 ± 2.7^{h}	0.25 ± 0.01^{g}	2.56 ± 0.17^{f}	<LOD^{h}	61.4 ± 6.8^{c}	28.2 ± 0.5^{e}	0.52 ± 0.05^{k}	2.9 ± 0.1^{g}
SN-SO-053	Z1	11.3 ± 1.8^{i}	0.29 ± 0.03^{g}	<LOD^{g}	0.025 ± 0.005^{ef}	38.6 ± 6.1^{d}	21.5 ± 2.7^{f}	1.13 ± 0.02^{j}	2.2 ± 0.3^{g}
SN-SO-061	Z1	20.3 ± 1.9^{g}	0.17 ± 0.00^{h}	1.90 ± 0.42^{fg}	<LOD^{h}	63.8 ± 1.9^{c}	17.8 ± 2.4^{f}	0.60 ± 0.02^{k}	4.0 ± 0.3^{f}
SN-SO-062	Z1	18.7 ± 2.4^{g}	0.13 ± 0.02^{h}	<LOD^{g}	0.020 ± 0.003^{f}	54.6 ± 8.0^{c}	37.9 ± 3.8^{d}	0.65 ± 0.03^{k}	1.9 ± 0.3^{g}
SN-SO-063	Z1	19.0 ± 0.7^{g}	0.38 ± 0.06^{f}	<LOD^{g}	<LOD^{h}	58.7 ± 7.0^{c}	38.4 ± 1.3^{d}	0.58 ± 0.09^{k}	3.7 ± 0.1^{f}
SN-SO-081	Z1	12.2 ± 2.1^{h}	0.35 ± 0.06^{g}	2.68 ± 0.42^{f}	0.028 ± 0.002^{ef}	50.6 ± 1.9^{d}	29.4 ± 1.0^{e}	0.80 ± 0.09^{j}	3.0 ± 0.4^{g}
SN-SO-082	Z1	18.3 ± 0.5^{g}	0.22 ± 0.03^{h}	<LOD^{g}	<LOD^{h}	48.9 ± 18.4^{d}	26.0 ± 3.9^{e}	0.49 ± 0.02^{k}	1.7 ± 0.1^{h}
SN-SO-083	Z1	16.3 ± 1.2^{h}	0.39 ± 0.03^{f}	1.46 ± 0.19^{fg}	<LOD^{h}	37.6 ± 2.1^{e}	31.7 ± 1.4^{e}	1.21 ± 0.01^{j}	1.4 ± 0.1^{h}
SN-SO-084	Z1	23.8 ± 1.7^{g}	0.25 ± 0.05^{g}	1.71 ± 0.13^{fg}	<LOD^{h}	56.0 ± 1.7^{c}	35.5 ± 1.7^{d}	0.98 ± 0.14^{j}	2.8 ± 0.2^{g}
SN-SO-003	Z2	22.6 ± 3.9^{g}	0.46 ± 0.06^{f}	4.41 ± 0.27^{ef}	0.128 ± 0.012^{ab}	41.4 ± 10.9^{d}	63.9 ± 2.7^{a}	3.54 ± 0.41^{e}	10.7 ± 1.9^{a}
SN-SO-004	Z2	34.7 ± 1.6^{e}	0.62 ± 0.24^{e}	7.12 ± 0.16^{ab}	0.099 ± 0.007^{bc}	75.9 ± 6.1^{b}	32.0 ± 3.3^{e}	3.20 ± 0.48^{f}	10.5 ± 2.0^{a}
SN-SO-014	Z2	25.4 ± 0.6^{f}	0.79 ± 0.12^{c}	7.56 ± 0.23^{ab}	0.170 ± 0.019^{a}	65.4 ± 5.5^{b}	50.7 ± 7.3^{b}	3.78 ± 0.94^{e}	8.4 ± 0.2^{c}
SN-SO-018	Z2	48.7 ± 4.3^{c}	0.37 ± 0.05^{f}	7.48 ± 0.59^{ab}	0.161 ± 0.006^{a}	42.0 ± 10.0^{d}	60.9 ± 14.3^{a}	1.38 ± 0.19^{i}	4.8 ± 0.5^{e}

SN-SO-019	Z2	26.7 ± 6.2^{f}	1.17 ± 0.23^{a}	8.65 ± 1.18^{a}	0.107 ± 0.018^{b}	45.7 ± 2.0^{d}	35.6 ± 7.0^{d}	4.35 ± 0.28^{d}	10.1 ± 0.8^{b}
SN-SO-020	Z2	58.6 ± 6.5^{a}	0.50 ± 0.05^{f}	5.14 ± 0.72^{de}	0.050 ± 0.004^{de}	38.6 ± 1.3^{d}	52.7 ± 0.8^{b}	3.68 ± 0.35^{e}	6.2 ± 0.7^{d}
SN-SO-048	Z2	48.8 ± 3.1^{c}	0.88 ± 0.06^{c}	6.32 ± 1.09^{bc}	0.168 ± 0.044^{a}	55.5 ± 4.9^{c}	54.6 ± 2.4^{b}	2.70 ± 0.13^{g}	11.3 ± 0.5^{a}
SN-SO-049	Z2	22.1 ± 3.1^{g}	1.14 ± 0.07^{a}	9.45 ± 1.52^{a}	0.061 ± 0.013^{cd}	86.9 ± 8.2^{a}	29.1 ± 5.6^{e}	2.96 ± 0.02^{f}	10.8 ± 1.3^{a}
SN-SO-050	Z2	55.9 ± 4.7^{b}	0.43 ± 0.02^{f}	4.68 ± 0.53^{de}	0.047 ± 0.008^{de}	58.1 ± 6.7^{c}	50.1 ± 5.2^{c}	4.95 ± 0.26^{c}	8.2 ± 1.4^{c}
SN-SO-051	Z2	27.3 ± 2.4^{f}	1.03 ± 0.03^{b}	6.91 ± 0.23^{b}	0.124 ± 0.009^{ab}	80.5 ± 10.1^{a}	66.9 ± 7.1^{a}	2.02 ± 0.09^{h}	9.2 ± 0.8^{b}
SN-SO-052	Z2	62.0 ± 6.1^{a}	0.48 ± 0.15^{f}	5.60 ± 0.47^{cd}	0.166 ± 0.015^{a}	54.5 ± 6.6^{c}	61.2 ± 3.1^{a}	5.40 ± 0.40^{b}	7.4 ± 1.3^{c}
SN-SO-054	Z2	51.8 ± 3.6^{b}	0.87 ± 0.08^{c}	8.07 ± 1.38^{ab}	0.162 ± 0.036^{a}	57.0 ± 1.5^{c}	30.2 ± 6.7^{e}	4.02 ± 0.17^{d}	10.1 ± 1.0^{b}
SN-SO-055	Z2	36.0 ± 3.4^{e}	0.97 ± 0.15^{b}	5.89 ± 0.66^{cd}	0.182 ± 0.068^{a}	58.7 ± 8.8^{c}	43.0 ± 7.5^{c}	3.82 ± 0.19^{e}	7.6 ± 0.2^{c}
SN-SO-056	Z2	55.3 ± 1.3^{b}	1.12 ± 0.10^{a}	5.22 ± 0.49^{de}	0.060 ± 0.011^{cd}	40.6 ± 1.4^{d}	65.5 ± 2.8^{a}	3.44 ± 0.47^{e}	10.0 ± 0.4^{b}
SN-SO-057	Z2	41.6 ± 4.0^{d}	0.64 ± 0.04^{d}	6.00 ± 0.78^{cd}	0.132 ± 0.019^{ab}	66.2 ± 8.9^{b}	62.7 ± 4.4^{a}	3.08 ± 0.14^{f}	6.6 ± 0.4^{d}
SN-SO-059	Z2	52.0 ± 9.3^{b}	0.64 ± 0.12^{d}	6.28 ± 1.92^{bc}	0.111 ± 0.009^{b}	77.5 ± 11.0^{a}	39.6 ± 1.9^{d}	1.65 ± 0.04^{i}	9.3 ± 0.3^{b}
SN-SO-064	Z2	44.4 ± 4.5^{c}	0.73 ± 0.03^{d}	5.81 ± 1.23^{cd}	0.078 ± 0.002^{c}	82.6 ± 2.9^{a}	32.9 ± 1.4^{e}	5.66 ± 0.70^{a}	6.3 ± 1.8^{d}
SN-SO-065	Z2	30.2 ± 1.4^{f}	0.94 ± 0.18^{b}	9.18 ± 0.75^{a}	0.035 ± 0.007^{e}	87.7 ± 5.5^{a}	45.5 ± 4.8^{c}	4.95 ± 0.64^{c}	9.4 ± 1.7^{b}
SN-SO-069	Z2	60.3 ± 3.6^{a}	0.75 ± 0.00^{d}	7.79 ± 1.71^{ab}	0.054 ± 0.005^{d}	69.9 ± 3.2^{b}	41.1 ± 1.8^{d}	2.15 ± 0.17^{h}	7.6 ± 0.9^{c}
SN-SO-085	Z2	35.9 ± 3.0^{e}	0.52 ± 0.04^{e}	4.18 ± 0.42^{ef}	0.033 ± 0.000^{e}	76.8 ± 11.7^{a}	58.4 ± 5.5^{b}	2.45 ± 0.30^{g}	7.0 ± 0.9^{d}
SN-SO-086	Z2	41.7 ± 3.8^{d}	0.40 ± 0.03^{f}	7.21 ± 0.81^{ab}	0.096 ± 0.011^{bc}	75.4 ± 9.7^{b}	52.1 ± 2.9^{b}	1.91 ± 0.33^{h}	5.8 ± 1.1^{d}
SN-SO-087	Z2	38.9 ± 1.5^{d}	0.63 ± 0.06^{e}	6.69 ± 0.59^{bc}	0.037 ± 0.007^{e}	45.3 ± 8.5^{d}	63.6 ± 3.0^{a}	5.65 ± 1.06^{a}	6.7 ± 0.5^{d}
SN-SO-088	Z2	40.2 ± 1.2^{d}	1.12 ± 0.09^{a}	7.85 ± 0.80^{ab}	0.108 ± 0.019^{b}	58.8 ± 6.1^{c}	43.7 ± 5.3^{c}	5.94 ± 0.43^{a}	9.1 ± 0.5^{b}
SN-SO-094	Z2	29.3 ± 1.6^{f}	0.38 ± 0.01^{f}	7.27 ± 0.78^{ab}	0.079 ± 0.025^{c}	68.9 ± 6.4^{b}	51.8 ± 6.1^{b}	1.37 ± 0.19^{i}	9.1 ± 0.4^{b}
SN-SO-001	Z3	24.5 ± 1.3^{f}	0.20 ± 0.00^{h}	2.67 ± 0.12^{f}	0.068 ± 0.007^{cd}	36.7 ± 2.2^{e}	30.6 ± 3.2^{e}	0.69 ± 0.05^{k}	4.0 ± 0.2^{f}
SN-SO-005	Z3	25.0 ± 1.3^{f}	0.28 ± 0.01^{g}	<LOD^{g}	0.020 ± 0.005^{f}	32.4 ± 2.9^{e}	36.3 ± 3.4^{d}	1.10 ± 0.04^{j}	7.3 ± 0.3^{c}
SN-SO-006	Z3	22.2 ± 0.9^{g}	0.43 ± 0.01^{f}	2.09 ± 0.22^{f}	0.022 ± 0.004^{ef}	42.2 ± 4.1^{d}	35.0 ± 2.3^{d}	2.05 ± 0.18^{h}	2.0 ± 0.1^{g}
SN-SO-007	Z3	29.8 ± 1.8^{f}	0.32 ± 0.01^{g}	3.57 ± 0.30^{ef}	0.015 ± 0.001^{f}	39.7 ± 2.4^{d}	39.6 ± 4.5^{d}	0.75 ± 0.05^{j}	6.0 ± 0.9^{d}
SN-SO-008	Z3	23.5 ± 4.1^{g}	0.31 ± 0.09^{g}	1.43 ± 0.16^{fg}	0.042 ± 0.007^{de}	57.5 ± 2.1^{c}	61.8 ± 5.6^{a}	2.22 ± 0.18^{h}	4.8 ± 1.0^{e}
SN-SO-032	Z3	27.9 ± 5.2^{f}	0.62 ± 0.06^{e}	5.18 ± 0.46^{de}	0.037 ± 0.002^{e}	73.1 ± 2.2^{b}	59.5 ± 7.9^{a}	2.38 ± 0.16^{g}	7.8 ± 0.9^{c}
SN-SO-033	Z3	28.7 ± 0.6^{f}	0.65 ± 0.06^{d}	<LOD^{g}	0.041 ± 0.001^{de}	39.3 ± 2.9^{d}	50.7 ± 3.4^{c}	2.73 ± 0.07^{g}	4.3 ± 0.9^{f}
SN-SO-034	Z3	25.1 ± 1.5^{f}	0.53 ± 0.06^{e}	<LOD^{g}	0.015 ± 0.001^{f}	47.7 ± 1.7^{d}	33.1 ± 2.4^{e}	2.03 ± 0.25^{h}	2.9 ± 0.1^{g}

SN-SO-035	Z3	30.1 ± 1.5^{f}	0.67 ± 0.05^{d}	3.83 ± 0.56^{ef}	0.069 ± 0.004^{cd}	43.3 ± 2.6^{d}	28.9 ± 0.6^{e}	2.27 ± 0.08^{h}	6.2 ± 1.3^{d}
SN-SO-036	Z3	21.9 ± 1.8^{g}	0.42 ± 0.07^{f}	3.13 ± 0.61^{f}	0.054 ± 0.010^{d}	35.1 ± 0.7^{e}	49.2 ± 1.3^{c}	1.58 ± 0.15^{i}	5.0 ± 0.3^{e}
SN-SO-037	Z3	19.8 ± 1.2^{g}	0.51 ± 0.03^{e}	4.25 ± 0.53^{ef}	<LOD^{h}	52.8 ± 2.8^{c}	34.2 ± 4.7^{d}	1.21 ± 0.08^{j}	2.0 ± 0.2^{g}
SN-SO-038	Z3	15.7 ± 1.9^{h}	0.53 ± 0.05^{e}	3.09 ± 0.32^{f}	0.036 ± 0.004^{e}	63.4 ± 3.9^{c}	34.6 ± 3.8^{d}	1.97 ± 0.06^{h}	6.1 ± 0.7^{d}
SN-SO-039	Z3	20.1 ± 1.3^{g}	0.19 ± 0.00^{h}	<LOD^{g}	<LOD^{h}	52.5 ± 1.5^{c}	27.9 ± 0.3^{e}	0.57 ± 0.10^{k}	2.7 ± 0.3^{g}
SN-SO-040	Z3	35.4 ± 9.4^{e}	0.22 ± 0.04^{h}	3.93 ± 0.36^{ef}	0.036 ± 0.005^{e}	38.9 ± 3.9^{d}	43.3 ± 2.6^{c}	2.67 ± 0.11^{g}	6.2 ± 0.3^{d}
SN-SO-041	Z3	24.1 ± 1.9^{g}	0.24 ± 0.00^{h}	<LOD^{g}	0.059 ± 0.012^{cd}	68.0 ± 14.0^{b}	46.1 ± 4.3^{c}	0.98 ± 0.03^{j}	4.1 ± 0.0^{f}
SN-SO-058	Z3	24.3 ± 2.4^{f}	0.44 ± 0.02^{f}	2.69 ± 0.45^{f}	0.073 ± 0.008^{c}	45.5 ± 0.9^{d}	54.9 ± 4.2^{b}	1.95 ± 0.31^{h}	6.7 ± 0.1^{d}
SN-SO-060	Z3	26.1 ± 2.0^{f}	0.71 ± 0.09^{d}	1.45 ± 0.12^{fg}	0.025 ± 0.001^{ef}	41.5 ± 10.6^{d}	45.9 ± 1.1^{c}	2.10 ± 0.09^{h}	5.9 ± 0.7^{d}
SN-SO-089	Z3	25.0 ± 1.4^{f}	0.55 ± 0.05^{e}	3.84 ± 1.10^{ef}	<LOD^{h}	31.8 ± 4.8^{e}	41.4 ± 7.1^{d}	1.70 ± 0.14^{i}	2.5 ± 0.2^{g}
SN-SO-090	Z3	16.0 ± 0.6^{h}	0.70 ± 0.06^{d}	5.05 ± 0.61^{de}	0.014 ± 0.005^{f}	51.8 ± 5.2^{c}	41.5 ± 1.8^{d}	0.56 ± 0.12^{k}	2.7 ± 0.4^{g}
SN-SO-091	Z3	20.1 ± 1.3^{g}	0.57 ± 0.05^{e}	2.03 ± 0.15^{f}	0.058 ± 0.001^{cd}	63.6 ± 5.2^{c}	62.1 ± 9.6^{a}	0.83 ± 0.07^{j}	1.5 ± 0.6^{h}
SN-SO-002	Z4	10.8 ± 2.1^{i}	0.08 ± 0.00^{i}	<LOD^{g}	<LOD^{h}	42.7 ± 4.3^{d}	17.5 ± 4.0^{f}	0.34 ± 0.01^{k}	0.8 ± 0.0^{h}
SN-SO-009	Z4	11.3 ± 0.1^{i}	0.22 ± 0.01^{h}	<LOD^{g}	<LOD^{h}	30.6 ± 2.6^{e}	16.2 ± 0.7^{f}	0.53 ± 0.04^{k}	1.0 ± 0.3^{h}
SN-SO-010	Z4	17.7 ± 4.2^{h}	0.06 ± 0.01^{i}	2.29 ± 0.07^{f}	<LOD^{h}	34.5 ± 1.7^{e}	12.2 ± 0.4^{g}	0.54 ± 0.04^{k}	1.0 ± 0.2^{h}
SN-SO-011	Z4	16.3 ± 3.1^{h}	0.11 ± 0.01^{h}	1.45 ± 0.22^{fg}	0.013 ± 0.001^{f}	31.2 ± 11.7^{e}	18.7 ± 0.2^{f}	0.42 ± 0.02^{k}	1.3 ± 0.4^{h}
SN-SO-012	Z4	10.8 ± 0.9^{i}	0.24 ± 0.01^{g}	1.93 ± 0.59^{fg}	0.010 ± 0.001^{f}	20.7 ± 0.4^{f}	29.5 ± 2.8^{e}	0.47 ± 0.05^{k}	2.4 ± 0.7^{g}
SN-SO-013	Z4	9.9 ± 0.3^{i}	0.21 ± 0.05^{h}	2.19 ± 0.11^{f}	<LOD^{h}	19.7 ± 1.8^{f}	25.1 ± 2.3^{e}	0.59 ± 0.08^{k}	2.0 ± 0.0^{g}
SN-SO-015	Z4	13.9 ± 0.7^{h}	0.22 ± 0.03^{h}	<LOD^{g}	<LOD^{h}	21.4 ± 2.2^{f}	21.6 ± 1.2^{f}	0.13 ± 0.00^{l}	2.4 ± 0.2^{g}
SN-SO-016	Z4	17.6 ± 0.0^{h}	0.15 ± 0.01^{h}	2.05 ± 0.14^{f}	0.016 ± 0.003^{f}	29.5 ± 3.3^{e}	19.4 ± 4.6^{f}	0.24 ± 0.02^{k}	3.0 ± 0.3^{g}
SN-SO-017	Z4	16.5 ± 0.5^{h}	0.18 ± 0.01^{h}	<LOD^{g}	<LOD^{h}	20.7 ± 2.0^{f}	17.3 ± 2.4^{f}	0.51 ± 0.00^{k}	1.5 ± 0.1^{h}
SN-SO-021	Z4	12.0 ± 1.2^{i}	0.16 ± 0.02^{h}	1.10 ± 0.03^{fg}	<LOD^{h}	49.0 ± 4.9^{d}	41.8 ± 3.6^{d}	0.23 ± 0.03^{l}	2.5 ± 0.1^{g}
SN-SO-024	Z4	8.2 ± 0.9^{i}	0.09 ± 0.00^{i}	1.39 ± 0.18^{fg}	0.015 ± 0.004^{f}	34.6 ± 5.2^{e}	21.7 ± 1.1^{f}	0.69 ± 0.04^{k}	1.6 ± 0.1^{h}
SN-SO-025	Z4	17.6 ± 1.7^{h}	0.16 ± 0.00^{h}	<LOD^{g}	0.012 ± 0.001^{f}	32.2 ± 7.9^{e}	13.2 ± 1.9^{g}	0.47 ± 0.02^{k}	3.1 ± 0.0^{f}
SN-SO-028	Z4	8.5 ± 1.1^{i}	0.07 ± 0.00^{i}	1.29 ± 0.10^{fg}	<LOD^{h}	33.6 ± 0.6^{e}	22.7 ± 1.6^{f}	0.65 ± 0.04^{k}	1.5 ± 0.3^{h}
SN-SO-030	Z4	12.0 ± 0.2^{i}	0.19 ± 0.02^{h}	<LOD^{g}	<LOD^{h}	24.9 ± 0.5^{f}	32.9 ± 1.8^{e}	0.44 ± 0.03^{k}	1.2 ± 0.1^{h}
SN-SO-031	Z4	17.6 ± 2.5^{h}	0.08 ± 0.01^{i}	1.42 ± 0.09^{fg}	<LOD^{h}	41.5 ± 0.7^{d}	13.0 ± 3.0^{g}	0.22 ± 0.02^{l}	1.2 ± 0.1^{h}
SN-SO-070	Z4	9.2 ± 0.2^{i}	0.22 ± 0.01^{h}	<LOD^{g}	<LOD^{h}	22.3 ± 1.9^{f}	22.9 ± 1.2^{f}	0.51 ± 0.02^{k}	1.2 ± 0.1^{h}

SN-SO-071	Z4	17.2 ± 1.6^{h}	0.10 ± 0.00^{i}	<LOD^{g}	<LOD^{h}	23.6 ± 2.5^{f}	31.3 ± 3.2^{e}	0.68 ± 0.07^{k}	2.8 ± 0.2^{g}
SN-SO-072	Z4	14.5 ± 3.2^{h}	0.06 ± 0.00^{i}	1.09 ± 0.02^{fg}	0.015 ± 0.002^{f}	42.6 ± 3.7^{d}	18.7 ± 3.0^{f}	0.37 ± 0.05^{k}	1.7 ± 0.1^{h}
SN-SO-042	Z5	28.2 ± 3.6^{f}	0.37 ± 0.03^{g}	4.57 ± 1.49^{de}	<LOD^{h}	48.2 ± 3.4^{d}	33.6 ± 3.8^{d}	0.43 ± 0.03^{k}	4.5 ± 0.4^{e}
SN-SO-043	Z5	30.3 ± 1.7^{e}	0.40 ± 0.02^{f}	2.54 ± 0.13^{f}	<LOD^{h}	46.4 ± 3.8^{d}	44.3 ± 5.0^{c}	1.09 ± 0.02^{j}	3.4 ± 0.1^{f}
SN-SO-044	Z5	24.7 ± 0.3^{f}	0.41 ± 0.01^{f}	4.32 ± 0.20^{ef}	<LOD^{h}	30.9 ± 3.0^{e}	41.4 ± 3.5^{d}	1.74 ± 0.03^{i}	1.9 ± 0.2^{g}
SN-SO-045	Z5	30.3 ± 3.3^{e}	0.43 ± 0.04^{f}	1.95 ± 0.12^{fg}	0.010 ± 0.001^{f}	47.3 ± 2.3^{d}	39.0 ± 3.4^{d}	1.76 ± 0.09^{i}	1.7 ± 0.1^{h}
SN-SO-046	Z5	32.8 ± 2.5^{e}	0.61 ± 0.07^{e}	3.29 ± 0.17^{ef}	0.044 ± 0.007^{de}	38.4 ± 8.9^{d}	19.1 ± 0.5^{f}	1.65 ± 0.11^{i}	5.0 ± 0.3^{e}
SN-SO-047	Z5	20.9 ± 1.3^{g}	0.33 ± 0.04^{g}	3.49 ± 1.00^{ef}	0.025 ± 0.007^{ef}	61.9 ± 6.5^{c}	21.6 ± 1.5^{f}	0.65 ± 0.02^{k}	1.7 ± 0.3^{g}
SN-SO-066	Z5	31.5 ± 5.0^{e}	0.52 ± 0.00^{e}	<LOD^{g}	0.031 ± 0.002^{e}	85.0 ± 11.1^{a}	40.7 ± 1.9^{d}	1.57 ± 0.14^{i}	3.4 ± 0.2^{f}
SN-SO-067	Z5	24.7 ± 1.5^{f}	0.19 ± 0.01^{h}	4.43 ± 0.47^{ef}	0.038 ± 0.006^{e}	59.0 ± 9.4^{c}	22.7 ± 1.2^{f}	1.16 ± 0.31^{j}	2.5 ± 0.2^{g}
SN-SO-068	Z5	15.4 ± 2.1^{h}	0.45 ± 0.01^{f}	2.67 ± 0.28^{f}	0.041 ± 0.003^{de}	43.4 ± 5.1^{d}	25.0 ± 3.6^{e}	0.87 ± 0.03^{j}	2.8 ± 0.2^{g}
SN-SO-073	Z5	35.9 ± 1.6^{e}	0.54 ± 0.05^{e}	<LOD^{g}	0.034 ± 0.001^{e}	59.4 ± 11.9^{c}	45.8 ± 8.5^{c}	0.41 ± 0.03^{k}	2.4 ± 0.1^{g}
SN-SO-074	Z5	17.6 ± 0.8^{h}	0.14 ± 0.01^{h}	4.07 ± 0.11^{ef}	0.029 ± 0.001^{ef}	61.3 ± 8.5^{c}	32.8 ± 2.1^{e}	1.00 ± 0.11^{j}	5.7 ± 0.3^{e}
SN-SO-075	Z5	31.1 ± 2.8^{e}	0.33 ± 0.05^{g}	<LOD^{g}	<LOD^{h}	53.9 ± 1.7^{c}	40.1 ± 3.2^{d}	0.47 ± 0.08^{k}	2.4 ± 0.8^{g}
SN-SO-076	Z5	24.2 ± 6.8^{f}	0.26 ± 0.01^{g}	1.29 ± 0.25^{fg}	0.043 ± 0.008^{de}	32.8 ± 2.1^{e}	51.2 ± 3.1^{b}	1.04 ± 0.03^{j}	5.5 ± 0.2^{e}
SN-SO-077	Z5	43.2 ± 3.0^{d}	0.48 ± 0.06^{f}	2.34 ± 0.17^{f}	0.010 ± 0.001^{f}	47.4 ± 7.8^{d}	35.8 ± 6.5^{d}	0.77 ± 0.06^{j}	3.2 ± 0.1^{f}
SN-SO-078	Z5	18.4 ± 1.8^{g}	0.35 ± 0.04^{g}	1.83 ± 0.07^{fg}	0.018 ± 0.004^{f}	24.7 ± 1.5^{f}	35.1 ± 2.8^{d}	0.71 ± 0.06^{k}	3.6 ± 0.1^{f}
SN-SO-079	Z5	17.6 ± 1.1^{h}	0.37 ± 0.08^{g}	3.42 ± 0.23^{ef}	0.038 ± 0.007^{e}	33.9 ± 2.4^{e}	28.1 ± 1.8^{e}	0.75 ± 0.09^{j}	4.1 ± 0.6^{f}
SN-SO-080	Z5	22.4 ± 3.1^{g}	0.46 ± 0.05^{f}	<LOD^{g}	<LOD^{h}	44.8 ± 3.6^{d}	28.8 ± 2.6^{e}	1.08 ± 0.02^{j}	2.4 ± 0.3^{g}
SN-SO-092	Z5	15.6 ± 0.4^{h}	0.11 ± 0.02^{h}	2.38 ± 0.14^{f}	0.050 ± 0.005^{de}	52.7 ± 13.7^{c}	48.4 ± 0.6^{c}	2.06 ± 0.24^{h}	3.9 ± 0.3^{f}
SN-SO-093	Z5	18.0 ± 1.6^{h}	0.16 ± 0.03^{h}	3.27 ± 0.17^{ef}	0.012 ± 0.002^{f}	35.7 ± 6.6^{e}	41.8 ± 8.2^{d}	0.81 ± 0.07^{j}	4.6 ± 0.4^{e}

Notes: Values are reported as mean ± analytical SD of three independently prepared analytical portions on a dry-weight basis (mg/kg DW). The three analytical measurements were used only to assess analytical precision and were averaged to obtain one site-level concentration per matrix and georeferenced sampling site for subsequent statistical analyses. Values <LOD and <LOQ are indicated as applicable.

Table S15. Individual concentrations of macroelements in *Sambucus nigra* L. bark samples collected from georeferenced sampling points across the investigated ecological sectors of the Oradea metropolitan area, Romania.

Sample ID	Zone	K (mg/kg)	Ca (mg/kg)	Mg (mg/kg)	Na (mg/kg)	P (mg/kg)	S (mg/kg)
SN-BA-022	Z1	1666 ± 154^{cgh}	2943 ± 222^{gh}	663 ± 55^{bh}	63 ± 6^{dj}	307 ± 23^{cd}	268 ± 29^{ef}
SN-BA-023	Z1	1454 ± 132^{chi}	4092 ± 227^{e}	617 ± 39^{ch}	56 ± 5^{dj}	282 ± 27^{d}	366 ± 29^{d}
SN-BA-026	Z1	1467 ± 128^{ch}	2779 ± 237^{h}	643 ± 47^{bh}	44 ± 3^{ek}	249 ± 20^{e}	145 ± 16^{gh}
SN-BA-027	Z1	1337 ± 106^{di}	3252 ± 265^{g}	571 ± 48^{ci}	58 ± 5^{dj}	263 ± 20^{de}	331 ± 36^{de}
SN-BA-029	Z1	1610 ± 139^{ch}	4072 ± 226^{e}	594 ± 35^{c}	65 ± 5^{d}	240 ± 23^{e}	304 ± 26^{e}
SN-BA-053	Z1	1485 ± 127^{ch}	3546 ± 304^{f}	636 ± 37^{bh}	55 ± 5^{dj}	223 ± 21^{ef}	173 ± 16^{g}
SN-BA-061	Z1	1240 ± 85^{di}	2961 ± 188^{gh}	586 ± 43^{ci}	61 ± 3^{d}	208 ± 22^{f}	218 ± 18^{fg}
SN-BA-062	Z1	1411 ± 132^{chi}	3038 ± 230^{gh}	543 ± 32^{ci}	56 ± 3^{dj}	233 ± 25^{e}	176 ± 14^{g}
SN-BA-063	Z1	1444 ± 99^{ch}	3407 ± 203^{fg}	681 ± 39^{bh}	46 ± 3^{ek}	228 ± 22^{ef}	207 ± 22^{fg}
SN-BA-081	Z1	1569 ± 131^{ch}	3604 ± 222^{f}	428 ± 25^{dj}	47 ± 3^{ek}	229 ± 25^{ef}	264 ± 22^{ef}
SN-BA-082	Z1	1429 ± 135^{chi}	3063 ± 213^{gh}	494 ± 39^{ci}	48 ± 3^{ek}	259 ± 24^{de}	223 ± 17^{f}
SN-BA-083	Z1	1759 ± 99^{cg}	3559 ± 272^{f}	511 ± 48^{ci}	54 ± 5^{dj}	277 ± 28^{d}	311 ± 35^{de}
SN-BA-084	Z1	1568 ± 123^{ch}	3389 ± 306^{fg}	617 ± 45^{ch}	55 ± 5^{dj}	255 ± 23^{de}	343 ± 36^{d}
SN-BA-003	Z2	1954 ± 159^{bg}	3974 ± 282^{e}	849 ± 63^{afg}	127 ± 10^{ag}	366 ± 32^{b}	485 ± 41^{b}
SN-BA-004	Z2	2190 ± 121^{b}	4488 ± 356^{d}	974 ± 82^{afg}	135 ± 9^{ag}	317 ± 35^{c}	345 ± 33^{d}
SN-BA-014	Z2	1998 ± 115^{bg}	5144 ± 415^{b}	769 ± 44^{bg}	141 ± 8^{ag}	237 ± 21^{e}	515 ± 45^{ab}
SN-BA-018	Z2	2108 ± 198^{bg}	3932 ± 371^{e}	795 ± 58^{afg}	137 ± 8^{ag}	306 ± 32^{cd}	558 ± 49^{a}
SN-BA-019	Z2	2850 ± 162^{a}	5604 ± 317^{a}	929 ± 68^{afg}	121 ± 11^{agh}	418 ± 31^{a}	423 ± 34^{c}
SN-BA-020	Z2	2625 ± 244^{af}	5645 ± 485^{a}	825 ± 71^{afg}	92 ± 6^{ch}	318 ± 29^{c}	365 ± 32^{d}
SN-BA-048	Z2	2850 ± 203^{af}	4775 ± 409^{c}	739 ± 62^{bgh}	144 ± 10^{ag}	418 ± 37^{a}	369 ± 32^{d}
SN-BA-049	Z2	2473 ± 227^{af}	4786 ± 282^{c}	807 ± 45^{afg}	111 ± 7^{bh}	418 ± 43^{a}	493 ± 53^{ab}
SN-BA-050	Z2	2631 ± 174^{af}	5176 ± 419^{b}	878 ± 48^{afg}	107 ± 9^{bh}	372 ± 42^{ab}	439 ± 41^{bc}
SN-BA-051	Z2	2560 ± 177^{af}	4784 ± 344^{c}	776 ± 72^{afgh}	126 ± 8^{ag}	337 ± 29^{bc}	362 ± 31^{d}
SN-BA-052	Z2	1814 ± 116^{bg}	4977 ± 320^{bc}	726 ± 52^{bh}	115 ± 9^{bh}	249 ± 20^{e}	278 ± 21^{ef}
SN-BA-054	Z2	2354 ± 132^{af}	4805 ± 297^{c}	674 ± 54^{bh}	94 ± 6^{ch}	344 ± 36^{bc}	380 ± 35^{cd}

SN-BA-055	Z2	2286 ± 217^{afg}	4738 ± 327^{c}	969 ± 64^{af}	128 ± 8^{ag}	253 ± 24^{de}	385 ± 36^{cd}
SN-BA-056	Z2	2217 ± 187^{bfg}	5782 ± 413^{a}	963 ± 85^{afg}	134 ± 10^{ag}	348 ± 38^{bc}	421 ± 37^{c}
SN-BA-057	Z2	1859 ± 111^{bg}	3829 ± 322^{ef}	773 ± 59^{afgh}	99 ± 7^{bh}	352 ± 30^{b}	475 ± 43^{b}
SN-BA-059	Z2	2139 ± 118^{bg}	4818 ± 421^{c}	878 ± 65^{afg}	135 ± 10^{ag}	367 ± 40^{b}	463 ± 39^{b}
SN-BA-064	Z2	2145 ± 200^{bfg}	4563 ± 391^{cd}	680 ± 64^{bh}	103 ± 9^{bh}	351 ± 36^{b}	333 ± 30^{de}
SN-BA-065	Z2	2630 ± 172^{af}	4313 ± 286^{d}	775 ± 51^{bfgh}	152 ± 9^{a}	366 ± 37^{b}	435 ± 34^{bc}
SN-BA-069	Z2	2298 ± 161^{af}	5517 ± 347^{a}	839 ± 54^{afg}	142 ± 8^{ag}	256 ± 23^{de}	255 ± 22^{ef}
SN-BA-085	Z2	2134 ± 129^{bg}	4714 ± 283^{c}	785 ± 48^{bfg}	129 ± 8^{ag}	339 ± 28^{bc}	446 ± 46^{bc}
SN-BA-086	Z2	2668 ± 193^{af}	5884 ± 526^{a}	913 ± 56^{afg}	140 ± 8^{ag}	362 ± 41^{b}	375 ± 40^{cd}
SN-BA-087	Z2	2541 ± 240^{af}	3462 ± 268^{fg}	791 ± 69^{afgh}	158 ± 14^{ag}	418 ± 35^{a}	255 ± 25^{ef}
SN-BA-088	Z2	2610 ± 178^{af}	4593 ± 378^{cd}	974 ± 54^{a}	97 ± 8^{bh}	308 ± 31^{cd}	409 ± 40^{c}
SN-BA-094	Z2	1863 ± 163^{bgh}	5526 ± 332^{a}	982 ± 89^{afg}	140 ± 13^{ag}	298 ± 28^{cd}	472 ± 49^{b}
SN-BA-001	Z3	2169 ± 186^{bfg}	4809 ± 414^{c}	641 ± 55^{bh}	108 ± 10^{bh}	321 ± 28^{c}	443 ± 37^{bc}
SN-BA-005	Z3	1750 ± 109^{cgh}	3663 ± 213^{f}	541 ± 34^{ci}	96 ± 9^{bhi}	267 ± 22^{de}	155 ± 14^{gh}
SN-BA-006	Z3	1647 ± 101^{ch}	4582 ± 276^{cd}	671 ± 48^{bh}	123 ± 7^{bg}	327 ± 25^{c}	275 ± 26^{ef}
SN-BA-007	Z3	2311 ± 170^{af}	3628 ± 202^{f}	632 ± 52^{bh}	125 ± 9^{ag}	265 ± 27^{de}	283 ± 26^{e}
SN-BA-008	Z3	1992 ± 186^{bg}	4615 ± 360^{cd}	693 ± 43^{bh}	103 ± 7^{bh}	150 ± 15^{g}	368 ± 36^{d}
SN-BA-032	Z3	2153 ± 130^{bg}	4459 ± 267^{d}	848 ± 78^{afg}	66 ± 5^{d}	174 ± 17^{fg}	495 ± 51^{ab}
SN-BA-033	Z3	2237 ± 138^{bf}	3387 ± 212^{fg}	515 ± 36^{ci}	121 ± 10^{agh}	244 ± 20^{e}	234 ± 25^{f}
SN-BA-034	Z3	2000 ± 152^{bg}	4339 ± 278^{d}	668 ± 49^{bh}	102 ± 6^{bh}	256 ± 28^{de}	327 ± 32^{de}
SN-BA-035	Z3	2375 ± 203^{af}	3711 ± 330^{ef}	636 ± 53^{bh}	107 ± 10^{bh}	308 ± 31^{cd}	254 ± 24^{ef}
SN-BA-036	Z3	1568 ± 100^{ch}	5151 ± 463^{b}	732 ± 63^{bgh}	95 ± 5^{ch}	259 ± 20^{de}	220 ± 25^{f}
SN-BA-037	Z3	2136 ± 192^{bg}	4850 ± 389^{c}	753 ± 69^{bfgh}	70 ± 6^{di}	299 ± 30^{cd}	213 ± 19^{fg}
SN-BA-038	Z3	1531 ± 109^{ch}	3343 ± 237^{fg}	817 ± 63^{afg}	106 ± 9^{bh}	130 ± 11^{gh}	260 ± 29^{ef}
SN-BA-039	Z3	1575 ± 132^{ch}	5428 ± 340^{ab}	466 ± 27^{d}	81 ± 5^{ci}	242 ± 19^{e}	381 ± 36^{cd}
SN-BA-040	Z3	1621 ± 152^{ch}	5049 ± 380^{bc}	588 ± 48^{ci}	95 ± 8^{chi}	261 ± 27^{de}	234 ± 20^{f}
SN-BA-041	Z3	1601 ± 120^{ch}	5808 ± 540^{a}	748 ± 52^{bgh}	111 ± 8^{bh}	296 ± 33^{cd}	253 ± 28^{ef}
SN-BA-058	Z3	1928 ± 129^{bg}	4324 ± 313^{d}	415 ± 25^{dj}	94 ± 8^{chi}	262 ± 23^{de}	378 ± 43^{cd}

SN-BA-060	Z3	1948 ± 155^{bg}	3511 ± 198^{f}	788 ± 60^{afgh}	126 ± 7^{ag}	222 ± 17^{ef}	217 ± 21^{fg}
SN-BA-089	Z3	2586 ± 239^{af}	4565 ± 382^{cd}	870 ± 70^{afg}	102 ± 7^{bh}	325 ± 26^{c}	332 ± 36^{de}
SN-BA-090	Z3	2014 ± 115^{bg}	2897 ± 228^{h}	568 ± 41^{ci}	80 ± 5^{ci}	269 ± 25^{de}	512 ± 50^{ab}
SN-BA-091	Z3	2125 ± 146^{bg}	3156 ± 187^{g}	559 ± 38^{ci}	103 ± 6^{bh}	144 ± 16^{gh}	387 ± 36^{cd}
SN-BA-002	Z4	1331 ± 99^{di}	2816 ± 186^{h}	527 ± 30^{ci}	38 ± 3^{fk}	118 ± 13^{h}	163 ± 17^{g}
SN-BA-009	Z4	1395 ± 92^{dh}	2386 ± 171^{i}	609 ± 41^{c}	41 ± 3^{ek}	142 ± 14^{gh}	145 ± 11^{gh}
SN-BA-010	Z4	1068 ± 92^{ei}	3522 ± 241^{f}	381 ± 34^{dj}	30 ± 3^{f}	186 ± 18^{fg}	190 ± 21^{fg}
SN-BA-011	Z4	1435 ± 88^{ch}	2823 ± 257^{h}	491 ± 33^{ci}	36 ± 3^{f}	151 ± 15^{g}	274 ± 24^{ef}
SN-BA-012	Z4	1036 ± 70^{e}	2820 ± 203^{h}	415 ± 27^{dj}	43 ± 4^{ek}	118 ± 11^{h}	176 ± 14^{g}
SN-BA-013	Z4	947 ± 67^{e}	2952 ± 255^{gh}	459 ± 27^{d}	50 ± 3^{ej}	182 ± 21^{fg}	205 ± 22^{fg}
SN-BA-015	Z4	1155 ± 77^{di}	2173 ± 130^{ij}	337 ± 31^{ej}	30 ± 3^{f}	193 ± 21^{f}	225 ± 23^{f}
SN-BA-016	Z4	1155 ± 88^{di}	3705 ± 254^{ef}	508 ± 34^{ci}	36 ± 3^{f}	159 ± 17^{g}	228 ± 18^{f}
SN-BA-017	Z4	991 ± 83^{e}	2776 ± 222^{h}	395 ± 33^{dj}	47 ± 4^{ek}	156 ± 12^{g}	251 ± 19^{ef}
SN-BA-021	Z4	1006 ± 95^{e}	2931 ± 264^{gh}	599 ± 45^{ci}	45 ± 3^{ek}	140 ± 15^{gh}	193 ± 18^{fg}
SN-BA-024	Z4	897 ± 66^{e}	2404 ± 194^{i}	612 ± 41^{ch}	42 ± 3^{ek}	118 ± 11^{h}	179 ± 18^{g}
SN-BA-025	Z4	1671 ± 144^{cgh}	2438 ± 147^{i}	469 ± 36^{di}	34 ± 3^{f}	235 ± 20^{e}	219 ± 16^{fg}
SN-BA-028	Z4	1185 ± 103^{di}	3766 ± 248^{ef}	334 ± 25^{e}	40 ± 4^{ek}	138 ± 13^{gh}	186 ± 18^{g}
SN-BA-030	Z4	1582 ± 95^{ch}	2788 ± 232^{h}	477 ± 40^{ci}	41 ± 3^{ek}	192 ± 16^{f}	192 ± 19^{fg}
SN-BA-031	Z4	1421 ± 84^{dh}	2902 ± 207^{gh}	435 ± 28^{dj}	46 ± 3^{ek}	160 ± 15^{g}	145 ± 15^{gh}
SN-BA-070	Z4	1150 ± 77^{di}	2903 ± 259^{gh}	330 ± 28^{e}	52 ± 4^{dj}	198 ± 18^{f}	152 ± 16^{gh}
SN-BA-071	Z4	1475 ± 95^{ch}	3539 ± 221^{f}	432 ± 33^{dj}	50 ± 4^{ejk}	145 ± 15^{gh}	205 ± 21^{fg}
SN-BA-072	Z4	1086 ± 75^{ei}	3038 ± 212^{gh}	450 ± 36^{dj}	48 ± 3^{ek}	128 ± 12^{h}	186 ± 15^{g}
SN-BA-042	Z5	2282 ± 198^{afg}	4514 ± 303^{cd}	735 ± 58^{bgh}	119 ± 8^{bg}	254 ± 21^{de}	214 ± 23^{fg}
SN-BA-043	Z5	2622 ± 162^{af}	3602 ± 237^{f}	651 ± 58^{bh}	105 ± 9^{bh}	357 ± 41^{b}	392 ± 34^{cd}
SN-BA-044	Z5	1952 ± 155^{bg}	4215 ± 383^{de}	695 ± 61^{bh}	82 ± 7^{ci}	213 ± 16^{ef}	352 ± 37^{d}
SN-BA-045	Z5	1752 ± 139^{bgh}	3864 ± 224^{ef}	422 ± 26^{dj}	87 ± 8^{ci}	149 ± 15^{gh}	191 ± 18^{fg}
SN-BA-046	Z5	2081 ± 130^{bg}	3973 ± 315^{e}	597 ± 39^{c}	84 ± 5^{ci}	324 ± 26^{c}	449 ± 49^{bc}
SN-BA-047	Z5	2107 ± 171^{bg}	4188 ± 356^{de}	473 ± 30^{di}	109 ± 7^{bh}	244 ± 28^{e}	378 ± 35^{cd}

SN-BA-066	Z5	2129 ± 163^{bg}	3499 ± 308^{fg}	487 ± 38^{ci}	61 ± 5^{dj}	325 ± 32^{c}	365 ± 36^{d}
SN-BA-067	Z5	1879 ± 105^{bg}	4488 ± 268^{d}	629 ± 44^{bh}	82 ± 8^{ci}	222 ± 19^{ef}	317 ± 32^{de}
SN-BA-068	Z5	1494 ± 99^{ch}	4274 ± 369^{de}	549 ± 42^{ci}	87 ± 6^{ci}	288 ± 22^{d}	339 ± 29^{de}
SN-BA-073	Z5	1565 ± 120^{ch}	3394 ± 205^{fg}	746 ± 67^{bfgh}	123 ± 9^{ag}	272 ± 26^{d}	196 ± 20^{fg}
SN-BA-074	Z5	1725 ± 119^{cgh}	4610 ± 405^{cd}	455 ± 28^{d}	92 ± 6^{ch}	351 ± 39^{b}	283 ± 28^{e}
SN-BA-075	Z5	1621 ± 130^{ch}	3528 ± 227^{f}	489 ± 44^{ci}	62 ± 4^{dj}	169 ± 17^{g}	172 ± 17^{g}
SN-BA-076	Z5	1592 ± 131^{ch}	2770 ± 168^{h}	492 ± 36^{ci}	84 ± 5^{ci}	250 ± 23^{de}	245 ± 19^{f}
SN-BA-077	Z5	2698 ± 150^{af}	2992 ± 193^{gh}	606 ± 46^{chi}	79 ± 5^{ci}	390 ± 43^{a}	160 ± 17^{g}
SN-BA-078	Z5	2299 ± 190^{af}	4086 ± 247^{e}	592 ± 48^{ci}	92 ± 7^{chi}	277 ± 29^{d}	306 ± 27^{e}
SN-BA-079	Z5	1921 ± 115^{bg}	4271 ± 309^{de}	571 ± 44^{ci}	58 ± 5^{dj}	282 ± 27^{d}	348 ± 31^{d}
SN-BA-080	Z5	2234 ± 160^{bf}	3872 ± 221^{ef}	507 ± 45^{ci}	93 ± 5^{ch}	240 ± 22^{e}	207 ± 17^{fg}
SN-BA-092	Z5	2043 ± 140^{bg}	2775 ± 257^{h}	805 ± 59^{afg}	114 ± 10^{bh}	201 ± 16^{f}	414 ± 33^{c}
SN-BA-093	Z5	2406 ± 222^{af}	4793 ± 328^{c}	645 ± 50^{bh}	55 ± 4^{dj}	267 ± 21^{de}	317 ± 30^{de}

Notes: Values are reported as mean ± analytical SD of three independently prepared analytical portions on a dry-weight basis (mg/kg DW). The three analytical measurements were used only to assess analytical precision and were averaged to obtain one site-level concentration per matrix and georeferenced sampling site for subsequent statistical analyses. Values <LOD and <LOQ are indicated as applicable.

Table S16. Individual concentrations of environmentally associated microelements and biologically relevant trace elements measured in georeferenced bark samples of *Sambucus nigra* L. collected across the investigated ecological sectors of the Oradea metropolitan area, Romania.

Sample ID	Zone	^{56}Fe (mg/kg)	^{55}Mn (mg/kg)	^{64}Zn (mg/kg)	^{63}Cu (mg/kg)	^{11}B (mg/kg)	^{98}Mo (mg/kg)	^{59}Co (mg/kg)	^{80}Se (mg/kg)
SN-BA-022	Z1	362 ± 31^{j}	85.0 ± 6.7^{g}	27.7 ± 2.5^{i}	9.0 ± 0.8^{i}	12.5 ± 0.8^{i}	0.151 ± 0.010^{e}	0.161 ± 0.010^{j}	0.034 ± 0.003^{j}
SN-BA-023	Z1	479 ± 38^{g}	70.3 ± 5.9^{i}	23.2 ± 2.0^{j}	9.4 ± 0.9^{i}	12.3 ± 0.9^{i}	0.115 ± 0.010^{h}	0.134 ± 0.010^{k}	0.062 ± 0.004^{c}
SN-BA-026	Z1	423 ± 32^{i}	58.1 ± 5.1^{k}	22.3 ± 2.1^{k}	11.6 ± 1.0^{e}	12.7 ± 0.9^{h}	0.065 ± 0.006^{k}	0.175 ± 0.010^{j}	0.036 ± 0.002^{j}
SN-BA-027	Z1	265 ± 17^{k}	64.0 ± 5.6^{j}	18.6 ± 1.1^{k}	8.2 ± 0.6^{j}	11.3 ± 0.8^{j}	0.121 ± 0.010^{h}	0.262 ± 0.020^{g}	0.046 ± 0.003^{g}
SN-BA-029	Z1	449 ± 32^{h}	84.0 ± 7.3^{g}	30.9 ± 2.9^{h}	8.2 ± 0.6^{j}	8.4 ± 0.5^{l}	0.105 ± 0.010^{i}	0.197 ± 0.010^{i}	0.055 ± 0.004^{e}
SN-BA-053	Z1	589 ± 37^{f}	59.1 ± 3.7^{k}	32.9 ± 3.0^{g}	10.8 ± 0.6^{g}	13.7 ± 0.9^{g}	0.057 ± 0.005^{l}	0.111 ± 0.010^{l}	0.074 ± 0.007^{a}
SN-BA-061	Z1	478 ± 36^{h}	86.9 ± 7.7^{f}	29.5 ± 2.4^{i}	7.8 ± 0.7^{j}	15.2 ± 1.1^{e}	0.106 ± 0.010^{h}	0.182 ± 0.020^{j}	0.068 ± 0.004^{b}
SN-BA-062	Z1	364 ± 32^{j}	76.7 ± 5.5^{h}	20.2 ± 1.4^{k}	11.0 ± 0.7^{g}	17.7 ± 1.4^{c}	0.100 ± 0.010^{i}	0.142 ± 0.010^{k}	0.058 ± 0.005^{d}

SN-BA-063	Z1	332 ± 21^{j}	81.6 ± 7.2^{g}	38.4 ± 2.3^{f}	9.9 ± 0.7^{h}	9.3 ± 0.6^{k}	0.054 ± 0.004^{l}	0.213 ± 0.020^{i}	0.042 ± 0.003^{i}
SN-BA-081	Z1	449 ± 30^{h}	64.2 ± 4.7^{j}	33.9 ± 2.8^{g}	9.5 ± 0.9^{h}	10.1 ± 0.9^{k}	0.144 ± 0.010^{f}	0.245 ± 0.020^{g}	0.046 ± 0.003^{g}
SN-BA-082	Z1	305 ± 24^{k}	83.2 ± 5.6^{g}	31.3 ± 2.7^{h}	10.8 ± 0.7^{g}	13.9 ± 1.1^{g}	0.079 ± 0.005^{j}	0.316 ± 0.020^{e}	0.065 ± 0.005^{c}
SN-BA-083	Z1	395 ± 34^{j}	80.7 ± 6.0^{g}	32.0 ± 2.6^{h}	9.9 ± 0.9^{h}	9.0 ± 0.8^{l}	0.081 ± 0.007^{j}	0.213 ± 0.010^{h}	0.053 ± 0.004^{f}
SN-BA-084	Z1	511 ± 34^{g}	64.4 ± 3.9^{j}	18.2 ± 1.6^{l}	11.4 ± 0.8^{f}	10.6 ± 0.7^{j}	0.095 ± 0.010^{i}	0.282 ± 0.030^{f}	0.045 ± 0.004^{g}
SN-BA-003	Z2	766 ± 61^{b}	107.8 ± 8.2^{c}	47.9 ± 3.1^{c}	11.7 ± 0.9^{e}	15.5 ± 1.0^{e}	0.132 ± 0.010^{g}	0.438 ± 0.030^{b}	0.062 ± 0.005^{c}
SN-BA-004	Z2	870 ± 54^{a}	107.1 ± 8.3^{c}	58.2 ± 4.1^{a}	14.7 ± 1.1^{b}	18.5 ± 1.2^{b}	0.216 ± 0.020^{b}	0.527 ± 0.040^{a}	0.080 ± 0.007^{a}
SN-BA-014	Z2	638 ± 36^{e}	135.2 ± 11.3^{a}	64.2 ± 3.8^{a}	15.4 ± 1.3^{a}	16.0 ± 1.0^{d}	0.195 ± 0.020^{c}	0.543 ± 0.050^{a}	0.054 ± 0.005^{e}
SN-BA-018	Z2	898 ± 50^{a}	106.4 ± 7.0^{c}	35.5 ± 3.2^{f}	14.7 ± 1.2^{b}	17.7 ± 1.2^{c}	0.252 ± 0.020^{a}	0.327 ± 0.030^{e}	0.046 ± 0.003^{g}
SN-BA-019	Z2	700 ± 53^{c}	125.5 ± 9.3^{a}	51.1 ± 3.5^{b}	16.0 ± 0.9^{a}	15.9 ± 1.4^{e}	0.156 ± 0.010^{e}	0.580 ± 0.040^{a}	0.052 ± 0.005^{f}
SN-BA-020	Z2	789 ± 48^{b}	98.5 ± 6.5^{d}	48.0 ± 4.6^{c}	14.3 ± 1.1^{b}	22.7 ± 1.3^{a}	0.207 ± 0.010^{b}	0.458 ± 0.040^{b}	0.042 ± 0.003^{h}
SN-BA-048	Z2	836 ± 56^{a}	124.0 ± 8.9^{a}	56.7 ± 3.1^{a}	15.8 ± 1.5^{a}	16.7 ± 1.0^{d}	0.154 ± 0.010^{e}	0.466 ± 0.030^{b}	0.059 ± 0.004^{d}
SN-BA-049	Z2	822 ± 62^{b}	71.5 ± 4.4^{i}	36.9 ± 2.2^{f}	9.2 ± 0.7^{i}	20.8 ± 1.6^{a}	0.163 ± 0.010^{e}	0.266 ± 0.020^{f}	0.036 ± 0.003^{j}
SN-BA-050	Z2	833 ± 65^{a}	97.0 ± 6.8^{e}	36.9 ± 2.7^{f}	14.6 ± 1.0^{b}	12.2 ± 0.9^{i}	0.104 ± 0.010^{i}	0.406 ± 0.020^{c}	0.063 ± 0.004^{c}
SN-BA-051	Z2	613 ± 56^{e}	131.3 ± 7.8^{a}	46.9 ± 4.0^{c}	14.8 ± 1.3^{a}	14.6 ± 1.3^{f}	0.177 ± 0.020^{d}	0.436 ± 0.020^{b}	0.068 ± 0.004^{b}
SN-BA-052	Z2	843 ± 59^{a}	73.0 ± 6.6^{i}	32.0 ± 2.1^{h}	12.5 ± 0.9^{d}	20.0 ± 1.5^{b}	0.180 ± 0.010^{d}	0.312 ± 0.020^{e}	0.021 ± 0.002^{k}
SN-BA-054	Z2	607 ± 50^{e}	104.1 ± 9.2^{c}	58.2 ± 4.6^{a}	12.8 ± 1.2^{d}	15.2 ± 1.1^{f}	0.085 ± 0.010^{j}	0.515 ± 0.040^{a}	0.042 ± 0.003^{h}
SN-BA-055	Z2	898 ± 82^{a}	96.3 ± 5.7^{e}	34.9 ± 2.1^{g}	11.9 ± 1.0^{e}	18.4 ± 1.5^{b}	0.273 ± 0.020^{a}	0.286 ± 0.030^{f}	0.021 ± 0.002^{k}
SN-BA-056	Z2	534 ± 39^{g}	135.1 ± 12.1^{a}	58.1 ± 3.8^{a}	15.3 ± 0.9^{a}	14.7 ± 1.3^{f}	0.191 ± 0.010^{c}	0.555 ± 0.030^{a}	0.058 ± 0.005^{d}
SN-BA-057	Z2	711 ± 46^{c}	91.4 ± 7.8^{f}	35.4 ± 2.0^{g}	9.6 ± 0.5^{h}	21.5 ± 1.8^{a}	0.236 ± 0.010^{a}	0.298 ± 0.020^{f}	0.054 ± 0.005^{e}
SN-BA-059	Z2	680 ± 43^{d}	101.2 ± 8.3^{d}	41.5 ± 2.7^{e}	14.1 ± 1.2^{b}	15.8 ± 1.2^{e}	0.128 ± 0.010^{g}	0.432 ± 0.040^{b}	0.070 ± 0.005^{b}
SN-BA-064	Z2	824 ± 48^{b}	99.7 ± 7.2^{d}	42.3 ± 3.0^{e}	11.4 ± 0.9^{f}	14.7 ± 1.0^{f}	0.227 ± 0.020^{b}	0.414 ± 0.030^{c}	0.071 ± 0.006^{b}
SN-BA-065	Z2	881 ± 79^{a}	116.3 ± 6.8^{b}	54.7 ± 3.0^{b}	14.7 ± 1.0^{b}	16.0 ± 1.2^{e}	0.152 ± 0.010^{e}	0.535 ± 0.040^{a}	0.059 ± 0.004^{d}
SN-BA-069	Z2	755 ± 43^{b}	134.0 ± 12.6^{a}	55.2 ± 4.2^{a}	15.7 ± 1.0^{a}	16.2 ± 1.4^{d}	0.195 ± 0.010^{c}	0.537 ± 0.050^{a}	0.075 ± 0.007^{a}
SN-BA-085	Z2	654 ± 44^{d}	110.3 ± 6.3^{b}	47.2 ± 4.4^{c}	12.8 ± 0.7^{d}	19.5 ± 1.1^{b}	0.207 ± 0.020^{b}	0.470 ± 0.040^{b}	0.052 ± 0.003^{f}
SN-BA-086	Z2	702 ± 49^{c}	96.7 ± 6.3^{e}	33.6 ± 2.5^{g}	11.6 ± 0.8^{e}	17.9 ± 1.5^{c}	0.194 ± 0.010^{c}	0.353 ± 0.030^{d}	0.060 ± 0.005^{d}
SN-BA-087	Z2	955 ± 59^{a}	93.5 ± 5.4^{e}	46.0 ± 3.4^{d}	12.8 ± 1.2^{c}	13.4 ± 1.2^{h}	0.118 ± 0.010^{h}	0.275 ± 0.020^{f}	0.042 ± 0.003^{h}
SN-BA-088	Z2	732 ± 63^{c}	124.0 ± 8.5^{a}	61.8 ± 5.1^{a}	16.0 ± 1.4^{a}	14.6 ± 0.9^{f}	0.114 ± 0.010^{h}	0.585 ± 0.050^{a}	0.033 ± 0.003^{j}

SN-BA-094	Z2	441 ± 37^{i}	120.7 ± 9.4^{a}	44.4 ± 3.9^{d}	14.8 ± 1.1^{a}	17.5 ± 1.3^{c}	0.217 ± 0.010^{b}	0.385 ± 0.030^{c}	0.021 ± 0.002^{k}
SN-BA-001	Z3	629 ± 55^{e}	108.2 ± 8.0^{b}	41.1 ± 3.0^{e}	11.2 ± 0.7^{f}	12.8 ± 1.1^{h}	0.163 ± 0.010^{d}	0.184 ± 0.020^{j}	0.046 ± 0.004^{g}
SN-BA-005	Z3	644 ± 53^{d}	98.0 ± 6.2^{d}	46.9 ± 3.7^{c}	13.7 ± 1.0^{c}	15.1 ± 1.0^{f}	0.178 ± 0.020^{d}	0.301 ± 0.020^{e}	0.044 ± 0.004^{h}
SN-BA-006	Z3	561 ± 32^{f}	62.1 ± 4.7^{k}	24.5 ± 2.1^{j}	8.5 ± 0.8^{j}	20.3 ± 1.3^{b}	0.160 ± 0.010^{e}	0.237 ± 0.020^{h}	0.048 ± 0.004^{f}
SN-BA-007	Z3	410 ± 32^{i}	102.0 ± 6.4^{c}	22.0 ± 1.6^{k}	13.4 ± 0.9^{c}	13.6 ± 1.1^{g}	0.067 ± 0.006^{k}	0.205 ± 0.020^{i}	0.040 ± 0.004^{i}
SN-BA-008	Z3	613 ± 58^{e}	73.7 ± 6.7^{h}	32.4 ± 2.2^{g}	11.0 ± 0.8^{f}	15.2 ± 1.0^{e}	0.138 ± 0.010^{f}	0.205 ± 0.010^{i}	0.054 ± 0.004^{e}
SN-BA-032	Z3	577 ± 44^{f}	91.9 ± 7.5^{f}	49.2 ± 3.0^{c}	13.6 ± 1.1^{c}	15.7 ± 1.2^{e}	0.074 ± 0.007^{k}	0.366 ± 0.030^{d}	0.019 ± 0.002^{l}
SN-BA-033	Z3	605 ± 35^{e}	90.3 ± 5.0^{f}	43.7 ± 3.4^{d}	11.9 ± 0.8^{e}	21.8 ± 1.4^{a}	0.105 ± 0.010^{h}	0.215 ± 0.020^{h}	0.032 ± 0.003^{k}
SN-BA-034	Z3	557 ± 40^{f}	96.7 ± 6.5^{e}	37.0 ± 2.1^{f}	11.8 ± 1.1^{e}	20.0 ± 1.6^{b}	0.218 ± 0.010^{b}	0.392 ± 0.020^{c}	0.065 ± 0.005^{c}
SN-BA-035	Z3	453 ± 39^{h}	75.7 ± 6.6^{h}	42.8 ± 2.5^{e}	11.5 ± 1.1^{f}	20.4 ± 1.5^{a}	0.078 ± 0.007^{j}	0.425 ± 0.040^{b}	0.028 ± 0.002^{k}
SN-BA-036	Z3	500 ± 32^{g}	91.9 ± 6.1^{f}	41.4 ± 3.1^{e}	11.3 ± 0.6^{f}	17.6 ± 1.2^{c}	0.142 ± 0.010^{f}	0.254 ± 0.010^{g}	0.049 ± 0.003^{f}
SN-BA-037	Z3	308 ± 21^{k}	95.8 ± 7.6^{e}	50.7 ± 4.6^{b}	10.9 ± 0.9^{g}	13.0 ± 1.1^{h}	0.157 ± 0.010^{e}	0.231 ± 0.020^{h}	0.053 ± 0.004^{f}
SN-BA-038	Z3	469 ± 38^{h}	96.5 ± 7.0^{e}	51.4 ± 3.9^{b}	9.1 ± 0.7^{i}	13.8 ± 0.9^{g}	0.138 ± 0.010^{f}	0.202 ± 0.020^{i}	0.068 ± 0.005^{b}
SN-BA-039	Z3	434 ± 36^{i}	95.2 ± 8.6^{e}	23.8 ± 1.8^{j}	10.4 ± 0.9^{g}	10.2 ± 0.9^{k}	0.182 ± 0.010^{d}	0.346 ± 0.030^{d}	0.043 ± 0.003^{h}
SN-BA-040	Z3	509 ± 38^{g}	113.1 ± 9.5^{b}	41.6 ± 2.5^{e}	9.3 ± 0.7^{i}	13.9 ± 0.9^{g}	0.126 ± 0.010^{g}	0.337 ± 0.020^{e}	0.067 ± 0.005^{b}
SN-BA-041	Z3	566 ± 44^{f}	100.4 ± 7.2^{d}	44.6 ± 4.0^{d}	10.8 ± 1.0^{g}	10.2 ± 0.9^{k}	0.054 ± 0.003^{l}	0.351 ± 0.020^{d}	0.078 ± 0.007^{a}
SN-BA-058	Z3	675 ± 55^{d}	66.0 ± 4.2^{j}	46.2 ± 3.6^{d}	7.2 ± 0.4^{k}	17.4 ± 1.6^{c}	0.123 ± 0.010^{g}	0.294 ± 0.020^{f}	0.073 ± 0.005^{a}
SN-BA-060	Z3	628 ± 50^{e}	72.1 ± 5.3^{i}	27.6 ± 2.3^{i}	9.7 ± 0.6^{h}	14.7 ± 1.3^{f}	0.180 ± 0.010^{d}	0.184 ± 0.020^{j}	0.063 ± 0.006^{c}
SN-BA-089	Z3	750 ± 56^{b}	107.4 ± 7.8^{c}	31.4 ± 2.6^{h}	12.2 ± 0.8^{d}	18.2 ± 1.1^{b}	0.084 ± 0.010^{j}	0.369 ± 0.030^{d}	0.066 ± 0.004^{b}
SN-BA-090	Z3	704 ± 53^{c}	70.7 ± 6.1^{i}	22.0 ± 1.3^{k}	7.2 ± 0.4^{k}	16.5 ± 1.0^{d}	0.130 ± 0.010^{g}	0.133 ± 0.010^{k}	0.041 ± 0.003^{i}
SN-BA-091	Z3	409 ± 24^{i}	86.8 ± 5.1^{g}	37.2 ± 3.2^{f}	11.1 ± 0.6^{f}	14.5 ± 0.9^{g}	0.192 ± 0.010^{c}	0.261 ± 0.020^{g}	0.025 ± 0.002^{k}
SN-BA-002	Z4	342 ± 29^{j}	58.4 ± 5.2^{k}	28.3 ± 2.7^{i}	6.9 ± 0.6^{l}	9.4 ± 0.6^{k}	0.050 ± 0.003^{l}	0.124 ± 0.010^{l}	0.037 ± 0.003^{j}
SN-BA-009	Z4	244 ± 16^{l}	53.5 ± 4.7^{l}	18.3 ± 1.3^{l}	10.0 ± 0.6^{h}	6.2 ± 0.4^{l}	0.087 ± 0.007^{j}	0.147 ± 0.010^{k}	0.024 ± 0.002^{k}
SN-BA-010	Z4	314 ± 29^{k}	81.7 ± 7.7^{g}	32.2 ± 1.9^{h}	7.8 ± 0.6^{k}	13.4 ± 1.1^{h}	0.078 ± 0.007^{j}	0.240 ± 0.010^{g}	0.041 ± 0.003^{i}
SN-BA-011	Z4	236 ± 17^{l}	70.1 ± 4.0^{i}	26.7 ± 1.9^{j}	8.7 ± 0.7^{j}	8.3 ± 0.7^{l}	0.097 ± 0.010^{i}	0.059 ± 0.004^{l}	0.044 ± 0.003^{h}
SN-BA-012	Z4	186 ± 10^{m}	61.7 ± 4.7^{k}	26.6 ± 1.8^{j}	5.2 ± 0.4^{l}	10.8 ± 0.8^{j}	0.139 ± 0.010^{f}	0.225 ± 0.020^{h}	0.037 ± 0.002^{j}
SN-BA-013	Z4	226 ± 13^{l}	80.7 ± 5.0^{h}	18.3 ± 1.4^{l}	7.6 ± 0.6^{k}	10.8 ± 0.9^{j}	0.108 ± 0.010^{h}	0.145 ± 0.010^{k}	0.019 ± 0.002^{l}
SN-BA-015	Z4	423 ± 31^{i}	56.1 ± 3.6^{k}	17.4 ± 1.3^{l}	5.9 ± 0.4^{l}	11.9 ± 0.8^{i}	0.109 ± 0.010^{h}	0.272 ± 0.020^{f}	0.016 ± 0.001^{l}

SN-BA-016	Z4	156 ± 9[n]	63.9 ± 5.6[j]	27.5 ± 1.7[j]	10.4 ± 0.8[g]	12.4 ± 0.9[i]	0.103 ± 0.010[i]	0.095 ± 0.010[l]	0.038 ± 0.003[i]
SN-BA-017	Z4	281 ± 26[k]	72.4 ± 6.6[i]	32.9 ± 2.5[g]	6.5 ± 0.6[l]	10.5 ± 0.6[j]	0.034 ± 0.003[l]	0.143 ± 0.010[k]	0.026 ± 0.002[k]
SN-BA-021	Z4	224 ± 17[l]	52.5 ± 4.9[l]	27.9 ± 2.3[i]	8.3 ± 0.7[j]	9.1 ± 0.7[k]	0.073 ± 0.007[k]	0.178 ± 0.010[j]	0.036 ± 0.003[j]
SN-BA-024	Z4	413 ± 29[i]	66.9 ± 4.6[j]	27.2 ± 1.7[j]	6.1 ± 0.5[l]	9.4 ± 0.8[k]	0.070 ± 0.006[k]	0.197 ± 0.010[i]	0.039 ± 0.003[i]
SN-BA-025	Z4	334 ± 32[j]	58.6 ± 3.5[k]	23.0 ± 1.5[j]	7.4 ± 0.6[k]	10.9 ± 0.7[j]	0.147 ± 0.010[f]	0.117 ± 0.010[l]	0.042 ± 0.003[h]
SN-BA-028	Z4	352 ± 24[j]	50.4 ± 3.2[l]	21.6 ± 1.3[k]	10.4 ± 0.8[g]	14.5 ± 0.9[g]	0.053 ± 0.004[l]	0.146 ± 0.010[k]	0.058 ± 0.005[d]
SN-BA-030	Z4	439 ± 31[i]	71.9 ± 5.5[i]	32.5 ± 2.3[g]	6.6 ± 0.5[l]	6.2 ± 0.4[l]	0.075 ± 0.005[k]	0.185 ± 0.010[i]	0.055 ± 0.004[e]
SN-BA-031	Z4	279 ± 17[k]	50.8 ± 4.6[l]	18.4 ± 1.6[k]	7.0 ± 0.5[k]	12.7 ± 0.8[i]	0.088 ± 0.010[j]	0.115 ± 0.010[l]	0.037 ± 0.003[j]
SN-BA-070	Z4	321 ± 21[k]	38.1 ± 3.1[l]	16.9 ± 1.0[l]	6.9 ± 0.4[k]	13.0 ± 1.0[h]	0.070 ± 0.006[k]	0.157 ± 0.010[j]	0.046 ± 0.003[g]
SN-BA-071	Z4	365 ± 27[j]	41.6 ± 3.7[l]	22.1 ± 2.0[k]	7.4 ± 0.5[k]	15.1 ± 0.9[f]	0.097 ± 0.010[i]	0.188 ± 0.010[i]	0.045 ± 0.004[h]
SN-BA-072	Z4	464 ± 42[h]	56.2 ± 4.4[k]	18.3 ± 1.2[l]	8.4 ± 0.8[j]	14.0 ± 1.0[g]	0.142 ± 0.010[f]	0.275 ± 0.020[f]	0.047 ± 0.004[g]
SN-BA-042	Z5	512 ± 39[g]	114.2 ± 6.7[b]	40.5 ± 2.4[e]	12.2 ± 0.9[d]	8.3 ± 0.5[l]	0.091 ± 0.010[i]	0.265 ± 0.020[g]	0.071 ± 0.006[a]
SN-BA-043	Z5	539 ± 30[g]	80.5 ± 6.8[h]	42.4 ± 2.5[e]	8.9 ± 0.8[i]	17.0 ± 1.1[d]	0.229 ± 0.020[a]	0.213 ± 0.010[h]	0.064 ± 0.006[c]
SN-BA-044	Z5	750 ± 47[b]	66.8 ± 3.8[j]	46.1 ± 4.3[d]	12.3 ± 1.0[d]	10.4 ± 1.0[j]	0.238 ± 0.020[a]	0.359 ± 0.030[d]	0.054 ± 0.005[e]
SN-BA-045	Z5	469 ± 43[h]	84.7 ± 4.9[g]	51.1 ± 4.1[b]	12.9 ± 1.1[c]	23.7 ± 1.6[a]	0.186 ± 0.010[c]	0.373 ± 0.030[d]	0.062 ± 0.004[c]
SN-BA-046	Z5	697 ± 63[d]	75.4 ± 4.9[h]	29.4 ± 1.7[i]	12.7 ± 1.1[d]	13.5 ± 1.1[h]	0.136 ± 0.010[g]	0.412 ± 0.040[c]	0.080 ± 0.007[a]
SN-BA-047	Z5	809 ± 61[b]	110.8 ± 8.6[b]	32.3 ± 2.2[h]	14.5 ± 1.3[b]	20.8 ± 1.5[a]	0.136 ± 0.010[g]	0.248 ± 0.020[g]	0.067 ± 0.005[b]
SN-BA-066	Z5	484 ± 33[g]	90.7 ± 8.4[f]	44.7 ± 4.0[d]	13.0 ± 0.9[c]	17.3 ± 1.5[d]	0.236 ± 0.010[a]	0.235 ± 0.020[h]	0.061 ± 0.005[d]
SN-BA-067	Z5	717 ± 67[c]	104.5 ± 9.9[c]	48.2 ± 3.1[c]	11.9 ± 0.8[e]	16.3 ± 0.9[d]	0.204 ± 0.010[c]	0.316 ± 0.020[e]	0.055 ± 0.004[e]
SN-BA-068	Z5	479 ± 31[g]	73.7 ± 6.3[h]	43.1 ± 2.9[d]	9.4 ± 0.6[i]	11.9 ± 0.9[i]	0.142 ± 0.010[f]	0.140 ± 0.010[k]	0.053 ± 0.005[f]
SN-BA-073	Z5	679 ± 54[d]	90.4 ± 7.0[f]	37.5 ± 3.0[f]	11.1 ± 0.8[f]	15.8 ± 0.9[e]	0.218 ± 0.020[b]	0.161 ± 0.010[j]	0.045 ± 0.003[g]
SN-BA-074	Z5	596 ± 36[f]	97.0 ± 8.0[d]	57.8 ± 5.0[a]	12.1 ± 1.1[d]	9.0 ± 0.8[k]	0.163 ± 0.010[d]	0.340 ± 0.020[e]	0.021 ± 0.002[l]
SN-BA-075	Z5	563 ± 34[f]	102.2 ± 7.4[c]	52.7 ± 3.0[b]	13.9 ± 1.2[b]	17.9 ± 1.2[b]	0.265 ± 0.020[a]	0.257 ± 0.020[g]	0.048 ± 0.004[f]
SN-BA-076	Z5	563 ± 36[f]	100.9 ± 5.6[d]	38.3 ± 2.7[f]	13.1 ± 1.1[c]	17.0 ± 0.9[d]	0.191 ± 0.010[c]	0.388 ± 0.030[c]	0.056 ± 0.005[d]
SN-BA-077	Z5	382 ± 23[j]	79.2 ± 5.0[h]	29.7 ± 2.3[h]	9.5 ± 0.6[h]	12.7 ± 1.0[i]	0.064 ± 0.006[k]	0.393 ± 0.020[c]	0.029 ± 0.002[k]
SN-BA-078	Z5	709 ± 51[c]	119.5 ± 10.1[b]	53.6 ± 5.0[b]	12.9 ± 1.2[c]	17.3 ± 1.6[c]	0.229 ± 0.010[a]	0.416 ± 0.020[b]	0.042 ± 0.004[i]
SN-BA-079	Z5	751 ± 63[b]	69.3 ± 3.9[j]	29.3 ± 1.6[i]	8.9 ± 0.7[j]	11.0 ± 0.7[j]	0.150 ± 0.010[e]	0.415 ± 0.030[c]	0.076 ± 0.006[a]
SN-BA-080	Z5	680 ± 58[d]	111.0 ± 8.9[b]	28.7 ± 2.6[i]	11.8 ± 1.0[e]	20.9 ± 1.8[a]	0.205 ± 0.010[b]	0.221 ± 0.020[h]	0.040 ± 0.003[i]

SN-BA-092	Z5	619 ± 45^{e}	92.7 ± 7.2^{f}	53.1 ± 5.0^{b}	10.2 ± 0.8^{h}	17.8 ± 1.0^{c}	0.129 ± 0.010^{g}	0.315 ± 0.020^{e}	0.031 ± 0.003^{k}
SN-BA-093	Z5	644 ± 51^{d}	98.7 ± 6.7^{d}	48.1 ± 3.7^{c}	9.2 ± 0.6^{i}	13.1 ± 1.0^{h}	0.166 ± 0.010^{d}	0.347 ± 0.030^{d}	0.054 ± 0.005^{e}

Notes: Values are reported as mean ± analytical SD of three independently prepared analytical portions on a dry-weight basis (mg/kg DW). The three analytical measurements were used only to assess analytical precision and were averaged to obtain one site-level concentration per matrix and georeferenced sampling site for subsequent statistical analyses. Values <LOD and <LOQ are indicated as applicable.

Table S17. Individual concentrations of lithogenic and geochemically associated elements in *Sambucus nigra* L. bark samples collected at georeferenced sampling points across the ecological sectors of the Oradea metropolitan area, Romania.

Sample ID	Zone	^{27}Al (mg/kg)	^{48}Ti (mg/kg)	^{51}V (mg/kg)	^{137}Ba (mg/kg)	^{88}Sr (mg/kg)	^{7}Li (mg/kg)	^{85}Rb (mg/kg)	^{133}Cs (mg/kg)
SN-BA-022	Z1	66.9 ± 3.9^{gh}	4.68 ± 0.31^{h}	0.86 ± 0.06^{hi}	20.4 ± 1.7^{gh}	12.5 ± 0.8^{fg}	0.54 ± 0.04^{ij}	2.08 ± 0.14^{g}	0.064 ± 0.005^{g}
SN-BA-023	Z1	73.7 ± 5.6^{gh}	5.24 ± 0.30^{h}	0.78 ± 0.06^{i}	23.8 ± 1.4^{g}	15.3 ± 1.1^{ef}	0.79 ± 0.05^{de}	2.54 ± 0.19^{ef}	0.081 ± 0.007^{f}
SN-BA-026	Z1	60.8 ± 5.7^{h}	4.25 ± 0.29^{i}	0.64 ± 0.06^{ij}	19.7 ± 1.0^{h}	12.2 ± 0.8^{g}	0.57 ± 0.04^{f}	1.94 ± 0.13^{g}	0.048 ± 0.003^{h}
SN-BA-027	Z1	62.2 ± 5.5^{h}	3.74 ± 0.34^{i}	0.77 ± 0.06^{i}	16.8 ± 1.3^{h}	11.4 ± 0.6^{g}	0.52 ± 0.05^{f}	1.55 ± 0.11^{h}	0.047 ± 0.003^{h}
SN-BA-029	Z1	70.6 ± 5.4^{gh}	4.57 ± 0.41^{h}	1.04 ± 0.08^{gh}	24.3 ± 1.9^{g}	13.2 ± 0.7^{f}	0.56 ± 0.05^{i}	2.34 ± 0.16^{f}	0.067 ± 0.006^{g}
SN-BA-053	Z1	51.1 ± 4.5^{hi}	3.14 ± 0.22^{j}	0.75 ± 0.06^{i}	21.7 ± 1.3^{g}	13.8 ± 0.9^{f}	0.61 ± 0.05^{g}	2.00 ± 0.11^{g}	0.061 ± 0.005^{g}
SN-BA-061	Z1	27.3 ± 2.1^{j}	2.44 ± 0.17^{j}	0.50 ± 0.03^{j}	16.3 ± 1.3^{h}	12.0 ± 0.9^{g}	0.51 ± 0.05^{f}	1.44 ± 0.11^{h}	0.046 ± 0.003^{h}
SN-BA-062	Z1	28.2 ± 2.1^{j}	2.30 ± 0.18^{jk}	0.51 ± 0.04^{j}	18.1 ± 1.3^{h}	12.5 ± 1.0^{fg}	0.49 ± 0.03^{fg}	1.61 ± 0.11^{h}	0.047 ± 0.003^{h}
SN-BA-063	Z1	52.4 ± 3.8^{hi}	4.32 ± 0.35^{hi}	0.84 ± 0.05^{hi}	17.4 ± 0.9^{h}	12.7 ± 0.7^{fg}	0.59 ± 0.04^{f}	1.89 ± 0.13^{g}	0.056 ± 0.005^{g}
SN-BA-081	Z1	34.1 ± 1.9^{j}	2.98 ± 0.18^{j}	0.66 ± 0.04^{ij}	16.4 ± 1.0^{h}	11.7 ± 0.9^{g}	0.53 ± 0.04^{f}	1.63 ± 0.13^{h}	0.047 ± 0.005^{h}
SN-BA-082	Z1	56.7 ± 4.8^{h}	4.14 ± 0.25^{i}	0.83 ± 0.06^{hi}	22.0 ± 1.2^{g}	14.6 ± 1.0^{f}	0.66 ± 0.04^{e}	2.00 ± 0.15^{g}	0.062 ± 0.006^{g}
SN-BA-083	Z1	60.8 ± 5.0^{h}	3.98 ± 0.24^{i}	0.81 ± 0.07^{i}	24.1 ± 2.0^{g}	13.9 ± 0.8^{f}	0.53 ± 0.04^{f}	1.99 ± 0.11^{g}	0.060 ± 0.005^{g}
SN-BA-084	Z1	68.9 ± 4.7^{gh}	5.26 ± 0.34^{h}	0.88 ± 0.08^{h}	17.5 ± 1.3^{h}	12.7 ± 0.9^{fg}	0.46 ± 0.04^{g}	1.82 ± 0.09^{g}	0.053 ± 0.004^{gh}
SN-BA-003	Z2	102.7 ± 9.2^{ef}	6.78 ± 0.45^{f}	1.24 ± 0.09^{fg}	42.4 ± 2.8^{b}	22.9 ± 1.4^{c}	1.08 ± 0.08^{c}	3.55 ± 0.19^{c}	0.126 ± 0.010^{c}
SN-BA-004	Z2	88.9 ± 7.1^{fg}	7.07 ± 0.46^{f}	1.41 ± 0.09^{f}	43.5 ± 2.4^{b}	23.7 ± 1.7^{bc}	1.12 ± 0.10^{bc}	3.28 ± 0.23^{d}	0.116 ± 0.010^{cd}
SN-BA-014	Z2	114.3 ± 6.6^{e}	6.03 ± 0.34^{g}	1.18 ± 0.10^{g}	46.8 ± 2.9^{ab}	24.8 ± 1.9^{b}	1.16 ± 0.08^{b}	4.30 ± 0.31^{a}	0.144 ± 0.010^{b}
SN-BA-018	Z2	113.2 ± 7.9^{e}	9.53 ± 0.72^{d}	1.63 ± 0.11^{e}	49.5 ± 2.5^{a}	28.8 ± 1.4^{a}	1.45 ± 0.11^{a}	4.65 ± 0.39^{a}	0.160 ± 0.014^{a}
SN-BA-019	Z2	147.9 ± 11.0^{bc}	10.32 ± 0.79^{d}	1.90 ± 0.14^{cd}	45.8 ± 3.2^{b}	25.9 ± 1.9^{b}	1.15 ± 0.08^{b}	4.24 ± 0.23^{ab}	0.143 ± 0.012^{b}
SN-BA-020	Z2	172.3 ± 12.8^{a}	13.99 ± 0.90^{a}	2.52 ± 0.23^{a}	37.9 ± 2.1^{c}	22.6 ± 1.9^{c}	1.14 ± 0.11^{b}	3.88 ± 0.29^{bc}	0.136 ± 0.008^{b}

SN-BA-048	Z2	177.6 ± 12.5^{a}	13.63 ± 1.04^{a}	2.45 ± 0.18^{a}	45.1 ± 3.0^{b}	24.1 ± 2.0^{b}	0.99 ± 0.06^{c}	3.50 ± 0.26^{c}	0.129 ± 0.011^{c}
SN-BA-049	Z2	144.6 ± 11.2^{c}	12.69 ± 1.05^{b}	2.08 ± 0.16^{bc}	42.6 ± 3.5^{b}	23.7 ± 1.7^{bc}	1.04 ± 0.09^{c}	3.90 ± 0.20^{b}	0.124 ± 0.009^{c}
SN-BA-050	Z2	157.4 ± 9.7^{b}	11.51 ± 0.73^{c}	2.33 ± 0.17^{ab}	41.1 ± 2.3^{c}	22.7 ± 1.8^{c}	1.20 ± 0.11^{b}	4.05 ± 0.32^{b}	0.139 ± 0.014^{b}
SN-BA-051	Z2	145.7 ± 10.2^{bc}	12.12 ± 0.80^{b}	2.36 ± 0.21^{a}	47.4 ± 3.0^{a}	23.7 ± 1.2^{bc}	1.01 ± 0.09^{c}	4.06 ± 0.27^{b}	0.136 ± 0.009^{b}
SN-BA-052	Z2	172.0 ± 13.9^{a}	12.33 ± 0.89^{b}	2.41 ± 0.18^{a}	46.6 ± 2.8^{ab}	25.8 ± 2.1^{b}	1.32 ± 0.12^{ab}	4.16 ± 0.23^{b}	0.148 ± 0.010^{b}
SN-BA-054	Z2	157.6 ± 10.9^{b}	12.12 ± 1.09^{b}	1.90 ± 0.12^{cd}	42.2 ± 2.9^{bc}	25.0 ± 2.1^{b}	1.25 ± 0.11^{b}	4.01 ± 0.30^{b}	0.139 ± 0.013^{b}
SN-BA-055	Z2	121.7 ± 9.5^{d}	9.86 ± 0.90^{d}	1.85 ± 0.12^{d}	42.9 ± 3.4^{b}	22.7 ± 1.5^{c}	1.21 ± 0.11^{b}	4.00 ± 0.33^{b}	0.135 ± 0.011^{bc}
SN-BA-056	Z2	141.3 ± 13.2^{c}	11.90 ± 0.78^{b}	2.10 ± 0.14^{bc}	43.1 ± 3.4^{b}	22.3 ± 1.8^{c}	1.02 ± 0.10^{c}	3.53 ± 0.18^{c}	0.119 ± 0.012^{c}
SN-BA-057	Z2	137.1 ± 9.8^{c}	12.35 ± 0.77^{b}	2.47 ± 0.14^{a}	49.5 ± 2.9^{a}	27.5 ± 2.2^{a}	1.34 ± 0.11^{a}	4.36 ± 0.24^{a}	0.148 ± 0.014^{b}
SN-BA-059	Z2	128.3 ± 7.8^{d}	12.38 ± 0.82^{b}	1.79 ± 0.15^{d}	48.6 ± 3.0^{a}	26.5 ± 1.8^{ab}	1.24 ± 0.10^{b}	4.51 ± 0.34^{a}	0.148 ± 0.014^{b}
SN-BA-064	Z2	160.8 ± 11.5^{ab}	12.54 ± 1.07^{b}	2.35 ± 0.16^{a}	50.4 ± 3.0^{a}	26.8 ± 2.2^{a}	1.40 ± 0.09^{a}	4.46 ± 0.26^{a}	0.172 ± 0.016^{a}
SN-BA-065	Z2	113.4 ± 7.6^{e}	10.05 ± 0.68^{d}	1.44 ± 0.08^{ef}	32.7 ± 2.0^{de}	18.9 ± 1.3^{d}	0.82 ± 0.08^{d}	2.70 ± 0.16^{e}	0.100 ± 0.008^{de}
SN-BA-069	Z2	139.5 ± 11.0^{c}	10.54 ± 0.61^{cd}	1.49 ± 0.11^{e}	41.1 ± 2.8^{c}	24.2 ± 1.6^{b}	1.15 ± 0.08^{b}	3.67 ± 0.23^{c}	0.132 ± 0.012^{bc}
SN-BA-085	Z2	161.8 ± 13.4^{ab}	11.68 ± 0.69^{bc}	2.29 ± 0.19^{b}	49.1 ± 3.4^{a}	26.8 ± 1.9^{a}	1.39 ± 0.09^{a}	4.47 ± 0.29^{a}	0.138 ± 0.012^{b}
SN-BA-086	Z2	95.3 ± 8.7^{f}	7.60 ± 0.55^{f}	1.39 ± 0.10^{f}	41.3 ± 2.4^{c}	26.9 ± 1.6^{a}	1.18 ± 0.08^{b}	4.07 ± 0.25^{b}	0.133 ± 0.013^{bc}
SN-BA-087	Z2	126.9 ± 10.0^{d}	10.89 ± 0.92^{c}	1.47 ± 0.08^{ef}	43.6 ± 2.5^{b}	25.9 ± 1.8^{b}	1.19 ± 0.08^{b}	4.28 ± 0.30^{ab}	0.125 ± 0.010^{c}
SN-BA-088	Z2	160.8 ± 13.8^{ab}	13.09 ± 0.77^{a}	1.97 ± 0.19^{c}	40.8 ± 2.8^{c}	23.1 ± 1.5^{c}	1.09 ± 0.10^{c}	3.71 ± 0.25^{c}	0.137 ± 0.009^{b}
SN-BA-094	Z2	115.6 ± 10.2^{de}	9.06 ± 0.51^{e}	1.66 ± 0.11^{de}	42.8 ± 2.9^{b}	22.7 ± 1.9^{c}	1.04 ± 0.08^{c}	3.82 ± 0.27^{bc}	0.123 ± 0.011^{c}
SN-BA-001	Z3	71.6 ± 4.4^{gh}	4.89 ± 0.39^{h}	0.87 ± 0.05^{h}	27.1 ± 2.3^{f}	17.5 ± 0.9^{de}	0.81 ± 0.06^{d}	2.54 ± 0.15^{ef}	0.086 ± 0.008^{ef}
SN-BA-005	Z3	78.4 ± 5.0^{g}	5.18 ± 0.46^{h}	0.78 ± 0.04^{i}	21.2 ± 1.2^{g}	17.0 ± 0.9^{e}	0.76 ± 0.05^{de}	2.88 ± 0.23^{e}	0.092 ± 0.009^{e}
SN-BA-006	Z3	55.4 ± 4.1^{h}	4.87 ± 0.45^{h}	0.69 ± 0.07^{i}	28.5 ± 1.6^{ef}	18.5 ± 1.4^{d}	0.79 ± 0.08^{de}	3.20 ± 0.24^{d}	0.094 ± 0.006^{e}
SN-BA-007	Z3	86.0 ± 6.0^{fg}	6.08 ± 0.49^{g}	0.95 ± 0.09^{h}	18.3 ± 1.0^{h}	11.9 ± 1.0^{g}	0.54 ± 0.05^{f}	2.13 ± 0.15^{fg}	0.067 ± 0.004^{g}
SN-BA-008	Z3	**103.2 ± 6.7^{ef}**	6.53 ± 0.44^{fg}	1.22 ± 0.11^{g}	31.6 ± 2.1^{e}	21.2 ± 1.6^{c}	0.91 ± 0.05^{d}	3.62 ± 0.29^{c}	0.095 ± 0.009^{e}
SN-BA-032	Z3	33.5 ± 2.7^{j}	2.48 ± 0.21^{j}	0.65 ± 0.05^{ij}	16.8 ± 0.9^{h}	8.6 ± 0.6^{h}	0.49 ± 0.04^{fg}	1.68 ± 0.14^{h}	0.053 ± 0.005^{gh}
SN-BA-033	Z3	60.4 ± 4.9^{h}	5.07 ± 0.43^{h}	0.79 ± 0.06^{i}	17.4 ± 1.2^{h}	13.4 ± 1.0^{f}	0.62 ± 0.05^{f}	2.00 ± 0.13^{g}	0.067 ± 0.006^{g}
SN-BA-034	Z3	79.5 ± 5.2^{g}	5.58 ± 0.52^{gh}	1.00 ± 0.07^{h}	23.1 ± 1.7^{g}	15.4 ± 1.2^{ef}	0.61 ± 0.04^{f}	2.75 ± 0.23^{e}	0.078 ± 0.005^{f}
SN-BA-035	Z3	113.7 ± 6.3^{e}	6.94 ± 0.51^{f}	1.09 ± 0.10^{g}	25.9 ± 1.5^{f}	15.8 ± 1.1^{e}	0.77 ± 0.08^{de}	2.98 ± 0.20^{de}	0.079 ± 0.006^{f}
SN-BA-036	Z3	**99.2 ± 8.2^{f}**	8.53 ± 0.80^{e}	1.46 ± 0.10^{ef}	29.9 ± 1.9^{e}	18.6 ± 1.2^{d}	0.89 ± 0.09^{d}	3.46 ± 0.23^{cd}	0.092 ± 0.008^{e}

SN-BA-037	Z3	112.7 ± 9.7^{e}	8.37 ± 0.53^{e}	1.13 ± 0.10^{g}	29.7 ± 1.8^{e}	16.8 ± 0.8^{e}	0.84 ± 0.06^{d}	2.82 ± 0.20^{e}	0.099 ± 0.010^{e}
SN-BA-038	Z3	72.3 ± 6.2^{gh}	5.73 ± 0.33^{g}	0.98 ± 0.09^{h}	25.8 ± 1.6^{f}	18.0 ± 1.0^{d}	0.78 ± 0.08^{de}	3.09 ± 0.19^{d}	0.080 ± 0.007^{f}
SN-BA-039	Z3	61.2 ± 3.7^{h}	4.90 ± 0.38^{h}	0.86 ± 0.08^{hi}	23.2 ± 1.3^{g}	15.0 ± 1.2^{ef}	0.78 ± 0.07^{de}	2.34 ± 0.19^{f}	0.082 ± 0.006^{f}
SN-BA-040	Z3	56.1 ± 4.0^{h}	4.82 ± 0.28^{h}	0.90 ± 0.06^{h}	22.1 ± 1.6^{g}	12.0 ± 0.7^{g}	0.73 ± 0.06^{e}	2.29 ± 0.15^{f}	0.083 ± 0.006^{f}
SN-BA-041	Z3	74.3 ± 6.8^{gh}	5.45 ± 0.31^{gh}	0.71 ± 0.05^{i}	23.5 ± 1.9^{g}	13.9 ± 1.0^{f}	0.68 ± 0.07^{e}	2.51 ± 0.18^{f}	0.078 ± 0.006^{f}
SN-BA-058	Z3	54.5 ± 4.5^{hi}	3.42 ± 0.32^{ij}	0.74 ± 0.06^{i}	20.9 ± 1.7^{g}	12.8 ± 0.8^{fg}	0.61 ± 0.06^{f}	1.94 ± 0.16^{g}	0.068 ± 0.006^{fg}
SN-BA-060	Z3	106.9 ± 8.1^{e}	6.98 ± 0.58^{f}	1.35 ± 0.12^{f}	27.2 ± 1.9^{f}	16.1 ± 1.1^{e}	0.80 ± 0.07^{d}	2.87 ± 0.23^{e}	0.090 ± 0.009^{e}
SN-BA-089	Z3	48.3 ± 3.8^{hi}	3.95 ± 0.26^{i}	0.87 ± 0.06^{hi}	22.5 ± 1.9^{g}	13.6 ± 1.0^{f}	0.68 ± 0.06^{e}	2.09 ± 0.15^{g}	0.079 ± 0.006^{f}
SN-BA-090	Z3	131.7 ± 10.7^{cd}	10.86 ± 0.77^{c}	1.58 ± 0.14^{e}	36.9 ± 2.4^{d}	22.7 ± 1.3^{c}	1.04 ± 0.10^{c}	4.35 ± 0.29^{a}	0.112 ± 0.008^{d}
SN-BA-091	Z3	58.7 ± 3.3^{h}	5.54 ± 0.39^{gh}	1.01 ± 0.08^{h}	24.3 ± 1.8^{g}	16.7 ± 1.2^{e}	0.78 ± 0.07^{de}	2.70 ± 0.19^{e}	0.082 ± 0.007^{f}
SN-BA-002	Z4	42.1 ± 2.4^{i}	3.37 ± 0.30^{ij}	0.51 ± 0.04^{j}	15.8 ± 1.1^{hi}	10.4 ± 0.7^{gh}	0.35 ± 0.03^{g}	1.62 ± 0.10^{h}	0.039 ± 0.004^{hi}
SN-BA-009	Z4	24.4 ± 1.7^{jk}	1.72 ± 0.12^{k}	0.39 ± 0.03^{k}	13.6 ± 0.7^{i}	10.0 ± 0.8^{gh}	0.32 ± 0.02^{gh}	1.17 ± 0.06^{i}	0.031 ± 0.002^{i}
SN-BA-010	Z4	41.5 ± 3.6^{i}	3.16 ± 0.26^{j}	0.58 ± 0.03^{j}	15.0 ± 0.8^{i}	8.7 ± 0.5^{h}	0.31 ± 0.02^{h}	1.17 ± 0.06^{i}	0.033 ± 0.002^{i}
SN-BA-011	Z4	48.8 ± 3.0^{hi}	4.04 ± 0.30^{i}	0.55 ± 0.03^{j}	15.5 ± 1.0^{hi}	8.8 ± 0.5^{h}	0.35 ± 0.03^{g}	1.53 ± 0.11^{h}	0.037 ± 0.003^{hi}
SN-BA-012	Z4	45.5 ± 3.7^{i}	3.12 ± 0.21^{j}	0.61 ± 0.04^{j}	16.2 ± 1.0^{hi}	8.2 ± 0.5^{h}	0.34 ± 0.03^{gh}	1.39 ± 0.11^{h}	0.041 ± 0.003^{h}
SN-BA-013	Z4	25.8 ± 2.4^{jk}	2.02 ± 0.12^{k}	0.36 ± 0.02^{k}	15.0 ± 0.9^{i}	9.5 ± 0.6^{h}	0.31 ± 0.02^{gh}	1.39 ± 0.08^{h}	0.035 ± 0.003^{i}
SN-BA-015	Z4	36.4 ± 2.2^{ij}	2.52 ± 0.19^{j}	0.40 ± 0.03^{k}	15.4 ± 1.3^{hi}	7.8 ± 0.4^{hi}	0.35 ± 0.02^{g}	1.46 ± 0.12^{h}	0.034 ± 0.003^{i}
SN-BA-016	Z4	32.5 ± 2.5^{j}	3.30 ± 0.22^{ij}	0.53 ± 0.03^{j}	13.6 ± 0.7^{i}	8.5 ± 0.6^{h}	0.31 ± 0.02^{h}	1.14 ± 0.06^{i}	0.034 ± 0.003^{i}
SN-BA-017	Z4	33.8 ± 3.0^{j}	2.56 ± 0.15^{j}	0.57 ± 0.05^{j}	15.4 ± 1.0^{hi}	10.1 ± 0.7^{gh}	0.33 ± 0.03^{gh}	1.34 ± 0.08^{h}	0.034 ± 0.002^{i}
SN-BA-021	Z4	27.6 ± 1.7^{j}	1.79 ± 0.14^{k}	0.41 ± 0.03^{k}	13.0 ± 1.0^{i}	6.7 ± 0.5^{i}	0.26 ± 0.02^{h}	1.04 ± 0.06^{i}	0.023 ± 0.002^{i}
SN-BA-024	Z4	33.6 ± 2.1^{j}	2.31 ± 0.15^{jk}	0.37 ± 0.02^{k}	14.5 ± 0.9^{i}	8.9 ± 0.7^{h}	0.36 ± 0.03^{g}	1.44 ± 0.12^{h}	0.037 ± 0.003^{hi}
SN-BA-025	Z4	33.3 ± 2.8^{j}	2.64 ± 0.21^{j}	0.59 ± 0.04^{j}	15.0 ± 1.0^{i}	8.4 ± 0.7^{h}	0.35 ± 0.03^{g}	1.33 ± 0.07^{h}	0.038 ± 0.003^{hi}
SN-BA-028	Z4	**39.3 ± 2.4^{ij}**	2.74 ± 0.16^{j}	0.56 ± 0.04^{j}	15.2 ± 1.3^{i}	9.5 ± 0.6^{h}	0.30 ± 0.02^{h}	1.37 ± 0.09^{h}	0.035 ± 0.003^{i}
SN-BA-030	Z4	**21.5 ± 1.9^{k}**	1.40 ± 0.13^{k}	0.36 ± 0.03^{k}	14.1 ± 1.2^{i}	8.2 ± 0.6^{h}	0.30 ± 0.02^{h}	1.08 ± 0.08^{i}	0.028 ± 0.003^{i}
SN-BA-031	Z4	36.7 ± 3.0^{ij}	2.59 ± 0.24^{j}	0.46 ± 0.03^{jk}	15.4 ± 1.0^{hi}	9.2 ± 0.7^{h}	0.36 ± 0.04^{g}	1.24 ± 0.10^{hi}	0.033 ± 0.002^{i}
SN-BA-070	Z4	34.8 ± 3.1^{j}	2.45 ± 0.18^{j}	0.42 ± 0.04^{k}	15.2 ± 1.1^{i}	10.6 ± 0.7^{g}	0.35 ± 0.02^{gh}	1.44 ± 0.11^{h}	0.038 ± 0.003^{hi}
SN-BA-071	Z4	54.4 ± 4.3^{hi}	4.40 ± 0.25^{hi}	0.75 ± 0.05^{i}	16.1 ± 1.4^{hi}	10.1 ± 0.5^{gh}	0.39 ± 0.02^{g}	1.42 ± 0.12^{h}	0.043 ± 0.003^{h}
SN-BA-072	Z4	29.2 ± 2.0^{j}	2.61 ± 0.16^{j}	0.49 ± 0.03^{j}	15.6 ± 1.3^{hi}	9.7 ± 0.7^{h}	0.36 ± 0.03^{g}	1.62 ± 0.14^{h}	0.038 ± 0.003^{hi}

SN-BA-042	Z5	90.5 ± 8.1^{f}	7.14 ± 0.46^{f}	1.04 ± 0.07^{gh}	26.7 ± 1.5^{f}	13.8 ± 1.1^{f}	0.77 ± 0.06^{de}	2.41 ± 0.13^{f}	0.080 ± 0.007^{f}
SN-BA-043	Z5	64.4 ± 5.6^{h}	4.51 ± 0.39^{hi}	0.86 ± 0.05^{hi}	23.8 ± 1.7^{g}	13.7 ± 0.9^{f}	0.73 ± 0.06^{e}	2.30 ± 0.17^{f}	0.073 ± 0.005^{f}
SN-BA-044	Z5	107.2 ± 6.8^{e}	8.54 ± 0.55^{e}	1.64 ± 0.15^{de}	31.8 ± 2.2^{e}	18.0 ± 1.4^{de}	0.89 ± 0.06^{d}	2.95 ± 0.19^{e}	0.097 ± 0.006^{e}
SN-BA-045	Z5	96.6 ± 8.2^{f}	6.09 ± 0.36^{g}	0.95 ± 0.08^{h}	31.1 ± 1.8^{e}	15.9 ± 1.2^{e}	0.87 ± 0.08^{d}	2.83 ± 0.17^{e}	0.092 ± 0.008^{e}
SN-BA-046	Z5	81.0 ± 6.9^{g}	5.08 ± 0.34^{h}	0.92 ± 0.08^{h}	32.0 ± 1.8^{e}	19.7 ± 1.1^{d}	0.80 ± 0.05^{d}	3.10 ± 0.23^{d}	0.100 ± 0.008^{de}
SN-BA-047	Z5	116.1 ± 10.8^{de}	10.80 ± 0.96^{c}	1.19 ± 0.10^{g}	33.1 ± 2.3^{de}	19.0 ± 1.2^{d}	0.96 ± 0.08^{cd}	3.41 ± 0.26^{cd}	0.101 ± 0.006^{de}
SN-BA-066	Z5	81.6 ± 5.3^{g}	6.53 ± 0.49^{fg}	0.80 ± 0.05^{i}	27.5 ± 1.8^{f}	13.7 ± 0.8^{f}	0.77 ± 0.07^{de}	2.69 ± 0.23^{e}	0.084 ± 0.008^{ef}
SN-BA-067	Z5	104.3 ± 8.2^{ef}	8.64 ± 0.82^{e}	1.14 ± 0.09^{g}	27.1 ± 2.0^{f}	14.1 ± 1.0^{f}	0.70 ± 0.07^{e}	2.66 ± 0.20^{e}	0.073 ± 0.006^{f}
SN-BA-068	Z5	103.4 ± 6.4^{ef}	6.77 ± 0.58^{f}	1.68 ± 0.12^{de}	32.4 ± 1.9^{e}	18.9 ± 1.2^{d}	1.00 ± 0.09^{c}	3.54 ± 0.29^{c}	0.094 ± 0.006^{e}
SN-BA-073	Z5	68.6 ± 5.2^{gh}	4.83 ± 0.40^{h}	0.57 ± 0.04^{j}	31.7 ± 2.5^{e}	17.7 ± 1.3^{de}	0.88 ± 0.07^{d}	3.15 ± 0.21^{d}	0.086 ± 0.008^{ef}
SN-BA-074	Z5	82.1 ± 5.6^{g}	5.08 ± 0.35^{h}	0.65 ± 0.06^{ij}	28.9 ± 1.5^{ef}	15.2 ± 1.2^{ef}	0.74 ± 0.07^{e}	2.84 ± 0.20^{e}	0.084 ± 0.008^{ef}
SN-BA-075	Z5	124.1 ± 8.8^{d}	8.66 ± 0.50^{e}	1.39 ± 0.11^{f}	31.0 ± 1.7^{e}	20.1 ± 1.7^{d}	1.00 ± 0.08^{c}	3.32 ± 0.27^{d}	0.093 ± 0.007^{e}
SN-BA-076	Z5	75.0 ± 5.1^{g}	5.80 ± 0.39^{g}	0.70 ± 0.06^{i}	32.4 ± 1.8^{e}	19.6 ± 1.2^{d}	0.83 ± 0.08^{d}	3.34 ± 0.28^{d}	0.092 ± 0.006^{e}
SN-BA-077	Z5	110.6 ± 8.7^{e}	7.21 ± 0.46^{f}	1.39 ± 0.10^{f}	28.2 ± 2.1^{f}	15.4 ± 1.1^{e}	0.77 ± 0.06^{de}	2.51 ± 0.20^{f}	0.088 ± 0.008^{e}
SN-BA-078	Z5	123.7 ± 11.0^{d}	9.61 ± 0.54^{d}	1.64 ± 0.15^{de}	32.0 ± 2.5^{e}	18.4 ± 1.0^{d}	0.78 ± 0.06^{de}	2.82 ± 0.23^{e}	0.091 ± 0.008^{e}
SN-BA-079	Z5	155.9 ± 8.6^{b}	10.97 ± 0.87^{c}	1.96 ± 0.17^{c}	35.4 ± 2.3^{d}	18.7 ± 1.5^{d}	0.97 ± 0.07^{c}	3.67 ± 0.28^{c}	0.104 ± 0.010^{d}
SN-BA-080	Z5	91.5 ± 6.0^{f}	6.90 ± 0.63^{f}	1.16 ± 0.11^{g}	32.8 ± 2.0^{de}	18.0 ± 1.2^{de}	0.81 ± 0.06^{d}	2.93 ± 0.21^{e}	0.090 ± 0.007^{e}
SN-BA-092	Z5	130.3 ± 10.3^{cd}	8.46 ± 0.59^{e}	1.48 ± 0.13^{ef}	34.4 ± 2.6^{d}	20.0 ± 1.6^{d}	0.88 ± 0.09^{d}	3.11 ± 0.24^{d}	0.107 ± 0.008^{d}
SN-BA-093	Z5	91.4 ± 8.5^{f}	7.42 ± 0.48^{f}	1.42 ± 0.08^{f}	26.4 ± 2.1^{f}	14.0 ± 0.8^{f}	0.70 ± 0.05^{e}	2.54 ± 0.20^{ef}	0.077 ± 0.007^{f}

Notes: Values are reported as mean ± analytical SD of three independently prepared analytical portions on a dry-weight basis (mg/kg DW). The three analytical measurements were used only to assess analytical precision and were averaged to obtain one site-level concentration per matrix and georeferenced sampling site for subsequent statistical analyses. Values <LOD and <LOQ are indicated as applicable.

Table S18. Individual concentrations of potentially toxic and environmentally relevant trace elements in *Sambucus nigra* L. bark samples collected at georeferenced sampling points across the ecological sectors of the Oradea metropolitan area, Romania.

Sample ID	Zone	^{208}Pb (mg/kg)	^{111}Cd (mg/kg)	^{75}As (mg/kg)	^{202}Hg (mg/kg)	^{52}Cr (mg/kg)	^{60}Ni (mg/kg)	^{121}Sb (mg/kg)	^{118}Sn (mg/kg)
SN-BA-022	Z1	<LOD	<LOD	<LOD	<LOD	0.19 ± 0.01^{h}	0.31 ± 0.02^{g}	<LOD	0.013 ± 0.001^{g}
SN-BA-023	Z1	<LOD	<LOD	<LOD	<LOD	0.57 ± 0.05^{g}	0.27 ± 0.03^{g}	<LOD	0.017 ± 0.001^{g}

SN-BA-026	Z1	<LOD	<LOD	<LOD	<LOD	0.58 ± 0.04^{g}	0.29 ± 0.02^{g}	<LOD	0.018 ± 0.002^{g}
SN-BA-027	Z1	<LOD	<LOD	<LOD	<LOD	0.49 ± 0.05^{g}	0.37 ± 0.02^{g}	<LOD	0.016 ± 0.001^{g}
SN-BA-029	Z1	<LOD	<LOD	<LOD	<LOD	0.42 ± 0.03^{g}	0.39 ± 0.04^{g}	<LOD	<LOD
SN-BA-053	Z1	<LOD	<LOD	<LOD	<LOD	0.44 ± 0.03^{g}	0.53 ± 0.05^{f}	<LOD	0.021 ± 0.002^{g}
SN-BA-061	Z1	<LOD	<LOD	<LOD	<LOD	0.33 ± 0.04^{h}	0.19 ± 0.01^{g}	<LOD	<LOD
SN-BA-062	Z1	<LOD	<LOD	<LOD	<LOD	0.50 ± 0.05^{g}	0.45 ± 0.03^{f}	<LOD	0.020 ± 0.002^{g}
SN-BA-063	Z1	<LOD	<LOD	<LOD	<LOD	0.56 ± 0.04^{g}	0.27 ± 0.02^{g}	<LOD	<LOD
SN-BA-081	Z1	0.103 ± 0.008^{g}	<LOD	<LOD	<LOD	0.31 ± 0.03^{h}	0.39 ± 0.04^{g}	<LOD	<LOD
SN-BA-082	Z1	<LOD	<LOD	<LOD	<LOD	0.30 ± 0.03^{h}	0.26 ± 0.01^{g}	<LOD	0.022 ± 0.001^{g}
SN-BA-083	Z1	<LOD	<LOD	<LOD	<LOD	0.25 ± 0.02^{h}	0.35 ± 0.04^{g}	<LOD	0.009 ± 0.001^{h}
SN-BA-084	Z1	<LOD	<LOD	0.019 ± 0.002^{f}	<LOD	0.58 ± 0.04^{g}	0.18 ± 0.01^{h}	<LOD	0.017 ± 0.001^{g}
SN-BA-003	Z2	0.599 ± 0.052^{a}	0.0181 ± 0.0019^{e}	0.017 ± 0.002^{f}	<LOD	2.05 ± 0.20^{a}	1.47 ± 0.15^{b}	0.0331 ± 0.0030^{b}	0.043 ± 0.005^{e}
SN-BA-004	Z2	0.363 ± 0.023^{d}	0.0441 ± 0.0033^{c}	0.095 ± 0.009^{a}	<LOD	1.15 ± 0.12^{d}	1.30 ± 0.12^{c}	0.0382 ± 0.0023^{a}	0.023 ± 0.002^{f}
SN-BA-014	Z2	<LOD	0.0356 ± 0.0026^{c}	0.029 ± 0.003^{e}	0.0039 ± 0.0004^{b}	1.01 ± 0.08^{e}	1.20 ± 0.08^{c}	<LOD	0.070 ± 0.004^{c}
SN-BA-018	Z2	0.446 ± 0.034^{c}	0.0551 ± 0.0035^{b}	<LOD	<LOD	0.86 ± 0.08^{e}	0.68 ± 0.04^{e}	<LOD	0.086 ± 0.008^{a}
SN-BA-019	Z2	0.079 ± 0.009^{g}	0.0233 ± 0.0015^{e}	0.015 ± 0.001^{g}	0.0025 ± 0.0002^{d}	1.88 ± 0.11^{a}	1.44 ± 0.14^{b}	0.0177 ± 0.0012^{d}	0.039 ± 0.003^{e}
SN-BA-020	Z2	<LOD	0.0495 ± 0.0051^{b}	0.024 ± 0.002^{f}	<LOD	**1.06 ± 0.10^{d}**	0.98 ± 0.08^{d}	0.0067 ± 0.0005^{f}	0.026 ± 0.002^{f}
SN-BA-048	Z2	0.402 ± 0.026^{c}	0.0478 ± 0.0050^{b}	<LOD	<LOD	1.53 ± 0.10^{c}	1.39 ± 0.14^{b}	<LOD	0.084 ± 0.007^{a}
SN-BA-049	Z2	0.457 ± 0.036^{c}	0.0331 ± 0.0021^{d}	0.042 ± 0.005^{d}	0.0061 ± 0.0007^{a}	1.11 ± 0.13^{d}	0.72 ± 0.05^{e}	0.0426 ± 0.0024^{a}	<LOD
SN-BA-050	Z2	0.486 ± 0.037^{b}	0.0404 ± 0.0026^{c}	0.065 ± 0.005^{b}	0.0028 ± 0.0003^{c}	0.76 ± 0.07^{f}	1.85 ± 0.20^{a}	0.0401 ± 0.0034^{a}	0.022 ± 0.002^{g}
SN-BA-051	Z2	0.344 ± 0.026^{d}	0.0504 ± 0.0058^{b}	0.033 ± 0.003^{e}	<LOD	1.64 ± 0.14^{b}	0.48 ± 0.04^{f}	<LOD	0.062 ± 0.007^{c}
SN-BA-052	Z2	0.186 ± 0.015^{f}	0.0377 ± 0.0031^{c}	0.053 ± 0.003^{c}	<LOD	1.26 ± 0.08^{d}	0.68 ± 0.06^{e}	0.0164 ± 0.0011^{e}	0.092 ± 0.009^{a}
SN-BA-054	Z2	0.615 ± 0.034^{a}	0.0660 ± 0.0053^{a}	0.029 ± 0.002^{e}	0.0035 ± 0.0003^{c}	2.00 ± 0.16^{a}	1.85 ± 0.12^{a}	0.0176 ± 0.0012^{d}	0.030 ± 0.003^{f}
SN-BA-055	Z2	0.094 ± 0.007^{g}	<LOD	0.031 ± 0.003^{e}	<LOD	0.76 ± 0.05^{f}	1.42 ± 0.09^{b}	0.0078 ± 0.0005^{f}	0.018 ± 0.002^{g}
SN-BA-056	Z2	0.162 ± 0.012^{f}	0.0462 ± 0.0037^{b}	0.048 ± 0.005^{c}	<LOD	1.73 ± 0.16^{b}	1.68 ± 0.15^{a}	0.0174 ± 0.0016^{d}	0.040 ± 0.003^{e}
SN-BA-057	Z2	0.357 ± 0.033^{d}	0.0581 ± 0.0043^{a}	0.050 ± 0.003^{c}	0.0034 ± 0.0002^{c}	1.09 ± 0.09^{d}	1.76 ± 0.14^{a}	0.0341 ± 0.0031^{b}	0.075 ± 0.004^{b}
SN-BA-059	Z2	0.174 ± 0.016^{f}	<LOD	0.017 ± 0.001^{f}	<LOD	1.27 ± 0.11^{d}	1.73 ± 0.18^{a}	0.0266 ± 0.0022^{c}	0.019 ± 0.001^{g}
SN-BA-064	Z2	0.250 ± 0.027^{e}	0.0262 ± 0.0027^{d}	0.020 ± 0.001^{f}	<LOD	1.79 ± 0.19^{b}	1.49 ± 0.12^{b}	0.0350 ± 0.0022^{b}	0.083 ± 0.005^{b}

SN-BA-065	Z2	0.477 ± 0.040[b]	0.0128 ± 0.0012[f]	0.069 ± 0.007[b]	<LOD	1.67 ± 0.18[b]	0.85 ± 0.09[d]	0.0157 ± 0.0018[e]	0.082 ± 0.006[b]
SN-BA-069	Z2	0.447 ± 0.049[c]	<LOD	<LOD	<LOD	1.51 ± 0.16[c]	0.76 ± 0.05[e]	0.0206 ± 0.0014[d]	0.066 ± 0.006[c]
SN-BA-085	Z2	0.324 ± 0.030[d]	0.0126 ± 0.0007[f]	<LOD	<LOD	1.13 ± 0.07[d]	1.58 ± 0.17[b]	0.0345 ± 0.0031[b]	0.085 ± 0.008[a]
SN-BA-086	Z2	0.514 ± 0.029[b]	0.0214 ± 0.0012[e]	0.054 ± 0.004[c]	<LOD	1.78 ± 0.18[b]	1.53 ± 0.11[b]	<LOD	0.054 ± 0.003[d]
SN-BA-087	Z2	0.198 ± 0.013[f]	0.0657 ± 0.0057[a]	<LOD	<LOD	1.18 ± 0.13[d]	1.85 ± 0.16[a]	0.0257 ± 0.0023[c]	0.093 ± 0.010[a]
SN-BA-088	Z2	0.343 ± 0.024[d]	0.0287 ± 0.0030[d]	0.027 ± 0.003[e]	0.0047 ± 0.0003[b]	1.43 ± 0.09[c]	1.75 ± 0.18[a]	0.0253 ± 0.0026[c]	0.037 ± 0.003[e]
SN-BA-094	Z2	0.527 ± 0.058[b]	0.0621 ± 0.0069[a]	0.034 ± 0.002[e]	<LOD	1.96 ± 0.16[a]	1.85 ± 0.17[a]	0.0284 ± 0.0020[c]	0.028 ± 0.002[f]
SN-BA-001	Z3	0.105 ± 0.007[g]	0.0206 ± 0.0018[e]	<LOD	<LOD	0.64 ± 0.07[f]	0.76 ± 0.07[e]	<LOD	0.011 ± 0.001[h]
SN-BA-005	Z3	<LOD	0.0180 ± 0.0013[e]	<LOD	<LOD	0.33 ± 0.02[h]	0.54 ± 0.06[f]	<LOD	0.009 ± 0.001[h]
SN-BA-006	Z3	<LOD	<LOD	0.029 ± 0.002[e]	0.0025 ± 0.0003[d]	0.75 ± 0.06[f]	0.59 ± 0.04[f]	<LOD	0.037 ± 0.003[e]
SN-BA-007	Z3	<LOD	0.0202 ± 0.0013[e]	<LOD	<LOD	0.75 ± 0.08[f]	0.37 ± 0.03[g]	<LOD	0.032 ± 0.004[f]
SN-BA-008	Z3	<LOD	<LOD	0.028 ± 0.002[e]	<LOD	0.85 ± 0.07[e]	0.22 ± 0.02[g]	<LOD	0.043 ± 0.003[e]
SN-BA-032	Z3	<LOD	<LOD	0.030 ± 0.003[e]	<LOD	0.26 ± 0.02[h]	0.73 ± 0.05[e]	<LOD	<LOD
SN-BA-033	Z3	0.125 ± 0.007[g]	<LOD	<LOD	<LOD	0.38 ± 0.04[h]	0.60 ± 0.07[f]	0.0097 ± 0.0006[f]	0.021 ± 0.002[g]
SN-BA-034	Z3	<LOD	0.0205 ± 0.0019[e]	<LOD	<LOD	0.93 ± 0.07[e]	0.50 ± 0.03[f]	0.0084 ± 0.0005[f]	<LOD
SN-BA-035	Z3	0.094 ± 0.008[g]	<LOD	<LOD	0.0024 ± 0.0002[d]	**1.07 ± 0.10[d]**	0.33 ± 0.03[g]	0.0132 ± 0.0012[e]	0.010 ± 0.001[h]
SN-BA-036	Z3	<LOD	<LOD	<LOD	<LOD	0.34 ± 0.04[h]	0.92 ± 0.09[d]	<LOD	0.025 ± 0.002[f]
SN-BA-037	Z3	0.179 ± 0.015[f]	0.0198 ± 0.0023[e]	<LOD	<LOD	0.40 ± 0.05[g]	0.82 ± 0.05[e]	0.0056 ± 0.0006[f]	0.045 ± 0.004[e]
SN-BA-038	Z3	<LOD	<LOD	<LOD	<LOD	0.24 ± 0.01[h]	0.55 ± 0.04[f]	<LOD	<LOD
SN-BA-039	Z3	0.116 ± 0.013[g]	<LOD	<LOD	<LOD	0.81 ± 0.09[f]	1.01 ± 0.07[d]	0.0138 ± 0.0009[e]	0.047 ± 0.003[d]
SN-BA-040	Z3	<LOD	<LOD	<LOD	<LOD	0.46 ± 0.05[g]	0.82 ± 0.09[e]	<LOD	0.030 ± 0.002[f]
SN-BA-041	Z3	0.216 ± 0.018[e]	<LOD	<LOD	<LOD	0.55 ± 0.04[g]	0.37 ± 0.03[g]	0.0080 ± 0.0007[f]	0.043 ± 0.003[e]
SN-BA-058	Z3	<LOD	<LOD	0.012 ± 0.001[g]	<LOD	0.80 ± 0.09[f]	0.34 ± 0.04[g]	<LOD	<LOD
SN-BA-060	Z3	0.273 ± 0.026[e]	<LOD	<LOD	<LOD	1.04 ± 0.10[e]	0.62 ± 0.04[f]	0.0082 ± 0.0009[f]	0.012 ± 0.001[h]
SN-BA-089	Z3	<LOD	<LOD	<LOD	<LOD	0.57 ± 0.04[g]	0.62 ± 0.07[e]	<LOD	0.023 ± 0.002[f]
SN-BA-090	Z3	0.066 ± 0.007[g]	<LOD	<LOD	<LOD	0.57 ± 0.05[g]	0.68 ± 0.06[e]	<LOD	0.045 ± 0.004[e]
SN-BA-091	Z3	0.122 ± 0.011[g]	<LOD	<LOD	<LOD	0.44 ± 0.03[g]	0.49 ± 0.04[f]	0.0056 ± 0.0004[f]	0.038 ± 0.004[e]
SN-BA-002	Z4	<LOD	<LOD	<LOD	<LOD	0.39 ± 0.03[g]	0.20 ± 0.02[g]	<LOD	<LOD

SN-BA-009	Z4	<LOD	<LOD	<LOD	<LOD	0.21 ± 0.01^{h}	0.28 ± 0.02^{g}	<LOD	<LOD
SN-BA-010	Z4	<LOD	<LOD	<LOD	<LOD	0.14 ± 0.01^{i}	0.21 ± 0.01^{g}	<LOD	0.008 ± 0.001^{h}
SN-BA-011	Z4	<LOD	<LOD	<LOD	<LOD	0.30 ± 0.02^{h}	0.11 ± 0.01^{h}	<LOD	<LOD
SN-BA-012	Z4	<LOD	<LOD	<LOD	<LOD	0.31 ± 0.03^{h}	0.22 ± 0.02^{g}	<LOD	0.011 ± 0.001^{h}
SN-BA-013	Z4	<LOD	<LOD	<LOD	<LOD	0.16 ± 0.02^{i}	0.35 ± 0.02^{g}	<LOD	<LOD
SN-BA-015	Z4	<LOD	<LOD	<LOD	<LOD	0.14 ± 0.02^{i}	0.36 ± 0.03^{g}	<LOD	0.007 ± 0.001^{h}
SN-BA-016	Z4	<LOD	<LOD	<LOD	<LOD	0.43 ± 0.05^{g}	0.25 ± 0.02^{g}	<LOD	<LOD
SN-BA-017	Z4	<LOD	<LOD	<LOD	<LOD	0.31 ± 0.04^{h}	0.16 ± 0.01^{h}	<LOD	0.010 ± 0.001^{h}
SN-BA-021	Z4	<LOD	<LOD	<LOD	<LOD	0.33 ± 0.03^{h}	0.20 ± 0.02^{g}	<LOD	0.018 ± 0.002^{g}
SN-BA-024	Z4	<LOD	<LOD	<LOD	<LOD	0.26 ± 0.03^{h}	0.23 ± 0.02^{g}	<LOD	<LOD
SN-BA-025	Z4	<LOD	<LOD	<LOD	<LOD	0.39 ± 0.04^{g}	0.30 ± 0.03^{g}	<LOD	<LOD
SN-BA-028	Z4	<LOD	<LOD	<LOD	<LOD	0.27 ± 0.02^{h}	0.10 ± 0.01^{h}	<LOD	0.010 ± 0.001^{h}
SN-BA-030	Z4	<LOD	<LOD	<LOD	<LOD	0.25 ± 0.02^{h}	0.39 ± 0.04^{g}	<LOD	<LOD
SN-BA-031	Z4	<LOD	<LOD	<LOD	<LOD	0.17 ± 0.01^{i}	0.27 ± 0.03^{g}	<LOD	<LOD
SN-BA-070	Z4	<LOD	<LOD	<LOD	<LOD	0.11 ± 0.01^{i}	0.39 ± 0.02^{g}	<LOD	<LOD
SN-BA-071	Z4	<LOD	<LOD	<LOD	<LOD	0.28 ± 0.02^{h}	0.11 ± 0.01^{h}	<LOD	0.012 ± 0.001^{h}
SN-BA-072	Z4	<LOD	<LOD	<LOD	<LOD	0.34 ± 0.03^{h}	0.36 ± 0.03^{g}	<LOD	0.009 ± 0.001^{h}
SN-BA-042	Z5	0.312 ± 0.035^{d}	<LOD	<LOD	<LOD	0.59 ± 0.03^{g}	0.81 ± 0.05^{e}	<LOD	0.029 ± 0.003^{f}
SN-BA-043	Z5	<LOD	<LOD	<LOD	0.0034 ± 0.0003^{c}	0.92 ± 0.06^{e}	0.85 ± 0.06^{d}	<LOD	0.024 ± 0.003^{f}
SN-BA-044	Z5	0.162 ± 0.017^{f}	<LOD	<LOD	<LOD	0.51 ± 0.03^{g}	1.16 ± 0.10^{c}	0.0117 ± 0.0011^{e}	0.063 ± 0.004^{c}
SN-BA-045	Z5	0.074 ± 0.007^{g}	<LOD	<LOD	<LOD	0.49 ± 0.04^{g}	0.50 ± 0.04^{f}	<LOD	0.056 ± 0.006^{d}
SN-BA-046	Z5	<LOD	<LOD	0.018 ± 0.002^{f}	<LOD	0.50 ± 0.03^{g}	0.76 ± 0.09^{e}	<LOD	0.025 ± 0.001^{f}
SN-BA-047	Z5	0.161 ± 0.015^{f}	0.0303 ± 0.0033^{d}	0.017 ± 0.001^{f}	<LOD	0.88 ± 0.06^{e}	0.68 ± 0.05^{e}	0.0182 ± 0.0013^{d}	<LOD
SN-BA-066	Z5	0.211 ± 0.021^{e}	<LOD	<LOD	<LOD	0.57 ± 0.06^{g}	0.74 ± 0.04^{e}	<LOD	0.065 ± 0.005^{c}
SN-BA-067	Z5	<LOD	<LOD	<LOD	<LOD	0.59 ± 0.05^{g}	0.86 ± 0.05^{d}	<LOD	0.027 ± 0.002^{f}
SN-BA-068	Z5	0.274 ± 0.016^{e}	0.0155 ± 0.0014^{e}	0.034 ± 0.002^{e}	<LOD	1.12 ± 0.06^{d}	1.30 ± 0.08^{c}	0.0170 ± 0.0018^{d}	0.067 ± 0.007^{c}
SN-BA-073	Z5	<LOD	<LOD	<LOD	<LOD	0.70 ± 0.08^{f}	0.82 ± 0.05^{e}	<LOD	0.020 ± 0.002^{g}
SN-BA-074	Z5	<LOD	<LOD	<LOD	<LOD	0.86 ± 0.09^{e}	0.56 ± 0.06^{f}	0.0143 ± 0.0014^{e}	<LOD

SN-BA-075	Z5	0.155 ± 0.011^{f}	0.0267 ± 0.0023^{d}	0.043 ± 0.005^{d}	0.0029 ± 0.0003^{c}	0.65 ± 0.05^{f}	0.29 ± 0.03^{g}	0.0173 ± 0.0015^{d}	0.062 ± 0.004^{c}
SN-BA-076	Z5	<LOD	<LOD	<LOD	<LOD	1.17 ± 0.11^{d}	0.87 ± 0.09^{d}	0.0189 ± 0.0017^{d}	<LOD
SN-BA-077	Z5	<LOD	0.0204 ± 0.0015^{e}	<LOD	<LOD	1.19 ± 0.11^{d}	0.82 ± 0.05^{e}	0.0095 ± 0.0007^{f}	0.017 ± 0.002^{g}
SN-BA-078	Z5	<LOD	<LOD	0.040 ± 0.004^{d}	<LOD	0.57 ± 0.04^{g}	0.45 ± 0.03^{f}	<LOD	0.017 ± 0.001^{g}
SN-BA-079	Z5	0.090 ± 0.005^{g}	0.0367 ± 0.0029^{c}	0.040 ± 0.004^{d}	0.0037 ± 0.0004^{c}	0.46 ± 0.05^{g}	0.77 ± 0.08^{e}	0.0142 ± 0.0009^{e}	0.066 ± 0.004^{c}
SN-BA-080	Z5	0.275 ± 0.015^{e}	0.0219 ± 0.0012^{e}	<LOD	<LOD	0.85 ± 0.09^{e}	0.45 ± 0.03^{f}	<LOD	0.057 ± 0.003^{d}
SN-BA-092	Z5	0.274 ± 0.030^{e}	0.0140 ± 0.0014^{f}	<LOD	0.0037 ± 0.0003^{c}	0.72 ± 0.06^{f}	1.02 ± 0.06^{d}	0.0220 ± 0.0015^{d}	<LOD
SN-BA-093	Z5	<LOD	<LOD	<LOD	<LOD	0.38 ± 0.03^{h}	0.53 ± 0.06^{f}	<LOD	0.042 ± 0.004^{e}

Notes: Values are reported as mean ± analytical SD of three independently prepared analytical portions on a dry-weight basis (mg/kg DW). The three analytical measurements were used only to assess analytical precision and were averaged to obtain one site-level concentration per matrix and georeferenced sampling site for subsequent statistical analyses. Values <LOD and <LOQ are indicated as applicable.

Table S19. Individual concentrations of macroelements in *Sambucus nigra* L. leaf samples collected from georeferenced sampling points across the investigated ecological sectors of the Oradea metropolitan area, Romania.

Sample ID	Zone	K (mg/kg)	Ca (mg/kg)	Mg (mg/kg)	Na (mg/kg)	P (mg/kg)	S (mg/kg)
SN-LE-022	Z1	21801 ± 1176^{e}	9492 ± 698^{e}	4595 ± 300^{f}	48.8 ± 2.7^{c}	3213 ± 130^{e}	1911 ± 138^{e}
SN-LE-023	Z1	21743 ± 1223^{e}	9925 ± 708^{e}	5192 ± 336^{e}	48.7 ± 3.1^{c}	3296 ± 157^{d}	1903 ± 119^{e}
SN-LE-026	Z1	21556 ± 1072^{e}	10220 ± 565^{e}	4817 ± 339^{f}	48.9 ± 2.2^{c}	3327 ± 148^{d}	2010 ± 148^{de}
SN-LE-027	Z1	22806 ± 1103^{d}	11685 ± 812^{d}	5428 ± 363^{e}	48.0 ± 2.2^{c}	3441 ± 198^{c}	2019 ± 119^{de}
SN-LE-029	Z1	21497 ± 1199^{e}	10717 ± 568^{d}	4974 ± 354^{e}	48.1 ± 2.5^{c}	3260 ± 182^{de}	1784 ± 129^{f}
SN-LE-053	Z1	23323 ± 1073^{c}	12524 ± 833^{c}	5510 ± 337^{de}	49.5 ± 3.2^{c}	3540 ± 189^{c}	2105 ± 121^{d}
SN-LE-061	Z1	22826 ± 1215^{d}	11938 ± 790^{c}	5630 ± 417^{de}	47.4 ± 2.6^{d}	3488 ± 199^{c}	2009 ± 147^{e}
SN-LE-062	Z1	21952 ± 1224^{e}	10853 ± 715^{d}	5555 ± 390^{e}	47.5 ± 2.9^{d}	3343 ± 166^{d}	1930 ± 138^{e}
SN-LE-063	Z1	22605 ± 1348^{d}	11654 ± 838^{d}	5367 ± 392^{e}	46.9 ± 2.3^{d}	3463 ± 206^{c}	1966 ± 107^{e}
SN-LE-081	Z1	22414 ± 1213^{d}	11592 ± 714^{d}	5114 ± 363^{e}	49.3 ± 2.4^{c}	3449 ± 199^{c}	1998 ± 128^{e}
SN-LE-082	Z1	23570 ± 1175^{c}	12647 ± 878^{c}	6206 ± 320^{cd}	49.2 ± 3.3^{c}	3731 ± 216^{b}	2192 ± 125^{c}
SN-LE-083	Z1	22491 ± 1411^{d}	10548 ± 538^{d}	4929 ± 281^{e}	48.6 ± 3.3^{c}	3216 ± 146^{e}	2110 ± 135^{d}
SN-LE-084	Z1	24396 ± 1334^{b}	11933 ± 708^{c}	6291 ± 339^{c}	46.3 ± 2.7^{d}	3537 ± 173^{c}	2157 ± 161^{c}

SN-LE-003	Z2	20092 ± 1266^{f}	9980 ± 731^{e}	5186 ± 272^{e}	58.4 ± 3.6^{a}	3037 ± 163^{f}	2494 ± 126^{a}
SN-LE-004	Z2	20324 ± 1191^{f}	9555 ± 675^{e}	4510 ± 253^{f}	57.3 ± 2.7^{a}	2955 ± 140^{f}	2325 ± 153^{b}
SN-LE-014	Z2	19678 ± 913^{f}	10032 ± 512^{e}	4531 ± 258^{f}	58.3 ± 3.0^{a}	2992 ± 132^{f}	2371 ± 169^{b}
SN-LE-018	Z2	20299 ± 1085^{f}	9546 ± 533^{e}	5161 ± 380^{e}	57.0 ± 3.9^{a}	3075 ± 153^{e}	2382 ± 142^{b}
SN-LE-019	Z2	20426 ± 948^{f}	8816 ± 534^{f}	5348 ± 288^{e}	54.1 ± 3.2^{ab}	3120 ± 185^{e}	2405 ± 122^{a}
SN-LE-020	Z2	19662 ± 1075^{f}	9357 ± 653^{e}	4450 ± 236^{f}	58.2 ± 2.8^{a}	3171 ± 150^{e}	2412 ± 181^{a}
SN-LE-048	Z2	20464 ± 1124^{f}	10800 ± 544^{d}	4793 ± 318^{f}	55.1 ± 3.1^{a}	3129 ± 176^{e}	2319 ± 170^{b}
SN-LE-049	Z2	21751 ± 1307^{e}	10963 ± 737^{d}	5972 ± 371^{d}	54.6 ± 2.7^{ab}	3357 ± 173^{d}	2500 ± 185^{a}
SN-LE-050	Z2	20458 ± 1244^{f}	10995 ± 712^{d}	5003 ± 294^{e}	57.4 ± 2.8^{a}	3139 ± 136^{e}	2323 ± 146^{b}
SN-LE-051	Z2	22106 ± 1429^{de}	10913 ± 675^{d}	6012 ± 337^{d}	57.2 ± 2.7^{a}	3435 ± 144^{cd}	2533 ± 140^{a}
SN-LE-052	Z2	21179 ± 1301^{e}	11252 ± 772^{d}	5307 ± 347^{e}	57.1 ± 3.7^{a}	3175 ± 165^{e}	2454 ± 176^{a}
SN-LE-054	Z2	20329 ± 930^{f}	9753 ± 728^{e}	4594 ± 334^{f}	56.3 ± 3.1^{a}	3217 ± 162^{de}	2467 ± 168^{a}
SN-LE-055	Z2	19020 ± 1068^{f}	8599 ± 478^{f}	3978 ± 242^{f}	57.7 ± 2.9^{a}	2956 ± 168^{f}	2330 ± 143^{b}
SN-LE-056	Z2	20839 ± 1287^{f}	10059 ± 693^{e}	5063 ± 271^{e}	56.9 ± 2.8^{a}	3183 ± 176^{e}	2409 ± 151^{a}
SN-LE-057	Z2	20496 ± 1269^{f}	10094 ± 636^{e}	4921 ± 347^{e}	58.1 ± 3.8^{a}	3146 ± 136^{e}	2383 ± 155^{b}
SN-LE-059	Z2	20705 ± 970^{f}	9513 ± 481^{e}	5347 ± 322^{e}	58.0 ± 2.8^{a}	3341 ± 160^{d}	2413 ± 127^{a}
SN-LE-064	Z2	21758 ± 1147^{e}	10765 ± 770^{d}	6278 ± 431^{c}	55.3 ± 3.1^{a}	3486 ± 154^{c}	2502 ± 172^{a}
SN-LE-065	Z2	20176 ± 1157^{f}	10287 ± 558^{e}	5214 ± 291^{e}	57.8 ± 3.1^{a}	2957 ± 150^{f}	2446 ± 148^{a}
SN-LE-069	Z2	21234 ± 1019^{e}	12136 ± 770^{c}	5268 ± 289^{e}	57.9 ± 2.8^{a}	3273 ± 146^{d}	2465 ± 131^{a}
SN-LE-085	Z2	20304 ± 916^{f}	9409 ± 554^{e}	4667 ± 240^{f}	56.6 ± 3.6^{a}	3112 ± 160^{e}	2369 ± 129^{b}
SN-LE-086	Z2	19976 ± 1061^{f}	7769 ± 576^{f}	4444 ± 273^{f}	56.8 ± 2.7^{a}	2958 ± 136^{f}	2301 ± 137^{b}
SN-LE-087	Z2	21204 ± 1139^{e}	10821 ± 773^{d}	5751 ± 376^{d}	56.0 ± 3.6^{a}	3378 ± 176^{d}	2358 ± 119^{b}
SN-LE-088	Z2	19048 ± 1105^{f}	7461 ± 506^{f}	3753 ± 260^{f}	56.7 ± 2.9^{a}	2974 ± 158^{f}	2276 ± 137^{b}
SN-LE-094	Z2	20391 ± 942^{f}	9349 ± 650^{e}	4949 ± 293^{e}	55.2 ± 2.5^{a}	3143 ± 154^{e}	2316 ± 123^{b}
SN-LE-001	Z3	24488 ± 1377^{b}	12767 ± 906^{c}	6556 ± 425^{c}	52.3 ± 3.2^{b}	3790 ± 171^{b}	2277 ± 170^{b}
SN-LE-005	Z3	23068 ± 1180^{d}	12712 ± 778^{c}	5790 ± 332^{d}	51.2 ± 3.1^{b}	3509 ± 157^{c}	2097 ± 148^{d}
SN-LE-006	Z3	23896 ± 1158^{c}	13061 ± 810^{b}	6296 ± 466^{c}	52.6 ± 2.6^{b}	3592 ± 146^{c}	2132 ± 111^{d}
SN-LE-007	Z3	24624 ± 1266^{b}	14555 ± 734^{ab}	7047 ± 411^{b}	50.5 ± 3.4^{b}	3791 ± 205^{b}	2324 ± 138^{b}

SN-LE-008	Z3	23938 ± 1353^{c}	11669 ± 744^{d}	6171 ± 339^{cd}	50.6 ± 3.0^{b}	3545 ± 174^{c}	2113 ± 114^{d}
SN-LE-032	Z3	24444 ± 1279^{b}	13083 ± 751^{b}	6659 ± 458^{c}	49.6 ± 2.7^{c}	3893 ± 168^{a}	2131 ± 150^{d}
SN-LE-033	Z3	24156 ± 1246^{c}	13494 ± 915^{b}	6405 ± 458^{c}	52.8 ± 2.5^{b}	3699 ± 198^{b}	2298 ± 159^{b}
SN-LE-034	Z3	24666 ± 1569^{b}	13580 ± 742^{b}	6569 ± 334^{c}	51.8 ± 2.9^{b}	3839 ± 214^{a}	2297 ± 140^{b}
SN-LE-035	Z3	24651 ± 1346^{b}	11725 ± 643^{d}	6969 ± 375^{b}	49.0 ± 3.2^{c}	3635 ± 180^{b}	2181 ± 155^{c}
SN-LE-036	Z3	24489 ± 1538^{b}	13837 ± 852^{b}	6799 ± 488^{b}	50.7 ± 3.0^{b}	3826 ± 184^{a}	2222 ± 155^{c}
SN-LE-037	Z3	24111 ± 1528^{c}	12765 ± 883^{c}	6085 ± 412^{d}	51.9 ± 3.2^{b}	3618 ± 201^{bc}	2090 ± 116^{d}
SN-LE-038	Z3	23667 ± 1117^{c}	12369 ± 758^{c}	6247 ± 315^{c}	52.0 ± 2.4^{b}	3507 ± 167^{c}	2191 ± 158^{c}
SN-LE-039	Z3	24052 ± 1334^{c}	13399 ± 729^{b}	6389 ± 448^{c}	50.4 ± 2.4^{b}	3576 ± 199^{c}	2263 ± 123^{b}
SN-LE-040	Z3	23799 ± 1290^{c}	11528 ± 616^{d}	5381 ± 355^{e}	52.4 ± 3.2^{b}	3384 ± 153^{cd}	2208 ± 142^{c}
SN-LE-041	Z3	22818 ± 1232^{d}	12642 ± 848^{c}	6136 ± 430^{cd}	53.0 ± 3.2^{b}	3454 ± 169^{c}	2179 ± 116^{c}
SN-LE-058	Z3	23405 ± 1379^{c}	12713 ± 909^{c}	5982 ± 449^{d}	51.4 ± 2.4^{b}	3562 ± 197^{c}	2161 ± 156^{c}
SN-LE-060	Z3	22936 ± 1068^{d}	13840 ± 1034^{b}	6365 ± 418^{c}	51.7 ± 2.8^{b}	3274 ± 170^{d}	2117 ± 134^{d}
SN-LE-089	Z3	22702 ± 1458^{d}	10903 ± 581^{d}	5671 ± 295^{d}	51.5 ± 3.0^{b}	3437 ± 188^{c}	2101 ± 128^{d}
SN-LE-090	Z3	23420 ± 1298^{c}	12111 ± 790^{c}	6205 ± 335^{cd}	51.3 ± 2.5^{b}	3483 ± 147^{c}	2195 ± 145^{c}
SN-LE-091	Z3	24856 ± 1119^{b}	13339 ± 810^{b}	7249 ± 504^{b}	51.6 ± 2.9^{b}	3735 ± 176^{b}	2381 ± 121^{b}
SN-LE-002	Z4	25935 ± 1169^{a}	14818 ± 1012^{a}	6970 ± 487^{b}	40.7 ± 2.1^{f}	3843 ± 168^{a}	1929 ± 125^{e}
SN-LE-009	Z4	25821 ± 1180^{a}	15460 ± 943^{a}	7075 ± 477^{b}	42.7 ± 2.2^{e}	3782 ± 168^{b}	1895 ± 128^{e}
SN-LE-010	Z4	24112 ± 1166^{bc}	13746 ± 940^{b}	5881 ± 370^{d}	44.2 ± 2.4^{e}	3307 ± 150^{d}	1924 ± 138^{e}
SN-LE-011	Z4	24298 ± 1460^{bc}	13343 ± 897^{b}	6514 ± 417^{c}	43.4 ± 2.2^{e}	3649 ± 181^{b}	1905 ± 130^{e}
SN-LE-012	Z4	26118 ± 1295^{a}	13175 ± 692^{b}	7440 ± 424^{b}	42.9 ± 2.5^{e}	3919 ± 175^{a}	1943 ± 126^{e}
SN-LE-013	Z4	27412 ± 1680^{a}	16106 ± 832^{a}	8497 ± 470^{a}	42.8 ± 2.4^{e}	4032 ± 224^{a}	2092 ± 154^{d}
SN-LE-015	Z4	24921 ± 1487^{b}	13176 ± 887^{b}	6806 ± 349^{b}	42.2 ± 2.4^{e}	3632 ± 217^{b}	1811 ± 116^{f}
SN-LE-016	Z4	26182 ± 1419^{a}	15900 ± 1048^{a}	6989 ± 368^{b}	43.0 ± 2.8^{e}	3783 ± 153^{b}	1946 ± 128^{e}
SN-LE-017	Z4	26388 ± 1516^{a}	13491 ± 983^{b}	7268 ± 502^{b}	42.5 ± 2.7^{e}	3679 ± 220^{b}	1692 ± 115^{f}
SN-LE-021	Z4	27236 ± 1478^{a}	16284 ± 1160^{a}	7925 ± 585^{a}	42.6 ± 2.3^{e}	4031 ± 197^{a}	1919 ± 111^{e}
SN-LE-024	Z4	24844 ± 1163^{b}	12500 ± 807^{c}	5976 ± 308^{d}	40.6 ± 2.1^{f}	3582 ± 152^{c}	1630 ± 82^{f}
SN-LE-025	Z4	25223 ± 1464^{b}	14033 ± 889^{b}	6398 ± 478^{c}	42.0 ± 2.9^{e}	3653 ± 178^{b}	1704 ± 117^{f}

SN-LE-028	Z4	25135 ± 1161[b]	13805 ± 953[b]	6448 ± 359[c]	41.2 ± 2.9[f]	3626 ± 149[b]	1620 ± 109[f]
SN-LE-030	Z4	26124 ± 1207[a]	13889 ± 767[b]	6493 ± 376[c]	39.7 ± 1.9[f]	3925 ± 195[a]	1706 ± 118[f]
SN-LE-031	Z4	25913 ± 1372[a]	14306 ± 752[ab]	7444 ± 424[b]	40.5 ± 2.6[f]	3861 ± 167[a]	1822 ± 129[f]
SN-LE-070	Z4	24578 ± 1382[b]	13348 ± 922[b]	6094 ± 434[d]	43.1 ± 2.3[e]	3594 ± 209[bc]	1800 ± 106[f]
SN-LE-071	Z4	25651 ± 1632[a]	14762 ± 849[a]	6923 ± 414[b]	40.4 ± 2.2[f]	3785 ± 156[b]	1812 ± 94[ef]
SN-LE-072	Z4	26486 ± 1565[a]	14945 ± 793[a]	7594 ± 461[b]	38.5 ± 2.3[f]	4015 ± 190[a]	1767 ± 89[f]
SN-LE-042	Z5	21648 ± 1108[e]	10247 ± 575[e]	4807 ± 249[f]	46.7 ± 2.8[d]	3495 ± 167[c]	2004 ± 109[de]
SN-LE-043	Z5	23750 ± 1451[c]	11957 ± 761[c]	7272 ± 370[b]	48.2 ± 2.3[c]	3876 ± 221[a]	2190 ± 131[c]
SN-LE-044	Z5	22571 ± 1126[d]	13132 ± 753[b]	6429 ± 327[c]	48.3 ± 3.1[c]	3622 ± 159[b]	2147 ± 160[c]
SN-LE-045	Z5	22466 ± 1445[d]	12006 ± 783[c]	6077 ± 413[d]	46.4 ± 2.4[d]	3401 ± 159[d]	2089 ± 142[d]
SN-LE-046	Z5	20736 ± 1275[f]	10355 ± 604[e]	4707 ± 314[f]	46.0 ± 2.8[d]	3110 ± 163[e]	1896 ± 115[e]
SN-LE-047	Z5	22139 ± 1363[e]	11471 ± 859[d]	6092 ± 360[d]	45.4 ± 2.5[de]	3503 ± 180[c]	1922 ± 139[e]
SN-LE-066	Z5	20415 ± 1091[f]	10009 ± 638[e]	4632 ± 323[f]	47.0 ± 3.0[d]	3011 ± 166[f]	1879 ± 119[ef]
SN-LE-067	Z5	22522 ± 1434[d]	10706 ± 572[d]	5396 ± 274[e]	48.5 ± 2.2[c]	3496 ± 172[c]	1969 ± 119[e]
SN-LE-068	Z5	24096 ± 1217[c]	13717 ± 824[b]	7235 ± 400[b]	45.5 ± 2.3[d]	3786 ± 202[b]	2102 ± 127[d]
SN-LE-073	Z5	23319 ± 1284[c]	12194 ± 909[c]	6364 ± 434[c]	47.3 ± 2.1[d]	3718 ± 153[b]	2126 ± 117[d]
SN-LE-074	Z5	23303 ± 1477[cd]	12744 ± 874[c]	6293 ± 373[c]	47.1 ± 3.0[d]	3497 ± 145[c]	2088 ± 131[d]
SN-LE-075	Z5	23167 ± 1177[cd]	13058 ± 862[b]	7119 ± 426[b]	47.9 ± 3.2[cd]	3650 ± 165[b]	2234 ± 148[c]
SN-LE-076	Z5	22943 ± 1285[d]	11551 ± 653[d]	6476 ± 351[c]	46.2 ± 2.3[d]	3532 ± 205[c]	2103 ± 153[d]
SN-LE-077	Z5	20685 ± 1337[f]	9702 ± 706[e]	4347 ± 270[f]	43.6 ± 2.1[de]	2959 ± 138[f]	1939 ± 128[e]
SN-LE-078	Z5	23781 ± 1155[c]	14844 ± 847[a]	7052 ± 367[b]	47.6 ± 3.1[cd]	3743 ± 177[b]	2180 ± 138[c]
SN-LE-079	Z5	21863 ± 1031[e]	8491 ± 611[f]	5029 ± 313[e]	48.4 ± 3.1[c]	3123 ± 154[e]	1934 ± 108[e]
SN-LE-080	Z5	23376 ± 1345[c]	10787 ± 745[d]	6082 ± 365[d]	47.2 ± 2.4[d]	3581 ± 196[c]	2053 ± 131[d]
SN-LE-092	Z5	22528 ± 1399[d]	11969 ± 726[c]	5435 ± 345[e]	45.9 ± 2.1[d]	3318 ± 148[d]	2037 ± 149[de]
SN-LE-093	Z5	22293 ± 1375[de]	9939 ± 568[e]	5368 ± 335[e]	45.6 ± 2.8[d]	3351 ± 190[d]	1931 ± 99[e]

Notes: Values are reported as mean ± analytical SD of three independently prepared analytical portions on a dry-weight basis (mg/kg DW). The three analytical measurements were used only to assess analytical precision and were averaged to obtain one site-level concentration per matrix and georeferenced sampling site for subsequent statistical analyses. Values <LOD and <LOQ are indicated as applicable.

Table S20. Individual concentrations of environmentally associated microelements and biologically relevant trace elements measured in georeferenced leaf samples of *Sambucus nigra* L. collected across the investigated ecological sectors of the Oradea metropolitan area, Romania.

Sample ID	Zone	^{56}Fe (mg/kg)	^{55}Mn (mg/kg)	^{64}Zn (mg/kg)	^{63}Cu (mg/kg)	^{11}B (mg/kg)	^{98}Mo (mg/kg)	^{59}Co (mg/kg)	^{80}Se (mg/kg)
SN-LE-022	Z1	148 ± 10^{d}	84.1 ± 3.2^{d}	34.2 ± 1.8^{d}	9.46 ± 0.60^{c}	32.8 ± 0.9^{c}	0.37 ± 0.04^{e}	0.35 ± 0.04^{d}	0.084 ± 0.009^{c}
SN-LE-023	Z1	155 ± 14^{c}	105.5 ± 5.0^{b}	34.1 ± 1.5^{d}	8.47 ± 0.36^{d}	34.8 ± 2.3^{b}	0.36 ± 0.07^{e}	0.27 ± 0.04^{d}	0.085 ± 0.011^{c}
SN-LE-026	Z1	115 ± 12^{f}	76.1 ± 3.3^{d}	28.6 ± 1.8^{e}	9.63 ± 0.47^{c}	34.3 ± 1.0^{b}	0.38 ± 0.05^{e}	0.38 ± 0.01^{c}	0.031 ± 0.007^{f}
SN-LE-027	Z1	140 ± 8^{de}	74.9 ± 7.4^{e}	31.4 ± 1.6^{d}	9.18 ± 0.53^{c}	30.9 ± 2.5^{d}	1.02 ± 0.07^{b}	0.19 ± 0.03^{e}	0.029 ± 0.011^{f}
SN-LE-029	Z1	164 ± 14^{c}	65.0 ± 4.9^{e}	34.8 ± 2.7^{d}	8.91 ± 0.54^{c}	31.4 ± 2.4^{c}	0.75 ± 0.06^{c}	0.37 ± 0.02^{c}	0.130 ± 0.006^{a}
SN-LE-053	Z1	142 ± 7^{d}	82.2 ± 3.3^{d}	25.4 ± 1.5^{e}	7.32 ± 0.68^{d}	31.4 ± 2.2^{c}	0.71 ± 0.06^{d}	0.45 ± 0.01^{c}	0.090 ± 0.008^{c}
SN-LE-061	Z1	164 ± 9^{c}	85.3 ± 6.8^{d}	43.5 ± 1.5^{b}	8.18 ± 0.56^{d}	38.1 ± 1.6^{a}	0.66 ± 0.10^{d}	0.26 ± 0.05^{d}	0.060 ± 0.007^{de}
SN-LE-062	Z1	140 ± 13^{de}	85.2 ± 6.2^{d}	41.8 ± 2.9^{b}	7.33 ± 0.62^{d}	30.6 ± 0.9^{d}	0.86 ± 0.05^{c}	0.25 ± 0.01^{d}	0.058 ± 0.013^{e}
SN-LE-063	Z1	170 ± 7^{bc}	66.3 ± 7.6^{e}	33.3 ± 2.9^{d}	8.97 ± 0.38^{c}	37.4 ± 1.7^{a}	0.30 ± 0.07^{f}	0.39 ± 0.02^{c}	0.067 ± 0.013^{d}
SN-LE-081	Z1	143 ± 10^{d}	91.5 ± 6.1^{c}	26.9 ± 1.6^{e}	8.11 ± 0.43^{d}	33.4 ± 1.4^{c}	0.76 ± 0.07^{c}	0.45 ± 0.02^{c}	0.059 ± 0.006^{e}
SN-LE-082	Z1	164 ± 8^{c}	95.7 ± 3.8^{c}	36.0 ± 3.3^{c}	11.19 ± 0.64^{b}	34.1 ± 1.7^{b}	0.58 ± 0.08^{d}	0.32 ± 0.04^{d}	0.116 ± 0.006^{b}
SN-LE-083	Z1	161 ± 10^{c}	94.7 ± 5.0^{c}	28.6 ± 3.1^{e}	9.24 ± 0.45^{c}	32.2 ± 1.2^{c}	0.37 ± 0.06^{e}	0.37 ± 0.03^{c}	0.091 ± 0.007^{c}
SN-LE-084	Z1	152 ± 14^{d}	119.2 ± 8.6^{b}	23.9 ± 3.1^{f}	9.44 ± 0.40^{c}	30.9 ± 1.7^{d}	0.61 ± 0.04^{d}	0.52 ± 0.03^{b}	0.082 ± 0.005^{c}
SN-LE-003	Z2	146 ± 11^{d}	123.1 ± 7.3^{b}	38.2 ± 1.6^{c}	12.28 ± 0.48^{a}	26.6 ± 2.4^{e}	0.86 ± 0.07^{c}	0.73 ± 0.03^{a}	0.056 ± 0.011^{e}
SN-LE-004	Z2	188 ± 11^{a}	100.8 ± 7.1^{c}	30.0 ± 1.8^{e}	10.14 ± 0.37^{b}	35.2 ± 2.3^{b}	1.18 ± 0.05^{b}	0.47 ± 0.03^{c}	0.076 ± 0.010^{d}
SN-LE-014	Z2	160 ± 12^{c}	103.0 ± 4.8^{c}	27.8 ± 2.0^{e}	9.79 ± 0.75^{c}	32.7 ± 1.0^{c}	1.09 ± 0.06^{b}	0.43 ± 0.05^{c}	0.105 ± 0.014^{bc}
SN-LE-018	Z2	184 ± 13^{b}	97.0 ± 7.2^{c}	37.3 ± 2.6^{c}	12.99 ± 0.89^{a}	35.5 ± 1.9^{b}	0.58 ± 0.03^{d}	0.40 ± 0.04^{c}	0.112 ± 0.007^{b}
SN-LE-019	Z2	157 ± 12^{c}	62.0 ± 4.2^{e}	40.0 ± 2.6^{c}	10.66 ± 0.46^{b}	33.0 ± 1.7^{c}	0.98 ± 0.09^{b}	0.43 ± 0.04^{c}	0.140 ± 0.005^{a}
SN-LE-020	Z2	163 ± 10^{c}	98.8 ± 3.1^{c}	47.8 ± 1.5^{a}	10.25 ± 0.83^{b}	28.3 ± 2.3^{d}	0.72 ± 0.07^{d}	0.43 ± 0.04^{c}	0.061 ± 0.010^{d}
SN-LE-048	Z2	180 ± 8^{b}	100.1 ± 3.6^{c}	36.0 ± 2.9^{c}	12.58 ± 0.83^{a}	32.7 ± 1.6^{c}	0.46 ± 0.07^{e}	0.48 ± 0.02^{c}	0.062 ± 0.013^{d}
SN-LE-049	Z2	199 ± 13^{a}	62.4 ± 8.2^{e}	43.1 ± 3.0^{b}	7.88 ± 0.46^{d}	36.4 ± 2.2^{b}	0.85 ± 0.04^{c}	0.29 ± 0.04^{d}	0.118 ± 0.013^{b}
SN-LE-050	Z2	176 ± 9^{b}	119.5 ± 8.2^{b}	34.2 ± 3.3^{d}	10.45 ± 0.47^{b}	31.1 ± 1.9^{c}	0.70 ± 0.06^{d}	0.50 ± 0.05^{b}	0.090 ± 0.009^{c}
SN-LE-051	Z2	179 ± 12^{b}	89.7 ± 4.9^{d}	41.5 ± 2.6^{b}	10.08 ± 0.61^{b}	32.2 ± 1.5^{c}	0.80 ± 0.07^{c}	0.54 ± 0.02^{b}	0.122 ± 0.011^{b}
SN-LE-052	Z2	202 ± 12^{a}	93.7 ± 5.0^{c}	31.6 ± 2.0^{d}	9.76 ± 0.81^{c}	29.9 ± 2.1^{d}	0.57 ± 0.05^{d}	0.42 ± 0.02^{c}	0.111 ± 0.008^{b}
SN-LE-054	Z2	165 ± 9^{c}	101.0 ± 6.3^{c}	43.4 ± 1.3^{b}	11.10 ± 0.47^{b}	35.5 ± 1.6^{b}	0.72 ± 0.07^{d}	0.55 ± 0.04^{b}	0.066 ± 0.009^{d}

SN-LE-055	Z2	163 ± 12[c]	96.7 ± 5.0[c]	28.4 ± 3.0[e]	10.61 ± 0.75[b]	31.8 ± 2.0[c]	0.70 ± 0.05[d]	0.53 ± 0.02[b]	0.084 ± 0.004[c]
SN-LE-056	Z2	175 ± 10[b]	109.3 ± 4.5[b]	39.4 ± 2.7[c]	9.81 ± 0.30[c]	26.4 ± 2.1[e]	0.94 ± 0.08[c]	0.47 ± 0.01[c]	0.108 ± 0.008[b]
SN-LE-057	Z2	191 ± 12[a]	126.3 ± 8.9[a]	37.0 ± 2.6[c]	10.16 ± 0.87[b]	29.7 ± 2.3[d]	1.18 ± 0.07[b]	0.25 ± 0.04[d]	0.105 ± 0.009[bc]
SN-LE-059	Z2	181 ± 13[b]	77.0 ± 6.1[d]	46.1 ± 2.6[a]	9.03 ± 0.46[c]	27.8 ± 1.0[e]	0.86 ± 0.05[c]	0.45 ± 0.02[c]	0.142 ± 0.007[a]
SN-LE-064	Z2	165 ± 10[c]	91.7 ± 4.2[c]	40.6 ± 2.2[b]	12.05 ± 0.84[a]	30.9 ± 1.8[d]	0.77 ± 0.08[c]	0.33 ± 0.04[d]	0.117 ± 0.012[b]
SN-LE-065	Z2	175 ± 11[b]	94.0 ± 6.6[c]	33.9 ± 1.8[d]	11.80 ± 0.43[a]	35.9 ± 1.6[b]	0.46 ± 0.03[e]	0.26 ± 0.02[d]	0.099 ± 0.011[c]
SN-LE-069	Z2	169 ± 7[c]	97.3 ± 6.9[c]	35.6 ± 1.6[c]	10.31 ± 0.58[b]	29.6 ± 1.0[d]	0.77 ± 0.06[c]	0.30 ± 0.02[d]	0.087 ± 0.007[c]
SN-LE-085	Z2	173 ± 14[b]	78.1 ± 5.1[d]	38.8 ± 2.7[c]	7.92 ± 0.77[d]	29.7 ± 1.9[d]	0.88 ± 0.08[c]	0.56 ± 0.05[b]	0.082 ± 0.006[c]
SN-LE-086	Z2	180 ± 8[b]	94.7 ± 6.6[c]	44.9 ± 2.9[b]	11.48 ± 0.37[b]	28.4 ± 2.2[d]	0.78 ± 0.10[c]	0.38 ± 0.01[c]	0.060 ± 0.004[de]
SN-LE-087	Z2	169 ± 7[c]	114.5 ± 7.6[b]	44.5 ± 3.1[b]	9.91 ± 0.66[c]	31.0 ± 1.1[d]	0.77 ± 0.10[c]	0.39 ± 0.04[c]	0.076 ± 0.007[d]
SN-LE-088	Z2	163 ± 12[c]	99.0 ± 8.0[c]	34.1 ± 2.8[d]	10.17 ± 0.56[b]	34.5 ± 2.1[b]	0.56 ± 0.10[d]	0.66 ± 0.02[a]	0.132 ± 0.012[a]
SN-LE-094	Z2	157 ± 14[c]	88.1 ± 4.5[d]	30.8 ± 2.3[d]	10.95 ± 0.67[b]	29.3 ± 1.5[d]	1.16 ± 0.03[b]	0.44 ± 0.04[c]	0.065 ± 0.006[d]
SN-LE-001	Z3	164 ± 7[c]	104.9 ± 7.3[c]	29.3 ± 2.3[e]	10.77 ± 0.75[b]	27.0 ± 2.0[e]	0.44 ± 0.08[e]	0.59 ± 0.03[b]	0.051 ± 0.012[e]
SN-LE-005	Z3	168 ± 12[c]	85.6 ± 5.0[d]	34.2 ± 2.8[d]	7.74 ± 0.46[d]	25.4 ± 1.3[e]	0.50 ± 0.04[e]	0.22 ± 0.02[e]	0.057 ± 0.005[e]
SN-LE-006	Z3	136 ± 9[e]	73.8 ± 3.6[e]	36.9 ± 1.7[c]	9.49 ± 0.44[c]	25.0 ± 1.2[f]	0.64 ± 0.03[d]	0.49 ± 0.03[b]	0.078 ± 0.007[d]
SN-LE-007	Z3	172 ± 10[b]	75.5 ± 5.6[d]	33.7 ± 1.7[d]	8.13 ± 0.33[d]	31.3 ± 2.4[c]	0.46 ± 0.06[e]	0.46 ± 0.04[c]	0.062 ± 0.008[d]
SN-LE-008	Z3	176 ± 13[b]	87.7 ± 6.6[d]	36.9 ± 3.0[c]	8.93 ± 0.44[c]	25.4 ± 1.7[e]	0.54 ± 0.06[e]	0.40 ± 0.03[c]	0.066 ± 0.013[d]
SN-LE-032	Z3	171 ± 12[b]	78.2 ± 3.9[d]	30.1 ± 1.7[d]	7.55 ± 0.35[d]	27.4 ± 0.9[e]	0.24 ± 0.05[f]	0.34 ± 0.03[d]	0.064 ± 0.007[d]
SN-LE-033	Z3	159 ± 12[c]	103.1 ± 8.6[c]	34.2 ± 2.2[d]	7.42 ± 0.63[d]	26.0 ± 1.9[e]	0.21 ± 0.09[f]	0.36 ± 0.04[d]	0.020 ± 0.005[f]
SN-LE-034	Z3	162 ± 14[c]	90.5 ± 6.0[c]	35.3 ± 2.3[c]	7.15 ± 0.35[d]	28.9 ± 1.0[d]	0.69 ± 0.06[d]	0.28 ± 0.02[d]	0.034 ± 0.007[f]
SN-LE-035	Z3	188 ± 7[a]	99.2 ± 7.5[c]	37.8 ± 2.3[c]	7.37 ± 0.69[d]	39.7 ± 2.4[a]	0.40 ± 0.04[e]	0.32 ± 0.03[d]	0.068 ± 0.010[d]
SN-LE-036	Z3	153 ± 9[d]	78.7 ± 8.7[d]	31.7 ± 3.0[d]	9.60 ± 0.63[c]	24.7 ± 1.6[f]	0.30 ± 0.05[f]	0.27 ± 0.02[d]	0.113 ± 0.014[b]
SN-LE-037	Z3	143 ± 13[d]	89.4 ± 6.8[d]	42.5 ± 2.9[b]	8.56 ± 0.42[c]	24.8 ± 1.8[f]	0.55 ± 0.09[de]	0.37 ± 0.03[c]	0.081 ± 0.011[c]
SN-LE-038	Z3	183 ± 14[b]	107.7 ± 8.5[b]	32.9 ± 2.3[d]	8.12 ± 0.37[d]	31.1 ± 2.2[c]	0.77 ± 0.06[c]	0.27 ± 0.04[d]	0.043 ± 0.009[e]
SN-LE-039	Z3	157 ± 14[c]	107.4 ± 8.2[b]	38.9 ± 1.9[c]	8.58 ± 0.77[c]	26.3 ± 1.6[e]	0.53 ± 0.10[e]	0.44 ± 0.03[c]	0.040 ± 0.012[e]
SN-LE-040	Z3	155 ± 11[c]	90.7 ± 6.3[c]	22.1 ± 2.3[f]	6.41 ± 0.49[e]	26.7 ± 1.2[e]	0.62 ± 0.08[d]	0.25 ± 0.03[d]	0.069 ± 0.006[d]
SN-LE-041	Z3	156 ± 14[c]	74.0 ± 6.0[e]	34.2 ± 1.4[d]	6.85 ± 0.60[e]	28.9 ± 1.8[d]	0.46 ± 0.09[e]	0.25 ± 0.04[d]	0.048 ± 0.008[e]
SN-LE-058	Z3	145 ± 9[d]	76.7 ± 7.2[d]	34.5 ± 2.7[d]	7.50 ± 0.81[d]	27.1 ± 2.3[e]	0.52 ± 0.09[e]	0.08 ± 0.03[f]	0.087 ± 0.007[c]

SN-LE-060	Z3	156 ± 12^{c}	54.9 ± 5.6^{f}	36.7 ± 1.5^{c}	8.46 ± 0.68^{d}	33.2 ± 2.3^{c}	0.69 ± 0.06^{d}	0.66 ± 0.04^{a}	0.020 ± 0.013^{f}
SN-LE-089	Z3	179 ± 8^{b}	86.7 ± 3.5^{d}	33.7 ± 3.1^{d}	9.80 ± 0.67^{c}	31.6 ± 1.3^{c}	0.76 ± 0.09^{c}	0.14 ± 0.02^{e}	0.029 ± 0.009^{f}
SN-LE-090	Z3	156 ± 9^{c}	89.8 ± 6.5^{d}	43.3 ± 2.6^{b}	8.82 ± 0.55^{c}	32.2 ± 1.3^{c}	0.44 ± 0.09^{e}	0.40 ± 0.02^{c}	0.081 ± 0.008^{c}
SN-LE-091	Z3	150 ± 9^{d}	79.7 ± 6.4^{d}	28.7 ± 2.7^{e}	8.03 ± 0.75^{d}	30.4 ± 1.0^{d}	0.61 ± 0.07^{d}	0.51 ± 0.03^{b}	0.075 ± 0.013^{d}
SN-LE-002	Z4	134 ± 12^{e}	127.7 ± 8.0^{a}	28.4 ± 2.6^{e}	7.12 ± 0.84^{d}	31.9 ± 1.9^{c}	0.39 ± 0.05^{e}	0.18 ± 0.03^{e}	0.043 ± 0.010^{e}
SN-LE-009	Z4	135 ± 11^{e}	95.2 ± 8.5^{c}	29.9 ± 3.0^{e}	9.71 ± 0.45^{c}	36.4 ± 2.2^{b}	0.65 ± 0.06^{d}	0.09 ± 0.03^{f}	0.046 ± 0.011^{e}
SN-LE-010	Z4	134 ± 14^{e}	107.2 ± 7.8^{b}	34.1 ± 2.1^{d}	7.63 ± 0.77^{d}	39.9 ± 0.9^{a}	0.47 ± 0.07^{e}	0.17 ± 0.03^{e}	0.020 ± 0.011^{f}
SN-LE-011	Z4	132 ± 11^{e}	82.6 ± 8.5^{d}	35.9 ± 1.8^{c}	6.18 ± 0.34^{e}	36.5 ± 2.2^{b}	0.62 ± 0.09^{d}	0.45 ± 0.02^{c}	0.048 ± 0.008^{e}
SN-LE-012	Z4	158 ± 12^{c}	128.6 ± 7.5^{a}	29.3 ± 3.3^{e}	9.12 ± 0.55^{c}	39.5 ± 2.1^{a}	0.78 ± 0.04^{c}	0.32 ± 0.03^{d}	0.078 ± 0.005^{d}
SN-LE-013	Z4	122 ± 7^{f}	102.7 ± 7.1^{c}	33.4 ± 2.4^{d}	7.72 ± 0.89^{d}	32.6 ± 1.5^{c}	0.72 ± 0.06^{d}	0.46 ± 0.02^{c}	0.061 ± 0.005^{d}
SN-LE-015	Z4	147 ± 8^{d}	88.2 ± 6.4^{d}	28.4 ± 1.4^{e}	6.35 ± 0.76^{e}	35.2 ± 1.2^{b}	0.55 ± 0.05^{de}	0.28 ± 0.04^{d}	0.057 ± 0.007^{e}
SN-LE-016	Z4	157 ± 12^{c}	126.5 ± 3.2^{a}	41.1 ± 1.3^{b}	7.84 ± 0.77^{d}	36.5 ± 1.3^{b}	0.63 ± 0.05^{d}	0.24 ± 0.02^{e}	0.070 ± 0.005^{d}
SN-LE-017	Z4	136 ± 14^{e}	81.9 ± 3.3^{d}	30.5 ± 2.9^{d}	6.42 ± 0.82^{e}	35.2 ± 1.7^{b}	0.33 ± 0.06^{f}	0.32 ± 0.05^{d}	0.057 ± 0.005^{e}
SN-LE-021	Z4	123 ± 7^{f}	102.9 ± 4.7^{c}	29.1 ± 2.6^{e}	6.50 ± 0.63^{e}	35.3 ± 1.4^{b}	0.51 ± 0.10^{e}	0.36 ± 0.03^{d}	0.060 ± 0.009^{de}
SN-LE-024	Z4	135 ± 10^{e}	97.5 ± 4.4^{c}	29.3 ± 2.0^{e}	6.05 ± 0.57^{e}	31.9 ± 1.6^{c}	0.32 ± 0.04^{f}	0.23 ± 0.03^{e}	0.048 ± 0.011^{e}
SN-LE-025	Z4	134 ± 13^{e}	107.7 ± 6.3^{b}	33.7 ± 2.5^{d}	7.61 ± 0.64^{d}	36.5 ± 1.4^{b}	0.51 ± 0.04^{e}	0.41 ± 0.02^{c}	0.100 ± 0.011^{c}
SN-LE-028	Z4	136 ± 12^{e}	70.4 ± 4.3^{e}	28.7 ± 1.5^{e}	6.05 ± 0.81^{e}	32.9 ± 1.5^{c}	0.47 ± 0.08^{e}	0.18 ± 0.03^{e}	0.062 ± 0.012^{d}
SN-LE-030	Z4	157 ± 9^{c}	66.6 ± 4.1^{e}	33.6 ± 2.8^{d}	6.29 ± 0.49^{e}	33.9 ± 1.4^{c}	0.52 ± 0.03^{e}	0.42 ± 0.04^{c}	0.030 ± 0.010^{f}
SN-LE-031	Z4	134 ± 9^{e}	105.9 ± 7.3^{b}	30.7 ± 2.9^{d}	7.56 ± 0.64^{d}	34.3 ± 1.0^{b}	0.41 ± 0.03^{e}	0.13 ± 0.02^{e}	0.049 ± 0.010^{e}
SN-LE-070	Z4	134 ± 10^{e}	91.2 ± 4.7^{c}	29.3 ± 2.7^{e}	6.61 ± 0.59^{e}	32.9 ± 2.0^{c}	0.60 ± 0.03^{d}	0.23 ± 0.03^{e}	0.037 ± 0.010^{f}
SN-LE-071	Z4	147 ± 8^{d}	102.0 ± 5.3^{c}	32.5 ± 2.8^{d}	8.44 ± 0.60^{d}	35.7 ± 2.2^{b}	0.73 ± 0.09^{d}	0.43 ± 0.01^{c}	0.072 ± 0.013^{d}
SN-LE-072	Z4	141 ± 7^{d}	101.2 ± 3.1^{c}	39.7 ± 2.0^{c}	6.68 ± 0.78^{e}	31.4 ± 1.9^{c}	0.47 ± 0.09^{e}	0.23 ± 0.05^{e}	0.054 ± 0.005^{e}
SN-LE-042	Z5	172 ± 8^{b}	78.0 ± 6.1^{d}	32.1 ± 1.5^{d}	8.73 ± 0.61^{c}	29.1 ± 2.3^{d}	0.82 ± 0.05^{c}	0.31 ± 0.04^{d}	0.105 ± 0.012^{bc}
SN-LE-043	Z5	167 ± 13^{c}	78.1 ± 6.0^{d}	40.2 ± 2.9^{b}	10.12 ± 0.76^{b}	27.1 ± 1.6^{e}	0.52 ± 0.09^{e}	0.31 ± 0.03^{d}	0.054 ± 0.010^{e}
SN-LE-044	Z5	144 ± 7^{d}	67.7 ± 6.0^{e}	36.8 ± 2.0^{c}	8.99 ± 0.57^{c}	35.6 ± 1.0^{b}	0.78 ± 0.09^{c}	0.31 ± 0.01^{d}	0.079 ± 0.013^{d}
SN-LE-045	Z5	176 ± 13^{b}	71.9 ± 6.9^{e}	32.6 ± 2.0^{d}	9.44 ± 0.59^{c}	27.4 ± 1.1^{e}	0.61 ± 0.03^{d}	0.45 ± 0.01^{c}	0.061 ± 0.013^{d}
SN-LE-046	Z5	154 ± 8^{d}	107.1 ± 5.2^{b}	34.2 ± 1.9^{d}	9.49 ± 0.55^{c}	32.0 ± 1.4^{c}	0.93 ± 0.07^{c}	0.25 ± 0.04^{d}	0.107 ± 0.010^{b}
SN-LE-047	Z5	167 ± 9^{c}	90.1 ± 6.5^{c}	35.6 ± 1.6^{c}	8.63 ± 0.51^{c}	30.5 ± 2.4^{d}	0.48 ± 0.08^{e}	0.56 ± 0.03^{b}	0.071 ± 0.010^{d}

SN-LE-066	Z5	173 ± 12^{b}	83.5 ± 6.2^{d}	32.0 ± 2.7^{d}	10.99 ± 0.55^{b}	33.4 ± 1.5^{c}	0.47 ± 0.06^{e}	0.45 ± 0.02^{c}	0.046 ± 0.006^{e}
SN-LE-067	Z5	180 ± 13^{b}	83.2 ± 8.3^{d}	39.2 ± 1.4^{c}	10.13 ± 0.50^{b}	38.0 ± 2.1^{a}	0.86 ± 0.04^{c}	0.49 ± 0.03^{b}	0.087 ± 0.006^{c}
SN-LE-068	Z5	176 ± 10^{b}	65.6 ± 4.7^{e}	39.1 ± 2.9^{c}	8.81 ± 0.57^{c}	29.7 ± 1.8^{d}	0.77 ± 0.05^{c}	0.31 ± 0.04^{d}	0.048 ± 0.009^{e}
SN-LE-073	Z5	168 ± 14^{c}	74.9 ± 7.9^{e}	50.9 ± 3.0^{a}	8.37 ± 0.38^{d}	29.3 ± 1.3^{d}	0.76 ± 0.07^{c}	0.26 ± 0.04^{d}	0.103 ± 0.010^{c}
SN-LE-074	Z5	184 ± 10^{b}	105.0 ± 8.7^{c}	40.0 ± 1.8^{c}	8.73 ± 0.57^{c}	31.4 ± 1.5^{c}	0.60 ± 0.03^{d}	0.43 ± 0.01^{c}	0.091 ± 0.009^{c}
SN-LE-075	Z5	170 ± 8^{bc}	136.7 ± 4.7^{a}	37.7 ± 3.0^{c}	9.72 ± 0.72^{c}	37.3 ± 1.1^{a}	0.59 ± 0.08^{d}	0.26 ± 0.03^{d}	0.060 ± 0.004^{de}
SN-LE-076	Z5	172 ± 9^{b}	94.2 ± 3.3^{c}	41.9 ± 2.2^{b}	8.19 ± 0.78^{d}	32.1 ± 1.6^{c}	0.50 ± 0.04^{e}	0.42 ± 0.05^{c}	0.087 ± 0.010^{c}
SN-LE-077	Z5	177 ± 12^{b}	89.7 ± 3.5^{d}	38.0 ± 3.3^{c}	10.15 ± 0.34^{b}	28.6 ± 1.7^{d}	0.81 ± 0.07^{c}	0.35 ± 0.05^{d}	0.085 ± 0.010^{c}
SN-LE-078	Z5	154 ± 12^{d}	97.9 ± 6.8^{c}	27.9 ± 2.9^{e}	6.94 ± 0.74^{e}	31.6 ± 1.4^{c}	0.74 ± 0.03^{d}	0.37 ± 0.03^{c}	0.047 ± 0.012^{e}
SN-LE-079	Z5	176 ± 10^{b}	110.9 ± 4.8^{b}	30.2 ± 1.4^{d}	9.92 ± 0.42^{c}	27.3 ± 1.0^{e}	0.49 ± 0.10^{e}	0.54 ± 0.03^{b}	0.105 ± 0.014^{bc}
SN-LE-080	Z5	153 ± 7^{d}	85.6 ± 5.2^{d}	38.7 ± 3.2^{c}	8.74 ± 0.75^{c}	28.5 ± 2.3^{d}	0.99 ± 0.06^{b}	0.45 ± 0.05^{c}	0.084 ± 0.009^{c}
SN-LE-092	Z5	133 ± 11^{e}	89.2 ± 4.9^{d}	29.7 ± 1.5^{e}	9.25 ± 0.70^{c}	32.6 ± 1.4^{c}	0.66 ± 0.07^{d}	0.21 ± 0.03^{e}	0.062 ± 0.011^{d}
SN-LE-093	Z5	164 ± 7^{c}	91.2 ± 6.8^{c}	40.9 ± 2.1^{b}	8.67 ± 0.78^{c}	35.0 ± 2.0^{b}	0.71 ± 0.10^{d}	0.61 ± 0.02^{a}	0.061 ± 0.010^{d}

Notes: Values are reported as mean ± analytical SD of three independently prepared analytical portions on a dry-weight basis (mg/kg DW). The three analytical measurements were used only to assess analytical precision and were averaged to obtain one site-level concentration per matrix and georeferenced sampling site for subsequent statistical analyses. Values <LOD and <LOQ are indicated as applicable.

Table S21. Individual concentrations of lithogenic and geochemically associated elements in *Sambucus nigra* L. leaf samples collected at georeferenced sampling points across the ecological sectors of the Oradea metropolitan area, Romania.

Sample ID	Zone	^{27}Al (mg/kg)	^{48}Ti (mg/kg)	^{51}V (mg/kg)	^{137}Ba (mg/kg)	^{88}Sr (mg/kg)	^{7}Li (mg/kg)	^{85}Rb (mg/kg)	^{133}Cs (mg/kg)
SN-LE-022	Z1	43.5 ± 2.5^{gh}	2.81 ± 0.19^{h}	0.86 ± 0.06^{hi}	20.4 ± 1.7^{gh}	12.5 ± 0.8^{fg}	0.54 ± 0.04^{f}	2.08 ± 0.14^{g}	0.064 ± 0.005^{g}
SN-LE-023	Z1	47.9 ± 3.6^{a}	3.14 ± 0.18^{h}	0.78 ± 0.06^{i}	23.8 ± 1.4^{cd}	15.3 ± 1.1^{d}	0.79 ± 0.05^{c}	2.54 ± 0.19^{ef}	0.081 ± 0.007^{c}
SN-LE-026	Z1	39.5 ± 3.7^{h}	2.55 ± 0.17^{i}	0.64 ± 0.06^{ij}	19.7 ± 1.0^{h}	12.2 ± 0.8^{g}	0.57 ± 0.04^{f}	1.94 ± 0.13^{g}	0.048 ± 0.003^{h}
SN-LE-027	Z1	40.4 ± 3.6^{d}	2.24 ± 0.20^{i}	0.77 ± 0.06^{i}	16.8 ± 1.3^{h}	11.4 ± 0.6^{g}	0.52 ± 0.05^{f}	1.55 ± 0.11^{h}	0.047 ± 0.003^{h}
SN-LE-029	Z1	45.9 ± 3.5^{g}	2.74 ± 0.25^{h}	1.04 ± 0.08^{c}	24.3 ± 1.9^{g}	13.2 ± 0.7^{f}	0.56 ± 0.05^{f}	2.34 ± 0.16^{f}	0.067 ± 0.006^{ef}
SN-LE-053	Z1	33.2 ± 2.9^{hi}	1.88 ± 0.13^{j}	0.75 ± 0.06^{i}	21.7 ± 1.3^{g}	13.8 ± 0.9^{f}	0.61 ± 0.05^{f}	2.00 ± 0.11^{g}	0.061 ± 0.005^{gh}
SN-LE-061	Z1	17.7 ± 1.4^{j}	1.46 ± 0.10^{j}	0.50 ± 0.03^{j}	16.3 ± 1.3^{h}	12.0 ± 0.9^{g}	0.51 ± 0.05^{f}	1.44 ± 0.11^{h}	0.046 ± 0.003^{h}
SN-LE-062	Z1	18.3 ± 1.4^{j}	1.38 ± 0.11^{jk}	0.51 ± 0.04^{j}	18.1 ± 1.3^{h}	12.5 ± 1.0^{i}	0.49 ± 0.03^{fg}	1.61 ± 0.11^{h}	0.047 ± 0.003^{h}

SN-LE-063	Z1	21.1 ± 1.3^{j}	1.85 ± 0.16^{i}	0.85 ± 0.06^{f}	17.8 ± 1.1^{i}	11.5 ± 0.7^{ij}	0.63 ± 0.06^{fg}	1.85 ± 0.16^{ij}	0.067 ± 0.006^{ef}
SN-LE-081	Z1	31.0 ± 2.2^{h}	2.40 ± 0.17^{f}	0.62 ± 0.05^{ij}	18.3 ± 1.5^{i}	14.4 ± 1.0^{ef}	0.70 ± 0.05^{de}	1.73 ± 0.13^{j}	0.061 ± 0.004^{h}
SN-LE-082	Z1	40.0 ± 3.1^{d}	2.43 ± 0.21^{f}	0.59 ± 0.03^{j}	20.1 ± 1.2^{gh}	12.5 ± 1.0^{i}	0.60 ± 0.04^{gh}	2.26 ± 0.15^{ef}	0.066 ± 0.004^{f}
SN-LE-083	Z1	46.2 ± 3.6^{ab}	1.85 ± 0.16^{ij}	0.86 ± 0.08^{f}	18.6 ± 1.1^{i}	13.8 ± 1.2^{fg}	0.66 ± 0.05^{f}	2.07 ± 0.15^{gh}	0.055 ± 0.004^{i}
SN-LE-084	Z1	26.4 ± 1.7^{ij}	2.54 ± 0.16^{e}	1.01 ± 0.07^{d}	19.4 ± 1.1^{h}	13.1 ± 0.8^{h}	0.55 ± 0.03^{i}	2.38 ± 0.21^{e}	0.051 ± 0.003^{ij}
SN-LE-003	Z2	47.5 ± 3.2^{a}	3.48 ± 0.21^{a}	1.02 ± 0.06^{cd}	30.1 ± 2.2^{a}	17.2 ± 1.4^{ab}	0.69 ± 0.04^{e}	2.99 ± 0.18^{ab}	0.077 ± 0.004^{d}
SN-LE-004	Z2	46.5 ± 3.3^{ab}	3.05 ± 0.23^{b}	1.09 ± 0.08^{b}	29.0 ± 2.5^{a}	14.4 ± 1.0^{f}	0.82 ± 0.07^{b}	3.02 ± 0.25^{a}	0.098 ± 0.007^{a}
SN-LE-014	Z2	43.0 ± 3.3^{c}	2.30 ± 0.19^{g}	0.86 ± 0.07^{f}	22.7 ± 1.6^{e}	17.7 ± 1.2^{a}	0.72 ± 0.04^{d}	3.24 ± 0.20^{a}	0.079 ± 0.007^{cd}
SN-LE-018	Z2	38.6 ± 2.9^{e}	2.64 ± 0.20^{d}	0.93 ± 0.06^{e}	25.8 ± 2.1^{b}	16.6 ± 1.2^{b}	0.87 ± 0.07^{ab}	2.90 ± 0.23^{b}	0.069 ± 0.006^{ef}
SN-LE-019	Z2	41.6 ± 3.3^{d}	2.50 ± 0.21^{ef}	1.20 ± 0.09^{a}	23.3 ± 1.4^{de}	14.1 ± 1.2^{fg}	0.75 ± 0.05^{cd}	2.74 ± 0.21^{c}	0.100 ± 0.008^{a}
SN-LE-020	Z2	44.6 ± 3.3^{bc}	3.48 ± 0.27^{a}	0.79 ± 0.06^{gh}	28.7 ± 2.3^{a}	14.6 ± 0.9^{e}	0.77 ± 0.05^{cd}	2.98 ± 0.24^{ab}	0.067 ± 0.005^{f}
SN-LE-048	Z2	39.5 ± 2.9^{de}	2.95 ± 0.20^{bc}	1.03 ± 0.08^{c}	25.8 ± 2.3^{b}	14.2 ± 1.0^{f}	0.82 ± 0.06^{bc}	2.26 ± 0.13^{ef}	0.079 ± 0.005^{cd}
SN-LE-049	Z2	45.8 ± 3.2^{b}	3.51 ± 0.20^{a}	1.18 ± 0.07^{a}	24.1 ± 1.6^{c}	14.8 ± 1.3^{de}	0.89 ± 0.07^{a}	2.74 ± 0.24^{cd}	0.081 ± 0.007^{c}
SN-LE-050	Z2	50.5 ± 3.2^{a}	2.64 ± 0.15^{de}	0.97 ± 0.08^{de}	28.5 ± 2.3^{a}	17.1 ± 1.2^{ab}	0.63 ± 0.04^{fg}	3.26 ± 0.20^{a}	0.101 ± 0.006^{a}
SN-LE-051	Z2	43.9 ± 2.8^{bc}	2.39 ± 0.15^{fg}	1.05 ± 0.08^{bc}	20.8 ± 1.7^{f}	18.2 ± 1.3^{a}	0.78 ± 0.05^{c}	2.82 ± 0.25^{c}	0.078 ± 0.006^{cd}
SN-LE-052	Z2	43.1 ± 3.7^{c}	3.64 ± 0.26^{a}	0.79 ± 0.05^{gh}	22.2 ± 1.5^{ef}	15.6 ± 0.9^{cd}	0.67 ± 0.05^{f}	2.39 ± 0.19^{de}	0.101 ± 0.006^{a}
SN-LE-054	Z2	42.5 ± 2.8^{cd}	2.82 ± 0.16^{c}	0.88 ± 0.07^{f}	23.5 ± 1.4^{d}	14.2 ± 0.9^{f}	0.91 ± 0.06^{a}	2.89 ± 0.19^{b}	0.095 ± 0.007^{a}
SN-LE-055	Z2	46.0 ± 3.1^{b}	2.65 ± 0.17^{d}	1.25 ± 0.11^{a}	22.9 ± 1.9^{e}	14.8 ± 0.9^{de}	0.84 ± 0.05^{b}	3.09 ± 0.19^{a}	0.067 ± 0.004^{f}
SN-LE-056	Z2	36.6 ± 2.4^{f}	2.89 ± 0.19^{c}	1.13 ± 0.10^{b}	23.7 ± 2.1^{d}	16.1 ± 0.9^{bc}	0.68 ± 0.05^{e}	2.85 ± 0.22^{bc}	0.065 ± 0.005^{fg}
SN-LE-057	Z2	42.5 ± 2.8^{c}	3.57 ± 0.21^{a}	1.03 ± 0.06^{c}	23.8 ± 1.4^{cd}	13.1 ± 0.8^{h}	0.90 ± 0.08^{a}	3.30 ± 0.28^{a}	0.080 ± 0.005^{c}
SN-LE-059	Z2	43.0 ± 3.7^{c}	2.62 ± 0.17^{de}	1.04 ± 0.07^{c}	27.8 ± 1.8^{ab}	15.5 ± 1.3^{cd}	0.83 ± 0.06^{b}	2.76 ± 0.17^{c}	0.083 ± 0.006^{bc}
SN-LE-064	Z2	39.2 ± 2.3^{e}	2.34 ± 0.20^{fg}	1.02 ± 0.07^{cd}	23.5 ± 2.1^{d}	15.5 ± 1.1^{d}	0.89 ± 0.06^{a}	2.22 ± 0.15^{f}	0.076 ± 0.007^{de}
SN-LE-065	Z2	40.6 ± 3.4^{d}	3.36 ± 0.24^{ab}	1.13 ± 0.08^{b}	26.1 ± 2.2^{b}	14.9 ± 0.9^{de}	0.91 ± 0.08^{a}	2.57 ± 0.16^{d}	0.087 ± 0.005^{bc}
SN-LE-069	Z2	47.0 ± 3.6^{ab}	2.90 ± 0.17^{c}	1.24 ± 0.08^{a}	24.9 ± 2.2^{bc}	15.3 ± 1.0^{d}	0.84 ± 0.06^{ab}	2.79 ± 0.23^{c}	0.093 ± 0.008^{ab}
SN-LE-085	Z2	34.0 ± 2.9^{g}	3.26 ± 0.27^{ab}	1.04 ± 0.06^{bc}	23.5 ± 1.4^{d}	18.5 ± 1.3^{a}	0.86 ± 0.05^{ab}	2.58 ± 0.22^{d}	0.078 ± 0.005^{cd}
SN-LE-086	Z2	47.9 ± 3.1^{a}	2.43 ± 0.20^{f}	0.91 ± 0.06^{ef}	24.3 ± 1.8^{c}	15.5 ± 1.2^{cd}	0.85 ± 0.06^{ab}	2.65 ± 0.24^{cd}	0.093 ± 0.006^{ab}
SN-LE-087	Z2	40.4 ± 2.3^{d}	3.19 ± 0.20^{b}	0.92 ± 0.07^{ef}	28.2 ± 1.6^{ab}	17.9 ± 1.6^{a}	0.82 ± 0.06^{bc}	2.92 ± 0.25^{b}	0.090 ± 0.007^{b}
SN-LE-088	Z2	33.0 ± 2.8^{g}	3.44 ± 0.21^{ab}	1.22 ± 0.10^{a}	29.4 ± 2.1^{a}	16.2 ± 1.3^{b}	0.83 ± 0.07^{b}	2.68 ± 0.21^{cd}	0.090 ± 0.007^{b}

SN-LE-094	Z2	41.9 ± 2.8cd	2.80 ± 0.19cd	0.80 ± 0.05g	21.3 ± 1.7f	15.8 ± 1.4bc	0.80 ± 0.05bc	2.63 ± 0.20cd	0.077 ± 0.006d
SN-LE-001	Z3	51.7 ± 3.2a	2.67 ± 0.18d	0.75 ± 0.06hi	20.1 ± 1.8g	14.7 ± 0.9e	0.72 ± 0.05d	2.33 ± 0.13e	0.062 ± 0.005g
SN-LE-005	Z3	44.7 ± 3.6b	3.17 ± 0.22b	1.11 ± 0.09b	22.3 ± 1.4e	13.6 ± 0.9g	0.78 ± 0.05c	2.50 ± 0.20d	0.075 ± 0.006de
SN-LE-006	Z3	43.9 ± 3.5bc	3.03 ± 0.19bc	1.01 ± 0.07d	21.2 ± 1.2f	15.8 ± 1.3bc	0.70 ± 0.06de	2.89 ± 0.17b	0.064 ± 0.005g
SN-LE-007	Z3	31.2 ± 1.7h	2.20 ± 0.18gh	0.85 ± 0.05fg	20.6 ± 1.7fg	15.3 ± 1.3d	0.58 ± 0.05hi	2.07 ± 0.12gh	0.085 ± 0.005bc
SN-LE-008	Z3	36.3 ± 3.2f	2.27 ± 0.17g	1.02 ± 0.07cd	23.9 ± 2.0cd	15.7 ± 1.4c	0.79 ± 0.05c	2.09 ± 0.16g	0.093 ± 0.008ab
SN-LE-032	Z3	39.7 ± 2.9de	2.57 ± 0.15e	1.05 ± 0.07bc	26.8 ± 2.0b	15.7 ± 0.9c	0.71 ± 0.05de	2.86 ± 0.22bc	0.070 ± 0.005ef
SN-LE-033	Z3	39.2 ± 3.4e	2.79 ± 0.23cd	0.91 ± 0.06ef	28.5 ± 2.4ab	13.3 ± 0.9gh	0.70 ± 0.06e	2.14 ± 0.16fg	0.084 ± 0.005bc
SN-LE-034	Z3	46.5 ± 3.4ab	2.61 ± 0.21de	0.95 ± 0.07e	20.7 ± 1.3fg	16.2 ± 1.0b	0.57 ± 0.05i	2.49 ± 0.15de	0.089 ± 0.006b
SN-LE-035	Z3	30.7 ± 2.4hi	2.43 ± 0.19f	1.07 ± 0.06b	20.6 ± 1.4fg	16.1 ± 1.4bc	0.82 ± 0.05bc	2.79 ± 0.21c	0.073 ± 0.005e
SN-LE-036	Z3	36.7 ± 3.1f	2.50 ± 0.16ef	1.18 ± 0.09ab	25.6 ± 2.3bc	15.5 ± 1.3d	0.59 ± 0.04h	2.19 ± 0.12f	0.081 ± 0.006c
SN-LE-037	Z3	36.8 ± 3.3ef	2.63 ± 0.23de	0.81 ± 0.05g	24.0 ± 1.7c	17.4 ± 1.1ab	0.69 ± 0.06e	3.00 ± 0.23ab	0.074 ± 0.006de
SN-LE-038	Z3	44.1 ± 3.0bc	2.06 ± 0.16h	0.76 ± 0.06h	19.1 ± 1.2hi	17.5 ± 1.4ab	0.65 ± 0.05f	2.07 ± 0.12gh	0.074 ± 0.006e
SN-LE-039	Z3	29.7 ± 1.8i	3.11 ± 0.18b	0.84 ± 0.06fg	20.4 ± 1.6g	14.6 ± 1.2e	0.73 ± 0.06cd	2.06 ± 0.12h	0.063 ± 0.005g
SN-LE-040	Z3	36.2 ± 2.1f	2.91 ± 0.23c	0.99 ± 0.06de	23.4 ± 1.7d	14.1 ± 1.0f	0.59 ± 0.03h	2.89 ± 0.22b	0.087 ± 0.005b
SN-LE-041	Z3	36.5 ± 2.8f	1.97 ± 0.12hi	1.15 ± 0.09ab	26.0 ± 1.7b	16.9 ± 1.3b	0.61 ± 0.05g	2.85 ± 0.21bc	0.073 ± 0.006e
SN-LE-058	Z3	32.6 ± 2.6gh	1.99 ± 0.12h	1.00 ± 0.08d	27.0 ± 1.6ab	14.7 ± 1.0e	0.67 ± 0.06ef	2.87 ± 0.18bc	0.058 ± 0.004h
SN-LE-060	Z3	42.2 ± 2.4cd	2.56 ± 0.17e	1.04 ± 0.08c	19.3 ± 1.3hi	15.6 ± 1.0cd	0.76 ± 0.06cd	1.99 ± 0.14hi	0.092 ± 0.007ab
SN-LE-089	Z3	45.8 ± 3.4b	2.98 ± 0.21bc	1.14 ± 0.07ab	25.4 ± 1.9bc	16.2 ± 1.4b	0.82 ± 0.07b	2.14 ± 0.15fg	0.092 ± 0.006b
SN-LE-090	Z3	30.2 ± 2.4hi	3.38 ± 0.24ab	0.93 ± 0.06e	22.5 ± 1.7e	18.2 ± 1.0a	0.73 ± 0.06d	1.94 ± 0.15i	0.077 ± 0.005d
SN-LE-091	Z3	34.9 ± 3.1fg	2.87 ± 0.24c	1.14 ± 0.07ab	24.5 ± 1.5bc	15.7 ± 1.3c	0.70 ± 0.06de	2.95 ± 0.22ab	0.065 ± 0.004fg
SN-LE-002	Z4	19.1 ± 1.5jk	1.74 ± 0.14ij	0.57 ± 0.05j	14.1 ± 1.0k	10.0 ± 0.9jk	0.39 ± 0.03k	2.12 ± 0.12g	0.054 ± 0.004ij
SN-LE-009	Z4	24.1 ± 2.0j	1.60 ± 0.11j	0.50 ± 0.04k	15.7 ± 1.3jk	10.9 ± 0.9j	0.57 ± 0.05hi	1.68 ± 0.14j	0.034 ± 0.003k
SN-LE-010	Z4	26.9 ± 1.8i	1.50 ± 0.13jk	0.70 ± 0.05i	20.1 ± 1.8g	13.2 ± 1.0h	0.48 ± 0.03jk	1.56 ± 0.12k	0.055 ± 0.005i
SN-LE-011	Z4	25.0 ± 1.7ij	1.95 ± 0.13hi	0.82 ± 0.07g	16.1 ± 1.1j	11.1 ± 0.9j	0.48 ± 0.04jk	1.99 ± 0.17hi	0.055 ± 0.003i
SN-LE-012	Z4	17.7 ± 1.5k	1.57 ± 0.13j	0.65 ± 0.05ij	20.9 ± 1.9f	9.3 ± 0.6k	0.54 ± 0.04ij	1.86 ± 0.10i	0.053 ± 0.003ij
SN-LE-013	Z4	15.9 ± 0.9k	1.55 ± 0.09jk	0.73 ± 0.05hi	15.0 ± 1.1jk	11.4 ± 0.7ij	0.55 ± 0.04i	2.15 ± 0.15fg	0.050 ± 0.004j
SN-LE-015	Z4	22.9 ± 1.4j	1.70 ± 0.10j	0.48 ± 0.04k	17.3 ± 1.0ij	14.5 ± 1.2ef	0.62 ± 0.04g	2.04 ± 0.13h	0.062 ± 0.004g

SN-LE-016	Z4	32.5 ± 1.9^{gh}	1.98 ± 0.17^{hi}	0.58 ± 0.05^{j}	14.5 ± 1.3^{jk}	10.6 ± 0.7^{jk}	0.48 ± 0.03^{jk}	1.63 ± 0.15^{jk}	0.058 ± 0.004^{h}
SN-LE-017	Z4	28.9 ± 2.5^{i}	1.28 ± 0.09^{k}	0.77 ± 0.05^{h}	19.3 ± 1.3^{hi}	14.2 ± 1.1^{f}	0.45 ± 0.04^{k}	1.63 ± 0.11^{j}	0.045 ± 0.003^{jk}
SN-LE-021	Z4	19.8 ± 1.3^{jk}	1.20 ± 0.07^{k}	0.61 ± 0.04^{j}	17.4 ± 1.0^{ij}	11.5 ± 0.9^{ij}	0.60 ± 0.04^{gh}	2.10 ± 0.15^{g}	0.065 ± 0.005^{f}
SN-LE-024	Z4	22.5 ± 1.8^{j}	1.62 ± 0.10^{j}	0.52 ± 0.04^{jk}	13.3 ± 0.8^{k}	11.4 ± 0.8^{ij}	0.47 ± 0.03^{k}	2.16 ± 0.13^{f}	0.037 ± 0.002^{k}
SN-LE-025	Z4	17.5 ± 1.3^{k}	1.98 ± 0.17^{h}	0.75 ± 0.05^{hi}	19.2 ± 1.3^{hi}	9.3 ± 0.7^{k}	0.65 ± 0.05^{f}	1.57 ± 0.14^{jk}	0.037 ± 0.003^{k}
SN-LE-028	Z4	24.4 ± 1.4^{ij}	1.86 ± 0.13^{i}	0.45 ± 0.04^{k}	17.5 ± 1.3^{ij}	11.0 ± 0.8^{j}	0.52 ± 0.04^{j}	1.83 ± 0.14^{ij}	0.060 ± 0.005^{h}
SN-LE-030	Z4	30.7 ± 2.5^{hi}	1.57 ± 0.10^{j}	0.54 ± 0.03^{jk}	17.6 ± 1.4^{ij}	13.5 ± 1.0^{g}	0.48 ± 0.03^{jk}	1.61 ± 0.12^{jk}	0.057 ± 0.003^{hi}
SN-LE-031	Z4	30.5 ± 2.3^{hi}	1.52 ± 0.10^{jk}	0.57 ± 0.04^{j}	13.7 ± 0.8^{k}	10.9 ± 1.0^{jk}	0.51 ± 0.04^{j}	1.81 ± 0.13^{ij}	0.061 ± 0.005^{gh}
SN-LE-070	Z4	30.9 ± 2.3^{h}	1.42 ± 0.11^{k}	0.62 ± 0.05^{ij}	13.5 ± 1.1^{k}	9.0 ± 0.7^{k}	0.63 ± 0.06^{fg}	1.84 ± 0.10^{ij}	0.045 ± 0.004^{jk}
SN-LE-071	Z4	18.8 ± 1.1^{jk}	1.85 ± 0.12^{i}	0.79 ± 0.06^{g}	15.2 ± 1.3^{jk}	9.6 ± 0.9^{k}	0.55 ± 0.04^{ij}	2.06 ± 0.18^{h}	0.040 ± 0.003^{jk}
SN-LE-072	Z4	27.3 ± 1.6^{i}	1.86 ± 0.11^{i}	0.51 ± 0.04^{jk}	16.0 ± 1.3^{j}	11.1 ± 1.0^{j}	0.46 ± 0.04^{k}	1.44 ± 0.12^{k}	0.035 ± 0.002^{k}
SN-LE-042	Z5	35.5 ± 3.1^{fg}	2.21 ± 0.20^{gh}	0.89 ± 0.06^{ef}	19.8 ± 1.5^{gh}	10.7 ± 0.7^{jk}	0.79 ± 0.07^{c}	2.05 ± 0.12^{h}	0.055 ± 0.004^{i}
SN-LE-043	Z5	41.6 ± 3.6^{cd}	2.31 ± 0.20^{g}	0.96 ± 0.06^{e}	18.2 ± 1.2^{i}	15.7 ± 1.1^{c}	0.63 ± 0.05^{g}	2.18 ± 0.13^{f}	0.057 ± 0.004^{hi}
SN-LE-044	Z5	24.5 ± 1.6^{ij}	2.45 ± 0.17^{ef}	1.00 ± 0.08^{d}	22.2 ± 1.8^{ef}	15.1 ± 1.0^{de}	0.68 ± 0.05^{ef}	1.75 ± 0.15^{j}	0.076 ± 0.005^{d}
SN-LE-045	Z5	33.9 ± 2.3^{g}	1.96 ± 0.16^{hi}	0.76 ± 0.05^{h}	23.9 ± 1.8^{cd}	13.5 ± 1.1^{g}	0.72 ± 0.06^{d}	2.28 ± 0.15^{e}	0.061 ± 0.005^{gh}
SN-LE-046	Z5	35.6 ± 2.1^{fg}	2.41 ± 0.14^{f}	0.95 ± 0.06^{e}	22.9 ± 1.4^{de}	15.8 ± 1.3^{c}	0.58 ± 0.04^{hi}	2.50 ± 0.14^{de}	0.051 ± 0.004^{j}
SN-LE-047	Z5	37.0 ± 2.8^{ef}	2.58 ± 0.18^{e}	0.72 ± 0.04^{i}	22.0 ± 1.5^{ef}	12.1 ± 1.0^{i}	0.67 ± 0.05^{ef}	1.97 ± 0.14^{i}	0.056 ± 0.005^{i}
SN-LE-066	Z5	32.1 ± 2.8^{h}	1.77 ± 0.11^{ij}	0.84 ± 0.06^{fg}	19.7 ± 1.3^{gh}	12.5 ± 1.0^{i}	0.55 ± 0.05^{ij}	2.26 ± 0.16^{f}	0.056 ± 0.005^{hi}
SN-LE-067	Z5	39.2 ± 3.0^{de}	2.37 ± 0.17^{fg}	0.80 ± 0.06^{g}	22.9 ± 1.6^{de}	14.4 ± 0.9^{ef}	0.59 ± 0.04^{h}	2.26 ± 0.18^{ef}	0.074 ± 0.006^{de}
SN-LE-068	Z5	22.0 ± 1.3^{j}	2.40 ± 0.14^{fg}	0.77 ± 0.05^{h}	19.5 ± 1.3^{h}	14.7 ± 1.0^{e}	0.72 ± 0.06^{d}	2.39 ± 0.15^{e}	0.064 ± 0.005^{fg}
SN-LE-073	Z5	32.6 ± 2.7^{gh}	2.46 ± 0.14^{ef}	0.68 ± 0.04^{i}	19.3 ± 1.4^{h}	14.0 ± 1.2^{fg}	0.56 ± 0.05^{i}	2.10 ± 0.14^{g}	0.061 ± 0.004^{h}
SN-LE-074	Z5	38.2 ± 2.2^{e}	2.78 ± 0.22^{cd}	0.97 ± 0.07^{de}	19.8 ± 1.3^{gh}	13.2 ± 1.2^{h}	0.64 ± 0.05^{fg}	2.45 ± 0.14^{de}	0.051 ± 0.003^{ij}
SN-LE-075	Z5	27.3 ± 1.8^{i}	2.96 ± 0.24^{bc}	1.00 ± 0.07^{d}	24.4 ± 1.9^{c}	14.4 ± 0.9^{ef}	0.68 ± 0.05^{ef}	2.11 ± 0.16^{g}	0.076 ± 0.004^{d}
SN-LE-076	Z5	36.9 ± 2.3^{ef}	2.17 ± 0.16^{gh}	0.85 ± 0.07^{fg}	20.1 ± 1.4^{g}	13.8 ± 1.0^{g}	0.59 ± 0.05^{h}	2.00 ± 0.17^{h}	0.074 ± 0.005^{e}
SN-LE-077	Z5	33.1 ± 1.9^{g}	2.68 ± 0.15^{d}	0.69 ± 0.05^{i}	23.2 ± 1.5^{de}	12.9 ± 0.9^{h}	0.59 ± 0.04^{h}	2.61 ± 0.15^{d}	0.073 ± 0.005^{e}
SN-LE-078	Z5	32.8 ± 1.9^{gh}	2.09 ± 0.18^{h}	1.01 ± 0.06^{cd}	21.5 ± 1.2^{f}	13.2 ± 1.1^{gh}	0.68 ± 0.05^{e}	1.67 ± 0.12^{j}	0.080 ± 0.007^{c}
SN-LE-079	Z5	37.6 ± 2.9^{e}	2.12 ± 0.15^{h}	0.79 ± 0.05^{gh}	17.0 ± 1.0^{j}	13.5 ± 1.2^{g}	0.64 ± 0.04^{f}	1.97 ± 0.16^{hi}	0.064 ± 0.006^{fg}
SN-LE-080	Z5	36.9 ± 2.8^{ef}	2.20 ± 0.18^{gh}	0.67 ± 0.05^{i}	20.6 ± 1.6^{fg}	13.2 ± 1.0^{gh}	0.60 ± 0.04^{gh}	2.13 ± 0.13^{fg}	0.068 ± 0.006^{ef}

SN-LE-092	Z5	32.3 ± 2.6^{h}	2.22 ± 0.18^{g}	0.98 ± 0.08^{de}	19.4 ± 1.4^{h}	12.8 ± 1.0^{hi}	0.60 ± 0.03^{gh}	2.26 ± 0.14^{ef}	0.057 ± 0.005^{hi}
SN-LE-093	Z5	35.4 ± 2.2^{fg}	1.76 ± 0.14^{ij}	1.03 ± 0.07^{c}	22.6 ± 2.0^{e}	12.8 ± 0.8^{hi}	0.60 ± 0.04^{g}	1.88 ± 0.13^{i}	0.061 ± 0.004^{gh}

Notes: Values are reported as mean ± analytical SD of three independently prepared analytical portions on a dry-weight basis (mg/kg DW). The three analytical measurements were used only to assess analytical precision and were averaged to obtain one site-level concentration per matrix and georeferenced sampling site for subsequent statistical analyses. Values <LOD and <LOQ are indicated as applicable.

Table S22. Individual concentrations of potentially toxic and environmentally relevant trace elements measured in georeferenced *Sambucus nigra* leaf samples collected across the investigated ecological sectors of the Oradea metropolitan area, Romania.

Sample ID	Zone	^{208}Pb (mg/kg)	^{111}Cd (mg/kg)	^{75}As (mg/kg)	^{202}Hg (mg/kg)	^{52}Cr (mg/kg)	^{60}Ni (mg/kg)	^{121}Sb (mg/kg)	^{118}Sn (mg/kg)
SN-LE-022	Z1	<LOD	<LOD	<LOD	<LOD	0.24 ± 0.015^{f}	0.17 ± 0.021^{g}	0.0041 ± 0.00035^{h}	<LOD
SN-LE-023	Z1	<LOD	<LOD	<LOD	<LOD	0.22 ± 0.015^{f}	0.20 ± 0.012^{f}	<LOD	0.005 ± 0.0005^{gh}
SN-LE-026	Z1	<LOD	<LOD	<LOD	<LOD	0.22 ± 0.023^{f}	0.16 ± 0.014^{g}	<LOD	0.008 ± 0.0004^{f}
SN-LE-027	Z1	<LOD	<LOD	<LOD	<LOD	0.37 ± 0.023^{d}	0.12 ± 0.012^{h}	<LOD	0.006 ± 0.0003^{g}
SN-LE-029	Z1	<LOD	<LOD	<LOD	<LOD	0.30 ± 0.028^{e}	0.19 ± 0.019^{fg}	<LOD	<LOD
SN-LE-053	Z1	<LOD	<LOD	<LOD	<LOD	0.18 ± 0.015^{g}	0.12 ± 0.008^{h}	<LOD	0.005 ± 0.0005^{h}
SN-LE-061	Z1	<LOD	<LOD	<LOD	<LOD	0.17 ± 0.018^{g}	0.27 ± 0.032^{ef}	<LOD	0.005 ± 0.0004^{gh}
SN-LE-062	Z1	<LOD	<LOD	<LOD	<LOD	0.20 ± 0.011^{f}	0.11 ± 0.013^{h}	0.0044 ± 0.00045^{h}	0.006 ± 0.0007^{g}
SN-LE-063	Z1	0.039 ± 0.0049^{h}	<LOD	<LOD	<LOD	0.20 ± 0.022^{f}	0.24 ± 0.024^{f}	<LOD	0.006 ± 0.0004^{g}
SN-LE-081	Z1	0.043 ± 0.0046^{h}	<LOD	0.009 ± 0.0010^{h}	<LOD	0.17 ± 0.009^{g}	0.18 ± 0.016^{fg}	<LOD	0.009 ± 0.0008^{f}
SN-LE-082	Z1	<LOD	<LOD	<LOD	<LOD	0.13 ± 0.014^{h}	0.13 ± 0.010^{gh}	<LOD	0.007 ± 0.0004^{fg}
SN-LE-083	Z1	<LOD	<LOD	<LOD	0.0012 ± 0.00011^{gh}	0.23 ± 0.015^{f}	0.14 ± 0.015^{g}	<LOD	0.005 ± 0.0005^{gh}
SN-LE-084	Z1	<LOD	<LOD	0.010 ± 0.0013^{gh}	<LOD	0.15 ± 0.018^{gh}	0.19 ± 0.011^{f}	<LOD	0.004 ± 0.0004^{h}
SN-LE-003	Z2	0.211 ± 0.0188^{ab}	0.0213 ± 0.00175^{b}	<LOD	<LOD	0.42 ± 0.045^{cd}	0.47 ± 0.058^{c}	0.0102 ± 0.00079^{bc}	0.019 ± 0.0011^{bc}
SN-LE-004	Z2	<LOD	<LOD	0.020 ± 0.0019^{cd}	<LOD	0.82 ± 0.084^{a}	0.63 ± 0.066^{b}	<LOD	0.021 ± 0.0023^{ab}
SN-LE-014	Z2	0.211 ± 0.0133^{ab}	0.0197 ± 0.00172^{bc}	0.039 ± 0.0024^{a}	0.0016 ± 0.00017^{cd}	0.82 ± 0.096^{a}	0.71 ± 0.035^{ab}	0.0108 ± 0.00079^{ab}	<LOD
SN-LE-018	Z2	0.230 ± 0.0229^{a}	<LOD	0.021 ± 0.0026^{c}	0.0010 ± 0.00005^{h}	0.54 ± 0.062^{b}	0.84 ± 0.066^{a}	<LOD	0.019 ± 0.0024^{bc}
SN-LE-019	Z2	0.095 ± 0.0100^{fg}	0.0176 ± 0.00185^{cd}	0.025 ± 0.0020^{b}	<LOD	0.84 ± 0.073^{a}	0.88 ± 0.106^{a}	<LOD	<LOD
SN-LE-020	Z2	0.135 ± 0.0074^{de}	0.0164 ± 0.00192^{de}	0.023 ± 0.0024^{c}	0.0019 ± 0.00024^{ab}	0.70 ± 0.041^{ab}	0.55 ± 0.064^{bc}	0.0059 ± 0.00070^{fg}	0.017 ± 0.0015^{cd}

SN-LE-048	Z2	0.214 ± 0.0218^{ab}	0.0148 ± 0.00100^{e}	0.028 ± 0.0017^{ab}	<LOD	0.50 ± 0.062^{b}	0.47 ± 0.058^{c}	0.0099 ± 0.00076^{bc}	0.014 ± 0.0007^{de}
SN-LE-049	Z2	0.190 ± 0.0162^{c}	<LOD	0.018 ± 0.0023^{d}	<LOD	0.42 ± 0.036^{cd}	0.61 ± 0.073^{b}	0.0146 ± 0.00151^{a}	0.020 ± 0.0019^{ab}
SN-LE-050	Z2	0.204 ± 0.0225^{bc}	0.0240 ± 0.00144^{b}	<LOD	<LOD	0.56 ± 0.056^{b}	0.71 ± 0.069^{ab}	0.0108 ± 0.00055^{ab}	0.020 ± 0.0014^{ab}
SN-LE-051	Z2	<LOD	0.0153 ± 0.00171^{de}	0.026 ± 0.0026^{b}	0.0013 ± 0.00017^{ef}	0.48 ± 0.046^{bc}	0.33 ± 0.027^{e}	0.0099 ± 0.00072^{bc}	0.017 ± 0.0021^{cd}
SN-LE-052	Z2	0.167 ± 0.0193^{d}	0.0253 ± 0.00256^{ab}	0.023 ± 0.0018^{bc}	<LOD	0.84 ± 0.056^{a}	0.45 ± 0.044^{cd}	0.0074 ± 0.00042^{de}	0.017 ± 0.0017^{cd}
SN-LE-054	Z2	0.179 ± 0.0200^{cd}	<LOD	<LOD	<LOD	0.50 ± 0.032^{b}	0.65 ± 0.037^{b}	0.0060 ± 0.00045^{fg}	0.028 ± 0.0023^{a}
SN-LE-055	Z2	<LOD	<LOD	<LOD	<LOD	0.99 ± 0.056^{a}	0.91 ± 0.061^{a}	<LOD	<LOD
SN-LE-056	Z2	0.227 ± 0.0152^{a}	<LOD	0.023 ± 0.0020^{bc}	<LOD	0.72 ± 0.034^{a}	0.78 ± 0.061^{a}	0.0114 ± 0.00101^{a}	0.030 ± 0.0034^{a}
SN-LE-057	Z2	0.221 ± 0.0199^{a}	0.0149 ± 0.00142^{e}	<LOD	<LOD	0.46 ± 0.032^{bc}	0.43 ± 0.046^{d}	0.0069 ± 0.00056^{ef}	0.022 ± 0.0016^{a}
SN-LE-059	Z2	0.140 ± 0.0075^{de}	0.0255 ± 0.00155^{ab}	0.030 ± 0.0020^{a}	<LOD	0.73 ± 0.090^{a}	0.65 ± 0.067^{b}	<LOD	0.019 ± 0.0010^{bc}
SN-LE-064	Z2	0.204 ± 0.0211^{bc}	<LOD	<LOD	<LOD	1.28 ± 0.159^{a}	0.85 ± 0.053^{a}	0.0061 ± 0.00066^{fg}	0.030 ± 0.0015^{a}
SN-LE-065	Z2	0.281 ± 0.0221^{a}	0.0197 ± 0.00232^{bc}	0.017 ± 0.0015^{de}	<LOD	0.64 ± 0.034^{b}	0.40 ± 0.034^{d}	<LOD	0.038 ± 0.0024^{a}
SN-LE-069	Z2	0.178 ± 0.0158^{cd}	0.0268 ± 0.00285^{a}	0.023 ± 0.0011^{bc}	<LOD	0.43 ± 0.023^{cd}	0.83 ± 0.047^{a}	0.0110 ± 0.00133^{ab}	0.012 ± 0.0013^{f}
SN-LE-085	Z2	0.187 ± 0.0130^{c}	<LOD	<LOD	<LOD	0.66 ± 0.036^{b}	0.80 ± 0.044^{a}	<LOD	0.025 ± 0.0017^{a}
SN-LE-086	Z2	0.232 ± 0.0198^{a}	0.0107 ± 0.00055^{fg}	0.011 ± 0.0006^{fg}	<LOD	0.46 ± 0.024^{bc}	0.98 ± 0.079^{a}	<LOD	0.018 ± 0.0011^{bc}
SN-LE-087	Z2	0.192 ± 0.0223^{bc}	0.0275 ± 0.00245^{a}	0.035 ± 0.0033^{a}	0.0020 ± 0.00010^{ab}	0.98 ± 0.100^{a}	0.77 ± 0.052^{a}	<LOD	0.017 ± 0.0013^{cd}
SN-LE-088	Z2	0.102 ± 0.0090^{ef}	0.0178 ± 0.00205^{cd}	0.016 ± 0.0012^{de}	0.0016 ± 0.00016^{cd}	0.39 ± 0.042^{d}	0.92 ± 0.052^{a}	<LOD	0.029 ± 0.0027^{a}
SN-LE-094	Z2	0.208 ± 0.0121^{ab}	0.0167 ± 0.00122^{d}	0.039 ± 0.0019^{a}	<LOD	0.86 ± 0.049^{a}	1.08 ± 0.061^{a}	0.0085 ± 0.00069^{cd}	<LOD
SN-LE-001	Z3	<LOD	0.0088 ± 0.00072^{g}	<LOD	<LOD	0.43 ± 0.044^{cd}	0.29 ± 0.021^{e}	<LOD	0.019 ± 0.0022^{bc}
SN-LE-005	Z3	<LOD	<LOD	<LOD	<LOD	0.71 ± 0.052^{ab}	0.31 ± 0.023^{e}	<LOD	0.011 ± 0.0010^{f}
SN-LE-006	Z3	0.103 ± 0.0104^{ef}	<LOD	<LOD	<LOD	0.33 ± 0.024^{e}	0.35 ± 0.044^{e}	<LOD	0.012 ± 0.0008^{ef}
SN-LE-007	Z3	0.066 ± 0.0057^{gh}	<LOD	<LOD	<LOD	0.45 ± 0.041^{c}	0.24 ± 0.018^{f}	0.0055 ± 0.00030^{g}	0.017 ± 0.0011^{cd}
SN-LE-008	Z3	<LOD	0.0076 ± 0.00060^{gh}	0.010 ± 0.0011^{gh}	<LOD	0.44 ± 0.029^{c}	0.53 ± 0.028^{bc}	<LOD	0.013 ± 0.0013^{e}
SN-LE-032	Z3	0.072 ± 0.0086^{g}	<LOD	<LOD	<LOD	0.21 ± 0.010^{f}	0.12 ± 0.013^{h}	<LOD	0.006 ± 0.0007^{g}
SN-LE-033	Z3	<LOD	0.0062 ± 0.00073^{h}	0.014 ± 0.0008^{e}	<LOD	0.16 ± 0.020^{g}	0.19 ± 0.012^{fg}	<LOD	0.007 ± 0.0005^{fg}
SN-LE-034	Z3	<LOD	0.0122 ± 0.00098^{f}	0.012 ± 0.0014^{ef}	<LOD	0.19 ± 0.012^{fg}	0.23 ± 0.026^{f}	<LOD	<LOD
SN-LE-035	Z3	<LOD	0.0116 ± 0.00063^{f}	0.011 ± 0.0014^{fg}	<LOD	0.44 ± 0.029^{c}	0.36 ± 0.036^{e}	<LOD	0.013 ± 0.0008^{ef}
SN-LE-036	Z3	0.060 ± 0.0063^{h}	<LOD	0.011 ± 0.0012^{fg}	<LOD	0.36 ± 0.036^{de}	0.38 ± 0.020^{de}	<LOD	<LOD

SN-LE-037	Z3	0.099 ± 0.0046[fg]	<LOD	<LOD	<LOD	0.32 ± 0.025[e]	0.42 ± 0.041[d]	<LOD	<LOD
SN-LE-038	Z3	<LOD	<LOD	<LOD	<LOD	0.37 ± 0.046[d]	0.55 ± 0.044[bc]	<LOD	0.016 ± 0.0015[cd]
SN-LE-039	Z3	0.095 ± 0.0045[fg]	<LOD	0.012 ± 0.0011[f]	<LOD	0.42 ± 0.031[cd]	0.28 ± 0.030[e]	<LOD	<LOD
SN-LE-040	Z3	<LOD	<LOD	<LOD	<LOD	0.30 ± 0.036[e]	0.40 ± 0.038[d]	<LOD	<LOD
SN-LE-041	Z3	<LOD	<LOD	0.008 ± 0.0005[h]	<LOD	0.41 ± 0.043[cd]	0.25 ± 0.018[f]	<LOD	0.011 ± 0.0009[f]
SN-LE-058	Z3	0.098 ± 0.0051[fg]	<LOD	<LOD	<LOD	0.44 ± 0.055[c]	0.42 ± 0.052[d]	<LOD	0.014 ± 0.0014[de]
SN-LE-060	Z3	<LOD	<LOD	<LOD	<LOD	0.69 ± 0.082[ab]	0.35 ± 0.021[e]	<LOD	0.013 ± 0.0016[e]
SN-LE-089	Z3	0.126 ± 0.0074[e]	<LOD	<LOD	<LOD	0.25 ± 0.030[ef]	0.27 ± 0.022[ef]	<LOD	0.012 ± 0.0005[f]
SN-LE-090	Z3	0.059 ± 0.0049[h]	<LOD	<LOD	<LOD	0.30 ± 0.036[e]	0.50 ± 0.034[c]	<LOD	0.017 ± 0.0021[c]
SN-LE-091	Z3	<LOD	<LOD	<LOD	<LOD	0.35 ± 0.031[de]	0.54 ± 0.031[bc]	<LOD	0.007 ± 0.0003[g]
SN-LE-002	Z4	<LOD	<LOD	<LOD	<LOD	0.11 ± 0.009[h]	0.16 ± 0.015[g]	<LOD	0.004 ± 0.0005[h]
SN-LE-009	Z4	<LOD	<LOD	<LOD	<LOD	0.18 ± 0.014[g]	0.13 ± 0.007[h]	<LOD	<LOD
SN-LE-010	Z4	<LOD	<LOD	<LOD	<LOD	0.10 ± 0.011[h]	0.18 ± 0.020[fg]	<LOD	<LOD
SN-LE-011	Z4	<LOD	<LOD	<LOD	<LOD	0.16 ± 0.017[g]	0.14 ± 0.015[gh]	<LOD	<LOD
SN-LE-012	Z4	<LOD	<LOD	<LOD	<LOD	0.15 ± 0.017[gh]	0.18 ± 0.011[fg]	<LOD	0.005 ± 0.0003[gh]
SN-LE-013	Z4	0.042 ± 0.0042[h]	<LOD	<LOD	<LOD	0.12 ± 0.012[h]	0.14 ± 0.014[g]	<LOD	<LOD
SN-LE-015	Z4	<LOD	<LOD	<LOD	<LOD	0.20 ± 0.012[f]	0.17 ± 0.020[g]	<LOD	0.005 ± 0.0005[gh]
SN-LE-016	Z4	<LOD	<LOD	<LOD	<LOD	0.10 ± 0.011[h]	0.10 ± 0.005[h]	<LOD	<LOD
SN-LE-017	Z4	<LOD	<LOD	<LOD	<LOD	0.09 ± 0.008[h]	<LOD	<LOD	<LOD
SN-LE-021	Z4	<LOD	<LOD	<LOD	<LOD	0.10 ± 0.009[h]	0.16 ± 0.014[g]	<LOD	<LOD
SN-LE-024	Z4	<LOD	<LOD	<LOD	<LOD	0.15 ± 0.017[g]	0.13 ± 0.010[h]	<LOD	<LOD
SN-LE-025	Z4	<LOD	<LOD	<LOD	<LOD	0.13 ± 0.006[h]	0.07 ± 0.006[h]	<LOD	<LOD
SN-LE-028	Z4	<LOD	<LOD	<LOD	<LOD	0.17 ± 0.017[g]	0.11 ± 0.010[h]	<LOD	<LOD
SN-LE-030	Z4	<LOD	<LOD	<LOD	<LOD	0.15 ± 0.014[g]	0.10 ± 0.010[h]	<LOD	<LOD
SN-LE-031	Z4	<LOD	<LOD	<LOD	<LOD	0.15 ± 0.012[gh]	0.19 ± 0.022[f]	<LOD	0.004 ± 0.0004[h]
SN-LE-070	Z4	<LOD	<LOD	<LOD	<LOD	0.11 ± 0.013[h]	0.08 ± 0.004[h]	<LOD	<LOD
SN-LE-071	Z4	<LOD	<LOD	<LOD	<LOD	0.14 ± 0.007[h]	0.13 ± 0.011[gh]	<LOD	<LOD
SN-LE-072	Z4	<LOD	<LOD	0.007 ± 0.0006[h]	<LOD	0.08 ± 0.006[h]	0.19 ± 0.020[fg]	<LOD	0.006 ± 0.0005[g]

SN-LE-042	Z5	<LOD	0.0098 ± 0.00074^{g}	<LOD	<LOD	0.34 ± 0.035^{e}	0.43 ± 0.045^{d}	0.0039 ± 0.00019^{h}	<LOD
SN-LE-043	Z5	<LOD	<LOD	<LOD	0.0013 ± 0.00006^{fg}	0.35 ± 0.041^{de}	0.42 ± 0.031^{d}	<LOD	0.029 ± 0.0035^{a}
SN-LE-044	Z5	<LOD	<LOD	<LOD	<LOD	0.34 ± 0.034^{de}	0.47 ± 0.038^{c}	<LOD	<LOD
SN-LE-045	Z5	0.179 ± 0.0214^{cd}	0.0077 ± 0.00056^{gh}	0.010 ± 0.0009^{gh}	<LOD	0.40 ± 0.042^{d}	0.42 ± 0.032^{d}	<LOD	<LOD
SN-LE-046	Z5	0.188 ± 0.0196^{c}	<LOD	<LOD	<LOD	0.42 ± 0.021^{cd}	0.45 ± 0.045^{cd}	0.0070 ± 0.00082^{ef}	0.014 ± 0.0009^{de}
SN-LE-047	Z5	<LOD	<LOD	0.018 ± 0.0016^{d}	<LOD	0.60 ± 0.033^{b}	0.50 ± 0.039^{c}	<LOD	0.019 ± 0.0010^{b}
SN-LE-066	Z5	0.099 ± 0.0051^{fg}	<LOD	<LOD	<LOD	0.32 ± 0.027^{e}	0.59 ± 0.049^{b}	<LOD	0.014 ± 0.0008^{de}
SN-LE-067	Z5	<LOD	<LOD	<LOD	<LOD	0.43 ± 0.045^{cd}	0.41 ± 0.025^{d}	<LOD	0.013 ± 0.0006^{ef}
SN-LE-068	Z5	0.120 ± 0.0135^{e}	0.0076 ± 0.00078^{gh}	<LOD	<LOD	0.25 ± 0.022^{ef}	0.67 ± 0.072^{b}	<LOD	<LOD
SN-LE-073	Z5	0.065 ± 0.0053^{gh}	<LOD	0.011 ± 0.0012^{fg}	<LOD	0.44 ± 0.027^{c}	0.28 ± 0.028^{e}	<LOD	0.013 ± 0.0013^{ef}
SN-LE-074	Z5	<LOD	<LOD	<LOD	<LOD	0.48 ± 0.033^{bc}	0.39 ± 0.021^{de}	<LOD	0.016 ± 0.0012^{cd}
SN-LE-075	Z5	0.107 ± 0.0052^{e}	<LOD	<LOD	<LOD	0.35 ± 0.028^{de}	0.43 ± 0.035^{cd}	0.0071 ± 0.00061^{de}	0.015 ± 0.0010^{d}
SN-LE-076	Z5	<LOD	<LOD	0.012 ± 0.0010^{ef}	<LOD	0.50 ± 0.045^{b}	0.69 ± 0.077^{ab}	<LOD	0.019 ± 0.0008^{bc}
SN-LE-077	Z5	0.099 ± 0.0092^{fg}	<LOD	<LOD	<LOD	0.49 ± 0.025^{bc}	0.47 ± 0.058^{c}	0.0077 ± 0.00051^{d}	0.019 ± 0.0010^{bc}
SN-LE-078	Z5	<LOD	<LOD	0.018 ± 0.0016^{d}	<LOD	0.26 ± 0.031^{ef}	0.46 ± 0.025^{cd}	<LOD	0.014 ± 0.0015^{de}
SN-LE-079	Z5	<LOD	<LOD	<LOD	<LOD	0.24 ± 0.019^{f}	0.30 ± 0.028^{e}	0.0056 ± 0.00055^{g}	0.016 ± 0.0013^{cd}
SN-LE-080	Z5	0.101 ± 0.0065^{ef}	<LOD	0.015 ± 0.0016^{e}	<LOD	0.40 ± 0.038^{d}	0.70 ± 0.076^{ab}	<LOD	0.016 ± 0.0017^{cd}
SN-LE-092	Z5	0.153 ± 0.0171^{d}	<LOD	<LOD	<LOD	0.39 ± 0.029^{d}	0.46 ± 0.023^{c}	<LOD	<LOD
SN-LE-093	Z5	0.110 ± 0.0076^{e}	<LOD	<LOD	<LOD	0.25 ± 0.021^{ef}	0.37 ± 0.037^{de}	<LOD	0.013 ± 0.0007^{e}

Notes: Values are reported as mean ± analytical SD of three independently prepared analytical portions on a dry-weight basis (mg/kg DW). The three analytical measurements were used only to assess analytical precision and were averaged to obtain one site-level concentration per matrix and georeferenced sampling site for subsequent statistical analyses. Values <LOD and <LOQ are indicated as applicable.

Table S23. Individual concentrations of macroelements in *Sambucus nigra* L. flower samples collected from georeferenced sampling points across the investigated ecological sectors of the Oradea metropolitan area, Romania.

Sample ID	Zone	K (mg/kg)	Ca (mg/kg)	Mg (mg/kg)	Na (mg/kg)	P (mg/kg)	S (mg/kg)
SN-FL-022	Z1	19071 ± 980^{g}	6609 ± 350^{ij}	3679 ± 191°	48.8 ± 2.6^{k}	5764 ± 306^{mn}	1956 ± 108^{hi}
SN-FL-023	Z1	18473 ± 937^{jk}	6226 ± 306^{l}	4445 ± 227^{h}	47.9 ± 2.5^{kl}	6447 ± 330^{g}	2140 ± 115^{de}

SN-FL-026	Z1	17561 ± 907^{p}	5969 ± 310^{mn}	3882 ± 198^{m}	43.7 ± 2.3^{o}	6229 ± 336^{i}	1874 ± 101^{jk}
SN-FL-027	Z1	17781 ± 912no	6954 ± 362^{gh}	4009 ± 207^{l}	50.9 ± 2.7^{i}	6030 ± 301^{k}	2152 ± 117^{d}
SN-FL-029	Z1	18285 ± 935^{kl}	6343 ± 328^{k}	3817 ± 201^{n}	49.7 ± 2.6^{j}	6217 ± 333^{i}	2027 ± 107^{g}
SN-FL-053	Z1	18015 ± 927^{m}	6505 ± 351^{j}	3834 ± 192^{mn}	39.4 ± 2.1^{rs}	5966 ± 311^{kl}	1917 ± 102^{ij}
SN-FL-061	Z1	17957 ± 926^{mn}	5847 ± 294no	4266 ± 222^{ij}	49.4 ± 2.5^{j}	5911 ± 314^{l}	1907 ± 104^{j}
SN-FL-062	Z1	17680 ± 913°	5735 ± 297°	4122 ± 218^{k}	48.3 ± 2.5^{k}	6504 ± 329^{f}	1936 ± 99^{i}
SN-FL-063	Z1	17402 ± 862^{q}	5941 ± 305^{n}	4242 ± 212^{j}	47.3 ± 2.5^{l}	6310 ± 317^{h}	2083 ± 114^{ef}
SN-FL-081	Z1	18210 ± 939^{l}	7104 ± 359^{fg}	4431 ± 235^{h}	48.7 ± 2.6^{k}	6090 ± 322^{j}	2015 ± 108^{g}
SN-FL-082	Z1	17369 ± 910^{q}	6246 ± 324^{l}	3782 ± 203^{n}	46.1 ± 2.4^{m}	6636 ± 353^{e}	1500 ± 78st
SN-FL-083	Z1	18048 ± 937^{m}	5226 ± 266^{r}	3570 ± 184^{p}	46.6 ± 2.5^{lm}	5851 ± 312^{lm}	1620 ± 90^{pq}
SN-FL-084	Z1	17438 ± 907^{pq}	6487 ± 346^{jk}	3701 ± 193°	44.8 ± 2.4^{n}	5925 ± 311^{l}	1691 ± 94°
SN-FL-003	Z2	19174 ± 1000^{f}	6974 ± 369^{gh}	3800 ± 200^{n}	51.4 ± 2.8^{hi}	6334 ± 339^{h}	2046 ± 110^{fg}
SN-FL-004	Z2	17629 ± 899^{op}	6820 ± 367^{hi}	4488 ± 234^{gh}	53.4 ± 2.9^{g}	6160 ± 334^{ij}	1824 ± 100^{l}
SN-FL-014	Z2	17815 ± 906^{n}	6895 ± 352^{h}	4771 ± 238^{e}	53.8 ± 2.8^{fg}	6137 ± 330^{j}	2067 ± 114^{f}
SN-FL-018	Z2	19138 ± 996^{fg}	7016 ± 373^{g}	3723 ± 191no	48.9 ± 2.5^{jk}	6600 ± 347^{ef}	1849 ± 102^{k}
SN-FL-019	Z2	18254 ± 936^{kl}	7289 ± 362^{ef}	4060 ± 207^{kl}	51.5 ± 2.8^{hi}	6475 ± 343^{fg}	1839 ± 98^{kl}
SN-FL-020	Z2	17718 ± 949°	6678 ± 352^{i}	4699 ± 244^{ef}	48.1 ± 2.5^{kl}	6298 ± 337^{h}	1883 ± 100^{jk}
SN-FL-048	Z2	18796 ± 961^{hi}	6324 ± 335^{kl}	4600 ± 249^{fg}	52.3 ± 2.8^{h}	6421 ± 344^{g}	1926 ± 104^{ij}
SN-FL-049	Z2	17954 ± 940^{mn}	6539 ± 323^{j}	4158 ± 220^{jk}	48.0 ± 2.5^{kl}	6252 ± 339^{hi}	1971 ± 107^{hi}
SN-FL-050	Z2	18912 ± 1008^{gh}	6661 ± 346^{ij}	4863 ± 243^{d}	51.0 ± 2.8^{i}	6358 ± 348^{gh}	2060 ± 115^{f}
SN-FL-051	Z2	18067 ± 934^{m}	6626 ± 352^{ij}	4085 ± 214^{kl}	48.5 ± 2.6^{k}	6078 ± 311^{jk}	1931 ± 103^{i}
SN-FL-052	Z2	18138 ± 943^{lm}	6839 ± 352^{h}	4549 ± 247^{g}	52.1 ± 2.9^{h}	6518 ± 353^{f}	1982 ± 106^{h}
SN-FL-054	Z2	17694 ± 895°	6713 ± 341^{i}	4365 ± 230^{hi}	50.7 ± 2.7^{i}	6206 ± 340^{i}	1878 ± 102^{jk}
SN-FL-055	Z2	18688 ± 1002^{i}	6766 ± 354^{hi}	4566 ± 240^{g}	50.6 ± 2.7^{i}	6194 ± 328^{i}	2033 ± 109^{g}
SN-FL-056	Z2	18360 ± 962^{k}	7204 ± 378^{f}	4637 ± 237^{f}	50.2 ± 2.7^{ij}	6383 ± 341^{gh}	1791 ± 99^{lm}
SN-FL-057	Z2	18145 ± 941^{lm}	6784 ± 348^{hi}	4290 ± 226^{ij}	53.2 ± 2.8^{g}	6434 ± 347^{g}	2218 ± 118^{bc}
SN-FL-059	Z2	17625 ± 923^{op}	6361 ± 341^{k}	4618 ± 250^{fg}	52.7 ± 2.8^{gh}	6395 ± 335^{gh}	1966 ± 104^{hi}
SN-FL-064	Z2	17895 ± 932^{n}	6522 ± 352^{j}	4206 ± 223^{j}	50.1 ± 2.7^{ij}	6656 ± 346^{e}	1869 ± 100^{k}

SN-FL-065	Z2	17768 ± 912no	6643 ± 334^{ij}	4315 ± 218^{i}	50.5 ± 2.6^{ij}	6346 ± 332^{h}	1912 ± 100^{ij}
SN-FL-069	Z2	19014 ± 998^{g}	7509 ± 391^{d}	4533 ± 235^{g}	58.7 ± 3.0^{bc}	6322 ± 331^{h}	1987 ± 106^{h}
SN-FL-085	Z2	18122 ± 947^{lm}	7178 ± 360^{f}	4657 ± 248^{f}	59.6 ± 3.2^{b}	6802 ± 354^{cd}	2075 ± 114^{f}
SN-FL-086	Z2	18802 ± 976^{hi}	7231 ± 380^{f}	4745 ± 243^{e}	55.6 ± 3.0^{e}	6773 ± 357^{cd}	2040 ± 111^{fg}
SN-FL-087	Z2	19201 ± 1019^{f}	7612 ± 401^{cd}	4583 ± 248^{fg}	58.0 ± 3.1^{c}	6961 ± 376^{b}	2004 ± 105^{gh}
SN-FL-088	Z2	19015 ± 992^{g}	7465 ± 383^{de}	4799 ± 253^{de}	54.0 ± 2.9^{fg}	6721 ± 351^{d}	2281 ± 128^{a}
SN-FL-094	Z2	19915 ± 1049^{bc}	7974 ± 410^{a}	5250 ± 273^{a}	54.6 ± 3.0^{f}	7023 ± 375^{a}	2165 ± 118^{cd}
SN-FL-001	Z3	20450 ± 1075^{a}	7081 ± 382^{g}	4073 ± 214^{kl}	51.8 ± 2.8^{hi}	5992 ± 307^{k}	1992 ± 104^{h}
SN-FL-005	Z3	17806 ± 924no	7059 ± 361^{g}	4327 ± 226^{i}	57.0 ± 3.1^{d}	6698 ± 346^{de}	1785 ± 96^{m}
SN-FL-006	Z3	17868 ± 902^{n}	7558 ± 383^{d}	4048 ± 210^{kl}	61.4 ± 3.2^{a}	7100 ± 373^{a}	2009 ± 108^{gh}
SN-FL-007	Z3	17527 ± 913^{p}	7388 ± 370^{e}	4194 ± 214^{jk}	55.9 ± 3.0^{e}	5679 ± 302^{n}	1829 ± 95^{kl}
SN-FL-008	Z3	18770 ± 982^{hi}	7320 ± 389^{ef}	4418 ± 237^{h}	56.2 ± 3.0^{e}	6275 ± 332^{hi}	2091 ± 115^{ef}
SN-FL-032	Z3	18827 ± 946^{h}	7353 ± 374^{e}	4721 ± 253^{ef}	47.7 ± 2.6^{kl}	5744 ± 295^{mn}	2244 ± 123^{b}
SN-FL-033	Z3	19026 ± 982^{g}	6876 ± 342^{h}	4901 ± 262^{d}	55.1 ± 3.0^{ef}	6834 ± 370^{c}	1922 ± 105^{ij}
SN-FL-034	Z3	20332 ± 1013^{a}	7844 ± 401^{b}	4110 ± 205^{k}	54.8 ± 2.8^{f}	6549 ± 347^{f}	2109 ± 110^{e}
SN-FL-035	Z3	17972 ± 966^{mn}	7425 ± 402^{de}	4996 ± 261^{c}	52.0 ± 2.9^{h}	6370 ± 337^{gh}	1961 ± 107^{hi}
SN-FL-036	Z3	18340 ± 943^{k}	7750 ± 406^{bc}	5061 ± 267^{b}	52.6 ± 2.7^{gh}	6870 ± 358^{c}	1760 ± 96^{mn}
SN-FL-037	Z3	17479 ± 926^{p}	7259 ± 390^{ef}	4677 ± 242^{f}	53.1 ± 2.9^{g}	6148 ± 320^{ij}	2021 ± 111^{g}
SN-FL-038	Z3	17273 ± 859^{qr}	7128 ± 372^{fg}	4518 ± 243^{gh}	49.1 ± 2.6^{jk}	6066 ± 306^{jk}	1747 ± 94^{mn}
SN-FL-039	Z3	18494 ± 982^{j}	8100 ± 416^{a}	4097 ± 208^{k}	57.4 ± 3.0^{d}	6582 ± 356^{ef}	2129 ± 120^{de}
SN-FL-040	Z3	18253 ± 956^{kl}	6995 ± 369^{g}	4134 ± 215^{k}	49.6 ± 2.7^{j}	6911 ± 370^{bc}	1951 ± 105^{i}
SN-FL-041	Z3	17700 ± 899^{o}	7675 ± 398^{c}	4503 ± 230^{gh}	54.4 ± 2.9^{f}	6676 ± 360^{de}	2099 ± 112^{e}
SN-FL-058	Z3	17703 ± 876^{o}	6914 ± 364^{h}	3996 ± 198^{l}	45.9 ± 2.4^{m}	5654 ± 298no	2118 ± 114^{e}
SN-FL-060	Z3	19813 ± 1048^{cd}	7037 ± 364^{g}	4944 ± 255^{cd}	56.6 ± 2.9^{de}	7100 ± 380^{a}	2198 ± 123^{c}
SN-FL-089	Z3	18335 ± 946^{k}	6934 ± 373^{gh}	3927 ± 203^{m}	53.6 ± 2.8^{g}	6171 ± 311^{ij}	1767 ± 96^{m}
SN-FL-090	Z3	17878 ± 926^{n}	6452 ± 329^{jk}	5151 ± 272^{ab}	55.3 ± 2.9^{ef}	6534 ± 338^{f}	2300 ± 123^{a}
SN-FL-091	Z3	18435 ± 941^{jk}	6434 ± 316^{jk}	4830 ± 244^{de}	54.2 ± 2.7^{fg}	5818 ± 307^{m}	1902 ± 101^{j}
SN-FL-002	Z4	17362 ± 881^{q}	5642 ± 281^{op}	4459 ± 224^{h}	52.4 ± 2.8^{h}	5291 ± 283^{r}	1888 ± 103^{jk}

SN-FL-009	Z4	17551 ± 913^{p}	6096 ± 312^{m}	3955 ± 196^{lm}	44.6 ± 2.3^{n}	6125 ± 319^{j}	1660 ± 85^{op}
SN-FL-010	Z4	18296 ± 957^{kl}	6557 ± 334^{j}	3941 ± 205^{lm}	43.4 ± 2.3^{o}	5882 ± 301^{l}	1898 ± 104^{j}
SN-FL-011	Z4	18716 ± 943^{i}	5996 ± 319^{mn}	4146 ± 214^{k}	49.3 ± 2.6^{jk}	5489 ± 287^{p}	1976 ± 103^{h}
SN-FL-012	Z4	17495 ± 863^{p}	6695 ± 336^{i}	3743 ± 188no	40.3 ± 2.2^{qr}	5135 ± 270st	1796 ± 94^{lm}
SN-FL-013	Z4	18379 ± 931^{k}	6119 ± 316^{lm}	4391 ± 226^{hi}	42.0 ± 2.2^{p}	6183 ± 329^{ij}	1648 ± 88^{p}
SN-FL-015	Z4	17527 ± 886^{p}	6266 ± 323^{l}	3655 ± 190^{op}	45.4 ± 2.5^{mn}	6489 ± 334^{fg}	1733 ± 91^{n}
SN-FL-016	Z4	19011 ± 987^{g}	6184 ± 316^{l}	4340 ± 225^{i}	51.1 ± 2.7^{i}	5377 ± 286^{q}	1709 ± 91no
SN-FL-017	Z4	18585 ± 977^{ij}	6305 ± 334^{kl}	4182 ± 211^{jk}	45.2 ± 2.4^{mn}	6102 ± 305^{j}	1808 ± 92^{l}
SN-FL-021	Z4	19322 ± 1000^{e}	6748 ± 346^{i}	4404 ± 235^{hi}	47.0 ± 2.5^{l}	6017 ± 310^{k}	1779 ± 97^{m}
SN-FL-024	Z4	18421 ± 953^{jk}	6072 ± 317^{m}	4352 ± 216^{i}	52.9 ± 2.7^{gh}	6408 ± 334^{g}	1740 ± 93^{n}
SN-FL-025	Z4	19038 ± 975^{g}	6398 ± 332^{k}	3456 ± 178^{q}	46.4 ± 2.5^{lm}	5439 ± 288^{pq}	1864 ± 100^{k}
SN-FL-028	Z4	18325 ± 927^{k}	6022 ± 301^{m}	4170 ± 216^{jk}	45.8 ± 2.4^{m}	6240 ± 319^{i}	1998 ± 109^{gh}
SN-FL-030	Z4	17498 ± 878^{p}	5880 ± 304^{n}	3629 ± 185^{op}	41.6 ± 2.2^{pq}	5939 ± 316^{l}	1813 ± 97^{l}
SN-FL-031	Z4	17765 ± 904no	6731 ± 347^{i}	3897 ± 197^{m}	49.8 ± 2.6^{j}	5782 ± 292^{m}	2053 ± 111^{fg}
SN-FL-070	Z4	18018 ± 897^{m}	5812 ± 315no	3339 ± 179^{r}	47.2 ± 2.5^{l}	5866 ± 308^{lm}	1844 ± 98^{kl}
SN-FL-071	Z4	17894 ± 901^{n}	6380 ± 334^{k}	3601 ± 183^{p}	38.0 ± 2.0st	5566 ± 288^{op}	1854 ± 98^{k}
SN-FL-072	Z4	17741 ± 904no	5450 ± 276^{q}	4035 ± 209^{l}	46.9 ± 2.4^{l}	5530 ± 288^{op}	1754 ± 95^{mn}
SN-FL-042	Z5	17871 ± 928^{n}	6205 ± 319^{l}	4278 ± 232^{ij}	51.7 ± 2.8^{hi}	6566 ± 334^{ef}	1946 ± 103^{i}
SN-FL-043	Z5	17654 ± 887°	6141 ± 320^{lm}	4378 ± 228^{hi}	51.3 ± 2.8^{hi}	6461 ± 336^{fg}	2180 ± 120^{cd}
SN-FL-044	Z5	18161 ± 929^{l}	7153 ± 376^{fg}	4473 ± 243^{gh}	49.9 ± 2.7^{j}	5953 ± 305^{kl}	1941 ± 105^{i}
SN-FL-045	Z5	17494 ± 924^{p}	6574 ± 338^{j}	3867 ± 206^{mn}	50.3 ± 2.7^{ij}	6746 ± 368^{d}	1582 ± 85^{qr}
SN-FL-046	Z5	17992 ± 932^{m}	5356 ± 273^{qr}	3537 ± 176^{pq}	47.6 ± 2.4^{kl}	5800 ± 300^{m}	1602 ± 88^{q}
SN-FL-047	Z5	17626 ± 917^{op}	6802 ± 361^{hi}	3499 ± 181^{q}	48.4 ± 2.5^{k}	6263 ± 333^{hi}	1834 ± 103^{kl}
SN-FL-066	Z5	19099 ± 985^{fg}	6416 ± 346^{k}	3200 ± 168st	43.1 ± 2.3^{op}	5896 ± 313^{l}	1802 ± 97^{lm}
SN-FL-067	Z5	17394 ± 882^{q}	6469 ± 338^{jk}	3982 ± 204^{l}	47.5 ± 2.6^{l}	5724 ± 305^{n}	1635 ± 90^{pq}
SN-FL-068	Z5	17407 ± 878^{pq}	6047 ± 310^{m}	4230 ± 213^{j}	45.6 ± 2.3^{mn}	5627 ± 294°	1773 ± 96^{m}
SN-FL-073	Z5	19096 ± 980^{fg}	6591 ± 333^{j}	3404 ± 177^{qr}	46.7 ± 2.4^{lm}	6618 ± 349^{e}	1556 ± 86^{r}
SN-FL-074	Z5	17831 ± 911^{n}	6857 ± 355^{h}	3249 ± 162^{s}	44.4 ± 2.3^{no}	5979 ± 318^{kl}	1519 ± 81^{s}

SN-FL-075	Z5	17553 ± 931^{p}	5588 ± 285^{p}	4303 ± 225^{i}	46.3 ± 2.5^{m}	6286 ± 339^{hi}	1859 ± 102^{k}
SN-FL-076	Z5	18534 ± 941^{j}	5000 ± 254st	4022 ± 213^{l}	43.9 ± 2.3^{no}	5834 ± 306^{m}	1682 ± 89°
SN-FL-077	Z5	17844 ± 908^{n}	5775 ± 287°	3851 ± 207^{mn}	42.4 ± 2.2^{p}	6005 ± 328^{k}	1701 ± 92°
SN-FL-078	Z5	18756 ± 993^{hi}	5691 ± 285°	4254 ± 216^{j}	44.2 ± 2.3^{no}	5598 ± 299°	1717 ± 94no
SN-FL-079	Z5	17964 ± 922^{mn}	6163 ± 327^{lm}	3763 ± 195no	42.8 ± 2.3^{op}	6114 ± 316^{j}	1893 ± 101^{j}
SN-FL-080	Z5	17764 ± 911no	5911 ± 299^{n}	3912 ± 212^{m}	45.0 ± 2.3^{n}	6054 ± 322^{jk}	1671 ± 88^{op}
SN-FL-092	Z5	17467 ± 890^{pq}	5525 ± 293^{pq}	4218 ± 221^{j}	49.2 ± 2.7^{jk}	6042 ± 328^{k}	1725 ± 92^{n}
SN-FL-093	Z5	18566 ± 985^{j}	6286 ± 328^{kl}	3969 ± 209^{lm}	41.0 ± 2.2^{q}	5702 ± 296^{n}	1818 ± 97^{l}

Notes: Values are reported as mean ± analytical SD of three independently prepared analytical portions on a dry-weight basis (mg/kg DW). The three analytical measurements were used only to assess analytical precision and were averaged to obtain one site-level concentration per matrix and georeferenced sampling site for subsequent statistical analyses. Values <LOD and <LOQ are indicated as applicable.

Table S24. Individual concentrations of environmentally associated microelements and biologically relevant trace elements measured in georeferenced flower samples of *Sambucus nigra* L. collected across the investigated ecological sectors of the Oradea metropolitan area, Romania.

Sample ID	Zone	^{56}Fe (mg/kg)	^{55}Mn (mg/kg)	^{64}Zn (mg/kg)	^{63}Cu (mg/kg)	^{11}B (mg/kg)	^{98}Mo (mg/kg)	^{59}Co (mg/kg)	^{80}Se (mg/kg)
SN-FL-022	Z1	106 ± 6^{de}	56.0 ± 4.1^{de}	36.7 ± 2.7^{cd}	11.15 ± 0.59^{de}	30.7 ± 1.9^{cd}	0.38 ± 0.04^{e}	0.125 ± 0.016^{de}	0.081 ± 0.008^{cd}
SN-FL-023	Z1	126 ± 10^{bc}	68.4 ± 5.0^{bc}	38.0 ± 2.4^{cd}	11.87 ± 0.65^{cd}	32.9 ± 2.0^{bc}	0.40 ± 0.05^{de}	0.096 ± 0.011^{ef}	0.107 ± 0.012^{bc}
SN-FL-026	Z1	97 ± 7^{ef}	52.9 ± 3.5^{de}	31.5 ± 1.6^{de}	12.00 ± 0.61^{cd}	33.0 ± 1.4^{cd}	0.33 ± 0.04^{ef}	0.131 ± 0.017^{de}	0.053 ± 0.006^{ef}
SN-FL-027	Z1	108 ± 9^{de}	50.7 ± 3.2^{e}	33.6 ± 2.5^{de}	11.60 ± 0.80^{cd}	33.6 ± 2.1^{bc}	0.69 ± 0.07^{bc}	0.108 ± 0.014^{de}	0.056 ± 0.006^{e}
SN-FL-029	Z1	126 ± 9^{bc}	50.7 ± 3.0^{e}	38.3 ± 2.7^{cd}	11.18 ± 0.73^{de}	30.7 ± 1.7^{cd}	0.54 ± 0.05^{cd}	0.132 ± 0.014^{de}	0.119 ± 0.013^{ab}
SN-FL-053	Z1	106 ± 8^{de}	53.5 ± 3.0^{de}	31.1 ± 1.9^{de}	10.59 ± 0.59^{de}	28.4 ± 1.5^{de}	0.47 ± 0.05^{de}	0.131 ± 0.018^{de}	0.101 ± 0.011^{bc}
SN-FL-061	Z1	124 ± 10^{bc}	57.3 ± 3.5^{de}	41.4 ± 2.6^{bc}	10.61 ± 0.70^{de}	35.1 ± 1.8^{bc}	0.50 ± 0.05^{cd}	0.112 ± 0.011^{de}	0.086 ± 0.011^{cd}
SN-FL-062	Z1	109 ± 8^{de}	56.9 ± 3.9^{de}	41.5 ± 3.0^{bc}	10.22 ± 0.54^{de}	31.3 ± 1.5^{cd}	0.61 ± 0.07^{cd}	0.095 ± 0.011^{ef}	0.083 ± 0.011^{cd}
SN-FL-063	Z1	120 ± 10^{cd}	44.4 ± 2.5^{f}	37.0 ± 1.9^{cd}	11.48 ± 0.78^{cd}	35.2 ± 1.3^{bc}	0.29 ± 0.03^{f}	0.139 ± 0.019^{d}	0.076 ± 0.009^{cd}
SN-FL-081	Z1	109 ± 8^{de}	60.0 ± 5.0^{cd}	31.2 ± 2.1^{de}	11.47 ± 0.66^{d}	32.1 ± 1.7^{cd}	0.57 ± 0.07^{cd}	0.196 ± 0.023^{bc}	0.081 ± 0.011^{cd}
SN-FL-082	Z1	117 ± 7^{cd}	63.0 ± 4.6^{cd}	40.4 ± 2.6^{bc}	13.70 ± 0.80^{bc}	32.8 ± 2.1^{bc}	0.48 ± 0.04^{d}	0.153 ± 0.015^{cd}	0.122 ± 0.013^{ab}
SN-FL-083	Z1	119 ± 7^{cd}	55.1 ± 4.6^{de}	32.1 ± 1.7^{de}	11.16 ± 0.67^{de}	30.4 ± 1.9^{cd}	0.34 ± 0.04^{ef}	0.128 ± 0.013^{de}	0.094 ± 0.011^{bc}
SN-FL-084	Z1	117 ± 7^{cd}	65.8 ± 4.1^{cd}	24.6 ± 1.7^{f}	11.35 ± 0.55^{d}	28.8 ± 1.8^{de}	0.52 ± 0.05^{cd}	0.182 ± 0.019^{bc}	0.097 ± 0.009^{bc}

SN-FL-003	Z2	119 ± 9^{cd}	79.7 ± 5.6^{ab}	40.4 ± 1.9^{bc}	14.70 ± 1.08^{ab}	28.9 ± 1.4^{de}	0.56 ± 0.05^{cd}	0.291 ± 0.031^{a}	0.084 ± 0.009^{cd}
SN-FL-004	Z2	145 ± 8^{ab}	70.6 ± 5.0^{bc}	38.4 ± 2.4^{cd}	13.85 ± 0.86^{bc}	36.2 ± 1.9^{ab}	0.85 ± 0.10^{ab}	0.207 ± 0.026^{bc}	0.121 ± 0.014^{ab}
SN-FL-014	Z2	125 ± 10^{bc}	77.9 ± 5.5^{ab}	36.3 ± 2.2^{cd}	13.83 ± 0.65^{bc}	32.6 ± 1.3^{cd}	0.89 ± 0.10^{ab}	0.195 ± 0.023^{bc}	0.110 ± 0.015^{ab}
SN-FL-018	Z2	155 ± 10^{a}	65.0 ± 4.9^{cd}	40.6 ± 2.4^{bc}	16.01 ± 1.05^{ab}	35.3 ± 2.2^{ab}	0.55 ± 0.06^{cd}	0.178 ± 0.018^{c}	0.093 ± 0.012^{bc}
SN-FL-019	Z2	123 ± 10^{bc}	55.9 ± 4.2^{de}	46.2 ± 2.4^{ab}	14.47 ± 0.88^{bc}	34.0 ± 1.3^{bc}	0.72 ± 0.07^{bc}	0.223 ± 0.027^{ab}	0.121 ± 0.011^{ab}
SN-FL-020	Z2	129 ± 10^{bc}	68.8 ± 3.9^{bc}	44.3 ± 3.3^{ab}	13.66 ± 0.77^{bc}	30.9 ± 1.3^{cd}	0.65 ± 0.06^{bc}	0.205 ± 0.028^{bc}	0.060 ± 0.008^{de}
SN-FL-048	Z2	128 ± 10^{bc}	71.6 ± 4.0^{bc}	45.3 ± 2.5^{ab}	16.79 ± 1.24^{a}	32.0 ± 1.7^{cd}	0.46 ± 0.06^{de}	0.209 ± 0.021^{bc}	0.078 ± 0.009^{cd}
SN-FL-049	Z2	150 ± 10^{ab}	48.0 ± 3.8^{ef}	42.9 ± 3.1^{bc}	10.92 ± 0.52^{de}	39.3 ± 1.8^{a}	0.70 ± 0.06^{bc}	0.141 ± 0.019^{d}	0.095 ± 0.010^{bc}
SN-FL-050	Z2	141 ± 11^{ab}	77.8 ± 5.3^{ab}	39.9 ± 2.1^{bc}	13.95 ± 0.79^{bc}	28.9 ± 1.5^{de}	0.55 ± 0.07^{cd}	0.203 ± 0.021^{bc}	0.105 ± 0.011^{bc}
SN-FL-051	Z2	121 ± 8^{cd}	65.0 ± 3.7^{cd}	46.9 ± 2.8^{ab}	13.39 ± 0.70^{bc}	32.9 ± 1.5^{cd}	0.70 ± 0.07^{bc}	0.229 ± 0.031^{ab}	0.114 ± 0.011^{ab}
SN-FL-052	Z2	148 ± 11^{ab}	67.4 ± 4.3^{bc}	35.3 ± 2.0^{cd}	12.18 ± 0.64^{cd}	33.2 ± 1.3^{bc}	0.53 ± 0.05^{cd}	0.162 ± 0.020^{cd}	0.084 ± 0.012^{cd}
SN-FL-054	Z2	132 ± 11^{bc}	66.3 ± 5.1^{bc}	48.9 ± 2.2^{ab}	14.28 ± 1.06^{bc}	35.5 ± 1.6^{ab}	0.56 ± 0.05^{cd}	0.241 ± 0.026^{ab}	0.076 ± 0.010^{cd}
SN-FL-055	Z2	139 ± 11^{ab}	65.4 ± 4.0^{cd}	34.9 ± 2.3^{cd}	13.35 ± 0.78^{bc}	32.8 ± 1.6^{cd}	0.60 ± 0.08^{cd}	0.200 ± 0.024^{bc}	0.077 ± 0.009^{cd}
SN-FL-056	Z2	121 ± 7^{cd}	69.1 ± 4.1^{bc}	42.4 ± 1.9^{bc}	13.39 ± 0.99^{bc}	26.8 ± 1.3^{ef}	0.75 ± 0.09^{ab}	0.220 ± 0.020^{b}	0.097 ± 0.011^{bc}
SN-FL-057	Z2	134 ± 11^{ab}	80.1 ± 5.4^{ab}	41.4 ± 2.6^{bc}	13.39 ± 0.75^{bc}	33.4 ± 1.9^{bc}	0.92 ± 0.08^{a}	0.130 ± 0.017^{de}	0.095 ± 0.011^{bc}
SN-FL-059	Z2	137 ± 10^{ab}	60.3 ± 4.9^{cd}	47.1 ± 2.7^{ab}	11.77 ± 0.63^{cd}	29.8 ± 1.5^{de}	0.75 ± 0.10^{ab}	0.197 ± 0.023^{bc}	0.134 ± 0.018^{ab}
SN-FL-064	Z2	133 ± 10^{bc}	65.5 ± 5.4^{bc}	44.4 ± 3.1^{ab}	15.22 ± 0.93^{ab}	32.4 ± 1.9^{cd}	0.66 ± 0.08^{bc}	0.174 ± 0.018^{cd}	0.122 ± 0.012^{ab}
SN-FL-065	Z2	145 ± 11^{ab}	67.5 ± 5.7^{bc}	40.5 ± 2.9^{bc}	14.82 ± 0.76^{ab}	35.4 ± 1.5^{bc}	0.40 ± 0.05^{de}	0.170 ± 0.023^{cd}	0.106 ± 0.014^{ab}
SN-FL-069	Z2	139 ± 8^{ab}	73.0 ± 4.9^{bc}	41.6 ± 2.6^{bc}	14.54 ± 0.98^{ab}	31.0 ± 1.7^{cd}	0.72 ± 0.08^{bc}	0.175 ± 0.021^{cd}	0.104 ± 0.011^{bc}
SN-FL-085	Z2	139 ± 8^{ab}	62.3 ± 4.6^{cd}	44.2 ± 2.3^{bc}	12.44 ± 0.73^{cd}	34.3 ± 2.1^{bc}	0.75 ± 0.09^{ab}	0.239 ± 0.033^{ab}	0.080 ± 0.008^{cd}
SN-FL-086	Z2	138 ± 10^{ab}	68.7 ± 4.5^{bc}	48.2 ± 3.2^{ab}	14.41 ± 0.83^{bc}	32.3 ± 1.5^{cd}	0.68 ± 0.07^{bc}	0.169 ± 0.022^{cd}	0.075 ± 0.010^{cd}
SN-FL-087	Z2	147 ± 11^{ab}	75.7 ± 5.8^{ab}	47.6 ± 2.5^{ab}	12.78 ± 0.64^{cd}	32.6 ± 1.7^{cd}	0.54 ± 0.07^{cd}	0.181 ± 0.020^{bc}	0.079 ± 0.010^{cd}
SN-FL-088	Z2	135 ± 7^{bc}	73.4 ± 4.5^{bc}	42.6 ± 2.5^{bc}	13.89 ± 0.67^{bc}	35.5 ± 2.3^{ab}	0.49 ± 0.05^{d}	0.265 ± 0.024^{ab}	0.118 ± 0.013^{ab}
SN-FL-094	Z2	121 ± 8^{cd}	64.3 ± 5.2^{cd}	36.3 ± 1.8^{cd}	14.41 ± 1.02^{bc}	32.7 ± 1.8^{bc}	0.92 ± 0.08^{a}	0.181 ± 0.019^{c}	0.063 ± 0.009^{de}
SN-FL-001	Z3	129 ± 10^{bc}	71.5 ± 6.1^{bc}	35.3 ± 1.9^{cd}	13.40 ± 0.66^{bc}	28.5 ± 1.5^{de}	0.47 ± 0.05^{de}	0.184 ± 0.017^{bc}	0.054 ± 0.007^{ef}
SN-FL-005	Z3	128 ± 9^{bc}	61.6 ± 4.0^{cd}	38.3 ± 2.3^{cd}	11.29 ± 0.82^{de}	28.3 ± 1.4^{de}	0.46 ± 0.04^{de}	0.133 ± 0.014^{de}	0.065 ± 0.008^{de}
SN-FL-006	Z3	123 ± 9^{bc}	49.7 ± 3.3^{ef}	41.3 ± 3.0^{bc}	12.14 ± 0.55^{cd}	31.1 ± 2.0^{cd}	0.52 ± 0.05^{cd}	0.179 ± 0.020^{cd}	0.086 ± 0.010^{cd}
SN-FL-007	Z3	125 ± 7^{bc}	55.1 ± 3.9^{de}	36.0 ± 2.0^{cd}	11.88 ± 0.54^{cd}	29.7 ± 1.9^{de}	0.36 ± 0.03^{e}	0.166 ± 0.021^{cd}	0.061 ± 0.007^{de}

SN-FL-008	Z3	133 ± 8^{bc}	57.5 ± 3.2^{d}	40.4 ± 3.0^{bc}	12.53 ± 0.62^{cd}	28.1 ± 1.1^{ef}	0.55 ± 0.05^{cd}	0.149 ± 0.018^{cd}	0.081 ± 0.008^{cd}
SN-FL-032	Z3	130 ± 9^{bc}	58.7 ± 4.4^{cd}	38.1 ± 1.9^{cd}	11.67 ± 0.55^{cd}	28.9 ± 1.7^{de}	0.23 ± 0.02^{f}	0.170 ± 0.020^{cd}	0.053 ± 0.006^{ef}
SN-FL-033	Z3	128 ± 7^{bc}	65.4 ± 4.3^{cd}	40.1 ± 2.8^{bc}	11.24 ± 0.73^{de}	27.7 ± 1.5^{ef}	0.25 ± 0.03^{f}	0.141 ± 0.019^{d}	0.035 ± 0.003^{f}
SN-FL-034	Z3	122 ± 9^{cd}	72.0 ± 4.9^{bc}	40.4 ± 1.9^{bc}	10.92 ± 0.67^{de}	31.8 ± 1.3^{cd}	0.70 ± 0.06^{bc}	0.165 ± 0.017^{cd}	0.060 ± 0.008^{de}
SN-FL-035	Z3	127 ± 10^{bc}	63.4 ± 4.9^{cd}	41.8 ± 2.8^{bc}	10.82 ± 0.78^{de}	36.8 ± 2.3^{ab}	0.35 ± 0.04^{ef}	0.168 ± 0.019^{cd}	0.069 ± 0.008^{de}
SN-FL-036	Z3	117 ± 7^{cd}	56.2 ± 3.3^{de}	37.8 ± 1.9^{cd}	11.68 ± 0.87^{cd}	28.6 ± 1.8^{de}	0.37 ± 0.03^{e}	0.144 ± 0.016^{d}	0.093 ± 0.009^{bc}
SN-FL-037	Z3	104 ± 8^{de}	64.1 ± 3.9^{cd}	47.4 ± 2.9^{ab}	11.96 ± 0.60^{cd}	26.1 ± 1.5^{ef}	0.49 ± 0.04^{d}	0.144 ± 0.015^{d}	0.088 ± 0.012^{cd}
SN-FL-038	Z3	124 ± 10^{bc}	69.2 ± 4.1^{bc}	39.5 ± 2.8^{bc}	10.93 ± 0.50^{de}	31.8 ± 1.4^{cd}	0.63 ± 0.07^{bc}	0.130 ± 0.016^{de}	0.067 ± 0.008^{de}
SN-FL-039	Z3	130 ± 9^{bc}	70.8 ± 5.5^{bc}	40.7 ± 2.6^{bc}	11.64 ± 0.74^{cd}	29.6 ± 1.5^{de}	0.48 ± 0.06^{de}	0.167 ± 0.023^{cd}	0.059 ± 0.006^{de}
SN-FL-040	Z3	119 ± 9^{cd}	68.7 ± 5.7^{bc}	29.9 ± 1.9^{e}	9.42 ± 0.46^{ef}	29.7 ± 1.2^{de}	0.53 ± 0.04^{cd}	0.132 ± 0.016^{de}	0.085 ± 0.011^{cd}
SN-FL-041	Z3	123 ± 10^{bc}	56.9 ± 3.2^{de}	41.6 ± 2.9^{bc}	10.42 ± 0.71^{de}	29.7 ± 1.6^{de}	0.39 ± 0.03^{e}	0.145 ± 0.015^{d}	0.086 ± 0.008^{cd}
SN-FL-058	Z3	112 ± 9^{cd}	51.2 ± 4.3^{de}	40.9 ± 2.8^{bc}	10.44 ± 0.48^{de}	31.3 ± 1.1^{cd}	0.39 ± 0.03^{e}	0.084 ± 0.009^{ef}	0.111 ± 0.012^{ab}
SN-FL-060	Z3	125 ± 11^{bc}	42.8 ± 3.0^{f}	40.3 ± 2.2^{bc}	12.11 ± 0.75^{cd}	35.4 ± 2.2^{ab}	0.67 ± 0.06^{bc}	0.221 ± 0.029^{ab}	0.064 ± 0.007^{de}
SN-FL-089	Z3	136 ± 11^{ab}	62.6 ± 4.7^{cd}	37.2 ± 2.4^{cd}	13.45 ± 0.89^{bc}	33.6 ± 2.2^{bc}	0.57 ± 0.05^{cd}	0.121 ± 0.014^{de}	0.068 ± 0.007^{de}
SN-FL-090	Z3	132 ± 9^{bc}	62.7 ± 4.7^{cd}	47.4 ± 2.7^{ab}	11.13 ± 0.64^{de}	33.9 ± 1.2^{bc}	0.43 ± 0.05^{de}	0.154 ± 0.015^{cd}	0.074 ± 0.009^{cd}
SN-FL-091	Z3	112 ± 8^{cd}	51.5 ± 3.0^{de}	32.9 ± 2.4^{de}	11.36 ± 0.64^{d}	31.6 ± 1.2^{cd}	0.58 ± 0.05^{cd}	0.191 ± 0.020^{bc}	0.065 ± 0.008^{de}
SN-FL-002	Z4	102 ± 6^{de}	71.7 ± 4.4^{bc}	32.3 ± 2.2^{de}	9.72 ± 0.68^{ef}	29.9 ± 1.7^{de}	0.36 ± 0.04^{e}	0.086 ± 0.011^{ef}	0.054 ± 0.005^{ef}
SN-FL-009	Z4	87 ± 7^{f}	57.2 ± 4.5^{de}	31.5 ± 1.9^{de}	12.10 ± 0.85^{cd}	34.0 ± 2.2^{bc}	0.50 ± 0.04^{d}	0.069 ± 0.008^{f}	0.046 ± 0.004^{ef}
SN-FL-010	Z4	93 ± 5^{ef}	72.6 ± 6.0^{bc}	35.9 ± 2.4^{cd}	10.34 ± 0.70^{de}	35.2 ± 1.5^{bc}	0.40 ± 0.03^{de}	0.099 ± 0.014^{ef}	0.050 ± 0.005^{ef}
SN-FL-011	Z4	96 ± 7^{ef}	53.4 ± 3.5^{de}	38.1 ± 2.1^{cd}	9.30 ± 0.60^{ef}	33.2 ± 1.7^{bc}	0.51 ± 0.05^{cd}	0.122 ± 0.013^{de}	0.059 ± 0.008^{de}
SN-FL-012	Z4	98 ± 8^{ef}	73.9 ± 5.6^{bc}	31.3 ± 1.8^{de}	10.43 ± 0.61^{de}	35.6 ± 2.3^{ab}	0.57 ± 0.05^{cd}	0.125 ± 0.016^{de}	0.075 ± 0.009^{cd}
SN-FL-013	Z4	88 ± 6^{f}	62.2 ± 5.3^{cd}	34.4 ± 1.7^{d}	10.11 ± 0.72^{de}	30.5 ± 1.3^{de}	0.53 ± 0.04^{cd}	0.144 ± 0.020^{cd}	0.053 ± 0.007^{ef}
SN-FL-015	Z4	107 ± 9^{de}	54.6 ± 3.2^{de}	29.7 ± 1.8^{e}	8.41 ± 0.51^{f}	33.1 ± 2.0^{bc}	0.47 ± 0.04^{d}	0.129 ± 0.015^{de}	0.051 ± 0.006^{ef}
SN-FL-016	Z4	104 ± 7^{de}	65.2 ± 3.6^{cd}	38.0 ± 2.5^{cd}	10.26 ± 0.72^{de}	33.3 ± 1.8^{bc}	0.50 ± 0.05^{cd}	0.082 ± 0.007^{f}	0.075 ± 0.008^{cd}
SN-FL-017	Z4	96 ± 8^{ef}	54.8 ± 3.5^{de}	35.2 ± 1.8^{cd}	8.94 ± 0.45^{ef}	33.7 ± 1.8^{bc}	0.29 ± 0.03^{f}	0.120 ± 0.014^{de}	0.056 ± 0.005^{e}
SN-FL-021	Z4	87 ± 6^{f}	61.7 ± 4.9^{cd}	32.0 ± 2.0^{de}	8.88 ± 0.54^{ef}	32.5 ± 1.4^{cd}	0.39 ± 0.05^{de}	0.127 ± 0.016^{de}	0.065 ± 0.006^{de}
SN-FL-024	Z4	104 ± 6^{de}	62.4 ± 3.4^{cd}	34.1 ± 2.0^{d}	8.61 ± 0.54^{f}	31.8 ± 2.0^{cd}	0.30 ± 0.03^{ef}	0.099 ± 0.010^{ef}	0.071 ± 0.007^{de}
SN-FL-025	Z4	96 ± 5^{ef}	66.8 ± 4.8^{bc}	36.3 ± 2.5^{cd}	10.08 ± 0.55^{de}	32.3 ± 1.4^{cd}	0.45 ± 0.05^{de}	0.122 ± 0.017^{de}	0.100 ± 0.014^{bc}

SN-FL-028	Z4	105 ± 8^{de}	48.4 ± 3.6^{ef}	32.3 ± 2.0^{de}	9.51 ± 0.56^{ef}	32.8 ± 1.7^{bc}	0.37 ± 0.03^{e}	0.075 ± 0.009^{f}	0.088 ± 0.011^{cd}
SN-FL-030	Z4	119 ± 7^{cd}	49.8 ± 3.8^{ef}	35.7 ± 2.6^{cd}	8.25 ± 0.52^{f}	30.7 ± 1.1^{d}	0.39 ± 0.03^{e}	0.147 ± 0.017^{cd}	0.070 ± 0.008^{de}
SN-FL-031	Z4	105 ± 7^{de}	66.3 ± 4.7^{bc}	32.4 ± 2.0^{de}	9.73 ± 0.69^{ef}	32.8 ± 1.7^{bc}	0.38 ± 0.03^{e}	0.071 ± 0.010^{f}	0.061 ± 0.008^{de}
SN-FL-070	Z4	98 ± 7^{ef}	52.9 ± 4.5^{de}	31.3 ± 2.3^{de}	9.02 ± 0.57^{ef}	31.6 ± 1.4^{cd}	0.46 ± 0.06^{de}	0.095 ± 0.011^{ef}	0.065 ± 0.008^{de}
SN-FL-071	Z4	109 ± 7^{de}	58.1 ± 4.8^{cd}	32.7 ± 1.7^{de}	10.71 ± 0.68^{de}	33.7 ± 2.1^{bc}	0.53 ± 0.06^{cd}	0.167 ± 0.016^{cd}	0.075 ± 0.010^{cd}
SN-FL-072	Z4	107 ± 6^{de}	61.2 ± 4.1^{cd}	37.3 ± 1.7^{cd}	9.15 ± 0.51^{ef}	30.5 ± 1.3^{de}	0.40 ± 0.04^{de}	0.119 ± 0.011^{de}	0.074 ± 0.010^{cd}
SN-FL-042	Z5	122 ± 8^{cd}	57.0 ± 4.8^{de}	37.9 ± 2.3^{cd}	11.84 ± 0.65^{cd}	31.7 ± 1.3^{cd}	0.56 ± 0.05^{cd}	0.141 ± 0.016^{d}	0.117 ± 0.015^{ab}
SN-FL-043	Z5	122 ± 10^{bc}	59.0 ± 5.0^{cd}	45.9 ± 2.1^{ab}	12.25 ± 0.57^{cd}	30.4 ± 1.1^{de}	0.53 ± 0.07^{cd}	0.128 ± 0.016^{de}	0.081 ± 0.008^{cd}
SN-FL-044	Z5	118 ± 7^{cd}	47.9 ± 3.1^{ef}	42.0 ± 2.8^{bc}	13.23 ± 0.75^{bc}	33.0 ± 2.0^{bc}	0.60 ± 0.06^{cd}	0.156 ± 0.019^{cd}	0.095 ± 0.009^{bc}
SN-FL-045	Z5	125 ± 10^{bc}	50.3 ± 3.0^{e}	39.3 ± 2.7^{bc}	12.21 ± 0.90^{cd}	30.7 ± 1.1^{d}	0.58 ± 0.07^{cd}	0.184 ± 0.017^{bc}	0.081 ± 0.011^{cd}
SN-FL-046	Z5	117 ± 9^{cd}	62.5 ± 4.7^{cd}	35.6 ± 1.9^{cd}	12.39 ± 0.69^{cd}	34.2 ± 1.8^{bc}	0.62 ± 0.05^{c}	0.139 ± 0.013^{d}	0.111 ± 0.013^{ab}
SN-FL-047	Z5	128 ± 11^{bc}	64.0 ± 4.4^{cd}	37.1 ± 2.1^{cd}	12.15 ± 0.86^{cd}	33.9 ± 1.2^{bc}	0.45 ± 0.06^{de}	0.169 ± 0.017^{cd}	0.085 ± 0.009^{cd}
SN-FL-066	Z5	115 ± 7^{cd}	54.0 ± 4.0^{de}	35.7 ± 2.3^{cd}	13.85 ± 0.97^{bc}	32.6 ± 1.9^{bc}	0.44 ± 0.04^{de}	0.169 ± 0.017^{cd}	0.065 ± 0.008^{de}
SN-FL-067	Z5	137 ± 11^{ab}	56.7 ± 4.3^{de}	41.4 ± 2.9^{bc}	12.70 ± 0.74^{cd}	35.5 ± 2.2^{ab}	0.66 ± 0.09^{bc}	0.173 ± 0.018^{cd}	0.096 ± 0.011^{bc}
SN-FL-068	Z5	118 ± 10^{cd}	48.1 ± 3.5^{ef}	38.2 ± 1.9^{cd}	10.99 ± 0.53^{de}	30.2 ± 1.2^{de}	0.60 ± 0.07^{cd}	0.105 ± 0.014^{e}	0.068 ± 0.006^{de}
SN-FL-073	Z5	124 ± 9^{bc}	54.5 ± 4.0^{de}	50.5 ± 2.8^{a}	10.69 ± 0.52^{de}	31.5 ± 1.1^{cd}	0.64 ± 0.07^{bc}	0.102 ± 0.012^{e}	0.094 ± 0.010^{bc}
SN-FL-074	Z5	135 ± 9^{bc}	62.2 ± 4.8^{cd}	43.6 ± 2.4^{bc}	11.05 ± 0.61^{de}	29.4 ± 1.4^{de}	0.54 ± 0.05^{cd}	0.174 ± 0.023^{cd}	0.074 ± 0.009^{cd}
SN-FL-075	Z5	133 ± 11^{bc}	86.1 ± 5.7^{a}	42.3 ± 2.1^{bc}	12.59 ± 0.89^{cd}	35.9 ± 1.7^{ab}	0.59 ± 0.07^{cd}	0.119 ± 0.016^{de}	0.070 ± 0.007^{de}
SN-FL-076	Z5	122 ± 7^{cd}	64.0 ± 5.3^{cd}	41.8 ± 2.7^{bc}	11.02 ± 0.54^{de}	32.5 ± 2.1^{bc}	0.44 ± 0.05^{de}	0.169 ± 0.016^{cd}	0.081 ± 0.008^{cd}
SN-FL-077	Z5	119 ± 8^{cd}	60.5 ± 4.5^{cd}	36.3 ± 2.6^{cd}	12.06 ± 0.76^{cd}	28.2 ± 1.3^{de}	0.61 ± 0.07^{cd}	0.163 ± 0.021^{cd}	0.081 ± 0.010^{cd}
SN-FL-078	Z5	118 ± 9^{cd}	67.3 ± 3.9^{bc}	35.7 ± 1.7^{cd}	11.02 ± 0.58^{de}	30.8 ± 1.1^{d}	0.61 ± 0.05^{c}	0.146 ± 0.016^{cd}	0.057 ± 0.007^{e}
SN-FL-079	Z5	140 ± 8^{ab}	70.7 ± 6.0^{bc}	33.1 ± 2.1^{de}	11.77 ± 0.68^{cd}	26.6 ± 1.1^{ef}	0.43 ± 0.05^{de}	0.195 ± 0.021^{bc}	0.122 ± 0.012^{ab}
SN-FL-080	Z5	123 ± 7^{cd}	61.1 ± 4.5^{cd}	36.6 ± 2.7^{cd}	11.69 ± 0.59^{cd}	30.7 ± 1.4^{cd}	0.79 ± 0.09^{ab}	0.164 ± 0.016^{cd}	0.082 ± 0.008^{cd}
SN-FL-092	Z5	103 ± 6^{de}	59.9 ± 4.2^{cd}	34.9 ± 2.1^{cd}	11.36 ± 0.57^{d}	34.2 ± 1.3^{bc}	0.55 ± 0.06^{cd}	0.119 ± 0.012^{de}	0.062 ± 0.006^{de}
SN-FL-093	Z5	122 ± 8^{cd}	58.8 ± 4.6^{cd}	43.3 ± 2.4^{bc}	10.33 ± 0.48^{de}	31.9 ± 1.6^{cd}	0.54 ± 0.06^{cd}	0.213 ± 0.022^{bc}	0.073 ± 0.009^{cd}

Notes: Values are reported as mean ± analytical SD of three independently prepared analytical portions on a dry-weight basis (mg/kg DW). The three analytical measurements were used only to assess analytical precision and were averaged to obtain one site-level concentration per matrix and georeferenced sampling site for subsequent statistical analyses. Values <LOD and <LOQ are indicated as applicable.

Table S25. Individual concentrations of lithogenic and geochemically associated elements in *Sambucus nigra* L. flower samples collected at georeferenced sampling points across the ecological sectors of the Oradea metropolitan area, Romania.

Sample ID	Zone	^{27} Al (mg/kg)	^{48} Ti (mg/kg)	^{51} V (mg/kg)	^{137} Ba (mg/kg)	^{88} Sr (mg/kg)	^{7} Li (mg/kg)	^{85} Rb (mg/kg)	^{133} Cs (mg/kg)
SN-FL-022	Z1	31.5 ± 2.5[bc]	2.05 ± 0.12[bc]	0.67 ± 0.06[cd]	20.4 ± 1.1[cd]	13.0 ± 0.7[de]	0.48 ± 0.04[de]	2.36 ± 0.15[de]	0.055 ± 0.006[cd]
SN-FL-023	Z1	36.0 ± 2.9[ab]	2.43 ± 0.19[ab]	0.60 ± 0.04[de]	20.1 ± 1.4[cd]	15.2 ± 1.1[bc]	0.67 ± 0.05[ab]	2.61 ± 0.24[bc]	0.072 ± 0.007[ab]
SN-FL-026	Z1	31.0 ± 2.4[cd]	1.91 ± 0.12[cd]	0.50 ± 0.03[ef]	17.9 ± 1.3[de]	12.4 ± 0.9[de]	0.46 ± 0.05[de]	2.12 ± 0.20[ef]	0.042 ± 0.005[f]
SN-FL-027	Z1	31.1 ± 2.0[bc]	1.77 ± 0.13[cd]	0.60 ± 0.05[de]	15.4 ± 1.1[ef]	12.2 ± 0.9[ef]	0.46 ± 0.04[de]	1.93 ± 0.14[f]	0.041 ± 0.004[f]
SN-FL-029	Z1	33.9 ± 2.5[ab]	1.94 ± 0.13[cd]	0.75 ± 0.06[bc]	23.3 ± 1.5[ab]	12.7 ± 0.9[de]	0.47 ± 0.03[de]	2.50 ± 0.16[cd]	0.059 ± 0.006[cd]
SN-FL-053	Z1	26.5 ± 1.6[de]	1.49 ± 0.13[ef]	0.56 ± 0.04[e]	19.6 ± 1.4[cd]	13.9 ± 0.8[cd]	0.50 ± 0.05[de]	2.28 ± 0.15[de]	0.053 ± 0.004[de]
SN-FL-061	Z1	19.3 ± 1.1[f]	1.16 ± 0.08[f]	0.40 ± 0.03[f]	15.3 ± 0.9[ef]	11.8 ± 0.9[ef]	0.43 ± 0.04[ef]	1.66 ± 0.15[f]	0.040 ± 0.004[f]
SN-FL-062	Z1	19.0 ± 1.2[f]	1.15 ± 0.09[f]	0.41 ± 0.03[f]	15.3 ± 1.2[ef]	13.3 ± 1.0[de]	0.44 ± 0.03[ef]	1.99 ± 0.18[f]	0.042 ± 0.004[f]
SN-FL-063	Z1	19.5 ± 1.2[f]	1.45 ± 0.08[ef]	0.64 ± 0.06[cd]	16.0 ± 1.0[ef]	12.1 ± 1.0[ef]	0.49 ± 0.05[de]	2.14 ± 0.19[ef]	0.056 ± 0.006[cd]
SN-FL-081	Z1	24.9 ± 1.5[ef]	1.76 ± 0.15[cd]	0.52 ± 0.04[ef]	16.6 ± 1.1[ef]	14.0 ± 1.0[cd]	0.53 ± 0.05[cd]	1.99 ± 0.12[f]	0.052 ± 0.005[de]
SN-FL-082	Z1	31.9 ± 1.9[bc]	1.87 ± 0.15[cd]	0.46 ± 0.03[f]	18.7 ± 1.2[de]	11.3 ± 0.8[ef]	0.47 ± 0.04[de]	2.34 ± 0.15[de]	0.054 ± 0.006[cd]
SN-FL-083	Z1	34.9 ± 2.9[ab]	1.44 ± 0.10[ef]	0.64 ± 0.05[cd]	17.5 ± 1.4[de]	13.3 ± 1.0[de]	0.55 ± 0.05[cd]	2.54 ± 0.23[cd]	0.049 ± 0.005[de]
SN-FL-084	Z1	23.2 ± 1.8[ef]	1.89 ± 0.14[cd]	0.70 ± 0.06[cd]	17.7 ± 1.1[de]	13.5 ± 0.8[de]	0.46 ± 0.04[de]	2.69 ± 0.23[bc]	0.044 ± 0.005[ef]
SN-FL-003	Z2	37.1 ± 2.4[ab]	2.64 ± 0.21[a]	0.81 ± 0.06[ab]	28.5 ± 2.0[a]	18.0 ± 1.3[a]	0.57 ± 0.04[bc]	3.26 ± 0.24[ab]	0.072 ± 0.006[ab]
SN-FL-004	Z2	33.5 ± 2.0[ab]	2.22 ± 0.12[ab]	0.83 ± 0.07[ab]	27.9 ± 1.5[a]	15.4 ± 1.2[bc]	0.64 ± 0.06[ab]	3.21 ± 0.27[ab]	0.082 ± 0.009[ab]
SN-FL-014	Z2	34.1 ± 2.9[ab]	1.73 ± 0.10[de]	0.67 ± 0.05[cd]	23.6 ± 2.0[ab]	18.6 ± 1.2[a]	0.62 ± 0.05[bc]	3.70 ± 0.27[a]	0.068 ± 0.006[ab]
SN-FL-018	Z2	30.0 ± 1.7[cd]	2.03 ± 0.12[bc]	0.79 ± 0.05[ab]	25.6 ± 2.2[ab]	18.5 ± 1.2[a]	0.78 ± 0.07[a]	3.19 ± 0.24[ab]	0.065 ± 0.006[bc]
SN-FL-019	Z2	33.0 ± 2.5[ab]	2.01 ± 0.11[bc]	0.98 ± 0.08[a]	22.6 ± 1.4[bc]	15.3 ± 0.9[bc]	0.61 ± 0.06[bc]	3.01 ± 0.20[ab]	0.087 ± 0.010[a]
SN-FL-020	Z2	35.1 ± 2.1[ab]	2.68 ± 0.15[a]	0.75 ± 0.06[bc]	25.6 ± 1.7[ab]	15.8 ± 1.1[ab]	0.68 ± 0.07[ab]	3.07 ± 0.25[ab]	0.060 ± 0.005[cd]
SN-FL-048	Z2	35.9 ± 2.2[ab]	2.34 ± 0.15[ab]	0.90 ± 0.07[ab]	25.3 ± 1.6[ab]	15.4 ± 1.2[bc]	0.72 ± 0.07[ab]	2.48 ± 0.18[cd]	0.069 ± 0.006[ab]
SN-FL-049	Z2	38.4 ± 3.2[a]	2.71 ± 0.21[a]	0.94 ± 0.08[a]	23.3 ± 1.9[ab]	15.9 ± 1.2[ab]	0.73 ± 0.06[ab]	2.98 ± 0.22[ab]	0.072 ± 0.008[ab]
SN-FL-050	Z2	39.3 ± 2.3[a]	2.23 ± 0.14[ab]	0.86 ± 0.07[ab]	27.4 ± 2.0[a]	17.5 ± 1.5[ab]	0.57 ± 0.04[bc]	3.40 ± 0.32[ab]	0.083 ± 0.007[a]
SN-FL-051	Z2	37.8 ± 2.8[a]	2.06 ± 0.17[bc]	0.91 ± 0.06[a]	21.9 ± 1.6[bc]	19.5 ± 1.2[a]	0.69 ± 0.05[ab]	3.12 ± 0.27[ab]	0.067 ± 0.007[bc]
SN-FL-052	Z2	36.4 ± 2.5[ab]	2.81 ± 0.19[a]	0.77 ± 0.07[ab]	21.8 ± 1.3[bc]	16.6 ± 1.2[ab]	0.58 ± 0.04[bc]	2.76 ± 0.21[bc]	0.083 ± 0.009[a]
SN-FL-054	Z2	34.8 ± 1.9[ab]	2.27 ± 0.15[ab]	0.78 ± 0.06[ab]	23.8 ± 1.8[ab]	15.1 ± 1.1[bc]	0.73 ± 0.06[ab]	3.30 ± 0.24[ab]	0.078 ± 0.007[ab]

SN-FL-055	Z2	34.6 ± 2.8[ab]	1.96 ± 0.15[bc]	0.92 ± 0.07[a]	21.6 ± 1.8[bc]	15.9 ± 1.1[ab]	0.73 ± 0.06[ab]	3.24 ± 0.20[ab]	0.065 ± 0.007[bc]
SN-FL-056	Z2	29.9 ± 1.9[cd]	2.31 ± 0.17[ab]	0.94 ± 0.07[a]	24.1 ± 1.6[ab]	17.3 ± 1.1[ab]	0.55 ± 0.04[cd]	3.10 ± 0.26[ab]	0.057 ± 0.007[cd]
SN-FL-057	Z2	36.9 ± 2.2[ab]	2.66 ± 0.16[a]	0.93 ± 0.08[a]	23.8 ± 1.4[ab]	15.0 ± 1.0[bc]	0.78 ± 0.07[a]	3.58 ± 0.33[a]	0.072 ± 0.006[ab]
SN-FL-059	Z2	33.1 ± 2.1[ab]	2.13 ± 0.14[bc]	0.85 ± 0.06[ab]	26.5 ± 2.1[a]	16.9 ± 1.2[ab]	0.71 ± 0.07[ab]	3.07 ± 0.21[ab]	0.070 ± 0.008[ab]
SN-FL-064	Z2	33.9 ± 2.6[ab]	1.96 ± 0.15[bc]	0.93 ± 0.07[a]	24.7 ± 1.4[ab]	16.4 ± 1.3[ab]	0.74 ± 0.07[ab]	2.61 ± 0.21[bc]	0.072 ± 0.008[ab]
SN-FL-065	Z2	32.2 ± 2.0[bc]	2.51 ± 0.14[a]	0.86 ± 0.06[ab]	24.8 ± 1.8[ab]	15.5 ± 1.3[bc]	0.69 ± 0.07[ab]	2.72 ± 0.17[bc]	0.075 ± 0.008[ab]
SN-FL-069	Z2	37.4 ± 3.0[ab]	2.17 ± 0.18[ab]	0.93 ± 0.08[a]	24.9 ± 2.0[ab]	16.1 ± 1.0[ab]	0.70 ± 0.06[ab]	3.00 ± 0.25[ab]	0.079 ± 0.009[ab]
SN-FL-085	Z2	31.5 ± 2.0[bc]	2.62 ± 0.17[a]	0.89 ± 0.08[ab]	24.8 ± 1.5[ab]	19.7 ± 1.2[a]	0.74 ± 0.07[ab]	2.99 ± 0.20[ab]	0.070 ± 0.008[ab]
SN-FL-086	Z2	37.4 ± 2.4[a]	1.98 ± 0.15[bc]	0.73 ± 0.05[bc]	23.3 ± 1.9[ab]	16.5 ± 0.9[ab]	0.76 ± 0.07[a]	2.88 ± 0.25[ab]	0.081 ± 0.009[ab]
SN-FL-087	Z2	33.9 ± 2.4[ab]	2.50 ± 0.15[a]	0.79 ± 0.06[ab]	27.9 ± 1.9[a]	18.8 ± 1.3[a]	0.80 ± 0.06[a]	3.44 ± 0.30[a]	0.080 ± 0.007[ab]
SN-FL-088	Z2	30.4 ± 2.1[cd]	2.70 ± 0.22[a]	0.96 ± 0.06[a]	27.9 ± 2.3[a]	18.0 ± 1.5[ab]	0.72 ± 0.05[ab]	3.25 ± 0.31[ab]	0.081 ± 0.007[a]
SN-FL-094	Z2	34.2 ± 2.3[ab]	2.20 ± 0.16[ab]	0.69 ± 0.04[cd]	22.7 ± 1.8[ab]	17.4 ± 1.1[ab]	0.68 ± 0.05[ab]	3.22 ± 0.29[ab]	0.071 ± 0.006[ab]
SN-FL-001	Z3	39.1 ± 3.1[a]	2.02 ± 0.17[bc]	0.61 ± 0.04[de]	19.9 ± 1.5[cd]	15.4 ± 0.9[bc]	0.67 ± 0.05[ab]	2.92 ± 0.26[ab]	0.059 ± 0.006[cd]
SN-FL-005	Z3	36.0 ± 2.9[ab]	2.39 ± 0.18[ab]	0.77 ± 0.07[ab]	21.6 ± 1.6[bc]	13.3 ± 0.9[de]	0.69 ± 0.07[ab]	2.98 ± 0.24[ab]	0.070 ± 0.008[ab]
SN-FL-006	Z3	37.1 ± 2.1[a]	2.36 ± 0.15[ab]	0.75 ± 0.05[bc]	21.3 ± 1.4[bc]	16.1 ± 1.1[ab]	0.59 ± 0.06[bc]	3.19 ± 0.27[ab]	0.059 ± 0.007[cd]
SN-FL-007	Z3	27.5 ± 1.6[de]	1.73 ± 0.14[de]	0.65 ± 0.05[cd]	18.7 ± 1.4[de]	16.0 ± 1.1[ab]	0.45 ± 0.05[ef]	2.33 ± 0.14[de]	0.077 ± 0.009[ab]
SN-FL-008	Z3	33.1 ± 2.7[ab]	1.84 ± 0.11[cd]	0.79 ± 0.06[ab]	22.5 ± 1.9[bc]	16.6 ± 1.1[ab]	0.67 ± 0.05[ab]	2.58 ± 0.16[cd]	0.087 ± 0.008[a]
SN-FL-032	Z3	31.4 ± 2.6[bc]	1.85 ± 0.12[cd]	0.75 ± 0.05[bc]	22.9 ± 1.3[ab]	15.8 ± 0.9[ab]	0.59 ± 0.04[bc]	2.98 ± 0.27[ab]	0.060 ± 0.005[cd]
SN-FL-033	Z3	32.7 ± 2.5[bc]	2.13 ± 0.16[bc]	0.71 ± 0.06[bc]	24.8 ± 2.0[ab]	13.7 ± 0.8[de]	0.59 ± 0.05[bc]	2.65 ± 0.22[bc]	0.074 ± 0.008[ab]
SN-FL-034	Z3	38.2 ± 2.4[a]	2.05 ± 0.16[bc]	0.68 ± 0.06[cd]	20.3 ± 1.4[cd]	16.2 ± 1.0[ab]	0.52 ± 0.04[cd]	2.94 ± 0.19[ab]	0.080 ± 0.008[ab]
SN-FL-035	Z3	29.1 ± 1.9[de]	1.94 ± 0.13[cd]	0.80 ± 0.07[ab]	19.9 ± 1.6[cd]	16.7 ± 1.4[ab]	0.69 ± 0.06[ab]	3.12 ± 0.21[ab]	0.062 ± 0.006[bc]
SN-FL-036	Z3	31.9 ± 2.3[bc]	2.04 ± 0.17[bc]	0.88 ± 0.06[ab]	22.9 ± 1.6[ab]	16.2 ± 1.4[ab]	0.56 ± 0.05[bc]	2.59 ± 0.16[cd]	0.070 ± 0.008[ab]
SN-FL-037	Z3	32.8 ± 2.0[bc]	2.18 ± 0.15[ab]	0.65 ± 0.05[cd]	22.8 ± 1.9[ab]	16.9 ± 1.1[ab]	0.58 ± 0.04[bc]	3.16 ± 0.24[ab]	0.069 ± 0.008[ab]
SN-FL-038	Z3	34.2 ± 2.5[ab]	1.64 ± 0.11[de]	0.61 ± 0.04[de]	19.8 ± 1.5[cd]	17.3 ± 1.4[ab]	0.53 ± 0.04[cd]	2.40 ± 0.15[cd]	0.060 ± 0.006[cd]
SN-FL-039	Z3	27.3 ± 2.2[de]	2.37 ± 0.16[ab]	0.67 ± 0.04[cd]	20.7 ± 1.6[cd]	14.7 ± 0.9[cd]	0.63 ± 0.06[ab]	2.51 ± 0.22[cd]	0.064 ± 0.006[bc]
SN-FL-040	Z3	31.2 ± 2.0[bc]	2.14 ± 0.15[bc]	0.74 ± 0.06[bc]	20.6 ± 1.2[cd]	13.9 ± 0.9[cd]	0.51 ± 0.04[de]	3.08 ± 0.20[ab]	0.074 ± 0.009[ab]
SN-FL-041	Z3	32.6 ± 2.7[bc]	1.64 ± 0.13[de]	0.80 ± 0.05[ab]	24.0 ± 2.0[ab]	17.4 ± 1.1[ab]	0.51 ± 0.04[de]	3.04 ± 0.29[ab]	0.066 ± 0.006[bc]
SN-FL-058	Z3	27.2 ± 1.8[de]	1.61 ± 0.11[e]	0.73 ± 0.05[bc]	24.9 ± 2.0[ab]	15.1 ± 1.1[bc]	0.49 ± 0.04[de]	2.67 ± 0.25[bc]	0.051 ± 0.006[de]

SN-FL-060	Z3	37.2 ± 2.5^{ab}	2.10 ± 0.13^{bc}	0.83 ± 0.06^{ab}	19.3 ± 1.5^{cd}	15.4 ± 1.2^{bc}	0.70 ± 0.06^{ab}	2.54 ± 0.24^{cd}	0.082 ± 0.007^{a}
SN-FL-089	Z3	34.8 ± 2.7^{ab}	2.19 ± 0.15^{ab}	0.84 ± 0.06^{ab}	23.4 ± 1.4^{ab}	16.3 ± 1.1^{ab}	0.64 ± 0.07^{ab}	2.60 ± 0.21^{bc}	0.080 ± 0.008^{ab}
SN-FL-090	Z3	31.7 ± 2.3^{bc}	2.62 ± 0.17^{a}	0.77 ± 0.05^{bc}	23.0 ± 1.9^{ab}	18.5 ± 1.5^{a}	0.65 ± 0.06^{ab}	2.70 ± 0.22^{bc}	0.069 ± 0.006^{ab}
SN-FL-091	Z3	30.3 ± 2.4^{cd}	2.10 ± 0.14^{bc}	0.77 ± 0.06^{bc}	23.4 ± 1.4^{ab}	16.0 ± 1.3^{ab}	0.64 ± 0.07^{ab}	3.32 ± 0.20^{a}	0.060 ± 0.005^{cd}
SN-FL-002	Z4	21.8 ± 1.2^{f}	1.34 ± 0.09^{f}	0.45 ± 0.04^{f}	13.6 ± 1.1^{f}	10.0 ± 0.8^{f}	0.38 ± 0.03^{f}	2.45 ± 0.19^{cd}	0.045 ± 0.005^{ef}
SN-FL-009	Z4	21.9 ± 1.7^{f}	1.28 ± 0.08^{f}	0.41 ± 0.03^{f}	15.4 ± 1.1^{ef}	10.5 ± 0.7^{f}	0.48 ± 0.04^{de}	2.03 ± 0.18^{ef}	0.033 ± 0.003^{f}
SN-FL-010	Z4	25.3 ± 1.7^{ef}	1.24 ± 0.10^{f}	0.56 ± 0.05^{de}	18.3 ± 1.1^{de}	13.1 ± 1.0^{de}	0.40 ± 0.03^{f}	1.90 ± 0.15^{f}	0.049 ± 0.004^{ef}
SN-FL-011	Z4	23.4 ± 1.8^{ef}	1.46 ± 0.09^{ef}	0.59 ± 0.04^{de}	15.0 ± 1.2^{ef}	11.0 ± 0.9^{ef}	0.42 ± 0.04^{f}	2.30 ± 0.20^{de}	0.050 ± 0.005^{de}
SN-FL-012	Z4	18.8 ± 1.2^{f}	1.25 ± 0.08^{f}	0.48 ± 0.04^{ef}	18.1 ± 1.5^{de}	9.3 ± 0.7^{f}	0.46 ± 0.04^{de}	2.18 ± 0.15^{ef}	0.045 ± 0.004^{ef}
SN-FL-013	Z4	17.7 ± 1.0^{f}	1.26 ± 0.07^{f}	0.51 ± 0.04^{ef}	13.5 ± 1.0^{f}	11.5 ± 0.6^{ef}	0.45 ± 0.03^{ef}	2.50 ± 0.17^{cd}	0.044 ± 0.004^{ef}
SN-FL-015	Z4	22.2 ± 1.5^{f}	1.30 ± 0.09^{f}	0.42 ± 0.03^{f}	16.0 ± 1.0^{ef}	12.9 ± 0.9^{de}	0.47 ± 0.05^{de}	2.22 ± 0.20^{de}	0.052 ± 0.005^{de}
SN-FL-016	Z4	26.4 ± 2.2^{de}	1.45 ± 0.10^{ef}	0.46 ± 0.03^{f}	14.0 ± 1.1^{f}	10.6 ± 0.7^{f}	0.44 ± 0.04^{ef}	2.19 ± 0.18^{de}	0.050 ± 0.004^{de}
SN-FL-017	Z4	25.1 ± 1.6^{ef}	1.12 ± 0.07^{f}	0.59 ± 0.04^{de}	18.3 ± 1.3^{de}	14.0 ± 1.0^{cd}	0.41 ± 0.04^{f}	2.15 ± 0.18^{ef}	0.042 ± 0.005^{f}
SN-FL-021	Z4	20.1 ± 1.2^{f}	1.06 ± 0.08^{f}	0.45 ± 0.03^{f}	15.8 ± 1.2^{ef}	10.8 ± 0.8^{f}	0.52 ± 0.04^{cd}	2.48 ± 0.19^{cd}	0.057 ± 0.006^{cd}
SN-FL-024	Z4	21.8 ± 1.8^{f}	1.35 ± 0.10^{ef}	0.42 ± 0.04^{f}	12.6 ± 0.8^{f}	11.3 ± 1.0^{ef}	0.43 ± 0.04^{ef}	2.52 ± 0.21^{cd}	0.035 ± 0.004^{f}
SN-FL-025	Z4	19.6 ± 1.2^{f}	1.53 ± 0.10^{ef}	0.57 ± 0.05^{de}	16.7 ± 1.3^{ef}	9.8 ± 0.7^{f}	0.57 ± 0.04^{bc}	1.76 ± 0.15^{f}	0.037 ± 0.004^{f}
SN-FL-028	Z4	24.9 ± 1.8^{ef}	1.50 ± 0.12^{ef}	0.40 ± 0.03^{f}	16.3 ± 1.0^{ef}	11.2 ± 0.8^{ef}	0.43 ± 0.03^{f}	2.07 ± 0.17^{ef}	0.052 ± 0.006^{de}
SN-FL-030	Z4	25.2 ± 1.8^{ef}	1.22 ± 0.08^{f}	0.40 ± 0.03^{f}	15.3 ± 1.0^{ef}	12.7 ± 0.7^{de}	0.40 ± 0.03^{f}	1.91 ± 0.16^{f}	0.050 ± 0.004^{de}
SN-FL-031	Z4	26.8 ± 2.2^{de}	1.25 ± 0.10^{f}	0.46 ± 0.03^{f}	13.4 ± 1.1^{f}	10.5 ± 0.6^{f}	0.43 ± 0.03^{f}	2.12 ± 0.16^{ef}	0.052 ± 0.005^{de}
SN-FL-070	Z4	25.2 ± 2.1^{ef}	1.19 ± 0.07^{f}	0.48 ± 0.04^{ef}	12.2 ± 0.8^{f}	9.5 ± 0.7^{f}	0.51 ± 0.04^{de}	2.25 ± 0.14^{de}	0.040 ± 0.003^{f}
SN-FL-071	Z4	20.6 ± 1.7^{f}	1.49 ± 0.10^{ef}	0.59 ± 0.05^{de}	14.5 ± 1.2^{f}	9.7 ± 0.6^{f}	0.48 ± 0.05^{de}	2.33 ± 0.19^{de}	0.038 ± 0.004^{f}
SN-FL-072	Z4	23.7 ± 1.8^{ef}	1.51 ± 0.11^{ef}	0.40 ± 0.03^{f}	14.6 ± 0.8^{f}	11.0 ± 0.8^{ef}	0.42 ± 0.04^{f}	1.83 ± 0.15^{f}	0.035 ± 0.004^{f}
SN-FL-042	Z5	30.3 ± 2.2^{cd}	1.93 ± 0.14^{cd}	0.69 ± 0.06^{cd}	19.1 ± 1.3^{cd}	11.4 ± 0.9^{ef}	0.62 ± 0.04^{bc}	2.24 ± 0.17^{de}	0.050 ± 0.006^{de}
SN-FL-043	Z5	33.3 ± 2.0^{ab}	1.84 ± 0.12^{cd}	0.73 ± 0.06^{bc}	17.3 ± 1.1^{e}	15.1 ± 0.9^{bc}	0.57 ± 0.05^{bc}	2.35 ± 0.15^{de}	0.047 ± 0.005^{ef}
SN-FL-044	Z5	24.8 ± 1.9^{ef}	1.98 ± 0.16^{bc}	0.81 ± 0.07^{ab}	23.1 ± 1.9^{ab}	15.9 ± 1.0^{ab}	0.61 ± 0.05^{bc}	2.18 ± 0.17^{ef}	0.064 ± 0.005^{bc}
SN-FL-045	Z5	29.5 ± 2.2^{cd}	1.63 ± 0.14^{de}	0.59 ± 0.05^{de}	21.8 ± 1.7^{bc}	14.5 ± 0.9^{cd}	0.57 ± 0.04^{bc}	2.38 ± 0.20^{cd}	0.054 ± 0.005^{cd}
SN-FL-046	Z5	29.6 ± 2.2^{cd}	1.81 ± 0.10^{cd}	0.69 ± 0.05^{cd}	20.6 ± 1.3^{cd}	15.2 ± 0.9^{bc}	0.49 ± 0.04^{de}	2.98 ± 0.19^{ab}	0.050 ± 0.005^{de}
SN-FL-047	Z5	31.2 ± 2.6^{bc}	2.16 ± 0.18^{ab}	0.65 ± 0.05^{cd}	20.3 ± 1.5^{cd}	13.0 ± 0.9^{de}	0.53 ± 0.05^{cd}	2.23 ± 0.18^{de}	0.053 ± 0.005^{de}

SN-FL-066	Z5	27.7 ± 2.3^{de}	1.47 ± 0.12^{ef}	0.65 ± 0.05^{cd}	19.2 ± 1.3^{cd}	13.2 ± 0.8^{de}	0.50 ± 0.04^{de}	2.75 ± 0.19^{bc}	0.056 ± 0.005^{cd}
SN-FL-067	Z5	31.4 ± 2.0^{bc}	1.88 ± 0.15^{cd}	0.63 ± 0.04^{de}	21.7 ± 1.2^{bc}	14.0 ± 1.1^{cd}	0.50 ± 0.04^{de}	2.43 ± 0.16^{cd}	0.061 ± 0.005^{cd}
SN-FL-068	Z5	22.8 ± 1.9^{ef}	1.81 ± 0.15^{cd}	0.60 ± 0.04^{de}	18.8 ± 1.3^{de}	15.4 ± 1.0^{bc}	0.65 ± 0.06^{ab}	2.53 ± 0.19^{cd}	0.059 ± 0.006^{cd}
SN-FL-073	Z5	27.7 ± 1.7^{de}	1.85 ± 0.14^{cd}	0.54 ± 0.04^{ef}	18.0 ± 1.0^{de}	14.7 ± 1.1^{bc}	0.49 ± 0.04^{de}	2.41 ± 0.16^{cd}	0.054 ± 0.005^{cd}
SN-FL-074	Z5	29.3 ± 1.9^{cd}	2.15 ± 0.14^{bc}	0.70 ± 0.05^{cd}	19.8 ± 1.3^{cd}	13.4 ± 1.0^{de}	0.60 ± 0.04^{bc}	2.79 ± 0.20^{bc}	0.049 ± 0.004^{ef}
SN-FL-075	Z5	28.1 ± 2.1^{de}	2.29 ± 0.14^{ab}	0.77 ± 0.07^{ab}	22.2 ± 1.7^{bc}	14.2 ± 1.2^{cd}	0.59 ± 0.06^{bc}	2.45 ± 0.17^{cd}	0.069 ± 0.007^{ab}
SN-FL-076	Z5	30.8 ± 2.3^{cd}	1.71 ± 0.11^{de}	0.62 ± 0.05^{de}	19.0 ± 1.6^{cd}	13.9 ± 0.8^{cd}	0.50 ± 0.04^{de}	2.36 ± 0.15^{de}	0.068 ± 0.006^{ab}
SN-FL-077	Z5	28.9 ± 1.8^{de}	2.09 ± 0.13^{bc}	0.56 ± 0.04^{e}	20.7 ± 1.4^{cd}	13.0 ± 0.9^{de}	0.52 ± 0.05^{cd}	2.57 ± 0.20^{cd}	0.058 ± 0.006^{cd}
SN-FL-078	Z5	32.1 ± 2.3^{bc}	1.74 ± 0.11^{de}	0.78 ± 0.07^{ab}	20.1 ± 1.2^{cd}	13.2 ± 0.8^{de}	0.58 ± 0.05^{bc}	2.16 ± 0.18^{ef}	0.067 ± 0.006^{bc}
SN-FL-079	Z5	32.9 ± 2.1^{ab}	1.87 ± 0.11^{cd}	0.68 ± 0.05^{cd}	17.8 ± 1.2^{de}	13.7 ± 1.0^{cd}	0.55 ± 0.05^{cd}	2.29 ± 0.21^{de}	0.056 ± 0.006^{cd}
SN-FL-080	Z5	30.4 ± 2.2^{cd}	1.79 ± 0.12^{cd}	0.58 ± 0.05^{de}	19.2 ± 1.6^{cd}	13.9 ± 1.1^{cd}	0.56 ± 0.04^{cd}	2.26 ± 0.14^{de}	0.059 ± 0.005^{cd}
SN-FL-092	Z5	29.2 ± 2.1^{cd}	1.77 ± 0.10^{de}	0.72 ± 0.04^{cd}	18.9 ± 1.4^{de}	13.6 ± 0.9^{de}	0.51 ± 0.04^{de}	2.56 ± 0.19^{cd}	0.054 ± 0.005^{cd}
SN-FL-093	Z5	29.8 ± 2.3^{cd}	1.53 ± 0.12^{ef}	0.77 ± 0.06^{bc}	21.3 ± 1.2^{cd}	13.3 ± 0.9^{de}	0.52 ± 0.05^{cd}	2.32 ± 0.15^{de}	0.056 ± 0.005^{cd}

Notes: Values are reported as mean ± analytical SD of three independently prepared analytical portions on a dry-weight basis (mg/kg DW). The three analytical measurements were used only to assess analytical precision and were averaged to obtain one site-level concentration per matrix and georeferenced sampling site for subsequent statistical analyses. Values <LOD and <LOQ are indicated as applicable.

Table S26. Individual concentrations of potentially toxic and environmentally relevant trace elements measured in georeferenced *Sambucus nigra* L. flower samples collected across the investigated ecological sectors of the Oradea metropolitan area, Romania.

Sample ID	Zone	^{208}Pb (mg/kg)	^{111}Cd (mg/kg)	^{75}As (mg/kg)	^{202}Hg (mg/kg)	^{52}Cr (mg/kg)	^{60}Ni (mg/kg)	^{121}Sb (mg/kg)	^{118}Sn (mg/kg)
SN-FL-022	Z1	0.038 ± 0.004^{f}	<LOD	<LOD	<LOD	0.25 ± 0.02^{e}	0.21 ± 0.01^{e}	0.0033 ± 0.0004^{ef}	0.006 ± 0.001^{ef}
SN-FL-023	Z1	<LOD	<LOD	0.006 ± 0.001^{ef}	<LOD	0.26 ± 0.02^{e}	0.20 ± 0.01^{e}	<LOD	0.008 ± 0.001^{de}
SN-FL-026	Z1	<LOD	<LOD	<LOD	<LOD	0.36 ± 0.03^{cd}	0.19 ± 0.01^{e}	<LOD	0.010 ± 0.001^{d}
SN-FL-027	Z1	<LOD	<LOD	<LOD	<LOD	0.41 ± 0.03^{cd}	0.19 ± 0.02^{ef}	<LOD	0.010 ± 0.001^{d}
SN-FL-029	Z1	<LOD	<LOD	0.006 ± 0.001^{ef}	<LOD	0.36 ± 0.02^{d}	0.21 ± 0.02^{e}	<LOD	0.006 ± 0.001^{ef}
SN-FL-053	Z1	<LOD	<LOD	<LOD	<LOD	0.25 ± 0.02^{e}	0.16 ± 0.01^{f}	<LOD	0.008 ± 0.001^{de}
SN-FL-061	Z1	<LOD	<LOD	<LOD	<LOD	0.29 ± 0.02^{de}	0.24 ± 0.02^{de}	<LOD	0.008 ± 0.001^{de}
SN-FL-062	Z1	<LOD	<LOD	<LOD	<LOD	0.29 ± 0.02^{de}	0.19 ± 0.01^{e}	0.0040 ± 0.0005^{de}	0.008 ± 0.001^{de}

SN-FL-063	Z1	0.045 ± 0.005[ef]	0.0054 ± 0.0005[ef]	<LOD	<LOD	0.29 ± 0.02[de]	0.23 ± 0.02[de]	<LOD	0.009 ± 0.001[de]
SN-FL-081	Z1	0.051 ± 0.005[e]	<LOD	0.010 ± 0.001[cd]	<LOD	0.25 ± 0.02[e]	0.22 ± 0.02[de]	<LOD	0.010 ± 0.001[d]
SN-FL-082	Z1	<LOD	<LOD	<LOD	<LOD	0.21 ± 0.02[ef]	0.17 ± 0.01[ef]	<LOD	0.009 ± 0.001[de]
SN-FL-083	Z1	<LOD	<LOD	<LOD	<LOD	0.25 ± 0.03[e]	0.18 ± 0.01[ef]	<LOD	0.007 ± 0.001[ef]
SN-FL-084	Z1	0.035 ± 0.003[f]	<LOD	0.009 ± 0.001[d]	<LOD	0.26 ± 0.02[e]	0.22 ± 0.02[de]	<LOD	0.008 ± 0.001[de]
SN-FL-003	Z2	0.231 ± 0.020[a]	0.0183 ± 0.0018[b]	0.009 ± 0.001[d]	<LOD	0.54 ± 0.04[b]	0.52 ± 0.05[b]	0.0129 ± 0.0014[a]	0.027 ± 0.003[a]
SN-FL-004	Z2	0.077 ± 0.009[d]	0.0107 ± 0.0014[c]	0.025 ± 0.003[ab]	<LOD	0.70 ± 0.06[a]	0.56 ± 0.04[b]	0.0090 ± 0.0009[b]	0.025 ± 0.002[ab]
SN-FL-014	Z2	0.169 ± 0.014[b]	0.0240 ± 0.0028[a]	0.035 ± 0.003[a]	0.0016 ± 0.0002[ab]	0.71 ± 0.05[a]	0.65 ± 0.06[ab]	0.0114 ± 0.0013[ab]	0.017 ± 0.002[bc]
SN-FL-018	Z2	0.244 ± 0.018[a]	0.0097 ± 0.0012[cd]	0.021 ± 0.003[ab]	0.0011 ± 0.0001[cd]	0.49 ± 0.05[bc]	0.70 ± 0.06[a]	0.0031 ± 0.0004[f]	0.026 ± 0.003[ab]
SN-FL-019	Z2	0.095 ± 0.011[cd]	0.0214 ± 0.0024[ab]	0.026 ± 0.002[a]	<LOD	0.72 ± 0.06[a]	0.79 ± 0.07[a]	0.0079 ± 0.0011[bc]	0.015 ± 0.002[c]
SN-FL-020	Z2	0.138 ± 0.015[bc]	0.0177 ± 0.0021[b]	0.019 ± 0.002[b]	0.0013 ± 0.0002[ab]	0.60 ± 0.06[ab]	0.49 ± 0.04[b]	0.0088 ± 0.0013[bc]	0.022 ± 0.003[ab]
SN-FL-048	Z2	0.217 ± 0.021[ab]	0.0211 ± 0.0024[ab]	0.024 ± 0.003[ab]	<LOD	0.62 ± 0.05[ab]	0.57 ± 0.05[ab]	0.0095 ± 0.0012[ab]	0.026 ± 0.003[ab]
SN-FL-049	Z2	0.196 ± 0.023[ab]	0.0124 ± 0.0012[bc]	0.024 ± 0.003[ab]	0.0008 ± 0.0001[ef]	0.55 ± 0.05[ab]	0.49 ± 0.04[b]	0.0162 ± 0.0020[a]	0.023 ± 0.002[ab]
SN-FL-050	Z2	0.245 ± 0.026[a]	0.0222 ± 0.0027[ab]	0.013 ± 0.002[bc]	<LOD	0.49 ± 0.04[bc]	0.73 ± 0.06[a]	0.0159 ± 0.0019[a]	0.024 ± 0.003[ab]
SN-FL-051	Z2	0.075 ± 0.006[d]	0.0197 ± 0.0019[ab]	0.025 ± 0.002[ab]	0.0011 ± 0.0001[cd]	0.59 ± 0.04[ab]	0.40 ± 0.03[c]	0.0079 ± 0.0011[bc]	0.027 ± 0.003[a]
SN-FL-052	Z2	0.173 ± 0.018[b]	0.0249 ± 0.0026[a]	0.024 ± 0.003[ab]	<LOD	0.80 ± 0.08[a]	0.44 ± 0.04[bc]	0.0127 ± 0.0014[a]	0.028 ± 0.003[a]
SN-FL-054	Z2	0.228 ± 0.024[a]	0.0147 ± 0.0015[b]	0.013 ± 0.001[bc]	0.0008 ± 0.0001[ef]	0.57 ± 0.06[ab]	0.68 ± 0.07[a]	0.0094 ± 0.0010[ab]	0.032 ± 0.003[a]
SN-FL-055	Z2	0.054 ± 0.006[e]	0.0088 ± 0.0011[cd]	0.013 ± 0.002[bc]	<LOD	0.78 ± 0.05[a]	0.79 ± 0.07[a]	0.0060 ± 0.0007[c]	0.012 ± 0.001[cd]
SN-FL-056	Z2	0.214 ± 0.020[ab]	0.0150 ± 0.0020[b]	0.025 ± 0.002[ab]	<LOD	0.69 ± 0.07[a]	0.74 ± 0.07[a]	0.0114 ± 0.0014[ab]	0.031 ± 0.003[a]
SN-FL-057	Z2	0.216 ± 0.022[ab]	0.0206 ± 0.0022[ab]	0.012 ± 0.001[bc]	0.0008 ± 0.0001[ef]	0.54 ± 0.04[b]	0.63 ± 0.05[ab]	0.0103 ± 0.0013[ab]	0.031 ± 0.003[a]
SN-FL-059	Z2	0.153 ± 0.016[bc]	0.0234 ± 0.0023[a]	0.027 ± 0.002[a]	<LOD	0.67 ± 0.06[a]	0.66 ± 0.06[ab]	0.0055 ± 0.0008[cd]	0.025 ± 0.003[ab]
SN-FL-064	Z2	0.182 ± 0.017[ab]	0.0079 ± 0.0009[cd]	0.010 ± 0.001[cd]	<LOD	1.04 ± 0.09[a]	0.79 ± 0.05[a]	0.0127 ± 0.0014[a]	0.035 ± 0.004[a]
SN-FL-065	Z2	0.258 ± 0.020[a]	0.0216 ± 0.0029[ab]	0.029 ± 0.003[a]	<LOD	0.72 ± 0.06[a]	0.42 ± 0.03[bc]	0.0086 ± 0.0009[b]	0.042 ± 0.004[a]
SN-FL-069	Z2	0.199 ± 0.017[ab]	0.0234 ± 0.0024[ab]	0.023 ± 0.003[ab]	<LOD	0.56 ± 0.05[ab]	0.67 ± 0.05[a]	0.0098 ± 0.0012[ab]	0.023 ± 0.003[ab]
SN-FL-085	Z2	0.189 ± 0.018[ab]	0.0070 ± 0.0007[de]	0.009 ± 0.001[d]	<LOD	0.69 ± 0.05[a]	0.78 ± 0.06[a]	0.0072 ± 0.0010[bc]	0.034 ± 0.003[a]
SN-FL-086	Z2	0.255 ± 0.020[a]	0.0122 ± 0.0015[bc]	0.020 ± 0.003[b]	<LOD	0.60 ± 0.06[ab]	0.91 ± 0.09[a]	0.0036 ± 0.0005[de]	0.023 ± 0.002[ab]
SN-FL-087	Z2	0.183 ± 0.018[ab]	0.0285 ± 0.0026[a]	0.027 ± 0.004[ab]	0.0014 ± 0.0002[ab]	0.80 ± 0.06[a]	0.78 ± 0.06[a]	0.0102 ± 0.0015[ab]	0.026 ± 0.003[ab]
SN-FL-088	Z2	0.139 ± 0.013[bc]	0.0230 ± 0.0032[ab]	0.021 ± 0.002[b]	0.0015 ± 0.0002[ab]	0.47 ± 0.03[bc]	0.82 ± 0.06[a]	0.0115 ± 0.0013[a]	0.035 ± 0.003[a]

SN-FL-094	Z2	0.208 ± 0.022[ab]	0.0211 ± 0.0025[ab]	0.034 ± 0.003[a]	<LOD	0.87 ± 0.07[a]	0.99 ± 0.07[a]	0.0087 ± 0.0011[bc]	0.013 ± 0.002[cd]
SN-FL-001	Z3	0.044 ± 0.004[ef]	0.0102 ± 0.0012[c]	<LOD	<LOD	0.39 ± 0.04[cd]	0.32 ± 0.03[d]	<LOD	0.019 ± 0.002[bc]
SN-FL-005	Z3	0.038 ± 0.004[f]	0.0057 ± 0.0008[ef]	<LOD	<LOD	0.51 ± 0.03[b]	0.31 ± 0.03[d]	0.0030 ± 0.0004[f]	0.016 ± 0.001[c]
SN-FL-006	Z3	0.087 ± 0.010[cd]	0.0057 ± 0.0007[ef]	0.008 ± 0.001[de]	<LOD	0.37 ± 0.02[cd]	0.33 ± 0.03[d]	0.0043 ± 0.0004[de]	0.014 ± 0.002[cd]
SN-FL-007	Z3	0.074 ± 0.009[d]	0.0058 ± 0.0007[ef]	0.007 ± 0.001[de]	<LOD	0.41 ± 0.03[cd]	0.23 ± 0.02[de]	0.0048 ± 0.0005[cd]	0.022 ± 0.002[ab]
SN-FL-008	Z3	0.035 ± 0.004[f]	0.0081 ± 0.0011[cd]	0.011 ± 0.001[cd]	<LOD	0.48 ± 0.04[bc]	0.43 ± 0.04[bc]	0.0040 ± 0.0004[de]	0.018 ± 0.002[bc]
SN-FL-032	Z3	0.073 ± 0.009[de]	0.0069 ± 0.0006[de]	0.011 ± 0.001[cd]	<LOD	0.31 ± 0.02[d]	0.25 ± 0.02[de]	0.0046 ± 0.0005[cd]	0.013 ± 0.001[cd]
SN-FL-033	Z3	0.054 ± 0.004[e]	0.0086 ± 0.0009[cd]	0.010 ± 0.001[cd]	<LOD	0.23 ± 0.02[e]	0.27 ± 0.02[d]	0.0055 ± 0.0005[cd]	0.012 ± 0.001[cd]
SN-FL-034	Z3	0.037 ± 0.004[f]	0.0141 ± 0.0015[b]	0.009 ± 0.001[d]	<LOD	0.33 ± 0.03[d]	0.24 ± 0.02[de]	0.0044 ± 0.0005[de]	0.005 ± 0.001[f]
SN-FL-035	Z3	0.046 ± 0.005[ef]	0.0120 ± 0.0013[bc]	0.011 ± 0.001[cd]	<LOD	0.47 ± 0.03[bc]	0.34 ± 0.03[cd]	0.0048 ± 0.0007[cd]	0.016 ± 0.002[bc]
SN-FL-036	Z3	0.064 ± 0.006[de]	0.0053 ± 0.0005[ef]	0.011 ± 0.001[cd]	<LOD	0.33 ± 0.02[d]	0.43 ± 0.03[bc]	0.0037 ± 0.0004[de]	0.009 ± 0.001[de]
SN-FL-037	Z3	0.105 ± 0.012[c]	0.0071 ± 0.0008[de]	0.007 ± 0.001[de]	<LOD	0.34 ± 0.03[d]	0.40 ± 0.04[bc]	0.0031 ± 0.0003[f]	0.008 ± 0.001[de]
SN-FL-038	Z3	<LOD	0.0057 ± 0.0007[ef]	<LOD	<LOD	0.41 ± 0.03[cd]	0.45 ± 0.04[bc]	0.0040 ± 0.0005[de]	0.017 ± 0.002[bc]
SN-FL-039	Z3	0.101 ± 0.009[c]	<LOD	0.009 ± 0.001[d]	<LOD	0.43 ± 0.04[bc]	0.36 ± 0.03[cd]	<LOD	0.009 ± 0.001[de]
SN-FL-040	Z3	0.045 ± 0.003[ef]	<LOD	0.008 ± 0.001[de]	<LOD	0.30 ± 0.03[de]	0.43 ± 0.03[bc]	0.0047 ± 0.0006[cd]	0.010 ± 0.001[d]
SN-FL-041	Z3	0.059 ± 0.007[de]	<LOD	0.006 ± 0.001[ef]	<LOD	0.47 ± 0.04[bc]	0.27 ± 0.02[d]	0.0032 ± 0.0004[ef]	0.017 ± 0.002[bc]
SN-FL-058	Z3	0.088 ± 0.010[cd]	0.0053 ± 0.0006[ef]	0.007 ± 0.001[de]	<LOD	0.39 ± 0.03[cd]	0.40 ± 0.03[c]	0.0040 ± 0.0005[de]	0.018 ± 0.002[bc]
SN-FL-060	Z3	0.065 ± 0.006[de]	0.0077 ± 0.0008[d]	<LOD	<LOD	0.62 ± 0.06[ab]	0.36 ± 0.04[cd]	0.0046 ± 0.0006[cd]	0.017 ± 0.002[bc]
SN-FL-089	Z3	0.109 ± 0.008[c]	0.0057 ± 0.0007[ef]	0.007 ± 0.001[de]	<LOD	0.26 ± 0.02[e]	0.29 ± 0.03[d]	0.0036 ± 0.0005[de]	0.014 ± 0.001[c]
SN-FL-090	Z3	0.069 ± 0.005[de]	0.0068 ± 0.0007[de]	0.008 ± 0.001[de]	<LOD	0.32 ± 0.02[d]	0.48 ± 0.04[b]	<LOD	0.019 ± 0.002[bc]
SN-FL-091	Z3	0.044 ± 0.005[ef]	0.0063 ± 0.0008[de]	<LOD	<LOD	0.34 ± 0.03[d]	0.47 ± 0.05[bc]	0.0030 ± 0.0004[f]	0.010 ± 0.001[d]
SN-FL-002	Z4	<LOD	<LOD	<LOD	<LOD	0.21 ± 0.02[ef]	0.16 ± 0.01[f]	<LOD	0.005 ± 0.001[f]
SN-FL-009	Z4	<LOD	<LOD	<LOD	<LOD	0.21 ± 0.02[ef]	0.15 ± 0.01[f]	<LOD	0.004 ± 0.001[f]
SN-FL-010	Z4	<LOD	<LOD	<LOD	<LOD	0.16 ± 0.01[f]	0.18 ± 0.01[ef]	<LOD	0.004 ± 0.001[f]
SN-FL-011	Z4	<LOD	<LOD	<LOD	<LOD	0.20 ± 0.01[ef]	0.15 ± 0.01[f]	<LOD	<LOD
SN-FL-012	Z4	<LOD	<LOD	<LOD	<LOD	0.18 ± 0.02[f]	0.19 ± 0.02[ef]	<LOD	0.007 ± 0.001[ef]
SN-FL-013	Z4	0.038 ± 0.003[f]	<LOD	<LOD	<LOD	0.14 ± 0.01[f]	0.16 ± 0.01[f]	<LOD	0.004 ± 0.001[f]
SN-FL-015	Z4	<LOD	<LOD	<LOD	<LOD	0.18 ± 0.02[f]	0.18 ± 0.01[ef]	<LOD	0.007 ± 0.001[ef]

SN-FL-016	Z4	<LOD	<LOD	<LOD	<LOD	0.18 ± 0.02^{f}	0.14 ± 0.01^{f}	<LOD	0.005 ± 0.001^{f}
SN-FL-017	Z4	<LOD	<LOD	<LOD	<LOD	0.15 ± 0.01^{f}	0.07 ± 0.01^{f}	<LOD	0.004 ± 0.001^{f}
SN-FL-021	Z4	<LOD	<LOD	<LOD	<LOD	0.22 ± 0.02^{ef}	0.19 ± 0.02^{ef}	<LOD	0.007 ± 0.001^{ef}
SN-FL-024	Z4	<LOD	<LOD	<LOD	<LOD	0.19 ± 0.02^{ef}	0.15 ± 0.01^{f}	<LOD	0.005 ± 0.001^{f}
SN-FL-025	Z4	<LOD	<LOD	<LOD	<LOD	0.19 ± 0.02^{ef}	0.11 ± 0.01^{f}	<LOD	0.005 ± 0.001^{f}
SN-FL-028	Z4	<LOD	<LOD	<LOD	<LOD	0.20 ± 0.02^{ef}	0.13 ± 0.01^{f}	<LOD	0.004 ± 0.001^{f}
SN-FL-030	Z4	<LOD	<LOD	<LOD	<LOD	0.18 ± 0.02^{f}	0.17 ± 0.01^{ef}	<LOD	0.004 ± 0.001^{f}
SN-FL-031	Z4	<LOD	<LOD	<LOD	<LOD	0.19 ± 0.02^{ef}	0.17 ± 0.01^{ef}	<LOD	0.005 ± 0.001^{f}
SN-FL-070	Z4	<LOD	<LOD	<LOD	<LOD	0.13 ± 0.01^{f}	0.13 ± 0.01^{f}	<LOD	0.004 ± 0.001^{f}
SN-FL-071	Z4	<LOD	<LOD	<LOD	<LOD	0.17 ± 0.02^{f}	0.15 ± 0.01^{f}	<LOD	0.007 ± 0.001^{ef}
SN-FL-072	Z4	<LOD	<LOD	0.007 ± 0.001^{de}	<LOD	0.17 ± 0.02^{f}	0.19 ± 0.02^{ef}	<LOD	0.007 ± 0.001^{ef}
SN-FL-042	Z5	0.066 ± 0.007^{de}	0.0092 ± 0.0012^{cd}	0.008 ± 0.001^{de}	<LOD	0.36 ± 0.03^{cd}	0.41 ± 0.04^{bc}	0.0035 ± 0.0004^{ef}	0.010 ± 0.001^{d}
SN-FL-043	Z5	0.045 ± 0.004^{ef}	0.0051 ± 0.0007^{ef}	0.006 ± 0.001^{ef}	0.0010 ± 0.0001^{cd}	0.42 ± 0.03^{c}	0.41 ± 0.04^{bc}	<LOD	0.022 ± 0.002^{ab}
SN-FL-044	Z5	0.049 ± 0.004^{e}	0.0053 ± 0.0006^{ef}	0.007 ± 0.001^{de}	<LOD	0.32 ± 0.03^{d}	0.45 ± 0.04^{bc}	0.0045 ± 0.0006^{de}	0.009 ± 0.001^{de}
SN-FL-045	Z5	0.151 ± 0.018^{bc}	0.0077 ± 0.0007^{d}	0.010 ± 0.001^{cd}	<LOD	0.39 ± 0.04^{cd}	0.38 ± 0.04^{cd}	0.0034 ± 0.0005^{ef}	0.009 ± 0.001^{de}
SN-FL-046	Z5	0.151 ± 0.018^{bc}	0.0057 ± 0.0008^{ef}	0.008 ± 0.001^{de}	<LOD	0.40 ± 0.03^{cd}	0.40 ± 0.04^{bc}	0.0054 ± 0.0008^{cd}	0.019 ± 0.002^{bc}
SN-FL-047	Z5	0.052 ± 0.005^{e}	0.0079 ± 0.0008^{cd}	0.016 ± 0.002^{b}	<LOD	0.55 ± 0.05^{ab}	0.40 ± 0.03^{c}	0.0035 ± 0.0005^{ef}	0.015 ± 0.002^{c}
SN-FL-066	Z5	0.105 ± 0.009^{c}	0.0057 ± 0.0006^{ef}	<LOD	<LOD	0.42 ± 0.04^{cd}	0.52 ± 0.04^{b}	0.0035 ± 0.0003^{ef}	0.017 ± 0.002^{bc}
SN-FL-067	Z5	<LOD	<LOD	0.008 ± 0.001^{de}	<LOD	0.43 ± 0.03^{c}	0.40 ± 0.04^{bc}	<LOD	0.013 ± 0.001^{cd}
SN-FL-068	Z5	0.119 ± 0.010^{c}	0.0091 ± 0.0011^{cd}	0.008 ± 0.001^{de}	<LOD	0.36 ± 0.03^{cd}	0.59 ± 0.06^{ab}	0.0037 ± 0.0005^{de}	0.010 ± 0.001^{d}
SN-FL-073	Z5	0.077 ± 0.006^{d}	0.0058 ± 0.0007^{ef}	0.007 ± 0.001^{de}	<LOD	0.45 ± 0.03^{bc}	0.31 ± 0.03^{d}	<LOD	0.012 ± 0.001^{cd}
SN-FL-074	Z5	<LOD	<LOD	0.007 ± 0.001^{de}	<LOD	0.49 ± 0.03^{bc}	0.36 ± 0.03^{cd}	0.0036 ± 0.0005^{de}	0.019 ± 0.002^{bc}
SN-FL-075	Z5	0.111 ± 0.010^{c}	0.0066 ± 0.0008^{de}	0.006 ± 0.001^{ef}	<LOD	0.38 ± 0.04^{cd}	0.39 ± 0.03^{cd}	0.0069 ± 0.0010^{bc}	0.017 ± 0.002^{bc}
SN-FL-076	Z5	0.036 ± 0.004^{f}	<LOD	0.009 ± 0.001^{d}	<LOD	0.44 ± 0.04^{bc}	0.55 ± 0.05^{b}	0.0042 ± 0.0005^{de}	0.019 ± 0.002^{bc}
SN-FL-077	Z5	0.109 ± 0.013^{c}	0.0067 ± 0.0009^{de}	<LOD	<LOD	0.56 ± 0.05^{ab}	0.44 ± 0.04^{bc}	0.0064 ± 0.0008^{c}	0.019 ± 0.002^{bc}
SN-FL-078	Z5	<LOD	<LOD	0.017 ± 0.002^{b}	<LOD	0.28 ± 0.02^{de}	0.39 ± 0.04^{cd}	<LOD	0.016 ± 0.002^{bc}
SN-FL-079	Z5	0.039 ± 0.003^{f}	0.0083 ± 0.0009^{cd}	0.009 ± 0.001^{d}	<LOD	0.26 ± 0.02^{e}	0.31 ± 0.02^{d}	0.0055 ± 0.0008^{cd}	0.022 ± 0.003^{ab}
SN-FL-080	Z5	0.106 ± 0.009^{c}	0.0067 ± 0.0009^{de}	0.011 ± 0.001^{cd}	<LOD	0.41 ± 0.04^{cd}	0.55 ± 0.04^{b}	<LOD	0.017 ± 0.002^{bc}

SN-FL-092	Z5	0.145 ± 0.016^{bc}	<LOD	<LOD	<LOD	0.44 ± 0.05^{bc}	0.44 ± 0.03^{bc}	0.0054 ± 0.0006^{cd}	0.006 ± 0.001^{ef}
SN-FL-093	Z5	0.088 ± 0.011^{cd}	<LOD	0.006 ± 0.001^{ef}	<LOD	0.26 ± 0.02^{e}	0.33 ± 0.03^{d}	<LOD	0.015 ± 0.001^{c}

Notes: Values are reported as mean ± analytical SD of three independently prepared analytical portions on a dry-weight basis (mg/kg DW). The three analytical measurements were used only to assess analytical precision and were averaged to obtain one site-level concentration per matrix and georeferenced sampling site for subsequent statistical analyses. Values <LOD and <LOQ are indicated as applicable.

Table S27. Individual concentrations of macroelements in *Sambucus nigra* L. fruit samples collected from georeferenced sampling points across the investigated ecological sectors of the Oradea metropolitan area, Romania.

Sample ID	Zone	K (mg/kg)	Ca (mg/kg)	Mg (mg/kg)	Na (mg/kg)	P (mg/kg)	S (mg/kg)
SN-FR-022	Z1	3665 ± 293^{b}	2247 ± 157^{i}	944 ± 76.0^{hi}	44.0 ± 4.00^{o}	1202 ± 108^{q}	1629 ± 147^{n}
SN-FR-023	Z1	3674 ± 294^{b}	2325 ± 163^{h}	757 ± 61.0^{no}	73.0 ± 7.00^{i}	1519 ± 137^{lm}	1913 ± 172^{k}
SN-FR-026	Z1	3743 ± 299^{b}	2192 ± 153^{j}	1009 ± 81.0^{fg}	76.0 ± 8.00^{hi}	1330 ± 120^{op}	1694 ± 152^{m}
SN-FR-027	Z1	3100 ± 248^{bc}	2114 ± 148^{k}	770 ± 62.0^{n}	42.0 ± 4.00^{o}	1188 ± 107^{qr}	2061 ± 186^{ij}
SN-FR-029	Z1	3196 ± 256^{bc}	2011 ± 141^{lm}	900 ± 72.0^{j}	78.0 ± 8.00^{h}	1053 ± 94.8st	1597 ± 144^{n}
SN-FR-053	Z1	3046 ± 244^{c}	2435 ± 170^{fg}	974 ± 78.0^{h}	79.0 ± 8.00^{h}	1370 ± 123°	2222 ± 200^{gh}
SN-FR-061	Z1	3235 ± 259^{bc}	2350 ± 165^{gh}	844 ± 68.0^{l}	53.0 ± 5.00^{m}	1323 ± 119^{op}	2048 ± 184^{ij}
SN-FR-062	Z1	3120 ± 250^{bc}	1893 ± 133^{n}	888 ± 71.0^{jk}	56.0 ± 6.00^{lm}	1472 ± 132^{m}	1462 ± 132^{op}
SN-FR-063	Z1	3321 ± 266^{bc}	1952 ± 137^{m}	883 ± 71.0^{jk}	51.0 ± 5.00^{mn}	1228 ± 111^{q}	1887 ± 170^{k}
SN-FR-081	Z1	3682 ± 295^{b}	2123 ± 149^{k}	840 ± 67.0^{l}	80.0 ± 8.00^{gh}	1282 ± 115^{p}	1816 ± 163^{l}
SN-FR-082	Z1	3432 ± 275^{b}	2409 ± 169^{g}	927 ± 74.0^{i}	71.0 ± 7.00^{ij}	1411 ± 127^{n}	1526 ± 137^{o}
SN-FR-083	Z1	3304 ± 264^{bc}	2004 ± 140^{lm}	739 ± 59.0^{o}	76.0 ± 8.00^{hi}	1289 ± 116^{p}	1990 ± 179^{j}
SN-FR-084	Z1	3701 ± 296^{b}	2166 ± 152^{j}	895 ± 72.0^{j}	71.0 ± 7.00^{ij}	1242 ± 112^{pq}	1500 ± 135^{o}
SN-FR-003	Z2	4424 ± 265^{a}	2681 ± 188^{c}	1052 ± 84.0^{ef}	84.0 ± 8.00^{g}	2263 ± 204^{ab}	2751 ± 248^{b}
SN-FR-004	Z2	3710 ± 297^{b}	2326 ± 163^{h}	1113 ± 89.0^{cd}	83.0 ± 8.00^{g}	1926 ± 173^{fg}	2338 ± 210^{fg}
SN-FR-014	Z2	3823 ± 306^{ab}	2299 ± 161^{hi}	1130 ± 90.0^{c}	79.0 ± 8.00^{h}	2161 ± 194^{c}	2394 ± 216^{f}
SN-FR-018	Z2	4264 ± 256^{a}	2802 ± 196^{a}	1115 ± 89.0^{cd}	88.0 ± 9.00^{f}	1887 ± 170^{g}	2912 ± 262^{a}
SN-FR-019	Z2	4425 ± 354^{a}	2533 ± 177^{e}	1173 ± 94.0^{ab}	115 ± 12.0^{a}	1942 ± 175^{f}	2653 ± 239^{c}
SN-FR-020	Z2	4334 ± 347^{a}	2610 ± 183^{d}	1123 ± 90.0^{c}	70.0 ± 7.00^{ij}	2278 ± 205^{a}	2821 ± 254^{ab}

SN-FR-048	Z2	4360 ± 262^{a}	2641 ± 185^{cd}	1148 ± 92.0^{b}	78.0 ± 8.00^{h}	2161 ± 194^{c}	2646 ± 238^{c}
SN-FR-049	Z2	3749 ± 300^{b}	2568 ± 180^{de}	1085 ± 87.0^{d}	97.0 ± 10.0^{d}	2083 ± 188^{d}	2730 ± 246^{bc}
SN-FR-050	Z2	4192 ± 335^{a}	2265 ± 159^{i}	1091 ± 87.0^{d}	97.0 ± 10.0^{d}	1895 ± 170^{g}	2422 ± 218^{ef}
SN-FR-051	Z2	4393 ± 351^{a}	2802 ± 196^{a}	998 ± 80.0^{g}	97.0 ± 10.0^{d}	1856 ± 167^{gh}	2618 ± 236^{cd}
SN-FR-052	Z2	4232 ± 254^{a}	2726 ± 191^{b}	1161 ± 93.0^{b}	97.0 ± 10.0^{d}	1910 ± 172^{fg}	2772 ± 250^{b}
SN-FR-054	Z2	4077 ± 326^{a}	2660 ± 186^{c}	976 ± 78.0^{gh}	87.0 ± 9.00^{f}	1848 ± 166^{gh}	2790 ± 195^{b}
SN-FR-055	Z2	4090 ± 327^{a}	2271 ± 159^{hi}	1173 ± 94.0^{ab}	108 ± 11.0^{b}	1989 ± 179^{ef}	2884 ± 260^{a}
SN-FR-056	Z2	4328 ± 260^{a}	2796 ± 196^{a}	1050 ± 84.0^{ef}	70.0 ± 7.00^{ij}	2036 ± 183^{e}	2856 ± 257^{a}
SN-FR-057	Z2	4473 ± 358^{a}	2587 ± 181^{d}	1008 ± 81.0^{fg}	104 ± 10.0^{c}	2247 ± 202^{ab}	2422 ± 218^{ef}
SN-FR-059	Z2	3743 ± 299^{b}	2706 ± 189^{bc}	1032 ± 83.0^{f}	102 ± 10.0^{c}	1989 ± 179^{ef}	2877 ± 259^{a}
SN-FR-064	Z2	4424 ± 265^{a}	2851 ± 200^{a}	933 ± 75.0^{i}	115 ± 12.0^{a}	1926 ± 173^{fg}	2905 ± 261^{a}
SN-FR-065	Z2	4200 ± 252^{a}	2315 ± 162^{h}	1032 ± 83.0^{f}	71.0 ± 7.00^{ij}	2318 ± 209^{a}	2394 ± 216^{f}
SN-FR-069	Z2	4232 ± 254^{a}	2380 ± 167^{g}	1150 ± 92.0^{b}	86.0 ± 9.00^{fg}	2271 ± 204^{a}	2793 ± 251^{b}
SN-FR-085	Z2	4264 ± 256^{a}	2545 ± 178^{e}	1204 ± 96.0^{a}	98.0 ± 10.0^{d}	2271 ± 204^{a}	2849 ± 256^{a}
SN-FR-086	Z2	3914 ± 313^{ab}	2802 ± 196^{a}	1096 ± 88.0^{d}	105 ± 10.0^{bc}	1793 ± 161^{hi}	2646 ± 238^{c}
SN-FR-087	Z2	4031 ± 322^{ab}	2602 ± 182^{d}	933 ± 75.0^{i}	102 ± 10.0^{c}	1817 ± 164^{h}	2457 ± 221^{e}
SN-FR-088	Z2	4360 ± 262^{a}	2246 ± 157^{i}	1054 ± 84.0^{e}	90.0 ± 9.00^{ef}	2208 ± 199^{b}	2779 ± 250^{b}
SN-FR-094	Z2	4392 ± 264^{a}	2722 ± 191^{bc}	985 ± 79.0^{gh}	79.0 ± 8.00^{h}	2200 ± 198^{b}	2681 ± 241^{c}
SN-FR-001	Z3	2929 ± 234^{c}	2454 ± 172^{f}	872 ± 70.0^{k}	95.0 ± 10.0^{de}	1614 ± 145^{k}	2193 ± 197^{h}
SN-FR-005	Z3	2782 ± 223^{cd}	1936 ± 136^{mn}	941 ± 75.0^{i}	70.0 ± 7.00^{ij}	1387 ± 125no	1386 ± 125^{pq}
SN-FR-006	Z3	4189 ± 335^{a}	2455 ± 172^{f}	896 ± 72.0^{j}	101 ± 10.0^{cd}	1464 ± 132^{mn}	1552 ± 140no
SN-FR-007	Z3	3286 ± 263^{bc}	2233 ± 156^{i}	753 ± 60.0^{no}	82.0 ± 8.00^{g}	1588 ± 143^{kl}	2446 ± 220^{ef}
SN-FR-008	Z3	3709 ± 297^{b}	2351 ± 165^{gh}	1103 ± 88.0^{cd}	71.0 ± 7.00^{ij}	1445 ± 130^{mn}	2353 ± 212^{fg}
SN-FR-032	Z3	3375 ± 270^{b}	2258 ± 158^{i}	1130 ± 90.0^{c}	73.0 ± 7.00^{i}	1601 ± 144^{k}	1971 ± 177^{jk}
SN-FR-033	Z3	2863 ± 229^{c}	2169 ± 152^{j}	1080 ± 86.0^{de}	75.0 ± 8.00^{hi}	1367 ± 123^{o}	2359 ± 212^{f}
SN-FR-034	Z3	3382 ± 271^{b}	2279 ± 160^{hi}	1074 ± 86.0^{de}	70.0 ± 7.00^{ij}	1646 ± 148^{jk}	1589 ± 143no
SN-FR-035	Z3	3992 ± 319^{ab}	2488 ± 174^{ef}	728 ± 58.0^{op}	98.0 ± 10.0^{d}	1102 ± 99.1^{s}	2156 ± 194^{hi}
SN-FR-036	Z3	3097 ± 248^{bc}	2422 ± 170^{fg}	981 ± 78.0^{gh}	102 ± 10.0^{c}	1056 ± 95.1st	1546 ± 139^{o}

SN-FR-037	Z3	3800 ± 304^{ab}	2567 ± 180^{de}	1064 ± 85.0^{e}	73.0 ± 7.00^{i}	1231 ± 111^{q}	1571 ± 141no
SN-FR-038	Z3	3668 ± 293^{b}	2447 ± 171^{f}	1146 ± 92.0^{bc}	82.0 ± 8.00^{g}	1283 ± 116^{p}	2224 ± 200^{gh}
SN-FR-039	Z3	4294 ± 344^{a}	2027 ± 142^{l}	741 ± 59.0^{o}	39.0 ± 4.00^{p}	1400 ± 126no	1756 ± 158^{lm}
SN-FR-040	Z3	3989 ± 319^{ab}	2407 ± 168^{g}	831 ± 66.0^{l}	65.0 ± 6.00^{jk}	1387 ± 125no	2033 ± 183^{ij}
SN-FR-041	Z3	4296 ± 344^{a}	2459 ± 172^{f}	726 ± 58.0^{op}	97.0 ± 10.0^{d}	1290 ± 116^{p}	1984 ± 178^{j}
SN-FR-058	Z3	4294 ± 344^{a}	2153 ± 151^{jk}	1040 ± 83.0^{ef}	45.0 ± 4.00^{no}	1536 ± 138^{l}	1916 ± 172^{k}
SN-FR-060	Z3	4054 ± 324^{ab}	2430 ± 170^{fg}	952 ± 76.0^{hi}	47.0 ± 5.00^{n}	1614 ± 145^{k}	1780 ± 160^{lm}
SN-FR-089	Z3	3742 ± 299^{b}	2060 ± 144^{kl}	927 ± 74.0^{i}	107 ± 11.0^{b}	1497 ± 135^{m}	2174 ± 196^{h}
SN-FR-090	Z3	4002 ± 320^{ab}	2451 ± 172^{f}	770 ± 62.0^{n}	99.0 ± 10.0^{d}	1076 ± 96.8^{s}	1799 ± 162^{l}
SN-FR-091	Z3	3861 ± 309^{ab}	2338 ± 164^{h}	924 ± 74.0^{ij}	94.0 ± 9.00^{e}	1542 ± 139^{l}	2131 ± 192^{hi}
SN-FR-002	Z4	2289 ± 183^{de}	1540 ± 108^{r}	752 ± 60.0^{o}	21.0 ± 2.00st	1500 ± 135^{m}	1623 ± 146^{n}
SN-FR-009	Z4	2452 ± 196^{d}	1511 ± 106^{rs}	864 ± 69.0^{k}	44.0 ± 4.00^{o}	1053 ± 94.8st	1383 ± 124^{pq}
SN-FR-010	Z4	2572 ± 206^{d}	2236 ± 157^{i}	613 ± 49.0^{s}	29.0 ± 3.00^{r}	1667 ± 150^{j}	1809 ± 163^{l}
SN-FR-011	Z4	2582 ± 207^{d}	2062 ± 144^{kl}	777 ± 62.0^{n}	44.0 ± 4.00^{o}	1167 ± 105^{r}	1523 ± 137°
SN-FR-012	Z4	2691 ± 215^{cd}	2303 ± 161^{h}	691 ± 55.0^{pq}	50.0 ± 5.00^{mn}	1474 ± 133^{m}	1796 ± 162^{l}
SN-FR-013	Z4	3165 ± 253^{bc}	1968 ± 138^{m}	857 ± 69.0^{kl}	58.0 ± 6.00^{l}	1641 ± 148^{jk}	1629 ± 147^{n}
SN-FR-015	Z4	2464 ± 197^{d}	1543 ± 108^{r}	643 ± 51.0^{r}	55.0 ± 6.00^{lm}	1167 ± 105^{r}	1350 ± 122^{q}
SN-FR-016	Z4	2085 ± 167^{e}	1506 ± 105^{rs}	726 ± 58.0^{op}	35.0 ± 4.00^{pq}	1132 ± 102^{rs}	1091 ± 98.2st
SN-FR-017	Z4	2141 ± 171^{e}	1679 ± 118^{q}	654 ± 52.0^{r}	55.0 ± 6.00^{lm}	1378 ± 124no	1390 ± 125^{pq}
SN-FR-021	Z4	2414 ± 193^{d}	2043 ± 143^{l}	695 ± 56.0^{pq}	54.0 ± 5.00^{m}	1395 ± 126no	1130 ± 102^{s}
SN-FR-024	Z4	2753 ± 220^{cd}	2248 ± 157^{i}	601 ± 48.0st	64.0 ± 6.00^{k}	1202 ± 108^{q}	1642 ± 148^{n}
SN-FR-025	Z4	2032 ± 163^{e}	1708 ± 120^{pq}	792 ± 63.0^{mn}	56.0 ± 6.00^{lm}	1053 ± 94.8st	1237 ± 111^{r}
SN-FR-028	Z4	2633 ± 211^{cd}	1755 ± 123^{p}	880 ± 70.0^{jk}	58.0 ± 6.00^{l}	1492 ± 134^{m}	1144 ± 103^{s}
SN-FR-030	Z4	2708 ± 217^{cd}	2228 ± 156^{ij}	790 ± 63.0^{mn}	50.0 ± 5.00^{mn}	1378 ± 124no	1277 ± 115^{qr}
SN-FR-031	Z4	2979 ± 238^{c}	1704 ± 119^{pq}	713 ± 57.0^{p}	47.0 ± 5.00^{n}	1053 ± 94.8st	1436 ± 129^{p}
SN-FR-070	Z4	2691 ± 215^{cd}	1747 ± 122^{p}	858 ± 69.0^{kl}	45.0 ± 4.00^{no}	1290 ± 116^{p}	1416 ± 128^{p}
SN-FR-071	Z4	2369 ± 190^{de}	1978 ± 138^{m}	785 ± 63.0^{mn}	56.0 ± 6.00^{lm}	1264 ± 114^{pq}	1742 ± 157^{lm}
SN-FR-072	Z4	2369 ± 190^{de}	2007 ± 140^{lm}	684 ± 55.0^{q}	30.0 ± 3.00^{qr}	1395 ± 126no	1337 ± 120^{q}

SN-FR-042	Z5	4360 ± 262^{a}	2681 ± 188^{c}	920 ± 74.0^{ij}	108 ± 11.0^{b}	1523 ± 137^{lm}	2434 ± 219^{ef}
SN-FR-043	Z5	3601 ± 288^{b}	2567 ± 180^{de}	916 ± 73.0^{ij}	64.0 ± 6.00^{k}	1242 ± 112^{pq}	2813 ± 253^{ab}
SN-FR-044	Z5	3672 ± 294^{b}	2453 ± 172^{f}	1017 ± 81.0^{fg}	69.0 ± 7.00^{j}	1678 ± 151^{j}	2846 ± 256^{a}
SN-FR-045	Z5	3637 ± 291^{b}	2594 ± 182^{d}	1081 ± 86.0^{de}	74.0 ± 7.00^{i}	1193 ± 107^{qr}	2673 ± 241^{c}
SN-FR-046	Z5	4232 ± 254^{a}	2678 ± 187^{c}	1148 ± 92.0^{b}	88.0 ± 9.00^{f}	1846 ± 166^{gh}	2534 ± 228^{de}
SN-FR-047	Z5	4000 ± 320^{ab}	1880 ± 132^{n}	1082 ± 87.0^{de}	54.0 ± 5.00^{m}	1390 ± 125no	2294 ± 206^{g}
SN-FR-066	Z5	2729 ± 218^{cd}	2250 ± 158^{i}	910 ± 73.0^{j}	106 ± 11.0^{bc}	1348 ± 121^{o}	2048 ± 184^{ij}
SN-FR-067	Z5	3201 ± 256^{bc}	2308 ± 162^{h}	772 ± 62.0^{n}	98.0 ± 10.0^{d}	1804 ± 162^{h}	2574 ± 232^{d}
SN-FR-068	Z5	4026 ± 322^{ab}	2175 ± 152^{j}	925 ± 74.0^{ij}	90.0 ± 9.00^{ef}	1720 ± 155^{ij}	2853 ± 257^{a}
SN-FR-073	Z5	3405 ± 272^{b}	2686 ± 188^{c}	1034 ± 83.0^{f}	77.0 ± 8.00^{h}	1250 ± 112^{pq}	1776 ± 160^{lm}
SN-FR-074	Z5	2826 ± 226^{c}	2145 ± 150^{jk}	1033 ± 83.0^{f}	71.0 ± 7.00^{ij}	1369 ± 123^{o}	2427 ± 218^{ef}
SN-FR-075	Z5	3355 ± 268^{b}	1835 ± 128^{o}	1109 ± 89.0^{cd}	64.0 ± 6.00^{k}	1257 ± 113^{pq}	1802 ± 162^{l}
SN-FR-076	Z5	3509 ± 281^{b}	2575 ± 180^{de}	767 ± 61.0^{n}	50.0 ± 5.00^{mn}	1481 ± 133^{m}	2780 ± 250^{b}
SN-FR-077	Z5	3983 ± 319^{ab}	2395 ± 168^{g}	1080 ± 86.0^{de}	54.0 ± 5.00^{m}	1130 ± 102^{rs}	2341 ± 211^{fg}
SN-FR-078	Z5	4389 ± 351^{a}	2180 ± 153^{j}	925 ± 74.0^{ij}	94.0 ± 9.00^{e}	1214 ± 109^{q}	2600 ± 234^{d}
SN-FR-079	Z5	3882 ± 311^{ab}	2221 ± 155^{ij}	1036 ± 83.0^{f}	74.0 ± 7.00^{i}	1446 ± 130^{mn}	2567 ± 231^{d}
SN-FR-080	Z5	3921 ± 314^{ab}	2353 ± 165^{gh}	1083 ± 87.0^{de}	55.0 ± 6.00^{lm}	1741 ± 157^{i}	2421 ± 218^{ef}
SN-FR-092	Z5	2839 ± 227^{c}	2266 ± 159^{i}	809 ± 65.0^{m}	81.0 ± 8.00^{gh}	1734 ± 156^{i}	1849 ± 166^{kl}
SN-FR-093	Z5	3551 ± 284^{b}	2485 ± 174^{ef}	1036 ± 83.0^{f}	87.0 ± 9.00^{f}	1425 ± 128^{n}	2640 ± 238^{cd}

Notes: Values are reported as mean ± analytical SD of three independently prepared analytical portions on a dry-weight basis (mg/kg DW). The three analytical measurements were used only to assess analytical precision and were averaged to obtain one site-level concentration per matrix and georeferenced sampling site for subsequent statistical analyses. Values <LOD and <LOQ are indicated as applicable.

Table S28. Individual concentrations of environmentally associated microelements and biologically relevant trace elements measured in georeferenced *Sambucus nigra* L. fruit samples collected across the investigated ecological sectors of the Oradea metropolitan area, Romania.

Sample ID	Zone	^{56}Fe (mg/kg)	^{55}Mn (mg/kg)	^{64}Zn (mg/kg)	^{63}Cu (mg/kg)	^{11}B (mg/kg)	^{98}Mo (mg/kg)	^{59}Co (mg/kg)	^{80}Se (mg/kg)
SN-FR-022	Z1	36 ± 3^{de}	16.9 ± 1.5^{de}	11.5 ± 0.7^{de}	4.93 ± 0.39^{de}	17.2 ± 0.9^{cd}	0.20 ± 0.02^{de}	0.047 ± 0.007^{de}	0.048 ± 0.005^{de}
SN-FR-023	Z1	36 ± 3^{de}	16.7 ± 1.6^{de}	11.2 ± 0.7^{de}	4.68 ± 0.36^{de}	18.4 ± 1.1^{bc}	0.19 ± 0.02^{de}	0.039 ± 0.005^{ef}	0.063 ± 0.009^{bc}

SN-FR-026	Z1	31 ± 2^{ef}	13.7 ± 1.0^{ef}	10.2 ± 0.7^{ef}	4.82 ± 0.34^{de}	18.2 ± 1.3^{bc}	0.14 ± 0.01^{f}	0.056 ± 0.008^{de}	0.029 ± 0.004^{f}
SN-FR-027	Z1	32 ± 3^{ef}	13.5 ± 1.2^{ef}	10.1 ± 0.8^{ef}	4.63 ± 0.37^{de}	16.1 ± 1.3^{de}	0.32 ± 0.04^{bc}	0.044 ± 0.005^{ef}	0.033 ± 0.005^{ef}
SN-FR-029	Z1	39 ± 3^{cd}	14.1 ± 1.1^{ef}	11.7 ± 1.0^{de}	4.59 ± 0.41^{de}	16.3 ± 0.9^{de}	0.26 ± 0.03^{cd}	0.052 ± 0.007^{de}	0.067 ± 0.008^{ab}
SN-FR-053	Z1	37 ± 3^{cd}	15.1 ± 1.3^{de}	10.0 ± 0.8^{ef}	4.51 ± 0.33^{de}	17.2 ± 1.4^{cd}	0.24 ± 0.03^{cd}	0.051 ± 0.006^{de}	0.066 ± 0.008^{bc}
SN-FR-061	Z1	39 ± 3^{cd}	15.3 ± 1.1^{de}	14.3 ± 1.1^{bc}	4.54 ± 0.31^{de}	18.9 ± 1.1^{bc}	0.21 ± 0.02^{de}	0.044 ± 0.006^{ef}	0.053 ± 0.006^{cd}
SN-FR-062	Z1	32 ± 2^{ef}	14.8 ± 1.2^{de}	13.8 ± 1.1^{bc}	4.49 ± 0.36^{de}	17.1 ± 1.3^{cd}	0.30 ± 0.03^{bc}	0.039 ± 0.004^{ef}	0.046 ± 0.007^{de}
SN-FR-063	Z1	37 ± 3^{cd}	13.3 ± 1.3^{ef}	11.7 ± 1.0^{de}	4.73 ± 0.35^{de}	18.3 ± 1.5^{bc}	0.13 ± 0.01^{f}	0.054 ± 0.008^{de}	0.047 ± 0.005^{de}
SN-FR-081	Z1	38 ± 3^{cd}	16.2 ± 1.6^{de}	10.1 ± 0.7^{ef}	4.64 ± 0.38^{de}	17.3 ± 1.3^{cd}	0.27 ± 0.03^{cd}	0.067 ± 0.010^{cd}	0.043 ± 0.005^{de}
SN-FR-082	Z1	38 ± 4^{cd}	17.0 ± 1.4^{de}	11.5 ± 0.9^{de}	5.35 ± 0.34^{cd}	18.6 ± 0.9^{bc}	0.21 ± 0.03^{de}	0.056 ± 0.006^{de}	0.074 ± 0.008^{ab}
SN-FR-083	Z1	35 ± 3^{de}	15.6 ± 1.3^{de}	10.4 ± 0.8^{ef}	5.07 ± 0.35^{cd}	16.6 ± 0.9^{de}	0.15 ± 0.01^{f}	0.059 ± 0.007^{cd}	0.054 ± 0.006^{cd}
SN-FR-084	Z1	39 ± 4^{cd}	19.1 ± 1.4^{bc}	8.3 ± 0.7^{f}	5.11 ± 0.32^{cd}	15.9 ± 1.0^{de}	0.25 ± 0.03^{cd}	0.073 ± 0.011^{bc}	0.052 ± 0.007^{cd}
SN-FR-003	Z2	41 ± 4^{bc}	23.1 ± 2.0^{ab}	13.6 ± 1.1^{bc}	6.35 ± 0.57^{ab}	16.3 ± 0.9^{de}	0.28 ± 0.04^{bc}	0.108 ± 0.015^{ab}	0.052 ± 0.008^{cd}
SN-FR-004	Z2	47 ± 4^{ab}	19.9 ± 1.5^{bc}	11.8 ± 0.9^{de}	5.90 ± 0.50^{bc}	19.2 ± 1.0^{bc}	0.37 ± 0.03^{ab}	0.082 ± 0.011^{bc}	0.054 ± 0.006^{cd}
SN-FR-014	Z2	41 ± 3^{bc}	20.9 ± 1.7^{bc}	11.9 ± 1.0^{de}	5.86 ± 0.36^{bc}	18.4 ± 1.4^{bc}	0.39 ± 0.05^{ab}	0.086 ± 0.013^{ab}	0.056 ± 0.008^{cd}
SN-FR-018	Z2	48 ± 5^{ab}	19.0 ± 1.5^{bc}	13.8 ± 1.1^{bc}	7.06 ± 0.48^{ab}	20.5 ± 1.6^{ab}	0.27 ± 0.02^{cd}	0.070 ± 0.007^{cd}	0.060 ± 0.007^{bc}
SN-FR-019	Z2	43 ± 4^{bc}	15.3 ± 1.5^{de}	15.9 ± 1.1^{ab}	6.20 ± 0.53^{ab}	18.6 ± 1.0^{bc}	0.35 ± 0.05^{ab}	0.094 ± 0.014^{ab}	0.079 ± 0.012^{ab}
SN-FR-020	Z2	48 ± 5^{ab}	19.1 ± 1.7^{bc}	16.2 ± 1.1^{ab}	6.05 ± 0.54^{bc}	17.8 ± 0.9^{cd}	0.28 ± 0.03^{bc}	0.075 ± 0.010^{bc}	0.041 ± 0.005^{de}
SN-FR-048	Z2	46 ± 3^{ab}	20.6 ± 2.0^{bc}	13.9 ± 1.2^{bc}	6.76 ± 0.44^{ab}	17.9 ± 1.4^{bc}	0.22 ± 0.02^{de}	0.084 ± 0.010^{bc}	0.051 ± 0.007^{cd}
SN-FR-049	Z2	47 ± 5^{ab}	14.7 ± 1.5^{de}	15.2 ± 1.0^{ab}	4.87 ± 0.40^{de}	21.7 ± 1.6^{ab}	0.33 ± 0.04^{ab}	0.053 ± 0.007^{de}	0.063 ± 0.006^{bc}
SN-FR-050	Z2	46 ± 4^{ab}	22.2 ± 1.9^{ab}	12.9 ± 1.1^{cd}	6.03 ± 0.53^{bc}	17.6 ± 1.3^{cd}	0.27 ± 0.03^{cd}	0.072 ± 0.008^{cd}	0.058 ± 0.007^{bc}
SN-FR-051	Z2	42 ± 3^{bc}	18.9 ± 1.7^{bc}	15.8 ± 1.0^{ab}	5.58 ± 0.36^{cd}	17.6 ± 1.2^{cd}	0.30 ± 0.03^{bc}	0.091 ± 0.010^{ab}	0.071 ± 0.008^{ab}
SN-FR-052	Z2	52 ± 5^{ab}	18.7 ± 1.6^{cd}	12.1 ± 0.8^{de}	5.27 ± 0.41^{cd}	17.7 ± 1.1^{cd}	0.26 ± 0.03^{cd}	0.066 ± 0.009^{cd}	0.055 ± 0.007^{cd}
SN-FR-054	Z2	39 ± 3^{cd}	20.3 ± 1.6^{bc}	16.5 ± 1.4^{ab}	5.99 ± 0.51^{bc}	18.9 ± 1.0^{bc}	0.26 ± 0.03^{cd}	0.085 ± 0.011^{ab}	0.048 ± 0.005^{de}
SN-FR-055	Z2	46 ± 4^{ab}	19.7 ± 1.9^{bc}	11.4 ± 0.9^{de}	5.66 ± 0.42^{bc}	19.0 ± 1.3^{bc}	0.30 ± 0.04^{bc}	0.075 ± 0.010^{bc}	0.048 ± 0.007^{cd}
SN-FR-056	Z2	42 ± 3^{bc}	22.4 ± 2.1^{ab}	14.7 ± 0.9^{bc}	5.72 ± 0.38^{bc}	16.0 ± 1.2^{de}	0.35 ± 0.03^{ab}	0.085 ± 0.012^{ab}	0.068 ± 0.010^{ab}
SN-FR-057	Z2	46 ± 4^{ab}	21.1 ± 1.7^{bc}	13.0 ± 1.2^{cd}	5.19 ± 0.34^{cd}	17.6 ± 1.3^{cd}	0.39 ± 0.05^{ab}	0.051 ± 0.007^{de}	0.064 ± 0.007^{bc}
SN-FR-059	Z2	46 ± 4^{ab}	16.2 ± 1.6^{de}	15.9 ± 1.2^{ab}	5.61 ± 0.34^{cd}	15.6 ± 1.1^{de}	0.29 ± 0.03^{bc}	0.082 ± 0.012^{bc}	0.079 ± 0.009^{ab}
SN-FR-064	Z2	44 ± 3^{bc}	18.3 ± 1.8^{cd}	13.8 ± 1.1^{bc}	6.58 ± 0.53^{ab}	17.0 ± 1.3^{cd}	0.31 ± 0.04^{bc}	0.059 ± 0.008^{cd}	0.083 ± 0.010^{ab}

SN-FR-065	Z2	48 ± 3[ab]	20.2 ± 1.7[bc]	13.2 ± 1.1[cd]	6.29 ± 0.46[ab]	19.8 ± 1.1[ab]	0.21 ± 0.02[de]	0.068 ± 0.008[cd]	0.065 ± 0.009[bc]
SN-FR-069	Z2	44 ± 3[bc]	21.8 ± 2.1[ab]	13.5 ± 0.9[cd]	5.82 ± 0.47[bc]	17.8 ± 1.4[bc]	0.28 ± 0.03[bc]	0.071 ± 0.009[cd]	0.067 ± 0.008[ab]
SN-FR-085	Z2	46 ± 5[ab]	17.7 ± 1.5[cd]	13.9 ± 0.9[bc]	5.09 ± 0.45[cd]	19.7 ± 1.1[ab]	0.31 ± 0.04[bc]	0.094 ± 0.011[ab]	0.054 ± 0.008[cd]
SN-FR-086	Z2	46 ± 3[ab]	18.4 ± 1.4[cd]	14.6 ± 1.0[bc]	6.01 ± 0.39[bc]	17.1 ± 0.9[cd]	0.29 ± 0.03[bc]	0.064 ± 0.008[cd]	0.047 ± 0.007[de]
SN-FR-087	Z2	44 ± 3[bc]	21.7 ± 2.2[ab]	15.3 ± 1.2[ab]	5.46 ± 0.34[cd]	17.4 ± 0.9[cd]	0.28 ± 0.03[bc]	0.073 ± 0.007[bc]	0.050 ± 0.007[cd]
SN-FR-088	Z2	46 ± 4[ab]	21.5 ± 2.0[ab]	13.3 ± 0.8[cd]	6.01 ± 0.38[bc]	20.0 ± 1.0[ab]	0.23 ± 0.03[cd]	0.103 ± 0.010[ab]	0.066 ± 0.009[ab]
SN-FR-094	Z2	40 ± 4[bc]	17.4 ± 1.6[cd]	11.8 ± 1.0[de]	5.95 ± 0.37[bc]	18.6 ± 1.4[bc]	0.41 ± 0.04[a]	0.080 ± 0.010[bc]	0.040 ± 0.006[de]
SN-FR-001	Z3	42 ± 4[bc]	19.8 ± 1.4[bc]	10.8 ± 0.8[de]	5.83 ± 0.51[bc]	16.5 ± 1.3[cd]	0.21 ± 0.02[de]	0.076 ± 0.008[bc]	0.037 ± 0.004[ef]
SN-FR-005	Z3	40 ± 3[cd]	17.3 ± 1.3[cd]	12.6 ± 0.8[cd]	4.98 ± 0.38[de]	14.6 ± 0.8[ef]	0.22 ± 0.03[de]	0.048 ± 0.007[de]	0.039 ± 0.006[de]
SN-FR-006	Z3	39 ± 3[cd]	13.6 ± 1.0[ef]	12.3 ± 1.1[cd]	5.36 ± 0.36[cd]	13.7 ± 1.0[f]	0.24 ± 0.02[cd]	0.071 ± 0.008[cd]	0.050 ± 0.007[cd]
SN-FR-007	Z3	41 ± 3[bc]	16.5 ± 1.6[de]	11.3 ± 0.9[de]	5.04 ± 0.41[cd]	17.6 ± 1.0[cd]	0.19 ± 0.02[de]	0.065 ± 0.007[cd]	0.041 ± 0.006[de]
SN-FR-008	Z3	40 ± 3[cd]	16.0 ± 1.2[de]	12.4 ± 0.8[cd]	5.26 ± 0.33[cd]	16.0 ± 0.8[de]	0.23 ± 0.03[cd]	0.064 ± 0.008[cd]	0.051 ± 0.005[cd]
SN-FR-032	Z3	41 ± 3[bc]	16.0 ± 1.5[de]	12.2 ± 1.1[cd]	4.98 ± 0.41[de]	15.4 ± 1.1[de]	0.12 ± 0.01[f]	0.065 ± 0.010[cd]	0.036 ± 0.005[ef]
SN-FR-033	Z3	39 ± 4[cd]	17.8 ± 1.5[cd]	12.7 ± 1.0[cd]	4.27 ± 0.35[ef]	15.9 ± 0.9[de]	0.12 ± 0.01[f]	0.061 ± 0.007[cd]	0.021 ± 0.002[f]
SN-FR-034	Z3	40 ± 4[bc]	19.6 ± 1.5[bc]	13.6 ± 1.0[bc]	4.78 ± 0.39[de]	17.3 ± 0.9[cd]	0.27 ± 0.03[cd]	0.064 ± 0.006[cd]	0.036 ± 0.004[ef]
SN-FR-035	Z3	42 ± 4[bc]	17.0 ± 1.5[cd]	13.0 ± 1.0[cd]	4.74 ± 0.33[de]	20.7 ± 1.3[ab]	0.16 ± 0.02[ef]	0.063 ± 0.006[cd]	0.039 ± 0.005[ef]
SN-FR-036	Z3	37 ± 3[cd]	16.5 ± 1.4[de]	11.0 ± 0.7[de]	5.15 ± 0.44[cd]	15.7 ± 1.2[de]	0.15 ± 0.02[f]	0.049 ± 0.007[de]	0.062 ± 0.009[bc]
SN-FR-037	Z3	32 ± 2[ef]	17.2 ± 1.7[cd]	15.3 ± 1.0[ab]	4.91 ± 0.33[de]	13.5 ± 1.0[f]	0.24 ± 0.03[cd]	0.057 ± 0.008[cd]	0.052 ± 0.006[cd]
SN-FR-038	Z3	42 ± 4[bc]	20.3 ± 1.8[bc]	13.0 ± 1.0[cd]	4.53 ± 0.30[de]	17.4 ± 1.3[cd]	0.27 ± 0.03[cd]	0.050 ± 0.007[de]	0.048 ± 0.005[de]
SN-FR-039	Z3	37 ± 3[cd]	18.2 ± 1.3[cd]	13.1 ± 1.0[cd]	4.52 ± 0.31[de]	15.5 ± 1.0[de]	0.21 ± 0.03[de]	0.068 ± 0.009[cd]	0.037 ± 0.004[ef]
SN-FR-040	Z3	38 ± 3[cd]	19.3 ± 1.7[bc]	8.9 ± 0.8[f]	4.06 ± 0.32[ef]	15.5 ± 1.2[de]	0.24 ± 0.03[cd]	0.055 ± 0.007[de]	0.052 ± 0.007[cd]
SN-FR-041	Z3	36 ± 3[de]	15.9 ± 1.3[de]	11.8 ± 0.9[de]	4.28 ± 0.28[ef]	15.2 ± 1.2[ef]	0.18 ± 0.02[ef]	0.054 ± 0.006[de]	0.046 ± 0.006[de]
SN-FR-058	Z3	41 ± 3[bc]	14.0 ± 1.1[ef]	13.4 ± 1.1[bc]	4.32 ± 0.30[ef]	15.9 ± 1.0[de]	0.21 ± 0.02[de]	0.037 ± 0.004[ef]	0.070 ± 0.008[ab]
SN-FR-060	Z3	38 ± 4[cd]	11.6 ± 0.8[f]	11.8 ± 0.8[de]	4.82 ± 0.33[de]	17.4 ± 1.2[cd]	0.28 ± 0.03[bc]	0.072 ± 0.011[bc]	0.036 ± 0.005[ef]
SN-FR-089	Z3	45 ± 4[ab]	18.9 ± 1.5[cd]	11.8 ± 1.0[de]	5.43 ± 0.43[cd]	17.9 ± 1.0[cd]	0.26 ± 0.03[cd]	0.047 ± 0.005[de]	0.039 ± 0.005[ef]
SN-FR-090	Z3	39 ± 3[cd]	16.5 ± 1.3[de]	14.2 ± 1.2[bc]	4.70 ± 0.41[de]	16.9 ± 1.3[cd]	0.18 ± 0.02[ef]	0.050 ± 0.005[de]	0.052 ± 0.006[cd]
SN-FR-091	Z3	34 ± 3[de]	15.7 ± 1.4[de]	11.1 ± 0.7[de]	4.88 ± 0.31[de]	16.0 ± 0.8[de]	0.24 ± 0.03[cd]	0.072 ± 0.010[bc]	0.041 ± 0.005[de]
SN-FR-002	Z4	31 ± 2[ef]	20.3 ± 2.0[bc]	10.2 ± 0.6[ef]	4.15 ± 0.29[ef]	16.3 ± 0.9[de]	0.17 ± 0.02[ef]	0.036 ± 0.005[ef]	0.036 ± 0.005[ef]

SN-FR-009	Z4	30 ± 2^{ef}	16.8 ± 1.5^{de}	9.2 ± 0.6^{f}	4.86 ± 0.39^{de}	17.6 ± 1.0^{cd}	0.21 ± 0.02^{de}	0.030 ± 0.004^{f}	0.031 ± 0.005^{ef}
SN-FR-010	Z4	31 ± 2^{ef}	18.3 ± 1.8^{cd}	12.0 ± 0.8^{de}	4.08 ± 0.28^{ef}	20.5 ± 1.1^{ab}	0.19 ± 0.02^{de}	0.039 ± 0.005^{ef}	0.029 ± 0.003^{f}
SN-FR-011	Z4	28 ± 2^{f}	15.9 ± 1.5^{de}	12.0 ± 0.9^{de}	3.76 ± 0.31^{f}	18.9 ± 1.5^{bc}	0.25 ± 0.03^{cd}	0.048 ± 0.005^{de}	0.037 ± 0.004^{ef}
SN-FR-012	Z4	33 ± 3^{de}	20.5 ± 1.6^{bc}	10.5 ± 0.9^{ef}	4.99 ± 0.31^{de}	20.0 ± 1.2^{ab}	0.26 ± 0.03^{cd}	0.052 ± 0.006^{de}	0.046 ± 0.005^{de}
SN-FR-013	Z4	28 ± 2^{f}	18.8 ± 1.6^{cd}	11.8 ± 0.8^{de}	4.50 ± 0.29^{e}	16.9 ± 1.0^{cd}	0.25 ± 0.03^{cd}	0.057 ± 0.006^{de}	0.038 ± 0.004^{ef}
SN-FR-015	Z4	34 ± 3^{de}	15.7 ± 1.2^{de}	9.4 ± 0.8^{ef}	3.62 ± 0.22^{f}	17.6 ± 1.2^{cd}	0.23 ± 0.03^{cd}	0.048 ± 0.006^{de}	0.032 ± 0.005^{ef}
SN-FR-016	Z4	31 ± 3^{ef}	19.2 ± 1.6^{bc}	13.3 ± 1.1^{cd}	4.39 ± 0.36^{ef}	18.5 ± 1.2^{bc}	0.21 ± 0.02^{de}	0.037 ± 0.004^{ef}	0.048 ± 0.007^{cd}
SN-FR-017	Z4	30 ± 2^{ef}	16.5 ± 1.4^{de}	10.7 ± 0.8^{de}	3.74 ± 0.23^{f}	17.9 ± 1.2^{bc}	0.12 ± 0.02^{f}	0.043 ± 0.006^{ef}	0.034 ± 0.005^{ef}
SN-FR-021	Z4	28 ± 3^{f}	16.6 ± 1.4^{de}	10.1 ± 0.7^{ef}	4.01 ± 0.34^{ef}	18.2 ± 0.9^{bc}	0.20 ± 0.02^{de}	0.048 ± 0.005^{de}	0.036 ± 0.004^{ef}
SN-FR-024	Z4	34 ± 3^{de}	17.0 ± 1.6^{cd}	9.7 ± 0.9^{ef}	3.64 ± 0.28^{f}	17.0 ± 1.0^{cd}	0.14 ± 0.01^{f}	0.042 ± 0.006^{ef}	0.039 ± 0.004^{e}
SN-FR-025	Z4	32 ± 2^{ef}	18.2 ± 1.4^{cd}	11.6 ± 0.8^{de}	4.38 ± 0.39^{ef}	19.4 ± 1.3^{ab}	0.21 ± 0.03^{de}	0.053 ± 0.006^{de}	0.057 ± 0.008^{bc}
SN-FR-028	Z4	33 ± 3^{de}	13.0 ± 1.3^{ef}	9.5 ± 0.7^{ef}	4.13 ± 0.31^{ef}	16.4 ± 0.8^{de}	0.17 ± 0.02^{ef}	0.031 ± 0.004^{f}	0.047 ± 0.005^{de}
SN-FR-030	Z4	37 ± 3^{cd}	14.7 ± 1.3^{de}	11.9 ± 0.7^{de}	3.57 ± 0.24^{f}	17.9 ± 1.0^{cd}	0.20 ± 0.02^{de}	0.057 ± 0.006^{de}	0.044 ± 0.006^{de}
SN-FR-031	Z4	33 ± 3^{de}	18.0 ± 1.5^{cd}	10.7 ± 0.8^{de}	4.24 ± 0.33^{ef}	17.1 ± 1.0^{cd}	0.18 ± 0.02^{ef}	0.029 ± 0.004^{f}	0.035 ± 0.005^{ef}
SN-FR-070	Z4	32 ± 3^{ef}	15.2 ± 1.1^{de}	9.3 ± 0.6^{f}	3.87 ± 0.23^{f}	17.5 ± 1.0^{cd}	0.21 ± 0.02^{de}	0.037 ± 0.005^{ef}	0.037 ± 0.005^{ef}
SN-FR-071	Z4	34 ± 2^{de}	15.9 ± 1.4^{de}	11.1 ± 0.8^{de}	4.72 ± 0.32^{de}	19.5 ± 1.5^{ab}	0.25 ± 0.02^{cd}	0.058 ± 0.008^{cd}	0.047 ± 0.005^{de}
SN-FR-072	Z4	34 ± 2^{de}	17.7 ± 1.6^{cd}	12.3 ± 1.1^{cd}	4.13 ± 0.33^{ef}	16.7 ± 1.2^{cd}	0.19 ± 0.02^{de}	0.052 ± 0.007^{de}	0.052 ± 0.006^{cd}
SN-FR-042	Z5	39 ± 3^{cd}	16.9 ± 1.3^{de}	11.9 ± 0.8^{de}	5.27 ± 0.33^{cd}	15.7 ± 1.1^{de}	0.25 ± 0.02^{cd}	0.058 ± 0.008^{cd}	0.073 ± 0.009^{ab}
SN-FR-043	Z5	39 ± 4^{cd}	16.5 ± 1.4^{de}	14.1 ± 0.9^{bc}	4.99 ± 0.39^{de}	15.8 ± 1.0^{de}	0.25 ± 0.03^{cd}	0.046 ± 0.005^{e}	0.047 ± 0.006^{de}
SN-FR-044	Z5	37 ± 3^{cd}	13.3 ± 1.1^{ef}	13.5 ± 0.9^{cd}	5.54 ± 0.44^{cd}	18.9 ± 1.0^{bc}	0.31 ± 0.03^{bc}	0.059 ± 0.008^{cd}	0.053 ± 0.008^{cd}
SN-FR-045	Z5	41 ± 3^{bc}	14.2 ± 1.2^{ef}	12.8 ± 0.9^{cd}	5.69 ± 0.35^{bc}	16.0 ± 1.1^{de}	0.25 ± 0.03^{cd}	0.074 ± 0.011^{bc}	0.049 ± 0.005^{de}
SN-FR-046	Z5	40 ± 4^{bc}	18.6 ± 1.3^{cd}	12.1 ± 0.9^{cd}	5.82 ± 0.47^{bc}	16.9 ± 1.0^{cd}	0.32 ± 0.03^{bc}	0.054 ± 0.006^{de}	0.069 ± 0.009^{ab}
SN-FR-047	Z5	43 ± 4^{bc}	18.5 ± 1.7^{cd}	13.2 ± 1.0^{cd}	5.27 ± 0.34^{cd}	17.3 ± 1.3^{cd}	0.19 ± 0.02^{de}	0.073 ± 0.010^{bc}	0.059 ± 0.008^{bc}
SN-FR-066	Z5	39 ± 4^{cd}	15.7 ± 1.6^{de}	11.3 ± 0.8^{de}	5.97 ± 0.50^{bc}	18.0 ± 1.1^{bc}	0.21 ± 0.03^{de}	0.061 ± 0.006^{cd}	0.046 ± 0.007^{de}
SN-FR-067	Z5	44 ± 4^{bc}	16.5 ± 1.4^{de}	14.4 ± 1.2^{bc}	5.53 ± 0.34^{cd}	19.5 ± 1.4^{ab}	0.31 ± 0.04^{bc}	0.081 ± 0.008^{bc}	0.057 ± 0.007^{cd}
SN-FR-068	Z5	41 ± 3^{bc}	13.5 ± 1.1^{ef}	13.8 ± 0.9^{bc}	4.99 ± 0.45^{cd}	16.6 ± 0.9^{de}	0.25 ± 0.03^{cd}	0.044 ± 0.005^{ef}	0.042 ± 0.005^{de}
SN-FR-073	Z5	42 ± 3^{bc}	15.9 ± 1.6^{de}	17.0 ± 1.2^{ab}	4.89 ± 0.41^{de}	17.1 ± 1.0^{cd}	0.29 ± 0.04^{bc}	0.042 ± 0.005^{ef}	0.052 ± 0.007^{cd}
SN-FR-074	Z5	42 ± 3^{bc}	19.9 ± 1.5^{bc}	13.7 ± 0.9^{bc}	5.17 ± 0.41^{cd}	16.9 ± 1.3^{cd}	0.23 ± 0.02^{d}	0.068 ± 0.010^{cd}	0.044 ± 0.005^{de}

SN-FR-075	Z5	42 ± 4^{bc}	24.7 ± 2.2^{ab}	13.8 ± 1.1^{bc}	5.37 ± 0.40^{cd}	19.0 ± 1.5^{ab}	0.30 ± 0.03^{bc}	0.052 ± 0.007^{de}	0.040 ± 0.006^{de}
SN-FR-076	Z5	40 ± 3^{cd}	19.2 ± 1.7^{bc}	14.5 ± 1.3^{bc}	5.14 ± 0.34^{cd}	17.8 ± 1.1^{cd}	0.24 ± 0.02^{cd}	0.072 ± 0.009^{bc}	0.054 ± 0.006^{cd}
SN-FR-077	Z5	42 ± 4^{bc}	16.6 ± 1.6^{de}	12.0 ± 0.8^{de}	5.19 ± 0.46^{cd}	15.4 ± 1.1^{de}	0.29 ± 0.03^{bc}	0.074 ± 0.010^{bc}	0.053 ± 0.005^{cd}
SN-FR-078	Z5	39 ± 3^{cd}	19.7 ± 1.8^{bc}	11.0 ± 0.9^{de}	4.88 ± 0.38^{de}	17.5 ± 1.1^{cd}	0.27 ± 0.04^{bc}	0.064 ± 0.007^{cd}	0.038 ± 0.004^{ef}
SN-FR-079	Z5	45 ± 4^{ab}	20.1 ± 1.7^{bc}	11.2 ± 0.8^{de}	4.97 ± 0.44^{cd}	15.6 ± 1.2^{de}	0.19 ± 0.02^{de}	0.081 ± 0.009^{bc}	0.065 ± 0.008^{bc}
SN-FR-080	Z5	40 ± 4^{bc}	19.2 ± 1.5^{bc}	13.8 ± 1.2^{bc}	5.38 ± 0.40^{cd}	15.8 ± 1.2^{de}	0.34 ± 0.03^{ab}	0.062 ± 0.008^{cd}	0.045 ± 0.005^{de}
SN-FR-092	Z5	35 ± 3^{de}	17.6 ± 1.3^{cd}	11.3 ± 0.9^{de}	4.67 ± 0.37^{de}	20.0 ± 1.1^{ab}	0.22 ± 0.03^{de}	0.049 ± 0.007^{de}	0.038 ± 0.005^{ef}
SN-FR-093	Z5	39 ± 3^{cd}	17.9 ± 1.3^{cd}	13.8 ± 1.1^{bc}	4.74 ± 0.40^{de}	17.5 ± 1.3^{cd}	0.27 ± 0.03^{cd}	0.082 ± 0.010^{bc}	0.047 ± 0.006^{de}

Notes: Values are reported as mean ± analytical SD of three independently prepared analytical portions on a dry-weight basis (mg/kg DW). The three analytical measurements were used only to assess analytical precision and were averaged to obtain one site-level concentration per matrix and georeferenced sampling site for subsequent statistical analyses. Values <LOD and <LOQ are indicated as applicable.

Table S29. Individual concentrations of lithogenic and geochemically associated elements in *Sambucus nigra* L. fruit samples collected at georeferenced sampling points across the ecological sectors of the Oradea metropolitan area, Romania.

Sample ID	Zone	^{27}Al (mg/kg)	^{48}Ti (mg/kg)	^{51}V (mg/kg)	^{137}Ba (mg/kg)	^{88}Sr (mg/kg)	^{7}Li (mg/kg)	^{85}Rb (mg/kg)	^{133}Cs (mg/kg)
SN-FR-022	Z1	10.2 ± 1.0^{ab}	0.61 ± 0.06^{bc}	0.21 ± 0.02^{cd}	6.5 ± 0.5^{de}	4.3 ± 0.4^{de}	0.12 ± 0.01^{ef}	0.79 ± 0.08^{cd}	0.015 ± 0.002^{cd}
SN-FR-023	Z1	11.2 ± 1.1^{ab}	0.69 ± 0.05^{ab}	0.19 ± 0.02^{de}	7.5 ± 0.7^{bc}	5.4 ± 0.5^{ab}	0.18 ± 0.02^{bc}	0.93 ± 0.07^{bc}	0.018 ± 0.002^{bc}
SN-FR-026	Z1	8.9 ± 0.8^{cd}	0.56 ± 0.06^{cd}	0.16 ± 0.02^{ef}	6.2 ± 0.5^{de}	4.4 ± 0.4^{de}	0.13 ± 0.01^{de}	0.77 ± 0.07^{de}	0.011 ± 0.001^{f}
SN-FR-027	Z1	9.1 ± 0.9^{cd}	0.54 ± 0.05^{cd}	0.18 ± 0.02^{de}	5.4 ± 0.5^{ef}	4.0 ± 0.3^{ef}	0.13 ± 0.01^{de}	0.60 ± 0.05^{f}	0.011 ± 0.001^{f}
SN-FR-029	Z1	10.2 ± 1.0^{ab}	0.60 ± 0.05^{bc}	0.24 ± 0.02^{ab}	7.6 ± 0.5^{bc}	4.8 ± 0.3^{cd}	0.13 ± 0.02^{de}	0.86 ± 0.08^{bc}	0.016 ± 0.002^{bc}
SN-FR-053	Z1	8.2 ± 0.6^{de}	0.47 ± 0.04^{ef}	0.19 ± 0.02^{de}	6.9 ± 0.5^{cd}	4.7 ± 0.5^{cd}	0.16 ± 0.01^{cd}	0.73 ± 0.07^{de}	0.013 ± 0.002^{de}
SN-FR-061	Z1	5.2 ± 0.4^{f}	0.39 ± 0.03^{f}	0.13 ± 0.01^{f}	5.3 ± 0.5^{ef}	4.2 ± 0.3^{ef}	0.13 ± 0.01^{de}	0.56 ± 0.06^{f}	0.011 ± 0.001^{f}
SN-FR-062	Z1	5.1 ± 0.5^{f}	0.33 ± 0.03^{f}	0.13 ± 0.01^{f}	5.7 ± 0.5^{ef}	4.2 ± 0.4^{de}	0.11 ± 0.01^{f}	0.63 ± 0.06^{f}	0.011 ± 0.001^{f}
SN-FR-063	Z1	5.8 ± 0.6^{f}	0.43 ± 0.04^{ef}	0.20 ± 0.02^{cd}	5.5 ± 0.5^{ef}	3.9 ± 0.3^{ef}	0.14 ± 0.01^{de}	0.70 ± 0.07^{ef}	0.015 ± 0.002^{cd}
SN-FR-081	Z1	7.4 ± 0.7^{ef}	0.52 ± 0.04^{de}	0.15 ± 0.01^{ef}	5.4 ± 0.5^{ef}	4.9 ± 0.4^{cd}	0.16 ± 0.02^{bc}	0.68 ± 0.07^{ef}	0.013 ± 0.001^{ef}
SN-FR-082	Z1	9.9 ± 0.9^{ab}	0.58 ± 0.04^{cd}	0.15 ± 0.01^{ef}	6.6 ± 0.5^{cd}	4.7 ± 0.5^{cd}	0.14 ± 0.02^{cd}	0.84 ± 0.09^{bc}	0.015 ± 0.002^{cd}
SN-FR-083	Z1	10.6 ± 1.1^{ab}	0.46 ± 0.04^{ef}	0.22 ± 0.02^{bc}	6.0 ± 0.5^{de}	4.5 ± 0.4^{de}	0.16 ± 0.02^{bc}	0.77 ± 0.08^{de}	0.013 ± 0.002^{de}
SN-FR-084	Z1	6.8 ± 0.7^{ef}	0.59 ± 0.05^{bc}	0.23 ± 0.02^{bc}	6.1 ± 0.5^{de}	4.5 ± 0.4^{de}	0.13 ± 0.01^{de}	0.92 ± 0.09^{bc}	0.012 ± 0.001^{ef}

SN-FR-003	Z2	10.8 ± 0.9^{ab}	0.78 ± 0.07^{a}	0.24 ± 0.02^{ab}	9.7 ± 1.0^{a}	6.2 ± 0.5^{a}	0.17 ± 0.01^{bc}	1.10 ± 0.12^{ab}	0.019 ± 0.002^{ab}
SN-FR-004	Z2	10.6 ± 0.8^{ab}	0.72 ± 0.07^{ab}	0.26 ± 0.02^{ab}	9.8 ± 1.0^{a}	5.2 ± 0.5^{bc}	0.19 ± 0.02^{ab}	1.06 ± 0.10^{ab}	0.022 ± 0.002^{ab}
SN-FR-014	Z2	10.5 ± 0.8^{ab}	0.55 ± 0.05^{cd}	0.22 ± 0.02^{bc}	7.8 ± 0.8^{ab}	6.1 ± 0.4^{a}	0.17 ± 0.02^{bc}	1.15 ± 0.09^{a}	0.019 ± 0.002^{ab}
SN-FR-018	Z2	9.7 ± 0.9^{bc}	0.63 ± 0.06^{bc}	0.23 ± 0.02^{bc}	8.9 ± 0.8^{ab}	5.9 ± 0.5^{ab}	0.23 ± 0.02^{a}	1.06 ± 0.10^{ab}	0.017 ± 0.002^{bc}
SN-FR-019	Z2	10.1 ± 0.8^{ab}	0.62 ± 0.06^{bc}	0.30 ± 0.02^{a}	8.1 ± 0.6^{ab}	5.4 ± 0.6^{ab}	0.18 ± 0.02^{bc}	1.07 ± 0.10^{ab}	0.023 ± 0.002^{a}
SN-FR-020	Z2	11.1 ± 1.2^{ab}	0.86 ± 0.08^{a}	0.24 ± 0.02^{ab}	9.4 ± 0.7^{a}	5.1 ± 0.5^{bc}	0.20 ± 0.02^{ab}	1.15 ± 0.12^{ab}	0.016 ± 0.002^{bc}
SN-FR-048	Z2	9.9 ± 0.7^{ab}	0.75 ± 0.05^{ab}	0.27 ± 0.02^{ab}	8.8 ± 0.6^{ab}	5.4 ± 0.6^{ab}	0.20 ± 0.02^{ab}	0.89 ± 0.08^{bc}	0.020 ± 0.002^{ab}
SN-FR-049	Z2	11.6 ± 1.2^{ab}	0.88 ± 0.09^{a}	0.31 ± 0.03^{a}	8.3 ± 0.9^{ab}	5.6 ± 0.5^{ab}	0.21 ± 0.02^{ab}	1.00 ± 0.11^{ab}	0.018 ± 0.002^{bc}
SN-FR-050	Z2	12.7 ± 1.3^{a}	0.67 ± 0.07^{ab}	0.26 ± 0.02^{ab}	9.0 ± 0.8^{ab}	6.4 ± 0.6^{a}	0.16 ± 0.01^{cd}	1.21 ± 0.13^{a}	0.023 ± 0.002^{a}
SN-FR-051	Z2	10.7 ± 1.0^{ab}	0.60 ± 0.05^{bc}	0.28 ± 0.03^{ab}	7.8 ± 0.8^{ab}	6.6 ± 0.7^{a}	0.19 ± 0.02^{ab}	1.02 ± 0.10^{ab}	0.017 ± 0.002^{bc}
SN-FR-052	Z2	11.0 ± 1.1^{ab}	0.86 ± 0.09^{a}	0.23 ± 0.02^{bc}	7.9 ± 0.6^{ab}	5.6 ± 0.5^{ab}	0.18 ± 0.02^{bc}	0.85 ± 0.07^{cd}	0.021 ± 0.003^{ab}
SN-FR-054	Z2	10.5 ± 1.0^{ab}	0.72 ± 0.06^{ab}	0.24 ± 0.02^{ab}	8.1 ± 0.8^{ab}	5.0 ± 0.5^{bc}	0.24 ± 0.02^{a}	1.02 ± 0.10^{ab}	0.023 ± 0.003^{a}
SN-FR-055	Z2	11.4 ± 0.9^{ab}	0.66 ± 0.06^{ab}	0.31 ± 0.03^{a}	7.4 ± 0.6^{bc}	5.4 ± 0.5^{ab}	0.21 ± 0.02^{ab}	1.11 ± 0.12^{ab}	0.018 ± 0.002^{bc}
SN-FR-056	Z2	9.9 ± 1.0^{ab}	0.71 ± 0.07^{ab}	0.29 ± 0.03^{a}	8.1 ± 0.8^{ab}	5.5 ± 0.5^{ab}	0.17 ± 0.02^{bc}	1.04 ± 0.09^{ab}	0.015 ± 0.002^{cd}
SN-FR-057	Z2	10.6 ± 0.8^{ab}	0.87 ± 0.08^{a}	0.29 ± 0.02^{a}	8.3 ± 0.7^{ab}	5.0 ± 0.4^{bc}	0.22 ± 0.02^{a}	1.16 ± 0.09^{a}	0.019 ± 0.002^{ab}
SN-FR-059	Z2	10.9 ± 0.8^{ab}	0.70 ± 0.06^{ab}	0.27 ± 0.02^{ab}	9.6 ± 0.8^{a}	5.5 ± 0.5^{ab}	0.20 ± 0.02^{ab}	1.03 ± 0.09^{ab}	0.020 ± 0.002^{ab}
SN-FR-064	Z2	10.6 ± 0.9^{ab}	0.64 ± 0.05^{bc}	0.25 ± 0.02^{ab}	8.2 ± 0.6^{ab}	5.5 ± 0.6^{ab}	0.23 ± 0.03^{a}	0.82 ± 0.08^{cd}	0.018 ± 0.002^{bc}
SN-FR-065	Z2	10.2 ± 0.8^{ab}	0.79 ± 0.08^{a}	0.28 ± 0.03^{ab}	8.1 ± 0.8^{ab}	5.2 ± 0.5^{bc}	0.21 ± 0.03^{ab}	0.96 ± 0.10^{ab}	0.019 ± 0.002^{ab}
SN-FR-069	Z2	11.1 ± 1.1^{ab}	0.72 ± 0.06^{ab}	0.29 ± 0.02^{a}	8.3 ± 0.7^{ab}	5.7 ± 0.5^{ab}	0.20 ± 0.02^{ab}	1.02 ± 0.10^{ab}	0.021 ± 0.003^{ab}
SN-FR-085	Z2	9.4 ± 0.9^{bc}	0.84 ± 0.07^{a}	0.28 ± 0.03^{ab}	8.3 ± 0.6^{ab}	6.9 ± 0.6^{a}	0.20 ± 0.02^{ab}	0.92 ± 0.10^{bc}	0.019 ± 0.002^{ab}
SN-FR-086	Z2	11.6 ± 1.0^{a}	0.61 ± 0.05^{bc}	0.23 ± 0.02^{bc}	8.1 ± 0.8^{ab}	5.6 ± 0.4^{ab}	0.20 ± 0.02^{ab}	0.94 ± 0.08^{bc}	0.022 ± 0.002^{ab}
SN-FR-087	Z2	9.9 ± 0.9^{ab}	0.77 ± 0.07^{ab}	0.24 ± 0.02^{ab}	8.8 ± 0.6^{ab}	6.2 ± 0.5^{a}	0.20 ± 0.02^{ab}	1.06 ± 0.08^{ab}	0.022 ± 0.002^{ab}
SN-FR-088	Z2	8.9 ± 0.7^{cd}	0.90 ± 0.07^{a}	0.30 ± 0.03^{a}	9.3 ± 0.8^{a}	5.6 ± 0.4^{ab}	0.20 ± 0.02^{ab}	0.95 ± 0.08^{bc}	0.022 ± 0.002^{ab}
SN-FR-094	Z2	10.1 ± 0.8^{ab}	0.69 ± 0.06^{ab}	0.22 ± 0.02^{bc}	7.1 ± 0.7^{bc}	5.7 ± 0.5^{ab}	0.20 ± 0.02^{ab}	0.90 ± 0.07^{bc}	0.018 ± 0.002^{bc}
SN-FR-001	Z3	11.6 ± 1.2^{ab}	0.59 ± 0.04^{bc}	0.18 ± 0.01^{de}	6.9 ± 0.6^{cd}	5.2 ± 0.5^{bc}	0.18 ± 0.02^{bc}	0.90 ± 0.09^{bc}	0.015 ± 0.002^{cd}
SN-FR-005	Z3	10.7 ± 0.8^{ab}	0.72 ± 0.06^{ab}	0.26 ± 0.02^{ab}	7.5 ± 0.8^{bc}	4.6 ± 0.4^{cd}	0.17 ± 0.02^{bc}	0.92 ± 0.10^{bc}	0.017 ± 0.002^{bc}
SN-FR-006	Z3	10.4 ± 0.8^{ab}	0.67 ± 0.07^{ab}	0.23 ± 0.02^{bc}	7.1 ± 0.6^{cd}	5.6 ± 0.5^{ab}	0.18 ± 0.02^{bc}	1.08 ± 0.09^{ab}	0.016 ± 0.002^{bc}
SN-FR-007	Z3	8.0 ± 0.7^{de}	0.56 ± 0.04^{cd}	0.19 ± 0.02^{de}	6.4 ± 0.7^{de}	5.1 ± 0.5^{bc}	0.14 ± 0.01^{de}	0.84 ± 0.09^{bc}	0.018 ± 0.002^{bc}

SN-FR-008	Z3	9.3 ± 0.9[bc]	0.59 ± 0.05[bc]	0.25 ± 0.02[ab]	7.7 ± 0.7[ab]	5.9 ± 0.6[ab]	0.20 ± 0.02[ab]	0.79 ± 0.07[cd]	0.022 ± 0.003[ab]
SN-FR-032	Z3	9.3 ± 0.7[bc]	0.58 ± 0.04[cd]	0.23 ± 0.02[bc]	7.9 ± 0.7[ab]	5.2 ± 0.5[bc]	0.16 ± 0.02[bc]	1.00 ± 0.08[ab]	0.016 ± 0.002[bc]
SN-FR-033	Z3	9.7 ± 0.8[bc]	0.63 ± 0.06[bc]	0.21 ± 0.02[cd]	8.6 ± 0.6[ab]	4.7 ± 0.4[cd]	0.17 ± 0.02[bc]	0.78 ± 0.07[de]	0.018 ± 0.002[bc]
SN-FR-034	Z3	10.4 ± 0.7[ab]	0.62 ± 0.05[bc]	0.22 ± 0.02[bc]	6.6 ± 0.7[cd]	5.6 ± 0.4[ab]	0.15 ± 0.02[cd]	0.90 ± 0.08[bc]	0.020 ± 0.002[ab]
SN-FR-035	Z3	8.3 ± 0.6[de]	0.58 ± 0.06[cd]	0.25 ± 0.02[ab]	6.6 ± 0.6[cd]	5.6 ± 0.5[ab]	0.20 ± 0.02[ab]	1.12 ± 0.11[ab]	0.018 ± 0.002[bc]
SN-FR-036	Z3	9.3 ± 0.9[bc]	0.61 ± 0.04[bc]	0.26 ± 0.02[ab]	8.0 ± 0.6[ab]	5.5 ± 0.5[ab]	0.14 ± 0.02[cd]	0.83 ± 0.08[cd]	0.017 ± 0.002[bc]
SN-FR-037	Z3	9.6 ± 0.9[bc]	0.63 ± 0.05[bc]	0.19 ± 0.02[de]	7.7 ± 0.7[ab]	5.8 ± 0.4[ab]	0.17 ± 0.02[bc]	1.06 ± 0.10[ab]	0.017 ± 0.002[bc]
SN-FR-038	Z3	10.2 ± 0.8[ab]	0.51 ± 0.05[de]	0.18 ± 0.02[de]	6.5 ± 0.6[cd]	6.0 ± 0.6[ab]	0.16 ± 0.02[bc]	0.78 ± 0.07[de]	0.017 ± 0.002[bc]
SN-FR-039	Z3	7.7 ± 0.6[de]	0.65 ± 0.05[bc]	0.19 ± 0.02[de]	6.5 ± 0.7[cd]	5.1 ± 0.4[bc]	0.17 ± 0.02[bc]	0.77 ± 0.07[de]	0.014 ± 0.002[cd]
SN-FR-040	Z3	9.1 ± 0.7[cd]	0.65 ± 0.06[ab]	0.23 ± 0.02[bc]	7.4 ± 0.6[bc]	4.8 ± 0.4[cd]	0.14 ± 0.02[cd]	1.08 ± 0.11[ab]	0.020 ± 0.002[ab]
SN-FR-041	Z3	9.0 ± 0.9[cd]	0.46 ± 0.04[ef]	0.25 ± 0.02[ab]	8.4 ± 0.7[ab]	5.6 ± 0.6[ab]	0.15 ± 0.02[cd]	1.02 ± 0.08[ab]	0.016 ± 0.002[bc]
SN-FR-058	Z3	8.0 ± 0.7[de]	0.48 ± 0.05[ef]	0.22 ± 0.02[bc]	8.5 ± 0.8[ab]	5.0 ± 0.4[bc]	0.16 ± 0.02[bc]	1.03 ± 0.11[ab]	0.015 ± 0.002[cd]
SN-FR-060	Z3	10.8 ± 1.0[ab]	0.60 ± 0.06[bc]	0.25 ± 0.02[ab]	6.4 ± 0.5[de]	5.3 ± 0.5[bc]	0.20 ± 0.02[ab]	0.83 ± 0.08[cd]	0.022 ± 0.002[ab]
SN-FR-089	Z3	10.5 ± 1.1[ab]	0.66 ± 0.05[ab]	0.26 ± 0.03[ab]	7.8 ± 0.6[ab]	5.4 ± 0.4[ab]	0.21 ± 0.02[ab]	0.81 ± 0.07[cd]	0.019 ± 0.002[ab]
SN-FR-090	Z3	8.3 ± 0.7[de]	0.73 ± 0.07[ab]	0.23 ± 0.02[bc]	7.3 ± 0.5[bc]	6.2 ± 0.6[a]	0.18 ± 0.02[bc]	0.82 ± 0.07[cd]	0.020 ± 0.002[ab]
SN-FR-091	Z3	9.2 ± 0.9[bc]	0.66 ± 0.07[ab]	0.26 ± 0.03[ab]	7.7 ± 0.8[ab]	5.7 ± 0.6[ab]	0.16 ± 0.02[bc]	1.07 ± 0.10[ab]	0.016 ± 0.002[bc]
SN-FR-002	Z4	5.8 ± 0.5[f]	0.41 ± 0.03[ef]	0.14 ± 0.01[f]	4.3 ± 0.4[f]	3.7 ± 0.3[ef]	0.10 ± 0.01[f]	0.77 ± 0.08[de]	0.013 ± 0.001[ef]
SN-FR-009	Z4	6.4 ± 0.5[ef]	0.38 ± 0.03[f]	0.13 ± 0.01[f]	5.0 ± 0.5[ef]	3.6 ± 0.3[f]	0.12 ± 0.01[ef]	0.65 ± 0.06[ef]	0.008 ± 0.001[f]
SN-FR-010	Z4	7.1 ± 0.7[ef]	0.37 ± 0.03[f]	0.17 ± 0.01[e]	6.4 ± 0.6[de]	4.6 ± 0.4[cd]	0.11 ± 0.01[f]	0.65 ± 0.06[ef]	0.013 ± 0.001[ef]
SN-FR-011	Z4	6.7 ± 0.6[ef]	0.44 ± 0.03[ef]	0.18 ± 0.02[de]	5.3 ± 0.4[ef]	3.9 ± 0.4[ef]	0.12 ± 0.01[ef]	0.71 ± 0.07[ef]	0.012 ± 0.002[ef]
SN-FR-012	Z4	5.7 ± 0.6[f]	0.37 ± 0.03[f]	0.17 ± 0.02[de]	6.3 ± 0.6[de]	3.3 ± 0.3[f]	0.13 ± 0.01[de]	0.76 ± 0.08[de]	0.012 ± 0.002[ef]
SN-FR-013	Z4	5.1 ± 0.4[f]	0.37 ± 0.03[f]	0.17 ± 0.02[de]	4.7 ± 0.4[f]	3.8 ± 0.4[ef]	0.13 ± 0.01[de]	0.84 ± 0.09[bc]	0.012 ± 0.001[ef]
SN-FR-015	Z4	6.5 ± 0.5[ef]	0.40 ± 0.03[f]	0.12 ± 0.01[f]	5.4 ± 0.4[ef]	4.8 ± 0.3[cd]	0.14 ± 0.01[de]	0.84 ± 0.07[cd]	0.015 ± 0.002[cd]
SN-FR-016	Z4	7.5 ± 0.7[ef]	0.46 ± 0.05[ef]	0.15 ± 0.01[ef]	4.5 ± 0.4[f]	3.4 ± 0.2[f]	0.11 ± 0.01[f]	0.62 ± 0.06[f]	0.014 ± 0.001[de]
SN-FR-017	Z4	7.4 ± 0.7[ef]	0.33 ± 0.02[f]	0.17 ± 0.02[de]	6.0 ± 0.6[de]	4.4 ± 0.4[de]	0.11 ± 0.01[f]	0.63 ± 0.06[f]	0.010 ± 0.001[f]
SN-FR-021	Z4	5.8 ± 0.6[f]	0.29 ± 0.03[f]	0.14 ± 0.01[f]	5.4 ± 0.5[ef]	3.9 ± 0.3[ef]	0.14 ± 0.01[de]	0.71 ± 0.07[ef]	0.015 ± 0.002[cd]
SN-FR-024	Z4	6.2 ± 0.6[f]	0.38 ± 0.04[f]	0.14 ± 0.01[f]	4.4 ± 0.3[f]	4.0 ± 0.4[ef]	0.11 ± 0.01[f]	0.87 ± 0.08[bc]	0.010 ± 0.001[f]
SN-FR-025	Z4	5.3 ± 0.5[f]	0.44 ± 0.03[ef]	0.17 ± 0.02[de]	6.0 ± 0.6[de]	3.2 ± 0.3[f]	0.14 ± 0.02[cd]	0.63 ± 0.05[f]	0.010 ± 0.001[f]

SN-FR-028	Z4	6.8 ± 0.6[ef]	0.44 ± 0.04[ef]	0.12 ± 0.01[f]	5.7 ± 0.5[ef]	3.8 ± 0.3[ef]	0.13 ± 0.01[de]	0.74 ± 0.08[de]	0.013 ± 0.002[de]
SN-FR-030	Z4	7.5 ± 0.7[ef]	0.36 ± 0.03[f]	0.13 ± 0.01[f]	5.3 ± 0.4[ef]	4.7 ± 0.4[cd]	0.12 ± 0.01[ef]	0.66 ± 0.07[ef]	0.013 ± 0.001[ef]
SN-FR-031	Z4	8.1 ± 0.8[de]	0.37 ± 0.03[f]	0.15 ± 0.01[ef]	4.5 ± 0.4[f]	3.6 ± 0.4[ef]	0.13 ± 0.01[de]	0.71 ± 0.08[ef]	0.014 ± 0.001[de]
SN-FR-070	Z4	7.4 ± 0.7[ef]	0.34 ± 0.03[f]	0.15 ± 0.01[ef]	4.7 ± 0.4[f]	3.1 ± 0.3[f]	0.14 ± 0.02[cd]	0.70 ± 0.06[ef]	0.012 ± 0.001[ef]
SN-FR-071	Z4	6.1 ± 0.6[f]	0.44 ± 0.04[ef]	0.20 ± 0.02[cd]	4.9 ± 0.4[f]	3.4 ± 0.2[f]	0.12 ± 0.01[ef]	0.76 ± 0.09[de]	0.010 ± 0.001[f]
SN-FR-072	Z4	6.9 ± 0.7[ef]	0.42 ± 0.04[ef]	0.13 ± 0.01[f]	5.0 ± 0.4[f]	3.9 ± 0.4[ef]	0.11 ± 0.01[f]	0.54 ± 0.04[f]	0.010 ± 0.001[f]
SN-FR-042	Z5	8.7 ± 0.7[cd]	0.56 ± 0.06[cd]	0.22 ± 0.02[bc]	6.9 ± 0.7[cd]	3.8 ± 0.4[ef]	0.20 ± 0.02[ab]	0.81 ± 0.08[cd]	0.014 ± 0.002[cd]
SN-FR-043	Z5	9.2 ± 0.7[cd]	0.57 ± 0.05[cd]	0.23 ± 0.02[bc]	5.9 ± 0.5[ef]	5.4 ± 0.4[ab]	0.15 ± 0.02[cd]	0.82 ± 0.09[cd]	0.013 ± 0.001[ef]
SN-FR-044	Z5	7.4 ± 0.8[ef]	0.63 ± 0.05[bc]	0.25 ± 0.03[ab]	7.3 ± 0.7[bc]	5.1 ± 0.4[bc]	0.17 ± 0.02[bc]	0.71 ± 0.05[ef]	0.017 ± 0.002[bc]
SN-FR-045	Z5	9.0 ± 0.7[cd]	0.48 ± 0.05[ef]	0.18 ± 0.02[de]	7.8 ± 0.8[ab]	4.6 ± 0.4[cd]	0.18 ± 0.02[bc]	0.84 ± 0.06[cd]	0.014 ± 0.002[cd]
SN-FR-046	Z5	9.4 ± 0.9[bc]	0.57 ± 0.04[cd]	0.23 ± 0.02[bc]	7.7 ± 0.7[ab]	5.6 ± 0.5[ab]	0.15 ± 0.02[cd]	0.95 ± 0.09[ab]	0.013 ± 0.002[de]
SN-FR-047	Z5	9.6 ± 1.0[bc]	0.64 ± 0.05[bc]	0.19 ± 0.02[de]	7.1 ± 0.6[cd]	4.3 ± 0.4[de]	0.17 ± 0.02[bc]	0.80 ± 0.08[cd]	0.014 ± 0.002[cd]
SN-FR-066	Z5	8.5 ± 0.7[cd]	0.46 ± 0.04[ef]	0.20 ± 0.02[cd]	6.5 ± 0.6[cd]	4.2 ± 0.3[ef]	0.14 ± 0.02[cd]	0.87 ± 0.09[bc]	0.014 ± 0.002[cd]
SN-FR-067	Z5	9.7 ± 0.7[bc]	0.61 ± 0.05[bc]	0.21 ± 0.02[cd]	7.5 ± 0.6[bc]	4.9 ± 0.4[cd]	0.15 ± 0.02[cd]	0.83 ± 0.08[cd]	0.018 ± 0.002[bc]
SN-FR-068	Z5	6.9 ± 0.6[ef]	0.61 ± 0.06[bc]	0.21 ± 0.02[cd]	6.9 ± 0.7[cd]	5.1 ± 0.5[bc]	0.20 ± 0.02[ab]	1.00 ± 0.11[ab]	0.016 ± 0.002[bc]
SN-FR-073	Z5	8.1 ± 0.8[de]	0.59 ± 0.05[bc]	0.16 ± 0.01[ef]	6.7 ± 0.6[cd]	5.0 ± 0.5[bc]	0.14 ± 0.02[cd]	0.79 ± 0.08[cd]	0.014 ± 0.001[de]
SN-FR-074	Z5	9.2 ± 0.7[cd]	0.62 ± 0.04[bc]	0.22 ± 0.02[bc]	6.8 ± 0.5[cd]	4.4 ± 0.4[de]	0.16 ± 0.01[cd]	0.94 ± 0.11[ab]	0.013 ± 0.002[de]
SN-FR-075	Z5	7.8 ± 0.6[de]	0.71 ± 0.05[ab]	0.23 ± 0.02[bc]	7.9 ± 0.8[ab]	5.0 ± 0.4[bc]	0.15 ± 0.02[cd]	0.79 ± 0.06[cd]	0.018 ± 0.002[bc]
SN-FR-076	Z5	9.2 ± 0.9[bc]	0.54 ± 0.05[cd]	0.21 ± 0.02[cd]	7.1 ± 0.5[cd]	4.9 ± 0.4[cd]	0.16 ± 0.02[bc]	0.83 ± 0.08[cd]	0.018 ± 0.002[bc]
SN-FR-077	Z5	8.4 ± 0.8[cd]	0.61 ± 0.04[bc]	0.18 ± 0.02[de]	7.6 ± 0.7[bc]	4.5 ± 0.4[de]	0.15 ± 0.02[cd]	0.90 ± 0.08[bc]	0.017 ± 0.002[bc]
SN-FR-078	Z5	8.9 ± 0.7[cd]	0.53 ± 0.05[de]	0.25 ± 0.02[ab]	7.3 ± 0.7[bc]	4.7 ± 0.4[cd]	0.18 ± 0.02[bc]	0.67 ± 0.05[ef]	0.020 ± 0.002[ab]
SN-FR-079	Z5	9.8 ± 0.8[bc]	0.54 ± 0.04[de]	0.22 ± 0.02[bc]	5.8 ± 0.5[ef]	4.8 ± 0.5[cd]	0.15 ± 0.02[cd]	0.81 ± 0.06[cd]	0.016 ± 0.002[bc]
SN-FR-080	Z5	9.4 ± 0.9[bc]	0.55 ± 0.04[cd]	0.19 ± 0.02[de]	6.9 ± 0.6[cd]	4.8 ± 0.4[cd]	0.15 ± 0.01[cd]	0.83 ± 0.08[cd]	0.017 ± 0.002[bc]
SN-FR-092	Z5	8.7 ± 0.8[cd]	0.56 ± 0.05[cd]	0.24 ± 0.02[ab]	6.5 ± 0.5[de]	4.6 ± 0.3[de]	0.14 ± 0.02[cd]	0.84 ± 0.06[cd]	0.015 ± 0.002[cd]
SN-FR-093	Z5	9.1 ± 0.9[cd]	0.45 ± 0.03[ef]	0.24 ± 0.02[ab]	7.0 ± 0.7[cd]	4.3 ± 0.4[de]	0.15 ± 0.02[cd]	0.72 ± 0.08[de]	0.015 ± 0.002[cd]

Notes: Values are reported as mean ± analytical SD of three independently prepared analytical portions on a dry-weight basis (mg/kg DW). The three analytical measurements were used only to assess analytical precision and were averaged to obtain one site-level concentration per matrix and georeferenced sampling site for subsequent statistical analyses. Values <LOD and <LOQ are indicated as applicable.

Table S30. Individual concentrations of potentially toxic and environmentally relevant trace elements measured in georeferenced *Sambucus nigra* L. fruit samples collected across the investigated ecological sectors of the Oradea metropolitan area, Romania.

Sample ID	Zone	^{208}Pb (mg/kg)	^{111}Cd (mg/kg)	^{75}As (mg/kg)	^{202}Hg (mg/kg)	^{52}Cr (mg/kg)	^{60}Ni (mg/kg)	^{121}Sb (mg/kg)	^{118}Sn (mg/kg)
SN-FR-022	Z1	<LOD	<LOD	<LOD	<LOD	0.09 ± 0.01^{ij}	0.09 ± 0.01^{e}	<LOD	<LOD
SN-FR-023	Z1	<LOD	<LOD	<LOD	<LOD	0.09 ± 0.01^{ij}	0.08 ± 0.01^{ef}	<LOD	<LOD
SN-FR-026	Z1	<LOD	<LOD	<LOD	<LOD	0.12 ± 0.01^{hij}	0.08 ± 0.01^{ef}	<LOD	0.004 ± 0.001^{ef}
SN-FR-027	Z1	<LOD	<LOD	<LOD	<LOD	0.14 ± 0.02^{ghi}	0.08 ± 0.01^{ef}	<LOD	0.004 ± 0.001^{ef}
SN-FR-029	Z1	<LOD	<LOD	<LOD	<LOD	0.12 ± 0.01^{hij}	0.09 ± 0.01^{e}	<LOD	<LOD
SN-FR-053	Z1	<LOD	<LOD	<LOD	<LOD	0.08 ± 0.01^{ij}	0.07 ± 0.01^{ef}	<LOD	<LOD
SN-FR-061	Z1	<LOD	<LOD	<LOD	<LOD	0.09 ± 0.01^{ij}	0.10 ± 0.01^{de}	<LOD	<LOD
SN-FR-062	Z1	<LOD	<LOD	<LOD	<LOD	0.10 ± 0.01^{hij}	0.07 ± 0.01^{ef}	<LOD	<LOD
SN-FR-063	Z1	<LOD	<LOD	<LOD	<LOD	0.10 ± 0.01^{hij}	0.10 ± 0.01^{de}	<LOD	0.004 ± 0.001^{ef}
SN-FR-081	Z1	<LOD	<LOD	<LOD	<LOD	0.09 ± 0.01^{ij}	0.08 ± 0.01^{ef}	<LOD	0.004 ± 0.001^{ef}
SN-FR-082	Z1	<LOD	<LOD	<LOD	<LOD	0.07 ± 0.01^{j}	0.06 ± 0.01^{f}	<LOD	<LOD
SN-FR-083	Z1	<LOD	<LOD	<LOD	<LOD	0.09 ± 0.01^{ij}	0.08 ± 0.01^{ef}	<LOD	<LOD
SN-FR-084	Z1	<LOD	<LOD	<LOD	<LOD	0.08 ± 0.01^{ij}	0.08 ± 0.01^{ef}	<LOD	<LOD
SN-FR-003	Z2	0.086 ± 0.011^{ab}	0.0072 ± 0.0010^{de}	<LOD	<LOD	0.20 ± 0.02^{ef}	0.22 ± 0.02^{b}	0.0047 ± 0.0005^{ab}	0.011 ± 0.001^{ab}
SN-FR-004	Z2	<LOD	<LOD	0.010 ± 0.001^{ab}	<LOD	0.31 ± 0.03^{bc}	0.22 ± 0.03^{ab}	<LOD	0.011 ± 0.001^{ab}
SN-FR-014	Z2	0.076 ± 0.010^{bc}	0.0079 ± 0.0011^{bc}	0.014 ± 0.002^{a}	<LOD	0.29 ± 0.03^{bc}	0.25 ± 0.03^{ab}	0.0046 ± 0.0005^{ab}	0.005 ± 0.001^{de}
SN-FR-018	Z2	0.094 ± 0.012^{ab}	<LOD	0.008 ± 0.001^{de}	<LOD	0.20 ± 0.02^{ef}	0.30 ± 0.03^{a}	<LOD	0.008 ± 0.001^{bc}
SN-FR-019	Z2	0.037 ± 0.005^{f}	0.0077 ± 0.0012^{bc}	0.011 ± 0.001^{ab}	<LOD	0.33 ± 0.03^{b}	0.36 ± 0.04^{a}	<LOD	0.005 ± 0.001^{de}
SN-FR-020	Z2	0.060 ± 0.006^{cd}	0.0066 ± 0.0008^{de}	0.009 ± 0.001^{cd}	<LOD	0.23 ± 0.02^{de}	0.23 ± 0.03^{ab}	0.0034 ± 0.0005^{de}	0.008 ± 0.001^{bc}
SN-FR-048	Z2	0.090 ± 0.011^{ab}	0.0081 ± 0.0009^{ab}	0.010 ± 0.001^{ab}	<LOD	0.22 ± 0.02^{de}	0.21 ± 0.02^{bc}	0.0036 ± 0.0006^{cd}	0.011 ± 0.002^{ab}
SN-FR-049	Z2	0.077 ± 0.009^{bc}	<LOD	0.010 ± 0.001^{ab}	<LOD	0.21 ± 0.02^{def}	0.22 ± 0.02^{b}	0.0058 ± 0.0010^{a}	0.011 ± 0.001^{ab}
SN-FR-050	Z2	0.093 ± 0.009^{ab}	0.0085 ± 0.0013^{ab}	<LOD	<LOD	0.20 ± 0.02^{ef}	0.29 ± 0.03^{a}	0.0059 ± 0.0008^{a}	0.010 ± 0.001^{ab}
SN-FR-051	Z2	<LOD	0.0075 ± 0.0011^{cd}	0.009 ± 0.001^{cd}	<LOD	0.22 ± 0.02^{de}	0.15 ± 0.01^{cd}	0.0031 ± 0.0004^{ef}	0.011 ± 0.001^{ab}
SN-FR-052	Z2	0.070 ± 0.007^{bc}	0.0089 ± 0.0010^{ab}	0.009 ± 0.001^{cd}	<LOD	0.30 ± 0.03^{bc}	0.20 ± 0.02^{bc}	0.0045 ± 0.0007^{ab}	0.009 ± 0.001^{b}
SN-FR-054	Z2	0.086 ± 0.010^{ab}	0.0051 ± 0.0006^{f}	<LOD	<LOD	0.22 ± 0.03^{de}	0.27 ± 0.02^{a}	0.0035 ± 0.0004^{de}	0.012 ± 0.002^{ab}

SN-FR-055	Z2	<LOD	<LOD	<LOD	<LOD	0.32 ± 0.03^{bc}	0.35 ± 0.04^{a}	<LOD	0.004 ± 0.001^{ef}
SN-FR-056	Z2	0.080 ± 0.009^{ab}	<LOD	0.009 ± 0.001^{cd}	<LOD	0.30 ± 0.03^{bc}	0.31 ± 0.04^{a}	0.0042 ± 0.0005^{bc}	0.014 ± 0.002^{a}
SN-FR-057	Z2	0.097 ± 0.010^{ab}	0.0077 ± 0.0010^{bc}	<LOD	<LOD	0.20 ± 0.02^{ef}	0.22 ± 0.03^{ab}	0.0037 ± 0.0005^{cd}	0.011 ± 0.002^{ab}
SN-FR-059	Z2	0.064 ± 0.008^{cd}	0.0083 ± 0.0011^{ab}	0.010 ± 0.001^{ab}	<LOD	0.29 ± 0.03^{bc}	0.29 ± 0.03^{a}	<LOD	0.010 ± 0.001^{ab}
SN-FR-064	Z2	0.076 ± 0.008^{bc}	<LOD	<LOD	<LOD	0.46 ± 0.04^{a}	0.31 ± 0.04^{a}	0.0043 ± 0.0007^{ab}	0.014 ± 0.002^{a}
SN-FR-065	Z2	0.105 ± 0.014^{a}	0.0076 ± 0.0011^{cd}	0.010 ± 0.001^{ab}	<LOD	0.26 ± 0.03^{cd}	0.17 ± 0.02^{cd}	<LOD	0.016 ± 0.002^{a}
SN-FR-069	Z2	0.076 ± 0.007^{bc}	0.0090 ± 0.0011^{ab}	0.009 ± 0.001^{cd}	<LOD	0.20 ± 0.02^{ef}	0.31 ± 0.04^{a}	0.0041 ± 0.0005^{bc}	0.008 ± 0.001^{bc}
SN-FR-085	Z2	0.071 ± 0.007^{bc}	<LOD	<LOD	<LOD	0.27 ± 0.03^{bcd}	0.32 ± 0.03^{a}	<LOD	0.012 ± 0.001^{a}
SN-FR-086	Z2	0.090 ± 0.010^{ab}	<LOD	0.007 ± 0.001^{ef}	<LOD	0.22 ± 0.02^{de}	0.36 ± 0.03^{a}	<LOD	0.009 ± 0.001^{b}
SN-FR-087	Z2	0.074 ± 0.010^{bc}	0.0104 ± 0.0011^{a}	0.012 ± 0.002^{ab}	<LOD	0.32 ± 0.03^{bc}	0.31 ± 0.03^{a}	0.0032 ± 0.0004^{ef}	0.009 ± 0.001^{b}
SN-FR-088	Z2	0.052 ± 0.006^{de}	0.0073 ± 0.0011^{cd}	0.008 ± 0.001^{de}	<LOD	0.19 ± 0.02^{efg}	0.32 ± 0.04^{a}	0.0037 ± 0.0005^{cd}	0.013 ± 0.001^{a}
SN-FR-094	Z2	0.084 ± 0.010^{ab}	0.0071 ± 0.0010^{de}	0.014 ± 0.002^{a}	<LOD	0.33 ± 0.03^{b}	0.41 ± 0.04^{a}	0.0034 ± 0.0005^{de}	0.004 ± 0.001^{ef}
SN-FR-001	Z3	<LOD	<LOD	<LOD	<LOD	0.15 ± 0.01^{fgh}	0.15 ± 0.02^{cd}	<LOD	0.007 ± 0.001^{bc}
SN-FR-005	Z3	<LOD	<LOD	<LOD	<LOD	0.21 ± 0.02^{def}	0.12 ± 0.01^{d}	<LOD	0.006 ± 0.001^{cd}
SN-FR-006	Z3	0.040 ± 0.004^{ef}	<LOD	<LOD	<LOD	0.13 ± 0.01^{ghi}	0.14 ± 0.01^{d}	<LOD	0.006 ± 0.001^{cd}
SN-FR-007	Z3	<LOD	<LOD	<LOD	<LOD	0.16 ± 0.02^{fgh}	0.11 ± 0.01^{de}	<LOD	0.008 ± 0.001^{bc}
SN-FR-008	Z3	<LOD	<LOD	<LOD	<LOD	0.17 ± 0.01^{efg}	0.21 ± 0.02^{bc}	<LOD	0.007 ± 0.001^{bc}
SN-FR-032	Z3	<LOD	<LOD	<LOD	<LOD	0.10 ± 0.01^{hij}	0.09 ± 0.01^{e}	<LOD	0.005 ± 0.001^{de}
SN-FR-033	Z3	<LOD	<LOD	<LOD	<LOD	0.09 ± 0.01^{ij}	0.11 ± 0.01^{de}	<LOD	0.004 ± 0.001^{ef}
SN-FR-034	Z3	<LOD	0.0052 ± 0.0008^{f}	<LOD	<LOD	0.10 ± 0.01^{hij}	0.10 ± 0.01^{de}	<LOD	<LOD
SN-FR-035	Z3	<LOD	0.0051 ± 0.0005^{f}	<LOD	<LOD	0.18 ± 0.02^{efg}	0.13 ± 0.01^{d}	<LOD	0.007 ± 0.001^{bc}
SN-FR-036	Z3	<LOD	<LOD	<LOD	<LOD	0.13 ± 0.01^{ghi}	0.16 ± 0.02^{cd}	<LOD	<LOD
SN-FR-037	Z3	0.043 ± 0.004^{ef}	<LOD	<LOD	<LOD	0.12 ± 0.01^{hij}	0.17 ± 0.01^{c}	<LOD	<LOD
SN-FR-038	Z3	<LOD	<LOD	<LOD	<LOD	0.14 ± 0.02^{ghi}	0.20 ± 0.02^{bc}	<LOD	0.007 ± 0.001^{bc}
SN-FR-039	Z3	0.036 ± 0.003^{f}	<LOD	<LOD	<LOD	0.17 ± 0.02^{efg}	0.13 ± 0.01^{d}	<LOD	<LOD
SN-FR-040	Z3	<LOD	<LOD	<LOD	<LOD	0.13 ± 0.01^{ghi}	0.17 ± 0.02^{cd}	<LOD	0.004 ± 0.001^{ef}
SN-FR-041	Z3	<LOD	<LOD	<LOD	<LOD	0.15 ± 0.02^{fgh}	0.11 ± 0.01^{de}	<LOD	0.006 ± 0.001^{cd}
SN-FR-058	Z3	0.035 ± 0.003^{f}	<LOD	<LOD	<LOD	0.16 ± 0.02^{fgh}	0.17 ± 0.02^{cd}	<LOD	0.006 ± 0.001^{cd}

SN-FR-060	Z3	<LOD	<LOD	<LOD	<LOD	0.23 ± 0.02^{de}	0.15 ± 0.01^{cd}	<LOD	0.007 ± 0.001^{bc}
SN-FR-089	Z3	0.042 ± 0.006^{ef}	<LOD	<LOD	<LOD	0.10 ± 0.01^{hij}	0.12 ± 0.01^{d}	<LOD	0.005 ± 0.001^{de}
SN-FR-090	Z3	<LOD	<LOD	<LOD	<LOD	0.13 ± 0.01^{ghi}	0.18 ± 0.02^{bc}	<LOD	0.007 ± 0.001^{bc}
SN-FR-091	Z3	<LOD	<LOD	<LOD	<LOD	0.14 ± 0.01^{ghi}	0.23 ± 0.02^{ab}	<LOD	0.004 ± 0.001^{ef}
SN-FR-002	Z4	<LOD	<LOD	<LOD	<LOD	0.06 ± 0.01^{j}	0.07 ± 0.01^{ef}	<LOD	<LOD
SN-FR-009	Z4	<LOD	<LOD	<LOD	<LOD	0.07 ± 0.01^{j}	0.06 ± 0.01^{f}	<LOD	<LOD
SN-FR-010	Z4	<LOD	<LOD	<LOD	<LOD	0.06 ± 0.01^{j}	0.07 ± 0.01^{ef}	<LOD	<LOD
SN-FR-011	Z4	<LOD	<LOD	<LOD	<LOD	0.07 ± 0.01^{j}	<LOD	<LOD	<LOD
SN-FR-012	Z4	<LOD	<LOD	<LOD	<LOD	0.06 ± 0.01^{j}	0.08 ± 0.01^{ef}	<LOD	<LOD
SN-FR-013	Z4	<LOD	<LOD	<LOD	<LOD	<LOD	0.08 ± 0.01^{ef}	<LOD	<LOD
SN-FR-015	Z4	<LOD	<LOD	<LOD	<LOD	0.07 ± 0.01^{j}	0.08 ± 0.01^{ef}	<LOD	<LOD
SN-FR-016	Z4	<LOD	<LOD	<LOD	<LOD	0.06 ± 0.01^{j}	0.06 ± 0.01^{f}	<LOD	<LOD
SN-FR-017	Z4	<LOD	<LOD	<LOD	<LOD	0.06 ± 0.01^{j}	<LOD	<LOD	<LOD
SN-FR-021	Z4	<LOD	<LOD	<LOD	<LOD	0.07 ± 0.01^{j}	0.08 ± 0.01^{ef}	<LOD	<LOD
SN-FR-024	Z4	<LOD	<LOD	<LOD	<LOD	0.07 ± 0.01^{j}	0.07 ± 0.01^{ef}	<LOD	<LOD
SN-FR-025	Z4	<LOD	<LOD	<LOD	<LOD	0.07 ± 0.01^{j}	<LOD	<LOD	<LOD
SN-FR-028	Z4	<LOD	<LOD	<LOD	<LOD	0.07 ± 0.01^{j}	<LOD	<LOD	<LOD
SN-FR-030	Z4	<LOD	<LOD	<LOD	<LOD	0.07 ± 0.01^{j}	0.07 ± 0.01^{ef}	<LOD	<LOD
SN-FR-031	Z4	<LOD	<LOD	<LOD	<LOD	0.08 ± 0.01^{ij}	0.07 ± 0.01^{ef}	<LOD	<LOD
SN-FR-070	Z4	<LOD	<LOD	<LOD	<LOD	<LOD	<LOD	<LOD	<LOD
SN-FR-071	Z4	<LOD	<LOD	<LOD	<LOD	0.06 ± 0.01^{j}	0.06 ± 0.01^{f}	<LOD	<LOD
SN-FR-072	Z4	<LOD	<LOD	<LOD	<LOD	0.06 ± 0.01^{j}	0.08 ± 0.01^{ef}	<LOD	<LOD
SN-FR-042	Z5	<LOD	<LOD	<LOD	<LOD	0.13 ± 0.01^{ghi}	0.18 ± 0.02^{bc}	<LOD	<LOD
SN-FR-043	Z5	<LOD	<LOD	<LOD	<LOD	0.15 ± 0.02^{fgh}	0.19 ± 0.02^{bc}	<LOD	0.010 ± 0.001^{ab}
SN-FR-044	Z5	<LOD	<LOD	<LOD	<LOD	0.13 ± 0.01^{ghi}	0.17 ± 0.02^{cd}	<LOD	<LOD
SN-FR-045	Z5	0.060 ± 0.007^{cd}	<LOD	<LOD	<LOD	0.15 ± 0.02^{fgh}	0.16 ± 0.02^{cd}	<LOD	<LOD
SN-FR-046	Z5	0.060 ± 0.006^{cd}	<LOD	<LOD	<LOD	0.16 ± 0.02^{fgh}	0.17 ± 0.02^{cd}	<LOD	0.006 ± 0.001^{cd}
SN-FR-047	Z5	<LOD	<LOD	0.007 ± 0.001^{ef}	<LOD	0.23 ± 0.03^{de}	0.18 ± 0.02^{bc}	<LOD	0.007 ± 0.001^{bc}

SN-FR-066	Z5	0.044 ± 0.004^{ef}	<LOD	<LOD	<LOD	0.13 ± 0.02^{ghi}	0.20 ± 0.02^{bc}	<LOD	0.007 ± 0.001^{bc}
SN-FR-067	Z5	<LOD	<LOD	<LOD	<LOD	0.16 ± 0.02^{fgh}	0.17 ± 0.02^{cd}	<LOD	0.006 ± 0.001^{cd}
SN-FR-068	Z5	0.050 ± 0.005^{de}	<LOD	<LOD	<LOD	0.12 ± 0.01^{hij}	0.23 ± 0.03^{ab}	<LOD	0.004 ± 0.001^{ef}
SN-FR-073	Z5	<LOD	<LOD	<LOD	<LOD	0.18 ± 0.02^{efg}	0.14 ± 0.02^{cd}	<LOD	0.006 ± 0.001^{cd}
SN-FR-074	Z5	<LOD	<LOD	<LOD	<LOD	0.18 ± 0.02^{efg}	0.15 ± 0.02^{cd}	<LOD	0.007 ± 0.001^{bc}
SN-FR-075	Z5	0.044 ± 0.004^{ef}	<LOD	<LOD	<LOD	0.14 ± 0.02^{ghi}	0.16 ± 0.02^{cd}	<LOD	0.006 ± 0.001^{cd}
SN-FR-076	Z5	<LOD	<LOD	<LOD	<LOD	0.18 ± 0.02^{efg}	0.25 ± 0.02^{ab}	<LOD	0.008 ± 0.001^{bc}
SN-FR-077	Z5	0.044 ± 0.006^{ef}	<LOD	<LOD	<LOD	0.18 ± 0.02^{efg}	0.18 ± 0.02^{bc}	<LOD	0.008 ± 0.001^{bc}
SN-FR-078	Z5	<LOD	<LOD	0.007 ± 0.001^{ef}	<LOD	0.11 ± 0.01^{hij}	0.17 ± 0.02^{cd}	<LOD	0.006 ± 0.001^{cd}
SN-FR-079	Z5	<LOD	<LOD	<LOD	<LOD	0.11 ± 0.01^{hij}	0.14 ± 0.02^{cd}	<LOD	0.008 ± 0.001^{bc}
SN-FR-080	Z5	0.043 ± 0.005^{ef}	<LOD	<LOD	<LOD	0.16 ± 0.02^{fgh}	0.22 ± 0.02^{b}	<LOD	0.007 ± 0.001^{bc}
SN-FR-092	Z5	0.060 ± 0.007^{cd}	<LOD	<LOD	<LOD	0.15 ± 0.01^{fgh}	0.22 ± 0.02^{b}	<LOD	<LOD
SN-FR-093	Z5	0.041 ± 0.005^{ef}	<LOD	<LOD	<LOD	0.10 ± 0.01^{hij}	0.15 ± 0.02^{cd}	<LOD	0.007 ± 0.001^{bc}

Notes: Values are reported as mean ± analytical SD of three independently prepared analytical portions on a dry-weight basis (mg/kg DW). The three analytical measurements were used only to assess analytical precision and were averaged to obtain one site-level concentration per matrix and georeferenced sampling site for subsequent statistical analyses. Values <LOD and <LOQ are indicated as applicable.

Table S31. Detection frequency and descriptive distribution of potentially toxic element concentrations in edible fruits.

Element	Detected *n*/94(%)	Mean ± SD (mg/kg DW)	Median [Q1–Q3]	CV (%)	Observed range (≥LOQ)	P90	P95	Skewness	Maximum; sample
Pb	35/94 (37.2)	0.0651 ± 0.0208	0.0640 [0.0440–0.0820]	31.9	0.035–0.105	0.0918	0.0949	0.117	0.105; SN-FR-065, Z2
Cd	18/94 (19.1)	0.00751 ± 0.00139	0.00765 [0.00713–0.00825]	18.5	0.0051–0.0104	0.00893	0.00921	−0.233	0.0104; SN-FR-087, Z2
As	19/94 (20.2)	0.00963 ± 0.00203	0.00900 [0.00850–0.01000]	21.1	0.007–0.014	0.0124	0.0140	0.873	0.014; SN-FR-014 and SN-FR-094, Z2
Hg	0/94 (0.0)	—	—	—	—	—	—	—	All samples <LOD
Cr	92/94 (97.9)	0.1560 ± 0.0807	0.140 [0.090–0.200]	51.8	0.060–0.460	0.288	0.315	1.091	0.460; SN-FR-064, Z2
Ni	89/94 (94.7)	0.1682 ± 0.0861	0.160 [0.090–0.220]	51.2	0.060–0.410	0.310	0.320	0.727	0.410; SN-FR-094, Z2
Sb	16/94 (17.0)	0.00411 ± 0.00084	0.00390 [0.00348–0.00453]	20.5	0.0031–0.0059	0.00525	0.00583	1.026	0.0059; SN-FR-050, Z2
Sn	59/94 (62.8)	0.00764 ± 0.00293	0.00700 [0.00600–0.00950]	38.3	0.004–0.016	0.0112	0.0131	0.774	0.016; SN-FR-065, Z2

Note: Elemental concentrations are expressed on a dry-weight basis (mg/kg DW). *Detected, n/94 (%)* represents fruit samples with concentrations ≥LOD, whereas descriptive concentration statistics were calculated using observations ≥LOQ. For elements affected by left-censoring, these descriptors characterize only the quantitatively determined fraction (≥LOQ), not the complete distribution across all 94 samples. Hg was <LOD in all samples; therefore, no concentration-based descriptors were calculated. Abbreviations: LOD, limit of detection; LOQ, limit of quantification; DW, dry weight.

Table S32. Rank-based spatial differentiation of potentially toxic elements in edible fruits with <LOD observations retained as tied lowest ranks.

Element	Kruskal–Wallis H (df = 4)	*p*	ε^2 effect size	Z2 vs. non-Z2 Mann–Whitney U	Rank-biserial *r*	*p*	Spatial-gradient Spearman r_s	*p*
Pb	56.27	<0.001	0.587	1537.5	0.830	<0.001	0.66	<0.001
Cd	48.97	<0.001	0.505	1390.5	0.655	<0.001	0.60	<0.001
As	53.32	<0.001	0.554	1427.0	0.699	<0.001	0.59	<0.001

Hg	—	—	—	—	—	—	—	—
Cr	78.76	<0.001	0.840	1650.5	0.965	<0.001	0.89	<0.001
Ni	78.51	<0.001	0.837	1607.5	0.914	<0.001	0.85	<0.001
Sb	55.01	<0.001	0.573	1400.0	0.667	<0.001	0.59	<0.001
Sn	62.64	<0.001	0.659	1553.0	0.849	<0.001	0.78	<0.001

Note: Elemental concentrations are expressed on a dry-weight basis (mg/kg DW). Observations <LOD were retained as tied lowest ranks without numerical substitution. Sector differences were evaluated using Kruskal–Wallis tests, while the predefined Z2 versus non-Z2 comparison used the Mann–Whitney U test. Spearman's coefficient assessed associations with the predefined spatial gradient Z4 < Z1 < Z5 < Z3 < Z2. Hg was excluded because all fruit concentrations were <LOD.

Table S33. Robustness assessment of sector effects for Cr and Ni concentrations in edible fruits.

Element	n included (≥LOQ)	Welch F (df1, df2)	Welch p	Kruskal–Wallis H	KW p
Cr	92	100.91 (4, 36.90)	<0.001	76.36	<0.001
Ni	89	111.95 (4, 40.93)	<0.001	72.64	<0.001

Note: Statistical analyses were performed using quantitatively reportable fruit concentrations (≥LOQ) only (n = 92 for Cr; n = 89 for Ni); observations <LOQ were excluded. Variance heterogeneity was significant for both elements (Brown–Forsythe test, $p < 0.001$); therefore, Welch's ANOVA was considered the preferred parametric analysis, with Games–Howell pairwise comparisons where applicable. Classical one-way ANOVA and Kruskal–Wallis tests were additionally reported as sensitivity analyses. Kruskal–Wallis statistics differ from those in Table S32 because Table S33 uses only ≥LOQ observations, whereas Table S32 retains all 94 samples and treats <LOD observations as tied lowest ranks. All p-values are two-sided, with statistical significance defined as $p < 0.05$.

Table S34. Pairwise co-detection associations among potentially toxic elements in edible fruits.

Element pair	Phi coefficient (φ)	Fisher's exact test *p*	FDR-adjusted *p*	Association strength
Cd–Sb	0.715	<0.001	<0.001	Strong
Cd–As	0.563	<0.001	<0.001	Moderate–strong
As–Sb	0.547	<0.001	<0.001	Moderate–strong
Pb–Sb	0.529	<0.001	<0.001	Moderate–strong
Pb–Cd	0.464	<0.001	<0.001	Moderate
Pb–As	0.434	<0.001	<0.001	Moderate
Pb–Sn	0.411	<0.001	<0.001	Moderate
As–Sn	0.388	<0.001	<0.001	Moderate
Sb–Sn	0.349	<0.001	<0.001	Moderate
Cd–Sn	0.319	0.002	0.004	Moderate
Ni–Sn	0.308	0.006	0.011	Moderate

Note: Pairwise co-detection was assessed across the 94 edible fruit samples from binary detection status (≥LOD vs. <LOD) using φ coefficients and two-sided Fisher's exact tests, with Benjamini–Hochberg correction for multiple comparisons. Hg was excluded because all samples were <LOD. Only associations remaining significant after FDR correction (adjusted $p < 0.05$) are presented.

Table S35. Sample-specific fresh- and dry-mass characteristics, dry-matter content, moisture content, and wet-weight concentrations of macroelements and biologically relevant micro/trace elements in edible *Sambucus nigra* L. fruits.

Sample/conversion data						Macroelements						Biologically relevant micro/trace elements							
Sample ID	Zone	Fresh mass (g)	Dry mass (g)	DM (%)	Mois-ture (%)	K mg/kg WW	Ca mg/kg WW	Mg mg/kg WW	Na mg/kg WW	P mg/kg WW	S mg/kg WW	Fe mg/kg WW	Mn mg/kg WW	Zn mg/kg WW	Cu mg/kg WW	B mg/kg WW	Mo mg/kg WW	Co mg/kg WW	Se mg/kg WW
SN-FR-022	Z1	116.30	23.88	20.53	79.47	752.54	461.38	193.83	9.03	246.81	334.48	7.39	3.47	2.36	1.01	3.53	0.04	0.0097	0.0099
SN-FR-023	Z1	173.23	37.66	21.74	78.26	798.72	505.45	164.57	15.87	330.23	415.88	7.83	3.63	2.43	1.02	4.00	0.04	0.0085	0.0137
SN-FR-026	Z1	155.97	30.27	19.41	80.59	726.43	425.41	195.82	14.75	258.12	328.76	6.02	2.66	1.98	0.94	3.53	0.03	0.0109	0.0056
SN-FR-027	Z1	154.35	34.14	22.12	77.88	685.68	467.59	170.31	9.29	262.77	455.86	7.08	2.99	2.23	1.02	3.56	0.07	0.0097	0.0073
SN-FR-029	Z1	134.67	26.31	19.54	80.46	624.39	392.88	175.83	15.24	205.72	312.00	7.62	2.75	2.29	0.90	3.18	0.05	0.0102	0.0131
SN-FR-053	Z1	148.19	31.61	21.33	78.67	649.73	519.40	207.76	16.85	292.23	473.97	7.89	3.22	2.13	0.96	3.67	0.05	0.0109	0.0141
SN-FR-061	Z1	142.29	30.54	21.46	78.54	694.33	504.39	181.15	11.38	283.96	439.57	8.37	3.28	3.07	0.97	4.06	0.05	0.0094	0.0114
SN-FR-062	Z1	138.47	30.16	21.78	78.22	679.56	412.31	193.41	12.20	320.61	318.44	6.97	3.22	3.01	0.98	3.72	0.07	0.0085	0.0100
SN-FR-063	Z1	161.85	34.83	21.52	78.48	714.68	420.07	190.02	10.98	264.26	406.08	7.96	2.86	2.52	1.02	3.94	0.03	0.0116	0.0101
SN-FR-081	Z1	129.95	25.41	19.55	80.45	719.97	415.12	164.25	15.64	250.68	355.09	7.43	3.17	1.97	0.91	3.38	0.05	0.0131	0.0084
SN-FR-082	Z1	155.23	32.72	21.08	78.92	723.41	507.78	195.40	14.97	297.42	321.66	8.01	3.58	2.42	1.13	3.92	0.04	0.0118	0.0156
SN-FR-083	Z1	156.28	32.46	20.77	79.23	686.25	416.24	153.49	15.79	267.73	413.33	7.27	3.24	2.16	1.05	3.45	0.03	0.0123	0.0112
SN-FR-084	Z1	141.27	27.93	19.77	80.23	731.71	428.23	176.95	14.04	245.55	296.56	7.71	3.78	1.64	1.01	3.14	0.05	0.0144	0.0103
SN-FR-003	Z2	157.16	33.74	21.47	78.53	949.77	575.57	225.85	18.03	485.83	590.60	8.80	4.96	2.92	1.36	3.50	0.06	0.0232	0.0112
SN-FR-004	Z2	140.71	29.61	21.04	78.96	780.71	489.47	234.21	17.47	405.29	491.99	9.89	4.19	2.48	1.24	4.04	0.08	0.0173	0.0114
SN-FR-014	Z2	108.63	23.52	21.65	78.35	827.74	497.77	244.66	17.10	467.89	518.34	8.88	4.53	2.58	1.27	3.98	0.08	0.0186	0.0121
SN-FR-018	Z2	159.39	30.19	18.94	81.06	807.64	530.73	211.19	16.67	357.42	551.56	9.09	3.60	2.61	1.34	3.88	0.05	0.0133	0.0114
SN-FR-019	Z2	192.54	38.41	19.95	80.05	882.75	505.31	234.00	22.94	387.41	529.25	8.58	3.05	3.17	1.24	3.71	0.07	0.0188	0.0158
SN-FR-020	Z2	130.32	28.67	22.00	78.00	953.47	574.19	247.06	15.40	501.15	620.61	10.56	4.20	3.56	1.33	3.92	0.06	0.0165	0.0090
SN-FR-048	Z2	119.06	24.23	20.35	79.65	887.31	537.47	233.63	15.87	439.79	538.49	9.36	4.19	2.83	1.38	3.64	0.04	0.0171	0.0104
SN-FR-049	Z2	147.58	30.76	20.84	79.16	781.40	535.25	226.15	20.22	434.16	569.01	9.80	3.06	3.17	1.02	4.52	0.07	0.0111	0.0131

SN-FR-050	Z2	171.46	37.29	21.75	78.25	911.70	492.60	237.28	21.10	412.13	526.75	10.00	4.83	2.81	1.31	3.83	0.06	0.0157	0.0126
SN-FR-051	Z2	177.18	36.91	20.83	79.17	915.15	583.71	207.90	20.21	386.64	545.38	8.75	3.94	3.29	1.16	3.67	0.06	0.0190	0.0148
SN-FR-052	Z2	136.59	29.31	21.46	78.54	908.12	584.96	249.13	20.81	409.86	594.83	11.16	4.01	2.60	1.13	3.80	0.06	0.0142	0.0118
SN-FR-054	Z2	151.25	33.23	21.97	78.03	895.73	584.41	214.43	19.11	406.01	612.97	8.57	4.46	3.63	1.32	4.15	0.06	0.0187	0.0106
SN-FR-055	Z2	130.64	28.41	21.75	78.25	889.44	493.87	255.09	23.49	432.54	627.18	10.00	4.28	2.48	1.23	4.13	0.07	0.0163	0.0104
SN-FR-056	Z2	142.41	28.28	19.86	80.14	859.46	555.23	208.51	13.90	404.31	567.15	8.34	4.45	2.92	1.14	3.18	0.07	0.0169	0.0135
SN-FR-057	Z2	187.26	40.99	21.89	78.11	979.11	566.28	220.64	22.76	491.85	530.16	10.07	4.62	2.85	1.14	3.85	0.09	0.0112	0.0140
SN-FR-059	Z2	129.51	26.16	20.20	79.80	756.06	546.59	208.46	20.60	401.76	581.13	9.29	3.27	3.21	1.13	3.15	0.06	0.0166	0.0160
SN-FR-064	Z2	149.64	32.16	21.49	78.51	950.79	612.72	200.52	24.72	413.93	624.33	9.46	3.93	2.97	1.41	3.65	0.07	0.0127	0.0178
SN-FR-065	Z2	122.45	24.96	20.38	79.62	856.12	471.89	210.36	14.47	472.50	487.99	9.78	4.12	2.69	1.28	4.04	0.04	0.0139	0.0133
SN-FR-069	Z2	129.99	26.70	20.54	79.46	869.25	488.85	236.21	17.66	466.46	573.68	9.04	4.48	2.77	1.20	3.66	0.06	0.0146	0.0138
SN-FR-085	Z2	148.57	31.88	21.46	78.54	914.96	546.10	258.35	21.03	487.31	611.34	9.87	3.80	2.98	1.09	4.23	0.07	0.0202	0.0116
SN-FR-086	Z2	180.18	35.55	19.73	80.27	772.24	552.84	216.24	20.72	353.76	522.06	9.08	3.63	2.88	1.19	3.37	0.06	0.0126	0.0093
SN-FR-087	Z2	129.19	26.82	20.76	79.24	836.84	540.18	193.69	21.18	377.21	510.08	9.13	4.50	3.18	1.13	3.61	0.06	0.0152	0.0104
SN-FR-088	Z2	169.78	37.78	22.25	77.75	970.20	499.79	234.54	20.03	491.33	618.39	10.24	4.78	2.96	1.34	4.45	0.05	0.0229	0.0147
SN-FR-094	Z2	165.80	36.26	21.87	78.13	960.52	595.29	215.42	17.28	481.13	586.33	8.75	3.81	2.58	1.30	4.07	0.09	0.0175	0.0088
SN-FR-001	Z3	172.34	35.76	20.75	79.25	607.76	509.20	180.94	19.71	334.90	455.04	8.71	4.11	2.24	1.21	3.42	0.04	0.0158	0.0077
SN-FR-005	Z3	133.73	28.89	21.60	78.40	601.00	418.24	203.29	15.12	299.64	299.42	8.64	3.74	2.72	1.08	3.15	0.05	0.0104	0.0084
SN-FR-006	Z3	123.21	24.37	19.78	80.22	828.55	485.58	177.22	19.98	289.57	306.97	7.71	2.69	2.43	1.06	2.71	0.05	0.0140	0.0099
SN-FR-007	Z3	144.76	28.60	19.76	80.24	649.21	441.17	148.77	16.20	313.74	483.25	8.10	3.26	2.23	1.00	3.48	0.04	0.0128	0.0081
SN-FR-008	Z3	160.74	34.01	21.16	78.84	784.76	497.43	233.38	15.02	305.74	497.86	8.46	3.39	2.62	1.11	3.39	0.05	0.0135	0.0108
SN-FR-032	Z3	119.01	24.84	20.87	79.13	704.44	471.29	235.86	15.24	334.16	411.39	8.56	3.34	2.55	1.04	3.21	0.03	0.0136	0.0075
SN-FR-033	Z3	153.89	32.92	21.39	78.61	612.45	463.99	231.03	16.04	292.43	504.63	8.34	3.81	2.72	0.91	3.40	0.03	0.0131	0.0045
SN-FR-034	Z3	115.52	25.66	22.21	77.79	751.23	506.23	238.56	15.55	365.62	352.96	8.89	4.35	3.02	1.06	3.84	0.06	0.0142	0.0080
SN-FR-035	Z3	175.59	33.52	19.09	80.91	762.07	474.96	138.97	18.71	210.37	411.58	8.02	3.25	2.48	0.90	3.95	0.03	0.0120	0.0075
SN-FR-036	Z3	151.54	33.61	22.18	77.82	686.88	537.17	217.58	22.62	234.21	342.89	8.21	3.66	2.44	1.14	3.48	0.03	0.0109	0.0138
SN-FR-037	Z3	162.03	33.36	20.59	79.41	782.37	528.51	219.06	15.03	253.45	323.45	6.59	3.54	3.15	1.01	2.78	0.05	0.0117	0.0107
SN-FR-038	Z3	197.00	38.65	19.62	80.38	719.64	480.08	224.84	16.09	251.72	436.33	8.24	3.98	2.55	0.89	3.41	0.05	0.0098	0.0094

SN-FR-039	Z3	147.70	32.39	21.93	78.07	941.66	444.51	162.50	8.55	307.01	385.08	8.11	3.99	2.87	0.99	3.40	0.05	0.0149	0.0081
SN-FR-040	Z3	159.17	31.20	19.60	80.40	781.91	471.81	162.89	12.74	271.88	398.50	7.45	3.78	1.74	0.80	3.04	0.05	0.0108	0.0102
SN-FR-041	Z3	167.44	34.69	20.72	79.28	890.04	509.45	150.41	20.10	267.26	411.04	7.46	3.29	2.44	0.89	3.15	0.04	0.0112	0.0095
SN-FR-058	Z3	174.12	36.32	20.86	79.14	895.69	449.10	216.94	9.39	320.40	399.66	8.55	2.92	2.80	0.90	3.32	0.04	0.0077	0.0146
SN-FR-060	Z3	169.44	37.55	22.16	77.84	898.42	538.52	210.97	10.42	357.68	394.47	8.42	2.57	2.62	1.07	3.86	0.06	0.0160	0.0080
SN-FR-089	Z3	143.29	28.77	20.08	79.92	751.32	413.61	186.12	21.48	300.57	436.50	9.04	3.79	2.37	1.09	3.59	0.05	0.0094	0.0078
SN-FR-090	Z3	175.65	37.64	21.43	78.57	857.59	525.22	165.00	21.21	230.58	385.51	8.36	3.54	3.04	1.01	3.62	0.04	0.0107	0.0111
SN-FR-091	Z3	120.86	27.16	22.47	77.53	867.65	525.40	207.64	21.12	346.52	478.88	7.64	3.53	2.49	1.10	3.60	0.05	0.0162	0.0092
SN-FR-002	Z4	154.53	33.73	21.83	78.17	499.63	336.14	164.14	4.58	327.41	354.26	6.77	4.43	2.23	0.91	3.56	0.04	0.0079	0.0079
SN-FR-009	Z4	192.10	40.80	21.24	78.76	520.78	320.92	183.50	9.35	223.65	293.73	6.37	3.57	1.95	1.03	3.74	0.04	0.0064	0.0066
SN-FR-010	Z4	173.93	33.66	19.35	80.65	497.75	432.72	118.63	5.61	322.61	350.09	6.00	3.54	2.32	0.79	3.97	0.04	0.0076	0.0056
SN-FR-011	Z4	151.35	31.87	21.06	78.94	543.70	434.20	163.61	9.27	245.74	320.70	5.90	3.35	2.53	0.79	3.98	0.05	0.0101	0.0078
SN-FR-012	Z4	139.74	30.30	21.68	78.32	583.49	499.36	149.83	10.84	319.61	389.43	7.16	4.45	2.28	1.08	4.34	0.06	0.0113	0.0100
SN-FR-013	Z4	141.50	28.91	20.43	79.57	646.64	402.08	175.09	11.85	335.27	332.82	5.72	3.84	2.41	0.92	3.45	0.05	0.0117	0.0078
SN-FR-015	Z4	162.18	35.78	22.06	77.94	543.61	340.42	141.86	12.13	257.46	297.84	7.50	3.46	2.07	0.80	3.88	0.05	0.0106	0.0071
SN-FR-016	Z4	164.72	32.78	19.90	80.10	414.92	299.70	144.48	6.97	225.27	217.11	6.17	3.82	2.65	0.87	3.68	0.04	0.0074	0.0096
SN-FR-017	Z4	112.32	22.81	20.31	79.69	434.80	340.97	132.81	11.17	279.84	282.28	6.09	3.35	2.17	0.76	3.64	0.02	0.0087	0.0069
SN-FR-021	Z4	176.67	39.49	22.35	77.65	539.59	456.66	155.35	12.07	311.82	252.58	6.26	3.71	2.26	0.90	4.07	0.04	0.0107	0.0081
SN-FR-024	Z4	138.57	30.24	21.82	78.18	600.78	490.58	131.16	13.97	262.31	358.33	7.42	3.71	2.12	0.79	3.71	0.03	0.0092	0.0085
SN-FR-025	Z4	140.44	26.74	19.04	80.96	386.90	325.21	150.80	10.66	200.49	235.53	6.09	3.47	2.21	0.83	3.69	0.04	0.0101	0.0109
SN-FR-028	Z4	126.60	25.41	20.07	79.93	528.47	352.25	176.63	11.64	299.46	229.61	6.62	2.61	1.91	0.83	3.29	0.03	0.0062	0.0094
SN-FR-030	Z4	165.59	34.01	20.54	79.46	556.19	457.60	162.26	10.27	283.02	262.28	7.60	3.02	2.44	0.73	3.68	0.04	0.0117	0.0090
SN-FR-031	Z4	148.29	33.48	22.58	77.42	672.58	384.72	160.98	10.61	237.74	324.21	7.45	4.06	2.42	0.96	3.86	0.04	0.0066	0.0079
SN-FR-070	Z4	137.64	27.45	19.94	80.06	536.68	348.41	171.11	8.97	257.27	282.40	6.38	3.03	1.85	0.77	3.49	0.04	0.0074	0.0074
SN-FR-071	Z4	184.61	40.98	22.20	77.80	525.87	439.08	174.26	12.43	280.58	386.69	7.55	3.53	2.46	1.05	4.33	0.06	0.0129	0.0104
SN-FR-072	Z4	159.85	33.90	21.21	78.79	502.40	425.63	145.06	6.36	295.84	283.54	7.21	3.75	2.61	0.88	3.54	0.04	0.0110	0.0110
SN-FR-042	Z5	155.48	33.75	21.71	78.29	946.42	581.96	199.70	23.44	330.60	528.35	8.47	3.67	2.58	1.14	3.41	0.05	0.0126	0.0159
SN-FR-043	Z5	160.14	32.35	20.20	79.80	727.44	518.56	185.04	12.93	250.90	568.26	7.88	3.33	2.85	1.01	3.19	0.05	0.0093	0.0095

SN-FR-044	Z5	180.83	38.86	21.49	78.51	789.11	527.14	218.55	14.83	360.60	611.60	7.95	2.86	2.90	1.19	4.06	0.07	0.0127	0.0114
SN-FR-045	Z5	135.81	29.62	21.81	78.19	793.23	565.75	235.76	16.14	260.19	582.98	8.94	3.10	2.79	1.24	3.49	0.05	0.0161	0.0107
SN-FR-046	Z5	149.89	29.51	19.69	80.31	833.19	527.24	226.02	17.33	363.44	498.89	7.88	3.66	2.38	1.15	3.33	0.06	0.0106	0.0136
SN-FR-047	Z5	127.82	25.93	20.29	79.71	811.45	381.38	219.50	10.95	281.98	465.37	8.72	3.75	2.68	1.07	3.51	0.04	0.0148	0.0120
SN-FR-066	Z5	160.83	35.48	22.06	77.94	602.03	496.36	200.75	23.38	297.38	451.80	8.60	3.46	2.49	1.32	3.97	0.05	0.0135	0.0102
SN-FR-067	Z5	160.19	33.11	20.67	79.33	661.62	477.05	159.57	20.26	372.87	532.03	9.09	3.41	2.98	1.14	4.03	0.06	0.0167	0.0118
SN-FR-068	Z5	170.43	37.44	21.97	78.03	884.43	477.80	203.20	19.77	377.85	626.75	9.01	2.97	3.03	1.10	3.65	0.05	0.0097	0.0092
SN-FR-073	Z5	121.75	26.94	22.13	77.87	753.43	594.34	228.80	17.04	276.59	392.98	9.29	3.52	3.76	1.08	3.78	0.06	0.0093	0.0115
SN-FR-074	Z5	168.87	32.79	19.42	80.58	548.73	416.50	200.58	13.79	265.82	471.26	8.16	3.86	2.66	1.00	3.28	0.04	0.0132	0.0085
SN-FR-075	Z5	160.95	34.49	21.43	78.57	718.94	393.22	237.65	13.71	269.36	386.15	9.00	5.29	2.96	1.15	4.07	0.06	0.0111	0.0086
SN-FR-076	Z5	114.79	24.44	21.29	78.71	747.10	548.24	163.30	10.65	315.32	591.89	8.52	4.09	3.09	1.09	3.79	0.05	0.0153	0.0115
SN-FR-077	Z5	117.32	26.66	22.72	77.28	905.10	544.24	245.42	12.27	256.78	531.97	9.54	3.77	2.73	1.18	3.50	0.07	0.0168	0.0120
SN-FR-078	Z5	174.54	38.82	22.24	77.76	976.17	484.86	205.73	20.91	270.01	578.27	8.67	4.38	2.45	1.09	3.89	0.06	0.0142	0.0085
SN-FR-079	Z5	112.89	23.99	21.25	78.75	824.96	471.98	220.16	15.73	307.29	545.51	9.56	4.27	2.38	1.06	3.32	0.04	0.0172	0.0138
SN-FR-080	Z5	149.45	30.40	20.34	79.66	797.58	478.63	220.30	11.19	354.14	492.46	8.14	3.91	2.81	1.09	3.21	0.07	0.0126	0.0092
SN-FR-092	Z5	147.80	32.35	21.89	78.11	621.39	495.97	177.07	17.73	379.53	404.70	7.66	3.85	2.47	1.02	4.38	0.05	0.0107	0.0083
SN-FR-093	Z5	184.47	36.25	19.65	80.35	697.80	488.32	203.58	17.10	280.03	518.78	7.66	3.52	2.71	0.93	3.44	0.05	0.0161	0.0092

Note: Fresh mass and dry mass are expressed in grams (g). Dry matter (DM) was calculated for each individual fruit sample as DM (%) = (dry mass/fresh mass) × 100, whereas moisture content was calculated as Moisture (%) = 100 − DM (%). Element concentrations are expressed as mg/kg wet weight (WW) and were obtained from the corresponding dry-weight (DW) concentrations using the sample-specific dry-matter fraction according to the equation: $C_{WW} = C_{DW} \times [DM (\%)/100]$, where C_{WW} is the concentration expressed on a wet-weight basis and C_{DW} is the corresponding concentration expressed on a dry-weight basis. Conversion was performed individually for each sample using its experimentally determined fresh and dry masses rather than a common moisture correction factor. Macroelements comprise K, Ca, Mg, Na, P, and S, whereas biologically relevant micro/trace elements comprise Fe, Mn, Zn, Cu, B, Mo, Co, and Se. DM = dry matter; DW = dry weight; WW = wet weight.

Table S36. Sample-specific fresh- and dry-mass characteristics, dry-matter content, moisture content, and wet-weight concentrations of lithogenic/geochemically associated and potentially toxic elements in edible *Sambucus nigra* L. fruits.

**Sample/conversion data**						**Lithogenic/ geochemically associated elements**								**Potentially toxic elements**							
Sample ID	Zone	Fresh mass (g)	Dry mass (g)	DM (%)	Moisture (%)	Al mg/kg WW	Ti mg/kg WW	V mg/kg WW	Ba mg/kg WW	Sr mg/kg WW	Li mg/kg WW	Rb mg/kg WW	Cs mg/kg WW	Pb mg/kg WW	Cd mg/kg WW	As mg/kg WW	Hg mg/kg WW	Cr mg/kg WW	Ni mg/kg WW	Sb mg/kg WW	Sn mg/kg WW
SN-FR-022	Z1	116.30	23.88	20.53	79.47	2.09	0.125	0.043	1.33	0.88	0.025	0.162	0.0031	<LOD	<LOD	<LOD	<LOD	0.018	0.018	<LOD	<LOD
SN-FR-023	Z1	173.23	37.66	21.74	78.26	2.43	0.150	0.041	1.63	1.17	0.039	0.202	0.0039	<LOD	<LOD	<LOD	<LOD	0.020	0.017	<LOD	<LOD
SN-FR-026	Z1	155.97	30.27	19.41	80.59	1.73	0.109	0.031	1.20	0.85	0.025	0.149	0.0021	<LOD	<LOD	<LOD	<LOD	0.023	0.016	<LOD	0.0008
SN-FR-027	Z1	154.35	34.14	22.12	77.88	2.01	0.119	0.040	1.19	0.88	0.029	0.133	0.0024	<LOD	<LOD	<LOD	<LOD	0.031	0.018	<LOD	0.0009
SN-FR-029	Z1	134.67	26.31	19.54	80.46	1.99	0.117	0.047	1.49	0.94	0.025	0.168	0.0031	<LOD	<LOD	<LOD	<LOD	0.023	0.018	<LOD	<LOD
SN-FR-053	Z1	148.19	31.61	21.33	78.67	1.75	0.100	0.041	1.47	1.00	0.034	0.156	0.0028	<LOD	<LOD	<LOD	<LOD	0.017	0.015	<LOD	<LOD

SN-FR-061	Z1	142.29	30.54	21.46	78.54	1.12	0.084	0.028	1.14	0.90	0.028	0.120	0.0024	<LOD	<LOD	<LOD	<LOD	0.019	0.021	<LOD	<LOD
SN-FR-062	Z1	138.47	30.16	21.78	78.22	1.11	0.072	0.028	1.24	0.91	0.024	0.137	0.0024	<LOD	<LOD	<LOD	<LOD	0.022	0.015	<LOD	<LOD
SN-FR-063	Z1	161.85	34.83	21.52	78.48	1.25	0.093	0.043	1.18	0.84	0.030	0.151	0.0032	<LOD	<LOD	<LOD	<LOD	0.022	0.022	<LOD	0.0009
SN-FR-081	Z1	129.95	25.41	19.55	80.45	1.45	0.102	0.029	1.06	0.96	0.031	0.133	0.0025	<LOD	<LOD	<LOD	<LOD	0.018	0.016	<LOD	0.0008
SN-FR-082	Z1	155.23	32.72	21.08	78.92	2.09	0.122	0.032	1.39	0.99	0.030	0.177	0.0032	<LOD	<LOD	<LOD	<LOD	0.015	0.013	<LOD	<LOD
SN-FR-083	Z1	156.28	32.46	20.77	79.23	2.20	0.096	0.046	1.25	0.93	0.033	0.160	0.0027	<LOD	<LOD	<LOD	<LOD	0.019	0.017	<LOD	<LOD
SN-FR-084	Z1	141.27	27.93	19.77	80.23	1.34	0.117	0.045	1.21	0.89	0.026	0.182	0.0024	<LOD	<LOD	<LOD	<LOD	0.016	0.016	<LOD	<LOD
SN-FR-003	Z2	157.16	33.74	21.47	78.53	2.32	0.167	0.052	2.08	1.33	0.036	0.236	0.0041	0.0185	0.0015	<LOD	<LOD	0.043	0.047	0.0010	0.0024
SN-FR-004	Z2	140.71	29.61	21.04	78.96	2.23	0.151	0.055	2.06	1.09	0.040	0.223	0.0046	<LOD	<LOD	0.0021	<LOD	0.065	0.046	<LOD	0.0023

SN-FR-014	Z2	108.63	23.52	21.65	78.35	2.27	0.119	0.048	1.69	1.32	0.037	0.249	0.0041	0.0164	0.0017	0.0030	<LOD	0.063	0.054	0.0010	0.0011
SN-FR-018	Z2	159.39	30.19	18.94	81.06	1.84	0.119	0.044	1.69	1.12	0.044	0.201	0.0032	0.0178	<LOD	0.0015	<LOD	0.038	0.057	<LOD	0.0015
SN-FR-019	Z2	192.54	38.41	19.95	80.05	2.01	0.124	0.060	1.62	1.08	0.036	0.213	0.0046	0.0074	0.0015	0.0022	<LOD	0.066	0.072	<LOD	0.0010
SN-FR-020	Z2	130.32	28.67	22.00	78.00	2.44	0.189	0.053	2.07	1.12	0.044	0.253	0.0035	0.0132	0.0014	0.0020	<LOD	0.051	0.051	0.0008	0.0018
SN-FR-048	Z2	119.06	24.23	20.35	79.65	2.01	0.153	0.055	1.79	1.10	0.041	0.181	0.0041	0.0183	0.0016	0.0020	<LOD	0.045	0.043	0.0007	0.0022
SN-FR-049	Z2	147.58	30.76	20.84	79.16	2.42	0.183	0.065	1.73	1.17	0.044	0.208	0.0037	0.0160	<LOD	0.0021	<LOD	0.044	0.046	0.0012	0.0023
SN-FR-050	Z2	171.46	37.29	21.75	78.25	2.76	0.146	0.057	1.96	1.39	0.035	0.263	0.0050	0.0202	0.0019	<LOD	<LOD	0.043	0.063	0.0013	0.0022
SN-FR-051	Z2	177.18	36.91	20.83	79.17	2.23	0.125	0.058	1.62	1.37	0.040	0.212	0.0035	<LOD	0.0016	0.0019	<LOD	0.046	0.031	0.0006	0.0023
SN-FR-052	Z2	136.59	29.31	21.46	78.54	2.36	0.185	0.049	1.70	1.20	0.039	0.182	0.0045	0.0150	0.0019	0.0019	<LOD	0.064	0.043	0.0010	0.0019

SN-FR-054	Z2	151.25	33.23	21.97	78.03	2.31	0.158	0.053	1.78	1.10	0.053	0.224	0.0050	0.0189	0.0011	<LOD	<LOD	0.048	0.059	0.0008	0.0026
SN-FR-055	Z2	130.64	28.41	21.75	78.25	2.48	0.144	0.067	1.61	1.17	0.046	0.241	0.0039	<LOD	<LOD	<LOD	<LOD	0.070	0.076	<LOD	0.0009
SN-FR-056	Z2	142.41	28.28	19.86	80.14	1.97	0.141	0.058	1.61	1.09	0.034	0.207	0.0030	0.0159	<LOD	0.0018	<LOD	0.060	0.062	0.0008	0.0028
SN-FR-057	Z2	187.26	40.99	21.89	78.11	2.32	0.190	0.063	1.82	1.09	0.048	0.254	0.0042	0.0212	0.0017	<LOD	<LOD	0.044	0.048	0.0008	0.0024
SN-FR-059	Z2	129.51	26.16	20.20	79.80	2.20	0.141	0.055	1.94	1.11	0.040	0.208	0.0040	0.0129	0.0017	0.0020	<LOD	0.059	0.059	<LOD	0.0020
SN-FR-064	Z2	149.64	32.16	21.49	78.51	2.28	0.138	0.054	1.76	1.18	0.049	0.176	0.0039	0.0163	<LOD	<LOD	<LOD	0.099	0.067	0.0009	0.0030
SN-FR-065	Z2	122.45	24.96	20.38	79.62	2.08	0.161	0.057	1.65	1.06	0.043	0.196	0.0039	0.0214	0.0015	0.0020	<LOD	0.053	0.035	<LOD	0.0033
SN-FR-069	Z2	129.99	26.70	20.54	79.46	2.28	0.148	0.060	1.70	1.17	0.041	0.210	0.0043	0.0156	0.0019	0.0019	<LOD	0.041	0.064	0.0008	0.0016
SN-FR-085	Z2	148.57	31.88	21.46	78.54	2.02	0.180	0.060	1.78	1.48	0.043	0.197	0.0041	0.0152	<LOD	<LOD	<LOD	0.058	0.069	<LOD	0.0026

SN-FR-086	Z2	180.18	35.55	19.73	80.27	2.29	0.120	0.045	1.60	1.10	0.039	0.185	0.0043	0.0178	<LOD	0.0014	<LOD	0.043	0.071	<LOD	0.0018
SN-FR-087	Z2	129.19	26.82	20.76	79.24	2.06	0.160	0.050	1.83	1.29	0.042	0.220	0.0046	0.0154	0.0022	0.0025	<LOD	0.066	0.064	0.0007	0.0019
SN-FR-088	Z2	169.78	37.78	22.25	77.75	1.98	0.200	0.067	2.07	1.25	0.044	0.211	0.0049	0.0116	0.0016	0.0018	<LOD	0.042	0.071	0.0008	0.0029
SN-FR-094	Z2	165.80	36.26	21.87	78.13	2.21	0.151	0.048	1.55	1.25	0.044	0.197	0.0039	0.0184	0.0015	0.0031	<LOD	0.072	0.090	0.0007	0.0009
SN-FR-001	Z3	172.34	35.76	20.75	79.25	2.41	0.122	0.037	1.43	1.08	0.037	0.187	0.0031	<LOD	<LOD	<LOD	<LOD	0.031	0.031	<LOD	0.0014
SN-FR-005	Z3	133.73	28.89	21.60	78.40	2.31	0.156	0.056	1.62	0.99	0.037	0.199	0.0037	<LOD	<LOD	<LOD	<LOD	0.045	0.026	<LOD	0.0013
SN-FR-006	Z3	123.21	24.37	19.78	80.22	2.06	0.133	0.045	1.40	1.11	0.036	0.214	0.0032	0.0079	<LOD	<LOD	<LOD	0.026	0.028	<LOD	0.0012
SN-FR-007	Z3	144.76	28.60	19.76	80.24	1.58	0.111	0.038	1.26	1.01	0.028	0.166	0.0036	<LOD	<LOD	<LOD	<LOD	0.032	0.022	<LOD	0.0016
SN-FR-008	Z3	160.74	34.01	21.16	78.84	1.97	0.125	0.053	1.63	1.25	0.042	0.167	0.0047	<LOD	<LOD	<LOD	<LOD	0.036	0.044	<LOD	0.0015

SN-FR-032	Z3	119.01	24.84	20.87	79.13	1.94	0.121	0.048	1.65	1.09	0.033	0.209	0.0033	<LOD	<LOD	<LOD	<LOD	0.021	0.019	<LOD	0.0010
SN-FR-033	Z3	153.89	32.92	21.39	78.61	2.07	0.135	0.045	1.84	1.01	0.036	0.167	0.0039	<LOD	<LOD	<LOD	<LOD	0.019	0.024	<LOD	0.0009
SN-FR-034	Z3	115.52	25.66	22.21	77.79	2.31	0.138	0.049	1.47	1.24	0.033	0.200	0.0044	<LOD	0.0011	<LOD	<LOD	0.022	0.022	<LOD	<LOD
SN-FR-035	Z3	175.59	33.52	19.09	80.91	1.58	0.111	0.048	1.26	1.07	0.038	0.214	0.0034	<LOD	0.0010	<LOD	<LOD	0.034	0.025	<LOD	0.0013
SN-FR-036	Z3	151.54	33.61	22.18	77.82	2.06	0.135	0.058	1.77	1.22	0.031	0.184	0.0038	<LOD	<LOD	<LOD	<LOD	0.029	0.035	<LOD	<LOD
SN-FR-037	Z3	162.03	33.36	20.59	79.41	1.98	0.130	0.039	1.59	1.19	0.035	0.218	0.0035	0.0089	<LOD	<LOD	<LOD	0.025	0.035	<LOD	<LOD
SN-FR-038	Z3	197.00	38.65	19.62	80.38	2.00	0.100	0.035	1.28	1.18	0.031	0.153	0.0033	<LOD	<LOD	<LOD	<LOD	0.027	0.039	<LOD	0.0014
SN-FR-039	Z3	147.70	32.39	21.93	78.07	1.69	0.143	0.042	1.43	1.12	0.037	0.169	0.0031	0.0079	<LOD	<LOD	<LOD	0.037	0.029	<LOD	<LOD
SN-FR-040	Z3	159.17	31.20	19.60	80.40	1.78	0.127	0.045	1.45	0.94	0.027	0.212	0.0039	<LOD	<LOD	<LOD	<LOD	0.025	0.033	<LOD	0.0008

SN-FR-041	Z3	167.44	34.69	20.72	79.28	1.86	0.095	0.052	1.74	1.16	0.031	0.211	0.0033	<LOD	<LOD	<LOD	<LOD	0.031	0.023	<LOD	0.0012
SN-FR-058	Z3	174.12	36.32	20.86	79.14	1.67	0.100	0.046	1.77	1.04	0.033	0.215	0.0031	0.0073	<LOD	<LOD	<LOD	0.033	0.035	<LOD	0.0013
SN-FR-060	Z3	169.44	37.55	22.16	77.84	2.39	0.133	0.055	1.42	1.17	0.044	0.184	0.0049	<LOD	<LOD	<LOD	<LOD	0.051	0.033	<LOD	0.0015
SN-FR-089	Z3	143.29	28.77	20.08	79.92	2.11	0.133	0.052	1.57	1.08	0.042	0.163	0.0038	0.0084	<LOD	<LOD	<LOD	0.020	0.024	<LOD	0.0010
SN-FR-090	Z3	175.65	37.64	21.43	78.57	1.78	0.156	0.049	1.56	1.33	0.039	0.176	0.0043	<LOD	<LOD	<LOD	<LOD	0.028	0.039	<LOD	0.0015
SN-FR-091	Z3	120.86	27.16	22.47	77.53	2.07	0.148	0.058	1.73	1.28	0.036	0.240	0.0036	<LOD	<LOD	<LOD	<LOD	0.031	0.052	<LOD	0.0009
SN-FR-002	Z4	154.53	33.73	21.83	78.17	1.27	0.089	0.031	0.94	0.81	0.022	0.168	0.0028	<LOD	<LOD	<LOD	<LOD	0.013	0.015	<LOD	<LOD
SN-FR-009	Z4	192.10	40.80	21.24	78.76	1.36	0.081	0.028	1.06	0.76	0.025	0.138	0.0017	<LOD	<LOD	<LOD	<LOD	0.015	0.013	<LOD	<LOD
SN-FR-010	Z4	173.93	33.66	19.35	80.65	1.37	0.072	0.033	1.24	0.89	0.021	0.126	0.0025	<LOD	<LOD	<LOD	<LOD	0.012	0.014	<LOD	<LOD

SN-FR-011	Z4	151.35	31.87	21.06	78.94	1.41	0.093	0.038	1.12	0.82	0.025	0.150	0.0025	<LOD	<LOD	<LOD	<LOD	0.015	<LOD	<LOD	<LOD
SN-FR-012	Z4	139.74	30.30	21.68	78.32	1.24	0.080	0.037	1.37	0.72	0.028	0.165	0.0026	<LOD	<LOD	<LOD	<LOD	0.013	0.017	<LOD	<LOD
SN-FR-013	Z4	141.50	28.91	20.43	79.57	1.04	0.076	0.035	0.96	0.78	0.027	0.172	0.0024	<LOD	<LOD	<LOD	<LOD	<LOD	0.016	<LOD	<LOD
SN-FR-015	Z4	162.18	35.78	22.06	77.94	1.43	0.088	0.026	1.19	1.06	0.031	0.185	0.0033	<LOD	<LOD	<LOD	<LOD	0.015	0.018	<LOD	<LOD
SN-FR-016	Z4	164.72	32.78	19.90	80.10	1.49	0.092	0.030	0.90	0.68	0.022	0.123	0.0028	<LOD	<LOD	<LOD	<LOD	0.012	0.012	<LOD	<LOD
SN-FR-017	Z4	112.32	22.81	20.31	79.69	1.50	0.067	0.035	1.22	0.89	0.022	0.128	0.0020	<LOD	<LOD	<LOD	<LOD	0.012	<LOD	<LOD	<LOD
SN-FR-021	Z4	176.67	39.49	22.35	77.65	1.30	0.065	0.031	1.21	0.87	0.031	0.159	0.0034	<LOD	<LOD	<LOD	<LOD	0.016	0.018	<LOD	<LOD
SN-FR-024	Z4	138.57	30.24	21.82	78.18	1.35	0.083	0.031	0.96	0.87	0.024	0.190	0.0022	<LOD	<LOD	<LOD	<LOD	0.015	0.015	<LOD	<LOD
SN-FR-025	Z4	140.44	26.74	19.04	80.96	1.01	0.084	0.032	1.14	0.61	0.027	0.120	0.0019	<LOD	<LOD	<LOD	<LOD	0.013	<LOD	<LOD	<LOD

SN-FR-028	Z4	126.60	25.41	20.07	79.93	1.36	0.088	0.024	1.14	0.76	0.026	0.149	0.0026	<LOD	<LOD	<LOD	<LOD	0.014	<LOD	<LOD	<LOD
SN-FR-030	Z4	165.59	34.01	20.54	79.46	1.54	0.074	0.027	1.09	0.97	0.025	0.136	0.0027	<LOD	<LOD	<LOD	<LOD	0.014	0.014	<LOD	<LOD
SN-FR-031	Z4	148.29	33.48	22.58	77.42	1.83	0.084	0.034	1.02	0.81	0.029	0.160	0.0032	<LOD	<LOD	<LOD	<LOD	0.018	0.016	<LOD	<LOD
SN-FR-070	Z4	137.64	27.45	19.94	80.06	1.48	0.068	0.030	0.94	0.62	0.028	0.140	0.0024	<LOD	<LOD	<LOD	<LOD	<LOD	<LOD	<LOD	<LOD
SN-FR-071	Z4	184.61	40.98	22.20	77.80	1.35	0.098	0.044	1.09	0.75	0.027	0.169	0.0022	<LOD	<LOD	<LOD	<LOD	0.013	0.013	<LOD	<LOD
SN-FR-072	Z4	159.85	33.90	21.21	78.79	1.46	0.089	0.028	1.06	0.83	0.023	0.115	0.0021	<LOD	<LOD	<LOD	<LOD	0.013	0.017	<LOD	<LOD
SN-FR-042	Z5	155.48	33.75	21.71	78.29	1.89	0.122	0.048	1.50	0.82	0.043	0.176	0.0030	<LOD	<LOD	<LOD	<LOD	0.028	0.039	<LOD	<LOD
SN-FR-043	Z5	160.14	32.35	20.20	79.80	1.86	0.115	0.046	1.19	1.09	0.030	0.166	0.0026	<LOD	<LOD	<LOD	<LOD	0.030	0.038	<LOD	0.0020
SN-FR-044	Z5	180.83	38.86	21.49	78.51	1.59	0.135	0.054	1.57	1.10	0.037	0.153	0.0037	<LOD	<LOD	<LOD	<LOD	0.028	0.037	<LOD	<LOD

SN-FR-045	Z5	135.81	29.62	21.81	78.19	1.96	0.105	0.039	1.70	1.00	0.039	0.183	0.0031	0.0131	<LOD	<LOD	<LOD	0.033	0.035	<LOD	<LOD
SN-FR-046	Z5	149.89	29.51	19.69	80.31	1.85	0.112	0.045	1.52	1.10	0.030	0.187	0.0026	0.0118	<LOD	<LOD	<LOD	0.032	0.033	<LOD	0.0012
SN-FR-047	Z5	127.82	25.93	20.29	79.71	1.95	0.130	0.039	1.44	0.87	0.034	0.162	0.0028	<LOD	<LOD	0.0014	<LOD	0.047	0.037	<LOD	0.0014
SN-FR-066	Z5	160.83	35.48	22.06	77.94	1.88	0.101	0.044	1.43	0.93	0.031	0.192	0.0031	0.0097	<LOD	<LOD	<LOD	0.029	0.044	<LOD	0.0015
SN-FR-067	Z5	160.19	33.11	20.67	79.33	2.00	0.126	0.043	1.55	1.01	0.031	0.172	0.0037	<LOD	<LOD	<LOD	<LOD	0.033	0.035	<LOD	0.0012
SN-FR-068	Z5	170.43	37.44	21.97	78.03	1.52	0.134	0.046	1.52	1.12	0.044	0.220	0.0035	0.0110	<LOD	<LOD	<LOD	0.026	0.051	<LOD	0.0009
SN-FR-073	Z5	121.75	26.94	22.13	77.87	1.79	0.131	0.035	1.48	1.11	0.031	0.175	0.0031	<LOD	<LOD	<LOD	<LOD	0.040	0.031	<LOD	0.0013
SN-FR-074	Z5	168.87	32.79	19.42	80.58	1.79	0.120	0.043	1.32	0.85	0.031	0.183	0.0025	<LOD	<LOD	<LOD	<LOD	0.035	0.029	<LOD	0.0014
SN-FR-075	Z5	160.95	34.49	21.43	78.57	1.67	0.152	0.049	1.69	1.07	0.032	0.169	0.0039	0.0094	<LOD	<LOD	<LOD	0.030	0.034	<LOD	0.0013

SN-FR-076	Z5	114.79	24.44	21.29	78.71	1.96	0.115	0.045	1.51	1.04	0.034	0.177	0.0038	<LOD	<LOD	<LOD	<LOD	0.038	0.053	<LOD	0.0017
SN-FR-077	Z5	117.32	26.66	22.72	77.28	1.91	0.139	0.041	1.73	1.02	0.034	0.204	0.0039	0.0100	<LOD	<LOD	<LOD	0.041	0.041	<LOD	0.0018
SN-FR-078	Z5	174.54	38.82	22.24	77.76	1.98	0.118	0.056	1.62	1.05	0.040	0.149	0.0044	<LOD	<LOD	0.0016	<LOD	0.024	0.038	<LOD	0.0013
SN-FR-079	Z5	112.89	23.99	21.25	78.75	2.08	0.115	0.047	1.23	1.02	0.032	0.172	0.0034	<LOD	<LOD	<LOD	<LOD	0.023	0.030	<LOD	0.0017
SN-FR-080	Z5	149.45	30.40	20.34	79.66	1.91	0.112	0.039	1.40	0.98	0.031	0.169	0.0035	0.0088	<LOD	<LOD	<LOD	0.033	0.045	<LOD	0.0014
SN-FR-092	Z5	147.80	32.35	21.89	78.11	1.90	0.123	0.053	1.42	1.01	0.031	0.184	0.0033	0.0131	<LOD	<LOD	<LOD	0.033	0.048	<LOD	<LOD
SN-FR-093	Z5	184.47	36.25	19.65	80.35	1.79	0.088	0.047	1.38	0.84	0.029	0.141	0.0029	0.0081	<LOD	<LOD	<LOD	0.020	0.029	<LOD	0.0014

Note: Fresh mass and dry mass are expressed in grams (g). Dry matter (DM) was calculated individually for each fruit sample as DM (%) = (dry mass/fresh mass) × 100, whereas moisture content was calculated as Moisture (%) = 100 − DM (%). Element concentrations are expressed as mg/kg wet weight (WW) and were obtained from the corresponding dry-weight (DW) concentrations using the sample-specific dry-matter fraction according to the equation: *CWW* = *CDW* × [DM (%)/100], where *CWW* represents the concentration expressed on a wet-weight basis and CDW represents the corresponding concentration expressed on a dry-weight basis. Conversion was performed separately for each sample using its experimentally determined fresh and dry masses, thereby accounting for sample-specific variation in moisture content. Lithogenic/geochemically associated elements comprise Al, Ti, V, Ba, Sr, Li, Rb, and Cs, whereas potentially toxic elements (PTEs) comprise Pb, Cd, As, Hg, Cr, Ni, Sb, and Sn. Values reported as <LOD indicate concentrations below the analytical limit of detection. DM = dry matter; DW = dry weight; WW = wet weight; LOD = limit of detection; PTEs = potentially toxic elements.

Table S37. Regulatory reference values, soil quality thresholds, and legally established maximum levels relevant to the investigated elements in soil and *Sambucus nigra* L. matrices.

**Matrix**	**Element**	**Normal/reference value**	**Alert threshold – sensitive use**	**Alert threshold – less-sensitive use**	**Intervention threshold – sensitive use**	**Intervention threshold – less-sensitive use**	**Food maximum level / MRL**	**Reporting basis**	**Regulatory framework and applicability**
Soil	Sb	5	12.5	20	20	40	—	mg/kg DM	Romanian Order No. 756/1997; directly relevant to soil assessment.
Soil	As	5	15	25	25	50	—	mg/kg DM	Romanian Order No. 756/1997.
Soil	Ba	200	400	1000	625	2000	—	mg/kg DM	Romanian Order No. 756/1997.
Soil	B*	1	2	5	3	10	—	mg/kg DM	Regulation refers specifically to soluble B; it should not be treated as directly equivalent to a total/pseudo-total B determination.
Soil	Cd	1	3	5	5	10	—	mg/kg DM	Romanian Order No. 756/1997.
Soil	Co	15	30	100	50	250	—	mg/kg DM	Romanian Order No. 756/1997.
Soil	Cr (total)	30	100	300	300	600	—	mg/kg DM	Threshold applies to total Cr. Romanian Order No. 756/1997.
Soil	Cu	20	100	250	200	500	—	mg/kg DM	Romanian Order No. 756/1997.
Soil	Mn	900	1500	2000	2500	4000	—	mg/kg DM	Romanian Order No. 756/1997.

Soil	Hg	0.10	1	4	2	10	—	mg/kg DM	Romanian Order No. 756/1997.
Soil	Mo	2	5	15	10	40	—	mg/kg DM	Romanian Order No. 756/1997.
Soil	Ni	20	75	200	150	500	—	mg/kg DM	Romanian Order No. 756/1997.
Soil	Pb	20	50	250	100	1000	—	mg/kg DM	Romanian Order No. 756/1997.
Soil	Se	1	3	10	5	20	—	mg/kg DM	Romanian Order No. 756/1997.
Soil	Sn	20	35	100	50	300	—	mg/kg DM	Romanian Order No. 756/1997.
Soil	V	50	100	200	200	400	—	mg/kg DM	Romanian Order No. 756/1997.
Soil	Zn	100	300	700	600	1500	—	mg/kg DM	Romanian Order No. 756/1997.
Soil	K, Ca, Mg, Na, P, S, Fe, Al, Ti, Sr, Li, Rb, Cs	—	—	—	—	—	—	mg/kg DM	No corresponding element-specific reference value is provided for these analytes in Table 1 of Romanian Order No. 756/1997.
Bark	All investigated elements	—	—	—	—	—	No directly applicable harmonized ML identified	mg/kg DW	Raw *S. nigra* bark, as sampled in this study, is not a food category for which the reviewed EU contaminant legislation establishes matrix-specific MLs.
Leaves	All investigated	—	—	—	—	—	No directly	mg/kg DW	The investigated *S. nigra* leaves should

	elements						applicable harmonized ML identified		not be compared with MLs established for edible leafy vegetables or herbs unless they are legally classified and marketed within such a food category.
Flowers	All investigated elements	—	—	—	—	—	No directly applicable harmonized ML identified	mg/kg DW	No direct matrix-specific EU ML was identified for raw *S. nigra* flowers as collected in the present environmental monitoring design.
Fruits – fresh elderberries	Pb	—	—	—	—	—	0.20 mg/kg	WW / fresh product	Directly applicable. Regulation (EU) 2023/915 explicitly lists cranberries, currants, elderberries, and strawberry-tree fruits at 0.20 mg/kg Pb.
Fruits – fresh elderberries	Cd	—	—	—	—	—	0.030 mg/kg	WW / fresh product	Directly applicable. Elderberries fall within berries and small fruits; the ML for this category, except specified exceptions such as raspberries, is 0.030 mg/kg Cd.
Fruits – fresh	Hg	—	—	—	—	—	0.010 mg/kg*	product basis	EU pesticide-residue

elder-berries									framework: mercury compounds, expressed as Hg. This is an MRL, not a contaminant ML under Regulation 2023/915, and should therefore be labeled separately.
Fruits – fresh elder-berries	As	—	—	—	—	—	No specific ML for fresh elder-berries	—	Regulation (EU) 2023/915 contains As MLs for specified food categories, but no directly applicable ML was identified for fresh elder-berries.
Fruits – fresh elder-berries	Cr	—	—	—	—	—	No specific ML	—	No harmonized EU ML directly applicable to fresh elderberries identified in the reviewed legislation.
Fruits – fresh elder-berries	Ni	—	—	—	—	—	No ML for the intact fresh berry	—	Regulation (EU) 2024/1987 introduced Ni MLs for several foods, but the 1.0 mg/kg value concerning small fruits and berries applies to specified fruit juices/nectars, not to raw elderberries.
Fruits – fresh	Sb	—	—	—	—	—	No specific ML	—	No directly applicable harmonized

elderberries									EU ML identified for fresh elderberries.
Fruits – fresh elderberries	Sn	—	—	—	—	—	No ML for fresh fruit	—	Tin MLs applicable to certain processed/canned food categories should not be transferred to untreated fresh elderberries.

Note: Values for soil are expressed as mg/kg dry matter (DM) and correspond to the normal/reference values, alert thresholds, and intervention thresholds established under Romanian Order No. 756/1997, with separate thresholds for sensitive and less-sensitive land uses, where applicable. Soil regulatory values are intended for environmental-quality assessment and should not be interpreted as food-safety maximum levels. Because the digestion procedure applied to the soil samples did not include hydrofluoric acid, the resulting concentrations represent an operationally defined acid-recoverable or pseudo-total elemental fraction rather than complete dissolution of silicate-bound elements. For *Sambucus nigra* fruits, legally established food-safety limits are expressed on a wet-weight (WW) or fresh-product basis. Therefore, dry-weight (DW) concentrations determined by ICP-MS should not be compared directly with these regulatory limits. In the present dataset, fruit concentrations were converted individually from DW to WW according to the experimentally determined dry-matter fraction of each sample using CWW = CDW × [DM (%)/100]. The maximum levels (MLs) for Pb and Cd refer to the food categories applicable to fresh elderberries under European Union legislation. The value reported for Hg represents a maximum residue level (MRL) under the EU pesticide-residue framework and is therefore legally and conceptually distinct from contaminant MLs; it is presented for regulatory context only and was not applied in the present compliance assessment. A dash (—) indicates that no directly applicable regulatory value was identified for the corresponding element–matrix combination within the regulatory frameworks considered. "No specific ML" indicates that an element may be regulated in other foods or product categories, but that such a value should not be transferred directly to untreated fresh *S. nigra* fruits. For bark, leaves, and flowers, no directly applicable harmonized maximum levels were identified for the raw *S. nigra* matrices investigated in the present environmental-monitoring design. Consequently, concentrations measured in these organs should be interpreted in terms of environmental exposure, spatial variation, and soil-to-plant transfer rather than regulatory food compliance. Regulatory limits established for other food commodities, herbal products, food supplements, or processed foods should not be applied unless the investigated material corresponds explicitly to the legally defined product category. Abbreviations: DM, dry matter; DW, dry weight; WW, wet weight; ML, maximum level; MRL, maximum residue level; PTE, potentially toxic element; LOD, limit of detection; —, no directly applicable regulatory value identified. Regulatory basis: Romanian Order No. 756/1997 for soil-quality reference and intervention thresholds; Commission Regulation (EU) 2023/915, as amended, for maximum levels of certain contaminants in food; and Regulation (EC) No. 396/2005, as amended, for pesticide maximum residue levels where applicable.

Table S38. Sector-specific middle-bound concentrations and estimated daily intakes of potentially toxic elements in edible *Sambucus nigra* L. fruits across the five ecological sectors.

Zone	n	Element	Detection n/N	Mean MB concentration (mg/kg WW)	Central EDI (mg/kg bw/day)
Z1	13	Pb	0/13	2.602×10^{-5}	3.717×10^{-8}
Z1	13	Cd	0/13	1.735×10^{-5}	2.478×10^{-8}
Z1	13	As	0/13	6.071×10^{-5}	8.673×10^{-8}
Z1	13	Hg	0/13	3.469×10^{-5}	4.956×10^{-8}
Z1	13	Cr	13/13	0.02023	2.890×10^{-5}
Z1	13	Ni	13/13	0.01708	2.440×10^{-5}
Z1	13	Sb	0/13	2.602×10^{-5}	3.717×10^{-8}

Z1	13	Sn	4/13	0.0003037	4.339×10^{-7}
Z2	24	Pb	21/24	0.01431	2.045×10^{-5}
Z2	24	Cd	16/24	0.001102	1.574×10^{-6}
Z2	24	As	17/24	0.001485	2.122×10^{-6}
Z2	24	Hg	0/24	3.503×10^{-5}	5.004×10^{-8}
Z2	24	Cr	24/24	0.05513	7.875×10^{-5}
Z2	24	Ni	24/24	0.05783	8.262×10^{-5}
Z2	24	Sb	16/24	0.0005877	8.395×10^{-7}
Z2	24	Sn	24/24	0.002071	2.958×10^{-6}
Z3	20	Pb	5/20	0.00204	2.914×10^{-6}
Z3	20	Cd	2/20	0.0001207	1.724×10^{-7}
Z3	20	As	0/20	6.099×10^{-5}	8.714×10^{-8}
Z3	20	Hg	0/20	3.485×10^{-5}	4.979×10^{-8}
Z3	20	Cr	20/20	0.03015	4.307×10^{-5}
Z3	20	Ni	20/20	0.0309	4.414×10^{-5}
Z3	20	Sb	0/20	2.614×10^{-5}	3.734×10^{-8}
Z3	20	Sn	16/20	0.001003	1.432×10^{-6}
Z4	18	Pb	0/18	2.622×10^{-5}	3.746×10^{-8}
Z4	18	Cd	0/18	1.748×10^{-5}	2.497×10^{-8}
Z4	18	As	0/18	6.119×10^{-5}	8.741×10^{-8}
Z4	18	Hg	0/18	3.496×10^{-5}	4.995×10^{-8}
Z4	18	Cr	16/18	0.01241	1.772×10^{-5}
Z4	18	Ni	13/18	0.01103	1.575×10^{-5}
Z4	18	Sb	0/18	2.622×10^{-5}	3.746×10^{-8}
Z4	18	Sn	0/18	6.119×10^{-5}	8.741×10^{-8}
Z5	19	Pb	9/19	0.005014	7.163×10^{-6}
Z5	19	Cd	0/19	1.764×10^{-5}	2.520×10^{-8}
Z5	19	As	2/19	0.0002131	3.044×10^{-7}
Z5	19	Hg	0/19	3.528×10^{-5}	5.041×10^{-8}
Z5	19	Cr	19/19	0.03174	4.534×10^{-5}
Z5	19	Ni	19/19	0.03826	5.466×10^{-5}
Z5	19	Sb	0/19	2.646×10^{-5}	3.780×10^{-8}
Z5	19	Sn	15/19	0.001145	1.636×10^{-6}

Note: Mean MB concentration is the arithmetic mean of the complete sector-specific wet-weight concentration distribution after middle-bound substitution of <LOD observations. Observations ≥LOQ retained their measured concentrations; no fruit PTE result between LOD and LOQ was used numerically. Central EDI was calculated as EDI = (C × 0.100)/70, where C is the sector-specific mean MB concentration (mg/kg WW), 0.100 is the assumed fruit ingestion rate (kg/day), and 70 is the assumed adult body weight (kg). Hg was <LOD in all 94 fruit samples; therefore, its MB concentrations arise entirely from the predefined censoring treatment rather than from detected Hg concentrations.

Table S39. Variance structure of the global principal component analysis of elemental profiles across soil and *Sambucus nigra* matrices.

Component	Eigenvalue	Explained variance (%)	Cumulative variance (%)
PC1	21.3339	70.9616	70.9616
PC2	4.5614	15.1724	86.1340
PC3	1.1939	3.9713	90.1053
PC4	0.6049	2.0121	92.1174
PC5	0.4745	1.5782	93.6956

Note: PCA was performed on the individual multi-element profiles of 470 samples, comprising soil, bark, leaves, flowers, and fruits (n = 94 per matrix). PC = principal component.

Table S40. Centroid scores of the five sample matrices on the first two principal components of the global PCA.

Sample matrix	n	PC1 centroid score	PC2 centroid score
Soil	94	8.675	−0.339
Bark	94	−0.850	−2.906
Leaves	94	−1.677	2.537
Flowers	94	−1.616	2.059
Fruits	94	−4.532	−1.352

Note: Centroid scores represent the mean PC1 and PC2 scores of the 94 individual samples belonging to each matrix. Positive and negative scores indicate the relative position of each matrix along the corresponding principal component and should not be interpreted as higher or lower elemental concentrations per se.

Table S41. Complete element loading matrix and relative contributions to PC1 and PC2 obtained from the global PCA of the 470 soil and plant samples.

Element	PC1 loading	PC1 contribution (%)	PC2 loading	PC2 contribution (%)
K	−0.363	0.616	0.914	18.322
Ca	0.544	1.387	0.709	11.035
Mg	−0.046	0.010	0.976	20.887
Na	0.885	3.675	−0.210	0.962
P	−0.754	2.666	0.623	8.521
S	−0.182	0.155	0.753	12.437
Fe	0.979	4.489	−0.137	0.412
Mn	0.952	4.246	0.098	0.210
Zn	0.871	3.554	0.222	1.085
Cu	0.949	4.222	0.093	0.188
B	−0.075	0.026	0.899	17.728
Mo	0.736	2.538	0.540	6.403
Co	0.975	4.458	0.037	0.030
Se	0.831	3.234	0.167	0.609
Al	0.985	4.548	−0.092	0.186
Ti	0.984	4.535	−0.103	0.232
V	0.992	4.612	−0.005	0.001
Ba	0.987	4.563	0.036	0.028
Sr	0.968	4.390	0.097	0.206
Li	0.976	4.465	0.006	0.001
Rb	0.980	4.503	0.003	<0.001
Cs	0.979	4.491	−0.007	0.001
Pb	0.831	3.238	0.075	0.122
Cd	0.845	3.348	0.047	0.047

As	0.754	2.663	0.112	0.274
Hg	0.781	2.857	0.001	<0.001
Cr	0.979	4.495	−0.048	0.051
Ni	0.952	4.246	−0.032	0.023
Sb	0.912	3.903	−0.002	<0.001
Sn	0.908	3.866	−0.002	<0.001
Total contribution	—	100.000	—	100.000

Note: Loadings indicate the direction and strength of the association between each element and the corresponding principal component. Relative contribution values quantify the percentage contribution of each variable to the respective component. PC1 and PC2 explained 70.9616% and 15.1724% of the total variance, respectively. Bold values may be used to highlight the elements showing the strongest associations with each component.

Table S42. Block normalization parameters of the multiple factor analysis of site-paired multi-matrix elemental profiles.

**Matrix block**	***n* sites**	**Active variables**	**First block eigenvalue (λ_1)**	**MFA block weight ($1/\sqrt{\lambda_1}$)**	**Variance explained by block PC1 (%)**
Soil	94	30	15.9817	0.2501	52.7056
Bark	94	30	18.2438	0.2341	60.1658
Leaves	94	30	13.1474	0.2758	43.3585
Flowers	94	30	14.9996	0.2582	49.4666
Fruits	94	29	15.2368	0.2562	51.9819

Note: MFA was performed using 94 site-paired sampling locations, with elemental variables organized into five matrix-specific blocks corresponding to soil, bark, leaves, flowers, and fruits. The first block eigenvalue (λ_1) represents the largest eigenvalue obtained from the separate PCA of each matrix block before global integration. In accordance with the MFA procedure, each block was weighted by the inverse square root of its first eigenvalue ($1/\sqrt{\lambda_1}$), thereby balancing the influence of the five matrices and preventing blocks with greater internal variance from disproportionately determining the global solution. The percentage of variance explained by block PC1 indicates the proportion of within-block variability represented by the first principal component of the corresponding matrix. Soil, bark, leaves, and flowers each contained 30 active elemental variables, whereas the fruit block contained 29 active variables because Hg was excluded owing to invariant concentrations below the analytical LOD in all fruit samples. MFA = multiple factor analysis; PC1 = first principal component; λ_1 = first block eigenvalue; LOD = limit of detection.

Table S43. Global variance structure and explained variance of the multiple factor analysis of site-paired multi-matrix elemental profiles.

**MFA dimension**	**Eigenvalue**	**Explained variance (%)**	**Cumulative variance (%)**
Dim1	4.8275	49.2042	49.2042
Dim2	0.4492	4.5789	53.7831
Dim3	0.3330	3.3946	57.1777
Dim4	0.2527	2.5760	59.7537
Dim5	0.2285	2.3286	62.0823

Note: MFA was performed on the 94 site-paired sampling locations, with elemental variables organized into five matrix-specific blocks: soil, bark, leaves, flowers, and fruits. Each block was normalized according to the MFA weighting procedure before construction of the global analysis to balance the influence of the five matrices. Eigenvalues describe the amount of multivariate variability represented by each global MFA dimension, while explained variance indicates the percentage of total variance accounted for by the

corresponding dimension. Cumulative variance represents the total percentage of variance explained up to and including the indicated dimension. Dim1 and Dim2 explained 49.2042% and 4.5789% of the total variance, respectively, corresponding to a cumulative explained variance of 53.7831%. The first five MFA dimensions together accounted for 62.0823% of the total variance. MFA = multiple factor analysis; Dim = global MFA dimension.

Table S44. Leading matrix-specific elemental variables contributing to the first two MFA dimensions.

Dimension	Rank	Matrix	Element	Association with dimension (*r*)	Contribution (%)
Dim1	1	Leaves	Na	0.912	1.324
Dim1	2	Leaves	Cr	0.867	1.196
Dim1	3	Leaves	S	0.849	1.149
Dim1	4	Flowers	Ni	0.906	1.146
Dim1	5	Flowers	Cr	0.898	1.126
Dim1	6	Flowers	Ba	0.878	1.077
Dim1	7	Bark	Cs	0.959	1.056
Dim1	8	Leaves	Li	0.811	1.048
Dim1	9	Leaves	K	−0.810	1.045
Dim1	10	Fruits	Ba	0.871	1.042
Dim2	1	Soil	Ti	0.602	5.095
Dim2	2	Leaves	B	−0.543	5.045
Dim2	3	Fruits	B	−0.548	4.440
Dim2	4	Leaves	Mo	−0.447	3.420
Dim2	5	Soil	Al	0.420	2.485
Dim2	6	Flowers	B	−0.406	2.478
Dim2	7	Flowers	Na	0.400	2.395
Dim2	8	Flowers	Ca	0.397	2.366
Dim2	9	Leaves	Sb	−0.369	2.336
Dim2	10	Leaves	Sr	0.363	2.256

Note: The table reports only the leading matrix-specific elemental variables contributing to the first two global MFA dimensions, rather than the complete set of active variables included in the analysis. Variables were ranked separately for Dim1 and Dim2 according to their relative contribution to the corresponding dimension, and the ten highest-contributing variables for each dimension are presented. The association coefficient (*r*) represents the direction and strength of the relationship between each standardized matrix-specific elemental variable and the corresponding global MFA dimension; positive and negative values indicate opposite directions along the axis. Contribution (%) quantifies the relative importance of each variable in defining that dimension. The complete MFA model included 149 active matrix-specific elemental variables distributed among soil, bark, leaves, flowers, and fruits; therefore, the variables shown here should be interpreted as the dominant contributors to Dim1 and Dim2 rather than as the only variables included in the analysis. Dim1 and Dim2 explained 49.2042% and 4.5789% of the total MFA variance, respectively. MFA = multiple factor analysis; Dim = global MFA dimension; *r* = Pearson correlation coefficient between the standardized variable and the corresponding MFA dimension.

Table S45. Integrated hierarchical-clustering structure and inferential validation of matrix- and ecological-zone-specific elemental signatures.

Matrix	Matrix × zone profiles included	HCA organization / relationship in integrated heatmap	Active elements (*n*)	PERMANOVA pseudo-*F*	R^2	PERMANOVA *p*	PERMDISP *F*	PERMDISP *p*	Integrated interpretation
Soil	Soil–Z1; Soil–Z2; Soil–Z3; Soil–Z4; Soil–Z5	Distinct soil-specific hierarchical group; the five zone profiles clustered separately from all plant matrices	30	31.223	0.584	<0.001	27.459	<0.001	Strong ecological-zone differentiation; interpretation should also account for significant heterogeneity of within-zone dispersion
Bark	Bark–Z1; Bark–Z2; Bark–Z3; Bark–Z4; Bark–Z5	Distinct bark-specific hierarchical group, clearly separated from soil, fruits, and the leaf–flower assemblage	30	34.379	0.607	<0.001	10.029	<0.001	Strongest zone-related multivariate differentiation among the

									investigated plant matrices, accompanied by heterogeneous dispersion
Leaves	Leaves–Z1; Leaves–Z2; Leaves–Z3; Leaves–Z4; Leaves–Z5	Leaf profiles occurred within the broader leaf–flower hierarchical assemblage, indicating comparatively similar standardized elemental signatures	30	21.049	0.486	<0.001	0.874	0.514	Significant ecological-zone differentiation with no evidence that the result was driven by unequal multivariate dispersion
Flowers	Flowers–Z1; Flowers–Z2; Flowers–Z3; Flowers–Z4;	Flower profiles intermingled with leaf profiles within the major canopy/reproductive-	30	25.727	0.536	<0.001	2.918	0.037	Significant ecological-zone differentiation,

	Flowers–Z5	tissue assemblage							although a modest but significant dispersion effect was also detected
Fruits	Fruits–Z1; Fruits–Z2; Fruits–Z3; Fruits–Z4; Fruits–Z5	Compact fruit-specific hierarchical group, separated from bark, soil, and the leaf–flower assemblage	29[1]	29.122	0.567	<0.001	2.343	0.095	Strong and robust ecological-zone differentiation without significant evidence of heterogeneous dispersion

Note: The integrated hierarchical analysis comprised 25 matrix × ecological-zone signatures generated from five matrices (soil, bark, leaves, flowers, and fruits) across five ecological sectors (Z1–Z5). Elemental concentrations were subjected to middle-bound treatment of left-censored observations (<LOD = LOD/2), log_{10} transformation, and unit-variance autoscaling prior to multivariate analysis. The heatmap was constructed from the mean standardized elemental profile of each matrix × zone combination. Hierarchical clustering was performed independently for the 25 profiles and for the elemental variables using Euclidean distance and Ward's linkage. PERMANOVA evaluated multivariate differentiation among the five ecological sectorsseparately within each matrix using 9,999 permutations, whereas PERMDISP assessed equality of multivariate dispersion among zones. R^2 represents the proportion of matrix-specific multivariate variation attributable to ecological zone. A significant PERMDISP result indicates that the corresponding PERMANOVA result may reflect both differences in multivariate centroid location and differences in within-zone dispersion. [1] Hg was excluded from the fruit-specific multivariate test because concentrations were <LOD in all 94 fruit samples, resulting in an invariant variable.

Table S46. Cross-matrix comparison of sector-level elemental patterns and Spearman rank associations with the predefined spatial gradient in soil and *Sambucus nigra* organs.

Group	Element	Soil	Bark	Leaves	Flowers	Fruits
Major elements	K	Z2^{a}; Z1/Z3/Z5^{b}; Z4^{c}r_s=0.74	Z2^{a}; Z5/Z3^{b}; Z1/Z4^{c}0.78	Z4^{a}; Z3^{b}; Z1/Z5^{c}; Z2^{d}–0.68	Z1/Z2/Z3/Z4/Z5^{a}0.21	Z2^{a}; Z3/Z5/Z1^{b}; Z4^{c}0.79
	Ca	Z2^{a}; Z1/Z3/Z5^{b}; Z4^{c}0.79	Z2^{a}; Z3^{b}; Z5^{bc}; Z1^{cd}; Z4^{d}0.75	Z4^{a}; Z3^{b}; Z5/Z1^{c}; Z2^{d}–0.57	Z3/Z2^{a}; Z1/Z4/Z5^{b}0.60	Z2^{a}; Z5/Z3/Z1^{b}; Z4^{c}0.69
	Mg	Z2^{a}; Z1/Z3/Z5^{b}; Z4^{c}0.76	Z2^{a}; Z3/Z5/Z1^{b}; Z4^{c}0.77	Z4^{a}; Z3^{ab}; Z5^{bc}; Z1^{cd}; Z2^{d}–0.49	Z3/Z2^{a}; Z1/Z4/Z5^{b}0.52	Z2^{a}; Z5^{b}; Z3^{bc}; Z1^{c}; Z4^{d}0.69
	Na	Z2^{a}; Z1/Z3/Z5^{b}; Z4^{c}0.80	Z2^{a}; Z3/Z5^{b}; Z1/Z4^{c}0.89	Z2^{a}; Z3^{b}; Z1^{c}; Z5^{d}; Z4^{e}0.93	Z3/Z2^{a}; Z1/Z5/Z4^{b}0.61	Z2^{a}; Z3^{ab}; Z5/Z1^{b}; Z4^{c}0.67
	P	Z2^{a}; Z1/Z3/Z5^{b}; Z4^{c}0.71	Z2^{a}; Z5/Z3/Z1^{b}; Z4^{c}0.69	Z4^{a}; Z3^{ab}; Z5^{bc}; Z1^{c}; Z2^{d}–0.57	Z2^{a}; Z3^{ab}; Z1/Z5^{bc}; Z4^{c}0.52	Z2^{a}; Z5/Z3/Z4/Z1^{b}0.68
	S	Z2^{a}; Z1/Z3/Z5^{b}; Z4^{c}0.75	Z2^{a}; Z3/Z5^{b}; Z1^{bc}; Z4^{c}0.68	Z2^{a}; Z3^{b}; Z5/Z1^{c}; Z4^{d}0.89	Z3/Z2^{a}; Z1^{ab}; Z4/Z5^{b}0.38	Z2^{a}; Z5^{b}; Z3/Z1^{c}; Z4^{d}0.71
Trace / biologically relevant	Fe	Z2^{a}; Z3^{ab}; Z5/Z1^{b}; Z4^{c}0.66	Z2^{a}; Z5/Z3^{b}; Z1/Z4^{c}0.76	Z2^{a}; Z5^{ab}; Z3^{bc}; Z1^{cd}; Z4^{d}0.62	Z2^{a}; Z3/Z5^{b}; Z1^{c}; Z4^{d}0.77	Z2^{a}; Z5/Z3^{b}; Z1^{c}; Z4^{d}0.81
	Mn	Z2^{a}; Z3^{ab}; Z5^{b}; Z4/Z1^{c}0.68	Z2^{a}; Z5/Z3^{b}; Z1/Z4^{c}0.72	Z1/Z2/Z3/Z4/Z5^{a}0.01	Z2^{a}; Z4/Z3/Z5/Z1^{b}0.38	Z2^{a}; Z5/Z4/Z3/Z1^{b}0.39
	Zn	Z2/Z5/Z3^{a}; Z1/Z4^{b}0.66	Z2^{a}; Z5^{ab}; Z3^{b}; Z1/Z4^{c}0.65	Z2^{a}; Z5/Z3^{ab}; Z1/Z4^{b}0.36	Z2/Z5/Z3^{a}; Z1/Z4^{b}0.60	Z2^{a}; Z5^{ab}; Z3^{bc}; Z1^{cd}; Z4^{d}0.57
	Cu	Z2^{a}; Z5^{ab}; Z3^{b}; Z1/Z4^{c}0.66	Z2^{a}; Z5^{b}; Z3^{bc}; Z1^{c}; Z4^{d}0.70	Z2^{a}; Z5/Z1/Z3^{b}; Z4^{c}0.59	Z2^{a}; Z5/Z3/Z1^{b}; Z4^{c}0.74	Z2^{a}; Z5^{b}; Z3/Z1^{c}; Z4^{d}0.72
	B	Z2^{a}; Z3^{ab}; Z5^{bc}; Z1/Z4^{c}0.67	Z2/Z3/Z5^{a}; Z1/Z4^{b}0.59	Z4^{a}; Z1^{ab}; Z2^{b}; Z5^{bc}; Z3^{c}–0.41	Z2^{a}; Z4/Z1/Z5^{ab}; Z3^{b}–0.01	Z2/Z4^{a}; Z1/Z5^{ab}; Z3^{b}0.004
	Mo	Z2^{a}; Z5^{ab}; Z3^{ac}; Z1^{bc}; Z4^{c}0.52	Z2/Z5^{a}; Z3^{b}; Z1^{bc}; Z4^{c}0.52	Z2^{a}; Z5^{ab}; Z1/Z4^{bc}; Z3^{c}0.34	Z2^{a}; Z5^{ab}; Z3/Z1^{bc}; Z4^{c}0.48	Z2^{a}; Z5^{ab}; Z1^{bc}; Z3/Z4^{c}0.48
	Co	Z2^{a}; Z5/Z3^{b}; Z1^{bc}; Z4^{c}0.65	Z2^{a}; Z5^{b}; Z3^{bc}; Z1^{cd}; Z4^{d}0.71	Z2^{a}; Z5/Z3/Z1^{ab}; Z4^{b}0.37	Z2^{a}; Z3/Z5^{b}; Z1^{bc}; Z4^{c}0.70	Z2^{a}; Z5/Z3^{b}; Z1^{bc}; Z4^{c}0.66
	Se	Z1^{a}; Z4/Z5/Z2/Z3^{b}–0.19; p=0.071	Z5/Z2^{a}; Z1/Z3^{ab}; Z4^{b}0.23	Z2^{a}; Z5/Z1^{ab}; Z3/Z4^{b}0.37	Z2^{a}; Z1/Z5^{ab}; Z3^{bc}; Z4^{c}0.33	Z2^{a}; Z1/Z5^{ab}; Z3^{bc}; Z4^{c}0.41
Lithogenic /	Al	Z4^{a}; Z3^{b}; Z5/Z1^{c}; Z2^{d}–0.68	Z2^{a}; Z5^{b}; Z3^{c}; Z1/Z4^{d}0.79	Z2^{a}; Z3^{ab}; Z1/Z5^{b}; Z4^{c}0.67	Z2^{a}; Z3^{ab}; Z5^{c}; Z1^{bcd}; Z4^{d}0.75	Z2^{a}; Z3/Z5/Z1^{b}; Z4^{c}0.76

geo-chemi-cal	Ti	Z3^{a}; Z5^{ab}; Z1^{b}; Z4^{c}; Z2^{d}–0.24	Z2^{a}; Z5/Z3^{b}; Z1/Z4^{c}0.81	Z2^{a}; Z3^{ab}; Z5/Z1^{b}; Z4^{c}0.74	Z2^{a}; Z3/Z5/Z1^{b}; Z4^{c}0.78	Z2^{a}; Z3^{b}; Z5^{bc}; Z1^{c}; Z4^{d}0.82
	V	Z2^{a}; Z4^{b}; Z3/Z5/Z1^{c}0.40	Z2^{a}; Z5^{b}; Z3^{bc}; Z1^{cd}; Z4^{d}0.82	Z2/Z3^{a}; Z5^{b}; Z1^{bc}; Z4^{c}0.72	Z2^{a}; Z3/Z5^{b}; Z1^{c}; Z4^{d}0.85	Z2^{a}; Z3/Z5^{b}; Z1^{c}; Z4^{d}0.81
	Ba	Z2^{a}; Z5/Z3^{b}; Z1/Z4^{c}0.75	Z2^{a}; Z5^{b}; Z3^{c}; Z1^{d}; Z4^{e}0.85	Z2^{a}; Z3^{ab}; Z5^{bc}; Z1^{c}; Z4^{d}0.78	Z2^{a}; Z3^{b}; Z5^{c}; Z1^{d}; Z4^{e}0.87	Z2^{a}; Z3/Z5^{b}; Z1^{c}; Z4^{d}0.86
	Sr	Z2/Z5/Z3^{a}; Z4/Z1^{b}0.64	Z2^{a}; Z5/Z3^{b}; Z1^{c}; Z4^{d}0.86	Z2/Z3^{a}; Z5/Z1^{b}; Z4^{c}0.76	Z2/Z3^{a}; Z5/Z1^{b}; Z4^{c}0.86	Z2/Z3^{a}; Z5/Z1^{b}; Z4^{c}0.83
	Li	Z4^{a}; Z3^{b}; Z5/Z1^{c}; Z2^{d}–0.72	Z2^{a}; Z5/Z3^{b}; Z1^{c}; Z4^{d}0.87	Z2^{a}; Z3^{b}; Z5^{bc}; Z1^{cd}; Z4^{d}0.80	Z2^{a}; Z3^{b}; Z5^{bc}; Z1^{cd}; Z4^{d}0.82	Z2^{a}; Z3/Z5^{b}; Z1/Z4^{c}0.83
	Rb	Z4^{a}; Z3^{b}; Z5^{c}; Z1^{d}; Z2^{e}–0.70	Z2^{a}; Z5/Z3^{b}; Z1^{c}; Z4^{d}0.86	Z2^{a}; Z3^{b}; Z5^{c}; Z1^{cd}; Z4^{d}0.76	Z2^{a}; Z3^{b}; Z5^{c}; Z1^{cd}; Z4^{d}0.81	Z2^{a}; Z3^{b}; Z5^{c}; Z1^{cd}; Z4^{d}0.76
	Cs	Z4^{a}; Z3^{b}; Z5^{bc}; Z1^{c}; Z2^{d}–0.74	Z2^{a}; Z5/Z3^{b}; Z1^{c}; Z4^{d}0.89	Z2/Z3^{a}; Z5^{b}; Z1^{bc}; Z4^{c}0.78	Z2/Z3^{a}; Z5^{b}; Z1^{bc}; Z4^{c}0.82	Z2^{a}; Z3^{b}; Z5^{c}; Z1/Z4^{d}0.81
Poten-tially toxic / envi-ron-men-tally rele-vant	Pb	Z2^{a}; Z5/Z3^{b}; Z1/Z4^{c}0.80	Z2 > Z5 > Z3 > Z1; Z4: all <LOD 0.69†	Z2 > Z5 > Z3 > Z4 > Z10.65†	Z2 > Z5 > Z3 > Z1 > Z40.83†	Z2 > Z5 > Z3; Z1/Z4: all <LOD0.66†
	Cd	Z2^{a}; Z3^{b}; Z5^{bc}; Z1^{c}; Z4^{d}0.80	Z2 > Z5 > Z3; Z1/Z4: all <LOD0.66†	Z2 > Z3 > Z5; Z1/Z4: all <LOD0.58†	Z2 > Z3 > Z5 > Z1; Z4: all <LOD0.86†	Z2 > Z3; Z1/Z4/Z5: all <LOD0.60†
	As	Z2 > Z3 > Z5 > Z1 > Z40.75†	Z2 > Z5 > Z3 > Z1; Z4: all <LOD 0.57†	Z2 > Z5 > Z3 > Z1 > Z40.54†	Z2 > Z5 > Z3 > Z1 > Z40.80†	Z2 > Z5; Z1/Z3/Z4: all <LOD0.59†
	Hg	Z2 > Z3 > Z5 > Z1 > Z40.79†	Z2 > Z5 > Z3; Z1/Z4: all <LOD0.29†	Z2 > Z5 > Z1; Z3/Z4: all <LOD 0.27†	Z2 > Z5; Z1/Z3/Z4: all <LOD0.40†	Z1/Z2/Z3/Z4/Z5: all <LOD —
	Cr	Z2^{a}; Z1^{ab}; Z3/Z5^{b}; Z4^{c}0.54	Z2^{a}; Z5^{b}; Z3^{bc}; Z1^{cd}; Z4^{d}0.79	Z2^{a}; Z5/Z3^{b}; Z1/Z4^{c}0.85	Z2^{a}; Z5/Z3^{b}; Z1^{c}; Z4^{d}0.87	Z2^{a}; Z5/Z3^{b}; Z1/Z4^{c}0.89†
	Ni	Z2^{a}; Z3^{ab}; Z5^{bc}; Z1^{c}; Z4^{d}0.71	Z2^{a}; Z5^{b}; Z3^{bc}; Z1^{cd}; Z4^{d}0.79	Z2^{a}; Z5^{b}; Z3^{c}; Z1/Z4^{d}0.80†	Z2^{a}; Z5/Z3^{b}; Z1^{c}; Z4^{d}0.87	Z2^{a}; Z5/Z3^{b}; Z1/Z4^{c}0.85†
	Sb	Z2^{a}; Z3^{b}; Z5^{bc}; Z1^{c}; Z4^{d}0.85	Z2 > Z5 > Z3; Z1/Z4: all <LOD0.61†	Z2 > Z5 > Z3 > Z1; Z4: all <LOD0.44†	Z2 > Z5 > Z3 > Z1; Z4: all <LOD0.83†	Z2; Z1/Z3/Z4/Z5: all <LOD0.59†
	Sn	Z2^{a}; Z3^{b}; Z5^{bc}; Z1^{c}; Z4^{d}0.82	Z2 > Z5 > Z3 > Z1 > Z40.62†	Z2 > Z5 > Z3 > Z1 > Z40.59†	Z2 > Z5 > Z3 > Z1 > Z40.84†	Z2 > Z5 > Z3 > Z1; Z4: all <LOD0.78†

Note: Z1–Z5 denote the five predefined ecological sectors, ordered along the spatial gradient Z4 < Z1 < Z5 < Z3 < Z2. Within each matrix, sectors sharing at least one superscript letter are not significantly different, whereas sectors without a common superscript letter differ significantly according to the post hoc multiple-comparison procedure described in the Statistical Analysis section. r_s denotes Spearman's rank correlation coefficient between elemental concentration and the predefined spatial gradient; positive values indicate increasing concentrations toward Z2, whereas negative values indicate increasing concentrations toward Z4. "All <LOD" indicates that all concentrations within the respective sector were below the analytical limit of detection. For extensively censored elements, sector sequences expressed using ">" represent descriptive concentration ordering and should not be interpreted as formal pairwise post hoc differences. † denotes correlations derived for elements affected by substantial left-censoring and should therefore be interpreted together with their detection frequencies. A dash (—) indicates that r_s could not be calculated because the element was not detected in any sample of the corresponding matrix. Elemental groups are provided for interpretative purposes only.

Table S47. Visual summary of the dominant cross-matrix spatial elemental signatures observed across the soil–*Sambucus nigra* continuum.

Elemental domain	Dominant sector pattern	Soil	Bark / Leaves / Flowers	Edible fruits	Central finding
Major elements: K, Ca, Mg, Na, P, S	Predominantly Z2 > Z3/Z5/Z1 > Z4	Z2-oriented for all six	Bark: Z2-oriented; Leaves: K, Ca, Mg, P reversed toward Z4, but Na and S remained Z2-oriented; Flowers: mainly Z2/Z3, while K was stable	Z2-oriented for all six	Strong organ-specific divergence, especially in leaves
Trace / biologically relevant elements: Fe, Mn, Zn, Cu, B, Mo, Co, Se	Generally, Z2 > intermediate sectors > Z4, with exceptions	Mostly Z2-oriented	Mostly positive toward Z2; exceptions included leaf Mn, leaf B, and weak/variable Se responses	Mostly Z2-oriented; B showed no monotonic gradient	Broad spatial coherence, but not universal across elements
Al, Li, Rb, Cs	Soil: Z4/Z3 > Z5/Z1 > Z2; Plant: generally, Z2/Z3 > Z5/Z1 > Z4	Strong inverse gradient	Clear positive Z2-oriented gradients in aerial organs	Positive Z2-oriented pattern	Most pronounced soil–plant spatial reversal
Ti, V, Ba, Sr	Soil mixed; aerial organs predominantly Z2/Z3 > Z5/Z1 > Z4	Ti inverse; V, Ba and Sr positive	Consistently positive spatial differentiation	Consistently positive spatial differentiation	Matrix dependence is strongest in soil
Pb, Cd, As, Sb, Sn	Predominantly Z2 > Z5/Z3 > Z1/Z4	Highest or among highest in Z2; lowest generally in Z4	Strong Z2 association; Z4 frequently showed no detections or very low detection frequencies	Strong concentration of detections in Z2; Pb, Cd, As and Sb were absent or rare in several other sectors	The PTE spatial signature extends into the edible compartment

Cr and Ni	Z2 > Z5/Z3 > Z1/Z4	Positive	Positive in bark → stronger in leaves → very strong in flowers	Very strong: r_s = 0.89 for Cr and 0.85 for Ni	Most coherent positive cross-matrix gradient in the study

Note: Z1–Z5 denote the five predefined ecological sectors arranged along the spatial gradient Z4 < Z1 < Z5 < Z3 < Z2. Sector sequences separated by ">" indicate the dominant descriptive spatial ordering observed across the corresponding elemental group. The terms Z2-oriented and positive spatial differentiation indicate increasing concentrations toward the upper end of the predefined gradient, particularly Z2, whereas inverse gradient indicates higher concentrations toward Z4 and/or Z3 and lower concentrations toward Z2. <LOD denotes a concentration below the analytical limit of detection. r_s denotes Spearman's rank correlation coefficient with the predefined spatial gradient. PTEs refers to potentially toxic elements. The table provides an integrated qualitative synthesis of the matrix- and organ-specific patterns derived from the detailed cross-matrix results and should be interpreted together with Table S46 and the corresponding statistical outputs.

Table S48. Complete-case within-site consistency of organ-specific partitioning of investigated elements across *Sambucus nigra* organs.

**Element**	**Complete profiles (*n*)**	**Friedman χ^2**	**Holm-adjusted *p***	**Kendall's *W***	**Most frequent highest-TF organ, *n* (%)**	**Most frequent lowest-TF organ, *n* (%)**
**Macroelements**						
K	94	282.000	<0.001	1.000	Leaves, 94 (100)	Bark, 94 (100)
Ca	94	280.813	<0.001	0.996	Leaves, 93 (98.9)	Fruits, 94 (100)
Mg	94	272.911	<0.001	0.968	Leaves, 88 (93.6)	Bark, 92 (97.9)
Na	94	126.122	<0.001	0.447	Bark, 53 (56.4)	Flowers, 45 (47.9)
P	94	282.000	<0.001	1.000	Flowers, 94 (100)	Bark, 94 (100)
S	94	182.131	<0.001	0.646	Fruits, 46 (48.9)	Bark, 94 (100)
**Microelements and biologically relevant trace elements**						
Fe	94	280.813	<0.001	0.996	Bark, 93 (98.9)	Fruits, 94 (100)
Mn	94	234.038	<0.001	0.830	Bark, 47 (50.0)	Fruits, 94 (100)
Zn	94	190.785	<0.001	0.677	Flowers, 44 (46.8)	Fruits, 94 (100)
Cu	94	226.123	<0.001	0.802	Flowers, 58 (61.7)	Fruits, 94 (100)
B	94	236.617	<0.001	0.839	Flowers, 48 (51.1)	Bark, 76 (80.9)
Mo	94	271.625	<0.001	0.963	Leaves, 84 (89.4)	Bark, 93 (98.9)

Co	94	249.671	<0.001	0.885	Leaves, 68 (72.3)	Fruits, 94 (100)
Se	94	170.845	<0.001	0.606	Flowers, 60 (63.8)	Bark, 47 (50.0)
**Lithogenic and geochemically associated elements**						
Al	94	263.313	<0.001	0.934	Bark, 91 (96.8)	Fruits, 94 (100)
Ti	94	279.651	<0.001	0.992	Bark, 92 (97.9)	Fruits, 94 (100)
V	94	233.501	<0.001	0.828	Bark, 51 (54.3)	Fruits, 94 (100)
Ba	94	209.571	<0.001	0.743	Bark, 61 (64.9)	Fruits, 94 (100)
Sr	94	184.916	<0.001	0.656	Bark, 57 (60.6)	Fruits, 94 (100)
Li	94	222.619	<0.001	0.789	Bark, 55 (58.5)	Fruits, 94 (100)
Rb	94	207.309	<0.001	0.735	Flowers, 47 (50.0)	Fruits, 94 (100)
Cs	94	221.450	<0.001	0.785	Bark, 60 (63.8)	Fruits, 94 (100)
**Potentially toxic and environmentally relevant elements**						
Pb	27	64.679	<0.001	0.799	Bark, 24 (88.9)	Fruits, 27 (100)
Cd	14	33.000	<0.001	0.786	Bark, 12 (85.7)	Fruits, 14 (100)
As	15	29.738	<0.001	0.661	Bark, 10 (66.7)	Fruits, 15 (100)
Hg	0	NE	NE	NE	NE	Fruits: all 94 samples <LOD
Cr	92	230.633	<0.001	0.836	Bark, 81 (88.0)	Fruits, 92 (100)
Ni	89	207.865	<0.001	0.779	Bark, 76 (85.4)	Fruits, 89 (100)
Sb	11	30.468	<0.001	0.923	Bark, 10 (90.9)	Fruits, 11 (100)
Sn	44	107.429	<0.001	0.814	Bark, 34 (77.3)	Fruits, 44 (100)

Note: Elemental concentrations were expressed on a dry-weight basis. Complete four-organ profiles included only sampling locations for which concentrations were ≥LOQ in the associated soil and simultaneously in bark, leaves, flowers, and fruits. Consequently, the number of complete profiles (*n*) varied among elements affected by left-censoring. The soil-to-organ concentration ratio (TF) was calculated as TF = Corgan/Csoil for each georeferenced sampling location. Organ rankings were established separately within each location using the four corresponding TF values. Differences among organs were evaluated separately for each element using

Friedman's test with three degrees of freedom. Kendall's *W* was calculated as a measure of effect size, and the resulting *p*-values were adjusted across the 29 evaluable elements using the Holm procedure. The highest- and lowest-TF classifications indicate the organs most frequently occupying the strict maximum and minimum positions, respectively; observations tied at an extreme position were not assigned to a single organ. Hg was not evaluated because concentrations were below LOD in all 94 fruit samples, preventing the construction of complete four-organ profiles. *n*, number of complete four-organ profiles; NE, not evaluated; LOD, limit of detection; LOQ, limit of quantification; TF, soil-to-organ concentration ratio.
